# Diretriz da SBC sobre Diagnóstico e Tratamento de Pacientes com Cardiomiopatia da Doença de Chagas – 2023

**DOI:** 10.36660/abc.20230269

**Published:** 2023-06-16

**Authors:** José Antonio Marin-Neto, Anis Rassi, Gláucia Maria Moraes Oliveira, Luís Claudio Lemos Correia, Alberto Novaes Ramos, Alejandro Ostermayer Luquetti, Alejandro Marcel Hasslocher-Moreno, Andréa Silvestre de Sousa, Angelo Amato Vincenzo de Paola, Antônio Carlos Sobral Sousa, Antonio Luiz Pinho Ribeiro, Dalmo Correia, Dilma do Socorro Moraes de Souza, Edecio Cunha-Neto, Felix Jose Alvarez Ramires, Fernando Bacal, Maria do Carmo Pereira Nunes, Martino Martinelli, Maurício Ibrahim Scanavacca, Roberto Magalhães Saraiva, Wilson Alves de Oliveira, Adalberto Menezes Lorga-Filho, Adriana de Jesus Benevides de Almeida Guimarães, Adriana Lopes Latado Braga, Adriana Sarmento de Oliveira, Alvaro Valentim Lima Sarabanda, Ana Yecê das Neves Pinto, Andre Assis Lopes do Carmo, Andre Schmidt, Andréa Rodrigues da Costa, Barbara Maria Ianni, Brivaldo Markman, Carlos Eduardo Rochitte, Carolina Thé Macêdo, Charles Mady, Christophe Chevillard, Cláudio Marcelo Bittencourt das Virgens, Cleudson Nery de Castro, Constança Felicia De Paoli de Carvalho Britto, Cristiano Pisani, Daniela do Carmo Rassi, Dário Celestino Sobral, Dirceu Rodrigues de Almeida, Edimar Alcides Bocchi, Evandro Tinoco Mesquita, Fernanda de Souza Nogueira Sardinha Mendes, Francisca Tatiana Pereira Gondim, Gilberto Marcelo Sperandio da Silva, Giselle de Lima Peixoto, Gustavo Glotz de Lima, Henrique Horta Veloso, Henrique Turin Moreira, Hugo Bellotti Lopes, Ibraim Masciarelli Francisco Pinto, João Marcos Bemfica Barbosa Ferreira, João Paulo Silva Nunes, José Augusto Soares Barreto-Filho, José Francisco Kerr Saraiva, Joseli Lannes-Vieira, Joselina Luzia Menezes Oliveira, Luciana Vidal Armaganijan, Luiz Cláudio Martins, Luiz Henrique Conde Sangenis, Marco Paulo Tomaz Barbosa, Marcos Antonio Almeida-Santos, Marcos Vinicius Simões, Maria Aparecida Shikanai Yasuda, Maria da Consolação Vieira Moreira, Maria de Lourdes Higuchi, Maria Rita de Cassia Costa Monteiro, Mauro Felippe Felix Mediano, Mayara Maia Lima, Maykon Tavares de Oliveira, Minna Moreira Dias Romano, Nadjar Nitz Silva Lociks de Araujo, Paulo de Tarso Jorge Medeiros, Renato Vieira Alves, Ricardo Alkmim Teixeira, Roberto Coury Pedrosa, Roque Aras, Rosalia Morais Torres, Rui Manoel dos Santos Povoa, Sergio Gabriel Rassi, Silvia Marinho Martins Alves, Suelene Brito do Nascimento Tavares, Swamy Lima Palmeira, Telêmaco Luiz da Silva, Thiago da Rocha Rodrigues, Vagner Madrini, Veruska Maia da Costa Brant, Walderez Ornelas Dutra, João Carlos Pinto Dias

**Affiliations:** 1 Universidade de São Paulo Faculdade de Medicina de Ribeirão Preto Ribeirão Preto SP Brasil Universidade de São Paulo , Faculdade de Medicina de Ribeirão Preto , Ribeirão Preto , SP – Brasil; 2 Hospital do Coração Anis Rassi Goiânia GO Brasil Hospital do Coração Anis Rassi , Goiânia , GO – Brasil; 3 Universidade Federal do Rio de Janeiro Rio de Janeiro RJ Brasil Universidade Federal do Rio de Janeiro , Rio de Janeiro , RJ – Brasil; 4 Escola Bahiana de Medicina e Saúde Pública Salvador BA Brasil Escola Bahiana de Medicina e Saúde Pública (EBMSP), Salvador , BA – Brasil; 5 Universidade Federal do Ceará Faculdade de Medicina Fortaleza CE Brasil Universidade Federal do Ceará , Faculdade de Medicina , Fortaleza , CE – Brasil; 6 Centro de Estudos da Doença de Chagas Hospital das Clínicas Universidade Federal de Goiás Goiânia GO Brasil Centro de Estudos da Doença de Chagas , Hospital das Clínicas da Universidade Federal de Goiás , Goiânia , GO – Brasil; 7 Instituto Nacional de Infectologia Evandro Chagas Fundação Oswaldo Cruz Rio de Janeiro RJ Brasil Instituto Nacional de Infectologia Evandro Chagas, Fundação Oswaldo Cruz , Rio de Janeiro , RJ – Brasil; 8 Universidade Federal de São Paulo São Paulo SP Brasil Universidade Federal de São Paulo , São Paulo , SP – Brasil; 9 Universidade Federal de Sergipe São Cristóvão SE Brasil Universidade Federal de Sergipe , São Cristóvão , SE – Brasil; 10 Hospital São Lucas Rede D`Or São Luiz Aracaju SE Brasil Hospital São Lucas , Rede D`Or São Luiz , Aracaju , SE – Brasil; 11 Universidade Federal de Minas Gerais Belo Horizonte MG Brasil Universidade Federal de Minas Gerais , Belo Horizonte , MG – Brasil; 12 Universidade Federal do Pará Belém PA Brasil Universidade Federal do Pará , Belém , PA – Brasil; 13 Universidade de São Paulo Faculdade de Medicina São Paulo SP Brasil Universidade de São Paulo , Faculdade de Medicina da Universidade, São Paulo , SP – Brasil; 14 Hospital das Clínicas Faculdade de Medicina Universidade de São Paulo São Paulo SP Brasil Instituto do Coração do Hospital das Clínicas da Faculdade de Medicina da Universidade de São Paulo , São Paulo , SP – Brasil; 15 Universidade de Pernambuco Faculdade de Ciências Médicas Recife PE Brasil Universidade de Pernambuco , Faculdade de Ciências Médicas , Recife , PE – Brasil; 16 Instituto de Moléstias Cardiovasculares São José do Rio Preto SP Brasil Instituto de Moléstias Cardiovasculares , São José do Rio Preto , SP – Brasil; 17 Hospital de Base de Rio Preto São José do Rio Preto SP Brasil Hospital de Base de Rio Preto , São José do Rio Preto , SP – Brasil; 18 Secretaria de Saúde do Distrito Federal Brasília DF Brasil Secretaria de Saúde do Distrito Federal , Brasília , DF – Brasil; 19 Escola Superior de Ciências da Saúde Brasília DF Brasil Escola Superior de Ciências da Saúde , Brasília , DF – Brasil; 20 Hospital Universitário Professor Edgard Santos Universidade Federal da Bahia Salvador BA Brasil Hospital Universitário Professor Edgard Santos , Universidade Federal da Bahia , Salvador , BA – Brasil; 21 Instituto de Cardiologia do Distrito Federal Brasília DF Brasil Instituto de Cardiologia do Distrito Federal , Brasília , DF – Brasil; 22 Hospital das Clínicas Universidade Federal de Minas Gerais Belo Horizonte MG Brasil Hospital das Clínicas da Universidade Federal de Minas Gerais , Belo Horizonte , MG – Brasil; 23 Hospital das Clínicas Universidade Federal de Pernambuco Recife PE Brasil Hospital das Clínicas da Universidade Federal de Pernambuco , Recife , PE – Brasil; 24 Hcor Associação Beneficente Síria São Paulo SP Brasil Hcor , Associação Beneficente Síria , São Paulo , SP – Brasil; 25 Hospital São Rafael Fundação Monte Tabor Salvador BA Brasil Hospital São Rafael , Fundação Monte Tabor , Salvador , BA – Brasil; 26 Institut National de la Santé Et de la Recherche Médicale Marselha França Institut National de la Santé Et de la Recherche Médicale (INSERM), Marselha – França; 27 Universidade de Brasília Brasília Distrito Federal Brasil Universidade de Brasília , Brasília , Distrito Federal – Brasil; 28 Instituto Oswaldo Cruz Fundação Oswaldo Cruz Rio de Janeiro RJ Brasil Instituto Oswaldo Cruz , Fundação Oswaldo Cruz , Rio de Janeiro , RJ – Brasil; 29 Universidade Federal de Goiás Faculdade de Medicina Goiânia GO Brasil Universidade Federal de Goiás , Faculdade de Medicina , Goiânia , GO – Brasil; 30 Hospital Universitário Antônio Pedro Faculdade Federal Fluminense Niterói RJ Brasil Hospital Universitário Antônio Pedro da Faculdade Federal Fluminense , Niterói , RJ – Brasil; 31 Hospital Universitário Walter Cantídio Universidade Federal do Ceará Fortaleza CE Brasil Hospital Universitário Walter Cantídio da Universidade Federal do Ceará, Fortaleza, CE – Brasil; 32 DentCor Clínica Médica e Odontológica Santo André SP Brasil DentCor Clínica Médica e Odontológica, Santo André, SP – Brasil; 33 Instituto de Cardiologia do Rio Grande do Sul Porto Alegre RS Brasil Instituto de Cardiologia do Rio Grande do Sul , Porto Alegre , RS – Brasil; 34 Hospital das Clínicas Faculdade de Medicina de Ribeirão Preto Universidade de São Paulo Ribeirão Preto SP Brasil Hospital das Clínicas , Faculdade de Medicina de Ribeirão Preto , Universidade de São Paulo , Ribeirão Preto , SP – Brasil; 35 Instituto Dante Pazzanese de Cardiologia São Paulo SP Brasil Instituto Dante Pazzanese de Cardiologia , São Paulo , SP – Brasil; 36 Grupo Fleury São Paulo SP Brasil Grupo Fleury , São Paulo , SP – Brasil; 37 Universidade do Estado do Amazonas Boca do Acre AM Brasil Universidade do Estado do Amazonas , Boca do Acre , AM – Brasil; 38 Hospital das Clínicas Faculdade de Medicina Universidade de São Paulo São Paulo SP Brasil Fundação Zerbini, Instituto do Coração do Hospital das Clínicas da Faculdade de Medicina da Universidade de São Paulo , São Paulo , SP – Brasil; 39 Sociedade Campineira de Educação e Instrução Campinas SP Brasil Sociedade Campineira de Educação e Instrução , Campinas , SP – Brasil; 40 Universidade Estadual de Campinas Faculdade de Ciências Médicas Campinas SP Brasil Universidade Estadual de Campinas , Faculdade de Ciências Médicas , Campinas , SP – Brasil; 41 Universidade Tiradentes Aracaju SE Brasil Universidade Tiradentes , Aracaju , SE – Brasil; 42 Organização Social de Saúde VivaRio Rio de Janeiro RJ Brasil Organização Social de Saúde VivaRio , Rio de Janeiro , RJ – Brasil; 43 Instituto Nacional de Cardiologia Rio de Janeiro RJ Brasil Instituto Nacional de Cardiologia (INC), Rio de Janeiro, RJ – Brasil; 44 Secretaria de Vigilância em Saúde Ministério da Saúde Brasília DF Brasil Secretaria de Vigilância em Saúde , Ministério da Saúde , Brasília , DF – Brasil; 45 Instituto René Rachou Fundação Oswaldo Cruz Belo Horizonte MG Brasil Instituto René Rachou , Fundação Oswaldo Cruz , Belo Horizonte , MG – Brasil; 46 Hospital Universitário Clementino Fraga Filho Instituto do Coração Edson Saad Universidade Federal do Rio de Janeiro RJ Brasil Hospital Universitário Clementino Fraga Filho , Instituto do Coração Edson Saad – Universidade Federal do Rio de Janeiro , RJ – Brasil; 47 Universidade Federal da Bahia Salvador BA Brasil Universidade Federal da Bahia (UFBA), Salvador , BA – Brasil; 48 Ambulatório de Doença de Chagas e Insuficiência Cardíaca Pronto Socorro Cardiológico Universitário Universidade de Pernambuco Recife PE Brasil Ambulatório de Doença de Chagas e Insuficiência Cardíaca do Pronto Socorro Cardiológico Universitário da Universidade de Pernambuco (PROCAPE/UPE), Recife , PE – Brasil; 49 Prefeitura Municipal de Goiânia Goiânia GO Brasil Prefeitura Municipal de Goiânia , Goiânia , GO – Brasil; 50 Cardion Uberlândia MG Brasil Cardion - Cardiologia Preventiva e Avançada, Uberlândia , MG – Brasil; 51 Hospital Felicio Rocho Belo Horizonte MG Brasil Hospital Felicio Rocho , Belo Horizonte , MG – Brasil

**Table:** 

Diretriz da SBC sobre Diagnóstico e Tratamento de Pacientes com Cardiomiopatia da Doença de Chagas – 2023	
O relatório abaixo lista as declarações de interesse conforme relatadas à SBC pelos especialistas durante o período de desenvolvimento deste posicionamento, 2021 a 2023.	
Especialista	Tipo de relacionamento com a indústria
Adalberto Menezes Lorga Filho	Nada a ser declarado
Adriana de Jesus Benevides de Almeida Guimarães	Nada a ser declarado
Adriana Lopes Latado Braga	Nada a ser declarado
Adriana Sarmento de Oliveira	Nada a ser declarado
Alberto Novaes Ramos Júnior	Nada a ser declarado
Alejandro Marcel Hasslocher-Moreno	Nada a ser declarado
Alejandro Ostermayer Luquetti	Nada a ser declarado
Alvaro Valentim Lima Sarabanda	Nada a ser declarado
Ana Yecê das Neves Pinto	Nada a ser declarado
Andre Assis Lopes do Carmo	Declaração financeira A - Pagamento de qualquer espécie e desde que economicamente apreciáveis, feitos a (i) você, (ii) ao seu cônjuge/ companheiro ou a qualquer outro membro que resida com você, (iii) a qualquer pessoa jurídica em que qualquer destes seja controlador, sócio, acionista ou participante, de forma direta ou indireta, recebimento por palestras, aulas, atuação como proctor de treinamentos, remunerações, honorários pagos por participações em conselhos consultivos, de investigadores, ou outros comitês, etc. Provenientes da indústria farmacêutica, de órteses, próteses, equipamentos e implantes, brasileiras ou estrangeiras: - Biosense Webster; Abbott. Outros relacionamentos Financiamento de atividades de educação médica continuada, incluindo viagens, hospedagens e inscrições para congressos e cursos, provenientes da indústria farmacêutica, de órteses, próteses, equipamentos e implantes, brasileiras ou estrangeiras: - Biosense Webster; Abbott.
Andre Schmidt	Nada a ser declarado
Andréa Rodrigues da Costa	Nada a ser declarado
Andréa Silvestre de Sousa	Nada a ser declarado
Angelo Amato Vincenzo de Paola	Nada a ser declarado
Anis Rassi Junior	Nada a ser declarado
Antônio Carlos Sobral Sousa	Nada a ser declarado
Antonio Luiz Pinho Ribeiro	Nada a ser declarado
Barbara Maria Ianni	Nada a ser declarado
Brivaldo Markman Filho	Nada a ser declarado
Carlos Eduardo Rochitte	Nada a ser declarado
Carolina Thé Macêdo	Nada a ser declarado
Charles Mady	Nada a ser declarado
Christophe Chevillard	Nada a ser declarado
Cláudio Marcelo Bittencourt das Virgens	Declaração financeira A - Pagamento de qualquer espécie e desde que economicamente apreciáveis, feitos a (i) você, (ii) ao seu cônjuge/ companheiro ou a qualquer outro membro que resida com você, (iii) a qualquer pessoa jurídica em que qualquer destes seja controlador, sócio, acionista ou participante, de forma direta ou indireta, recebimento por palestras, aulas, atuação como proctor de treinamentos, remunerações, honorários pagos por participações em conselhos consultivos, de investigadores, ou outros comitês, etc. Provenientes da indústria farmacêutica, de órteses, próteses, equipamentos e implantes, brasileiras ou estrangeiras: - Daiichi-Sankyo: Lixiana e Benicar Triplo; Pfizer: Eliquis; Astrazeneca: Forxiga; Novo Nordisk: Ozempic. Outros relacionamentos Financiamento de atividades de educação médica continuada, incluindo viagens, hospedagens e inscrições para congressos e cursos, provenientes da indústria farmacêutica, de órteses, próteses, equipamentos e implantes, brasileiras ou estrangeiras: - Novo Nordisk: Ozempic; Daiichi-Sankyo: Lixiana e Benicar Triplo.
Cleudson Nery de Castro	Nada a ser declarado
Constança Felicia De Paoli de Carvalho Britto	Nada a ser declarado
Cristiano Faria Pisani	Declaração financeira A - Pagamento de qualquer espécie e desde que economicamente apreciáveis, feitos a (i) você, (ii) ao seu cônjuge/ companheiro ou a qualquer outro membro que resida com você, (iii) a qualquer pessoa jurídica em que qualquer destes seja controlador, sócio, acionista ou participante, de forma direta ou indireta, recebimento por palestras, aulas, atuação como proctor de treinamentos, remunerações, honorários pagos por participações em conselhos consultivos, de investigadores, ou outros comitês, etc. Provenientes da indústria farmacêutica, de órteses, próteses, equipamentos e implantes, brasileiras ou estrangeiras: - Johnson & Jonhson; Biosense Webster.
Dalmo Correia Filho	Nada a ser declarado
Daniela do Carmo Rassi	Nada a ser declarado
Dário Celestino Sobral Filho	Nada a ser declarado
Dilma do Socorro Moraes de Souza	Declaração financeira B - Financiamento de pesquisas sob sua responsabilidade direta/pessoal (direcionado ao departamento ou instituição) provenientes da indústria farmacêutica, de órteses, próteses, equipamentos e implantes, brasileiras ou estrangeiras: - Novartis: biociência.
Dirceu Rodrigues de Almeida	Nada a ser declarado
Edecio Cunha Neto	Nada a ser declarado
Edimar Alcides Bocchi	Nada a ser declarado
Evandro Tinoco Mesquita	Declaração financeira A - Pagamento de qualquer espécie e desde que economicamente apreciáveis, feitos a (i) você, (ii) ao seu cônjuge/ companheiro ou a qualquer outro membro que resida com você, (iii) a qualquer pessoa jurídica em que qualquer destes seja controlador, sócio, acionista ou participante, de forma direta ou indireta, recebimento por palestras, aulas, atuação como proctor de treinamentos, remunerações, honorários pagos por participações em conselhos consultivos, de investigadores, ou outros comitês, etc. Provenientes da indústria farmacêutica, de órteses, próteses, equipamentos e implantes, brasileiras ou estrangeiras: - AVE; AstraZeneca; Pfizzer; Bayer; Anylan; Boeringer; Novo Nordisk; Norvartis. Outros relacionamentos Participação societária de qualquer natureza e qualquer valor economicamente apreciável de empresas na área de saúde, de ensino ou em empresas concorrentes ou fornecedoras da SBC: - Sócio da EC Tinoco. Vínculo empregatício com a indústria farmacêutica, de órteses, próteses, equipamentos e implantes, brasileiras ou estrangeiras, assim como se tem relação vínculo empregatício com operadoras de planos de saúde ou em auditorias médicas (incluindo meio período) durante o ano para o qual você está declarando: - UnitedHealth Group (UHG). Atuação no último ano como auditor médico para empresa operadora de planos de saúde ou assemelhada: - Vice-Presidente da Sociedad Interamericana de Cardiología (SIAC).
Felix Jose Alvarez Ramires	Declaração financeira A - Pagamento de qualquer espécie e desde que economicamente apreciáveis, feitos a (i) você, (ii) ao seu cônjuge/ companheiro ou a qualquer outro membro que resida com você, (iii) a qualquer pessoa jurídica em que qualquer destes seja controlador, sócio, acionista ou participante, de forma direta ou indireta, recebimento por palestras, aulas, atuação como proctor de treinamentos, remunerações, honorários pagos por participações em conselhos consultivos, de investigadores, ou outros comitês, etc. Provenientes da indústria farmacêutica, de órteses, próteses, equipamentos e implantes, brasileiras ou estrangeiras: - Novartis; Pfizer; AstraZeneca; Amgen.
Fernanda de Souza Nogueira Sardinha Mendes	Nada a ser declarado
Fernando Bacal	Outros relacionamentos Financiamento de atividades de educação médica continuada, incluindo viagens, hospedagens e inscrições para congressos e cursos, provenientes da indústria farmacêutica, de órteses, próteses, equipamentos e implantes, brasileiras ou estrangeiras: - Novartis: insuficiência cardíaca; Pfizer: amiloidose.
Francisca Tatiana Pereira Gondim	Nada a ser declarado
Gilberto Marcelo Sperandio da Silva	Nada a ser declarado
Giselle de Lima Peixoto	Nada a ser declarado
Gláucia Maria Moraes de Oliveira	Nada a ser declarado
Gustavo Glotz de Lima	Declaração financeira A - Pagamento de qualquer espécie e desde que economicamente apreciáveis, feitos a (i) você, (ii) ao seu cônjuge/ companheiro ou a qualquer outro membro que resida com você, (iii) a qualquer pessoa jurídica em que qualquer destes seja controlador, sócio, acionista ou participante, de forma direta ou indireta, recebimento por palestras, aulas, atuação como proctor de treinamentos, remunerações, honorários pagos por participações em conselhos consultivos, de investigadores, ou outros comitês, etc. Provenientes da indústria farmacêutica, de órteses, próteses, equipamentos e implantes, brasileiras ou estrangeiras: - Abbott: fibrilação atrial; Bayer: fibrilação atrial; Sankyo: anticoagulação.
Henrique Horta Veloso	Nada a ser declarado
Henrique Turin Moreira	Nada a ser declarado
Hugo Bellotti Lopes	Outros relacionamentos Financiamento de atividades de educação médica continuada, incluindo viagens, hospedagens e inscrições para congressos e cursos, provenientes da indústria farmacêutica, de órteses, próteses, equipamentos e implantes, brasileiras ou estrangeiras: - Abbott. Participação em comitês de compras de materiais ou fármacos em instituições de saúde ou funções assemelhadas: - Licitação de materiais OPME pelo SUS.
Ibraim Masciarelli Francisco Pinto	Outros relacionamentos Participação em órgãos governamentais de regulação, ou de defesa de direitos na área de cardiologia: - Delegado CFM.
João Carlos Pinto Dias	Nada a ser declarado
João Marcos Bemfica Barbosa Ferreira	Nada a ser declarado
João Paulo Silva-Nunes	Nada a ser declarado
Jose Antonio Marin Neto	Nada a ser declarado
José Augusto Soares Barreto-Filho	Nada a ser declarado
José Francisco Kerr Saraiva	Nada a ser declarado
Joseli Lannes Vieira	Nada a ser declarado
Joselina Luzia Menezes Oliveira	Nada a ser declarado
Luciana Vidal Armaganijan	Nada a ser declarado
Luis Cláudio Lemos Correia	Outros relacionamentos Participação em órgãos governamentais de regulação, ou de defesa de direitos na área de cardiologia: - Representante do CONASS no CONITEC.
Luiz Cláudio Martins	Nada a ser declarado
Luiz Henrique Conde Sangenis	Nada a ser declarado
Marco Paulo Tomaz Barbosa	Nada a ser declarado
Marcos Antonio Almeida-Santos	Nada a ser declarado
Marcus Vinicius Simões	Declaração financeira B - Financiamento de pesquisas sob sua responsabilidade direta/pessoal (direcionado ao departamento ou instituição) provenientes da indústria farmacêutica, de órteses, próteses, equipamentos e implantes, brasileiras ou estrangeiras: - MSD: insuficiência cardíaca; Novartis: hipercolesterolemia; Alnylam: amiloidose; IONIS: amiloidose; Behringer: insuficiência cardíaca. Outros relacionamentos Financiamento de atividades de educação médica continuada, incluindo viagens, hospedagens e inscrições para congressos e cursos, provenientes da indústria farmacêutica, de órteses, próteses, equipamentos e implantes, brasileiras ou estrangeiras: - BMS: cardiomiopatias.
Maria Aparecida Shikanai Yasuda	Nada a ser declarado
Maria Carmo Pereira Nunes	Nada a ser declarado
Maria da Consolação Vieira Moreira	Nada a ser declarado
Maria de Lourdes Higuchi	Nada a ser declarado
Maria Rita de Cassia Costa Monteiro	Nada a ser declarado
Martino Martinelli Filho	Declaração financeira B - Financiamento de pesquisas sob sua responsabilidade direta/pessoal (direcionado ao departamento ou instituição) provenientes da indústria farmacêutica, de órteses, próteses, equipamentos e implantes, brasileiras ou estrangeiras: - Impulse Dynamics Inc.
Mauricio Ibrahim Scanavacca	Declaração financeira B - Financiamento de pesquisas sob sua responsabilidade direta/pessoal (direcionado ao departamento ou instituição) provenientes da indústria farmacêutica, de órteses, próteses, equipamentos e implantes, brasileiras ou estrangeiras: - J&J: ablação por cateter de pacientes com taquicardia ventricular e Chagas; ABBOTT: denervação autonômica de pacientes com síncope vasovagal; Medtronic: crioablação de pacientes com insuficiência cardíaca e fração de ejeção do VE reduzida com fibrilação atrial.
Mauro Felippe Felix Mediano	Nada a ser declarado
Mayara Maia Lima	Nada a ser declarado
Maykon Tavares de Oliveira	Nada a ser declarado
Minna Moreira Dias Romano	Nada a ser declarado
Nadjar Nitz Silva Lociks de Araujo	Nada a ser declarado
Paulo de Tarso Jorge Medeiros	Nada a ser declarado
Renato Vieira Alves	Nada a ser declarado
Ricardo Alkmim Teixeira	Declaração financeira A - Pagamento de qualquer espécie e desde que economicamente apreciáveis, feitos a (i) você, (ii) ao seu cônjuge/ companheiro ou a qualquer outro membro que resida com você, (iii) a qualquer pessoa jurídica em que qualquer destes seja controlador, sócio, acionista ou participante, de forma direta ou indireta, recebimento por palestras, aulas, atuação como proctor de treinamentos, remunerações, honorários pagos por participações em conselhos consultivos, de investigadores, ou outros comitês, etc. Provenientes da indústria farmacêutica, de órteses, próteses, equipamentos e implantes, brasileiras ou estrangeiras: - Boehringer-Ingelheim: Pradaxa, Jardiance; Daiichi-Sankyo: Lixiana; Abbott: Dispositivos Cardíacos Eletrônicos Implantáveis; Biotronik: Dispositivos Cardíacos Eletrônicos Implantáveis; Medtronic: Dispositivos Cardíacos Eletrônicos Implantáveis; Biomedical: Extração de Cabos-Eletrodos de Dispositivos Cardíacos Eletrônicos Implantáveis. Outros relacionamentos Financiamento de atividades de educação médica continuada, incluindo viagens, hospedagens e inscrições para congressos e cursos, provenientes da indústria farmacêutica, de órteses, próteses, equipamentos e implantes, brasileiras ou estrangeiras: - Biomedical: Extração de Cabos-Eletrodos de Dispositivos Cardíacos Eletrônicos Implantáveis.
Roberto Coury Pedrosa	Nada a ser declarado
Roberto Magalhães Saraiva	Nada a ser declarado
Roque Aras Junior	Declaração financeira B - Financiamento de pesquisas sob sua responsabilidade direta/pessoal (direcionado ao departamento ou instituição) provenientes da indústria farmacêutica, de órteses, próteses, equipamentos e implantes, brasileiras ou estrangeiras: - Novartis: Sacubitril.
Rosalia Morais Torres	Nada a ser declarado
Rui Manuel dos Santos Povoa	Outros relacionamentos Financiamento de atividades de educação médica continuada, incluindo viagens, hospedagens e inscrições para congressos e cursos, provenientes da indústria farmacêutica, de órteses, próteses, equipamentos e implantes, brasileiras ou estrangeiras: - Servier: Perindopril.
Sergio Gabriel Rassi	Nada a ser declarado
Silvia Marinho Martins Alves	Outros relacionamentos Financiamento de atividades de educação médica continuada, incluindo viagens, hospedagens e inscrições para congressos e cursos, provenientes da indústria farmacêutica, de órteses, próteses, equipamentos e implantes, brasileiras ou estrangeiras: - Novartis: Entresto.
Suelene Brito do Nascimento Tavares	Nada a ser declarado
Swamy Lima Palmeira	Nada a ser declarado
Telêmaco Luiz da Silva Júnior	Nada a ser declarado
Thiago da Rocha Rodrigues	Nada a ser declarado
Vagner Madrini Junior	Nada a ser declarado
Veruska Maia da Costa Brant	Nada a ser declarado
Walderez Ornelas Dutra	Nada a ser declarado
Wilson Alves de Oliveira Junior	Declaração financeira B - Financiamento de pesquisas sob sua responsabilidade direta/pessoal (direcionado ao departamento ou instituição) provenientes da indústria farmacêutica, de órteses, próteses, equipamentos e implantes, brasileiras ou estrangeiras: - Entresto/Novartis: insuficiência cardíaca em Doença de Chagas Crônica.

## Lista de Siglas/Abreviaturas

^18^ F-FDG – fluordesoxiglicose marcado com flúor-18 

^123^ I-MIBG – meta-iodo-benzil-guanidina marcado com iodo-123 

 ACLS – Suporte Avançado de Vida Cardiovascular ( *advanced cardiovascular life support* ) 

 AIDS – síndrome da imunodeficiência adquirida ( *acquired immunodeficiency syndrome* ) 

AIT – ataque isquêmico transitório

ANVISA – Agência Nacional de Vigilância Sanitária

APS – atenção primária à saúde

 ATP – adenosina trifosfato (adenosine triphosphate) 

AVC – acidente vascular cerebral

BAV – bloqueio atrioventricular

BAVT – bloqueio atrioventricular total

 BDASE – bloqueio divisional anterossuperior esquerdo 

 BNP – peptídeo natriurético tipo B ( *brain natriuretic peptide* ) 

BRA – bloqueador de receptor da angiotensina II

BRD – bloqueio de ramo direito

BRE – bloqueio de ramo esquerdo

CCDC – cardiomiopatia crônica da doença de Chagas

CDC – cardiomiopatia da doença de Chagas

CDI – cardioversor-desfibrilador implantável

 CLIA – quimioluminescência ( *chemiluminescence immunoassay* ) 

CMD – cardiomiopatia dilatada

CMI – cardiomiopatia isquêmica

 CMIA – quimioluminescência magnética ( *chemiluminescent microparticle immunoassay* ) 

 CONITEC – Comissão Nacional de Incorporação de Tecnologias 

COVID-19 – doença causada pelo novo coronavírus

 DACM – dispositivo de assistência circulatória mecânica 

DC – doença de Chagas

DDVE – diâmetro diastólico do ventrículo esquerdo

DTN – doenças tropicais negligenciadas

 DTU – unidades discretas de tipagem ( *discrete typing units* ) 

ECG – eletrocardiograma

 ECLIA – eletroquimioluminescência ( *electrochemiluminescence immunoassay* ) 

ECO – ecocardiograma

ECR – ensaio clínico randomizado

EEF – estudo eletrofisiológico

 ELISA – ensaio imunoenzimático ( *enzyme-linked immunoassay* ) 

EUA – Estados Unidos da América

EV – extrassístole ventricular

FA – fibrilação atrial

FC – frequência cardíaca

 FDA – Agência Norte-Americana de Alimentos e Medicamentos ( *Food and Drug Administration* ) 

FEVE – fração de ejeção de ventrículo esquerdo

FIDC – forma indeterminada da doença de Chagas

 FINDECHAGAS – Federação Internacional de Associações de Pessoas Afetadas pela Doença de Chagas 

FIOCRUZ – Fundação Oswaldo Cruz

FV – fibrilação ventricular

 G-CFS – fator estimulador de colônias de granulócitos ( *granulocyte colony-stimulating factor* ) 

 GLS – deformação longitudinal global ( *global longitudinal strain* ) 

 GRADE – *Grading of Recommendations Assessment, Development and Evaluation*

 GWAS – *Genome Wide Association Study*

HAI – hemaglutinação indireta

HAS – hipertensão arterial sistêmica

 HIV – vírus da imunodeficiência humana ( *human immunodeficiency virus* ) 

IC – insuficiência cardíaca

 ICFElr – insuficiência cardíaca com fração de ejeção levemente reduzida 

 ICFEr – insuficiência cardíaca com fração de ejeção reduzida 

ICT – índice cardiotorácico

IDI – discriminação integrada

 IECA – inibidor da enzima de conversão da angiotensina 

IFI – imunofluorescência indireta

IFN-γ – interferon-gama

IL – interleucina

INSS – Instituto Nacional do Seguro Social

 IPEC-FIOCRUZ – Instituto de Pesquisa Evandro Chagas-Fundação Oswaldo Cruz 

 ISHLT – *International Society for Heart & Lung Transplantation*

LACEN – Laboratório Central de Saúde Pública

 LAVA – *local abnormal ventricular activities*

MEE – medicina embasada em evidências

 MESH – descritores de assuntos médicos ( *medical subject headings* ) 

miRNA – microRNA

MMF – micofenolato de mofetil

MP – marca-passo

 mTOR – *mechanistic target of rapamycin*

 NK – exterminadores naturais ( *natural killers* ) 

NNT – número necessário a tratar

 NT-proBNP – porção N-terminal do pró-peptídeo natriurético tipo B ( *N-terminal pro-brain natriuretic peptide* ) 

 NRI – índice de reclassificação líquida ( *net reclassification index* ) 

 NYHA – *New York Heart Association*

ODS – objetivos de desenvolvimento sustentável

OMS – Organização Mundial da Saúde

OPAS – Organização Panamericana da Saúde

PCDT – Protocolo Clínico e Diretrizes Terapêuticas

PCP – pressão capilar pulmonar

 PCR – reação em cadeia da polimerase ( *polymerase chain reaction* ) 

 PET/TC – tomografia computadorizada por emissão de pósitrons 

 PRA – painel imunológico ( *panel reactive antibody)*

PVC – pressão venosa central

qPCR – PCR quantitativa ou em tempo real

RDC – reativação da doença de Chagas

RM – ressonância magnética

RMC – ressonância magnética cardíaca

RNI – relação normatizada internacional

 SARS-CoV-2 – coronavírus da síndrome respiratória aguda grave 2 

SBC – Sociedade Brasileira de Cardiologia

SGLT2 – cotransportador de sódio e glicose tipo 2

 SINAN – Sistema de Informação de Agravos de Notificação 

 SNP – polimorfismos de nucleotídeos simples ( *single nucleotide polymorphisms* ) 

 SPECT-CT – tomografia computadorizada de emissão de fóton único/tomografia computadorizada 

 SSFP – *steady-state free precession*

 STE – ecocardiografia com rastreamento de pontos ( *speckle tracking echocardiography* ) 

SUS – Sistema Único de Saúde

*T. cruzi* – *Trypanosoma cruzi*

 TAPSE – deslocamento sistólico do plano do anel tricúspide 

TC – transplante cardíaco

TNF-α – fator de necrose tumoral alfa

TRC – terapia de ressincronização cardíaca

Tregs – células T reguladoras

TV – taquicardia ventricular

TVNS – taquicardia ventricular não sustentada

TVS – taquicardia ventricular sustentada

UBS – Unidade Básica de Saúde

UPAE – Unidade de Pronto-Atendimento Especializado

VD – ventrículo direito

VE – ventrículo esquerdo

VFC – variabilidade da frequência cardíaca

## Sumário

1. Considerações Iniciais 12

 1.1. Metodologia Utilizada na Elaboração desta Diretriz 14 

 1.2. Racional Científico para Recomendações de Métodos Diagnósticos 16 

2. Epidemiologia – Atualização no SÉCULO XXI 17

2.1. Introdução 17

2.2. Distribuição Mundial da Doença de Chagas 19

2.3. Situação da Doença de Chagas no Brasil 20

2.4. Vigilância Epidemiológica no Brasil 21

 2.5. Associação de Doença de Chagas com COVID-19 23 

 2.6. Reflexão Final sobre o Cenário Epidemiológico Atual Relativo à Doença de Chagas 24 

 3. Patogênese da Cardiomiopatia da Doença de Chagas 24 

3.1. Introdução 24

 3.2. Dinâmica Imune e Progressão Diferencial para Cardiomiopatia Crônica da Doença de Chagas 25 

 3.3. Disfunção Mitocondrial Miocárdica e Cardiomiopatia Crônica da Doença de Chagas 26 

 3.4. Genética na Cardiomiopatia Crônica da Doença de Chagas 27 

3.5. Distúrbio Microvascular Coronário 27

3.6. Denervação cardíaca 29

3.7. Considerações finais 29

 4. Fisiopatologia da Cardiomiopatia - Fases Aguda e Crônica 29 

4.1. Introdução 29

4.2. Parasitismo Miocárdico e Resposta Imune 30

**4.2.1. Resposta Imune na Fase Aguda** 30 

**4.2.2. Resposta Imune na Fase Crônica** 30 

 4.3. Alterações do Sistema Nervoso Autonômico na Doença de Chagas: Evidências de Estudos Histopatológicos 31 

 4.4. Fisiopatologia da Doença de Chagas Dependente de Características Genéticas Parasitárias e do Hospedeiro Humano 32 

 4.5. Histopatologia Peculiar da Doença de Chagas 33 

4.6. Lesões da Microcirculação Coronária 33

 4.7. Aplicações Terapêuticas Potenciais de Alvos Fisiopatológicos na Cardiomiopatia Crônica da Doença de Chagas 34 

5. História Natural 34

5.1. A Miocardite Aguda da Doença de Chagas 34

 5.2. A Forma Indeterminada e as Síndromes Clínicas da Cardiomiopatia Crônica da Doença de Chagas 35 

**5.2.1. História Natural da Fase Crônica da Doença de Chagas** 35 

**5.2.2. Forma Indeterminada da Doença de Chagas: Importância do Conceito e Alterações aos Exames Complementares Mais Sofisticados** 36 

**5.2.3. Evolução para Cardiomiopatia Crônica** 36 

**5.2.4. Formas Clínicas da Cardiomiopatia Crônica da Doença de Chagas** 37 

**5.2.4.1. Alterações em Exames Subsidiários** 37 

**5.2.4.2. Arritmias Cardíacas** 38 

**5.2.4.3. Síndrome de Insuficiência Cardíaca** 39 

**5.2.4.4. Síndrome Tromboembólica Sistêmica e Pulmonar** 39 

 6. Diagnóstico da Cardiomiopatia da Doença de Chagas 40 

 6.1. Métodos para Evidenciar a Infecção pelo Agente Etiológico ( *T. cruzi* ) 40 

**6.1.1. Introdução** 40 

**6.1.2. Exames Sorológicos Disponíveis e Testes a Solicitar** 40 

**6.1.3. Interpretação dos Resultados** 40 

**6.1.4. Situações Especiais** 41 

**6.1.4.1. Resultados Sorológicos Inconclusivos** 41 

**6.1.4.2. Resultado Laboratorial Não Corresponde ao Esperado Clinicamente** 41 

**6.1.4.3. Parasitemia** 41 

**6.1.4.4. Sorologia Negativa em Pacientes na Fase Crônica** 41 

**6.1.4.5. Cura Espontânea** 41 

**6.1.4.6. Diagnóstico de Fase Aguda** 42 

**6.1.4.7. Serviços de Hemoterapia** 43 

**6.1.4.8. Transmissão Congênita** xx 

**6.1.4.9. Sorologia no Indivíduo Infectado, mas Tratado com Quimioterápicos** 43 

**6.1.4.10. Testes Sorológicos Rápidos** 43 

**6.1.4.11. Testes Parasitológicos** 43 

**6.1.4.11.1. Indicações de Testes Parasitológicos, em Particular, Reação em Cadeia da Polimerase** 44 

**6.1.4.11.2. Interpretação de Resultados de Testes Parasitológicos** 44 

**6.1.4.12. Reação em Cadeia da Polimerase** 44 

**6.1.4.13. Procedimentos Operacionais para Uso da PCR** 45 

 6.2. Métodos Diagnósticos de Alterações Cardíacas Estruturais e Funcionais 45 

**6.2.1. Eletrocardiograma na Doença de Chagas** 45 

**6.2.2. Radiografia de Tórax** 48 

**6.2.3. Ecocardiografia** 48 

**6.2.3.1. Função Sistólica do Ventrículo Esquerdo** 48 

**6.2.3.2. Alterações Segmentares da Contratilidade Ventricular** 48 

**6.2.3.3. Função Diastólica do Ventrículo Esquerdo** 48 

**6.2.3.4. Avaliação do Ventrículo Direito** 49 

**6.2.3.5. Ecocardiograma sob Estresse** 49 

**6.2.4. Ressonância Magnética Cardíaca** 49 

**6.2.5. Medicina Nuclear** 50 

**6.2.5.1. Ventriculografia Radioisotópica** 50 

**6.2.5.2. Perfusão Miocárdica** 50 

**6.2.5.3. Avaliação da Inervação Simpática** 50 

**6.2.6. Tomografia Computadorizada das Artérias Coronárias** 50 

**6.2.7. Eletrocardiografia Dinâmica (Holter)** 51 

**6.2.8. Estudo Eletrofisiológico Intracardíaco** 51 

**6.2.9. Teste Ergométrico e Teste Cardiopulmonar** 51 

**6.2.10. Cateterismo Cardíaco** 51 

7. Estratificação de Risco e Prognóstico 52

 8. Condutas Terapêuticas na Forma Indeterminada da Doença de Chagas 57 

9. Tratamento Etiológico da Doença de Chagas 59

9.1. Introdução 59

9.2. Fármacos e Administração 60

 9.3. Tratamento Etiológico de Indivíduos com Doença de Chagas 62 

9.4. Infecção Aguda 64

9.5. Infecção Congênita 64

 9.6. Crianças e Adolescentes com Infecção Crônica 65 

 9.7. Mulheres em Idade Fértil com Infecção Crônica 65 

9.8. Adultos em Geral com Infecção Crônica 65

9.9. Reativação da Doença de Chagas 67

9.10. Infecção Acidental 68

 9.11. Avaliação de Cura da Doença de Chagas Pós-Tratamento Etiológico 68 

**9.11.1. Onde Realizar Tratamento da Pessoa Acometida** 68 

 10. Condutas Terapêuticas na Disfunção Ventricular e Insuficiência Cardíaca 69 

10.1. Recursos Farmacológicos 69

**10.1.1. Classificação da Insuficiência Cardíaca** 69 

**10.1.2. Dose Máxima de Medicações** 70 

**10.1.3. O Paciente Contemporâneo** 70 

**10.1.4. Revisão da Literatura** 70 

**10.1.5. Terapia Farmacológica** 70 

**10.1.5.1. Diuréticos** 70 

**10.1.5.2. Inibidores do Sistema Renina-Angiotensina-Aldosterona** 71 

**10.1.5.3. Betabloqueadores** 71 

**10.1.5.4. Espironolactona** 72 

**10.1.5.5. Ivabradina** 72 

**10.1.5.6. Digoxina** 72 

**10.1.5.7. Sacubitril-Valsartana** 72 

**10.1.5.8. Inibidores do Cotransportador de Sódio e Glicose do Tipo 2** 73 

10.2. Recursos Não Farmacológicos 75

**10.2.1. Transplante Cardiaco** 75 

**10.2.1.1. Estratégias de Imunossupressão** 75 

**10.2.1.2. Terapia de Indução** 75 

**10.2.1.3. Terapia de Manutenção** 76 

**10.2.2. Diagnóstico e Tratamento da Rejeição** 78 

**10.2.3. Diagnóstico e Tratamento da Reativação da Infecção pelo T. cruzi** 78 

**10.2.3.1. Apresentação Clínica** 78 

**10.2.3.2. Diagnóstico Parasitológico da Reativação** 79 

**10.2.3.3. Tratamento Etiológico da Reativação** 79 

**10.2.3.4. Complicações Pós-Transplante Cardíaco e Sobrevivência** 79 

**10.2.4. Assistência Circulatória Mecânica** 79 

 11. Condutas Terapêuticas nas Arritmias Cardíacas 81 

11.1. Recursos Farmacológicos 81

**11.1.1. Introdução** 81 

**11.1.2. Prevenção da Morte Súbita com Fármacos Não Antiarrítmicos** 81 

**11.1.3. Arritmias Ventriculares em Cardiopatias de Outras Etiologias** 82 

**11.1.4. Amiodarona em Pacientes com Cardiopatias de Outras Etiologias: Prevenção Primária** 82 

**11.1.5. Amiodarona em Pacientes com Cardiopatias de Outras Etiologias: Prevenção Secundária** 84 

**11.1.6. Arritmias Ventriculares em Pacientes com Cardiomiopatia Crônica da Doença de Chagas: Características e Tratamento** 85 

**11.1.6.1. Extrassístoles Ventriculares** 85 

**11.1.6.2. Taquicardia Ventricular Não Sustentada** 85 

**11.1.6.3. Taquicardia Ventricular Sustentada e Fibrilação Ventricular** 86 

**11.1.7. Cuidados Durante Utilização de Amiodarona** 87 

**11.1.8. Prevenção de Choques Elétricos Recorrentes em Pacientes Tratados com Cardioversor-Desfibrilador Implantável** 88 

**11.1.9. Tratamento Medicamentoso da Fibrilação Atrial na Cardiomiopatia Crônica da Doença de Chagas** 88 

**11.1.10. Tratamento na Sala de Emergência** 90 

**11.1.11. Tratamento Ambulatorial** 90 

**11.1.11.1. Reversão para Ritmo Sinusal** 90 

**11.1.11.2. Controle da Frequência Cardíaca** 90 

 11.2. Marca-passo, Cardioversor-Desfibrilador e Ressincronizador 90 

**11.2.1. Marca-passo Cardíaco Artificial** 90 

**11.2.2. Cardioversor-Desfibrilador Implantável na CCDC** 91 

**11.2.2.1. Prevenção Primária de Morte Súbita Cardíaca** 91 

**11.2.2.2. Prevenção Secundária de Morte Súbita Cardíaca** 93 

**11.2.3. Terapia de Ressincronização Cardíaca** 94 

11.3. Métodos de Ablação 96

**11.3.1. Taquicardia Ventricular Sustentada: Apresentação Clínica, Mecanismos Eletrofisiológicos e Localizações** 96 

**11.3.2. Avaliação Clínica e Laboratorial Antes da Ablação** 97 

**11.3.3. Técnicas de Mapeamento das Taquicardias Ventriculares** 97 

**11.3.4. Desfechos e Complicações Durante o Procedimento de Ablação da Taquicardia Ventricular** 98 

**11.3.5. Resultados da Ablação e Seguimento dos Pacientes** 98 

 12. Condutas para Prevenção e Tratamento de Complicações Tromboembólicas 99 

12.1. Introdução 99

12.2. Epidemiologia dos Eventos Tromboembólicos 99

12.3. Fatores de Risco e Mortalidade 99

 12.4. Avaliação de Risco de Acidente Vascular Cerebral 101 

 12.5. Quadro Clínico e Investigação Diagnóstica do Acidente Vascular Cerebral Isquêmico na Doença de Chagas 101 

 12.6. Tratamento do Acidente Vascular Cerebral Isquêmico na Doença de Chagas 103 

 12.7. Prevenção de Eventos Cardioembólicos na Doença de Chagas 104 

 13. Condutas em Subgrupos Especiais e Abordagem de Problemas Relativos a Gravidez, Atividade Física, Risco Cirúrgico, Anestesia Geral e COVID-19 106 

13.1. Coinfecção T. cruzi-HIV 106

 13.2. Soropositividade em Doadores Potenciais nos Bancos de Sangue 107 

13.3. Atividade Física 107

13.4. Gestantes 108

13.5. Recém-natos 109

13.6. Risco Cirúrgico e Anestesiológico 110

 13.7. Doença de Chagas e Infecção por Coronavírus 111 

 13.8. Transplante Não Cardíaco e Terapia Imunossupressora 111 

**13.8.1. Doador com Doença de Chagas e Receptor sem Doença de Chagas** 112 

**13.8.2. Receptor com Doença de Chagas** 112 

**13.8.3. Doenças Autoimunes** 113 

13.9. Doença de Chagas e Senescência 113

 14. Recomendações para Constituição de Serviços Estruturados para Acompanhamento de Pessoas Com Cardiomiopatia Crônica da Doença de Chagas 114 

 14.1. Atribuições dos Serviços Estruturados para Acompanhamento de Pessoas com Cardiomiopatia Crônica da Doença de Chagas 115 

 14.2. Benefícios Esperados dos Serviços Estruturados para Acompanhamento de Pessoas com Cardiomiopatia Crônica da Doença de Chagas 116 

 15. Definição de Cardiopatia Grave e Avaliação Médico-Trabalhista 116 

15.1. Introdução 116

15.2. Conceito e Âmbito 117

 15.3. Escore Capaz de Predizer o Risco de Óbito em Pacientes com Cardiomiopatia Crônica da Doença de Chagas 117 

15.4. Aspectos Clínicos 117

15.5. Função Pericial 118

15.6. Conclusão 118

Agradecimentos 118

Referências 119

## 1. Considerações Iniciais

 Em 2021, por iniciativa de seu então presidente, Dr. Marcelo Queiroga Cartaxo Lopes, a Sociedade Brasileira de Cardiologia (SBC) nos comissionou para a coordenação dos trabalhos, visando à elaboração da nova diretriz relativa à doença de Chagas (DC). Justificava-se a empreitada, uma vez que, desde 2011, a SBC não se responsabilizava diretamente por uma diretriz no contexto. Diversamente daquela, publicada há mais de uma década nos *Arquivos Brasileiros de Cardiologia* , ^1^ a atual não mais seria “latino-americana”, mas passaria a contar essencialmente “apenas” com contingente expressivo de colaboradores nacionais. A plêiade ilustre de investigadores ativos no contexto, que então convocamos, seria representativa de uma equipe ainda mais dilatada de profissionais dos mais diversificados pontos do país, que se envolvem e contribuem diretamente para o avanço no combate à DC, e passou a responder integralmente pela autoria desta diretriz, conforme explicitado abaixo. 

 Além disso, considerando que em 2015 havíamos colaborado extensamente com a edição pela Sociedade Brasileira de Medicina Tropical de outra diretriz sobre o contexto geral da DC, ^2^ resolveu-se limitar o escopo da atual, para focalizar “somente” os aspectos relacionados com o diagnóstico e o tratamento da manifestação mais frequente e grave, a cardiopatia da doença de Chagas (CDC). 

 Apesar de existir enorme gama de documentos que aborda esse tema em seus variados aspectos ( Quadro1.1 ), ^1 - 22^ discrepâncias entre eles no que diz respeito, principalmente, às forças de recomendações e níveis de evidências relacionados aos diversos tipos de tratamentos, assim como o surgimento de novas evidências científicas, corroboram o entendimento de que as diretrizes precisam ser periodicamente revistas e atualizadas. 

**Quadro 1.1:** – Consensos, diretrizes e guias relevantes na abordagem de pacientes com doença de Chagas.

TÍTULO DO DOCUMENTO	TEMA	ANO DE PUBLICAÇÃO	RESPONSÁVEL
“Consenso Brasileiro em Doença de Chagas” ^3^	Geral	2005	Ministério da Saúde do Brasil
“Diagnosis, Management and Treatment of Chronic Chagas' Heart Disease in Areas Where *Trypanosoma cruzi* Infection is not Endemic” ^4^	Geral	2007	SEMTSI
“Evaluation and Treatment of Chagas Disease in the United States: A Systematic Review” ^5^	Geral	2007	CDC e painel de *experts* em Chagas
“I Diretriz Latino Americana para o Diagnóstico e Tratamento da Cardiopatia Chagásica” ^1^	Geral	2011	SBC
“Consenso de Enfermedad de Chagas-Mazza” ^6^	Geral	2011	SAC
“II Consenso Brasileiro em Doença de Chagas” ^2^	Geral	2015	Ministério da Saúde do Brasil
“Chagas Cardiomyopathy: An Update of Current Clinical Knowledge and Management: A Scientific Statement From the American Heart Association” ^7^	Geral	2018	AHA
“Protocolo Clínico e Diretrizes Terapêuticas (PCDT) da Doença de Chagas” ^8^	Geral	2018	CONITEC
“Acuerdo Regional de los Expertos en Chagas de las Sociedades de Cardiología Sudamericanas” ^9^	Geral	2018	SSC
“Guía para el Diagnóstico y el Tratamiento de la Enfermedad de Chagas” ^10^	Geral	2018	OPS
“Consenso Enfermedad de Chagas 2019” ^11^	Geral	2020	SAC
“Consenso do Comitê de Eletrofisiologia da USCAS sobre o Tratamento das Arritmias Ventriculares na Doença de Chagas” ^12^	Arritmia	2002	SBC
“Diretrizes Brasileiras de Dispositivos Cardíacos Eletrônicos Implantáveis (DCEI)” ^13^	Arritmia	2007	SBC
“Consenso de Prevención Primaria y Secundaria de Muerte Súbita” ^14^	Arritmia	2012	SAC/SUC
“Diretrizes Brasileiras de Dispositivos Cardíacos Eletrônicos Implantáveis” ^15^	Arritmia	2015	DECA/SBCCV
“II Diretrizes da Sociedade Brasileira de Cardiologia para o Diagnóstico e Tratamento da Insuficiência Cardíaca” ^16^	IC	1998	SBC
“Revisão das II Diretrizes da Sociedade Brasileira de Cardiologia para o Diagnóstico e Tratamento da Insuficiência Cardíaca” ^17^	IC	2002	SBC
“III Diretriz Brasileira de Insuficiência Cardíaca Crônica” ^18^	IC	2009	SBC
“Atualização da Diretriz Brasileira de Insuficiência Cardíaca” ^19^	IC	2012	SBC
“3ª Diretriz Brasileira de Transplante Cardíaco” ^20^	IC	2018	SBC
“Diretriz Brasileira de Insuficiência Cardíaca Crônica e Aguda” ^21^	IC	2018	SBC
“Consenso de Enfermedad de Chagas. Insuficiencia cardíaca en miocardiopatía chagásica crónica” ^22^	IC	2019	FAC

AHA: American Heart Association; CDC: US Centers for Disease Control and Prevention; CONITEC: Comissão Nacional de Incorporação de Tecnologias no SUS; DECA/SBCCV: Departamento de Estimulação Cardíaca Artificial da Sociedade Brasileira de Cirurgia Cardiovascular; FAC: Federación Argentina de Cardiología; IC: insuficiência cardíaca; OPS: Organização Panamericana da Saúde; SAC: Sociedad Argentina de Cardiologia; SBC: Sociedade Brasileira de Cardiologia; SEMTSI: Sociedad Española de Medicina Tropical y Salud Internacional; SSC: Sociedad Sudamericana de Cardiología; SUC: Sociedad Uruguaya de Cardiología.

 Esta diretriz, à parte seu arcabouço habitual naturalmente voltado para formulação de normas de conduta e evidências científicas que a embasam quanto aos inúmeros aspectos de diagnóstico e tratamento da CDC, reveste-se de algumas características que o contexto temporal durante o qual foi elaborada lhe emprestou. De fato, vivia-se a angustiante circunstância de, em muitos pacientes, à CDC, entidade nosológica marcantemente inflamatória, somar-se o agravo da pandemia da doença causada pelo novo coronavírus (COVID-19), também com seu inerente componente de inflamação. Então, a coletividade científica, tanto em âmbito mundial como, em especial, no Brasil, teve que se arrostar com pelo menos três grandes obstáculos para controlar a pandemia: primeiro, trata-se de vírus especial, com comportamento bastante peculiar quanto ao ataque aos órgãos do hospedeiro individual; segundo, havia dificuldades inerentes e imprevisíveis quanto ao seu comportamento em termos epidemiológicos; terceiro, nossa indigência nacional, quando se constata que para se dominar a pandemia, as medidas adequadas esbarram em fatos básicos, como as muito precárias condições sanitárias de 30-40% de nossa população, carente de esgoto, água encanada e habitação minimamente condizente. 

 Posturas negacionistas e disseminação de falsos conceitos, inclusive por elementos de parte da coletividade médica, representaram óbice incremental ao desempenho da Ciência e da Medicina no combate à pandemia. ^23^ Ao conjunto desses desafios e obstáculos, a comunidade científica nacional respondeu com notável presteza e eficiência, como exemplificado pelo desenvolvimento e aplicação, em larga escala, de vacinas contra a COVID-19. Convém destacar que o difícil cenário que se enfrentava para ampliar a proteção contra o contágio e implementar a vacinação populacional era reminiscente das guerras que travamos durante o século XX contra as perniciosas influências industriais, as quais, durante tanto tempo e tão renitentemente, tentavam ocultar os malefícios do tabagismo. ^24^ De realce, alguns aspectos da concomitância das duas infecções - pelo *Trypanosoma cruzi* ( *T. cruzi)* e pelo coronavírus - no mesmo indivíduo foram adequadamente focados em tópicos específicos desta diretriz. 

 Neste ponto, é inafastável a lembrança de que as conquistas sanitárias no combate à pandemia deste século XXI, angariadas pela comunidade científica, tão bem representada pela FIOCRUZ, como herdeira histórica de seu primeiro e inexcedível epígono, o próprio Oswaldo Cruz, sejam reminiscentes de seu êxito com as campanhas de vacinação contra a febre amarela, no início do século XX. Mas também é oportuno traçar-se um paralelo entre a atual e admirável conjuntura vivificada pela comunidade científica e médica no combate à pandemia de COVID-19 e o difícil contexto vivido por Carlos Chagas e seu mentor Oswaldo Cruz, durante as primeiras décadas do século XX. 

 À semelhança do negacionismo que enfrentamos atualmente, o grande brasileiro, a despeito de sua cientificamente épica descoberta, teve que confrontar o niilismo e a incompreensão com que parte considerável da comunidade médica de então recebia o feito singular de Carlos Chagas na história da Medicina, nas palavras do professor João Carlos Pinto Dias, filho de seu colaborador direto, Emmanuel Dias, e também participante desta diretriz. E, talvez, o desaparecimento precoce de Carlos Chagas, por morte súbita, tenha sido deflagrado por gatilho emocional, consequente à agressão obscurantista. 

 Como assinalamos em outra divulgação, “É também plausível que sua grande perspicácia humanística lhe tenha propiciado a antevisão do tragicamente real significado social da moléstia que revelara ao mundo, por afligir literalmente milhões de indivíduos desvalidos em vastas áreas do território brasileiro. Em acerbo contraste com o negacionismo de parte da comunidade acadêmica em aceitar a própria existência da nova entidade mórbida, possivelmente Carlos Chagas pressentisse o caráter de tragédia nacional que se desvendava a partir de sua descoberta e que se desenrola em múltiplos atos e capítulos deploráveis socialmente até hoje”. ^25^

 Nunca será demasiado glorificar a memória de Carlos Chagas. No dizer inspirado de Alejandro Hasslocher-Moreno, outro colaborador desta diretriz, “Carlos Chagas foi o médico e o cientista certo, na hora certa, no lugar certo. As circunstâncias que envolveram a descoberta da doença tiveram como protagonista um indivíduo amplamente preparado para enfrentar um desafio conhecido e, ao mesmo tempo, descobrir um desconhecido. No contexto biomédico, a ciência brasileira ganhou um grande impulso após a descoberta da DC, passando a ter reconhecimento internacional, um dos principais legados de Carlos Chagas para a ciência e para a medicina brasileira”. ^26^

 Nesse sentido e quando se revisitam e elaboram diretrizes, torna-se plenamente justificável reconhecer a excepcional contribuição dos médicos e cientistas eméritos que nos deixaram justo quando se publicava a de 2011, e agora, quando finalizamos a de 2022. Entre tantos outros, cujos nomes aqui omitimos por razão de espaço, queremos reverenciar a memória ilustre dos Professores Joaquim Romeu Cançado 1913-2011 (Belo Horizonte), Aluízio Rosa Prata 1920-2011 (Uberaba), desaparecidos há já uma década, e de Zilton Araújo Andrade 1924-2020 (Salvador), José Rodrigues Coura 1927-2021 (Rio de Janeiro) e Anis Rassi 1929-2021 (Goiânia), que mais recentemente nos legaram a continuidade de seus trabalhos com a DC. A esses luminares devotamos, por ocasião e lembrança de seu passamento, nossa gratidão e o reconhecimento por permitirem, com sua influência nesta diretriz, nos mantermos na senda luminosamente científica traçada por Carlos Chagas. 

 Os autores, colaboradores e coordenadores em geral deste documento têm plena consciência de que, nesta fase de percepção intensificada quanto a ser a DC ainda negligenciada, impõe-se a premente necessidade de resgatar os indivíduos por ela afligidos de suas miseráveis condições humanas e suas deploráveis implicações médico-sociais. Nesse sentido, deve-se envidar todo esforço para minimizar o estigma que a acompanha, a começar pela abolição do termo “chagásico”, eliminado desta diretriz, a partir da compreensão recente de que em vez de constituir um epônimo fiel à trajetória histórica do grande cientista brasileiro, em alguns pacientes, o termo soa como indicando que em seu coração existe uma verdadeira e dolorosa “chaga” incurável. 

 Perpassa também pelo espírito dos envolvidos na elaboração da diretriz a clara noção de que nossa responsabilidade se incrementou sobremaneira nos últimos tempos. Porquanto, além de dirigir-se precipuamente aos profissionais médicos e paramédicos, os princípios aqui exarados devem ser úteis para nortear a atuação de gestores e órgãos incumbidos de prover condições adequadas de saúde pública em âmbito nacional. E, por último, mas não menos significativo, existe o factual moderno de serem os próprios indivíduos infelicitados pela doença muito mais carentes hoje do que antigamente em termos de orientações seguras por parte dos profissionais que os atendem; de fato, com a democratização inerente ao provimento de recursos informáticos pela web, cresceu paralelamente o contingente dos indivíduos com a doença, que elicitam dos profissionais melhores instruções sobre como gerenciar e minorar o drama acarretado por sua triste condição mórbida. 

 Devemos aproveitar a introdução desta nova diretriz como uma oportunidade para descrever o processo que culminou com este documento. Logo de início, notamos que um cronograma inicialmente planejado para término em alguns meses não correspondia à nossa ambição de construir um documento reflexivo, cientificamente profundo e de implicações clínicas e populacionais consistentes. 

 O ponto de partida ocorreu em reunião onde os coordenadores gerais discutiram os princípios científicos a nortear a confecção dos capítulos, introduzindo a ideia de que o conhecimento dos especialistas seria essencial para interpretação e julgamento da aplicabilidade das evidências, mas não para fomentar opiniões baseadas em preferências pessoais. Discordâncias seriam resolvidas com aprofundamento da análise das evidências, mas não por votação baseada em maioria. Naquele momento, foi plantada a semente para uma diretriz que teve a coragem de desafiar nossas próprias intuições e reconhecer que, muitas vezes, a verdade contraria nossas expectativas, sendo necessário o cultivo da dúvida que, muitas vezes, contrasta com a eloquência de um grupo de formadores de opinião em suas respectivas áreas. Assim foi plantada a semente para uma diretriz construída sob a forma de debates intensos, abrasão criativa e aprendizado de todos, totalizando 7 reuniões virtuais e cerca de 28 horas de debates. Esse processo poderá ser percebido nas entrelinhas do documento final assim engendrado. 

 Desde sua concepção até o último conceito exarado nesta diretriz, intentou-se sempre seguir os mais legítimos e ínclitos princípios do clássico paradigma da Medicina Embasada em Evidências (MEE). Mesmo não tendo sido possível a realização geral exaustiva de revisões sistemáticas da literatura, em alguns contextos mais polêmicos recorreu-se ao método de analisar as evidências que deveriam responder à chamada questão PICO, que engloba as características atinentes à população (”P”), à intervenção (“I”), ao controle comparador (“C”), e ao desfecho ( *outcome* - “O”). ^27^ E esperamos ter sido possível escoimar, pelo menos em grande parte, as recomendações e as análises das evidências que as embasam de vieses e outros desvios de conduta identificáveis em alguns contextos anteriores. Temos a convicção de que, infelizmente, o próprio paradigma da MEE encontra-se atualmente abusado e distorcido, paradoxalmente em meio à exponencial multiplicação de pesquisas e conhecimentos assim gerados e divulgados sem controle proporcional por entidades que deveriam supervisionar todo o processo de avanços nessa área tão nobre da atividade humana. Um exemplo dessas distorções, felizmente não observado no contexto da DC, mas muito nítido em algumas áreas da Medicina, consiste na profusão de meta-análises inadequadas, contraditórias, perfunctórias ou redundantes, resultando em provável forma de “ *fake news* ”, como aventado há algum tempo. ^28^

 Dessa forma, recupera-se e reenfatiza-se o princípio essencial do último trecho da diretriz SBC de 2011, ^1^ consoante o qual, em síntese, “Aos Cardiologistas incumbe, precipuamente, aperfeiçoar o manejo clínico de seus pacientes, administrando-lhes judiciosamente medicamentos e intervenções que respeitem o quanto for possível a fisiopatologia peculiar da doença, não recorrendo a medidas sem comprovação definida de benefício, mas também não desperdiçando oportunidades terapêuticas plausíveis”. 

### 1.1. Metodologia Utilizada na Elaboração desta Diretriz 

 A construção de uma diretriz de condutas médicas traz uma oportunidade pouco apreciada: a da reflexão a respeito da racionalidade que permite a tradução do paradigma científico para a decisão clínica. Esse tipo de reflexão tem o potencial de fazer evoluir estratégias que reduzam a atrição entre evidência e recomendação. 

 Evidências científicas possuem duas funções primordiais: a epistemológica (contrafactual), relativamente à construção do conhecimento de causalidade, e a pragmática, que influencia o processo de decisão (consequencialista). Na primeira função, evidências de caráter “exploratório”, de qualidade satisfatória, têm valor em sugerir os caminhos da ciência. Na segunda função, a de influenciar decisões, a utilização de evidências com alto risco de viés ou imprecisão estatística serve mais para justificar o desejo de agir do que para aumentar a probabilidade de a ação representar a melhor escolha para o paciente ou a população. É o desejo intuitivo buscando justificativa científica. 

 As diretrizes, de modo geral, fazem recomendações clínicas baseadas na qualidade das evidências encontradas, após processo de busca pormenorizada. Vários sistemas têm sido propostos para classificar as evidências e também para categorizar a “força” da recomendação clínica, como o GRADE, CEBM, SIGN, NZGG, SORT, USPSTF, ACCF/AHA/ESC, ACCP, IDSA e NICE. ^29^ A força de recomendação está geralmente ligada ao nível de evidência. Para esta diretriz, resolvemos adotar uma classificação simples, baseada no sistema GRADE ( *Grading of Recommendations, Assessment, Development and Evaluations* ), ^27^ mas com algumas modificações, agrupando os estudos em apenas 3 níveis de evidências (A=alto nível; B=nível moderado; e C=nível baixo), de onde derivam 2 graus de recomendações (1=FORTE; e 2=PONDERADA ou CONDICIONAL). O ponto de partida na avaliação da qualidade da evidência deve ser o tipo de delineamento de pesquisa utilizado. Evidências provenientes de estudos analíticos experimentais, como os ensaios clínicos randomizados (ECR), e de revisões sistemáticas com meta-análises desses estudos estão menos propensas a vieses e, consequentemente, são consideradas de melhor qualidade, ou seja, de alto nível (A). Por outro lado, evidências provenientes de estudos analíticos observacionais (caso-controle, transversal e coorte) são consideradas de nível moderado (B) e aquelas oriundas de estudos observacionais descritivos (sem grupo comparativo), como as séries de casos, de qualidade inferior ou nível baixo (C). 

 No caso específico da CDC, devido à constatação de que não se dispõe, habitualmente, de evidências de qualidade por meio de ECR ou, em algumas situações, nem mesmo por meio de estudos observacionais com resultados substanciais para gerar recomendações avalizadas, existe uma tendência natural de se recorrer à livre “opinião de especialistas” ou “consenso”, palavras abstratas de significado e consequências incertas, que não devem ser formalmente caracterizadas como evidências. ^30^ Uma das soluções para essa questão está no reconhecimento do valor de evidências indiretas, a partir de resultados de ECR realizados em outras cardiopatias, e no entendimento da diferença entre amostra representativa e amostra generalizável. ^27^

 Em estudos observacionais descritivos, é essencial a representatividade da amostra. Por exemplo, se o intuito é descrever o prognóstico de um paciente com insuficiência cardíaca (IC), o que é observado em cardiomiopatia isquêmica (CMI) pode não ser aplicável à CDC. Por outro lado, em estudos analíticos, observacionais ou experimentais (de causalidade), uma amostra não representativa pode vir a ser generalizável. Para que a generalização se justifique, é necessário ausência de interação (modificação de efeito) entre as diferenças das populações e o efeito de um fator de risco ou conduta médica. 

 Como interação biológica é um fenômeno raro, normalmente amostras não representativas geram conceitos generalizáveis para diferentes tipos de pacientes. Isso justifica boa parte das recomendações para idosos ou crianças, subgrupos em geral não representados adequadamente no âmbito de ECR. Prescrever, por exemplo, inibidor da enzima de conversão da angiotensina (IECA) para um paciente com CDC e fração de ejeção ventricular esquerda (FEVE) reduzida não é uma conduta baseada em vontade ou uso de evidência de baixa qualidade nesse tipo de população. É uma conduta baseada em evidência de alta qualidade em outras doenças cursando com IC, alinhada à percepção de que “modificação de efeito” pela etiologia da cardiomiopatia (interação) é improvável. Assim se constrói o conhecimento científico. Por exemplo, a teoria que embasa o conhecimento de que a velocidade da luz é constante não derivou da medida desse parâmetro em todos os ambientes e circunstâncias. Apenas algumas medidas, em linha com a noção de baixa probabilidade de interação entre o ambiente e a velocidade da luz, permitem generalizar que essa velocidade seja, de fato, constante. 

 No exercício da generalização, devemos nos questionar se há característica na população de interesse que mudaria o resultado do estudo. Por exemplo, há alguma característica do paciente com CDC com alto potencial de modificar o efeito (interação) benéfico da terapia vasodilatadora, comprovado em CMI ou em cardiomiopatia dilatada (CMD)? Provavelmente não. 

 Na ausência de evidências experimentais diretas, ou seja, aquelas obtidas a partir de resultados de ECR realizados na CDC (nível A), e de evidências indiretas, obtidas por extrapolação de resultados de ECR realizados em outras cardiopatias (nível B), optamos também por valorizar resultados obtidos a partir de estudos observacionais analíticos (nível B) ou de estudos observacionais descritivos (nível C), ambos realizados na CDC, e ainda adotamos o princípio da plausibilidade extrema e o princípio da assimetria como níveis C de evidências. 

 Vale lembrar que decisões que dispensam evidências empíricas são comuns em Medicina. Na ausência essencial de *equipoise* , as decisões não derivam de dados experimentais, mas de dados naturais. Existem situações que não requerem “julgamento” (mensuração mental de probabilidades) e seria antiético realizar um experimento com grupo controle. Um dos exemplos é o uso de diurético em IC com congestão pronunciada, cujo benefício nunca foi especificamente mensurado por ensaio clínico placebo-controlado, devido ao seu caráter quase determinístico. Se o fosse, teríamos um número necessário a tratar (NNT) de 1 para melhora de sintomas e possivelmente um NNT também muito relevante para redução de mortalidade. 

 A incompreensão dessa afirmação pela comunidade médica posiciona o diurético em um nível inferior de benefício devido à falta de comprovação experimental de redução de mortalidade. Sendo assim, na presença de IC com congestão sistêmica e/ou pulmonar, a prescrição (criteriosa) de diurético deve ser considerada embasada em evidência essencial, o que leva à sua forte recomendação. Para situações desse tipo, costuma-se utilizar a metáfora do paraquedas como estratégia para reduzir mortalidade de pessoas em queda livre. ^31^ Essa é outra circunstância onde o nível de evidência C deve ser aplicado: ausência de evidência experimental, mas forte evidência natural. Isso deve ser enfaticamente diferenciado do paradigma da vontade, contido no “consenso”, pois as evidências a respeito do uso de paraquedas não requerem consenso. São indiscutíveis. 

 Outro princípio que será utilizado como nível C de evidência é o da assimetria de efeito, que pode ser aplicado em situações em que, apesar de não existir ainda comprovação de eficácia de determinada intervenção, há grande assimetria entre a magnitude de um potencial benefício e a magnitude de um eventual malefício, em prol do primeiro, como, por exemplo, uso de máscaras no controle da COVID-19 e tratamento etiológico em adultos com a forma indeterminada da DC (FIDC). 

 Uma vez resolvidas as situações de plausibilidade extrema e assimetria (nível C), devemos partir para resolver as indicações baseadas em nível B de evidências. Esse nível não deve ser representado por evidência de qualidade duvidosa. A qualidade da evidência deve ser de baixo risco de viés e alta precisão, estando aqui representadas as evidências indiretas de alto nível e as diretas de qualidade satisfatória. 

 Enquanto a classificação do nível de evidência faz parte da dimensão científica, a força de recomendação envolve e traduz mais a dimensão do pensamento clínico: da probabilidade individual de benefício (tamanho de efeito) *versus* risco (dano/prejuízo), da dúvida quanto à factibilidade (efetividade) ou até mesmo sobre questões de custo-efetividade (impacto no sistema de saúde). 

 Assim, fazendo um paralelo com o sistema de classificação adotado pelo ACC/AHA, denominaremos o grau de recomendação I e, na maioria das vezes, também o grau de recomendação IIa, como “fortes”, devendo ser aplicados àquelas situações em que há pouca ou nenhuma dúvida quanto ao processo de “prescrição”, que se torna quase uma regra, salvo contraindicações específicas. Por exemplo, prescrever tratamento etiológico em casos de reativação da DC (RDC). Por outro lado, será considerado recomendação “ponderada” ou “condicional” o grau IIb (e, eventualmente, também o IIa), cuja decisão depende de uma análise clínica individualizada em sua magnitude de benefício e risco, valores e preferências do paciente (decisão compartilhada) e de aspectos atinentes ao sistema de saúde ( Quadro 1.2 ). 

**Quadro 1.2:** – Graus de recomendação e níveis de evidência.

PÚBLICO-ALVO	GRAUS DE RECOMENDAÇÃO		NÍVEIS DE EVIDÊNCIA	
	(1) FORTE	(2) PONDERADA (CONDICIONAL)	A	Evidência direta de boa/ótima qualidade (ECR sem limitações importantes ou estudos observacioais com resultados inquestionáveis e expressivos realizados na CCDC).
Gestores	A intervenção deve ser adotada como política de saúde coletiva.	A intervenção pode ser adotada como política de saúde em alguns contextos específicos, levando em consideração o balanço entre benefícios e riscos desta e de outras intervenções alternativas e as prioridades em saúde.		
Profissionais de saúde	Médicos estão seguros e convictos em recomendar a intervenção.	Diferentes escolhas podem ser adotadas pelos médicos e o processo de tomada de decisão compartilhada e informada deve também levar em consideração os valores e as preferências dos pacientes.	B	Evidência indireta de boa/ótima qualidade (extrapolação de resultados de ECR sem limitações importantes ou de estudos observacionais com resultados inquestionáveis e expressivos realizados em outras cardiopatias) ou Evidência direta de moderada qualidade (ECR com limitações, subanálises de ECR incluindo pacientes com CCDC, estudos observacionais com resultados satisfatórios realizados na CCDC)
Pacientes	A maioria dos pacientes, quando bem informados, desejaria a intervenção; apenas uma minoria não a desejaria.	A maioria dos pacientes, quando bem informados, desejaria a intervenção, mas muitos não a desejariam.	c	Ausência de evidência empírica (séries de casos, plausibilidade extrema e princípio da assimetria).

CCDC: cardiomiopatia crônica da doença de Chagas; ECR: ensaios clínicos randomizados.

### 1.2. Racional Científico para Recomendações de Métodos Diagnósticos 

 Em paralelo à organização do pensamento científico aplicado à recomendação sobre conduta terapêutica, tema que predomina em qualquer diretriz, devemos ampliar a discussão para recomendação de testes diagnósticos, visto que também temos capítulos que abordam essas dimensões da decisão médica. 

 Para o contexto do diagnóstico, o conceito científico a alicerçar o nível de evidência não é o de eficácia, como ocorre em tratamento. Aqui se trata do conceito de acurácia, a capacidade de discriminar entre doentes (sensibilidade) e saudáveis (especificidade). Portanto, a questão não é a de prova conceitual de causalidade, nem da necessidade de estudos experimentais randomizados para minimização de fatores de confusão. A necessidade é de demonstração de acurácia suficiente para que a nova informação trazida pelo exame solicitado incremente de forma significativa a probabilidade diagnóstica pré-teste, dentro de uma estrutura de pensamento bayesiano. 

 Nesse caso, o melhor nível de evidência para acurácia diagnóstica deriva de estudos transversais, com metodologia adequada de seleção de pacientes, execução e leitura dos exames pré-definidos e realizados de forma a reduzir erros sistemáticos. Deve-se salientar que estudos de acurácia diagnóstica são muito sensíveis a vieses provocados por observações retrospectivas de bancos de dados (viés de seleção, viés de espectro, viés de observação não cega e não padronizada). 

 Portanto, a qualidade da evidência é essencial, evitando-se a recomendação baseada em informações preliminares. Sendo assim, à semelhança do utilizado para tratamento, a atual diretriz classifica como nível de evidência diagnóstica A e B aquelas com precisão satisfatória e baixo risco de viés, sendo que o nível B se refere à evidência indireta com alto potencial de generalização ou à evidência direta de qualidade satisfatória. O nível de evidência C fica reservado para situações que não requerem evidência empírica, situações incontroversas. Por exemplo, acurácia do eletrocardiograma (ECG) para definir o ritmo cardíaco de base. 

 Quanto à força de recomendação, essa tem na acurácia observada uma condição necessária, porém não suficiente. Um exame acurado não é, obrigatoriamente, de forte indicação. Para tanto, três condições são essenciais: primeiro, o diagnóstico deve ter utilidade clínica, ou seja, implicar em condutas que, em sucessão, beneficiem o paciente; segundo, a informação adicional do teste deve ser necessária e suficiente para incrementar uma probabilidade pré-teste diagnóstica antes indefinida; e terceiro, opções menos complexas, menos invasivas, de menor risco, ou menos custosas devem estar ausentes. Por exemplo, embora a ressonância magnética cardíaca (RMC) seja um teste de melhor acurácia para avaliação de função sistólica, ela não é fortemente recomendada, pois, na grande maioria das vezes, a acurácia do ecocardiograma (ECO) já é suficiente e o método encontra-se largamente disponível, ao contrário da RMC. 

 Essa análise da necessidade e do impacto de um determinado teste diagnóstico é o que norteia sua força de recomendação e, em boa parte das vezes, tem sua definição baseada em racionalidade clínica. Por exemplo, no caso de paciente sintomático, a descoberta de um problema definido é de óbvia utilidade, se houver uma solução específica. A utilidade diagnóstica fica mais duvidosa, no entanto, no caso de rastreamentos, em que há forte *equipoise* entre as consequências intencionais do diagnóstico precoce e a probabilidade de dano. Nessas circunstâncias mais duvidosas, propõe-se a realização de exames diagnósticos por meio de ECR para concretização do esforço diagnóstico. 

 Finalmente, destacamos que o racional descrito para diagnóstico também se aplica, de forma análoga, à definição do nível de evidência e força de recomendação para marcadores e modelos prognósticos. 

## 2. Epidemiologia – Atualização no Século XXI

### 2.1. Introdução

 A DC (tripanossomíase americana) é entidade mórbida transmissível, potencialmente fatal, causada pelo protozoário parasita *T. cruzi* e que integra o grupo de doenças tropicais negligenciadas (DTN) da Organização Mundial da Saúde (OMS). ^32 - 36^ Descoberta por Carlos Ribeiro Justiniano Chagas em 1909, ^37^ segue no século XXI acometendo principalmente pessoas com maior vulnerabilidade social, podendo gerar graves impactos físicos (em especial morte e incapacitação permanente), psicológicos (como medo e estigma) e socioeconômicos também muito ominosos, com reflexos diretos e indiretos na qualidade de vida. ^1 , 2 , 36 , 38 - 43^

 Fatores político-institucionais, econômicos, ambientais (degradação ambiental, alterações climáticas - particularmente o aumento da temperatura) e sociais (migrações humanas nacionais e internacionais e precariedade de condições socioeconômicas, habitação, educação, saneamento, renda, dentre outras) inserem-se igualmente como elementos centrais na determinação do impacto global da transmissão de *T. cruzi* à espécie humana. ^2 , 34 , 36 , 42 , 44^

 Para análise mais aprofundada sobre a DC, torna-se fundamental a identificação de cenários epidemiológicos e sua dinâmica de transmissão, envolvendo desde pessoas infectadas ou sob risco de infecção até diferentes “cepas” de *T. cruzi* , espécies do vetor e reservatórios do agente etiológico, ^2^ em uma perspectiva de Saúde Única - *One Health* . ^45^

*T. cruzi* é parasito hemoflagelado, transmitido principalmente pelo contato com dejetos de diferentes espécies da ordem *Hemiptera* , família *Reduviidae* , subfamília *Triatominae* , cujo habitat se estende da Argentina e Chile até a metade sul dos Estados Unidos da América (EUA), contaminadas ao sugarem o sangue de pessoas ou animais infectados. ^2 , 36 , 38 , 39^

 A transmissão também pode ocorrer pelos meios a seguir mencionados: 1- ingestão de alimentos ou bebidas contaminados com triatomíneos ou seus dejetos; 2- via transplacentária, da mãe infectada para seu feto ou recém-nascido durante a gestação ou o parto; 3- transfusão de sangue ou hemocomponentes de pessoas candidatas à doação, infectadas por *T. cruzi* ; 4- transplantes de órgãos sólidos a partir de doadores infectados; e 5- acidentes com materiais biológicos, particularmente em laboratórios, além de compartilhamento de agulhas/seringas contaminadas por pessoas em uso de drogas ilícitas. ^2 , 32 , 34 , 36 , 38 , 39^ Nessa perspectiva, as ações de prevenção e controle da DC estão diretamente relacionadas às modalidades de transmissão de *T. cruzi* . ^34 , 44^

 A DC é multissistêmica e sua história natural é caracterizada por uma fase aguda, que pode durar até algumas semanas ou meses, geralmente com expressão clínica leve ou assintomática, e uma fase crônica. ^1 , 38 , 44 , 46 - 48^ Caso não seja adequadamente tratada, a infecção por *T. cruzi* pode seguir por toda a vida. ^46^ Estima-se que 30-40% das pessoas infectadas não tratadas desenvolvem síndromes clínicas graves na fase crônica, às vezes fatais, ao longo de suas vidas. Essas lesões estão associadas a acometimento de órgãos-alvo, levando a manifestações cardíacas, digestivas, neurológicas ou mistas, que podem exigir tratamento etiológico. ^2 , 32 , 44^ Esse aspecto reforça a importância do diagnóstico oportuno, em ciclos de vida ainda iniciais, particularmente em pessoas oriundas de comunidades em condição de pobreza e vulnerabilidade social. ^2 , 42 , 44 , 49^

 A carga econômica gerada pela DC nos sistemas nacionais de saúde e para a sociedade é expressiva, igualando-se ou superando a de outras doenças, como infecção por rotavírus ou câncer de colo de útero, mesmo em áreas não endêmicas. ^36 , 50 , 51^ Uma proporção substancial da carga econômica é consequente à perda de produtividade pela morbimortalidade precoce induzida, particularmente, pela cardiomiopatia crônica. ^34 , 50 , 51^ Globalmente, a carga anual é de US$ 627,46 milhões em custos de saúde, com valor líquido global atual de US$ 24,73 bilhões (custos anuais por pessoa de US$ 4.660 e, ao longo da vida, por pessoa, de US$ 27.684). Os custos globais alcançam níveis de US$ 7,19 bilhões por ano e de US$188 bilhões ao longo da vida. Ressalta-se que aproximadamente 10% desses custos associam-se a áreas onde a DC não é endêmica, como EUA e Canadá. ^50^ Assim, superar as barreiras de acesso a diagnóstico e tratamento com a adequada implementação da atenção integral às pessoas com DC reduziria a ocorrência de complicações crônicas e os custos associados aos sistemas nacionais de saúde [por exemplo, para implantes de marca-passo (MP) e cirurgias corretivas], com impacto benéfico para toda a sociedade. ^34 , 44 , 50 , 51^

 Nessa perspectiva, uma avaliação econômica abrangente relativa a medidas destinadas à ampliação do acesso ao diagnóstico e tratamento da doença indicou a importância da triagem sorológica de candidatos (as) à doação de sangue e de gestantes, como estratégias de saúde pública com melhor custo-efetividade. ^52 , 53^ Políticas mais abrangentes, que reconheçam as diferentes dimensões de determinação social, são fundamentais para redução dessa carga, demandando o envolvimento de outras áreas que ultrapassem o setor saúde. ^2 , 33 , 45 , 54^ A agenda trazida pelos Objetivos de Desenvolvimento Sustentável (ODS) integra a DC em seu terceiro objetivo: “assegurar uma vida saudável e promover o bem-estar para todos, em todas as idades”, na meta de “acabar com as epidemias de AIDS, tuberculose, malária e doenças tropicais negligenciadas, e combater a hepatite, doenças transmitidas pela água e outras doenças transmissíveis” até 2030. ^33 , 34 , 55^

 Apesar da alta carga de morbimortalidade da DC e dos elevados custos para os sistemas nacionais de saúde e, sobretudo, para a sociedade, registra-se que 70-90% das pessoas com a doença desconhecem o seu diagnóstico e somente 1% recebe, efetivamente, o tratamento etiológico adequado no século XXI. ^49 , 54 , 56^ Há evidências contundentes de que o diagnóstico e o tratamento etiológico adequado da DC resultam em muitos benefícios, incluindo a prevenção da transmissão congênita futura em mães tratadas, cura sorológica em bebês e crianças e redução da progressão para formas clínicas avançadas da doença nas pessoas aguda e cronicamente infectadas. ^1 , 2 , 7 , 8 , 41 , 44 , 56 - 60^ No entanto, uma vez que a doença tenha progredido para uma fase clínica mais avançada, com comprometimento cardíaco grave, o tratamento etiológico não parece trazer benefícios clínicos. ^1 , 2 , 7 , 8 , 44 , 46 , 60^ Esse fato reforça a necessidade de potencializar o desenvolvimento de métodos diagnósticos mais aprimorados nos cenários locais dos serviços de saúde para garantia de acesso a tratamento precoce, seguro e eficaz. ^8 , 53 , 54 , 56 , 57 , 60^

 Além dos complexos desafios políticos, geográficos, socioeconômicos, culturais, tecnológicos e jurídicos inerentes aos territórios de maior endemicidade para a DC, reconhece-se a persistência de barreiras que limitam o acesso a diagnóstico, tratamento e cuidado longitudinal. ^44 , 54^ Essas barreiras incluem: incompletude e inconsistência de dados sobre a doença; limitação de ações integradas de vigilância, controle e cuidado na rede de Atenção Primária à Saúde (APS); distância geográfica aos serviços de saúde, fluxograma e processo de diagnóstico muitas vezes complicados (sistemas de referência e contrarreferência), demorados e com custos elevados; limitada integração de políticas e ações para saúde reprodutiva, materna, neonatal e infantil; impacto desproporcional da doença em populações mais vulneráveis; conhecimento limitado sobre a doença, tanto na população em geral quanto entre profissionais de saúde; limitado interesse da mídia e da indústria farmacêutica; reduzidas iniciativas de educação em saúde; disponibilidade limitada de ferramentas e materiais nos centros de saúde; medo; estigma e discriminação contra pessoas acometidas; baixa capacidade de mobilização social e protagonismo político limitado das pessoas com maior risco. ^2 , 40 , 41 , 44 , 45 , 49 , 61^

 Ressalta-se que o limitado conhecimento dos profissionais de saúde sobre a DC representa um dos fatores críticos para que os sistemas nacionais de saúde possam garantir amplo acesso a diagnóstico e tratamento adequados. ^2 , 61^ Ademais, há ainda clara necessidade de superar barreiras de acesso relacionadas ao tratamento etiológico, limitado a dois medicamentos eficazes apenas - benznidazol e nifurtimox - que requerem períodos de administração relativamente longos e que podem estar associados a reações adversas que podem complexificar o tratamento, demandando monitoramento clínico e laboratorial. ^1 , 41 , 49 , 54 , 56 , 58 , 60^ Deve-se frisar também que os medicamentos para tratamento etiológico têm limitação de uso em mulheres durante a gestação ou em casos de estágio avançado da doença com comprometimento cardíaco ou cardiodigestivo. ^2^ Entretanto, em gestantes, diante de quadro clínico agudo e grave de DC (por exemplo, miocardite ou meningoencefalite), ^8^ a decisão desse dilema ético relativo ao tratamento etiológico, no contexto da gravidez, impõe-se. ^2^

 Como forma de enfrentamento, no dia 24 de maio de 2019, durante a 72ª sessão da Assembleia Mundial da Saúde, foi instituído o Dia Mundial da Doença de Chagas, em uma das 11 campanhas globais de saúde pública da OMS. ^36^ Além disso, a OMS, em seu documento-guia para DTN, identificou três ações estratégicas para alcançar a eliminação da doença: ação 1 – advogar junto a instituições/órgãos públicos que executam ações de prevenção e controle dos países (ministérios da saúde) para que reconheçam a DC como problema de saúde pública e estabeleçam políticas e ações de prevenção, controle, atenção e vigilância eficazes em todos os territórios endêmicos; ação 2 – qualificar a atenção médica, desde educação permanente em serviço até a integração das ações em toda a rede de atenção; e ação 3 – garantir que os países onde a transmissão vetorial domiciliar/peridomiciliar ainda é registrada possam cumprir com os protocolos de prevenção, controle e vigilância. ^62^

 Ressalta-se nesse enfrentamento a crescente participação social, com engajamento e protagonismo de movimentos sociais em DC globalmente, com mobilização articulada a outros movimentos voltados para DTN, visando a garantia de direitos fundamentais como o de acesso à saúde. ^2 , 34^ Esses movimentos unem-se inclusive em um Fórum Social mais amplo para enfrentamento de DTN no Brasil. ^49^ Além disso, esses movimentos para DC compõem uma federação internacional (FINDECHAGAS – Federação Internacional de Associações de Pessoas Afetadas pela Doença de Chagas [https://findeChagas.org/home-po/]) representativa de países endêmicos e não endêmicos. ^2 , 34^

### 2.2. Distribuição Mundial da Doença de Chagas

 A DC no século XXI mantém padrão epidemiológico de endemicidade em 21 países da região da América Latina, com aproximadamente 70 milhões de pessoas sob risco de exposição à infecção por *T. cruzi* . Há relativa dificuldade no estabelecimento de estimativas mais precisas dentro do contexto de uma DTN, o que traz incertezas. Entretanto, as estimativas atualmente disponíveis têm sido fundamentais para subsidiar agendas para controle da doença. A OMS estima que 6 a 7 milhões de pessoas em todo o mundo estejam infectadas, a maioria na América Latina, traduzindo uma redução de aproximadamente 65% em comparação a 1980 (17 milhões). 

 Cerca de 63% desses casos estão em países da Iniciativa de Países do Cone Sul, com destaque para Argentina (1,5 milhão), Brasil (1,2 milhão), México (880 mil) e Bolívia (610 mil). ^38 , 39 , 63^ O Quadro 2.1 apresenta os diferentes padrões de indicadores epidemiológicos da DC na América Latina em diferentes momentos. 

**Quadro 2.1:** – Mudanças na mortalidade, prevalência e incidência por transmissão vetorial e por transmissão congênita da doença de Chagas em 21 países endêmicos da América Latina, nos anos 1980–1985, 2005 e 2010.

PARÂMETROS - ESTIMATIVAS	1980–1985	2005	2010
Número de mortes/ano	>45.000	12.500	12.000
Número e percentual de pessoas infectadas	17.395.000 (4,3%)	7.694.500 (1,4%)	5.742.167 (1,1%)
Casos novos/ano – total	700.000	55.585	38.593
Casos novos/ano – transmissão vetorial	>500.000	41.200	29.925
Casos novos/ano – transmissão congênita	7.000 - 49.000	14.385	8.668
Número e percentual da população total sob risco	92.895.000 (25,0%)	108.595.000 (20,4%)	70.199.360 (12,9%)

Fonte: Adaptado de Dias et al., 2016; ^2^ WHO, 2002; ^38^ PAHO, 2006; ^39^ e WHO, 2015. ^63^

 Entretanto, esses dados globais divergem das estimativas individualizadas em vários países, o que dificulta o estabelecimento exato da prevalência da doença nas Américas. ^2^ A subnotificação de casos e o não registro de óbitos por DC também representam críticos obstáculos, pois impedem a adoção de medidas de controle mais ajustadas às realidades locais, a partir da vigilância epidemiológica. ^34 , 64 , 65^

 Não obstante essa dificuldade, a expressiva redução da prevalência global está associada ao desenvolvimento de iniciativas regionais, coordenadas com o objetivo de interromper a transmissão de *T. cruzi* . ^2 , 34 , 66 , 67^ A consecução de metas pactuadas de eliminação da transmissão vetorial pela principal espécie ( *Triatoma infestans* ) e por transfusões de sangue foi alcançada por vários países a partir de iniciativas desde a década de 1990, com significativa redução do número de casos novos, persistindo, entretanto, algumas áreas críticas de transmissão na atualidade. ^2 , 66 , 68 , 69^

 Os atuais desafios são ainda muito vultosos. Apenas cerca de 10%-30% das pessoas acometidas por DC sabem do seu diagnóstico, o que contribui para que somente 1% daquelas que necessitam de tratamento etiológico tenha acesso de fato, mantendo o elevado impacto de morbimortalidade e de custo social, com limitação da qualidade de vida. ^36 , 43 , 54 , 56 , 60^ Acresce que, na maioria das áreas onde foi alcançada a interrupção vetorial ou redução da transmissão, ocorre um processo de envelhecimento da população acometida, ampliando a carga de morbimortalidade pela coexistência com doenças crônico-degenerativas, em grande parte cardiovasculares. ^41 , 66 , 70 - 72^ Na população mais idosa, a CDC mantém-se como forte fator preditor de maior risco para morte. ^70^

 Apesar da significativa redução registrada na prevalência, aproximadamente 10 a 15 mil mortes relacionadas à DC ainda são registradas a cada ano. ^2 , 36 , 59^ Em relação à mortalidade específica, também tem sido verificada significativa redução, considerando-se o registro de mais de 45 mil mortes anuais nos anos 1980. No entanto, a mortalidade mantém-se em patamares elevados, ^63 , 65 , 70 , 73^ o que contribui para sustentar a DC como problema de saúde pública. ^44 , 70 , 74 , 75^

 Para além das áreas classicamente endêmicas da América Latina, a DC tem sido progressivamente registrada em países não endêmicos (alguns países da Europa, EUA, Austrália e Japão), em virtude de movimentos migratórios associados a crises político-institucionais, sanitárias, ambientais e econômicas nos países de origem. ^2 , 42 , 67 , 76 - 81^

 Essas estimativas globais são corroboradas por dados recentes oriundos, por exemplo, de um país como a Espanha, onde a doença não é endêmica, mas há pesquisa ativa e foco em medidas de saúde pública para controle. Nesse país, estimou-se que, para 2018, mais de 55 mil dos quase 2,6 milhões de migrantes originários de países endêmicos (54% da Bolívia) vivam com a DC, uma prevalência estimada de 2,1%. Aproximadamente 70% das pessoas migrantes não tinham o diagnóstico estabelecido e a maioria não foi tratada, 83% maiores de 15 anos de idade e 60% crianças. ^82^

 Essas populações também apresentam condições muito precárias de vida, com alta vulnerabilidade social por restrições sociais e econômicas que complicam o acesso à atenção à saúde, inclusive pela baixa experiência profissional no setor específico de saúde. ^42 , 61 , 67 , 77 , 80 , 81^ Destaque-se ainda que o *T. cruzi* também pode atuar como microrganismo oportunista em pessoas com outras patologias associadas a imunossupressão, desencadeando síndromes clínicas potencialmente fatais pela RDC. ^1 , 83 , 84^

 Nesses novos contextos não endêmicos, a possibilidade de transmissão transfusional de *T. cruzi* tem sido cada vez mais reconhecida. Embora a DC raramente seja definida como um problema de saúde pública em países não endêmicos, muitos hemocentros têm implementado nos últimos 10 anos medidas para mitigar o risco relativo à segurança sanguínea com base no reconhecimento de fatores de risco epidemiológico associados a imigrantes latino-americanos e na adoção de testes sorológicos de triagem. ^42 , 79^

 Em contextos endêmicos, o controle de outros modos de transmissão (particularmente vetorial e transfusional) coloca em perspectiva de realce a congênita, responsável por quase um terço das novas infecções em 2010. ^41 , 63 , 69 , 85 - 87^ Estima-se, em regiões endêmicas, que 1,12 milhão de mulheres em idade reprodutiva estejam infectadas ^59^ e que a taxa média de transmissão congênita estimada seja de cerca de 5%, principalmente em áreas endêmicas de alto risco. ^59 , 88^ Como o acesso ao diagnóstico da infecção por *T. cruzi* em mães ou crianças recém-nascidas é limitado na maioria das áreas endêmicas, a prevalência em mulheres grávidas e recém-nascidos pode estar subestimada. ^59 , 88^ Mesmo com essas limitações, a incidência estimada é de 8.000 a 15.000 casos de transmissão congênita por ano na América Latina. ^85^ Por outro lado, essa modalidade de transmissão tem representado um papel central como principal modo de manutenção de *T. cruzi* em áreas não endêmicas. ^59 , 63 , 86 , 87^ Assim, a ocorrência de infecção congênita pode sustentar a transmissão de *T. cruzi* indefinidamente, mesmo em países sem a modalidade vetorial clássica. ^86 , 88^

 Para que seja alcançada a prevenção da transmissão congênita em áreas endêmicas, é fundamental garantir acesso a diagnóstico e tratamento etiológico de meninas e mulheres em idade fértil antes da gravidez. ^57 , 59 , 89^ Além disso, o diagnóstico da infecção por *T. cruzi* em grávidas durante o pré-natal, oportunizando o rastreamento precoce de infecção no recém-nascido, e o diagnóstico da infecção em crianças nascidas de mães infectadas, possibilitando a implementação de tratamento etiológico, seriam medidas altamente eficazes e seguras. ^41 , 57 , 59 , 85 , 86 , 88 , 89^

 A transmissão oral, por sua vez, tem sido registrada particularmente na região amazônica e nos Andes subtropicais, ^90^ tendo papel relevante na geração de casos agudos na Amazônia brasileira e na Venezuela. ^2 , 48 , 90^ Nesses cenários, verifica-se maior mortalidade durante a fase aguda, quando se compara ao que ocorre em casos agudos causados por transmissão vetorial clássica. ^2 , 47 , 48^ A DC aguda transmitida por via oral tem letalidade considerável ao longo do primeiro ano após a infecção, ^48^ como discutido em outro capítulo desta diretriz. 

### 2.3. Situação da Doença de Chagas no Brasil

 É inequívoca a importância de se sustentar no século XXI a vigilância e o controle da DC em todas as suas fases clínicas evolutivas, considerando-se como critérios a magnitude, o potencial de disseminação, a transcendência, a vulnerabilidade e os compromissos internacionais do Brasil. ^2 , 34 , 69 , 91^ Como país de dimensões continentais, vem passando ao longo deste século por transformações demográficas, sociais, econômicas e ambientais, sem que se consigam superar as críticas desigualdades socioeconômicas e regionais. ^2 , 33 , 52^

 Por outro lado, o país possui o Sistema Único de Saúde (SUS), de caráter público, universal e de base democrática, que deve avançar em constante aprimoramento de sua qualidade, com a finalidade de estabelecer a garantia ao direito à saúde para todas as pessoas, o qual foi consagrado na Constituição Federal de 1988. ^2 , 33 , 49 , 92^

 Nesse contexto, a DC mantém-se como a DTN com maior carga de morbimortalidade, particularmente entre homens idosos e residentes no passado em importantes áreas endêmicas para transmissão vetorial. ^73 , 74 , 75^ Tendo em vista a extensão e diversidade do território do país, com implicações nas dinâmicas ecológica, demográfica, social e econômica das regiões, verificam-se múltiplos cenários clínicos, epidemiológicos e operacionais para o controle da doença. ^2 , 34^

 O controle vetorial em áreas endêmicas teve impacto considerável também em relação às transmissões transfusional e congênita, ^64 , 69 , 87^ mas preocupa o cenário atual de fragilização das operações da vigilância entomológica nos municípios endêmicos do país. A “Certificação da Interrupção da Transmissão da Doença de Chagas pelo principal vetor domiciliado, *T. infestans* ”, foi concedida em 2006 pela Organização Panamericana da Saúde (OPAS), dentro da Iniciativa dos Países do Cone Sul. ^2 , 34^ A despeito dos avanços, o risco de transmissão vetorial da DC persiste e tem sido avaliado sob diferentes perspectivas, em decorrência de diversos fatores, entre os quais a existência de espécies de triatomíneos autóctones com elevado potencial de colonização, a presença de reservatórios silvestres e domésticos de *T. cruzi* , a aproximação cada vez mais frequente das populações humanas a esses ambientes, além de persistência de focos residuais de *T. infestans* , mesmo em áreas específicas do estado da Bahia, e a limitação das ações de vigilância entomológica. ^2 , 34^

 No Brasil, em 1980-1985, a estimativa era de 6.180.000 (4,2%) pessoas infectadas por *T. cruzi* , passando para 1.900.000 (1,0%) em 2000. ^38 , 39^ As estimativas mais recentes da OMS indicam infecção em 2010 de 1.156.821 pessoas por *T. cruzi* (0,6%). ^63^ Entretanto, a limitação de estudos de base populacional dificulta avaliações mais realistas da magnitude da DC no país. ^64^ Assim, alguns estudos com base em revisões sistemáticas e meta-análises de dados disponíveis no Brasil estimaram o número de pessoas infectadas variando de 1,9 a 4,6 milhões, provavelmente cifras mais próximas atualmente à variação de 1,0% a 2,4% da população. ^2 , 64^ Com base nessas proporções, estimou-se para 2020 entre 1.365.585 e 3.213.142 o número de brasileiros infectados por *T. cruzi* , sendo 136.559 a 321.314 pessoas com a forma crônica digestiva e 409.676 a 963.943 com a forma crônica cardíaca. Por outro lado, a população estimada com infecção por *T. cruzi* na FIDC variou de 819.350 a 1.927.885 pessoas. ^2^ O Quadro 2.2 apresenta as projeções do número de infectados por *T. cruzi* e o número de casos com DC na fase crônica com formas cardíaca e digestiva no Brasil de 2020 a 2055. 

**Quadro 2.2:** – Projeções do número de pessoas infectadas por *T. cruzi* e do número de casos com doença de Chagas na fase crônica com a forma cardíaca e com a forma digestiva no Brasil, 2020–2055.*

ANO	ESTIMATIVA DA POPULAÇÃO BRASILEIRA	FAIXA ETÁRIA DE REFERÊNCIA			ESTIMATIVA DO NÚMERO DE PESSOAS INFECTADAS		ESTIMATIVA DE CASOS COM A FORMA DIGESTIVA		ESTIMATIVA DE CASOS COM A FORMA CARDÍACA	
		FAIXA ETÁRIA	POPULAÇÃO	%	INFECÇÃO 1,02%	INFECÇÃO 2,4%	INFECÇÃO 1,02%	INFECÇÃO 2,4%	INFECÇÃO 1,02%	INFECÇÃO 2,4%
2020	212.077.375	≥ 25	133.880.929	63,1	1.365.585	3.213.142	136.559	321.314	409.676	963.943
2025	218.330.014	≥ 30	127.334.466	58,3	1.298.812	3.056.027	129.881	305.603	389.644	916.808
2030	223.126.917	≥ 35	120.096.221	53,8	1.224.981	2.882.309	122.498	288.231	367.494	864.693
2035	226.438.916	≥ 40	112.013.898	49,5	1.142.542	2.688.334	114.254	268.833	342.763	806.500
2040	228.153.204	≥ 45	102.983.115	45,1	1.050.428	2.471.595	105.043	247.160	315.128	741.479
2045	228.116.279	≥ 50	92.984.144	40,8	948.438	2.231.619	94.844	223.162	284.531	669.486
2050	226.347.688	≥ 55	82.097.220	36,3	837.392	1.970.333	83.739	197.033	251.218	591.100
2055	222.975.532	≥ 60	70.485.475	31,6	718.952	1.691.651	71.895	169.165	215.686	507.495

*Para taxas de infecção de 1,02% e 2,4% e, considerando-se, 30% dos pacientes com a forma cardíaca e 10% com a forma digestiva da doença de Chagas. Fonte: Adaptado de Dias et al., 2016. ^2^

 Estimou-se para o país, em 2010, prevalência de infecção por *T. cruzi* em gestantes de 1,1% (34.629 mulheres), com média de 589 crianças nascendo com infecção congênita (taxa de transmissão de 1,7%), ^87^ semelhante às estimativas da OMS (571 casos). ^63^ A taxa de transmissão congênita é menor (1,5-2,0%) quando comparada à média de 5% verificada em outros países do Cone Sul, como Argentina, Paraguai e Bolívia. Esses achados sugerem que a presença de TcII se associa a menor transmissão quando comparada a TcV, que predomina na região Sul do Brasil e naqueles países. ^2 , 57^

 Com base nos dados do Sistema de Informação de Agravos de Notificação (SINAN), a ocorrência de casos de DC aguda tem sido alvo da vigilância epidemiológica, segundo a definição de “caso” do Ministério da Saúde do Brasil. Entre 2007 e 2019, foram confirmados 3.060 casos de DC aguda (média de 222 casos/ano) em 219 municípios. ^34^ Já em 2020, foram confirmados 146 casos, principalmente na região Norte, com letalidade de 2% (3/146 - todos os óbitos no estado do Pará). A forma de transmissão mais frequentemente notificada no país nos últimos 15 anos em casos de DC aguda tem sido a via oral, ^34 , 93^ fato revelador de limitações operacionais do processo de vigilância no país, que têm induzido mudanças do perfil epidemiológico da doença na última década. ^2 , 90^

 A carga da mortalidade relacionada à DC no Brasil persiste em níveis significativamente elevados, a despeito das ações de controle empreendidas. A mortalidade é reconhecidamente mais expressiva para idades de 50 a 64 anos e coortes mais idosas, provavelmente relacionada aos efeitos do período de intensificação de ações de controle vetorial, além de mudanças demográficas. ^35 , 75^ As diferenças que têm sido observadas entre as regiões, em especial com maior carga no Centro-Oeste e Sudeste, indicam iniquidades socioeconômicas e o padrão diferencial de acesso aos serviços de saúde no SUS. ^35 , 73^ Registra-se que a região Sul também apresenta redução da tendência de mortalidade, mas com aumento na região Norte, enquanto a região Nordeste não tem tendência definida. ^35 , 74^

 Destaca-se de novo que é justamente a região Norte que concentra a grande maioria dos casos novos notificados no país. ^34 , 47 , 93^ Além da provável subnotificação de casos não associados à transmissão vetorial domiciliar, essa região obteve pouco impacto resultante das ações sistemáticas de controle triatomínico. Esse fato justifica-se uma vez que o ciclo local de transmissão de *T. cruzi* não envolve vetores com capacidade de domiciliação, mas se sustenta em um ciclo enzoótico, com vetores silvestres, implicados nos casos associados à transmissão oral ou vetorial extradomiciliar. ^2 , 47 , 48 , 60 , 73^ É razoável estimar, portanto, que o acúmulo de centenas ou mesmo milhares de casos de infecção por *T. cruzi* ao longo do tempo, na região amazônica, possa estar contribuindo para esse padrão epidemiológico específico. ^47^

 A DC segue tendo forte impacto na Previdência Social e nos Serviços do Instituto Nacional do Seguro Social (INSS) nos estados brasileiros com maior prevalência, ^34^ particularmente com o envelhecimento da população acometida. ^2 , 72^ A análise global para o período de 2030 a 2034 indica declínio progressivo na mortalidade (mais de 75% em comparação a 2010-2014), principalmente entre os mais jovens, variando de 86%, na faixa etária entre 20 e 24 anos, a 50% naqueles com 80 anos ou mais. ^75^ Registra-se ainda o significativo impacto com a redução da qualidade de vida das pessoas com a doença e de suas famílias. ^43^

 A integração das ações de atenção, vigilância e controle da DC na APS tem sido disposta como fundamental e estratégica para a redução da carga de morbimortalidade, sobretudo em territórios endêmicos, para se ampliar acesso a diagnóstico e tratamento etiológico. ^2 , 34 , 44 , 58 , 75^ O documento da OPAS “Cuidados crônicos para doenças infecciosas negligenciadas: hanseníase, filariose linfática, tracoma e doença de Chagas – Um guia para manejo da morbidade e prevenção de incapacidade para serviços de atenção primária à saúde” é um verdadeiro marco, pois assinala vários aspectos fundamentais no cuidado de pessoas acometidas por DC, com vistas a instrumentalizar as equipes de APS e reforçar a importância da integração com as ações de vigilância. ^44^

### 2.4. Vigilância Epidemiológica no Brasil

 A vigilância epidemiológica da DC engloba ações necessariamente integradas, que envolvem a abordagem de casos humanos, vetores e reservatórios, com interface estreita com a rede de atenção à saúde e especial realce para o papel da APS. ^2 , 34 , 91 , 93^

 As ações de vigilância epidemiológica da DC no Brasil têm os seguintes objetivos principais: 1) detectar precocemente casos de DC aguda para tratamento etiológico adequado, bem como para aplicação de medidas de prevenção de ocorrência de novos casos; 2) proceder à investigação epidemiológica de todos os casos agudos, visando identificar a forma de transmissão e adotar medidas adequadas de controle; 3) monitorar a infecção por *T. cruzi* na população humana, por meio de programas de rastreamento na APS, inquéritos sorológicos periódicos em populações estratégicas e análise do processo de triagem de candidatos à doação de sangue em hemocentros; 4) monitorar o perfil de morbimortalidade da DC, delineando ações para fortalecimento da rede de atenção à saúde às pessoas infectadas; 5) manter eliminada a transmissão vetorial por *T. infestans* e sob monitoramento/controle as outras espécies importantes; e 6) integrar ações de vigilância sanitária, ambiental, de vetores e reservatórios às ações de vigilância epidemiológica. ^2 , 93^

 Os dados disponíveis relativos à vigilância epidemiológica de casos humanos não permitem estimar a magnitude nosológica da tripanossomíase americana. Estima-se que somente 10-20% dos casos de DC aguda sejam de fato notificados. ^2 , 47^ Até maio de 2020, quando foi instituída a inclusão da fase crônica da DC também como evento de interesse para fins de vigilância epidemiológica, por meio da notificação compulsória de casos (Portaria nº 1.061, de 18 de maio de 2020), somente a tradicional vigilância de casos na fase aguda era realizada e estava incluída na Lista Nacional de Doenças de Notificação Compulsória e Imediata. ^34 , 93^ Essa ampliação no escopo da vigilância configura ação de grande importância para o país no sentido de se alcançar o reconhecimento nacional de padrões de ocorrência da doença e pode ser seguida por outros países endêmicos. Há uma expectativa de que esse novo processo de vigilância epidemiológica no Brasil esteja implantado em todo o território a partir de 2022. 

 Mais recentemente, para o reconhecimento da magnitude da DC crônica no país, tem sido discutida a importância de se rearticular e integrar as ações de vigilância em saúde, buscando o desenvolvimento de uma ampla rede hierarquizada de serviços de saúde nos vários territórios geográficos, para garantir acesso a milhões de pessoas infectadas por *T. cruzi* . ^44 , 91 , 94^ Com vistas a elaborar um modelo de priorização de municípios para vigilância da DC crônica, uma equipe do Ministério da Saúde realizou análise multicritério preliminar baseada em três índices construídos a partir dos seguintes indicadores: (a) epidemiológicos, diretamente relacionados à DC crônica; (b) decorrentes da evolução da DC crônica; e (c) relacionados ao acesso aos serviços de saúde. O modelo definido como o mais adequado era composto por 1.345 municípios de média prioridade, 1.003 de alta e 601 de muito alta prioridade para DC crônica, principalmente no Sudeste e Nordeste do país. ^94^

**Figura 2.1:**
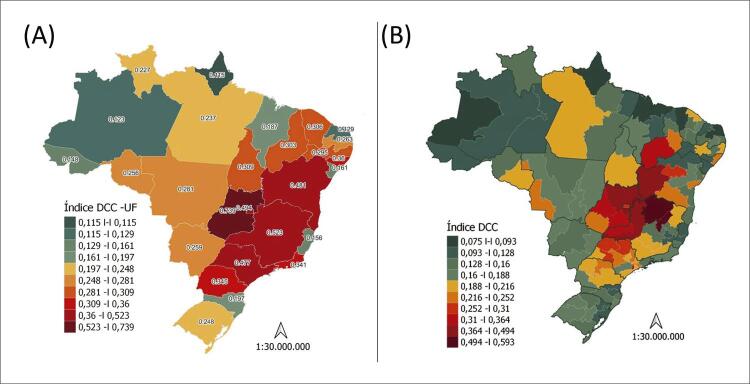
– Distribuição espacial do índice de vulnerabilidade para doença de Chagas crônica (DCC), por unidades federadas e Distrito Federal (A) e macrorregiões de Saúde (B).

 Posteriormente, o Ministério da Saúde propôs a elaboração de um índice de vulnerabilidade para DC crônica, com objetivo de evidenciar áreas com maior risco para morbimortalidade nessa fase da doença, levando em consideração contextos de limitação de acesso à rede de serviços de saúde, com baixa suspeição diagnóstica e detecção de casos e com limitação da qualidade de vida das pessoas acometidas. ^91^ Para tanto, foram desenvolvidos três subíndices a partir dos três indicadores integrados na análise anterior. ^91 , 94^ O valor do índice pode variar no intervalo entre 0 e 1, sendo que quanto mais próximo do valor ‘1’, maior a vulnerabilidade para DC crônica ( Figura 2.1 ). ^91^

Fonte: CGZV/Deidt/SVS

 Uma perspectiva adicional da vigilância da DC no Brasil remete à recomendação para que toda pessoa com infecção pelo vírus da imunodeficiência humana (HIV) ou síndrome da imunodeficiência adquirida (AIDS) tenha, à disposição, a solicitação de teste de anticorpos anti- *T. cruzi,* com base na existência de antecedente epidemiológico. Essa recomendação também tem sido debatida mais recentemente em outros países, como nos EUA. ^95^ Ressalta-se que, desde 2004, o Brasil inseriu a RDC na lista de doenças indicativas de AIDS, na vigência de infecção por HIV, para fins de vigilância epidemiológica a partir do diagnóstico definitivo de meningoencefalite e miocardite associadas à DC. ^2 , 83 , 84^

### 2.5. Associação de Doença de Chagas com COVID-19

 A emergência da COVID-19, causada pelo coronavírus da síndrome respiratória aguda grave 2 (SARS-CoV-2), trouxe desafios críticos e sem precedentes globalmente para os sistemas nacionais de saúde e para a humanidade em geral. ^42 , 80 , 96 , 97^ O caráter pandêmico foi amplificado em pouco tempo por sua alta infectividade, mesmo em fases assintomáticas da doença, fato que levou à sua rápida disseminação. ^98 , 99^

 À medida que a pandemia global da COVID-19 avança, impacta desproporcionalmente cada vez mais as populações com elevada vulnerabilidade social, ^80 , 98 , 99^ que já carregam uma carga de morbimortalidade considerável para DTN. Dessa forma, a análise do atual contexto de DTN oferece possibilidades relevantes para abordar lacunas do controle da COVID-19, pois representa referencial importante para o progresso na resposta às necessidades das populações mais vulneráveis. O sucesso na resposta ao controle da COVID-19, sem estar acompanhado por redução da carga de DTN, sinaliza falhas na sustentabilidade dos sistemas nacionais de saúde para manter esse controle. ^97^

 A concomitância de DC é particularmente preocupante por causar, potencialmente, complicações cardíacas, gastrointestinais, neurológicas e outras, ampliando a suscetibilidade à COVID-19. ^71 , 100 - 102^ De fato, a maior prevalência de comorbidades parece estar relacionada a um pior prognóstico na coinfecção. ^102^ Desde o surgimento da pandemia causada pelo SARS-CoV-2, o envolvimento cardiovascular tem sido identificado como complicação frequente da COVID-19. ^96 , 100^ Entretanto, há ainda poucas evidências sobre os efeitos da COVID-19 em pessoas acometidas pela DC. ^100 , 102 - 104^ Alguns estudos indicam que a COVID-19 pode trazer novos desafios relativos à garantia de acesso à atenção integral (diagnóstico e tratamento, assim como longitudinalidade do cuidado) a essas pessoas, bem como ao necessário desenvolvimento de novas pesquisas no futuro para análise das implicações da coinfecção com SARS-CoV-2. ^97 , 103^

 Em adição, verifica-se que as duas doenças apresentam semelhanças relativas à suscetibilidade e aos fatores de risco, padrões moleculares associados ao patógeno, reconhecimento de glicosaminoglicanos, processo de inflamação, hipercoagulabilidade vascular, microtrombose e endoteliopatia, podendo, assim, requerer tratamentos com princípios semelhantes. ^105^ Entretanto, ressalta-se a importância de se considerarem as diversas formas clínicas da doença e os mecanismos fisiopatológicos específicos a elas associados. ^102^ Assim, não obstante alguma similaridade quanto à fisiopatologia, que envolve risco elevado de tromboembolismo na COVID-19 e na cardiomiopatia crônica da doença de Chagas (CCDC), demanda-se cautela quanto à recomendação de tratamento imediato da DC com fármacos anticoagulantes, restringindo-se o benefício potencial dessa conduta a cenários clínicos em que uma adequada relação de riscos de hemorragia *versus* trombose seja individualizadamente favorável ao uso desses fármacos. Tais princípios são discutidos de forma pertinente em outro capítulo desta diretriz. 

 Alguns estudos têm apontado para altos níveis de comorbidades em casos com DC associada a formas graves de COVID-19. É importante ressaltar que essas comorbidades também refletem a idade mais avançada das populações que são especialmente impactadas pela DC e pela COVID-19. ^71 , 100 - 102^ Embora mais de 80% dos casos de COVID-19 sejam leves ou assintomáticos, casos graves têm sido mais frequentes entre pessoas idosas e com comorbidades, enquanto que, para a DC, pessoas idosas com cardiomiopatia crônica apresentam maior risco de morte, justificado, em parte, pela associação com idade ou outras condições crônicas, mas também pela condição de pobreza social. ^71 , 100^

 Embora a coinfecção possa estar associada a maior risco potencial de complicações, com pior prognóstico clínico, achados de um estudo multicêntrico prospectivo com 37 hospitais em 17 municípios de 5 estados brasileiros (Minas Gerais, Pernambuco, Rio Grande do Sul, Santa Catarina e São Paulo) indicam não ter havido diferenças significativas na apresentação clínica e nos desfechos de casos com DC em comparação a controles, a despeito da evidência no início do estudo de maior frequência de IC crônica e fibrilação atrial (FA). Além disso, nesse estudo foi observado nível mais baixo de proteína C reativa entre participantes com DC. ^104^

 A maior vulnerabilidade social de pessoas acometidas por DC em contexto de pobreza pode ser ainda mais ampliada com a COVID-19, por seus impactos político-econômicos. ^80 , 97 - 99^ O significativo aumento da pobreza extrema globalmente na última década traz consigo a ameaça de tornar o acesso à saúde ainda mais crítico para pessoas acometidas por DC. ^71 , 101 , 106^

 Por outro lado, pessoas acometidas pela DC podem ter receio de procurar atendimento por medo de exposição à COVID-19, retardando a busca de solução para complicações relacionadas à doença e ampliando a carga emocional da doença pelas preocupações associadas. Acresce-se o cenário de enfraquecimento, desestruturação e sobrecarga dos sistemas nacionais de saúde. ^71^

 O Brasil é um dos países com maior carga de morbimortalidade por COVID-19 e tem se destacado negativamente no cenário internacional pela falta de coordenação e liderança das ações de vigilância e controle da COVID-19. ^92 , 99 , 107^ Por outro lado, a desigualdade na expressão da COVID-19 no país tem sido demarcada, por exemplo, pelo excesso de mortalidade entre negros/pardos em todas as faixas etárias dessa população. ^99 , 108^ Essas disparidades raciais podem ser justificadas por condições socioeconômicas historicamente determinadas, que muitas vezes definem quem é capaz de se manter em distanciamento social e evitar a exposição ao SARS-CoV-2. ^71 , 99 , 108^

 Verificou-se ainda forte gradiente para o risco de morte por COVID-19 durante os estágios iniciais da pandemia, ampliando a vulnerabilidade de áreas periféricas, onde se encontram comunidades mais vulneráveis, colocando em risco a capacidade de resposta do sistema de saúde e aumentando as desigualdades em atenção à saúde. ^99 , 106^

 Por intermédio da nota informativa nº 9 de 2020 (CGZV/DEIDT/SVS/MS) foram estabelecidas no Brasil recomendações do Ministério da Saúde para adequações das ações de vigilância e atenção às pessoas acometidas por DC frente à situação epidemiológica da COVID-19. ^109^ A despeito dessas orientações, a possibilidade de ocorrência de impacto da pandemia por COVID-19 frente ao perfil de morbimortalidade e às ações de vigilância da doença no país foi levantada como hipótese em Boletim Epidemiológico específico do Ministério da Saúde. ^93^

 Nesse documento, são trazidas evidências que apontam as doenças cardiovasculares como fatores de risco críticos para maior gravidade da síndrome clínica associada à COVID-19. Com base nesses aspectos, ressalta-se o fato de que as pessoas acometidas por DC devam ser consideradas também como população com maior risco para pior evolução clínica da COVID-19, demandando maior cuidado e atenção pelo SUS no contexto pandêmico. ^93^

 Ainda em caráter preliminar, considerando-se o período de março a agosto de 2020, aquele boletim epidemiológico indica que foram registrados no país 1.746 óbitos em que a DC foi inserida como causa básica (dados oriundos do Sistema de Informação sobre Mortalidade), dos quais 29 mencionam a COVID-19 ou Síndrome Respiratória Aguda Grave como condição que agravou ou contribuiu direta ou indiretamente na cadeia causal do óbito (partes I e II da Declaração de Óbito), com maior proporção nas regiões Sudeste e Nordeste. ^93^ Naquele mesmo período, foram registrados 125.691 óbitos por COVID-19, dos quais em 207 (0,2%) havia menção à DC como condição que contribuiu para a morte (parte II da Declaração de Óbito), com maior proporção nas regiões Sudeste e Nordeste. A maioria desses óbitos ocorreu em pessoas do sexo feminino (52,7%), de raça/cor parda (42,0%), com média de 74 anos de idade (DP±11,36) e faixa etária acima de 75 anos (53,0%). ^93^

 Existem hipóteses que apontam a coinfecção *T. cruzi* e SARS-CoV-2 como importante binômio causal não investigado de morte em regiões endêmicas para a DC. ^101^

 A análise de tendência temporal regionalizada no país, de 2009 a 2019, revela propensão a redução estatisticamente significativa quanto ao coeficiente de mortalidade específica pela doença. Entretanto, verificou-se tendência de aumento do coeficiente de incidência de casos na fase aguda, estatisticamente significativa para a região Norte; contudo, em 2020, o número de casos registrados foi inferior ao previsto. ^93^

 Em termos de diagnóstico, verificou-se redução de 24% no número de requisições de exames laboratoriais para diagnóstico da DC que foram processadas nesse período de 2020, em comparação com a média verificada de 2017 a 2019. ^93^ Esse cenário de redução também foi verificado em relação ao tratamento, avaliado por meio da redução da distribuição do benznidazol, e também pela avaliação da vigilância entomológica junto a coordenações estaduais, ^93^ indicando possível redução da sensibilidade da rede de atenção e vigilância em saúde, provavelmente relacionada ao direcionamento de esforços municipais e estaduais para o enfrentamento da pandemia por COVID-19. 

 Mesmo com as orientações acerca da necessidade de readaptação das atividades de vigilância entomológica no contexto da COVID-19, ^109^ os relatos e informes de representantes estaduais indicam que em muitos territórios não foi possível realizar, mesmo que parcialmente, as atividades de controle previstas para o ano de 2020. ^93^

 Finalmente, como há evidências recentes de que persistam a longo prazo sequelas cardiovasculares em pessoas acometidas pela COVID-19, ^110^ isso poderá ser ainda mais ominoso no contexto daquelas já afligidas pela CCDC ao se infectarem pelo SARS-CoV-2. 

### 2.6. Reflexão Final sobre o Cenário Epidemiológico Atual Relativo à Doença de Chagas 

 Publicações recentes, tanto por investigadores e gestores de países não endêmicos ^111 , 112^ como por aqueles onde a DC ainda é endêmica, ^113 , 114^ indigitam a premente necessidade de se adotarem políticas abrangentes em termos de saúde pública para controle eficaz da transmissão inter-humanos da infecção pelo *T. cruzi* e se alcançar um nível otimizado de atendimento aos indivíduos já infectados, com foco em oportunização tanto diagnóstica como terapêutica. 

## 3. Patogênese da Cardiomiopatia da Doença de Chagas 

### 3.1. Introdução

 A patogênese da CCDC ainda é objeto de intenso debate. Enquanto na fase aguda da DC o intenso parasitismo tissular foi sempre reconhecido como mecanismo essencial, na fase crônica isso não ocorreu e outras hipóteses patogenéticas predominaram durante a segunda metade do século XX. Foi somente a partir dos anos 2000 que se consolidou a noção de que a persistência parasitária no miocárdio seja o mecanismo primordial também para que se instale a CCDC. Isso resgatou o conceito da DC como verdadeira entidade infecciosa e da CCDC como causada por processo inflamatório focal de baixa intensidade, porém virtualmente incessante. A agressão tissular, causando necrose e fibrose reativa e reparativa, por sua vez, é diretamente estimulada pelo *T. cruzi* e por reação imune adversa à persistência parasitária. 

 Entre outras noções, deve-se reconhecer que o prognóstico da CCDC é em geral mais ominoso do que o das cardiomiopatias não inflamatórias. A identificação dos fatores prognósticos e dos alvos terapêuticos é criticamente dependente desse conhecimento. A lise direta das células infectadas é relevante principalmente durante a fase aguda da infecção, quando os parasitas intracelulares são abundantes e a miocardite costuma ser difusa e intensa. Já os indivíduos cronicamente infectados apresentam progressão nitidamente diferencial da doença. Décadas após a infecção, cerca de 60% dos indivíduos infectados permanecem livres de manifestações clínicas da doença por toda a vida (estágio A - FIDC), 10% desenvolvem doença gastrointestinal e 30% desenvolvem CCDC, que pode apresentar estágios B1/B2 (cardiomiopatia menos avançada) ou C/D (cardiopatia grave), conforme será visto em outro capítulo desta diretriz. 

 As principais hipóteses patogênicas propostas para explicar o início e a progressão da CCDC incluem: 1) danos diretos aos tecidos induzidos por parasitas; 2) danos indiretos inflamatórios/imunológicos aos tecidos; 3) distúrbios neurogênicos; 4) distúrbios microvasculares. A hipótese neurogênica foi embasada na depleção neuronal intracardíaca e na consequente disautonomia, mas há obstáculos incontornáveis para a postulada cardiopatia “parassimpaticopriva”. As evidências em modelos experimentais e na doença humana indicam que os infiltrados inflamatórios são os principais causadores de dano ao tecido cardíaco. Mas, também, evidências mais recentes mostram que a suscetibilidade genética e os danos mitocondriais são partes importantes da patogênese da CCDC. Foram relatadas lesões microcirculatórias cardíacas na CCDC, mas a isquemia microvascular pode ser consequência da ação de mediadores inflamatórios e constituir mecanismo de *feedback* positivo, potencializando os danos inflamatórios e mitocondriais, como discutido a seguir. 

### 3.2. Dinâmica Imune e Progressão Diferencial para Cardiomiopatia Crônica da Doença de Chagas 

 Na fase aguda da infecção, que tem sido investigada mais detalhadamente em modelos murinos, a parasitemia e o parasitismo intenso dos tecidos desencadeiam forte resposta imunológica. Ocorre inicialmente resposta imune inata, logo seguida pela que depende de linfócitos T citotóxicos e linfócitos T produzindo citocinas inflamatórias como interferon-gama (IFN-γ) e fator de necrose tumoral alfa (TNF-α), juntamente com anticorpos anti- *T. cruzi* específicos que controlam parcialmente o parasitismo, estabelecendo infecção persistente embora de baixo grau. ^115 , 116^

 Observa-se que diferentes linhagens de camundongos infectados com a mesma linhagem de *T. cruzi* mostram graus distintos de gravidade de CCDC, caracterizados por alterações eletrocardiográficas e ecocardiográficas, associadas a variados níveis séricos de TNF-α e óxido nítrico, sugerindo que variações na genética do hospedeiro possam condicionar a gravidade da doença crônica. ^117 , 118^

 A estimulação parasitária persistente induz produção sistêmica de IFN-γ e TNF-α em indivíduos com DC crônica, que é particularmente intensa naqueles com CCDC em comparação aos que apresentam a FIDC. ^119 , 120^ Propõe-se existir relação entre a intensidade da fase aguda e a gravidade da fase crônica da infecção por *T. cruzi* . Pacientes com CCDC apresentam miocardite difusa (rica em macrófagos, linfócitos citotóxicos CD8+ e linfócitos CD4+ T) com fibrose e hipertrofia. A miocardite é devida tanto aos linfócitos *T. cruzi* específicos como aos linfócitos T autoimunes, que produzem grandes quantidades de IFN-γ e TNF-α. IFN-γ desempenha papel patogênico central na CCDC ao induzir danos celulares por vários mecanismos, enquanto outros mediadores inflamatórios também atuam relevantemente. 

 Recente revisão sobre alterações imunológicas sistêmicas e específicas do coração revelou que os pacientes com CCDC apresentam característico perfil inflamatório de citocinas. ^115^ Foram observados efeitos imunológicos sistêmicos significativos no sangue periférico de pacientes com DC crônica, que estão associados com as distintas formas clínicas. É importante notar que diferenças qualitativas são claramente observadas nas respostas celulares sistêmicas de pacientes com formas clínicas indeterminada e cardíaca. Essas diferenças estão sob a influência de uma rede imunorreguladora de citocinas, que orquestra a resposta imunológica. Enquanto os indivíduos com a FIDC apresentam perfil imunorregulatório equilibrado e modulado pela produção de interleucina (IL)-10, ^121^ os pacientes com CCDC apresentam frequência aumentada de CD4+ e CD4-CD8- células T produtoras de IFN-γ, assim como de níveis aumentados de TNF-α circulantes no sangue periférico. ^122^

**Figura 3.1:**
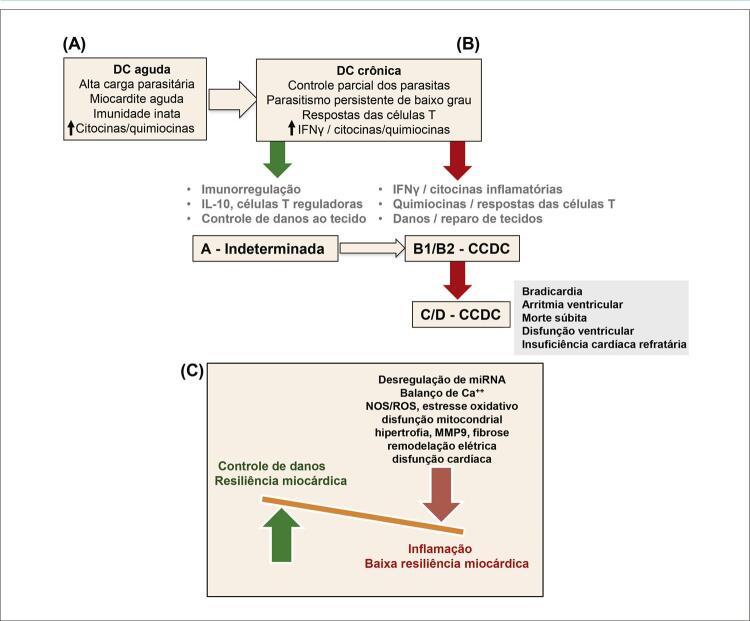
– Eventos patogênicos na progressão da cardiomiopatia crônica da doença de Chagas (CCDC). (A): eventos na fase aguda da doença de Chagas (DC); (B): eventos patogênicos primários na fase crônica da DC, com indicação também dos estágios e manifestações clínicas fundamentais (vide capítulos de história natural da DC nesta diretriz); (C) eventos e distúrbios fisiopatológicos em fases mais avançadas da DC. miRNA: microRNA.

 Além disso, a expressão de IFN-γ é aumentada em pacientes com a forma de CMD na DC em comparação com CCDC ainda com a forma não dilatada. ^123 , 124^ Por outro lado, os pacientes com CCDC apresentam frequências reduzidas de células T Th17 circulantes ^125 , 126^ e de monócitos produtores de IL-10, ^127^ células T reguladoras CD4+CD25+ (Tregs), ^128 - 132^ bem como níveis reduzidos de Ebi/IL-27p28 ^133^ em comparação a indivíduos com a FIDC ( Figura 3.1 ). Essas alterações imunorregulatórias correlacionam-se à depressão contrátil, já que a alta frequência de células produtoras de IFN-γ e TNF-α está associada à baixa FEVE. ^134 , 135^

 Por outro lado, uma maior frequência de células produtoras de IL-17 e IL-10 está associada à preservação de valores normais da FEVE. ^136 - 138^ Células B autorreativas ^139^ e subpopulações de células B associadas a respostas potencialmente protetoras ou patogênicas foram identificadas em pacientes com DC. ^140^ Além disso, a produção de anticorpos líticos antiparasitários foi proposta como um mecanismo de controle de parasitas. ^141^ A ativação de monócitos, ^127 , 142 , 143^ células T CD4+ com receptores específicos de células T, ^136 - 138^ células T CD8+ ^144 - 147^ e outras populações de células T menos numerosas, mas muito ativas, tais como CD4-CD8- células T, foi demonstrada em pacientes com DC. ^135 , 148^

 A resposta Th1 exacerbada no sangue periférico de pacientes com CCDC reflete-se no infiltrado inflamatório rico em Th1 predominantemente secretando IFN-γ e TNF-α, com menor produção de IL-4, IL-6, IL-7, IL-15, IL-18, como evidenciado por estudos de imuno-histoquímica e expressão de mRNA. ^123 , 149 - 153^ De fato, IFN-γ é a citocina mais *up-regulada* no tecido cardíaco do paciente com CCDC. Assim, observa-se a expressão significativa do T-bet, o fator de transcrição Th1, no miocárdio desses pacientes com CCDC. ^154^

 Além disso, encontra-se correlação positiva entre a expressão de T-bet e a dilatação do ventrículo esquerdo (VE), corroborando o papel patogênico das células T produtoras de IFN-γ. Em contrapartida, a expressão de RNAm do GATA3, RORγT e FoxP3, subconjunto de células T que define fatores de transcrição das populações Th1-antagonizante Th2, Th17 e Treg, juntamente com suas assinaturas de citocinas IL-4, IL-13, IL-17, IL-10 e marcadores moleculares (FoxP3 e CTLA4), era baixa ou indetectável. ^154^

 As células T Th1 CCR5+ CXCR3+ produtoras de IFN-γ são mais abundantes em pacientes com CCDC do que naqueles com a FIDC, ^155^ e as mesmas células foram identificadas no tecido cardíaco de pacientes com CCDC, juntamente com seus ligantes de quimiocinas (CCL3-5, CXCL9 e CXCL10, respectivamente). CCL5 e CXCL9 foram as quimiocinas mais expressas e a intensidade da inflamação miocárdica foi positivamente correlacionada com a expressão do RNAm de CXCL9. ^151 , 156^

 Em modelos animais de CCDC, nas fases aguda e crônica da infecção pelo *T. cruzi* , CCL3, CCL4 e CCL5, agindo via CCR1 ou CCR5, controlam a migração das células T e macrófagos para o tecido cardíaco, levando à lesão cardiomiocitária, anomalias de condução e disfunção ventricular. ^157 , 158^ Em conjunto, isso sugere que as quimiocinas quimioatrativas Th1 produzidas localmente desempenhem papel significativo no acúmulo seletivo de células T Th1 no coração com CCDC. Além disso, indica essencialmente não haver regulação por células T ou citocinas reguladoras no miocárdio infiltrado por Th1 de pacientes com CCDC. 

 Por sofrer pouca regulação, isso poderia explicar a destrutividade do infiltrado inflamatório, muito provavelmente devido aos danos colaterais excessivos causados pelas células T produtoras de IFN-γ. Acredita-se que a ação não antagônica ao IFN-γ no paciente com CCDC esteja ligada ao fato de que as células T produtoras de IL10, Ebi/IL27R e reguladoras, todas capazes de suprimir a produção do IFN-γ e/ou a diferenciação das células T Th1, encontram-se diminuídas. 

### 3.3. Disfunção Mitocondrial Miocárdica e Cardiomiopatia Crônica da Doença de Chagas 

 Wan *et al* . foram os primeiros a implicar disfunção mitocondrial miocárdica e estresse oxidativo na patogênese da CCDC em modelos murinos. ^115 , 158^ A notável semelhança entre os distúrbios cardíacos, digestivos e autonômicos nas mitocondriopatias (15% desenvolvem distúrbios de motilidade gastrointestinal e 40% desenvolvem cardiomiopatia e arritmia), ^159 , 160^ bem como o amplo espectro clínico da DC sintomática, ^161^ sugerem que a patogênese da CCDC possa ter como componente fundamental a disfunção mitocondrial. 

 De fato, o miocárdio na CCDC apresenta sinais de redução da atividade mitocondrial e da produção de energia. Redução do RNA ribossomal mitocondrial ^151^ e do DNA mitocondrial, ^162^ assim como outras observações (não publicadas) e produção *in vivo* de adenosina trifosfato (ATP), ^163^ foram descritas no miocárdio do paciente com CCDC. 

 Os níveis miocárdicos e a atividade das enzimas do metabolismo energético mitocondrial ATP-sintase e creatina-quinase são ainda mais baixos do que em outras cardiomiopatias, ^164^ o que poderia contribuir para o pior prognóstico associado à CCDC. A descoberta da associação de CCDC com variantes raras de genes mitocondriais, descritas com mais detalhes abaixo neste capítulo, corrobora o papel da disfunção mitocondrial no dano miocárdico do paciente com CCDC e pode ser um mecanismo para perpetuação da inflamação e dano cardiomiocitário. ^115 , 161^

 Estudos recentes mostraram a modulação da expressão de alguns microRNAs (miRNAs), moléculas que controlam especificamente a tradução do RNAm, no tecido cardíaco de pacientes com CCDC ^165 , 166^ e na infecção murina aguda por *T. cruzi* . ^167^

 As descobertas em camundongos infectados com *T. cruzi* geneticamente deficientes em microRNA-155 também apoiam a relação do miRNA com o controle da infecção e a produção de citocinas inflamatórias. ^168^

### 3.4. Genética na Cardiomiopatia Crônica da Doença de Chagas 

 A verificação de que apenas cerca de 30% dos pacientes com DC desenvolvem a cardiomiopatia crônica, bem como a agregação familiar de casos de CCDC, ^169^ sugeriu a participação de fatores genéticos na progressão diferencial da doença. Os pacientes com CCDC apresentam resposta inflamatória mais intensa do que aqueles com FIDC, que parecem ter resposta imunológica mais bem regulada. 

 Dada a importância dos mecanismos inflamatórios na patogênese da CCDC, muitos estudos focaram nos polimorfismos “comuns” ou frequentes nos genes relacionados às respostas inflamatórias e imunológicas, que assim acarretariam importantes variações na expressão de citocinas inflamatórias e quimiocinas envolvidas na patogênese da doença. Cada polimorfismo comum ou frequente é tipicamente responsável por pequenos efeitos fenotípicos (cerca de 10% da população/fenótipo). 

 Revisão recente revelou 145 estudos de associação abordando polimorfismos candidatos em 76 genes, encontrando 62 polimorfismos de nucleotídeos simples (SNP) de 44 genes a serem associados ao fenótipo da CCDC. ^115^ Desses, SNP em 8 genes foram associadas com a gravidade da CCDC: SNP nos genes IL17a, IL18, IL27b/Ebi3, CCR2, CXCL9, CXCL10 e MICA foram mais frequentes entre os pacientes CCDC com disfunção ventricular esquerda significativa (FEVE < 40%) em comparação aos demais pacientes com CCDC. 

 Foram realizados dois estudos de associação do genoma utilizando a técnica GWAS ( *Genome Wide Association Study* ), comparando CCDC e FIDC, um em 2013 ^170^ envolvendo 600 pacientes com DC e outro em 2021 envolvendo 3413 indivíduos; ^171^ apenas esse último revelou uma única variante significativa para todo o genoma (p < 10 ^-8^ ) perto do gene SAC3D1. 

 Estudo recente abordou o papel de variantes genéticas raras na progressão para CCDC em famílias nucleares com múltiplos casos de DC usando sequenciamento de exomas inteiros. ^172^ Nas seis famílias estudadas, foram encontradas 22 variantes patogênicas heterozigotas raras e não sinônimas de alto impacto, associadas à CCDC, localizadas em 20 genes. Somente indivíduos soropositivos e portadores das variantes genéticas patogênicas desenvolveram CCDC, mas não pacientes soropositivos não portadores das variantes genéticas, nem irmãos soronegativos portadores da variante patogênica. Um acúmulo impressionante de variantes específicas da CCDC (86%) ocorreu em genes mitocondriais ou relacionados à inflamação e todas as famílias estudadas apresentaram pelo menos um gene de variante associada à CCDC pertencente a essas vias. Os resultados desse estudo indicaram que a contribuição genética para causar CCDC é poligênica e mediada por diversas variantes raras em genes que diferem entre famílias, mas que estão relacionados com alterações em mitocôndrias e com inflamação. 

**Figura 3.2:**
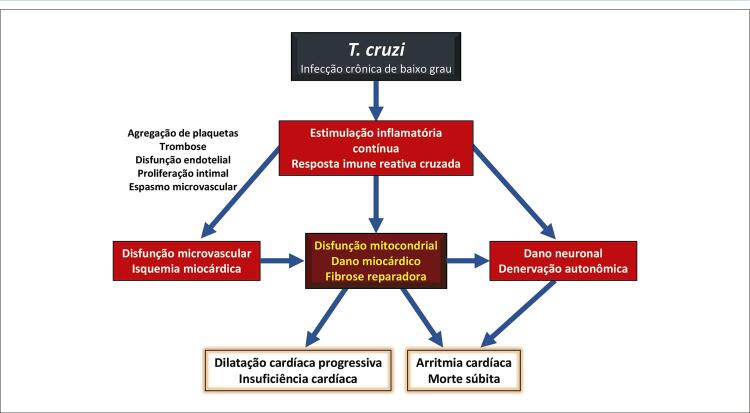
– Interações entre mecanismos imunes, microvasculares e neurogênicos na cardiomiopatia crônica da doença de Chagas.

 Os resultados implicam que a disfunção e inflamação mitocondrial, processos-chave na fisiopatologia da CCDC, sejam, pelo menos em parte, determinados geneticamente. Isso pode ser dependente de mecanismo de dupla agressão. Dessa forma, o IFN-γ e citocinas pró-inflamatórias induzidas por infecção crônica desencadeariam disfunção mitocondrial e doença clínica em pacientes com variantes que causam comprometimento subclínico da função mitocondrial em órgãos de alta demanda metabólica, como coração e células neuronais ganglionares mioentéricas. Lesão mitocondrial pode constituir mecanismo de perpetuação de alterações inflamatórias tissulares visto que há liberação de componentes internos por mitocôndrias danificadas pela resposta imune inata. A Figura 3.2 mostra os destaques dos pontos-chave inflamatórios associados à progressão da CCDC. 

### 3.5. Distúrbio Microvascular Coronário

 Há evidências crescentes, no campo tanto clínico como experimental, da participação das anormalidades microvasculares coronárias como mecanismo patogênico da CCDC. Vários estudos indicam que a lesão miocárdica possa ser consequente a alterações microvasculares, fundamentalmente associadas a inflamação e que levam a isquemia e necrose miocárdica, com eventual fibrose reparadora. ^173 - 176^

 A primeira evidência de que distúrbios da microcirculação coronária possam participar dos mecanismos de lesão miocárdica na DC em humanos foi obtida em estudos necroscópicos descrevendo alterações vasculares intensas, com hiperproliferação intimal, espessamento parietal e obstrução de pequenas arteríolas coronárias intramurais em corações de pacientes com CCDC. ^177 , 178^ Além disso, as fibras miocárdicas nas proximidades das lesões vasculares apresentavam necrose miocitolítica, uma lesão celular intimamente relacionada a isquemia miocárdica. 

 Em estudo mais recente, Higuchi *et al* . descreveram alterações estruturais intensas da microcirculação coronária com dilatação vascular e rarefação em corações de pacientes com CCDC, que eram diversas das geralmente observadas em pacientes com CMD idiopática. ^179^

 Assim, as observações necroscópicas sugerem fortemente a participação da isquemia microvascular na gênese dos focos inflamatórios e da miocitólise, que levam a fibrose reparadora e que são as características histopatológicas fundamentais da CCDC. 

 No cenário clínico, estudos utilizando cintilografia de perfusão miocárdica mostraram elevada prevalência (30% a 50%) de defeitos perfusionais em pacientes com CCDC e artérias coronárias angiograficamente normais, sugerindo fortemente a presença de disfunção microvascular coronária. ^180 - 183^ Vários estudos também mostraram que os defeitos de perfusão miocárdica estavam topograficamente relacionados ao comprometimento do movimento da parede regional do VE, ocorrendo em pacientes em fases iniciais da CCDC e sem outras evidências de envolvimento cardíaco, ^183^ sugerindo que a isquemia microvascular seja distúrbio precoce na evolução da doença, precedendo a disfunção ventricular regional e possivelmente relacionado à indução de hibernação ou atordoamento miocárdico. Resultados similares foram obtidos por estudos com doppler-ecocardiografia, mostrando diminuição da reserva vasodilatadora coronária, um índice de disfunção microvascular, em pacientes com FIDC quando comparados a controles normais. ^184^

 Também os resultados de estudo retrospectivo longitudinal utilizando cintilografia de perfusão miocárdica em pacientes com CCDC mostraram que a isquemia microvascular está topograficamente relacionada com áreas que, em última instância, desenvolvem fibrose miocárdica durante a progressão da doença. Esses resultados corroboram a hipótese de que a isquemia microvascular possa estar diretamente envolvida no mecanismo que leva à fibrose regional e à progressão da disfunção sistólica do VE na CCDC. ^181^

 Estudos mais recentes em modelo experimental de hamsters sírios cronicamente infectados por *T. cruzi* mostraram estreita relação topográfica entre defeitos de perfusão miocárdica em repouso, utilizando cintilografia de perfusão miocárdica de alta resolução *in vivo* , com inflamação histologicamente verificada e disfunção sistólica ventricular esquerda regional/global. ^185^ Além disso, a tomografia computadorizada por emissão de pósitrons (PET/TC) com ^18^ F-fluordesoxiglicose ( ^18^ F-FDG) confirmou que as regiões com hipoperfusão miocárdica em repouso correspondiam às áreas com miocárdio viável e inflamação em outra investigação nesse modelo experimental. ^186^

 Ainda outro estudo recente no mesmo modelo de hamsters cronicamente infectados pelo *T. cruzi* mostrou que o uso prolongado do dipiridamol, um agente vasodilatador da microcirculação coronária, estava associado à redução significativa dos defeitos de perfusão miocárdica de repouso, apoiando indiretamente a presença de miocárdio viável, mas hipoperfundido, causado pela disfunção da microcirculação coronária na CCDC experimental. ^187^

 Os mecanismos potencialmente envolvidos na gênese de disfunção microvascular coronariana na CCDC são: 1. Alterações funcionais na árvore coronária, com aumento da vasorreatividade e espasmo dos pequenos ramos arteriais intramurais; ^188 , 189^ 2. Lesões endoteliais causadas diretamente pela agressão parasitária; ^190^ 3. Alterações funcionais e estruturais induzidas por substâncias secretadas pelo infiltrado inflamatório no tecido miocárdico próximo aos microvasos coronários, principalmente endotelina e citocinas. Esse mecanismo tardio é ainda apoiado por estudos que evidenciam que as alterações da inflamação miocárdica estão associadas à ocorrência de *plugs* plaquetários, à proliferação obstrutiva da íntima vascular e a espasmo microarteriolar. ^191^

### 3.6. Denervação Cardíaca

 A denervação autonômica cardíaca é característica proeminente da CCDC e foi descrita pela primeira vez em estudos de autópsia em humanos mostrando intenso despovoamento neuronal intramural, superior ao observado em qualquer outra doença cardiovascular. ^192 , 193^ Essas descobertas foram corroboradas por estudos em animais experimentalmente infectados com o *T. cruzi* , que demonstraram parasitismo neuronal cardíaco associado com periganglionite e anormalidades degenerativas em células de Schwann e fibras nervosas. ^194 , 195^ Importante enfatizar que essa despopulação neural também acomete os gânglios intramurais de vários órgãos do sistema digestório, entre os quais avultam o esôfago e o cólon, sendo esse fato claramente incriminado na fisiopatologia do megaesôfago e do megacólon da DC. 

 Postulou-se que a despopulação neuronal na CCDC ocorra durante a fase aguda da infecção, secundária ao parasitismo direto dos neurônios, degeneração causada pela inflamação periganglionar e reação autoimune antineuronal. ^196 , 197^ Há também indícios de que o dano pode prosseguir na fase crônica devido a inflamação localizada. 

 Diversas anormalidades funcionais do controle autonômico reflexo da frequência cardíaca (FC) em pacientes com CCDC foram descritas como consequência da denervação autonômica cardíaca anatomicamente detectada. ^198 - 201^ Pacientes com CCDC apresentam privação da ação inibitória tônica do sistema parassimpático no nó sinusal e falta do mecanismo vagalmente mediado para responder com bradicardia rápida ou taquicardia a mudanças transitórias na pressão sanguínea ou no retorno venoso. ^202^ A disautonomia em pacientes com CCDC pode ser detectada antes do desenvolvimento da disfunção ventricular, bem como em estágio precoce da fase crônica e mesmo nas formas indeterminada e digestiva da DC. ^203 , 204^

 Mais recentemente, a cintilografia miocárdica com meta-iodo-benzil-guanidina marcado com iodo-123 ( ^123^ I-MIBG) foi empregada em pacientes com CD para fornecer informações precisas sobre a integridade das fibras nervosas simpáticas na intimidade do miocárdio ventricular esquerdo. ^183^ Nesse estudo, 37 pacientes foram investigados com imageamento por ^123^ I-MIBG e os resultados foram correlacionados com a perfusão miocárdica e a perda regional de mobilidade parietal do VE. Defeitos de captação de ^123^ I-MIBG foram observados na maioria dos pacientes: em 33% daqueles sem evidência de cardiopatia ao ECG e ECO e em 77% daqueles com distúrbio regional da movimentação parietal ventricular. Além disso, os pacientes com disfunções ventriculares mais graves tinham também maior prevalência de defeitos de captação do ^123^ I-MIBG (92%). Notavelmente, havia nítida correlação topográfica entre áreas de denervação simpática miocárdica, defeitos de perfusão miocárdica e anormalidades parietais segmentares do VE. 

 Outro estudo demonstrou forte concordância topográfica entre áreas de denervação miocárdica simpática utilizando cintilografia com ^123^ I-MIBG e áreas de miocárdio com hipoperfusão durante estresse. ^205^ Esses resultados indicaram que a denervação simpática é distúrbio precoce na fisiopatologia da CCDC, antes do desenvolvimento de anormalidades regionais de contração do VE ou de disfunção contrátil global. Essa hipótese foi corroborada pelos resultados de estudo independente evidenciando a absorção anormal de ^123^ I-MIBG na maioria dos pacientes com DC sem sinais de envolvimento cardíaco. ^206^

 Estudos clínicos também documentaram relação quantitativa entre a extensão da denervação miocárdica, usando imageamento com ^123^ I-MIBG, e o risco de arritmias ventriculares malignas. Esse aspecto, clinicamente muito relevante, por associar a presença e extensão da denervação simpática com a arritmia grave em pacientes com CCDC, é potencialmente implicado como mecanismo de morte súbita. ^207 , 208^

 Apesar de extensa documentação da conspícua denervação autonômica em estágios iniciais da DC e da recente demonstração de sua participação potencial em mecanismo que desencadeia arritmias ventriculares graves, a “teoria neurogênica” ainda carece de demonstração fundamental dos elos fisiopatológicas que ligam esses fenômenos às lesões miocárdicas essenciais da CCDC. 

 Também foi proposto que a denervação autonômica poderia se associar a espasmo microvascular coronário e desencadear isquemia miocárdica, levando eventualmente a necrose miocárdica. No entanto, esse mecanismo também aguarda por evidenciação mais claramente fundamentada. A Figura 3.2 mostra a interação da inflamação com os mecanismos microvasculares e neurogênicos. 

### 3.7. Considerações Finais

 A patogênese da CCDC ainda constitui enigma composto por inúmeros aspectos entrelaçados, de natureza complexa, ligados à variabilidade de patógenos e à genética e ao sistema imunológico do hospedeiro, como exposto nas Figuras 3.1 e 3.2 . Há também indícios recentemente aventados de que várias lacunas no conhecimento do próprio ciclo vital do parasito no hospedeiro humano e no vetor transmissor devam ser revisitadas e esclarecidas, permitindo que alvos mais apropriados para terapêuticas mais eficazes sejam delineados e aproveitados em pesquisas assim dirigidas. ^209^

## 4. Fisiopatologia da Cardiomiopatia – Fases Aguda e Crônica 

### 4.1. Introdução

 A fisiopatologia essencial da CDC pode ser assim resumidamente descrita: na fase aguda, a grande maioria dos indivíduos infectados pelo *T. cruzi* pode cursar com miocardite difusa, mas de intensidade baixa, que não se associa a graves distúrbios cardiovasculares e nem sequer é diagnosticada. Em raros pacientes, a inflamação aguda pode levar à perda significante da contratilidade miocárdica, com dilatação de câmaras e IC, com redução de fração de ejeção biventricular, às vezes, com distúrbios elétricos concomitantes (bloqueios de condução, extrassístoles) e derrame pericárdico. Tais alterações costumam ser autolimitadas em curso de poucas semanas, não causando, em geral, sequelas clinicamente manifestas. 

 Já o dano cardíaco na CCDC resulta das alterações fundamentais (inflamação, necrose e fibrose) que o *T. cruzi* provoca, direta ou indiretamente, no tecido especializado de condução, no miocárdio contrátil e no sistema autonômico intramural. 

 O frequente comprometimento do nó sinusal, do nó atrioventricular e do feixe de His, por alterações inflamatórias, degenerativas e fibróticas, leva à disfunção sinusal e a bloqueios variados atrioventriculares e intraventriculares. Por serem estruturas mais individualizadas, o ramo direito e o fascículo anterior-superior esquerdo são mais vulneráveis e mais frequentemente lesados. Focos inflamatórios e áreas de fibrose no miocárdio ventricular, especialmente em regiões apical, posterior-lateral e inferior-basal, podem produzir alterações eletrofisiológicas e favorecer o aparecimento de reentrada, principal mecanismo eletrofisiológico das taquiarritmias ventriculares malignas, que acarretam morte súbita mesmo em pacientes sem IC pregressa e sem grave disfunção sistólica de VE. 

 Outra consequência bastante comum das lesões miocárdicas é a disfunção biventricular, característica da CCDC. Inicialmente, há comprometimento regional, assemelhando-se ao que ocorre na cardiopatia por obstrução coronária, mas, paulatinamente, verifica-se dilatação e hipocinesia generalizada, em geral de ambos os ventrículos, conferindo padrão hemodinâmico de CMD à CCDC. Em fases mais avançadas da história natural, observa-se dilatação cardíaca global e notável aumento da massa do coração, o que se deve à combinação de hipertrofia miocárdica e fibrose em graus variáveis de paciente a paciente. 

 Desde as fases mais precoces, dissinergias ou aneurismas ventriculares predispõem a complicações tromboembólicas. Em estágios avançados, a dilatação global, a estase venosa e a FA são fatores adicionais que propiciam a formação de trombos e a consequente embolização pulmonar e sistêmica, como no sistema nervoso central, onde provocam o acidente vascular cerebral (AVC). Esse aspecto confere à CCDC, além das predominantes características de provocar arritmias malignas e IC refratária, a de ser precipuamente embolizante tanto no circuito pulmonar como em diversos órgãos sistêmicos, com infartos renais, esplênicos, mesentéricos ou nas artérias de membros, por exemplo. 

 Tais características da fisiopatologia própria da CCDC podem ser entendidas, em grande parte, como consequentes a importantes mecanismos patogênicos, como os abordados no capítulo específico da patogênese, e com ênfase adicional nos aspectos descritos a seguir. 

### 4.2. Parasitismo Miocárdico e Resposta Imune

 A DC, moléstia infecto-parasitária causada pelo protozoário *T. cruzi* , tem sua história natural dividida em fases aguda e crônica. ^37 , 46^ A fase aguda é usualmente oligossintomática e com sintomas inespecíficos, mas pode cursar com sintomas mais expressivos em cerca de 5-10% dos casos, quando há intensa parasitemia, ^210^ acompanhada por febre e lesão no local de inoculação do patógeno, e se complicar por meningoencefalite, miocardite, entre outras manifestações. 

 Cerca de quatro a oito semanas após a infecção, quando a parasitemia cai para níveis indetectáveis e os sintomas da fase aguda desaparecem, surge a fase crônica, que costuma durar várias décadas. Na fase crônica, cerca de 60-70% dos indivíduos não apresentam sintomas e os exames complementares de rotina, relacionados ao coração e ao aparelho digestivo, não demonstram alterações. Quando isso ocorre, configura-se para tais indivíduos a FIDC. ^211^ O restante dos pacientes cronicamente infectados desenvolve as formas determinadas, com acometimento cardíaco e/ou digestivo. ^46 , 212^

#### 4.2.1. Resposta Imune na Fase Aguda

 Desde a fase aguda, a DC tem fisiopatologia multifatorial e os mecanismos imunológicos, associados aos inflamatórios primários (desencadeados *pelo T. cruzi* em si), desempenham papel fundamental no processo. ^213 , 214^

 Na fase aguda, ocorre exposição das moléculas de superfície do *T. cruzi* aos receptores dos macrófagos e das células dendríticas, ocasionando pronta ativação das células envolvidas na imunidade inata, como neutrófilos e linfócitos NK ( *natural killers* ), que vão desencadear intensa resposta inflamatória visando a controlar a parasitemia. A ativação da imunidade inata gera intensa secreção de citocinas pró-inflamatórias, como o TNF-α, IFN-γ e diversas interleucinas, em especial IL-10. ^119 , 215^ Essa intensa resposta inflamatória pela ativação das células de imunidade inata e produção de mediadores pró-inflamatórios, apesar de ser decisiva para controlar a infecção, contribui para provocar lesão direta dos cardiomiócitos - também agredidos pelo usualmente conspícuo parasitismo tissular. Esse conjunto fisiopatológico configura a típica miocardite difusa da fase aguda da DC, que, na maioria dos casos, tem curso benigno e autolimitado. 

 Após a fase de intensa atividade inflamatória, ocasionando redução da parasitemia e do parasitismo tissular, os macrófagos e as células dendríticas que fagocitaram o *T. cruzi* desencadeiam a resposta imune humoral e celular, com ativação dos linfócitos B e T. Inicia-se, assim, a fase crônica na grande maioria dos pacientes que não conseguiram a eliminação total do parasita nessa janela de oportunidade da fase aguda. ^216^

#### 4.2.2. Resposta Imune na Fase Crônica

 A presença do DNA do parasita no miocárdio ^149 , 217^ e o reconhecimento cruzado por células T CD4+ de antígenos do *T. cruzi* e de sequências de aminoácidos existentes na miosina cardíaca constituem aspectos importantes envolvidos na fisiopatologia da disfunção miocárdica durante a fase crônica. ^218^

 Quanto à resposta imune celular, demonstrou-se que macrófagos infectados apresentam antígenos de *T.cruzi* de reação cruzada com o coração aos linfócitos T CD4+, que migram para o coração produzindo citocinas inflamatórias que levam a maior recrutamento e ativação de células do sistema imune, desencadeando reação de hipersensibilidade tardia. Dentre essas citocinas no infiltrado inflamatório, TNF-α e IFN-γ estão notadamente aumentados nos pacientes com CCDC. ^119 , 124^

 Estudo recente comparou diretamente a subpopulação linfocitária-T em indivíduos com CCDC e com CMD idiopática, evidenciando diferença nítida quanto ao perfil imunorregulador e maior ativação imunológica na CCDC, apesar de serem duas condições com características hemodinâmicas similares. ^219^

 Diversos são os fatores implicados na etiopatogenia da DC no coração, mas, independentemente dos mecanismos primordiais de agressão tissular, a via final comum é constituída pelo intenso infiltrado inflamatório e a fibrose miocárdica reativa e reparativa. A desorganização estrutural, geométrica e funcional do coração é resultado essencial da necrose miocárdica e consequente reposição por tecido fibrótico, agredindo o conteúdo perivascular e intersticial, importantes marcadores histopatológicos na DC. 

 Tais alterações são suficientes para causar dilatação e consequente disfunção contrátil biventricular, sendo a fibrose miocárdica de grau muito mais intenso quando comparada à de outras cardiomiopatias. Mecanismos complexos, como descritos, ativam cascata de resposta celular e molecular, intensificando a resposta inflamatória, o estresse oxidativo e a perda progressiva de cardiomiócitos por necrose e/ou apoptose, além de promover a sobrecarga e ulterior disfunção do miocárdio remanescente. ^200 , 220^

## 4.3. Alterações do Sistema Nervoso Autonômico na Doença de Chagas: Evidências de Estudos Histopatológicos 

 Alterações anatomopatológicas e funcionais do sistema nervoso autonômico foram descritas (com níveis variados de gravidade, desde os estudos primordiais de Carlos Chagas e seus colaboradores) em humanos e animais de experimentação. ^176 , 200 , 221 - 229^ Relatou-se que tais alterações são mais conspícuas em pacientes com a DC, comparativamente ao que ocorre, em menor grau, em outras cardiomiopatias. ^230 , 231^ Todavia, a despeito de constituírem aspecto dos mais marcantes na fisiopatologia da DC, o real papel etiopatogênico dessas alterações, inclusive as descritas no plexo intertruncal cardíaco, permanece imerso em incertezas. 

 Diretamente dependentes da infecção pelo *T. cruzi* , alterações como ganglionite, periganglionite, neurite e perineurite acarretam redução acentuada da densidade ganglionar e despovoamento neural em modelos animais experimentais ^223^ e em pacientes com a DC. ^224^ Postula-se, com base em estudos de modelos experimentais, que essas alterações anatomopatológicas no plexo intertruncal do coração ocorram predominantemente durante a fase aguda da infecção, ^225 , 226^ mas que continuem na fase crônica, ^227 , 228^ mesmo que com menor intensidade. Tais alterações decorrem de 4 fatores, atuando isoladamente ou em combinação: parasitismo direto de neurônios, ^229^ intenso processo inflamatório periganglionar, ^221^ reação antineural autoimune ^232^ e disfunção microvascular periganglionar. ^233^

 A agressão às estruturas autonômicas pode ser parcialmente compensada, pois os neurônios autonômicos mantêm, dentro de limites, certa capacidade de recuperação funcional. ^227^ Além disso, reinervação simpática foi relatada em humanos durante a fase crônica da DC após procedimentos como transplante cardíaco (TC) ^234^ e terapia com células-tronco. ^235^

 No entanto, a restauração das junções neuroefetoras funcionais, devido à regeneração axonal durante a fase crônica, é desorganizada, aleatória e incompleta. A inervação parassimpática apresenta comportamento análogo: ocorre destruição acentuada das fibras nervosas, com diminuição dos níveis de acetilcolina cardíaca durante a fase aguda, seguida de funcional restabelecimento de forma desorganizada, aleatória e incompleta durante a fase crônica. ^227^ Vários testes fisiológicos e farmacológicos evidenciam respostas funcionais anormais, coerentes com essa hipótese fisiopatológica. ^161^

 Como a despopulação neuronal ocorre predominantemente em gânglios intramurais parassimpáticos do coração e também dos plexos mientéricos, ^221 - 228^ avançou-se inicialmente a teoria de que no coração se instalaria uma cardiopatia “parassimpaticopriva” ou, em outros termos, haveria uma verdadeira “cardioneuropatia induzida por excesso relativo não antagonizado de catecolaminas”. ^221 , 223^ De acordo com essa teoria fisiopatológica, o coração, desprotegido pela ausência do efeito moderador parassimpático, estaria sujeito ao estresse de intensa estimulação tóxica do sistema adrenérgico. 

 Entretanto, várias evidências dificultam a comprovação de que uma “cardioneuropatia induzida por catecolaminas” contribua de forma decisiva para a patogênese na forma cardíaca da DC. Por outro lado, é virtualmente impossível descartar a possibilidade de que esse mecanismo não esteja envolvido no processo. Mais importante ainda, haveria indícios de que a via vagal-colinérgica desempenha papel fundamental direto na prevenção do envolvimento cardíaco que ocorre na DC. ^229^

 Entre as dificuldades antepostas à teoria “parassimpaticopriva” inclui-se a constatação de que, embora a disfunção vagal seja predominante, há concomitante atenuação da regulação adrenérgica do cronotropismo cardíaco mediado pelo nó sinusal. ^176 , 236^ Além disso, no nível miocárdico, a denervação simpática também é descrita em estudos de cintilografia cardíaca com I-MIBG. ^123^ Os distúrbios de captação deste radiotraçador, que reflete a integridade adrenérgica nesse nível ventricular, tendem a recrudescer à medida que a doença progride. ^205^ Tais investigações evidenciam forte associação entre áreas de denervação simpática, alterações da mobilidade parietal e hipoperfusão miocárdica em muitos pacientes, contribuindo para a instalação de arritmias potencialmente fatais. Em conjunto, esses estudos em humanos e em modelo experimental de infecção pelo *T. cruzi* no hamster sírio sugerem que a denervação autonômica simpática e a disfunção microvascular estejam intimamente relacionadas e atuantes nos estágios iniciais da CCDC. ^183 , 205^

 Aspectos adicionais relacionados com a complexa fisiopatologia disautonômica observada na DC envolvem a chamada via anti-inflamatória colinérgica. A base conceitual aqui envolvida reside em evidências de que o processo inflamatório instalado na DC influencia e é influenciado pelo equilíbrio autonômico mediado pelo sistema imunológico. ^237 - 239^ Assim, observou-se atenuação da citotoxicidade de linfócitos T pela estimulação colinérgico-muscarínica, postulando-se vias de sinais aferentes e eferentes que comporiam um arco, o reflexo “neuroimune” ou “inflamatório”. 

 De acordo com essa visão conceitual, os sistemas nervoso e imunológico comunicam-se de forma bidirecional usando essa interação como mediadora de citocinas e neurotransmissores comuns a ambos. A via eferente do sistema nervoso central atuaria no sistema imunológico através de seu componente parassimpático, compondo a chamada via anti-inflamatória colinérgica. O sistema parassimpático inerva os órgãos do sistema imunológico e seu mediador, a acetilcolina, atua sobre as células do mesmo, especialmente em macrófagos, por meio da ativação do receptor de acetilcolina. ^240^ No contexto da DC, levanta-se a hipótese de que a depressão do tônus parassimpático cardíaco poderia contribuir para exacerbar a inflamação durante a fase crônica, uma concepção fisiopatológica que remonta aos primórdios das investigações sobre a DC. ^229^

 Os mecanismos que induzem disfunção autonômica na DC incluem produção de autoanticorpos circulantes [particularmente contra receptores colinérgicos (Ac-M), bem como contra receptores adrenérgicos (Ac-β)]. ^240^ Postula-se que tais anticorpos sejam resultantes do mimetismo antigênico (reação cruzada entre a proteína ribossomal P do *T. cruzi* e a proteína ribossomal humana), ^241 - 243^ sendo plausível conceber que tais distúrbios mediados por autoanticorpos circulantes possam conferir características particulares à disautonomia da DC, entre as outras afecções neuronais. ^233 , 244 - 246^

## 4.4. Fisiopatologia da Doença de Chagas Dependente de Características Genéticas Parasitárias e do Hospedeiro Humano 

 O desenvolvimento de uma doença infecciosa é usualmente fenômeno complexo relacionado a vários fatores ambientais, do patógeno infectante e do hospedeiro. Assim, a avaliação das características genéticas do hospedeiro e do patógeno poderá contribuir decisivamente para que se decifre o “ *conundrum* ” de porque aproximadamente 30% dos indivíduos infectados desenvolvem a CCDC, enquanto o restante permanece assintomático e sem manifestações clínicas por toda a vida. 

 A diversidade genética do *T. cruzi* reconhece sete unidades discretas de tipagem (DTU), TcI-TcVI e Tcbat. ^247^ Essa diversidade genética constitui, em essência, alvo potencial de inovações a serem conseguidas com novos fármacos tripanocidas. ^248^

 Recentes pesquisas indicam que as cepas do parasita detectadas em pacientes, independentemente da apresentação clínica, refletem as principais DTU circulantes nos ciclos de transmissão doméstica de uma determinada região. Recente revisão sistemática e meta-análise de investigações *in vitro* evidenciou que, a despeito de existirem indícios preliminares de relevantes diferenças na sensibilidade do parasito ao tratamento etiológico, há considerável heterogeneidade de resultados, mesmo considerando-se apenas estudos relativos à sensibilidade das diversas DTU do *T. cruzi* a um único tripanocida, o benznidazol, impossibilitando a identificação precisa de cepas parasitárias mais e menos sensíveis ao tratamento. ^249^

 Em vários estudos de micro surtos com parasitos transmitidos oralmente, cepas silvestres estão implicadas. Como consequência das diferenças genotípicas e fenotípicas das cepas de *T. cruzi* e da distribuição geográfica diferencial das DTU em humanos, verificam-se variações regionais na sensibilidade dos testes sorológicos, acarretando potenciais implicações na resposta às opções de tratamento parasiticida. ^250^

 Tais características genotípicas foram recentemente sumarizadas para aclarar suas potenciais associações com manifestações clínicas da DC, ressaltando-se que persistem significativas incertezas de conhecimento e relevantes desafios nessas linhas de pesquisa. ^251^

 De forma similar, estudos de polimorfismo genético focalizam características do hospedeiro que influenciam no desenvolvimento e na gravidade das apresentações clínicas. Nesse contexto, os SNP são definidos quando pelo menos dois nucleotídeos alternativos ocorrem no genoma em frequências apreciáveis (geralmente > 1%). Os SNP exibem herança mendeliana e são usados como marcadores genéticos. ^252^

 Diversas pesquisas foram desenvolvidas avaliando o polimorfismo genético humano e incluindo correlações com elementos da resposta imune, adaptativa e de regulação, durante a infecção pelo *T. cruzi.* ^253^ O polimorfismo do TNF figura entre os mais estudados na DC. No Brasil, relatou-se redução de sobrevida de pacientes em que se encontrava o alelo TNF-308A ou do microssatélite TNFa2, ^254^ mas não se comprovou associação entre o polimorfismo do TNF-308 e as apresentações clínicas da DC. ^255^ De maneira análoga, outra investigação em pacientes peruanos, comparando aqueles com DC *versus* indivíduos controles sem infecção pelo *T. cruzi* , não evidenciou maior associação dos polimorfismo -308, -244 e -238 com a DC. ^256^

 Em contraposição ao descrito para mediadores relacionados ao perfil imunológico, avaliação genética relacionada ao sistema da enzima de conversão da angiotensina evidencia algumas discordâncias, mas o genótipo DD tem sido associado com maior risco de IC e mortalidade na doença miocárdica de etiologia isquêmica. ^257^

 Em outro estudo de coorte, na IC por cardiomiopatia idiopática, demonstrou-se que o genótipo DD mantinha-se como preditor de mortalidade. ^258^ Já em duas populações distintas com DC, incluindo uma brasileira, não foram observadas associações válidas quanto a esses polimorfismos. ^259 , 260^ No entanto, em outra população do nordeste brasileiro, relatou-se maior prevalência do polimorfismo I/D em pacientes com IC em comparação a pacientes com DC assintomáticos. ^261^ Essas discrepâncias podem dever-se a que fenótipos finais sejam vistos na dependência de fatores ambientais, ^262^ tornando necessárias grandes amostras para se demonstrar efeitos dos genes envolvidos em traços complexos, como as vigentes em síndromes clínicas complicadas, como a IC de etiologia da DC. 

 Estudos mais recentes focalizando aspectos genéticos e utilizando a tecnologia GWAS já envolvem amostras de grande amplitude e podem gerar informações mais consistentes e relevantes. Por exemplo, anteriormente, não foram os SNP altamente associados com a CCDC. ^170^ Mas recente meta-análise revelou a associação do genoma com o desenvolvimento de CCDC em posição rs2458298, próximo ao gene SAC3D1, e indigitou-se a variabilidade genética do hospedeiro como fator de suscetibilidade ao desenvolvimento da CCDC após a infecção pelo *T. cruzi* . ^171^

## 4.5. Histopatologia Peculiar da Doença de Chagas

 Na fase aguda da infecção, a adesão e a penetração do parasito nas células do hospedeiro ocorrem por meio de lecitinas que se ligam a resíduos de carboidratos existentes na membrana da célula hospedeira, principalmente o ácido siálico. Revisão recente sobre família de proteínas humanas ligantes de galactosídeos, denominadas galectinas, advoga por sua atuação significante na imunomodulação inata e adaptativa à infecção pelo *T. cruzi,* com potenciais implicações fisiopatológicas e terapêuticas. ^263^

 Formas tripomastigotas transformam-se no interior das células do hospedeiro em formas amastigotas, mas, enquanto as células parasitadas se mantêm íntegras, não se observa reação inflamatória local. Quando a célula parasitada se rompe, há liberação das formas epi, tripo e amastigotas do parasito, íntegras ou degeneradas, bem como de componentes celulares que atuam como imunógenos (por exemplo mitocôndrias, restos de miofibrilas) no meio extracelular, estimulando a presença de mediadores inflamatórios, que causam vasodilatação e aumento da permeabilidade vascular, fatores tipicamente implicados na exacerbação do processo inflamatório. 

 Na fase aguda inicial, a inflamação é focal e associada topograficamente ao parasitismo intenso, podendo confluir e tornar-se difusa. Em contraste, na fase crônica, a situação é mais obscura e complexa, pois embora se verifique reação inflamatória ativa, o parasitismo é escasso e não explica completamente os focos inflamatórios. Por isso, tem-se aventado a hipótese de hipersensibilidade tardia e de autoimunidade na manutenção da inflamação e das lesões na fase crônica da doença, explicada por: (1) moléculas do parasito e de miocardiócitos têm alguma semelhança estrutural, o que poderia explicar propriedades antigênicas comuns e reação imunitária cruzada: *in vitro* , linfócitos sensibilizados ao *T. cruzi* têm ação citotóxica contra miocardiócitos; (2) o infiltrado inflamatório mononuclear e a eventual formação de granulomas sugerem possível reação de hipersensibilidade tardia. 

 Esses aspectos mais controversos da fisiopatologia das lesões inflamatórias da fase crônica da cardiomiopatia da DC foram parcialmente esclarecidos por estudos recentes usando testes mais sensíveis para detecção do parasito. Esses testes sugerem que, mesmo escassa, a persistência parasitária nos tecidos é fonte contínua de antígenos, que podem mediar resposta inflamatória de baixo grau, mas virtualmente incessante. 

 Técnicas de biologia molecular, como a reação em cadeia da polimerase (PCR), aplicadas em fragmentos miocárdicos de pacientes com a CCDC, mostram DNA do *T. cruzi* nos focos inflamatórios em praticamente todos os casos estudados. Além disso, o acúmulo de linfócitos T CD8+, que predominam na miocardite crônica, correlaciona-se com a presença focal de antígenos parasitários. É possível que, além de tripanossomas degenerados, a ruptura celular promova liberação de microrganismos que estariam no citoplasma dos parasitas. Essa hipótese vem da observação de que biópsias endomiocárdicas de pacientes com CCDC evidenciaram micropartículas e nanovesículas elétron-lucentes contendo DNA de arqueias - microrganismos mais antigos da natureza, que podem parasitar tripanossomos - na região dos focos inflamatórios. ^264^

 Arqueias, numerosas no soro de pacientes com IC por DC, associam-se a inflamação, pois captam proteínas do interstício e geram resposta imune com linfócitos T CD8+, sem resposta de células T CD4+. Já arqueias lipídicas estão aumentadas na FIDC, assim como exossomos protetores que captam AMZ1 (metaloprotease específica de arqueia) do meio externo, impedindo a ativação das enzimas e protegendo contra a degradação do colágeno e a inflamação. Assim, por essa hipótese, arqueias poderiam ter papel fundamental no surgimento de inflamação miocárdica e dilatação da microcirculação. ^265^

 Ainda do ponto de vista da histopatologia da DC, pesquisas antigas evidenciaram que a infecção pelo *T. cruzi* tenha inclusive certo tropismo para o tecido adiposo e que esse fato possa constituir outro elo fisiopatológico da extensa alça de alterações inflamatórias presentes na fase crônica, eventualmente passível de exploração como alvo terapêutico. ^266 - 268^

## 4.6. Lesões da Microcirculação Coronária

 Diversas manifestações clínicas em pacientes com a CCDC mimetizam as que ocorrem em doentes afetados por doença obstrutiva coronária. Assim, constata-se que cerca de 30-40% deles exibem precordialgia, embora usualmente atípica por não guardar relação nítida com o esforço físico e ter duração muito variável, por longos períodos sintomáticos. O ECG desses pacientes, com frequência, exibe alterações de ST-T, além de áreas de inatividade elétrica, simulando alterações comumente devidas a isquemia e/ou infarto do miocárdio. Ainda mais caracteristicamente, os pacientes com CCDC com frequência apresentam alterações de mobilidade parietal ventricular semelhantes às que decorrem de necrose e infarto associado a obstruções coronárias. Finalmente, variados distúrbios perfusionais miocárdicos são descritos em pacientes em diversas fases da história natural da CCDC. 

 Todavia, todas essas alterações estruturais e funcionais são encontradas na presença de coronárias subepicárdicas angiograficamente normais e sem aterosclerose precocemente detectável por angiotomografia. ^269^ Em conjunto, essas alterações fisiopatológicas são atribuídas a anormalidades estruturais e de regulação coronária em nível microvascular. Histologicamente, descreve-se vasodilatação extrema, não vista em outras CMD, com redução da pressão de perfusão distal, miocitólise e isquemia em regiões limítrofes de dupla irrigação coronariana (zonas de “ *watershed* ” vascular, como, por exemplo, na região da *crux cordis* , em que artéria septal, ramo da artéria descendente anterior, compete com artéria originada da coronária direita), postuladas como mais suscetíveis a isquemia. ^270^ Admite-se que tais lesões isquêmicas possam contribuir para a instalação de áreas acinéticas e aneurismas ventriculares, como no adelgaçamento da ponta e na fibrose típica inferolateral frequentemente detectada como origem de taquicardia ventricular sustentada (TVS). 

 Consequência comum desses distúrbios microcirculatórios é a fibrose, que se desenvolve lenta e progressivamente, com deposição de fibronectina, laminina e colágeno no interstício, levando à expansão e distensão da matriz extracelular e contribuindo para perda progressiva da atividade contrátil do miocárdio e aparecimento de arritmias cardíacas. Não há outra miocardite humana em que a fibrose se desenvolva de forma tão intensa e com características tão peculiares como na CCDC. ^271^

## 4.7. Aplicações Terapêuticas Potenciais de Alvos Fisiopatológicos na Cardiomiopatia Crônica da Doença de Chagas 

 Diversas investigações recentes focalizaram variadas alterações fisiopatológicas, com potencial de constituir alvos terapêuticos para se influenciar favoravelmente a história natural da infecção pelo *T. cruzi* ou mesmo da CCDC. O próprio ciclo vital do parasito, mediante novos conhecimentos de sua interação com o hospedeiro humano, e o vetor como hospedeiro intermediário, com suas características genéticas melhor compreendidas, poderão ser revisitados com vistas a possibilidades terapêuticas de efeito tripanocida. ^209^

 Mas é sobre a possibilidade de modulação da resposta inflamatória que residem as perspectivas mais recentemente divisadas. Por exemplo, há demonstração de que, nas formas indeterminada e cardiomiopática da DC, existem mecanismos diversificados de ativação inflamatória da IL-1Beta. ^143^ Assim, após estudo pré-clínico, evidenciando redução de fibrose com inibidor do fator TGF-beta, ^157^ o antagonismo dessa citocina passa a representar um importante alvo terapêutico nesse contexto. ^272^

 Ademais, há evidências de que as formas clínicas da DC (indeterminada e cardiomiopática) envolvam diferentes subpopulações de células de memória imunológica CD4- e CD8- e abram a perspectiva de nova estratégia anti-inflamatória para controle da DC no coração. ^273^ Uma visão aprofundada sobre vários aspectos hipoteticamente ligados a múltiplas estratégias visando controlar o parasito e suas consequências inflamatórias para se melhorar o prognóstico de indivíduos infectados foi recentemente divulgada. ^274^

 Em outra linha de pesquisa sobre fármacos naturais dotados de potente atividade anti-inflamatória e antioxidante, como o curcumin e o resveratrol, resultados em animais de experimentação foram revistos e encorajam futuras iniciativas em humanos. ^275^ Estudo pioneiro randomizado em pequena amostra de 37 pacientes com CCDC reportou que a terapia com o fator estimulador de colônias de granulócitos (G-CSF), usada em aplicações clínicas como suporte para quimioterapia e transplante de medula óssea, visando controlar outros contextos de doenças, e também com resultados promissores em camundongos infectados pelo *T. cruzi* , apresenta boa tolerabilidade ao tratamento durante 1 ano, sugerindo a possibilidade de pesquisas mais extensas a serem realizadas com esse fator G-CSF em humanos com CCDC. ^276^

 Finalmente, o enfoque sobre o polimorfismo genético, que regula fisiopatologicamente níveis de fatores pró- e anti-inflamatórios (como exemplificado por IL-10), tem sido recentemente revisitado mediante meta-análise de vários estudos em diversas subpopulações de indivíduos infectados pelo *T. cruzi* , com a perspectiva de obter biomarcadores preditores de risco de desenvolvimento de CCDC e eventualmente servir para se monitorar a evolução e as intervenções terapêuticas nesse contexto. ^277^

## 5. História Natural

### 5.1. A Miocardite Aguda da Doença de Chagas

 A miocardite aguda da DC tem incidência variável decorrente da carga e cepa parasitária, do hospedeiro e da via de infecção (oral ou vetorial clássica, principalmente). Dependendo da ferramenta utilizada para o diagnóstico, a detecção de miocardite pode variar de 40% a 100% na fase aguda da infecção pelo *T. cruzi* . ^278 - 281^

 Conforme amplamente discutido no capítulo sobre a patogênese da DC, a anatomopatologia na fase aguda está diretamente relacionada ao parasitismo das células cardíacas, à reação inflamatória imediatamente suscitada pelo processo infeccioso e à perturbação microcirculatória consequente. ^282^ Há lesões inflamatórias no miocárdio, endocárdio, pericárdio e sistema nervoso autônomo intramural do coração e de vários outros órgãos, à semelhança do verificado em miocardites virais. Nas colorações com hematoxilina-eosina e Giemsa, podem-se evidenciar, com certa facilidade, formas amastigotas do parasito. ^283 , 284^

 De característico, podem-se encontrar pequenos nódulos enfileirados com aspecto de contas de rosário, aos quais se denomina epicardite moniliforme. Conquanto ocorra verdadeira pancardite, frequentemente há preservação das valvas cardíacas, estruturas tipicamente avasculares. As lesões cardíacas têm intensidade bastante influenciada pela via de infecção (oral ou vetorial clássica), podendo cursar, na maioria dos casos, de forma muito benigna, virtualmente oligossintomática, ou, ao contrário, muito grave, acarretando inclusive a morte do paciente. ^283 - 285^

 Os aspectos clínicos têm sido mais focados recentemente no que respeita à miocardite provocada pelo *T. cruzi* após transmissão oral (por ingestão de alimentos não preparados higienicamente e contaminados por barbeiros macerados ou seus dejetos junto com o material alimentar), verificando-se com muita frequência aspectos subclínicos. A inflamação aguda pode ter início pouco antes do desaparecimento da febre, o que ocorre em média cerca de 15 a 20 dias do início da doença. ^2^

 Alguns pacientes, à semelhança do que ocorre nas miocardites virais, podem manifestar sintomas de dor precordial, dispneia e palpitações, às vezes simulando quadros de doença arterial coronária. ^286^ Comumente, encontra-se taquicardia e, nos casos mais graves, sintomas e sinais de IC aguda em que alguns pacientes apresentam o perfil hemodinâmico C (má perfusão tissular e congestão pulmonar e/ou sistêmica). ^287^ No ECG, registram-se alterações inespecíficas da repolarização ventricular, complexos QRS de baixa voltagem, extrassístoles supra ou ventriculares, podendo inclusive ocorrer supradesnivelamento mantido do segmento ST. Os distúrbios de condução atrioventricular ou mesmo intraventricular, comuns na fase crônica, são menos frequentes na miocardite da fase aguda. ^279 , 288^

 O ECO detecta frequentemente derrame pericárdico de proporções variáveis, com hipocontratilidade difusa de ambos os ventrículos, sendo um apanágio dos casos de miocardite mais graves. ^289^

 A hipótese mais aceita para evolução fatal em porcentagem mais significativa de pacientes em que a transmissão foi por via oral deve-se a que um grande inóculo, com elevada carga parasitária ingerida, ocorreu, além de facilitada pela intensa penetração do parasita através da mucosa gastrointestinal, muito permeável ao *T. cruzi* . ^48 , 290^ Em estudo envolvendo 126 indivíduos com idade < 18 anos, nos quais a forma aguda foi diagnosticada em sua maioria (68,3%) após transmissão oral e seguidos por 10,9 anos, a evolução foi considerada benigna, embora 2,4% tivessem persistido com alterações cardíacas. ^291^

 A história natural da fase aguda da DC causada por transmissão vetorial clássica (dejetos do inseto hematófago) inclui elevada fração de indivíduos cuja infecção não é sequer diagnosticada por serem assintomáticos ou oligossintomáticos e que evoluem para remissão praticamente espontânea dos escassos sintomas. Em reduzida proporção dos casos, a infecção aguda pelo *T. cruzi* pode ser fatal (estimativa de 3-5% dos casos sintomáticos, por miocardite e/ou meningoencefalite fulminante). 

 A história natural da miocardite da fase aguda causada por transmissão vetorial clássica ^292 , 293^ é menos esclarecida do que a registrada em micro surtos recentemente verificados após transmissão oral. Entretanto, é patente que casos mais sintomáticos se associam a desfechos desfavoráveis pela óbvia razão de ocorrerem em condições de miocardite aguda mais intensa. ^294^ Todavia, de forma geral, a grande maioria dos indivíduos agudamente infectados pelo *T. cruzi* evoluem para a fase crônica e são caracterizados como tendo inicialmente a FIDC. 

 A história natural da miocardite aguda da DC e da FIDC ainda apresenta aspectos obscuros e controversos. Alguns estudos avaliaram de forma adequada essa evolução. Entretanto, diversas influências podem enviesar os resultados, tais como, faixa etária da população acometida, via de transmissão, carga e cepa parasitária, tempo de acompanhamento, além do tratamento etiológico pregresso, o que, aliás, tem o potencial de descaracterizar completamente - em sentido benéfico - a história natural. 

 Em estudo transversal realizado no município de Bambuí (MG), nas décadas de 1940-1950, a partir de fase aguda da DC diagnosticada após transmissão vetorial clássica, foi descrita 8,3% de letalidade na fase aguda de crianças < 10 anos. De 130 indivíduos acompanhados entre 1 e 3 anos após a fase aguda, 71,5% não apresentaram alterações no ECG e 30% tinham a área cardíaca normal. Após 3 a 5 anos, esses números foram, respectivamente, 65,7% e 87,5%. Deve-se lembrar que essa amostra populacional era basicamente composta de crianças em época pós-segunda guerra mundial e que não receberam tratamento etiológico. ^293 , 295 , 296^

 Em outro enfoque, mais recente, sobre história natural da DC, em dois grupos distintos de pacientes, o primeiro acompanhado desde o diagnóstico da fase aguda e o segundo, a partir da FIDC, avaliou-se o risco de desenvolvimento de cardiomiopatia crônica, por meio de revisão sistemática e meta-análise de 32 estudos. Considerou-se como diagnóstico de cardiomiopatia crônica o aparecimento de arritmias ou alterações no ECG, evidências de anormalidades na contração ventricular ao ECO ou mortalidade associada com a DC. Após a fase aguda, o risco estimado anual de evoluir para cardiopatia crônica foi elevado, de 4,6% (IC 95%, 2,7%-7,9%; I2 = 86,6%; τ2 [ *ln scale* ] = 0,4946). Já nos indivíduos acompanhados a partir da FIDC, esse risco foi de 1,9% (IC 95%, 1,3%-3,0%; I2 = 98,0%; τ2 [ *ln scale* ] = 0,9992). ^297^

 Estudos observacionais caracterizam a miocardite como potencial causa subdiagnosticada de IC aguda, que poderia evoluir para morte súbita, ou, mais tardia e comumente, como CMD. O prognóstico é considerado bom a curto prazo para o grupo de indivíduos que não expressam manifestações clínicas de acometimento cardíaco, ou quando há plena remissão da depressão miocárdica biventricular. Não há comprovação de quantos pacientes com miocardite clinicamente detectada na fase aguda recuperam a função ventricular, mas, a longo prazo, venham a apresentar CMD. Entretanto, observações bastante antigas relatam que o prognóstico de pacientes em que a fase aguda foi manifesta e a doença diagnosticada seja consideravelmente mais adverso do que quando a miocardite aguda passou sem percepção clínica. ^293 , 295 , 296^

 Há evidência por casos anedóticos e registros da OMS referindo que alguns indivíduos com miocardite aguda evoluem diretamente para a fase crônica com graves manifestações clínicas, sem passar pelo caracteristicamente longo período da FIDC. ^38^ Finalmente, como ocorre com outras etiologias potenciais de miocardite, explora-se o acesso atual a ferramentas diagnósticas que sejam de baixo custo, no sentido de prever o risco de eventos cardiovasculares e guiar a terapêutica. Nesse contexto, além da restrição logística de acessibilidade, permanece por se determinar o real papel da biópsia miocárdica e da RMC para abordagem de pacientes com suspeita de miocardite aguda de etiologia da DC. ^298^

### 5.2. A Forma Indeterminada e as Síndromes Clínicas da Cardiomiopatia Crônica da Doença de Chagas 

#### 5.2.1. História Natural da Fase Crônica da Doença de Chagas 

 Após a fase aguda, os indivíduos infectados pelo *T. cruzi* não tratados evoluem para a forma indeterminada da fase crônica, ou simplesmente FIDC. ^299 , 300^ Essa é classicamente definida pela evidência de infecção pelo *T. cruzi* , confirmada por exame sorológico ou parasitológico, na ausência de sintomas e sinais físicos da doença e de anormalidades no ECG em repouso e ao estudo radiológico do tórax, esôfago e cólon. ^301^ Pacientes classificados na FIDC têm excelente prognóstico de médio prazo (5 a 10 anos de seguimento), sendo as mortes entre eles muito raras e provavelmente não mais frequentes que as ocorrendo em grupos de indivíduos pareados por sexo e idade sem infecção pelo *T. cruzi* . ^299 , 300^

 Embora muitos indivíduos possam permanecer indefinidamente com a FIDC, observa-se em outros que, algumas décadas após a infecção aguda, a DC torna-se clinicamente evidente por acometimento específico de órgãos, principalmente coração, esôfago e cólon, caracterizando as formas clínicas crônicas determinadas: cardíaca, digestiva ou mista (cardiodigestiva). 

 Estudos epidemiológicos em áreas endêmicas, observações em doadores de sangue e resultado de meta-análise, após revisão sistemática, mostraram que cerca de 2% dos pacientes evoluem, a cada ano, a partir da FIDC para uma forma clínica da doença. ^297 , 302^ No Brasil, estima-se que cerca de 20% a 30% dos pacientes desenvolvem a forma cardíaca, de 5% a 8%, esofagopatia, e de 4% a 6%, colopatia. Com o envelhecimento da população, parcela maior dos infectados tende a evoluir para a forma cardíaca, embora o reconhecimento da real prevalência fique prejudicado pela coexistência de outras doenças cardiovasculares típicas da senescência. ^303^ Há significativas diferenças geográficas nas manifestações clínicas da DC em diversas regiões da América Latina e síndromes digestivas são menos comumente relatadas fora do Brasil. Do ponto de vista epidemiológico e clínico, a cardiomiopatia crônica é a forma mais importante da DC em decorrência de suas elevadas morbidade e mortalidade associadas e consequente impacto médico e social. 

#### 5.2.2. Forma Indeterminada da Doença de Chagas: Importância do Conceito e Alterações aos Exames Complementares Mais Sofisticados 

 O termo “forma indeterminada” foi utilizado, pela primeira vez, por Carlos Chagas, em 1916, para designar a infecção pelo *T. cruzi* na “ausência de qualquer das síndromes clínicas predominantes” da doença. ^229^ O seu potencial evolutivo foi descrito originalmente por Eurico Villela e Carlos Chagas em 1923 ^304^ e ressaltado, na década de 1950, por Laranja et al., ^305^ que definiram como FIDC o período assintomático de cerca de 10 a 30 anos entre o fim da fase aguda e o estabelecimento tardio da cardiopatia da infecção crônica. 

 Desde então, diversos autores utilizaram diferentes termos para se referir a esse estágio da doença, incluindo forma latente, assintomática, subclínica, laboratorial ou de “cardíacos potenciais”, sem padronização estrita dos critérios diagnósticos e levando a interpretações diferentes e até conflitantes sobre o real significado da FIDC. 

 Foi nesse contexto que um grupo de especialistas reunidos em Araxá, Minas Gerais, em 1984, elaborou um documento consensual reafirmando a validade do conceito da FIDC, bem como definindo os critérios diagnósticos objetivos citados acima. ^301^ O consenso ressaltou que a existência de alterações à propedêutica mais sofisticada não invalida o conceito acima exposto, reforçando o bom prognóstico dos casos em médio prazo, como confirmado pelo seguimento clínico e pelo ECG e ECO. ^211^

 Existem críticas e sugestões de modificação do conceito de FIDC, como a substituição da normalidade à radiografia de tórax pelo ECO normal para a definição da presença da FIDC ^306^ e até mesmo a abolição do termo, substituindo-o por “DC crônica sem patologia demonstrada”, quando não apenas o ECG convencional e a radiografia de tórax, mas também o ecodopplercardiograma, Holter e teste ergométrico, realizados de rotina, apresentassem resultados normais. ^11^ Entretanto, o conceito clássico de FIDC tem sido reafirmado em diretrizes nacionais e internacionais. ^2 , 7^

 Deve-se ressaltar não ser habitual na prática clínica e em estudos epidemiológicos que se avalie rotineiramente os pacientes com DC, ECG e radiografia de tórax normais e sem manifestações digestivas, por meio da propedêutica radiológica do trato gastrointestinal, o que tem levado ao conceito operacional de “DC crônica sem cardiopatia aparente”, visto que a definição clássica de FIDC requer a exploração radiológica de esôfago e cólon. ^299^

 À medida que os métodos de investigação utilizados se tornaram mais sofisticados, várias alterações, geralmente discretas e sem implicações prognósticas, puderam ser detectadas nesses indivíduos, como relatado em estudos com ecodopplercardiograma, ventriculografia radioisotópica, teste ergométrico, ergoespirometria, provas autonômicas e ECG dinâmico. ^204 , 299 , 307 , 308^ Além disso, métodos invasivos, como a biópsia endomiocárdica, mostraram alterações histológicas em pacientes com FIDC, em substancial porcentagem de casos, mas de baixa intensidade. Entre 33 pacientes com essa forma da doença submetidos à biópsia endomiocárdica, 60% mostraram alterações degenerativas, alteração no volume de fibras, edema intersticial, infiltrado inflamatório e fibrose em pequenas quantidades. ^309^

 Atualmente, a RMC fornece os mesmos dados, com a vantagem de ser método não invasivo. ^310 , 311^ Métodos ecocardiográficos mais sensíveis, como o Doppler tecidual ^312^ e a deformação ( *strain)* miocárdica longitudinal global (GLS) ^313^ medida com *speckle tracking echocardiography* (STE), também se mostraram alterados em pacientes na FIDC. Todavia, tais estudos ainda não tiveram seguimento suficiente para definir se os pacientes com tais alterações sutis evoluiriam de forma diferenciada e, eventualmente, com disfunção ventricular. 

#### 5.2.3. Evolução para Cardiomiopatia Crônica

 O risco de desenvolvimento de cardiomiopatia crônica tem sido avaliado em estudos de coorte nos últimos 60 anos, que foram congregados em revisão sistemática e meta-análise recente. ^297^ Os seguintes desfechos cardíacos primários foram considerados nessa revisão sistemática: (1) desenvolvimento de sintomas em geral ou de IC em específico; (2) desenvolvimento de cardiomiopatia estrutural ou arritmias cardíacas, conforme observado em resultados anormais por ECG ou ecocardiografia; e (3) presença de complicações decorrentes de cardiomiopatia grave, incluindo morte súbita, mortalidade associada a IC avançada, embolia pulmonar ou AVC. Vinte e três estudos apresentaram resultados observacionais longitudinais para pacientes com a FIDC. A maioria foi de coortes prospectivas e conduzidas no Brasil ou na Argentina entre 1960 e 2005. Nos estudos que incluíram dados de idade, as médias etárias variaram de 10 anos a 44 anos, com média geral de 31 anos. A duração média do acompanhamento foi de 8,5 anos (variação de 3 anos a 18 anos). O estudo concluiu que a taxa anual estimada combinada de desenvolvimento de CCDC foi de 1,9% (IC 95%, 1,3% - 3,0%). A probabilidade cumulativa do aparecimento de evidências de cardiomiopatia foi de aproximadamente 17% em 10 anos e 31% em 20 anos. ^297^

 Embora a taxa de evolução para cardiomiopatia seja assim estimada, ainda persistem muitas dúvidas sobre os mecanismos envolvidos na progressão da doença. Na mesma revisão sistemática citada acima, ^297^ os autores não encontraram diferenças quanto à taxa de evolução com base no ano das investigações (anteriores ou posteriores a 1985), no tamanho do estudo (> ou < 200 participantes), na idade média dos participantes (< ou > 32 anos) ou no sexo predominante. Entretanto, nos estudos originários do Brasil, os participantes tiveram uma taxa anual significativamente maior de desenvolvimento de cardiomiopatia (2,3%; IC 95%, 1,2% - 4,3%) em comparação com estudos de pacientes de outros países da América do Sul (1,1%; IC 95%, 0,5% - 2,4%; P = 0,05), mais uma vez ressaltando a importância de diferenças regionais no curso da doença. 

 De importância clínica, os autores relataram que o subgrupo de participantes que recebeu tratamento antiparasitário teve uma estimativa de taxa anual combinada significativamente menor de desenvolvimento de cardiomiopatia (1,0%; IC de 95%, 0,5% - 1,9%) em comparação com o subgrupo que não recebeu tratamento etiológico (2,3%; IC de 95%, 1,5 % - 3,5%; P = 0,03). ^297^

 Tais resultados são coerentes com a noção fisiopatológica geral de que existam, na verdade, evidências substanciais de que a persistência (carga) do parasita seja fator primordial para a progressão da FIDC para a CCDC. Em estudo referencial, ^314^ mostrou-se, em modelo murino de infecção pelo *T. cruzi* , que a persistência do parasita se correlaciona com a presença de doença cardíaca e que a eliminação dos parasitas dos tecidos foi associada à melhora da inflamação. 

 Estudos subsequentes demonstraram que a extensão da inflamação e da fibrose e a gravidade da doença estavam associadas à persistência do DNA do parasita em lesões cardíacas observadas em pacientes com a DC. ^315 , 316^ A presença de parasitemia correlaciona-se significativamente com marcadores conhecidos de progressão da doença, como prolongamento do QRS, FEVE reduzida e níveis mais elevados de troponina e da porção N-terminal do pró-hormônio do peptídeo natriurético do tipo B (NT-proBNP). ^317^

 Em coorte de 1.813 pacientes com CCDC, aqueles previamente tratados com benznidazol apresentaram parasitemia significativamente reduzida, menor prevalência de marcadores de cardiomiopatia grave e menor mortalidade após 2 anos de acompanhamento. ^318^ Resultados adicionais da coorte NIH-REDS2, com seguimento médio de 8,7 anos da coorte original, ^319^ mostraram que a incidência de cardiomiopatia em doadores de sangue soropositivos para *T. cruzi* foi de 13,8 (IC 95% 9,5-19,6) eventos/1000 aa (32/262, 12%) em comparação com 4,6 (IC 95% 2,3-8,3) eventos/1000 aa (11/277, 5%) em controles soronegativos, com uma diferença de incidência absoluta associada à infecção por *T. cruzi* de 9,2 (IC 95% 3,6-15,0) eventos/1000 aa. O nível de anticorpos anti- *T. cruzi* no início do estudo, uma medida indireta da carga parasitária, foi associado ao desenvolvimento de cardiomiopatia, com razão de chances ajustada de 1,4 (IC 95% 1,1-1,8) por unidade de aumento no nível de anticorpo. ^319^

 A importância da persistência do parasita no desenvolvimento da CCDC também é corroborada por extenso ensaio clínico não randomizado relatado por Viotti *et al* ., ^320^ mostrando que o tratamento com benznidazol, em comparação com ausência de tratamento etiológico, foi associado à redução da progressão da DC e aumento da soroconversão negativa. Outros estudos observacionais mostraram resultados semelhantes. ^321 - 323^

 Por outro lado, existem evidências de que, uma vez estabelecida a cardiopatia, o parasitismo tecidual possa ter menor importância no curso clínico da doença, predominando os danos imunológicos aos tecidos. De acordo com essa possibilidade hipotética, uma vez estabelecida a cardiopatia, ao se eliminar o fator parasitário tissular, não haveria mais chance de reversão em benefício de história natural menos ominosa, porquanto lesões irreversíveis já estariam instaladas. Assim, o estudo BENEFIT, prospectivo, multicêntrico e randomizado, envolvendo 2.854 pacientes com CCDC que receberam benznidazol ou placebo por até 80 dias e foram acompanhados por uma média de 5,4 anos, mostrou que o uso do tripanomicida reduziu a parasitemia nos pacientes tratados, mas não influenciou significativamente a deterioração clínica cardíaca em relação ao grupo controle. ^324^ Tais resultados têm sido objeto de discussões e interpretações distintas e complementares ^325^ e a questão da importância da persistência do parasita nos pacientes com cardiopatia estabelecida continua controversa, conforme exposto em detalhes no capítulo de tratamento etiológico desta diretriz. 

#### 5.2.4. Formas Clínicas da Cardiomiopatia Crônica da Doença de Chagas 

 A CCDC apresenta história natural caracteristicamente lenta e progressiva, embora ocasionalmente possa ter evolução mais abrupta. Suas manifestações clínicas variam desde quadros assintomáticos (cardiopatia “silenciosa’’) até apresentações graves, com IC refratária, distúrbios do ritmo e fenômenos tromboembólicos, as três síndromes clínicas principais. ^326^ Os sintomas mais importantes são: dispneia aos esforços, fadiga, palpitações, tontura, síncope, dor torácica (angina, usualmente atípica) e edema de membros inferiores. 

 O exame físico geralmente demonstra uma ou mais alterações: sopro sistólico de regurgitação mitral e/ou tricúspide; desdobramento da segunda bulha cardíaca, geralmente associado a bloqueio de ramo direito (BRD); impulso apical difuso e deslocado no tórax; e arritmia, sendo as extrassístoles a forma mais comum. 

***5.2.4.1. Alterações em Exames Subsidiários***

 O ECG na DC tem fundamental valor diagnóstico e prognóstico. ^327 , 328^ A ausência de alterações eletrocardiográficas, todavia, não é indicador fidedigno absoluto da ausência de acometimento cardíaco. ^299^ O BRD é a anormalidade eletrocardiográfica mais comum, isoladamente ou em associação com outras alterações. ^329 , 330^ É mais tipicamente associado com bloqueio divisional anterossuperior esquerdo (BDASE) e extrassístoles ventriculares (EV). A duração do QRS está diretamente relacionada ao tamanho do VE e inversamente relacionada com a FEVE. ^331^ A duração do QRS > 120ms e o intervalo QT > 440ms têm acurácia moderada em predizer FEVE reduzida em pacientes com DC. ^332^

 As anormalidades no ECG mais frequentemente associadas à redução da FEVE na DC são as extrassístoles supraventriculares e ventriculares frequentes, FA, bloqueios intraventriculares, ondas Q patológicas e alterações de ST-T. ^332 , 333^ A combinação de distúrbios de condução intraventricular com extrassístoles ou com bradicardia sinusal associa-se tanto à redução da FEVE quanto ao aumento do volume do VE. ^334^ Deve-se ainda reconhecer que alterações eletrocardiográficas causadas pela DC tendem, em indivíduos mais longevos, a somar-se àquelas ocasionadas pelo próprio processo inerente ao envelhecimento biológico. ^303^ Apresentação mais detalhada das alterações de ECG que configuram CCDC e das que não são consideradas suficientes para firmar esse diagnóstico encontra-se em outros capítulos desta diretriz. 

 A radiografia de tórax é importante exame complementar no diagnóstico dos pacientes com CCDC, possibilitando não somente avaliar-se o aumento das câmaras cardíacas como, em especial, o grau de congestão pulmonar, alteração não perceptível pela ecocardiografia habitual. ^335 , 336^ Há baixa correlação entre o aumento da silhueta cardíaca à radiografia de tórax e o grau de disfunção ventricular sistólica. ^335^ Por outro lado, a cardiomegalia detectada por índice cardiotorácico (ICT) > 0.5 à radiografia tem melhor correlação com o aumento do diâmetro diastólico do VE (DDVE) e sugere a presença de disfunção ventricular esquerda sistólica. ^337^

 De forma geral, na CCDC, as alterações radiológicas são semelhantes às detectadas em outras CMD. Porém, uma particularidade interessante refere-se a um fato conspícuo descrito por clínicos há várias décadas: em muitos pacientes com evidente congestão sistêmica, incluindo ascite, hepatomegalia e anasarca, há nítida desproporção entre o grau avançado de cardiomegalia e a pouco intensa congestão pulmonar. ^336^

 O ECO transtorácico tornou-se, há décadas, importante instrumento no diagnóstico e acompanhamento dos pacientes com DC em suas diversas formas. ^338 , 339^ O ECO é o exame não invasivo mais utilizado na avaliação da função cardíaca por ser altamente disponível e confiável em sua obtenção e interpretação, além de ter custo relativamente baixo. O ECO permite determinar o estado evolutivo e as alterações mais sutis do comprometimento cardíaco, especialmente em fases menos avançadas da cardiomiopatia. Particularidade muito expressiva nessa cardiomiopatia, verifica-se que até 13% dos pacientes com CCDC no estágio B (ver gradação da IC mais adiante) apresentam característico déficit segmentar, apesar de função sistólica biventricular global preservada. ^340^ É relevante observar que tais alterações isoladas de mobilidade segmentar do VE evidenciam nítida conotação de mau prognóstico, como verificado em estudos seriados com ecocardiografia. ^341 , 342^

 Dentre os vários parâmetros analisados, os mais importantes são: FEVE, diâmetro do átrio esquerdo, volume do átrio esquerdo, diâmetros sistólico e diastólico do VE, função diastólica, função sistólica do ventrículo direito (VD), contratilidade global e segmentar do VE, contratilidade global do VD e presença de aneurisma vorticilar ou de ponta do VE. 

 O estudo ecocardiográfico do VD é de mais difícil realização por óbices técnicos inerentes tanto à própria câmara ventricular como à essência do método ultrassonográfico. Em parte, por isso, há percepção de que a disfunção do VD seja mais evidente quando há envolvimento concomitante e significativo do VE. ^343 , 344^ A despeito dessa noção fisiopatológica, há evidência derivada de estudos empregando outros métodos - como a ventriculografia radionuclear, a RMC e a própria ecocardiografia mais especializada - de que alguns pacientes com a CCDC apresentam precocemente importantes alterações morfofuncionais isoladas do VD. ^203 , 345 - 348^ Nessas condições, na ausência de concomitante envolvimento patológico do VE e enquanto a impedância do circuito pulmonar se mantiver reduzida, a disfunção da câmara ventricular direita deve passar sem repercussão perceptível, uma vez que a *vis-a-tergo* ventricular esquerda é suficiente para a manutenção de fluxo e de resistência vascular pulmonar normais, conforme aventado em publicação seminal sobre o tema. ^349^

 Finalmente, quando aparece na história natural da doença, a disfunção sistólica ventricular direita clinicamente manifesta agrega significativo fator negativo ao prognóstico de pacientes com a CCDC. ^350^

***5.2.4.2. Arritmias Cardíacas***

 Arritmia cardíaca é manifestação extremamente comum na CCDC, sendo a atividade ectópica ventricular a predominante desde as fases iniciais de sua história natural. Assim, globalmente se constata que 15% a 55% dos indivíduos com sorologia positiva para o *T. cruzi* apresentam EV. Quando tais pacientes com alterações no ECG em repouso e IC manifesta são estudados através da eletrocardiografia dinâmica, praticamente todos (99%) apresentam EV, sendo que, em 87% deles, elas são multiformes ou se apresentam como formas repetitivas (pareadas) ou mesmo como taquicardia ventricular não sustentada (TVNS), ou seja, três ou mais ectopias ventriculares sucessivas, com duração inferior a 30 segundos. ^351^

 O acometimento do nó sinusal e do sistema de condução atrioventricular também é muito frequente nos pacientes com CCDC. A disfunção do nó sinusal pode se manifestar como bradicardia ou mesmo parada sinusal, bloqueio sinoatrial de segundo grau, ritmo juncional e ritmo idioventricular acelerado. O bloqueio atrioventricular (BAV) de 1° grau constitui um dos distúrbios de condução atrioventricular mais encontrados, podendo ser transitório ou fixo. O BAV de 2° grau é menos frequente, podendo ser do tipo Mobitz I (Wenckebach), Mobitz II ou de grau avançado. O BAV de 3° grau ou total (BAVT) pode ocorrer em 10% dos pacientes, sendo mais frequente do que em qualquer outra cardiopatia adquirida. Fibrilação atrial tende a ser manifestação mais tardia, geralmente associada a graus mais avançados de disfunção sistólica e dilatação ventricular. 

 As arritmias podem ser assintomáticas ou causar palpitações, tonturas, dispneia, fraqueza, pré-síncope, síncope ou parada cardíaca. A morte súbita é responsável por 50% a 65% dos óbitos por DC. ^352^ A morte súbita costuma ser precipitada por exercícios físicos e pode ser associada a TVS ou fibrilação ventricular (FV) e, menos frequentemente, assistolia ou BAVT. Cerca de 40% a 50% dos casos de morte súbita são assintomáticos antes do episódio fatal, porém, na maioria dos pacientes, há concomitância de comprometimento grave da função sistólica ventricular e do sistema de condução. A gravidade das arritmias ventriculares tende a se correlacionar com o grau de disfunção ventricular. Entretanto, diversamente do que ocorre em outras doenças, não é incomum que pacientes com CCDC e arritmias ventriculares malignas apresentem função ventricular esquerda global relativamente preservada (mas, muitas vezes, com discinesias regionais indicativas de fibrose localizada). ^353^ Episódios de arritmias ventriculares malignas são muito mais frequentes em pacientes com CCDC do que naqueles com outras formas de cardiopatia (como a decorrente de doença coronária ou CMD de outras etiologias). ^354 - 356^

***5.2.4.3. Síndrome de Insuficiência Cardíaca***

 Essa manifestação também aparece em muitos pacientes durante a história natural da CCDC, usualmente com evidências de disfunção biventricular, incluindo sintomas precoces como dispneia, fatigabilidade, edema de membros inferiores e dor torácica atípica. A disfunção diastólica pode ser observada precocemente na história natural da CCDC, na ausência de disfunção sistólica regional ou global do VE, e pode ser explicada por certo grau de fibrose difusa dessa câmara. ^357^

 Conforme apontado acima, em alguns pacientes, a IC direita pode ser mais proeminente do que a IC esquerda, mas a disfunção do VD, quando clinicamente manifesta, em geral está associada à disfunção ventricular esquerda em estágio avançado da CCDC. ^343 , 349^

 Uma classificação para IC de etiologia da DC, considerando-se a presença ou não de defeitos funcionais e/ou estruturais em geral e a função sistólica ventricular esquerda, em especial, mostra-se útil quando aplicada à CCDC, após discretas modificações a partir das diretrizes de 2011 da SBC, permitindo a identificação de subgrupos ou estágios evolutivos distintos do ponto de vista prognóstico e terapêutico. ^1^ A classificação da IC de acordo com a FEVE é mostrada na Tabela 5.1 . A classificação em estágios evolutivos aparece a seguir, na Tabela 5.2 . 

**Tabela 5.1:** – Cardiomiopatia crônica da doença de Chagas: função sistólica normal e classificação da insuficiência cardíaca de acordo com a fração de ejeção ventricular esquerda (FEVE).

CATEGORIA	CRITÉRIO
Função ventricular sistólica global normal	• FEVE ≥ 55% - sem disfunção segmentar - com disfunção segmentar
Insuficiência cardíaca com fração de ejeção levemente reduzida (ICFElr)	• FEVE entre 41% e 54%
Insuficiência cardíaca com fração de ejeção reduzida (ICFEr)	• FEVE ≤ 40%
Insuficiência cardíaca com fração de ejeção melhorada (ICFEm)	• FEVE prévia < 40% com aumento mínimo de 10 pontos percentuais, atingindo valores acima de 40%

**Tabela 5.2:** – Classificação da doença de Chagas crônica em estágios evolutivos.

	FORMA INDETERMINADA	CARDIOMIOPATIA CRÔNICA DA DOENÇA DE CHAGAS			
	Estágio A	Estágio B1	Estágio B2	Estágio C	Estágio D
**Características**	Assintomático; Sem doença estrutural cardíaca e digestiva (ECG e estudo radiológico); Risco de desenvolver CCDC (30%)	Doença estrutural cardíaca; Função ventricular sistólica global normal; Sem sintomas de IC	Doença estrutural cardíaca; Disfunção ventricular sistólica global; Sem sintomas de IC	Doença estrutural cardíaca; Disfunção ventricular sistólica global; Sintomas prévios ou atuais de IC	Doença estrutural cardíaca; Disfunção ventricular sistólica global; Sintomas de IC em repouso, refratários ao tratamento clínico otimizado
**ECG**	Normal	Alterado	Alterado	Alterado	Alterado
**Disfunção ventricular segmentar**	Geralmente ausente	Pode estar presente	Pode estar presente	Pode estar presente	Pode estar presente
**FEVE (Eco – Simpson)**	≥ 55%	≥ 55%	< 55% (geralmente entre 41% e 54%)	< 55% (geralmente ≤ 40%)	Geralmente ≤ 25%
**Classe funcional (NYHA)**	Não aplicável	I	I	I, II, III ou IV	IV
**Cardiomegalia (Rx tórax)**	Ausente	Ausente	Pode estar presente	Geralmente presente	Presente
Arritmia ventricular complexa* (Holter 24h)	Geralmenete ausente	Pode estar presente	Geralmente presente	Presente	Presente
**Fibrose miocárdica (realce tardio à RMC)**	Pode estar presente	Geralmente presente	Geralmente presente	Presente	Presente

* pares/salvas de extrassistoles ventriculares. CDCC: cardiomiopatia crônica da doença de Chagas; ECG: eletrocardiograma; FEVE: fração de ejeção ventricular esquerda; Insuficiência cardíaca; NYHA: New York Heart Association; RMC: ressonância magnética cardíaca.

***5.2.4.4. Síndrome Tromboembólica Sistêmica e Pulmonar***

 Essa síndrome também é bastante comum na CCDC e fenômenos tromboembólicos venosos e arteriais constituem a terceira causa de morte. ^352 , 358^ Do ponto de vista clínico, predominam os fenômenos tromboembólicos que atingem o cérebro, seguidos por embolia para outros órgãos sistêmicos e membros e por embolia pulmonar diagnosticada em vida. O AVC pode ser a primeira e devastadora manifestação da doença. 

 Para a síndrome tromboembólica ser tão frequente na CCDC concorrem diversos fatores que podem ser variavelmente predominantes de acordo com a fase da história natural da doença. Assim, o aneurisma apical emboligênico pode ser alteração precoce na CCDC, mas, muito mais comumente, tromboses em veias sistêmicas com potencial de causar embolia pulmonar são complicações da IC, quando o débito cardíaco e o retorno venoso estão prejudicados. De forma análoga, nessa condição de IC, a dilatação de câmaras favorece a trombose parietal em átrios e ventrículos, provocando embolias sistêmicas e/ou pulmonares. A FA também é mais frequente em casos avançados de CCDC e concorre para aumentar o risco de complicações tromboembólicas. 

 A DC é uma das principais causas de AVC na América Latina, com essa etiologia representando até 20% dessa complicação em áreas endêmicas. ^358 - 360^ A incidência de AVC em pacientes com DC conhecida varia de 0,56 a 2,67 por 100 pessoas-ano. ^361^ A CCDC deve, portanto, ser regularmente incluída no diagnóstico diferencial do AVC na América Latina. ^362^ Disfunção sistólica ventricular, aumento do volume do átrio esquerdo, aneurisma apical, trombose cavitária mural e arritmias, como a FA, parecem ser importantes fatores de risco na gênese do AVC de etiologia da DC, caracteristicamente de natureza cardioembólica. ^363^ De fato, em 50-70% dos pacientes, o AVC se manifesta com síndrome de circulação anterior parcial, que inclui dois dos três sinais: déficit motor ou sensorial envolvendo face, braço e perna; hemianopsia homônima; e disfunção cerebral superior, expressa por afasia ou déficit visuoespacial. Com menos frequência, os pacientes apresentarão uma síndrome lacunar ou de circulação posterior. 

 Um escore de risco ( *IPEC-FIOCRUZ)* para AVC foi desenvolvido em estudo observacional prospectivo de 1.043 pacientes. ^364^ Conforme discutido em capítulo específico desta diretriz sobre complicações tromboembólicas na DC, há presentemente necessidade de o escore ser revisitado para atender a considerações científicas mais atualizadas. 

## 6. Diagnóstico da Cardiomiopatia da Doença de Chagas 

### 6.1. Métodos para Evidenciar a Infecção pelo *T. cruzi*

#### 6.1.1. Introdução

 O diagnóstico de uma doença infecciosa deve ser apoiado por dados clínicos, epidemiológicos e laboratoriais. Esses três elementos devem ser considerados para confirmar o diagnóstico ou excluí-lo. ^365^

 Alguns dados clínicos podem ser considerados muito sugestivos de CCDC, como o BRD no ECG. ^366^ Mas nenhuma anormalidade eletrocardiográfica é específica da CCDC, tampouco ocorre em todos os pacientes com a doença. Os dados epidemiológicos, em particular a procedência do paciente de áreas reconhecidamente endêmicas, também auxiliam no diagnóstico. Assim, outro dado que deve ser valorizado relaciona-se aos antecedentes familiares, presentes em dois terços dos pacientes de área endêmica, em particular mãe ou irmãos infectados ou com história de morte súbita. ^367^

 O laboratório pode detectar o parasito ou, mais comumente, os anticorpos anti- *T. cruzi.* Na fase crônica da DC a maioria dos pacientes apresenta baixa parasitemia e os parasitos não são encontrados no exame de sangue. Portanto, com base nessa informação, não se deve excluir a etiologia pela ausência do protozoário. Ao contrário, praticamente todos os infectados crônicos apresentam anticorpos anti- *T. cruzi* em níveis/concentrações variáveis. Pelo exposto, o clínico que deseja confirmar ou excluir a etiologia *tripanossomótica cruzi* em paciente com cardiopatia deve solicitar, inicialmente, os exames sorológicos. 

#### 6.1.2. Exames Sorológicos Disponíveis e Testes a Solicitar 

 Os exames sorológicos disponíveis podem ser divididos em testes convencionais e não convencionais. Cada laboratório utiliza diferentes testes, tais como imunofluorescência indireta (IFI), hemaglutinação indireta (HAI), ensaio imunoenzimático (ELISA), dentre os convencionais, e, nos últimos anos, testes não convencionais, como quimioluminescência magnética (CMIA) e eletroquimioluminescência (ECLIA) em plataforma automatizada, assim como testes rápidos. Todos esses testes podem utilizar, como antígenos, produtos não purificados ou purificados (recombinantes, sintéticos e outros). ^365^ A OMS recomenda solicitar dois testes de princípios diferentes para o diagnóstico de DC, que podem ser convencionais ou não convencionais. ^2 , 38 , 60 , 368^

#### 6.1.3. Interpretação dos Resultados

**Figura 6.1:**
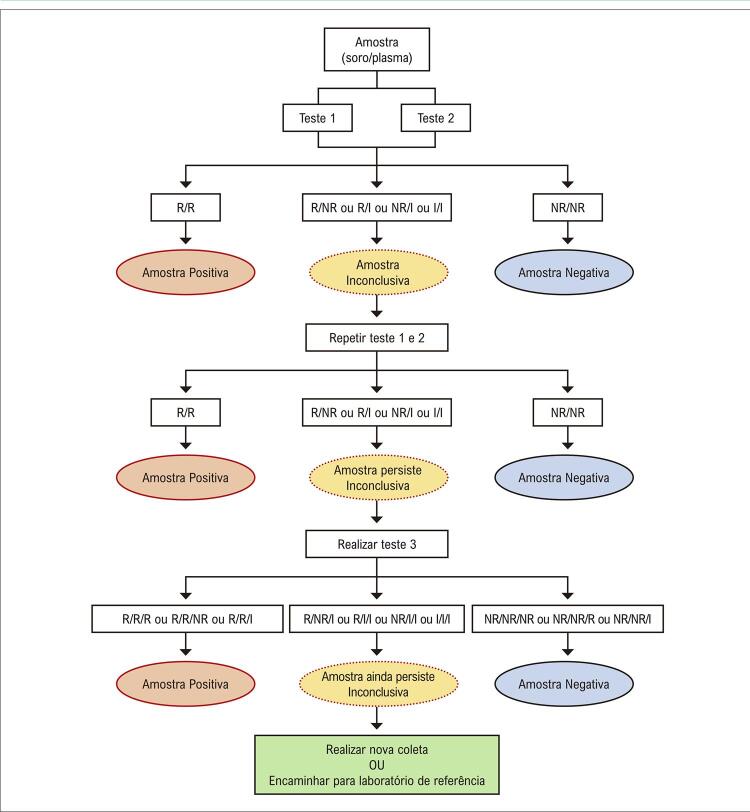
– Fuxograma do diagnóstico sorológico da doença de Chagas. R: reagente; NR: não reagente; I: indeterminada. Observação: Teste 1: IFI ou HAI ou ELISA ou Quimioluminescência ou Eletroquimioluminescência; Teste 2: Teste diferente do Teste 1; Teste 3: de princípio diferente do Teste 1 e do Teste 2.

 A combinação do resultado dos dois testes permite classificar o soro do paciente como positivo (dois testes reagentes) ou negativo (dois testes não reagentes). Trata-se de resultados concordantes entre os dois testes realizados. ^369^ Dois testes positivos (reagentes) indicam que o paciente é soropositivo, ou seja, que o paciente apresenta anticorpos anti- *T. cruzi* por duas metodologias diferentes, o que significa que é infectado pelo *T. cruzi* . Quando o resultado do exame é não reagente (concordante por dois testes de princípios diferentes), a sorologia é negativa; nesses casos, em geral não há antecedentes epidemiológicos e as alterações clínicas, se existentes, podem ser explicadas por outras causas, diferentes da infecção pelo *T. cruzi* . Em uma terceira possibilidade, que não é habitual (< 5% dos casos), o resultado não é concordante, ou seja, um teste fornece resultado reagente e o outro teste resultado não reagente ( Figura 6.1 ). 

 Finalmente, existe ainda a possibilidade de que um dos testes apresente resultado indeterminado, ou seja, situe-se numa faixa estreita entre o considerado negativo e positivo. Trata-se de resultado na região chamada “cinza”, observado, por exemplo, na transferência passiva de anticorpos maternos de mãe infectada para seu filho. A queda progressiva da concentração de anticorpos maternos no recém-nascido não infectado, em torno do 3º mês de idade, pode corresponder a essa região cinza, com resultado indeterminado. ^369^

 Nessas raras eventualidades de discordância, o médico, após avaliar os dados epidemiológicos e clínicos, pode adotar as seguintes atitudes: avaliar se o paciente foi submetido a tratamento específico anteriormente; e verificar se houve antecedentes de leishmaniose tegumentar ou de outras doenças, em particular, as autoimunes. Nesses casos, deve-se solicitar nova coleta de sangue. Com frequência, o resultado discordante torna-se concordante na nova amostra. Se o resultado indeterminado persistir, deve-se encaminhar o paciente para um serviço/laboratório especializado, onde outras técnicas serão realizadas, até se chegar a uma conclusão final. Na excepcional eventualidade de que mesmo o laboratório de referência não consiga precisar se o indivíduo é infectado ou não, pode-se recorrer a exames parasitológicos (vide abaixo). Nesses casos deve-se fazer avaliação clínica com ECG. No entanto, ainda que tenha um EGC normal, o paciente com sorologia inconclusiva deverá ser orientado a não doar sangue. 

#### 6.1.4. Situações Especiais

***6.1.4.1. Resultados Sorológicos Inconclusivos***

 Como já apontado, resultados sorológicos inconclusivos não são habituais (< 5%) e frequentemente estão associados à presença de outras doenças, em particular leishmaniose visceral ou tegumentar, lúpus eritematoso disseminado, hepatopatias crônicas, em geral com aumento de gamaglobulina. São as chamadas reações cruzadas. Assim, devem-se investigar outras causas e questionar se o paciente recebeu tratamento com benznidazol no passado. Caso isso se comprove, poderia indicar que a concentração de anticorpos do indivíduo diminuiu como consequência do tratamento e o resultado da sorologia tornou-se indeterminado. 

***6.1.4.2. Resultado Laboratorial Não Corresponde ao Esperado Clinicamente***

 Como já referido, devem ser solicitados dois testes sorológicos de princípios diferentes, de preferência incluindo os títulos obtidos, indicando a concentração de anticorpos. Quase sempre ambos os resultados são positivos ou negativos. Raras vezes os resultados dos dois testes empregados são discordantes e podem se apresentar em algumas combinações: um negativo e outro positivo ou um positivo e outro indeterminado. Nessas situações, deve-se solicitar nova coleta de sangue, empregando as mesmas técnicas e, se possível, uma terceira técnica [por ex. se ELISA for reagente e IFI não reagente, solicitar HAI ou quimioluminescência (CLIA), ou ELISA com outros antígenos]. Em geral, com esse procedimento, é possível obter um resultado conclusivo. 

 Interferências de diversas origens podem dar lugar a um resultado falso reagente que não se confirma pelos outros dois testes, negativos. Em outros casos, um teste pode ser não reagente e o mesmo soro reagente pelos outros dois testes. Os dados clínicos e os antecedentes epidemiológicos em geral permitem chegar ao diagnóstico. Em outras circunstâncias, os dados clínicos e epidemiológicos apontam para infecção por *T. cruzi* , porém os testes sorológicos solicitados são negativos. Pode-se recorrer a um laboratório de referência (ou serviço especializado) para nova coleta de sangue e execução de outros testes. Na experiência desses laboratórios, quando o resultado é totalmente negativo em três testes de princípios diferentes, em geral não se trata de infecção pelo *T. cruzi* . Assim, existem casos de BRD por outras causas, sendo relativamente frequente encontrar famílias em que alguns dos membros não são infectados pelo *T. cruzi* , o que leva à hipótese de resistência natural à doença, reconhecida em outras infecções como hanseníase e tuberculose. Também pode se tratar de um caso de cura espontânea, raro, porém possível. Há relatos na literatura de casos excepcionais de infecção pelo *T. cruzi* sem a presença de anticorpos no soro dos pacientes. ^370^ Se houver essa suspeita, devem ser solicitados testes parasitológicos para esclarecer a dúvida. 

***6.1.4.3. Parasitemia***

 Embora a maioria dos pacientes crônicos apresente baixa carga parasitária no sangue periférico, cerca de 20% deles podem apresentar elevada parasitemia, detectada por testes de multiplicação (hemocultura, PCR) seriados. Nos casos de RDC por imunossupressão (HIV e outros), a maioria dos pacientes vai apresentar elevada parasitemia. Deve-se lembrar que “reativação” significa que o indivíduo, do ponto de vista laboratorial, está na fase aguda, que é definida pela presença de parasitos no sangue periférico por exame direto, só observável, no contexto da história natural da infecção, em curto período da fase aguda inicial e durante a própria reativação a partir de fase crônica. Ressalte-se que a definição laboratorial de fase aguda é dada pela verificação de parasitos viáveis no sangue periférico. 

***6.1.4.4. Sorologia Negativa em Pacientes na Fase Crônica***

 Embora possível, é excepcional e foi observada em pacientes na Bolívia. ^370^

***6.1.4.5. Cura Espontânea***

 A cura espontânea da DC foi relatada por Zeledón *et al* ., ^371^ após coletar sangue de pacientes infectados na América Central anos após a respectiva fase aguda, que foi devidamente registrada com testes parasitológicos diretos positivos, durante a época em que não existia tratamento específico. A raridade desse fenômeno foi comprovada quando, posteriormente, relatou-se em estudo com 110 indivíduos na fase crônica da doença, seguidos por mais de 10 anos, que nenhum deles apresentou titulação posterior menor do que a inicial. ^372^ Ainda assim, tem-se observado esse fenômeno com ocorrência < 1%, ou seja, é possível, porém muito raro; habitualmente, a segunda coleta de amostra sempre apresenta algum nível de anticorpos por algumas das técnicas empregadas, isso é, não há negativação total. Se houver, outras hipóteses devem ser avaliadas, dentre as quais a mais provável se deve às diferenças entre testes de procedências distintas. 

***6.1.4.6. Diagnóstico de Fase Aguda***

 Excepcional no Brasil nos dias de hoje, é praticamente limitada a casos de transmissão pela via oral, em particular na região amazônica (aproximadamente 350 casos por ano), por meio de alimentos contaminados com triatomíneos infectados ou por suas fezes. A transmissão oral representa atualmente a principal causa da doença aguda em vários países sul-americanos. ^114^

 Em contexto geral, a DC aguda pode ser causada por triatomíneos (transmissão vetorial e oral), transmissão transfusional ou transplante de órgãos sólidos, vertical ou congênita e por acidente de laboratório. A RDC em indivíduo imunossuprimido natural ou iatrogenicamente também é considerada como fase aguda. Nesses casos, o diagnóstico laboratorial é realizado pela pesquisa direta do parasito com utilização dos métodos parasitológicos que podem incluir a PCR. ^373^

***6.1.4.7. Serviços de Hemoterapia***

 O objetivo desses serviços é oferecer sangue de qualidade e, para tal, devem utilizar testes de elevada sensibilidade, capazes de detectar > 99% das amostras infectadas. Porém, esse raciocínio não se aplica ao diagnóstico da doença. Como consequência do zelo necessário para obter sangue sem agentes infecciosos, a especificidade pode ser menor (98%), acarretando exclusão do sangue, porém não significando automaticamente que esse doador em particular esteja infectado. Com frequência, lida-se com um indivíduo que, ao doar sangue, é notificado da sua condição de possivelmente infectado. Nessas circunstâncias, é obrigatório solicitar os dois testes sorológicos de princípios diferentes, como já abordado. Embora na casuística de serviços de referência, entre 70% e 80% dos doadores excluídos sejam efetivamente infectados, uma proporção significativa (20% a 30%) desses indivíduos não terá confirmação de DC, reforçando a necessidade de nova coleta e solicitação de dois testes sorológicos. ^374^

***6.1.4.8. Transmissão Congênita***

 A transmissão vertical (materno-fetal) representa a principal via de transmissão do *T. cruzi* em regiões livres do vetor, assim como em muitas áreas endêmicas. ^59^ A taxa de prevalência deste tipo de transmissão no Brasil é de 1,7%, um dos menores índices comparado a outros países sul-americanos. ^57 , 87^ É importante considerar que bebês nascidos de mulheres com infecção crônica por *T. cruzi,* apresentando sinais clínicos sugestivos de DC aguda, devem ser submetidos aos testes de diagnóstico para infecção o mais rápido possível. O diagnóstico precoce da DC congênita é de enorme importância, considerando que o tratamento tripanossomicida dos recém-nascidos infectados, no primeiro ano de vida, apresenta 100% de cura. 

 Mas, é necessário levar em consideração a possibilidade da passagem de anticorpos (IgG) entre a mãe e o feto por via transplacentária durante a gestação, sendo a mãe infectada com *T. cruzi* e não tendo ocorrido infecção fetal. Assim, para se detectar a transmissão congênita, recomenda-se, preferencialmente, o diagnóstico parasitológico no sangue do cordão ou do recém-nascido nas primeiras 72 horas. Alternativamente, o diagnóstico poderá ser firmado, na ausência de sintomas e sinais de infecção, durante os primeiros meses de vida por meio de métodos parasitológicos diretos (exame a fresco, microhematócrito, creme leucocitário e PCR), com avaliação de duas ou três amostras para ampliação da sensibilidade. ^2^ Os bebês negativos no teste parasitológico inicial devem ser testados por sorologia entre 9 e 12 meses de idade, quando os anticorpos maternos terão desaparecido. A persistência de títulos inalterados de anticorpos anti- *T. cruzi* em crianças a partir de 9 meses de idade é indicativa de infecção congênita e, em contrapartida, a ausência desses anticorpos nesse momento afasta a possibilidade de infecção na criança. ^2^

***6.1.4.9. Sorologia no Indivíduo Infectado, mas Tratado com Quimioterápicos***

 O seguimento de pacientes por meio de exames laboratoriais após o tratamento específico da infecção será abordado mais detalhadamente no capítulo referente à quimioterapia da doença em geral. Aqui, para quem procura subsídios para a exclusão ou confirmação diagnóstica, registre-se que se trata de assunto muito sensível e complexo a ser resumido a alguns princípios. Assim, segundo J. R. Cançado, “é óbvio que se o infectado tem anticorpos e parasitos, para se considerar que está curado (após quimioterapia), ambos teriam que desaparecer”. Essa máxima aplica-se aos tratados na fase aguda (70% de cura) em períodos de meses. Também foi demonstrada em crianças que receberam o tratamento tripanossomicida já em fase crônica, mas recente, comprovando negativação da sorologia (ELISA com antígenos recombinantes) em 58% a 62% dos casos, após 3 a 4 anos de seguimento. ^375 , 376^ Foi verificado também, em proporção menor (25%), nos pacientes tratados na fase crônica tardia, que essa negativação só ocorreu após décadas da realização do tratamento. Trata-se de questão de tempo relacionada ao período de convívio do parasito com o paciente (semanas, anos, décadas). ^377 - 381^ A análise de cura deve ser baseada na negativação das provas sorológicas ou até mesmo na diminuição (desde que expressiva) da concentração dos anticorpos, preferencialmente com testes diagnósticos que utilizem antígenos não purificados. 

***6.1.4.10. Testes Sorológicos Rápidos***

 Os testes de diagnóstico rápido, em geral, são de fácil manipulação e dispensam realização em laboratórios de referência para diagnóstico especializado, em relação às técnicas sorológicas clássicas. Existem diversos tipos disponíveis para diagnóstico da DC. Muitos deles podem ser realizados com soro ou com sangue periférico e podem ser armazenados em temperatura ambiente por longo período de tempo. Seu uso é indicado em áreas endêmicas, principalmente em pesquisa de campo (inquéritos soroepidemiológicos), por contribuir para aumentar o acesso ao diagnóstico em localidades de difícil cobertura. No entanto, apesar de serem utilizados para essa finalidade, os testes rápidos para DC não são comumente recomendados como método de diagnóstico independente pela OMS, devido à baixa sensibilidade. ^382^

***6.1.4.11. Testes Parasitológicos***

 Devem ser solicitados em situações especiais e não de rotina. Existem vários tipos de testes parasitológicos utilizados na fase crônica da DC, que, devido à baixa parasitemia, têm como objetivo promover a multiplicação daqueles poucos parasitos existentes, por meio de hemocultura, xenodiagnóstico, inoculação em animais de experimentação ou a identificação de ácidos nucleicos (DNA ou RNA, pela técnica da PCR) específicos a esse protozoário. 

 A multiplicação de parasitos pode levar várias semanas e, portanto, o resultado pode demorar. São técnicas “ *in house* ”, que demandam condições especiais (reagentes, insetário, biotério), assim como pessoal altamente qualificado. Em geral, são realizados apenas em centros especializados de pesquisa. A hemocultura e o xenodiagnóstico aplicados na fase crônica apresentam sensibilidades baixas e variáveis (cerca de 20%) e, quando repetidos, a probabilidade de detecção pode ser aumentada, atingindo até 60% de sensibilidade. ^365 , 368 , 369 , 383^ Para alguns pacientes com parasitemias muito reduzidas, até mesmo exames sucessivos apresentarão, persistentemente, resultados negativos. 

 No caso do método empregando PCR, a identificação de parte do material genético do parasito demanda menos tempo (horas), porém também exige reagentes e condições técnicas especiais. Pela sua importância, a técnica de PCR será enfatizada a seguir. 

***6.1.4.11.1. Indicações de Testes Parasitológicos, em Particular, Reação em Cadeia da Polimerase***

 Entre as principais, encontra-se o seguimento de pacientes tratados com benznidazol ou outros quimioterápicos. Métodos de diagnóstico acurados e marcadores fidedignos de resposta ao tratamento parasiticida são prioridades na pesquisa e desenvolvimento de recursos em geral para aplicação em DC. ^384^ A PCR tem sido valorizada para avaliação e monitoramento de pacientes, quando um resultado positivo de detecção de material genético do parasito, ao final do tratamento tripanocida, indica falha terapêutica. ^385^ Em contraste, no pós-tratamento, um resultado negativo de PCR não é indicativo de cura da infecção. Cumpre também destacar que a conversão sorológica negativa em pacientes crônicos tratados que apresentam resposta favorável ao tratamento pode levar muitos anos. ^386^ A PCR pode indicar antecipadamente uma resposta de falha terapêutica, demonstrando resistência ao tratamento tripanocida, ou seja, ineficácia do esquema terapêutico. 

 Em casos de RDC, a PCR também é de utilidade, permitindo detecção precoce da mesma. O monitoramento de RDC em indivíduos imunossuprimidos é área de crescente interesse. A RDC em pacientes infectados na fase crônica que adquiriram HIV ou durante terapias imunossupressoras, após transplante de órgãos, doenças autoimunes ou câncer, geralmente induz aumento da parasitemia, caracterizando DC aguda. ^387 - 389^ Nos casos de imunossupressão resultantes de TC, a exclusão do processo de rejeição e detecção da RDC podem ser efetivadas precocemente por meio de PCR realizada em amostras de sangue periférico e de biópsia endomiocárdica. ^390 - 392^

***6.1.4.11.2. Interpretação de Resultados de Testes Parasitológicos***

 Os testes parasitológicos, por definição, só têm valor se forem positivos, ou seja, por crescimento numérico dos parasitos ou pela demonstração de estruturas amplificadas do parasito (PCR). Um teste negativo, em si, não tem valor, pois o resultado só é válido para aquela amostra no dia da coleta. É possível que nova amostra, coletada em outro dia, seja positiva. Ou seja, um teste parasitológico negativo não significa que o indivíduo não esteja infectado pelo *T. cruzi* nem que tenha sido curado da infecção. 

***6.1.4.12. Reação em Cadeia da Polimerase***

 A partir dos anos 1990, a PCR passou a ser utilizada como método molecular de apoio para o diagnóstico de pacientes na fase crônica da DC, devido à sua maior sensibilidade em relação aos testes de multiplicação de parasitos (hemocultura e xenodiagnóstico), além de demonstrar elevado potencial de aplicação no monitoramento de quimioterapia tripanocida. ^393 - 396^

 Vários estudos têm demonstrado resultados positivos por PCR em 40% a 70% dos pacientes crônicos diagnosticados previamente por sorologia convencional. Essa variabilidade na positividade é dependente de inúmeros fatores, como o grau de parasitemia, volume de sangue coletado e da amostra de sangue para isolamento de DNA, método de purificação do DNA, região-alvo a ser amplificada, características das populações de estudo e ainda a elevada diversificação genética, observada entre as DTU do parasito. ^397 - 401^

 Diferentes combinações de alvos moleculares, conjuntos de iniciadores da reação, métodos de extração e plataformas de amplificação de DNA têm sido usadas para avaliar a acurácia do método em amostras de sangue periférico de pacientes com DC crônica; em geral, a sensibilidade alcançada para fins de diagnóstico é mais baixa, comparada aos testes sorológicos. ^401^ Nesse contexto, para esses pacientes, os métodos de detecção com base molecular apresentam um valor diagnóstico limitado, por sensibilidade significativamente mais baixa do que os testes baseados em sorologia. ^400 , 402^

 Ressalte-se que a positividade da PCR confirma a presença do parasito em uma determinada amostra; porém, devido à escassez e intermitência da circulação dos parasitos, características da fase crônica, um resultado de PCR negativo não exclui a infecção. ^398^ Por outro lado, no caso de amostras positivas, a PCR possibilita a caracterização das DTU infectantes do *T. cruzi* diretamente do sangue do paciente, não sendo necessário o isolamento prévio do parasito. 

 Para a seleção do alvo molecular de detecção do material genético de *T. cruzi,* recomenda-se o uso de sequências conservadas do DNA (presentes em todas as linhagens genéticas do parasito), que sejam exclusivas de *T. cruzi* (especificidade), e que essas sequências sejam representadas em múltiplas cópias no genoma (maior sensibilidade). Os alvos mais frequentemente usados na PCR convencional (qualitativa) têm sido o DNA do cinetoplasto ou kDNA (genoma mitocondrial) e as unidades de repetição (DNA satélite) presentes no genoma nuclear. ^402^

 A PCR em tempo real ou quantitativa (qPCR) possibilita determinar a carga parasitária pela quantificação de sequências de DNA específicas. Para os ensaios de quantificação, as sequências de DNA satélite são preferencialmente usadas, devido à menor variabilidade no número de cópias entre as diferentes linhagens genéticas de *T. cruzi* , comparadas aos minicírculos do kDNA. ^403^

***6.1.4.13. Procedimentos Operacionais para Uso da PCR***

1. Coleta de sangue: em geral são coletados 10mL de sangue periférico (mínimo de 5mL) em tubos com EDTA (qualquer outro anticoagulante inibe a enzima da reação). O sangue é imediatamente transferido para tubo contendo o mesmo volume (1:1) de uma solução de lise e preservação da amostra, a solução de 6M guanidina-HCl contendo 0,2M EDTA (pH 8,0).
2. Processamento da amostra: o sangue em guanidina passa por fervura em banho-maria (100ºC, 15 min), a fim de promover uma distribuição homogênea das sequências de DNA-alvo do parasita, possibilitando a extração de DNA de um volume menor da amostra (300 µL). O material fervido permanece à temperatura ambiente por 48 a 72 horas e pode ser submetido à extração de DNA. O restante do material é armazenado em geladeira ou câmara fria, sem jamais congelar.
3. Duas réplicas de 300 µL cada são submetidas à extração de DNA utilizando kits comerciais baseados na purificação por minicolunas de sílica, seguindo as recomendações do fabricante.
4. Os protocolos para PCR seguem aqueles padronizados “ *in house* ” pelos laboratórios, geralmente com base no descrito no consenso internacional. ^402^
5. Para a PCR qualitativa, o resultado do teste se dá pela visualização do produto amplificado (do kDNA ou DNA-satélite) a partir da eletroforese em gel de agarose corado com agentes fluorescentes que se intercalam no DNA.
6. Para a qPCR, os protocolos também seguem o consenso internacional ^403^ e exigem a inclusão, em cada ensaio, de amostras-padrão com concentrações preestabelecidas de parasitos (equivalentes de parasito por reação), que servem como amostras calibradoras para a quantificação absoluta de *T. cruzi* . Os resultados gerados pela qPCR são visualizados, em tempo real, na forma de gráficos emitidos pelo próprio equipamento, sem haver a necessidade de corrida eletroforética.
7. A utilização de controles positivo (DNA extraído de cultivo de células de *T. cruzi* ) e negativo (DNA extraído de sangue sabidamente não infectado e um tubo contendo água ultrapura sem DNA) é fortemente recomendada.
8. Nos casos que resultam em PCR negativa nos ensaios qualitativos, a extração de DNA deve ser repetida a partir de outras duas amostras de sangue em guanidina (300 µL) para a realização de novo teste de PCR dirigido para algum gene humano (β-globina, β-actina, etc). Isso representa um passo decisivo para excluir resultados falso-negativos devido à presença de agentes inibidores nas amostras de sangue ou pela perda ou má qualidade do DNA extraído.

Foi disponibilizado, recentemente, conjunto diagnóstico (kit) para PCR produzido pela FIOCRUZ (Bio-Manguinhos) e aprovado pelas autoridades sanitárias, que facilitará o seu emprego no Laboratório Central de Saúde Pública (LACEN). 

## 6.2. Métodos Diagnósticos de Alterações Cardíacas Estruturais e Funcionais 

 A Tabela 6.1 engloba os exames complementares usados para diagnóstico de cardiomiopatia em indivíduos com DC suspeitada ou já confirmada. Também se explicitam nessa Tabela a força de recomendação e o correspondente nível de evidência que a suporta. A notar que em vários desses exames, além de seu alcance diagnóstico, agrega-se conotação prognóstica. 

**Tabela 6.1:** – Métodos complementares para o diagnóstico e prognóstico da cardiomiopatia crônica da doença de Chagas (CCDC)

Exame	Grau de recomendação	Nível de evidência
**Eletrocardiograma de 12 derivações**		
Avaliação diagnóstica e prognóstica inicial de todo indivíduo com sorologia positiva para doença de Chagas	Forte	B
Repetição anual (ECG com alterações específicas*) ou bianual (ECG normal ou com alterações inespecíficas†) para avaliação evolutiva e prognóstico	Forte	C
Repetição a qualquer momento diante de mudança no quadro clínico	Forte	C
**Radiografia de tórax**		
Avaliação diagnóstica e prognóstica inicial de todo indivíduo com CCDC	Forte	B
Evidência clínica de congestão pulmonar ou sistêmica	Forte	B
**Teste ergométrico ou cardiopulmonar**		
Avaliação diagnóstica e prognóstica inicial de todo indivíduo com CCDC	Ponderado	C
Presença de sintomas, como dor precordial, palpitações, síncope ou pré-síncope, relacionados ao esforço físico ou duvidosos ou de origem não esclarecida	Forte	B
Avaliação periódica de dispositivos implantáveis para otimização da programação e avaliação da capacidade funcional	Ponderado	C
Teste de esforço cardiopulmonar para avaliação funcional, estratificação de risco e auxílio na indicação de transplante cardíaco em IC avançada	Forte	B
**Holter de 24 horas**		
Avaliação diagnóstica e prognóstica inicial de todo indivíduo com CCDC	Forte	B
Investigação de sintomas, como palpitação, pré-síncope e síncope	Forte	B
Seguimento de pacientes com arritmias ventriculares complexas e avaliação de eficácia terapêutica antiarrítmica	Ponderado	C
Seguimento de pacientes com disfunção do nó sinusal ou distúrbios da condução AV/IV potencialmente de risco	Ponderado	C
Seguimento de portadores de dispositivos cardíacos implantáveis (MP, CDI, TRC)	Forte	C
**Estudo eletrofisiológico intracardíaco**		
Avaliação de síncope (ou pré-síncope inquestionável) e suspeita de bradi ou taquiarritmia, quando exames não invasivos foram inconclusivos	Forte	B
Diagnóstico diferencial de taquicardia com QRS largo e diagnóstico incerto	Ponderado	C
**Ecocardiografia convencional**		
Avaliação diagnóstica e prognóstica inicial de todo indivíduo com CCDC	Forte	B
Suspeita de CCDC pela história, exame clínico ou alterações eletrocardiográficas	Forte	B
Quando há piora dos sintomas de IC ou em vigência de síncope, eventos arrítmicos, AVC ou tromboembolismo periférico	Forte	B
Reavaliação periódica** independente da presença de disfunção sistólica global ou regional em exame prévio	Ponderado	C
Avaliação de indivíduos com ECG normal e sintomas sugestivos de CCDC	Ponderado	C
**Medicina Nuclear (Ventriculografia Radioisotópica)**		
Avaliação da função ventricular, especialmente do ventrículo direito, em complementação ao ecocardiograma, como alternativa à ressonância magnética	Ponderado	C
Identificação de defeitos da contratilidade regional, quando o ecocardiograma é tecnicamente inadequado, como alternativa à ressonância magnética	Ponderado	C
**Medicina Nuclear (Perfusão Miocárdica)**		
Avaliação inicial de indivíduos com CCDC e dor precordial	Ponderado	C
Avaliação complementar e detecção de defeitos microvasculares em casos com dor precordial e coronárias angiograficamente normais	Ponderado	B
**Medicina Nuclear (Avaliação da Inervação Simpática)**		
Como método complementar para avaliação de arritmias ventriculares complexas	Ponderado	C
**Ressonância Magnética Cardíaca**		
Para suspeita de concomitância de CCDC com doença arterial coronária ou outra cardiomiopatia não isquêmica e avaliar a etiologia da fibrose miocárdica e sua extensão	Ponderado	B
Avaliação morfológica e funcional, global e segmentar, e pesquisa de trombos, como alternativa ao ecocardiograma com limitações técnicas	Ponderado	B
Quando não há suspeita clínica inicial de doença de Chagas (p.ex. área não endêmica), a RMC pode favorecer um diagnóstico extremamente provável de CCDC pelo padrão de realce tardio e motivar a realização de testes sorológicos para confirmação do diagnóstico etiológico	Ponderado	C
Discordância entre sintomas e grau de disfunção miocárdica no contexto de indicação ambígua de procedimentos, como o implante de cardioversor-desfibrilador	Ponderado	C
Planejamento de estudos eletrofisiológicos com possível ablação por radiofrequência (guia local para ablação) ou implante de dispositivo para definir se a terapia de ressincronização deve ou não ser adicionada (fibrose lateral e/ou septal extensa é considerada relativa contraindicação por elevar a taxa de não respondedores)	Ponderado	C
**Tomografia computadorizada**		
Avaliação da anatomia coronariana em pacientes com CCDC e ainda com alta probabilidade de doença arterial coronária obstrutiva	Ponderado	B
Caracterização do miocárdio normal/fibrose pela técnica do realce tardio em alternativa à ressonância magnética cardíaca	Ponderado	C
Avaliação da função sistólica biventricular em complementação à ecocardiografia em pacientes com contraindicação à ressonância magnética cardíaca	Ponderado	C
**Cateterismo Cardíaco**		
Avaliação da anatomia coronariana em pacientes com CCDC e ainda com alta probabilidade de doença arterial coronária obstrutiva	Forte	B
Avaliação de resistência vascular pulmonar em candidatos a transplante cardíaco com evidência não invasiva de hipertensão pulmonar	Ponderado	B

*Principalmente quando múltiplas: bloqueio completo do ramo direito, em especial associado a bloqueio divisional anterossuperior esquerdo (BDASE), extrassístoles ventriculares frequentes, ondas Q patológicas ou áreas de inatividade elétrica, bloqueio atrioventricular (BAV) de segundo (Mobitz 2) ou terceiro grau e fibrilação atrial; †Principalmente quando únicas: BDASE isolado, bradicardia sinusal, BAV de primeiro grau, baixa voltagem do complexo QRS em derivações periféricas e anormalidades inespecíficas do segmento ST-T. IC: insuficiência cardíaca; AV: atrioventricular; IV: intraventricular; MP: marca-passo; CDI: cardioversor-desfibrilador implantável; TRC: terapia de ressincronização cardíaca. **3 a 5 anos para casos com ecocardiograma prévio evidenciando fração de ejeção preservada e sem alteração segmentar da contratilidade; 1 a 2 anos para casos com disfunção ventricular esquerda global (mesmo leve) ou segmentar. IC: insuficiência cardíaca; AVC: acidente vascular cerebral

### 6.2.1. Eletrocardiograma na Doença de Chagas

 O ECG é o exame cardiovascular inicial mais importante para avaliação de pacientes com DC, permitindo a classificação da forma clínica da doença. ^330 , 404^ Assim, alterações eletrocardiográficas bem definidas no indivíduo infectado indicam a presença de cardiomiopatia. ^334^ As alterações mais frequentes e definidas são retardos da condução atrioventricular, da condução no ramo direito e no fascículo anterossuperior, alterações da repolarização ventricular e ectopias ventriculares. Praticamente todas as anormalidades eletrocardiográficas podem ser encontradas na DC, com predomínio de alterações na formação e condução da atividade elétrica cardíaca. 

 O BRD, completo ou incompleto, é o distúrbio de condução mais comum na DC, sendo encontrado em 10% a 50% dos pacientes infectados, dependendo das características da amostra estudada. ^330 , 404 , 405^ O BRD está frequentemente associado ao BDASE, a mais comumente encontrada combinação na CCDC. O bloqueio do ramo esquerdo (BRE) é raro e apresenta pior prognóstico. 

 Os BAV são também comuns, apresentam-se de graus variados e podem ser a primeira manifestação da doença. Os BAV avançados são decorrentes de lesões extensas do nó atrioventricular e sistema de His-Purkinje, podem evoluir com quadros sincopais e necessidade de implante de MP artificial definitivo e predispõem a morte súbita por assistolia. 

 A disfunção do nó sinusal frequentemente se expressa por bradicardia e pode ocasionar episódios de bloqueio sinoatrial e paradas sinusais. Quando a disfunção dessa estrutura é acompanhada por sintomas de hipofluxo cerebral, caracteriza-se a doença do nó sinusal, que, em alguns pacientes, tipicamente alterna a bradicardia com episódios de taquicardia. 

 A FA na CCDC constitui alteração mais tardia, encontrada em até 5% dos traçados eletrocardiográficos. ^330 , 332 , 404^ Em geral, a FA está associada a dano miocárdico mais pronunciado e extenso, envolvimento difuso do sistema de condução, arritmias ventriculares e AVC. 

 As arritmias ventriculares, como as EV polimórficas e a taquicardia ventricular (TV), são preditoras de síncopes e de morte súbita cardíaca por FV. Ondas Q patológicas ou perda de progressão de ondas R de V1 a V3-V4 traduzem áreas elétricas inativas e são decorrentes de fibrose miocárdica. Já os transtornos difusos da condução e a baixa voltagem de QRS geralmente estão associados a disfunção ventricular acentuada. ^332^

 A associação de duas ou mais anormalidades no mesmo traçado eletrocardiográfico constitui uma das características de cardiopatia grave. A mais frequente é a presença de distúrbios de condução associados a arritmias ventriculares. A coexistência de ondas Q patológicas também indica comprometimento mais significativo da função ventricular. Dessa forma, quanto maior for o número de alterações eletrocardiográficas apresentadas pelo paciente, pior será seu prognóstico. 

 Os tradicionais estudos epidemiológicos, avaliando as alterações eletrocardiográficas na DC, foram realizados no contexto predominante de infectados por transmissão vetorial clássica, incluindo indivíduos mais jovens. ^334 , 404^ Com o atual controle mais abrangente da transmissão vetorial e o envelhecimento da população infectada pelo *T. cruzi* , doenças crônicas, como a cardiopatia hipertensiva e a cardiopatia isquêmica, podem coexistir com a CCDC e anormalidades típicas dessas condições podem se sobrepor às típicas da DC. ^332^ Além disso, embora existam anormalidades típicas na CCDC, nenhuma delas é específica para essa etiologia, tampouco aparece em todos os casos. 

 Corroborando a conotação apontada acima sobre o efeito prognóstico das anormalidades eletrocardiográficas, recentes investigações por grupos independentes de pesquisadores destacam a potencial contribuição da análise de alterações no ECG, inclusive usando recursos de inteligência artificial e aprendizado de máquina, para se prever a detecção de disfunção ventricular e fibrose miocárdica, dois prognosticadores fundamentais na DC. ^406 , 407^

 O ECG deve ser realizado quando se suspeita ou se confirma o diagnóstico da DC, devendo ser repetido regularmente para se avaliar o aparecimento ou evolução de anormalidades. Nos indivíduos com ECG normal, novas alterações indicam progressão para a forma cardíaca, o que implica na realização de exames adicionais. ^5 , 7^ Para pacientes com sintomas sugestivos de arritmias cardíacas, como palpitações, lipotimia, síncope e morte súbita recuperada, um ECG de repouso é obrigatório antes da realização de novos testes, como Holter, ECG de estresse ou estudo eletrofisiológico (EEF) intracardíaco. 

### 6.2.2. Radiografia de Tórax

 A radiografia de tórax, dada sua ampla disponibilidade, é um dos exames utilizados no diagnóstico de comprometimento cardiovascular e, principalmente, na avaliação de congestão pulmonar. Mesmo em pacientes sintomáticos, é comum encontrar-se aumento de área cardíaca com campos pulmonares pouco congestos. Os sinais de aumento do VD em projeções póstero-anterior e perfil também são comuns e significativos, assim como pode haver sinais de derrame pleural à direita, secundários à congestão sistêmica. O aumento do ICT é fator preditor independente de morte em indivíduos com CCDC. ^408^ Estudo recente demonstrou que a presença de cardiomegalia pelo ICT é adequadamente identificada pelo aumento do DDVE, medido pela ecocardiografia. ^337^

### 6.2.3. Ecocardiografia

 O ECO é o exame de imagem mais utilizado na avaliação inicial e no seguimento de pacientes com DC. ^339^ Os sinais ecocardiográficos podem variar desde alterações localizadas de contração segmentar nos estágios iniciais da cardiopatia até dilatação importante das câmaras cardíacas com disfunção biventricular nos estágios mais avançados. A presença e a gravidade das alterações ao ECO, associadas aos dados clínicos, são critérios empregados para a classificação da DC em estágios de A a D, com valor prognóstico intrínseco, como exposto em outro capítulo desta diretriz. 

***6.2.3.1. Função Sistólica do Ventrículo Esquerdo***

 A CMD da DC caracteriza-se pelo aumento ventricular esquerdo e por hipocinesia segmentar e/ou difusa, sendo a disfunção sistólica dessa câmara o mais importante preditor de morte. ^408^ Em razão da presença de alterações geométricas e segmentares, o modo M não é recomendado para a avaliação das dimensões e da função sistólica do VE. Essa análise deve ser realizada preferencialmente pelo modo bidimensional, por meio da estimativa de volumes, com o método biplanar (Simpson). Assim como em outras cardiomiopatias, a ecocardiografia tridimensional é superior à bidimensional para a avaliação dos volumes e da fração de ejeção, principalmente quando há suspeita de encurtamento da imagem apical do VE ou quando há anormalidades na contração segmentar com distorção da geometria, como nos aneurismas frequentemente visibilizados com o método. 

 A ecocardiografia com rastreamento de pontos, ou STE, permite o diagnóstico precoce de disfunção sistólica pela avaliação da deformação miocárdica em pacientes com DC. A deformação sistólica nos eixos longitudinal, radial e circunferencial já foi avaliada em pacientes com FIDC ou com cardiopatia em vários estudos. Os resultados mais consistentes avaliaram o GLS, assim como em outras cardiomiopatias não isquêmicas. Mesmo em pacientes nos estágios mais precoces da cardiopatia, como aqueles com fração de ejeção preservada (estágio B1) ou ainda aqueles com a FIDC (estágio A), alterações regionais na deformação miocárdica são observadas. Nos pacientes com a FIDC, as alterações regionais descritas pela STE ocorrem principalmente em segmentos inferiores e ínfero-laterais de VE. ^313 , 409 , 410^ O valor prognóstico dessas alterações regionais precoces em pacientes na FIDC ainda não está definido. Estudo recente, incluindo 144 pacientes com DC, porém sem evidências de acometimento cardíaco, mostrou que o *strain* radial avaliado pelo STE foi preditor de desenvolvimento de cardiomiopatia. ^411^ Em pacientes com FEVE reduzida e CCDC ou CMD idiopática, o GLS reduzido foi preditor de desfechos combinados independentemente da FEVE. ^412^

***6.2.3.2. Alterações Segmentares da Contratilidade Ventricular***

 As alterações segmentares podem estar presentes em 10% dos pacientes no estágio inicial da doença e em até 50% quando há dilatação e disfunção sistólica. Essas alterações regionais de mobilidade parietal, quando incipientes, identificam indivíduos sob risco de evolução para disfunção ventricular global e surgimento de arritmias. ^341 , 413^ Em pacientes com CCDC, o índice de escore de mobilidade segmentar alterado em repouso (> 1) foi capaz de identificar aqueles com maior risco para desfechos clinicamente relevantes, inclusive mortalidade global, apesar de função ventricular global inicialmente preservada. ^342^ As alterações segmentares são encontradas mais frequentemente nas paredes inferior e inferolateral, além de nos segmentos apicais. O padrão regional de acometimento, não relacionado ao território coronariano, é característica dessa cardiomiopatia. 

 Os aneurismas ventriculares apresentam-se de forma variável, desde tamanho diminuto, com conformação digitiforme (em “dedo de luva”), até grandes aneurismas apicais (“saculares”), que podem ser difíceis de diferenciar dos encontrados na cardiopatia isquêmica. ^339^ A prevalência média de aneurisma apical nas diferentes séries ecocardiográficas foi de 8,5% (variando de 1,6% a 8,6%) em pacientes assintomáticos ou com cardiopatia leve e de até 55% (variando de 47% a 64%) em pacientes com moderada a importante disfunção sistólica de VE. ^339^ Os aneurismas não são limitados ao ápice ou à parede inferolateral, podendo ser encontrados no septo, na parede ântero-lateral e no VD. ^340^ Trombos intraventriculares podem estar associados a esses aneurismas e são considerados fator de risco importante para eventos embólicos. 

 Apesar de o exame ecocardiográfico transtorácico em repouso ser de fundamental importância na avaliação da CCDC, pois permite identificar alterações segmentares, principalmente os aneurismas apicais, sua execução pode ser tecnicamente desafiadora. O uso de inspiração profunda e de incidências ecocardiográficas não convencionais, como corte intermediário entre apical de 4 e 2 câmaras, com angulação posterior do transdutor, pode ser necessário, assim como o uso complementar de imageamento com contraste ultrassonográfico. 

***6.2.3.3. Função Diastólica do Ventrículo Esquerdo***

 A alteração do relaxamento miocárdico é a primeira a surgir, podendo estar presente mesmo em pacientes com a FIDC. Com a progressão da cardiomiopatia, a disfunção diastólica pode agravar-se e apresentar padrão restritivo típico. ^414 , 415^ A análise da função diastólica pode ser desafiadora, por fatores de confundimento, em razão da presença eventual de FA e de MP em câmaras direitas. O aumento gradual da relação E/e’ ocorre a partir da FIDC e um valor maior que 15 é preditor de pior desfecho em pacientes com disfunção sistólica apenas discreta a moderada. ^416^ Há evidências de que a relação E/e’ se correlaciona, de forma independente, com os níveis sanguíneos de peptídeo natriurético do tipo B (BNP). ^417^

 A disfunção diastólica contribui decisivamente para o remodelamento atrial, que pode ter seu volume aumentado em qualquer estágio da CCDC. ^418^ O volume do átrio esquerdo correlaciona-se, de forma independente, com a mortalidade. ^342 , 415 , 419^ A função atrial esquerda na CCDC está mais comprometida do que em outras etiologias, como na CMD idiopática, provavelmente devido a um acometimento miopático atrial intrínseco associado. ^420^ Quando avaliada pelo *strain* , a função atrial esquerda também se mostrou preditor independente de eventos clínicos em pacientes com a DC. ^418^ De forma semelhante, índices de disfunção do átrio esquerdo avaliados pela ecocardiografia tridimensional e pelo *strain* foram preditores independentes para o surgimento de FA de início recente no seguimento desses pacientes. ^421^

***6.2.3.4. Avaliação do Ventrículo Direito***

 A avaliação de VD pela ecocardiografia convencional, usando projeções dedicadas, permite a quantificação de suas dimensões, volumes (ECO 3D) e função contrátil, e deve ser realizada em todos os pacientes com CCDC. Embora frequentemente associado à disfunção de VE, ^348^ o comprometimento do VD pode, mais raramente, ocorrer de forma primária e prematuramente em relação ao acometimento do VE. ^345^ A disfunção sistólica de VD, avaliada por meio de parâmetros ecocardiográficos convencionais, como o índice de Tei, foi preditor independente de mau prognóstico na CCDC. ^350^ O estudo da função sistólica de VD pela técnica de STE, em especial na parede livre da câmara, apresentou acurácia satisfatória, correlacionando-se com outros métodos, como a RMC. ^347^ A ecocardiografia tridimensional também constitui ferramenta promissora na avaliação da função sistólica do VD. 

***6.2.3.5. Ecocardiograma sob Estresse***

 O ECO sob estresse farmacológico (ou talvez também com esforço físico) pode demonstrar a presença de reserva contrátil bifásica nesses pacientes, que tipicamente apresentam coronárias subepicárdicas sem obstruções. ^422^ Embora o exame farmacológico use comumente a dobutamina, provida de potencial arritmogênico, evidenciou-se segurança do método na CCDC, sendo o índice de contração segmentar alterado em repouso um preditor independente para o surgimento de arritmias durante o exame. ^423^

### 6.2.4. Ressonância Magnética Cardíaca

 Embora a RMC não seja exame de avaliação inicial da DC, o método tem se mostrado útil no diagnóstico e estratificação de risco da CCDC. Pacientes em investigação de cardiomiopatia e sem suspeita específica de DC e que não vivem em área endêmica frequentemente não são submetidos a testes sorológicos para DC. Nesses casos, um padrão de disfunção sistólica global ou regional típico, associado a padrão e localização específica da fibrose miocárdica pela RMC, pode levantar a suspeita e indicar a necessidade de se desencadear o teste sorológico específico. 

 Além disso, a RMC é capaz de estimar o prognóstico. A quantidade de fibrose miocárdica correlaciona-se fortemente com marcadores de gravidade da doença, arritmias ventriculares, eventos cardiovasculares graves e mesmo morte. ^311 , 424^ A RMC pode ainda ser útil para detectar envolvimento miocárdico precoce na DC, principalmente na FIDC, quando, em geral, todos os outros exames são normais. ^310 , 311^

 À RMC, novas ferramentas não invasivas podem identificar atividade inflamatória miocárdica (edema e hiperemia miocárdica) em estágio inicial antes do desenvolvimento de lesões irreversíveis, como necrose e fibrose, e eventualmente auxiliar na estratificação de risco e, quiçá, na decisão terapêutica. ^310 , 425^

 O imageamento por RMC provou ainda ser útil para detectar trombos intracardíacos em pacientes selecionados, especialmente aqueles com imagens ecocardiográficas limitadas e sem indicação de angiocardiografia invasiva. ^310 , 311 , 426 , 427^

 Investigações recentes indicam ter a RMC bom potencial para avaliar o prognóstico de pacientes com CCDC, independentemente do já provido pelo escore de RASSI, talvez permitindo a reestratificação daqueles com risco baixo ou intermediário de morte. ^428 , 429^ Esse potencial prognóstico da RMC na CCDC muito provavelmente dependerá de confirmação por estudos em andamento e deverá corroborar a amplificação dos métodos de estratificação de risco já empregados. ^430^

 O exame de RMC deve incluir avaliação da função sistólica biventricular por técnicas de SSFP ( *steady-state free precession* ), imagens ponderadas em T2 e/ou mapa T2 para avaliação de edema miocárdico e obrigatoriamente o emprego de gadolínio para detectar pelo realce tardio miocárdico a fibrose miocárdica regional macroscópica. É ainda oportuno que seja incluída a técnica de mapa T1 miocárdico pré (nativo) e pós-contraste para cálculo do volume extracelular do miocárdio, que é uma medida de fibrose intersticial e difusa, que pode estar presente nessa cardiomiopatia, mesmo em regiões miocárdicas sem realce tardio evidente. O realce global ponderado em T1 antes e depois do contraste (técnica de *spin-echo* rápido, semelhante ao critério de Lake Louise, original para miocardite viral) ou o realce precoce com gadolínio pode ser útil para a detecção de hiperemia/inflamação. A aquisição de realce tardio com um tempo de inversão longo (~ 600ms) também deve ser usada, especificamente na suspeita de trombo intracavitário, para aumentar a sensibilidade de sua detecção. 

 Para avaliação de insuficiência mitral ou tricúspide, usualmente presentes na cardiomiopatia avançada da DC, cine-ressonância e cine com contraste de fase (mapa de fluxo) são as técnicas utilizadas. 

 Merece relato o exemplo clássico de CCDC pela RMC, que envolve os segmentos ínfero-laterais basais e médio e o ápice do VE, com alterações contráteis típicas pela cine-ressonância e fibrose miocárdica de padrão e distribuição característicos pelo realce tardio. Um aneurisma apical típico de VE com morfologia “dedo em luva” pode ser claramente visto nas imagens de cine-ressonância e no realce tardio. 

 Recente posicionamento científico sobre DC da *American Heart Association* recomendou a RMC em pacientes selecionados com cardiopatia para avaliar a extensão da fibrose e até mesmo exames seriados de RMC para indivíduos com arritmias ventriculares complexas, especialmente TVNS. ^7^

 Em outro documento de consenso sobre imageamento em DC da *European Association of Cardiovascular Imaging* e do Departamento de Imagem Cardiovascular da SBC, foi recomendado que a RMC deva ser indicada em pacientes selecionados com arritmias ventriculares graves para quantificar a extensão da fibrose miocárdica e avaliar o risco de morte súbita com potencial impacto na indicação de implante de cardioversor-desfibrilador implantável (CDI). Ainda, a RMC deveria ser indicada para avaliação da FEVE quando a ecocardiografia básica for considerada insatisfatória e não estiver disponível a ecocardiografia com contraste ou a tridimensional. 

### 6.2.5. Medicina Nuclear

 É modalidade de imageamento não invasiva, mas que requer uso de radiação. No caso da DC, o exame pode ser utilizado para a análise da função biventricular como alternativa à RMC e para analisar a perfusão miocárdica diante da suspeita de coronariopatia em nível subepicárdico ou microvascular, além de para avaliar a inervação cardíaca simpática. ^205^

***6.2.5.1. Ventriculografia Radioisotópica***

 A medicina nuclear é opção para a análise da função sistólica de ambos os ventrículos, em especial nos pacientes que mostram impedimento ou contraindicação à realização de RMC e nos raros casos em que a ecocardiografia se mostra inexequível tecnicamente. Poderia ser considerado o método padrão-ouro para mensuração da fração de ejeção de ambos os ventrículos por permitir amostragem integrada de muitos ciclos cardíacos, assim minimizando a variabilidade ocasional que limita, em algumas circunstâncias, a confiabilidade de métodos que analisam apenas poucos ciclos, e para determinação dos volumes diastólico e sistólico, sem recorrer a pressupostos de ordem geométrica. Também fornece informações relacionadas à contratilidade regional e à presença de aneurismas ventriculares, tão característicos dessa entidade. 

 A avaliação da função diastólica, cuja alteração pode ser uma das manifestações mais precoces na DC, é feita, mas com limitações, pela ventriculografia radioisotópica. Por outro lado, a disfunção ventricular direita, que também pode ser um sinal precoce dessa cardiopatia, pode ser avaliada com precisão por meio das técnicas de medicina nuclear, mas seu emprego em pacientes com CCDC ainda é limitado logisticamente. ^205 , 431^

***6.2.5.2. Perfusão Miocárdica***

 A prevalência de doença coronária obstrutiva não costuma ser elevada em pacientes com CCDC, mesmo quando apresentam dor precordial. Por outro lado, há relatos independentes, por vários investigadores, de que ocorra disfunção da microcirculação coronária nesses pacientes e a presença de defeitos de perfusão tem valor prognóstico, pois pode preceder o desenvolvimento de disfunção contrátil miocárdica. 

 A existência de alterações cintilográficas em pacientes com CCDC pode traduzir o mecanismo inflamatório pelo qual, ao menos em parte, há destruição de músculo cardíaco nessa entidade e sua substituição por tecido fibrótico. A cintilografia miocárdica de perfusão baseada em tomografia computadorizada empregando radiotraçadores emissores de fótons singulares ( *SPECT-CT* ) é eficaz para detectar distúrbios da irrigação no músculo cardíaco, mesmo diante da ausência de lesões nas artérias coronárias epicárdicas. ^182^

 Com menor disponibilidade logística, o PET/TC é alternativa para estudo de alterações inflamatórias, de perfusão e de perda ou preservação de viabilidade miocárdica em áreas ventriculares exibindo déficit contrátil, como exame adequado para o estudo da microcirculação. ^431^

***6.2.5.3. Avaliação da Inervação Simpática***

 A depressão da inervação simpática do miocárdio em nível ventricular ocorre precocemente na DC e talvez em maior intensidade do que em outras cardiopatias. Isso pode estar associado com a perda do controle autonômico reflexo e mesmo anteceder qualquer outro comprometimento cardíaco. A medicina nuclear, utilizando cintilografia com I-MIBG, ^123^ permite detectar defeitos da inervação simpática ventricular, em especial nas paredes inferior, póstero-lateral e apical, muito antes de haver defeito contrátil nesses segmentos. ^183^ É possível que essas alterações de inervação miocárdica estejam associadas a maior risco de ocorrer TVS e a pior prognóstico evolutivo. ^205 , 208^

### 6.2.6. Tomografia Computadorizada das Artérias Coronárias 

 Esse método diagnóstico, à semelhança do que ocorre com a angiocardiografia invasiva baseada em cateterismo cardíaco, também emprega radiação ionizante e meio de contraste iodado, sendo primariamente utilizado para o estudo não invasivo da anatomia coronária em diversos contextos clínicos. 

 Na DC, a experiência com essa abordagem na prática clínica ainda é limitada e aplica-se mais a pacientes que apresentam contraindicação para outros métodos de imagem, tais como a RMC e a cintilografia do miocárdio, e nos quais o estudo ecocardiográfico mostra limitações técnicas. ^432^ Seu emprego, em termos gerais, talvez seja mais indicado quando a probabilidade de doença coronária obstrutiva subepicárdica é baixa, mas deve ser descartada em pacientes com CCDC apresentando precordialgia atípica. 

 Experiência preliminar com o método demonstrou que pacientes brasileiros com DC têm prevalência reduzida de doença coronária obstrutiva em nível subepicárdico, assim corroborando evidências mais antigas, lastreadas em estudos angiográficos invasivos. ^269^

### 6.2.7. Eletrocardiografia Dinâmica (Holter)

 A CCDC possui patogênese complexa, multifatorial, que inclui agressão tecidual pelo parasita e resposta imunológica exacerbada, levando a reação inflamatória, acometimento do sistema nervoso autônomo e comprometimento de microcirculação. O resultado final desses mecanismos patogenéticos é a necrose celular e sua substituição por áreas localizadas de fibrose miocárdica. 

 As zonas de fibrose apresentam predileção pelo sistema excito-condutor (nó sinusal, nó atrioventricular, ramos e fascículos do sistema His-Purkinje), ^433^ sendo, frequentemente, manifestação primordial do acometimento cardíaco. A combinação de áreas de fibrose, disfunção autonômica e comprometimento do sistema excito-condutor favorece a ocorrência tanto de bradiarritmias quanto de taquiarritmias, muitas vezes antes que alterações estruturais cardíacas sejam detectadas por exames de imagem, como o ECO. Essa manifestação precoce das arritmias na CCDC caracteriza a doença como uma forma de miocardiopatia arritmogênica, ^434^ podendo ser a morte súbita sua primeira manifestação clínica. ^353^ De fato, a morte súbita cardíaca é a principal causa de morte na doença, sendo responsável por cerca de 60% dos óbitos. ^352^

 A detecção de arritmias cardíacas pelo Holter ou durante teste ergométrico é parte essencial da avaliação rotineira de pacientes com CCDC, possibilitando diagnosticar disfunção do nó sinusal, distúrbios na condução atrioventricular, ectopias e taquiarritmias supraventriculares, ectopias ventriculares e TVNS ou TVS. 

 Estudo avaliando a ocorrência das ectopias ventriculares pelo Holter em pacientes com CCDC evidenciou que o comportamento aparentemente aleatório dessa arritmia em gravações de 24 horas deixa de existir quando se analisam períodos mais longos, de 7 dias, sugerindo que gravações mais longas de Holter seriam mais adequadas nesse contexto. ^435^

 A presença de TVNS no Holter é preditor independente de mortalidade geral em pacientes com CCDC. ^408 , 436^ No escore de RASSI, a identificação de TVNS ao Holter soma 3 pontos de um total de 18 ou 20 pontos possíveis (mulheres e homens, respectivamente). Além da estratificação de risco, o Holter permite a avaliação de sintomas como palpitações, lipotimias e síncopes, frequentes nesses pacientes e, em muitos casos, decorrentes das diversas formas de arritmia encontradas. 

 O Holter também possibilita a avaliação do sistema nervoso autônomo por meio de análise da variabilidade da frequência cardíaca (VFC). Vários estudos demonstraram alterações autonômicas em diferentes estágios e formas da DC. ^437 , 438^ Há disfunção parassimpática predominante, mas também acometimento simpático (menor intensidade), ^204^ e indícios preliminares, em estudo retrospectivo, de que tais alterações, refletindo-se em diversos parâmetros de VFC, possam sinalizar risco de morte súbita. ^439^ A VFC avaliada durante registros curtos de Holter e com emprego de técnica de aprendizado de máquina também mostrou capacidade de predição de alterações ecocardiográficas ^440^ e pôde ser correlacionada ao escore de RASSI, o mais avalizado prognosticador do risco de mortalidade, em cardiomiopatas com ou sem envolvimento digestivo associado. ^441^

### 6.2.8. Estudo Eletrofisiológico Intracardíaco

 Na CCDC ocorrem substratos arritmogênicos reentrantes relacionados às áreas de fibrose e o EEF permite a indução de TVS ou mesmo FV que, em alguns contextos, passam a ter conotação prognóstica. ^442^ O EEF permite também avaliar o nó sinusal e a condução atrioventricular, além de definir com precisão se o distúrbio dromotrópico localiza-se no nó atrioventricular, no feixe de His ou é infra-hissiano. A frequente ocorrência paroxística de BAVT pelo acometimento do sistema His-Purkinje, consequente ao seu conhecido comportamento de condução na forma “tudo ou nada” (ou conduz ou não conduz, sem apresentar estágios intermediários de mau funcionamento), faz com que, em determinados casos, apenas a investigação invasiva com EEF permita o diagnóstico preciso e o tratamento adequado do paciente. ^442^

### 6.2.9. Teste Ergométrico e Teste Cardiopulmonar

 O teste de esforço máximo convencional e o de avaliação cardiopulmonar podem detectar alterações importantes, incluindo arritmias ventriculares induzidas pelo exercício e incompetência cronotrópica. ^405 , 443^ No entanto, a aplicabilidade clínica geral dos testes de exercício não está bem estabelecida, embora o cardiopulmonar, com medida direta do consumo de oxigênio (VO _2_ máximo), possa ser considerado o padrão-ouro para avaliação da capacidade funcional e eficácia dos programas de reabilitação. ^444^

 Arritmias ventriculares induzidas pelo esforço constituem um marcador de risco de morte cardiovascular em pacientes com DC. ^443^ Como essas arritmias também ocorrem em pacientes sem cardiopatia aparente, o teste de exercício máximo convencional é clinicamente relevante para estratificação de risco na população com DC, especialmente para orientações trabalhistas. 

 Poucos estudos verificaram a eficácia das variáveis avaliadas por meio dos testes de exercício na predição de sobrevida dos pacientes com CCDC. O VO _2_ pico é critério importante para o TC em pacientes com formas avançadas de cardiopatia. Entretanto, seu valor prognóstico deve ser mais bem compreendido no contexto de estratégias preventivas, estratificação de risco e diagnóstico precoce. Além disso, é necessário estabelecer pontos de corte para serem empregados especificamente na CCDC. 

### 6.2.10. Cateterismo Cardíaco

 Conforme apontado acima, pacientes com CCDC frequentemente apresentam dor torácica atípica e anormalidades eletrocardiográficas, como alterações no segmento ST e ondas Q patológicas, além de distúrbios regionais de contratilidade e de perfusão miocárdica que mimetizam doença coronária aterosclerótica. Na maioria desses casos, a avaliação das coronárias epicárdicas demonstra ausência de doença aterosclerótica obstrutiva subepicárdica, atribuindo-se essas alterações à disfunção microvascular coronariana. ^336 , 445^

 Estudos recentes evidenciaram que a disfunção ventricular associada à doença microvascular de etiologia da DC é mais proeminente do que a verificada quando esse distúrbio microcirculatório decorre de outras etiologias. ^446^ Além disso, esses investigadores relataram melhora sintomática e da perfusão miocárdica quando os pacientes com CCDC foram tratados com inibidor plaquetário e vasodilatador microvascular, a primeira demonstração de benefício alcançado nesse contexto. ^447^

 O cateterismo cardíaco pode ser empregado, portanto, quando pacientes com média ou alta probabilidade de doença arterial coronariana obstrutiva apresentam dor anginosa típica e/ou múltiplos fatores de risco para doença aterosclerótica ou têm grande área isquêmica demonstrada em exames não invasivos. Durante o estudo hemodinâmico, a ventriculografia de contraste radiológico, por sua elevada resolução temporal e espacial, pode indigitar pequenos aneurismas apicais e/ou outras alterações segmentares na contração ventricular, que poderiam não ser detectadas por outros métodos de imageamento. ^336^ O cateterismo cardíaco também pode ser realizado em pacientes candidatos a TC por IC avançada para avaliar a resistência vascular pulmonar. Além disso, possibilita a biópsia endomiocárdica pós-transplante, quando a diferenciação de rejeição *versus* reativação da infecção por *T. cruzi* se torna mandatória em alguns pacientes. 

## 7. Estratificação de Risco e Prognóstico

**Figura 7.1:**
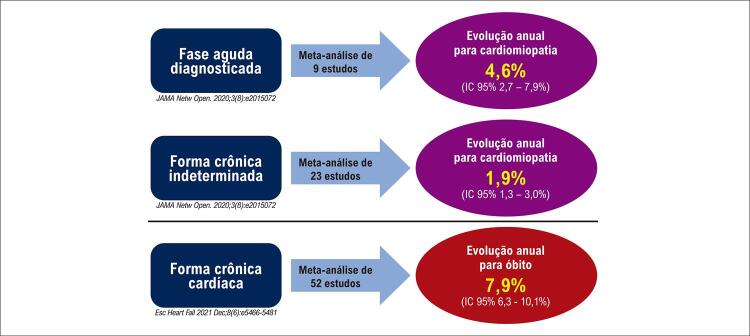
– Taxa anual de evolução da doença de Chagas (fase aguda diagnosticada e forma crônica indeterminada) para cardiomiopatia e dessa para óbito.

 A CCDC pode manifestar-se de inúmeras formas, dependendo basicamente da gravidade das alterações do miocárdio e do sistema específico de geração e condução elétrica, da presença e do tipo de arritmia e da existência de IC. Duas revisões sistemáticas com meta-análises respectivas foram recentemente divulgadas. Em uma delas, avaliou-se o risco do desenvolvimento de cardiomiopatia crônica em indivíduos que estavam na fase aguda (estimativa global de que isso ocorra em 4,6% [IC 95%: 2,7%-7,9%] anualmente) ou que tinham a FIDC (estimativa anual de 1,9% [IC 95%: 1,3%-3,0%]). ^297^ Em outra com 52 estudos incluindo somente pacientes com cardiopatia manifesta, revelaram-se taxa anual média de mortalidade de 7,9% [IC 95%: 6,3%-10,1%], mas com ampla heterogeneidade de resultados, e taxas individuais variando entre 0,5% e 38,3%/ano, dependendo das características de base da população incluída em cada estudo. ^448^ ( Figura 7.1 ) 

 Nas últimas décadas, vários fatores de risco para morbimortalidade foram identificados para quantificar a gravidade da CCDC, avaliar seu prognóstico e, eventualmente, sugerir estratégias terapêuticas mais adequadas. Infelizmente, quando consideradas isoladamente, variáveis associadas a um pior prognóstico em geral apresentam baixo valor preditivo positivo, limitando seu uso. Assim, passou-se a investigar modelos prognósticos construídos a partir de combinações variadas de parâmetros demográficos, clínicos e laboratoriais. 

 Para ser aplicado na prática clínica, o modelo de estratificação de risco deve ser simples e utilizar variáveis bem definidas, de fácil acesso e em número não excessivo, além de apresentar poder discriminatório (estatística C) satisfatório. Mais importante ainda, deve ser validado por investigadores de outros centros (validação geográfica) e em períodos posteriores (validação temporal) e, se possível, capaz de predizer outros desfechos diferentes daquele para o qual foi desenvolvido e em diferentes cenários (validação ampla ou expandida). ^449^

 Vale ressaltar que modelos prognósticos sem validação externa, mesmo que desenvolvidos de maneira adequada, são considerados de pouca utilidade e nível baixo de sustentação por evidências, não sendo recomendados para uso na prática diária. Geralmente, o modelo prognóstico tem melhor desempenho no conjunto de dados que deu origem ao modelo do que com os novos dados em análises de validação. ^449^

 Em 2006, Rassi Jr *et al* . ^408^ desenvolveram e validaram um escore de risco para predizer morte por todas as causas na CCDC. Na coorte original envolvendo 424 pacientes ambulatoriais seguidos em média por 7,9 anos, a mortalidade total foi de 31% (130/424), sendo 87% (113/130) do total de óbitos por causas cardiovasculares e 62% (81/130) devidos a morte súbita cardíaca. Na coorte de validação externa (153 pacientes), a taxa de mortalidade total foi de 23% (35/153) durante seguimento médio de 7,7 anos, com a maioria dos óbitos (57%) também ocorrendo subitamente. 

**Figura 7.2:**
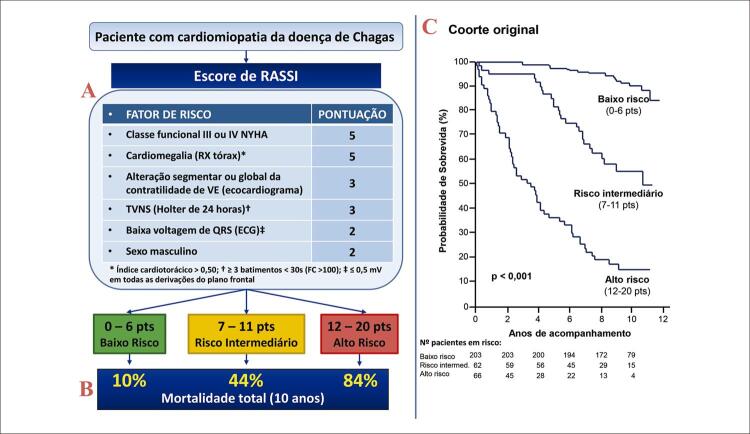
– Escore de RASSI. (A) Marcadores de risco e pontuação; (B) Morte total em 10 anos nos subgrupos de risco baixo, intermediário e alto; (C) Curvas atuariais de Kaplan-Meier. pts: pontos; TVNS: taquicardia ventricular não sustentada

 A análise multivariada identificou seis preditores independentes de mortalidade, sendo atribuídos a cada um deles pontos correspondentes à sua força de associação com o desfecho em questão (mortalidade geral) a partir de valores baseados no coeficiente beta de regressão do modelo de Cox ( Figura 7.2A ). Com base na soma total de pontos para cada paciente, os indivíduos foram classificados em subgrupos de risco baixo, intermediário e alto. A mortalidade total em 10 anos e as curvas atuariais de sobrevida desses três subgrupos são apresentadas nas Figuras 7.2B e 7.2C . A estatística C para o sistema de pontos foi de 0,84 na coorte de desenvolvimento e de 0,81 na coorte de validação. Com exceção do sexo masculino para morte cardiovascular e da baixa voltagem do QRS para morte súbita, que mostraram significância estatística limítrofe, as demais variáveis também foram fortes preditores de risco desses dois tipos específicos de óbitos. ^408^

 Além de apresentar inequívoco poder discriminatório, o escore de RASSI possui as seguintes vantagens: utiliza apenas seis variáveis fáceis de serem mensuradas ou coletadas e extraídas de exames complementares habitualmente disponíveis (ECG, radiografia de tórax, ECO bidimensional e Holter de 24 horas) e que fazem parte da investigação inicial obrigatória de pacientes com CCDC; avalia a função ventricular esquerda de maneira subjetiva, dispensando a medida da fração de ejeção pelo método de Simpson e valoriza as alterações tanto globais quanto segmentares da contratilidade miocárdica, essas últimas recentemente corroboradas como importantes preditores independentes de risco de eventos cardiovasculares por meio de análise criteriosa do banco de dados do estudo BENEFIT; ^342^ permite substituir a medida do ICT à radiografia de tórax pela medida do DDVE ao ECO, uma vez que foi observada, subsequentemente, boa correlação entre ICT > 0,50 e DDVE > 60 mm; ^337^ dispensa o uso de fórmulas ou calculadoras por se tratar de escore de memorização numericamente simples e factível; é capaz de predizer as três principais causas de óbitos: total, cardiovascular e súbito; ^408^ e, por fim, foi validado externamente em quatro coortes distintas, em momentos diferentes e por pesquisadores independentes. ^408 , 424 , 450 , 451^ Deve-se enfatizar que em duas dessas coortes, ^424 , 451^ o desfecho avaliado foi diferente daquele contemplado na publicação original (morte total) e, mesmo assim, o escore de RASSI mostrou resultados bastante reprodutíveis ( Tabela 7.1 ). 

**Tabela 7.1:** – Escore de RASSI: resultados na coorte original (Hospital São Salvador, Goiânia) e validação externa em quatro coortes distintas.

Autor	Período do estudo	Local do estudo	Nº de pacientes	Desfecho	% Desfecho (5 anos)			% Desfecho (10 anos)			Estatística C
					baixo risco (0-6 pts)	risco intermediário (7-11 pts)	alto risco (12-20 pts)	baixo risco (0-6 pts)	risco intermediário (7-11 pts)	alto risco (12-20 pts)	
Rassi A Jr. et al. ^3^	1986-1991	Hospital São Salvador (Goiânia)	331*	Morte Total	2 (0-5)	18 (8-28)	63 (51-75)	10 (5-14)	44 (31-57)	84 (74-93)	0,84 (0,79-0,89) ^†^
Rassi A Jr. et al. ^3^	1990-2001	Hospital Evandro Chagas (RJ)	153	Morte total	0	15 (1-28)	53 (31-75)	9 (2-16)	37 (16-59)	85 (63-100)	0,81 (0,72-0,90) ^†^
Rocha MOC & Ribeiro AL ^6^	1998-2006	Universidade Federal de Minas Gerais	158	Morte total	3 (1-7)	10 (4-22)	67 (30-90)	ND	ND	ND	0,84 (0,72-0,96)
Benchimol Barbosa PR et al. ^7^	1995-2003	Hospital Universitário Pedro Ernesto (RJ)	100	Morte cardíaca ou TV ^§ //^	4 (1-11) ^¶^	42 (18-83) ^¶^	50 (6-100) ^¶^	28 (18-43)	58 (29-100)	75 (15-100)	0,79 (0,70-0,88) ^†^
				Morte cardíaca ^#^	ND	ND	ND	ND	ND	ND	0,81 (0,69-0,93) ^†^
Senra T et al. ^8^	2001-2011	Instituto do Coração - INCOR (SP)	130	Morte total, transplante cardíaco, terapia apropriada do CDI ou recuperado PC ^//^	16	42	76	ND	ND	ND	ND
				Morte total ^#^	11	33	57,5	ND	ND	ND	ND

Os números entre parênteses correspondem ao intervalo de confiança de 95%. *modelo multivariado aplicado a 331 pacientes da coorte original com 424 pacientes (pacientes com dados faltantes foram excluídos); †referente a 10 anos; §definida como 3 ou mais batimentos sucessivos; //desfecho primário; ¶desfecho aos 50 meses; #desfecho secundário; CDI - cardioversor-desfibrilador implantável; N: número; ND: não disponível; PC: parada cardiorrespiratória; TV: taquicardia ventricular.

 A robustez do escore de RASSI, particularmente no que diz respeito à acurácia de sua estratificação em subgrupos de risco, é respaldada por resultados de investigações recentes em diferentes contextos, demonstrando, por exemplo, haver forte correlação positiva dos níveis de risco com o grau de disautonomia cardíaca, ^441 , 452^ com a presença e extensão da fibrose miocárdica à RMC detectada pela técnica do realce tardio ^424 , 428 , 453^ ou do mapeamento T1, ^425^ essa última avaliando o componente intersticial da fibrose miocárdica, e ainda com a indução de taquiarritmias ventriculares sustentadas ao EEF. ^454^

 Em outro estudo, avaliando pacientes que realizaram teste cardiopulmonar de esforço, a adição do escore de RASSI ao limiar anaeróbio aumentou a área sob a curva ROC de 0,706 para 0,800, tendo óbito por todas as causas como desfecho primário à análise de regressão logística. ^455^

 Ao se investigarem a prevalência e o valor prognóstico da dissincronia ventricular ao ECO e do escore de RASSI em pacientes com CCDC, tendo como desfecho a combinação de morte total e hospitalização, apenas o escore de RASSI foi capaz de predizer os eventos combinados em análise multivariada (OR = 1,19; IC 95%: 1,02-1,40; p = 0,01). ^456^

 Em pacientes com IC e FEVE < 45%, ao se avaliar o valor prognóstico de variáveis obtidas no teste cardiopulmonar de esforço juntamente com outras variáveis, apenas a inclinação VE/VCO _2_ aumentada (HR = 2,80; IC 95%: 1,30-5,80; p = 0,001, com ponto de corte de 32,5) e o escore de RASSI (HR = 1,28; IC 95%: 1,10-1,48; p = 0,001) estiveram associados a maior mortalidade em análise multivariada após seguimento médio de 32 meses. ^457^

**Figura 7.3:**
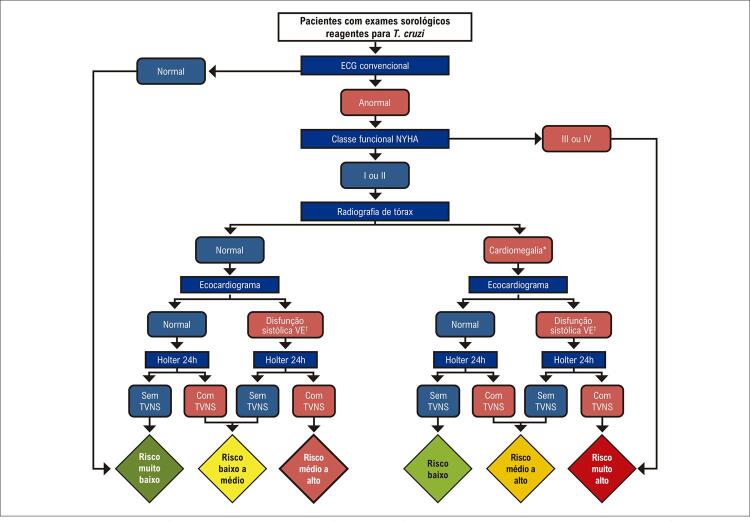
– Algoritmo de estratificação de risco na doença de Chagas. TVNS: taquicardia ventricular não sustentada; VE: ventrículo esquerdo. *pode ser substituída por diâmetro diastólico VE > 60 mm ao ecocardiograma; †global ou segmentar. Adaptado de Rassi A Jr, Rassi A, Rassi SG. Predictors of mortality in chronic Chagas disease. Circulation. 2007;115:1101-8. ^458^

 Em revisão sistemática de 12 estudos (1985 a 2006), ^458^ que utilizou análise multivariada para melhor avaliação do prognóstico na CCDC, englobando aproximadamente 4.300 pacientes, um enfoque mais detalhado dessas variáveis demonstrou que os preditores mais consistentes e relevantes de mortalidade total, morte súbita cardíaca ou morte cardiovascular foram classe funcional III ou IV da *New York Heart Association* (NYHA), cardiomegalia na radiografia de tórax, disfunção ventricular esquerda avaliada por ECO ou cineventriculografia, além de TVNS ao Holter de 24 horas. Utilizando essas quatro variáveis de forma integrada, é possível elaborar um algoritmo capaz de estratificar o risco de óbito de pacientes com DC de maneira simplificada e lógica por meio de parâmetros clínicos e métodos complementares disponíveis na maioria dos serviços de atendimento cardiológico em nosso meio ( Figura 7.3 ). 

 A presença de classe funcional III ou IV da NYHA, *per se* , identifica casos de alto risco, uma vez que praticamente todos esses pacientes apresentam disfunção ventricular sistólica ao ECO e TVNS ao Holter. Já a combinação de disfunção ventricular com TVNS, independentemente da classe funcional, identifica grupo com risco cerca de 15 vezes maior quando comparado a pacientes nos quais essas duas anormalidades estão ausentes. ^458^

 Apesar de o escore de RASSI apresentar base teórica solidamente estabelecida na literatura como preditor independente de eventos fatais, validado externamente em múltiplos estudos, o mesmo ainda é pouco utilizado no dia a dia. Talvez um dos motivos possíveis seja a baixa disponibilidade no SUS brasileiro, fora do ambiente dos hospitais universitários, dos métodos diagnósticos simples usados no cálculo do escore, como o ECO e o Holter de 24 horas. Esta diretriz, conforme exposto em outros capítulos, ao recomendar fortemente a aplicação do escore como principal método de estratificação de risco em todos os pacientes tão logo se confirme o diagnóstico da cardiomiopatia, a exemplo do que já estabelecem outros consensos de sociedades internacionais, ^459^ espera sanar tal deficiência. 

 Além disso, apesar de projetar o risco de óbito a longo prazo em condições de prognóstico bastante heterogêneo, a utilidade do escore de RASSI em guiar a conduta clínica e terapêutica subsequente ainda resta por ser determinada. É razoável considerar que a valiosa informação do risco, assim providenciada pelo escore para pacientes e seus médicos, poderá orientar estratégias de acompanhamento e, possivelmente, de tratamento. Vale ressaltar que se encontra em fase de conclusão o ECR multicêntrico brasileiro CHAGASICS ( *CHronic use of Amiodarone aGAinSt Implantable Cardioverter-defibrillator* ), comparando amiodarona *versus* desfibrilador na redução de mortalidade total como estratégia de prevenção primária, tendo como critérios de inclusão a presença de pelo menos um episódio de TVNS ao Holter de 24 horas e escore de RASSI ≥ 10 pontos. ^460^

 Ademais, é plausível especular que pacientes de baixo risco (mortalidade em 10 anos de 10%, semelhante à do grupo de baixo risco pelo escore de Framingham) possam se submeter a revisões clínicas anuais, ao passo que os de risco intermediário ou alto devam fazer revisões mais amiúde (a cada 3 ou 6 meses). 

 Outros estudos acerca do prognóstico na CCDC focaram diferentes marcadores de risco, como redução da FEVE, ^461 - 463^ disfunção sistólica do VD (índice de Tei), ^350^ disfunção diastólica do VE (relação E/e’), ^416^ aumento de volume do átrio esquerdo, ^415^ alterações nos índices de deformação miocárdica, ^412 , 464^ disfunção parassimpática e simpática, ^207 , 465^ alterações específicas no ECG, ^303^ variabilidade da amplitude da onda T, ^466^ desvio do eixo da onda T, ^467^ dispersão do intervalo QT, ^463 , 468^ alterações no ECG de alta resolução (turbulência espectral e QRS filtrado), ^436 , 451^ diminuição da VFC, ^451^ aumento da duração do complexo QRS, ^469^ diminuição do VO _2_ pico, ^461^ diminuição do tempo de exercício ^470^ e aumento dos valores plasmáticos dos peptídeos natriuréticos tipo B (BNP e NT-proBNP) ^469 , 471^ entre outros. ^472^ Tais fatores e variáveis, quando analisados por meio de modelos multivariados ou transformados em escores de risco, associam-se a pior prognóstico. Além disso, contribuem para trazer informações sobre os mecanismos relacionados à progressão da doença e desvendar aspectos menos explorados da sua complexa prognosticação. 

 Entretanto, os estudos acima são bastante heterogêneos, sendo imperativo reconhecer limitações a essas abordagens. ^473 , 474^ As seguintes limitações afetam sua aplicabilidade: uso de variáveis de mensuração difícil ou trabalhosa, não padronizadas e de baixa reprodutibilidade, muitas vezes extraídas de exames complementares de acesso restrito ou não disponíveis na prática comum; inclusão de número inadequadamente reduzido de pacientes ou de desfechos; não inclusão de todas as variáveis reconhecidamente associadas a um pior prognóstico na maioria desses modelos (aquelas 4 citadas anteriormente); e, particularmente, ausência de validação externa. ^473^

 Assim, os escores propostos não estão prontos para serem utilizados na assistência médica de rotina, já que, na quase totalidade dos estudos, carece-se de validação externa e independente. ^473 , 474^ Infelizmente, parece haver número crescente de publicações tentando desenvolver novos modelos de risco em vez de validar ou aperfeiçoar modelos existentes. ^474^

 Um estudo recente utilizou os dados da coorte NIH SaMi-Trop ^475^ e desenvolveu um escore simplificado para uso em regiões endêmicas sem acesso à propedêutica cardiológica além do ECG. O escore incluiu dados clínicos e eletrocardiográficos, além da dosagem do NT-proBNP, para a predição do risco de morte em 2 anos em pacientes com CCDC. ^469^ Cinco preditores independentes de óbito foram identificados, dando-se pontos aos mesmos, da seguinte forma: idade (10 pontos por década); classe funcional da NYHA superior a I (15 pontos); FC ≥ 80 batimentos/min (20 pontos); duração do QRS ≥ 150ms (15 pontos); e NT-proBNP anormal ajustado pela idade (55 pontos). Os pacientes foram então classificados em três categorias de risco (baixo, < 50 pontos; intermediário, entre 50 e 100 pontos; e alto, > 100 pontos). A validação externa foi realizada aplicando-se o escore a outra população independente com DC. Após 2 anos de seguimento, na coorte de desenvolvimento, 110 pacientes morreram, com uma taxa de mortalidade global de 3,5 mortes por 100 pessoas-ano. As taxas de mortalidade observadas nos grupos de risco baixo, intermediário e alto foram 0%, 3,6% e 32,7%, respectivamente, na coorte de derivação e 3,2%, 8,7% e 19,1%, respectivamente, na coorte de validação. A discriminação do escore foi boa na coorte de desenvolvimento (estatística C: 0,82) e na coorte de validação (estatística C: 0,71). ^469^ As principais limitações do escore são a utilização da dosagem de NT-proBNP, que não é habitualmente disponível na APS, e a ausência de validação externa independente e extensiva, como já foi realizado para o escore de RASSI. 

 Em dois outros estudos, a simples identificação ^428^ ou a quantificação ^424^ de fibrose miocárdica pela técnica do realce tardio na RMC, seja como variável contínua (expressa em valor unitário de grama adicional) ou como variável dicotômica (utilizando ponto de corte de 12,3g), mostrou ser um importante preditor de risco para eventos cardiovasculares graves, como morte total, morte cardiovascular e ocorrência de taquiarritmias ventriculares sustentadas, independentemente da função ventricular e do escore de RASSI. Vale destacar que um dos estudos ^424^ possibilitou a comparação direta entre o valor prognóstico da quantidade de fibrose miocárdica e o escore de RASSI. 

 Utilizando mortalidade total como desfecho final (considerado desfecho secundário), após seguimento mediano de 5,4 anos, o poder de associação pelo escore de RASSI foi mais marcante do que o da fibrose miocárdica. Expressas como variáveis categóricas (risco baixo, intermediário e alto para o escore de RASSI e massa < 12,3 e ≥ 12,3 g para fibrose miocárdica), apenas o escore de RASSI esteve associado a pior prognóstico (HR: 1,24; IC 95%: 1,13-1,36; p < 0,001 *versus* HR: 1,33; IC 95%: 0,68-2,61; p = 0,406). Expressas como variáveis contínuas (escore de RASSI em pontos e fibrose miocárdica em gramas), ambas foram preditoras de risco, mas com maior relevância para o escore de RASSI (HR: 1,23; IC 95%: 1,12-1,35; p < 0,001 *versus* HR: 1,02; IC 95%: 1,00-1,04; p = 0,043), ou seja, para cada ponto adicional no escore de RASSI, o risco de óbito aumenta em 23%, enquanto para cada 1 grama adicional de fibrose miocárdica, esse aumento é de apenas 2%. A massa de fibrose, como variável dicotômica, apresentou estatística C de 0,709 (IC 95%: 0,618-0,793) na predição de óbito por qualquer causa, ao passo que, para o escore de RASSI, esse valor não foi informado. ^476^

 Outro resultado relevante deste estudo foi a capacidade do escore de RASSI e da fibrose miocárdica predizerem o desfecho combinado que incluiu óbito por qualquer causa, TC, morte súbita revertida, choque apropriado ou terapia antitaquicardia pelo CDI, um desfecho de menor importância do ponto de vista hierárquico como risco, mas que foi considerado o desfecho primário no estudo em questão. Apesar desses resultados expressivos com o escore de RASSI, ^477^ os autores concluíram apenas que a fibrose miocárdica poderia contribuir para uma melhor estratificação de risco e, possivelmente, guiar o tratamento de pacientes com CCDC. ^424^

 Como a RMC não faz parte do arsenal diagnóstico inicial de avaliação da CCDC e, provavelmente, não entrará para o rol dos exames cardiológicos de rotina devido a seu alto custo, indisponibilidade em muitos centros e algumas contraindicações relativas ou absolutas para sua realização (e.g. pacientes com claustrofobia, portadores de alguns tipos de órteses/próteses ou modelos mais antigos de MP, ou insuficiência renal quando exames de ressonância exigem injeção de gadolínio), o passo lógico seguinte será testar se o método é capaz de melhorar o desempenho de modelos de estratificação de risco já existentes por meio de novas técnicas estatísticas, como a Tabela de reclassificação, o índice de reclassificação líquida (NRI) e a melhora da discriminação integrada (IDI). ^478^

 De acordo com a prevalência dos grupos de risco no escore de RASSI, sabe-se que, se o mesmo for aplicado a 1.000 pacientes com cardiomiopatia, 610 serão classificados como de baixo risco para óbito total, 190 como de risco intermediário e 200 como de alto risco. Com taxas de óbito de 10%, 44% e 84% em 10 anos, respectivamente, para os três subgrupos, ^408^ ao final de 10 anos teríamos 61 óbitos no grupo de baixo risco, 84 óbitos no grupo intermediário e 168 óbitos no grupo de alto risco. Assim, de um total de 313 óbitos durante os 10 anos de seguimento, apesar de a maioria (168 ou 54%) ocorrer no grupo de alto risco (o que é desejável em termos de estratificação de risco), ainda teríamos 145 óbitos nos grupos de risco baixo e intermediário. Para que um novo preditor de risco prove sua utilidade clínica, o ideal é que, uma vez adicionado ao escore de RASSI, ele seja capaz de identificar corretamente os pacientes de risco baixo e intermediário que irão a óbito ou, menos provavelmente, aqueles de alto risco que irão sobreviver. Talvez a fibrose miocárdica seja esse marcador, uma hipótese ainda a ser testada. 

 Ademais, não há na literatura dados informando se a mudança na pontuação do escore de RASSI (particularmente com diminuição no número de pontos) é capaz de avaliar e monitorar a eficácia de determinado tratamento e de melhorar o prognóstico dos pacientes. No entanto, como o escore integra seis variáveis, atribuindo-se de 0 a 20 pontos ao conjunto, e duas delas possuem maior chance de sofrer alterações (a classe funcional III/IV da NYHA e a presença de TVNS ao Holter), essa é outra investigação atraente a ser considerada. 

 Por fim, deve-se enfatizar que pacientes com idade > 70 anos, MP cardíaco artificial, TVS ou FV (documentadas), por apresentarem, *a priori* , risco de óbito elevado, bem como pacientes com doença isquêmica, hipertensiva ou valvular associada, para evitar confusão com óbitos não relacionados à CCDC, foram excluídos do cálculo e padronização do escore de RASSI. ^408^

 O valor prognóstico do EEF em pacientes com CCDC ainda não está bem estabelecido. No tocante à prevenção primária de morte súbita, os dados disponíveis sugerem que o EEF não tenha utilidade prognóstica em pacientes com EV isoladas ou TVNS, desde que a função sistólica de VE seja normal. Em estudo incluindo 72 pacientes com função de VE preservada (fração de ejeção média de 0,60) e 400 a 1.200 EV/hora ao Holter (35% com TVNS), a estimulação ventricular programada não induziu TVS em nenhum dos pacientes. ^479^ Durante seguimento médio de 36 meses, apenas 1 dos 72 pacientes apresentou TVS espontânea. 

 Posteriormente, outro estudo ^480^ avaliou o valor prognóstico da indução de TVS em resposta à estimulação ventricular programada em 78 pacientes com TVNS ao Holter (FEVE média de 0,47 ± 0,18) e sem história clínica de arritmias sustentadas. TVS monomórfica foi induzida em 25 pacientes (32%), todos tratados com fármacos antiarrítmicos da classe III, a maioria com amiodarona, e apenas um com sotalol. Após acompanhamento médio de 56 meses, as probabilidades de ocorrência de morte cardíaca e de eventos combinados (morte cardíaca, TVS espontânea ou recorrência de síncope) foram 2,2 e 2,6 vezes maiores (p < 0,05), respectivamente, nos pacientes indutíveis em comparação aos não indutíveis. Por outro lado, a indução de TV polimórfica ou de FV não teve significado prognóstico, tratando-se, provavelmente, de resposta ventricular inespecífica ao teste. ^480^

 Quanto à prevenção secundária (pacientes com arritmias ventriculares sustentadas documentadas ou com morte súbita ressuscitada), alguns autores avaliaram a importância do EEF na estratificação de risco e escolha da terapia antiarrítmica, mas os dados disponíveis são limitados. 

 O maior estudo de natureza observacional ^481^ incluiu 115 pacientes apresentando TV sintomática (FEVE média de 0,49 ± 0,14), dos quais, 78 com TVS espontânea e 37 com TVNS espontânea e TVS induzida ao EEF. Após impregnação com antiarrítmicos da classe III de Vaughan-Williams (sotalol ou amiodarona), os pacientes foram divididos em três grupos, com base em suas respostas aos testes eletrofisiológicos. Os pacientes do grupo 1 não tinham TVS indutível, aqueles do grupo 2 tinham TVS indutível bem tolerada e aqueles do grupo 3 tinham TVS indutível, hemodinamicamente instável. Após seguimento médio de 52 meses, a taxa de mortalidade total foi significativamente maior no grupo 3 em comparação com os grupos 1 e 2 (69% *versus* 26% e 22%, respectivamente). ^481^

 Com base nesses resultados, embora o EEF seja capaz de identificar pacientes com maior risco de óbito ou que não respondem bem ao tratamento com fármacos antiarrítmicos, seu papel em guiar outros tipos de terapias, como, por exemplo, o implante de um CDI, permanece indefinido, o que torna o método de pouca utilidade para esse fim. 

## 8. Condutas Terapêuticas na Forma Indeterminada da Doença de Chagas 

 A FIDC constitui período latente que, em geral, se inicia logo após o término da fase aguda, podendo permanecer indefinidamente, ou seja, por toda a existência do indivíduo. Esse estágio da DC foi reconhecido desde os estudos primordiais por Carlos Chagas, ^229 , 304 , 482^ sendo depois classicamente ratificado, em 1985, ^301^ para definir a situação de um indivíduo cronicamente infectado pelo *T. cruzi* mas assintomático, com exame físico normal e sem alterações na radiografia de tórax, no ECG convencional e nos exames radiológicos contrastados de esôfago e cólon. 

 A clássica definição de FIDC não considera indivíduos com alterações eletrocardiográficas “inespecíficas”, ou seja, não definidoras de CCDC. ^483 , 484^ Nessa situação, orienta-se o uso do termo “sem cardiopatia aparente” para esses pacientes em específico. Da mesma forma, essa denominação clássica não se aplica a pacientes assintomáticos em relação ao sistema digestório, porém sem avaliação de esôfago e cólon por exames contrastados. 

 No quadro abaixo são listadas as alterações que em geral definem a presença de CCDC e aquelas que, isoladamente, não são suficientes para firmar esse diagnóstico, sendo consideradas “não definidoras”. Essas alterações “inespecíficas” devem ser interpretadas judiciosamente, levando em consideração o contexto clínico subjacente. ^328 , 485^ Por exemplo, mesmo a baixa voltagem do complexo QRS no plano frontal, que conota mau prognóstico pelo escore de RASSI, é também detectável em indivíduos enfisematosos ou obesos mórbidos ( Quadro 8.1 ). 

**Quadro 8.1:** – Alterações eletrocardiográficas da doença de Chagas

Definidoras de CCDC	Não definidoras (inespecíficas ^*^ )
Bradicardia sinusal ≤ 40 bpm	Bradicardia sinusal > 40 bpm
Extrassistolia ventricular polimórfica	Extrassistolia ventricular isolada
Bloqueio completo do ramo direito	Bloqueio incompleto do ramo direito
Alteração primária da repolarização ventricular	Alteração secundária da repolarização ventricular
Bloqueio atrioventricular de 2º e 3º graus	Bloqueio atrioventricular de 1º grau
Bloqueio completo do ramo esquerdo	Bloqueio do ramo esquerdo de 1º grau
Zona eletricamente inativa	Desvio de eixo elétrico médio de QRS para esquerda
Disfunção do nó sinusal	Arritmia sinusal (não respiratória)
Taquicardia ventricular não sustentada e sustentada	Taquicardia sinusal
Fibrilação atrial	Bloqueio divisional anterossuperior esquerdo
Flutter atrial	Baixa voltagem de QRS
Fibrilação ventricular	Marca-passo atrial migratório

*Considera-se em geral que as alterações “inespecíficas” não firmam o diagnóstico de cardiopatia crônica da doença de Chagas (CCDC) quando isoladas, isso é, são únicas. Mas é plausível considerar que, quando se aglutinam, tornam o diagnóstico mais provável. Por exemplo, extrassistolia ventricular em associação a baixa voltagem de QRS e ainda distúrbio de condução AV ou em ramos (mesmo sem bloqueio completo) deve sinalizar como mais sugestivo do diagnóstico de CCDC. Há, ainda, margem para situações individuais de incerteza em zonas cinzentas de alterações de ECG, que devem ser dirimidas pelo médico de forma judiciosa. Adaptado de Biolo et al. ^485^

 Também é necessário reconhecer que o ECG normal, embora constitua critério definidor da FIDC, não é indicador fidedigno absoluto da ausência de acometimento cardíaco. Assim, quando se aprofunda a investigação com uso de outros métodos propedêuticos complementares, como o ECO, ^312 , 313 , 486 - 489^ o teste de esforço ergométrico ou mesmo cardiopulmonar, ^490 , 491^ o Holter de 24 horas, ^413 , 492 - 496^ as provas autonômicas não invasivas, ^236 , 405 , 438 , 497 - 499^ a cintilografia cardíaca, ^183 , 345 , 500 , 501^ os estudos hemodinâmicos e cineangiográficos, ^502^ a RMC ^311 , 453 , 503 , 504^ e até mesmo a biópsia endomiocárdica, ^505^ um número substancial desses pacientes com ECG normal apresenta anormalidades em alguns desses exames, muitas vezes de pequena monta ou baixa intensidade e frequência isolada. 

 Tais anormalidades, em sua maioria, com pouca ou nenhuma repercussão clínica, podem ser ocasionalmente encontradas em indivíduos saudáveis, sem infecção pelo *T. cruzi* . ^506 - 508^ Dessa forma, não obstante algum criticismo embasado em estudos que evidenciaram tais alterações em alguns dos indivíduos, o conceito de FIDC permanece vigente, ^299^ sendo prático e de ampla aplicabilidade. ^300^ Proposta para modificação dos critérios - incluindo a de substituição da radiografia de tórax por um ECO transtorácico em repouso - não teve maior receptividade. ^306^

 Por outro lado, embora haja trabalhos que demonstrem que essas alterações não impactam na progressão para a CCDC, ^211 , 509^ respaldando, assim, a noção de que são desprovidas de conotação prognóstica, os resultados da maioria desses estudos ainda precisam ser melhor avaliados em seguimento de longo prazo, não existindo prova definitiva de que não constituam potenciais gatilhos para ocorrência de futuros eventos cardiovasculares. 

 Infelizmente, a despeito de décadas de pesquisa, ainda não estão totalmente esclarecidos os fatores que levam cerca de 30% dos indivíduos na FIDC a desenvolverem a CCDC. ^176^ Muitos fatores estão envolvidos no risco de progressão da FIDC para a CCDC, tais como: idade; sexo masculino; origem geográfica; intensidade da carga parasitária; cepa do *T. cruzi* e suas “ *discrete typing units* ” (TcI–TcVI e Tc-bat); aspectos genéticos do hospedeiro; gravidade da infecção aguda inicial relacionada com a via de transmissão; exposição à reinfecção pelo parasito em áreas com transmissão vetorial sustentada; estado nutricional e presença de comorbidades; contexto social; qualidade de vida dos indivíduos com DC; e ausência de tratamento antiparasitário. ^247 , 297 , 510 - 517^

 A despeito do pouco conhecimento sobre a história natural da DC, é importante enfatizar que a FIDC tem geralmente bom prognóstico, ^509 , 518 , 519^ sendo a própria mortalidade superponível à da população geral não infectada, enquanto o ECG for normal. ^404^ O indivíduo com a FIDC pode permanecer por muitas décadas nessa condição, ^299 , 300^ sendo que a realização anual ou mesmo bianual do ECG, de maneira seriada, pode detectar a evolução para CCDC. ^2^

 Alterações eletrocardiográficas podem surgir no seguimento, em porcentagens variáveis, porém sem correspondente direto com a FEVE, que costuma permanecer inalterada. ^211^ As taxas anuais de progressão para CCDC, a partir da FIDC, variam de 0,3% a 10,3%, com média de 1,9%. ^297^ Na FIDC, a presença de ECO alterado por dissinergias regionais, mesmo em vigência de função ventricular sistólica global preservada, pode significar risco para eventos clínicos, como BAVT, AVC, taquiarritmias ventriculares e/ou IC, traduzindo pior prognóstico quando comparado a indivíduos na FIDC com ECO normal. ^341 , 418^

 O bom prognóstico dos pacientes com a FIDC foi relatado em vários estudos longitudinais, concluindo que as taxas de mortalidade são similares entre indivíduos com a FIDC e controles não infectados pelo *T. cruzi* na mesma faixa etária. ^299 , 404 , 519^ A incidência anual de morte súbita entre os indivíduos com DC e ECG normal é baixa e se assemelha à da população sem DC, ^520^ sendo essa uma complicação rara, que incide de igual forma na população geral e, portanto, sua causa não deve ser atribuída à DC. 

 Em relação ao tratamento com medicamentos tripanocidas na fase crônica da DC, a FIDC constitui uma das principais indicações, ^8 , 60^ sendo que pacientes adultos jovens tratados etiologicamente progridem menos para CCDC, em comparação aos não tratados. ^320 - 323 , 521^

 O seguimento de indivíduos com FIDC deve ser mantido em nível de APS, sendo recomendada a realização anual ou bianual do ECG, uma vez que algumas alterações eletrocardiográficas têm caráter evolutivo e são prioritárias como definidoras de CCDC. ^522^ No caso particular de paciente com ECG normal e ECO evidenciando alterações segmentares da contração ventricular, esse indivíduo deve receber a mesma abordagem propedêutica daquele que apresenta ECG definidor de CCDC. 

 Pacientes com FIDC podem apresentar comorbidades que são mais frequentes à medida que essa população envelhece. ^523^ A questão da relação entre envelhecimento e comorbidades nos pacientes com FIDC parece ser independente da própria presença de DC. ^524^ Porém, pacientes idosos com a FIDC constituem grupo populacional particularmente vulnerável em relação aos efeitos prejudiciais de doenças crônicas degenerativas. ^525^ Entre as comorbidades cardiovasculares, predomina a hipertensão arterial sistêmica (HAS) e, menos frequentemente, a doença arterial coronariana. ^526^ O monitoramento e o tratamento dessas comorbidades, associadas a dislipidemia e diabetes *mellitus* , devem ser feitos de forma individualizada. O controle desses agravos é fundamental na prevenção secundária da CCDC. ^527 , 528^

 A conduta médica geral frente a indivíduo cronicamente infectado pelo *T. cruzi* (comprovado por pelo menos duas sorologias positivas com técnicas laboratoriais distintas) deve ser, a princípio, conservadora, com objetivo de caracterizar-se a FIDC e estabelecerem-se as seguintes recomendações: 1) Na ausência de sintomas cardiovasculares e digestivos (particularmente, disfagia e constipação) e sendo o exame físico e o ECG (de preferência com registro de 30seg em derivação única) normais, não há necessidade de exames adicionais, dispensando-se os exames radiológicos de tórax, esôfago e cólon; 2) Devem-se repetir a anamnese dirigida, o exame físico e o ECG anualmente ou bianualmente; 3) Não deve ser instituída restrição para exercícios físicos (mesmo competitivos); 4) Não se deve implementar restrição profissional, inclusive para condução de veículos coletivos; e 5) O apoio psicológico é indispensável, explicitando-se as noções prognósticas favoráveis, que norteiam essas condutas médicas mais conservadoras. 

 A realização anual ou bianual de ECG em pacientes com FIDC tem recomendação forte, com nível de evidência B. Reiterando o exposto em capítulo específico da diretriz, o tratamento tripanocida com benznidazol deve ser oferecido, como recomendação forte, nível de evidência B, aos indivíduos cursando com a FIDC até os 50 anos de idade. 

## 9. Tratamento Etiológico da Doença de Chagas

### 9.1. Introdução

 Assegurar acesso a tratamento etiológico antiparasitário (tripanocida) eficaz, eficiente e seguro para a infecção por *T. cruzi* persiste como um desafio crítico ao se analisarem os avanços ao longo dos últimos 50 anos. ^2 , 3 , 38 , 44 , 56 , 58 , 112 , 113 , 529^ É inequívoca a importância da realização dessa modalidade de tratamento da DC para tanto as pessoas acometidas, quanto, de modo mais amplo, suas famílias e comunidades. Trata-se de questão central para os sistemas nacionais de saúde e barreiras devem ser superadas para que todos os pacientes possam ter acesso a diagnóstico e tratamento adequados. ^2 , 5^ Esse dilema ético depende da atuação mais proativa de gestores, profissionais de saúde (particularmente profissionais da Medicina, incluindo a Cardiologia como especialidade), movimentos sociais e todas as demais pessoas interessadas. 

 A DC insere-se no extenso grupo de DTN, em que falhas críticas da ciência, do ambiente mercadológico e da saúde pública, tornam esse desafio ainda maior. ^49 , 52 , 530^ Apesar dos avanços, esses ainda têm sido insuficientes para uma resposta consistente em saúde pública, com vista ao controle da doença na rede de serviços locais de saúde nos diversos países. ^44 , 54 , 94 , 531 - 533^ Em muitos cenários locorregionais, métodos para diagnóstico complementar e medicamentos para tratamento não estão disponíveis e as populações locais não estão suficientemente informadas de sua factibilidade. 

 Nessas últimas cinco décadas, ainda se registra gritante limitação de opções para tratamento etiológico, havendo disponibilidade apenas de dois medicamentos comprovadamente eficazes, o benznidazol (1971) e o nifurtimox (1965). ^1 , 2 , 5 , 8 , 44 , 54 , 377 , 534 - 536^ Em linhas gerais, há evidências contundentes de que ambos são efetivos em reduzir a duração e a gravidade clínica da doença, ao possibilitarem a eliminação de parasitos quando do tratamento precoce na história natural da doença, ^1 , 2 , 8 , 44 , 46 , 60 , 377 , 379^ com ganhos potenciais em termos da qualidade de vida mediante prevenção de eventuais limitações de capacidade física. ^43 , 537^

 Em geral, o benznidazol ainda é o mais eficaz tripanocida, com sistemática comprovação em ensaios clínicos que o utilizaram como comparador para avaliação de novos fármacos. Entretanto, ainda há críticas lacunas para o desenvolvimento de novas opções terapêuticas com menor toxicidade, visando a melhorar o perfil de segurança e o acesso ao tratamento. Reconhece-se o caráter estratégico de desenvolvimento de novos estudos para avaliar não apenas o uso de terapias combinadas, mas também de esquemas mais curtos temporalmente, com doses fixas e menores, em consonância com a busca de melhores e mais confiáveis parâmetros clínicos e biomarcadores laboratoriais, para se avaliar a eficácia do tratamento. ^536^ Entretanto, os estudos disponíveis até o momento não permitem recomendar esquemas terapêuticos diferentes dos classicamente estabelecidos. Ressalte-se que, na realidade brasileira do SUS, o benznidazol representa o fármaco mais disponível e utilizado, ainda com limitada operacionalização frente à demanda esperada. ^2 , 54 , 113^

 O tratamento etiológico adequado é reconhecidamente custo-efetivo ^5 , 50 , 52 , 53^ e traz, como benefícios potenciais para a pessoa acometida, a redução da parasitemia, com impacto positivo na evolução clínica, como o impedimento da progressão para a forma cardíaca, a redução de complicações clínicas nas duas fases da doença e o aumento da expectativa de vida, além da melhora da capacidade física e da qualidade vital. ^2 , 8 , 32 , 41 , 43 , 53 , 318 , 322 , 323 , 537 - 540^

 Reconhece-se que, entre os principais desafios, insere-se a necessidade de que o tratamento etiológico esteja disponível e implementado nos sistemas locais de saúde ^2 , 8 , 112 , 113 , 533^ e de que a oferta desses medicamentos seja contínua, fato ainda limitado pelo número restrito de seus fornecedores e a baixa demanda pelos produtos nos próprios sistemas locais de saúde. ^56^ Torna-se, portanto, fundamental evitar oportunidades perdidas para o estabelecimento de diagnóstico e tratamento. Tendo em vista estar relacionada à pobreza e a contextos de grande vulnerabilidade social, reconhece-se também que a atenção integral às pessoas com DC potencialmente reduzirá inequidades em saúde, em particular nos territórios endêmicos. ^2 , 8 , 49 , 54 , 113^

 Este capítulo específico sobre tratamento etiológico fundamenta-se na análise de consensos, protocolos clínicos e diretrizes terapêuticas, que foram escritos e atualizados em diferentes contextos recentes. Representam estratégias relevantes que visam a contribuir com a ampliação do acesso a diagnóstico e tratamento, com base no apoio consubstanciado a decisões clínicas. ^2 , 41 , 49 , 56^ Os documentos referenciais apresentados a seguir estiveram fundamentados, em maior ou menor grau, nos procedimentos metodológicos do sistema GRADE, ^27^ adaptado para fins específicos destas diretrizes (ver capítulo relacionado). 

 Entre as diretrizes clínicas regionais, incluiu-se a análise do guia para diagnóstico e tratamento da DC ( *Guía para el diagnóstico y el tratamiento de la enfermedad de Chagas / Guidelines for the diagnosis and treatment of Chagas disease* ), publicado em 2019 pela OPAS/OMS. ^60^ Considerou-se ainda na revisão, a I Diretriz Latino-Americana para o Diagnóstico e Tratamento da Cardiopatia Chagásica, de 2011, coordenada pela SBC. ^1^

 Tendo em vista tratar-se de uma diretriz de base nacional e respeitando-se as especificidades das pactuações e organização do SUS, tomou-se como principal referência o Protocolo Clínico e Diretrizes Terapêuticas (PCDT) em DC, conduzido pela Comissão Nacional de Incorporação de Tecnologias no SUS (CONITEC), da Secretaria de Ciência, Tecnologia e Insumos Estratégicos do Ministério da Saúde. ^8^

 Adicionalmente, foi analisado o 2º Consenso Brasileiro em DC de 2015, um importante marco referencial, coordenado pela Secretaria de Vigilância em Saúde do Ministério da Saúde em parceria com a Sociedade Brasileira de Medicina Tropical, ^2^ na sequência do Consenso Brasileiro em DC de 2005. ^3^

### 9.2. Fármacos e Administração

 Dois compostos antiparasitários nitro-heterocíclicos estão disponíveis, com eficácia estabelecida para o tratamento etiológico da DC: benznidazol, um agente derivado nitroimidazólico, e o nifurtimox, um composto nitrofurânico. ^54 , 60 , 377 , 534 , 535^ Há pesquisas clínicas que incluíram outros fármacos sem eficácia comprovada, por exemplo, alopurinol e antifúngicos azólicos (por reposicionamento de moléculas), ^8^ não fazendo, entretanto, parte do escopo da presente diretriz. 

 Nesse sentido, estudos têm sido conduzidos nos últimos 7 anos para avaliação da eficácia e segurança de monoterapia ou terapias em combinação de benznidazol com outros agentes, como posaconazol ou fosravuconazol. Tais pesquisas foram conduzidas em indivíduos infectados com *T. cruzi* , mas sem evidência de dano em órgão-alvo, e tiveram resultados limitados apenas a aspectos parasitológicos por meio da avaliação via qPCR de longo prazo (12 meses). ^385 , 541 , 542^ Apesar dos resultados desapontadores com os novos fármacos, os estudos comparativos reforçaram o papel relevante do benznidazol no tratamento da doença. 

 Revisão recente identificou 109 estudos epidemiológicos publicados após 1997 sobre tratamento etiológico da DC (31 observacionais e 78 de intervenção), incluindo 23.116 indivíduos, com grande heterogeneidade não apenas do manejo clínico para tratamento etiológico, assim como no delineamento e na condução dos estudos, o que limita as evidências disponíveis. ^543^

 Em linhas gerais, o grau de recomendação ‘ponderado’ ou ‘condicional’ estabelecido pela OPAS para o uso de benznidazol e nifurtimox, principalmente em casos com DC crônica, justifica-se pelo limitado nível de certeza do corpo de evidências sobre resultados de eficácia, oriundos da escassez de ECR nessa área. ^60^ Em grande parte, as evidências em DC devem ser aduzidas por serem fundamentadas em tratamento focado na infecção por *T. cruzi* . Diante da comprovação da ação tripanocida, na ausência de estudos experimentais aleatorizados utilizando desfechos clínicos relevantes, evidências por meio de estudos menos robustos, observacionais e de boa qualidade devem ser consideradas. 

 Ademais, dentro do princípio da assimetria, a magnitude de um eventual dano do tratamento, caso ocorra, é significativamente menor do que o benefício associado, particularmente com seguimento qualificado. Portanto, justifica-se o tratamento etiológico para DC em número considerável dos casos. 

 Na perspectiva dos gestores, o tratamento com benznidazol, portanto, pode ser adotado como política de saúde em contextos específicos, levando em consideração o balanço entre benefícios e riscos e prioridades em saúde. Para profissionais de saúde, há a possibilidade de diferentes escolhas para a tomada de decisão, que deve ser sempre compartilhada e informada em relação às pessoas acometidas pela doença. Por fim, a maioria das pessoas acometidas, quando bem-informadas, teria elevada probabilidade de desejar receber a intervenção. 

 O benznidazol representa a primeira opção no contexto brasileiro, devido não apenas à maior experiência de uso, mas também ao perfil de eventos adversos e à disponibilidade, particularmente de apresentações pediátricas. ^2 , 8 , 54 , 113^ O uso do nifurtimox no Brasil é recomendado nos casos em que o benznidazol não foi tolerado, como na ocorrência de eventos adversos graves, e em algumas outras circunstâncias mais particularizadas e específicas. ^2 , 8 , 46^

 Em geral, o tratamento etiológico com quaisquer dos medicamentos anteriores não deve ser instituído de modo rotineiro e indiscriminado em mulheres em idade fértil que não estejam em uso regular de método anticoncepcional reconhecidamente eficaz. ^2 , 8 , 60^ De forma análoga, a indicação em casos com outras afecções graves (insuficiência hepática e renal) deve ser avaliada criteriosamente de modo individualizado, de acordo com a gravidade clínica. 

 O benznidazol encontra-se disponível como comprimidos de 100 mg e 50 mg (adultos) e de 12,5mg e 50mg (crianças). A absorção ocorre através do trato gastrointestinal, enquanto a excreção é predominantemente renal, com meia vida de 12 horas. ^1^ No Brasil, somente as apresentações de 100mg e 12,5mg estão disponíveis na rede do SUS. ^54 , 544^ O benznidazol foi aprovado em 2017 pela Agência Norte-Americana de Alimentos e Medicamentos (FDA) para tratamento da infecção por *T. cruzi* , fato que não foi suficiente para garantir o pleno acesso de pacientes ao medicamento naquele país. ^545^

 O Ministério da Saúde brasileiro adquire o benznidazol 100mg e o distribui às Secretarias Estaduais de Saúde mediante solicitação no Sistema de Informação de Insumos Estratégicos. O fluxo de distribuição para regionais de saúde e/ou municípios é estabelecido por cada secretaria, integrando ações da Assistência Farmacêutica, Vigilância Epidemiológica e Atenção Básica. ^2 , 54^ Por outro lado, a distribuição do benznidazol 12,5 mg é centralizada no Ministério da Saúde, considerando o limitado registro de casos pediátricos no país. ^2 , 54^

 Reconhece-se que o processo de definição da dose apropriada de benznidazol para garantir eficácia e tolerabilidade foi estabelecido por uma abordagem de tentativa e erro. ^546^ Em adultos com DC crônica, o benznidazol é utilizado por via oral na dose de 5mg/kg/dia dividida em duas ou três tomadas, durante 60 dias, com dose máxima recomendada de 300mg/dia. Para casos de DC aguda, essa dose pode ser de até 10mg/kg/dia. Pessoas com peso acima de 60kg podem estender o esquema terapêutico para que se alcance a dose-alvo ideal, mantendo-se 300 mg como limite diário, com vistas a prevenir a ocorrência de eventos adversos. ^2 , 8^ Pode-se utilizar o esquema de 300mg de benznidazol pelo número de dias equivalente ao peso da pessoa, limitado ao total de 80 dias, mesmo que a pessoa possua mais de 80kg. ^1 , 2 , 8^ Esse esquema posológico, que parece ser melhor tolerado, foi proposto originalmente pelo Professor Anis Rassi ( *in memoriam* ) e adotado posteriormente na segunda metade da investigação com os cerca de 1.500 indivíduos arrolados no estudo BENEFIT, publicado em 2015. ^324^

 Em crianças, a dose utilizada pode variar de 5 a 10mg/kg/dia, dividida em duas tomadas diárias por 60 dias, com dose máxima de 300 mg/dia. Quando a dose diária ultrapassar os 300mg, recomenda-se estender o tempo de tratamento até alcançar a dose total calculada para 60 dias. ^1 , 2 , 8^ Há a possibilidade de uso da formulação pediátrica de 12,5mg em comprimidos solúveis, tendo a vantagem de poder ser utilizada para tratar desde recém-nascidos até crianças de 2 anos de idade. ^2 , 54^ A principal vantagem do comprimido de 50mg (não disponível no Brasil) é poder utilizá-lo para tratar o restante da população pediátrica, incluindo adolescentes e adultos jovens. ^544^

 Mais recentemente, os ensaios clínicos randomizados CHAGASAZOL, ^385^ STOP-CHAGAS ^542^ e E1224, ^541^ que não demonstraram efeito parasitológico de longo prazo com posaconazol ou fosravuconazol isoladamente, descreveram evidência superior a 85% de depuração parasitológica precoce (PCR negativo) após 2 a 4 semanas de tratamento com benznidazol isoladamente ou associado a posaconazol ou fosravuconazol, efeito que foi sustentado durante o seguimento de 12 meses. ^385 , 541 , 542^

 Posteriormente, foi publicado o ensaio clínico BENDITA, um estudo clínico duplo-cego, duplo simulado, de fase 2, multicêntrico e randomizado conduzido na Bolívia, que incluiu pessoas com 18 anos a 50 anos de idade com a FIDC. ^531^ Como resultados, evidenciou-se que o benznidazol induziu resposta antiparasitária eficaz (variando de 83% a 89%), independentemente da duração do tratamento (2 ou 4 semanas), dose diária (150 mg ou 300 mg) ou de combinação com fosravuconazol, tendo sido bem tolerado (3% de eventos adversos graves) em adultos com doença crônica. ^531^ Mesmo não sendo “definitivo”, esse estudo sugere o uso do benznidazol como padrão de tratamento e ressalta a necessidade de se avançar em novos estudos para utilização de esquemas encurtados ou com doses reduzidas de benznidazol. ^531^

 Esses achados ampliam as evidências de que o uso do benznidazol, nesses novos esquemas, poderia ampliar o acesso ao tratamento etiológico, assim como assegurar sua maior tolerabilidade. ^536^ Entretanto, há ainda a necessidade de se disponibilizar evidências mais contundentes para a futura adoção de um esquema terapêutico encurtado. Nesse sentido, estão em curso outros ensaios clínicos, como por exemplo: estudo BETTY - um ECR de não inferioridade do tratamento com benznidazol em curto prazo para reduzir a carga parasitária de *T. cruzi,* em mulheres em idade reprodutiva; ^547^ o estudo MULTIBENZ – um ECR multicêntrico de não inferioridade de fase II, para avaliação de eficácia e segurança de diferentes doses de benznidazol para tratamento da DC em fase crônica em adultos; ^548^ e o estudo TESEO - um ensaio clínico aberto, randomizado, prospectivo, de fase 2, para avaliação de segurança e eficácia de novos esquemas terapêuticos com benznidazol e nifurtimox, em adultos na fase crônica da DC, além de ampla avaliação com biomarcadores. ^549^

 O benznidazol tem sua eficácia demonstrada por vários estudos, mas tem limitações relacionadas à tolerabilidade, por sua relativamente elevada toxicidade, que pode levar à interrupção do tratamento antiparasitário em cerca de 10-25% dos casos. ^1 , 2 , 5 , 8 , 58 , 60 , 324 , 536 , 550 , 551^ A incidência média de eventos adversos associados ao uso de benznidazol é de cerca de 50%, sendo que manifestações cutâneas, sintomas gastrointestinais e distúrbios do sistema nervoso têm representado as razões mais comuns para interrupção do tratamento. ^2 , 60 , 324^

 Os eventos adversos dermatológicos são os mais frequentes, particularmente dermatite urticariforme (45%) e *rash* (30%), e geralmente não demandam a interrupção do tratamento por sua baixa intensidade. ^8^ A dermatite inicia-se já no final da primeira semana de tratamento, apresentando boa resposta ao tratamento com anti-histamínicos ou com pequenas doses orais de corticosteroides. ^2 , 58^ Podem ocorrer ainda intolerância gastrointestinal (13%), com náuseas, vômitos e diarreia, parestesias (10%) e artralgias (8%). ^8^ A frequência observada de eventos adversos ao benznidazol foi de 20,2% em crianças e adolescentes com DC na fase aguda, a partir de casuísticas amazônicas em focos de maior ocorrência de casos. Nesses relatos, as alterações dermatológicas ( *rash* , erupção urticariforme ou exantema heterogêneo descamativo e angioedema angioneurótico) foram as principais (72%), seguidas por alopecia (3%), distúrbios gastrointestinais (2%) e insônia (2%). ^279 , 291^

 A ocorrência de polineuropatia periférica com parestesias e dor em membros inferiores é mais comum em adultos e, em geral, inicia-se ao final do tratamento de 60 dias (particularmente após 50 dias), podendo ter importante impacto sobre a funcionalidade e qualidade de vida, já que pode permanecer por alguns meses, mesmo após a interrupção do tratamento e não responde bem a tratamento com anti-inflamatórios e polivitamínicos. Já a ocorrência de febre, adenomegalia e dor em orofaringe é sugestiva de depressão precoce da medula óssea e agranulocitose, um dos efeitos mais graves, apesar de raro, do benznidazol. Nesses casos, há desenvolvimento de leucopenia significativa às custas de segmentados (neutropenia febril), indicando a necessidade de interrupção imediata e proscrição definitiva do fármaco. Por esse efeito, está indicada a realização rotineira de hemograma 3 semanas após o início do tratamento. ^1 , 2 , 8 , 58^

 Em síntese, a despeito dos aspectos anteriormente mencionados, ressalta-se que o tratamento etiológico com benznidazol pode ser conduzido com segurança no contexto da APS. Protocolo da organização Médicos sem Fronteiras demonstrou resultados consistentes, pois até 89,8% das pessoas tratadas concluíram o tratamento, apesar de que 56,0% tivessem desenvolvido algum evento adverso. ^58^ O sucesso alcançado foi associado ao monitoramento próximo dos casos, o que fortaleceu a vigilância, mas também ao aconselhamento com informação qualificada e identificação oportuna de eventos adversos e seu manejo, que levou à menor taxa de abandono, ^58^ reforçando a importância da longitudinalidade do cuidado. ^44^

 No contexto da assistência farmacêutica, recomenda-se o protocolo de dispensação de benznidazol em intervalos de aproximadamente 7 dias, o que pode ampliar a segurança do uso por possibilitar um seguimento mais próximo e qualificado, com detecção e registro mais oportunos de eventos adversos associados. ^58^ Ressalta-se que, para além do tratamento etiológico, considerando o caráter crônico da DC, o acompanhamento farmacoterapêutico também possibilita o reconhecimento de eventos associados a outros medicamentos utilizados no manejo dos casos, além de melhorar a adesão e a qualidade de vida. ^5 , 43 , 44 , 536 , 552^

 Nos casos em que for registrada intolerância ao benznidazol, o nifurtimox poderá ser recomendado. Encontra-se disponível em comprimidos de 120mg (adultos) e de 30mg (crianças). ^1 , 2 , 8 , 534 , 535^ Em 2020, obteve aprovação da FDA/EUA para uso no tratamento da DC em crianças com menos de 18 anos de idade, ^534 , 535^ abrindo oportunidade para ampliar acesso diante das evidências disponíveis. ^5^

 A absorção do fármaco é gastrointestinal, com metabolização hepática via citocromo P450 e eliminação preferencial por via renal. ^1 , 534 , 535^ O nifurtimox não é disponibilizado pelo mercado farmacêutico do Brasil e o seu fornecimento tem sido regulado por meio de protocolo padronizado pela Secretaria de Vigilância em Saúde do Ministério da Saúde via OPAS, mediante demanda específica, em geral relacionada à suspeita ou confirmação de resistência ou intolerância ao benznidazol. ^2^

 Em adultos, é utilizado na dose de 10mg/kg/dia por via oral, em três tomadas diárias, durante 60 dias. Já em crianças, a dose preconizada é de 15 mg/kg/dia por via oral, também em três tomadas diárias, durante 60 dias. ^2 , 534 , 535^ O estudo CHICO, um ensaio clínico prospectivo, controlado para avaliar a eficácia e segurança de uma nova formulação pediátrica de nifurtimox em crianças com idades entre 0 e 17 anos com DC após 1 ano de tratamento, reiterou que o esquema de tratamento por 60 dias foi mais eficaz do que a mesma dosagem por 30 dias. ^553^

 Para o nifurtimox, a frequência média de eventos adversos é de aproximadamente 85%, sendo os mais frequentes a intolerância gastrointestinal, como anorexia e perda de peso (60%), eventos reumatológicos, como artralgias (35%), e acometimento dermatológico (15%). ^8^ Nos EUA, verificou-se que, na análise de 243 casos que iniciaram o tratamento, 222 (91,4%) relataram pelo menos um evento adverso (total de 1.155 eventos adversos, mediana de 4 por paciente). Os eventos adversos relatados incluíram as seguintes categorias: gastrointestinal (68,7%), neurológica (60,5%) e constitucionais (46,5%), sendo que os mais comumente relatados foram náusea (50,6%), anorexia (46,1%), perda de peso (35,0%), cefaleia (33,3%) e dor abdominal (23,1%). Pelo menos 90% dos pacientes de todas as faixas etárias do estudo (menor de 18 anos, 18 a 50 anos e maior de 50 anos) relataram eventos adversos. ^554^ De 1.042 eventos adversos com dados quanto à gravidade disponíveis, 680 (65,3%) foram leves, 254 (24,4%) moderados e 108 (10,4%) graves. Os eventos adversos graves mais frequentes foram: depressão (22,6%), neuropatia periférica (18,5%), parestesia (17,9%) e tontura/vertigem (17,2%). A proporção de pessoas com pelo menos um evento adverso grave foi maior entre os casos com mais de 50 anos (31,8%) comparativamente à queles de 18 a 50 anos (18,1%). ^554^

 Em seu guia de 2019, a OPAS considerou não haver diferenças substanciais, com base na análise comparativa de efeitos adversos, entre os dois fármacos por meio das evidências analisadas e da experiência do seu painel técnico. Entretanto, foram reconhecidos perfis específicos de eventos adversos predominantes, nifurtimox principalmente associado a perda de peso e efeitos adversos psiquiátricos, e benznidazol a reações cutâneas e neurológicas. ^60^ Nesse sentido, os eventos adversos e a toxicidade do nifurtimox destacam-se pela menor tolerância digestiva, refletida em anorexia, náuseas e vômitos, com perda de peso e distúrbios psiquiátricos mais frequentes em adultos. ^1 , 2 , 8 , 534 , 536 , 554^

 Para ambos os antiparasitários, torna-se fundamental garantir o monitoramento clínico do uso para avaliação e manejo oportuno desses eventos adversos, com ênfase em sua tolerabilidade. ^1 , 2 , 8 , 58 , 535 , 536^ O Quadro 9.1 resume os principais efeitos adversos do benznidazol e as condutas adequadas para cada situação. 

**Quadro 9.1:** – Efeitos adversos do benznidazol e condutas recomendadas para cada situação

BENZNIDAZOL						
Efeitos adversos	Aparecimento	Características	Localização	Intensidade	Conduta	Medidas complementares
Dermopatia por hipersensibilidade é a mais frequente. NÃO é dose-dependente e NÃO se relaciona com a infecção por *T. cruzi* . Recuperação sem sequelas	10º dia de tratamento, podendo ocorrer mais precoce ou mais tardiamente	Eritema polimorfo não bolhoso, pruriginoso, seguindo-se de descamação. Raramente ocorrem onicólise e edema angioneurótico Raros relatos de Síndrome de Stevens-Johnson	Focal (restrita à parte do tegumento) ou generalizada	Leve (geralmente focal)	Continuar o tratamento	Tratamento específico para desidratação e descamação cutânea Internação de acordo com o quadro clínico
				Moderada (focal ou generalizada)	Continuar o tratamento com associação de baixa dose de corticosteroide (prednisona). Interromper se houver piora	
				Grave, geralmente acompanhada de febre e linfoadenomegalia ou Síndrome de Stevens-Johnson	Interromper o tratamento. Utilizar corticosteroide	
Polineuropatia periférica. Dose-dependente, de regressão lenta (meses)	Final do tratamento	Dor e parestesia	Regiões plantares (mais frequente) e palmares	Leve a moderada	Interromper o tratamento	Tratamento geral para polineuropatia periférica
Ageusia (rara). Recuperação sem sequela	Final do tratamento	Perda total ou parcial do paladar	-	-	Interromper o tratamento	-
Depressão da medula óssea com recuperação sem sequela	Entre 20 ^o^ e 30 ^o^ dias de tratamento	Febre, adenomegalia e dor de garganta podem sinalizar precocemente leucopenia com neutropenia de variados graus, podendo chegar à agranulocitose	-	-	Interromper o tratamento	Tratamento geral para depressão da medula óssea
A intolerância digestiva (rara) é controlada com uso de medicação habitualmente utilizada para gastrite e úlceras pépticas. Acometimento hepático grave é raramente descrito. Acometimento renal não tem sido observado.						

Adaptado de 2º Consenso Brasileiro em DC, 2015 (Dias, 2016) ^2^ e Protocolo Clínico e Diretrizes Terapêuticas em DC, 2018 (Brasil, 2018). ^8^

### 9.3. Tratamento Etiológico de Indivíduos com Doença de Chagas 

 Como já expresso em outro capítulo desta diretriz, na história natural da DC, a maioria dos indivíduos com infecção estabelecida permanece assintomática ao longo de toda a vida. Na fase aguda, 90% dos casos por transmissão clássica vetorial evoluem de forma assintomática ou oligossintomática, sendo que, dos 10% que apresentam alguma evidência de síndrome clínica, menos da metade evolui com formas mais graves ou óbito. ^2 , 7 , 46 , 555^ Já em contextos com predominância da transmissão por via oral (surtos ou microepidemias familiares), em 75% a 100% dos casos, verifica-se síndrome clínica leve, como no caso de crianças, ou adoecimento evidente de síndrome febril prolongada. ^279 , 556^

 É oportuno salientar que as lesões orgânicas derivadas da infecção por *T. cruzi* na fase aguda dependem exclusivamente da presença do parasito, enquanto na fase crônica essas lesões são parcialmente explicadas pela persistência parasitária tissular e pelo grau de resposta imunológica ao parasito. ^1 , 5 , 38 , 46 , 323^

 A fase crônica da DC inclui a forma indeterminada (assintomática) e as formas cardíaca, digestiva e cardiodigestiva. ^2 , 46^ Na fase crônica, aproximadamente 60-70% dos casos permanecem assintomáticos enquanto 30-40% progridem para as formas clínicas da doença, em geral após vários anos, ^32 , 46 , 539^ com algumas complicações potencialmente graves, em particular aquelas de natureza cardiovascular, associadas a elevada carga de morbimortalidade. ^1 , 2 , 7 , 46 , 297 , 448^ O tratamento, quando indicado na fase crônica, tem como objetivo reduzir os níveis de parasitemia, prevenir o surgimento ou a progressão de lesões em órgãos-alvo, além de evitar a transmissão. ^1 , 2 , 297 , 318 , 322^

 A resposta comprovada em termos parasitológicos ao tratamento etiológico é variável e está na dependência de fatores que incluem: idade do caso no diagnóstico; fase e tempo de duração da doença; exames complementares utilizados para avaliação de eficácia terapêutica; tempo de seguimento após o tratamento; condições associadas; e susceptibilidade de diferentes linhagens (TcI a TcVI) de *T. cruzi* a medicamentos antiparasitários. ^38 , 248 , 379 , 538 , 550 , 557 , 558^ Esses aspectos reforçam a importância do seguimento de todos os casos, independentemente do local onde estejam sendo tratados na rede de serviços de saúde. 

 O tratamento etiológico da pessoa acometida pela DC deve, portanto, ser conduzido considerando-se o perfil do caso e a forma clínica da doença, conforme demonstrado no Quadro 9.2 . ^2 , 8^

**Quadro 9.2:** – Recomendações para tratamento etiológico da doença de Chagas, segundo fase da doença ou forma clínica e faixa etária.

Fase/forma da DC	Faixa etária	Tratamento etiológico
Aguda ou congênita	Todas as faixas etárias	1ª linha: benznidazol 2ª linha: nifurtimox
Crônica indeterminada ou digestiva	Crianças (≤ 12 anos) e adolescentes (13-18 anos)	1ª linha: benznidazol 2ª linha: nifurtimox
	Adultos < 50 anos	1ª linha: benznidazol Não usar nifurtimox
	Adultos ≥ 50 anos	Decisão compartilhada: possibilidade de tratamento, caso não haja contraindicações 1ª linha: benznidazol Não usar nifurtimox
Crônica cardíaca não avançada (estágio inicial: B1*)	Todas as faixas etárias	Decisão compartilhada: oferecer possibilidade de tratamento, caso não haja contraindicações 1ª linha: benznidazol Não usar nifurtimox
Crônica cardíaca ou digestiva (fase avançada)	Todas as faixas etárias	Não tratar

*Ver gradação dos estágios de cardiopatia em tabela 5.2 desta diretriz. Adaptado de Protocolo Clínico e Diretrizes Terapêuticas em DC, 2018 (Brasil, 2018). ^8^

### 9.4. Infecção Aguda

 O tratamento etiológico para todos os casos (crianças, adolescentes e adultos) na fase aguda da DC tem grau de recomendação ‘forte’, mesmo com nível de evidência B, de moderada qualidade em termos do benefício do efeito tripanocida. ^60^ Esse tratamento deve ser realizado o mais precocemente possível após o diagnóstico da infecção, independentemente do modo de transmissão do *T. cruzi* , tendo em vista os benefícios potenciais. ^1 , 2 , 5 , 8 , 32 , 60 , 555 , 556 , 559 - 562^

 Nessa fase, a despeito da evidência científica em nível moderado e da limitação da certeza quanto a desfechos clínicos da doença, o tratamento apresenta elevada eficácia, aumenta a probabilidade de negativação sorológica e/ou da parasitemia, além de melhorar a síndrome clínica potencialmente grave da fase aguda e, consequentemente, a princípio, prevenir a progressão para a forma crônica manifesta da doença pela redução de danos em órgãos específicos. ^8 , 41 , 44 , 291 , 556 , 560 , 562 - 565^ Tendo em vista que o contexto da DC aguda não tratada pode associar-se a mortalidade de até 5% entre os casos diagnosticados ^559^ e ainda a potencial evolução para a fase crônica da doença em todos os casos, considera-se que os benefícios potenciais são muito superiores em relação aos eventos adversos, em sua maioria leves. ^60 , 555^

 Nesse sentido, mesmo em casos assintomáticos ou na impossibilidade de confirmação diagnóstica, mas com suspeita persistente (síndrome clínica compatível e vínculo epidemiológico, com evidência de presença de pessoas de convívio domiciliar/familiar com a doença ou exposição a triatomíneos ou suspeita de transmissão oral ou congênita), o tratamento empírico pode ser considerado. ^8^

 Reconhece-se, portanto, que a intervenção deve ser adotada por gestores da saúde como política de saúde na maioria das situações, considerando-se inclusive que a grande maioria dos profissionais de saúde concorda com a recomendação desse tratamento e que a maioria das pessoas acometidas, quando bem-informadas, deseja realizar a intervenção. 

 No caso de gestantes (em qualquer idade gestacional) com síndrome clínica aguda grave relacionada a miocardite ou a meningoencefalite, o tratamento antiparasitário deve ser indicado independentemente da idade gestacional, em virtude da elevada morbimortalidade materna. ^8^ Além disso, mesmo com nível C de evidência, justifica-se essa indicação pelo elevado risco associado (20-70%) de transmissão congênita, com potencial impacto na saúde de neonatos afetados, considerando-se ainda que os raros relatos de tratamento etiológico durante a gestação estariam associados às poucas evidências relatadas de malformações. ^2 , 57 , 86 , 559^

 Por outro lado, gestantes na fase aguda sem evidências de gravidade clínica devem aguardar, idealmente, o segundo trimestre da gestação para realizar tratamento etiológico. Apesar do benefício potencial de redução da DC neonatal, não existe certeza sobre a eventual ocorrência de mortalidade perinatal ou de malformações fetais. Dessa forma, recomenda-se sempre realizar aconselhamento acerca dos riscos e benefícios da abordagem, com compartilhamento da decisão, sendo justificável o não tratamento em alguns casos. ^2 , 8^

### 9.5 Infecção Congênita

 Assim como os casos de infecção aguda, pessoas diagnosticadas com DC por transmissão congênita também devem receber o tratamento etiológico. Nesses casos, o grau de recomendação também é considerado ‘forte’, independentemente de o diagnóstico ter sido estabelecido por meio de métodos parasitológicos, ainda nas primeiras semanas, ou por testes sorológicos convencionais, 9 meses após o nascimento. ^1 - 3 , 5 , 8 , 57 , 60 , 86 , 112 , 553 , 559 , 566^

 Essa forte recomendação, a despeito da moderada qualidade das evidências disponíveis (nível B) favoráveis ao tratamento tripanocida, é fundamentada nos benefícios previsíveis no contexto de uma situação clínica potencialmente grave, assim como na maior probabilidade de cura concreta da infecção. ^1 , 2 , 8 , 60^

 O tratamento etiológico da pessoa acometida na fase crônica com suspeita de transmissão congênita deve ser realizado, considerando-se a idade atual, o momento da infecção por *T. cruzi* e a expressão do estágio clínico da doença. ^2^ Esses aspectos serão detalhados nas seções a seguir. Reitera-se que diante das atuais evidências para DC e da relevância para a vigilância epidemiológica dos casos crônicos no país, torna-se estratégico ampliar o acesso a saúde e o desenvolvimento de atenção integral para além do tratamento etiológico, devendo-se atentar para a possibilidade de transmissão de mãe para filho. 

### 9.6. Crianças e Adolescentes com Infecção Crônica

 Para essa população, o grau de recomendação do tratamento etiológico é considerado ‘forte’, com nível de evidência B. ^60^ Para essa conduta ressaltam-se os benefícios potenciais em contexto epidemiológico de maior gravidade, além da possibilidade de influenciar, com o tratamento, desfechos como negativação da sorologia e da parasitemia. ^5 , 8 , 60 , 375 , 376 , 567 , 568^

 O tratamento antiparasitário está indicado a todas as crianças (12 anos de idade ou menos) e adolescentes (13 a 18 anos idade) com diagnóstico de FIDC, considerando-se a maior probabilidade de negativação sorológica, traduzindo, assim, adequação da resposta à terapêutica. ^1 , 2 , 5 , 8 , 60 , 375 , 376 , 567^ A fundamentação para essa decisão remete-se a benefícios significativos em termos da redução de danos em órgãos específicos, sem aumento do risco de efeitos adversos diante da melhor tolerância aos antiparasitários nesses grupos etários. ^60^

 A maior expectativa de vida dessa população também justifica a maior probabilidade de que o tratamento apresente melhor efetividade em crianças quando comparadas a adultos. ^8^ Coortes de seguimento de longo prazo utilizando métodos sorológicos convencionais, como controle de cura com período médio de seguimento superior a 10 anos para cada caso, e realizados em contextos reais amazônicos revelaram o sucesso do tratamento etiológico nessa população. Nessas coortes, considerou-se que o tratamento provocou mínimas complicações com potencial de cronicidade, a despeito da persistência de sorologias reagentes. ^291^

 Entretanto, as evidências relativas à prevenção de manifestações clínicas da doença com uso de benznidazol seguem limitadas pelo curto período de seguimento dos estudos, sendo ainda mais reduzidas para o nifurtimox, que deve seguir como alternativa terapêutica. ^2 , 8^ O uso de nifurtimox pode ser considerado ainda como alternativa válida, particularmente em casos envolvendo crianças, adolescentes e adultos jovens com infecção recente e na vigência de intolerância ao benznidazol. ^2 , 8 , 534^

### 9.7. Mulheres em Idade Fértil com Infecção Crônica

 Ressalta-se que, para mulheres em idade fértil (15 a 49 anos) com infecção crônica por *T. cruzi,* considera-se como ‘forte’ o grau de recomendação de tratamento etiológico com benznidazol, inclusive pelo benefício adicional dessa conduta ligado a seu caráter estratégico para controle da transmissão congênita da DC. ^2 , 5 , 8 , 60 , 86 , 89 , 112 , 569 - 572^

 O tratamento antiparasitário diminui significativamente a probabilidade de ocorrência da transmissão congênita, sem observação de eventos adversos fetais ou neonatais. ^8 , 60 , 86 , 89 , 559 , 569 , 570 , 572^ Assim, mesmo com um nível de evidência B, com certeza moderada quando da análise da relação de benefícios e riscos, estabeleceu-se como ‘forte’ o grau de recomendação de tratamento. ^60^ Deve-se recomendar ainda que essas mulheres utilizem métodos anticoncepcionais eficazes de modo sistemático e correto durante todo o período do tratamento tripanocida, descartando-se gravidez antes do início do tratamento. ^2 , 8 , 41 , 60 , 89 , 569 , 570 , 572^ Essas populações devem ainda ser sistematicamente aconselhadas e avaliadas em áreas endêmicas quanto à possibilidade da presença de triatomíneos, que devem ser eliminados do domicílio (intra e peridomicílio) para prevenir reinfecção. 

 Caso ocorra a gravidez, não se recomenda o tratamento da gestante com DC cursando na fase crônica, tendo em vista que o risco de transmissão congênita é baixo, em torno de 1,5% a 2% no Brasil. ^2 , 46 , 57 , 86 , 559^ No entanto, gestantes com quadro clínico agudo e grave de DC, expresso por miocardite ou meningoencefalite, ou ainda na fase aguda, mesmo não grave da doença diagnosticada no primeiro trimestre, devem passar por avaliação criteriosa e decisão compartilhada, individualizada caso a caso, quanto à possibilidade de tratamento etiológico, em consonância com o que foi previamente discutido. ^8^

 Na Amazônia brasileira, onde predominam infecções agudas, há registro de transmissão vertical por desconhecimento da gravidez em contextos de surto ou microepidemia familiar, com alguns relatos bem documentados de infecção congênita mesmo após início de tratamento materno com benznidazol. ^279 , 556^

 Finalmente, deve-se registrar a importante iniciativa internacional sob o acrônimo ‘CUIDA Chagas’, à qual aderiu o Ministério da Saúde do Brasil, envolvendo também Bolívia, Colômbia e Paraguai, além de cinco estados brasileiros (Bahia, Goiás, Minas Gerais, Pará e Rio Grande do Sul). Com início em 2022, o projeto inclui medidas e modelos de implementação diagnóstica e terapêutica para eventual eliminação da transmissão vertical da DC entre mulheres em idade fértil, cronicamente infectadas por *T. cruzi* , a serem avaliadas ao longo de quatro anos de desenvolvimento. Entre outros relevantes aspectos, incluiu-se nesse consórcio internacional o objetivo de testar, em estudo controlado randômico, se um regime terapêutico tripanocida com benznidazol menos prolongado (duas semanas) é pelo menos tão eficaz quanto o habitual e se tem menos efeitos colaterais (de 60 dias). ^573^

### 9.8. Adultos em Geral com Infecção Crônica

 O potencial de benefício do tratamento etiológico para todo adulto com infecção crônica por DC não é sustentado por evidências suficientes que possam embasar uma recomendação forte com nível elevado de evidência para essa indicação, genericamente, para quaisquer situações clínico-epidemiológicas. ^8 , 60^ Essa recomendação assume assim um nível condicional a depender do caso em análise, tendo em vista a limitada evidência disponível para algumas populações, ^60^ reconhecendo-se, entretanto, nesses casos, aspectos relativos ao princípio da assimetria (se o benefício potencial supera em muito o risco de efeitos colaterais, no caso em apreço). Dessa forma, em linhas gerais, essa decisão deve ser compartilhada entre o profissional médico e a equipe de saúde, a pessoa acometida e sua família, a depender do momento de infecção, da idade e das condições clínicas. ^1 , 2 , 5 , 8^

 Em geral, reitera-se que, como discutido para adolescentes, para adultos em qualquer idade com infecção recentemente adquirida, a despeito do modo de transmissão, o grau de recomendação do tratamento é considerado ‘forte’, com nível de evidência B. ^60^

 Tendo em vista a estratificação definida no delineamento em parte significativa dos estudos consistentes para avaliação do tratamento etiológico, optou-se neste documento por estabelecer como parâmetro de corte a idade de 50 anos, assim como em outros documentos referenciais nacionais e internacionais. ^2 , 5 , 8^ Ressalta-se que as diretrizes de prática clínica da OPAS publicadas em 2018 não adotaram essa estratificação etária, trazendo em perspectiva as perguntas: ‘ *Qual é a intervenção terapêutica mais segura e eficaz para doentes adultos com infecção crônica por T. cruzi e [sem/com] lesões de órgãos específicos* ?’. Para os casos na forma crônica indeterminada, o tratamento etiológico foi estabelecido como ‘condicional’ com nível de evidência ‘fraco’, enquanto, para os casos com lesão de órgãos, o tratamento não foi recomendado, com nível de evidência moderado. Os procedimentos metodológicos das diretrizes da OPAS foram desenvolvidos a partir de revisões sistemáticas e estudos primários publicados até agosto de 2017 (PubMed, EMBASE, Cochrane) e por meio de pesquisas manuais com análise pelo GRADE. ^27 , 60^

 Conforme apresentado a seguir, desde 2017, foram publicados novos estudos que agregaram evidências às já disponíveis, o que demarcou o estabelecimento das recomendações constantes no presente documento, ampliando a oportunidade de acesso a tratamento da infecção por *T. cruzi* . 

 Em adultos até 50 anos de idade com a forma crônica indeterminada, o tratamento é recomendado, considerando-se que as vantagens de sua realização parecem superar as desvantagens e que há benefício mais evidente quanto à prevenção de doença cardíaca. ^2 , 8 , 38 , 318 , 320 - 323 , 379 , 574^ Trata-se de recomendação forte com nível de evidência B, tendo em vista estudos mais recentes reconhecendo que o tratamento etiológico pode reduzir o risco de desenvolvimento da doença cardíaca a longo prazo, ^2 , 60 , 297 , 318 , 321 - 325 , 542^ mesmo sem uma clara evidência sobre o impacto na mortalidade. ^8 , 60 , 575 - 577^ A probabilidade de se obter parasitemia negativa em curto prazo é maior, enquanto a de sorologia não reagente é evidenciada apenas em longo prazo. ^38 , 41 , 60 , 318 , 324 , 542 , 557 , 578 , 579^ Por outro lado, o tratamento está potencialmente associado a risco considerável de eventos adversos, que, embora na maioria sejam considerados leves e minimizados por meio de monitoramento qualificado, ^58 , 60 , 318 , 323 , 536^ em alguns casos são suficientemente graves para acarretar interrupção terapêutica. 

 Considera-se ainda que para pessoas com 50 anos de idade ou mais e DC em fase crônica, o benefício do tratamento etiológico na FIDC associa-se a grau de incerteza maior, o que leva a uma recomendação condicional (ou ponderada) do tratamento etiológico com nível de evidência C. ^1 , 2 , 5 , 60 , 318 , 322 - 325 , 542 , 579^

 Como apresentado anteriormente, o fator idade para tratamento etiológico deve ser relativizado, considerando-se particularmente para pessoas com infecção recente (por exemplo, em contextos epidemiológicos de transmissão oral ou por transfusão onde a idade é um fator independente da evolução clínica) ou que tiveram sua infecção durante a vida adulta sem comorbidades e dentro de um processo claro na sociedade brasileira de transição demográfica com maior expectativa de vida. ^2 , 8^ Em geral, essas perspectivas trazem a possibilidade de recomendação condicional de tratamento etiológico nessa população. 

 Nos casos de adultos com formas crônicas determinadas em fases iniciais não avançadas (cardíaca e digestiva), a decisão para indicar o tratamento etiológico também deve ser compartilhada, com aconselhamento sobre os potenciais benefícios e riscos, podendo-se, assim, oferecer a possibilidade de tratamento, sendo tratar com benznidazol ou não tratar alternativas válidas, caso não haja contraindicações. A recomendação do tratamento etiológico nesses casos é condicional ou ponderada, com nível de evidência C. ^1 , 2 , 5 , 8 , 60 , 318 , 322 , 323 , 542^ Entende-se por CCDC em fases pouco avançadas (iniciais) a daqueles casos apenas com alterações no ECG (por exemplo, distúrbio da repolarização ventricular, EV, BRD, BDASE, BAV de 1º grau, dentre outras), mas função ventricular sistólica global preservada ou levemente reduzida (FEVE superior a 40%), estágios B1 e B2 de IC e sem arritmias graves. ^1 , 2 , 8 , 318 , 322 , 323^

 Ao se optar pelo tratamento etiológico, esse pode ser considerado independentemente do diagnóstico da forma crônica digestiva isolada ou em associação, isso é, com doença cardiodigestiva, ^8^ pois o objetivo do tratamento é a prevenção das lesões cardíacas. Nos casos com alterações digestivas instaladas e mesmo naqueles sem a forma digestiva, não existem evidências indicando benefício da adoção do tratamento antiparasitário em prevenir ou retardar o aparecimento ou a progressão do megaesôfago e do megacolo. ^8 , 559^ Alguns pacientes com megaesôfago podem ter a eficácia do tratamento com benznidazol comprometida por interferência com a ingestão ou absorção do fármaco. ^2 , 8^ Apesar de o diagnóstico da forma crônica digestiva não representar contraindicação para o tratamento etiológico, recomenda-se realizar reabilitação clínica, dilatação ou correção cirúrgica do megaesôfago previamente à adoção do tratamento etiológico, com a finalidade de garantir o trânsito do medicamento e sua absorção. ^2 , 8 , 44^

 Quando já há cardiomiopatia crônica instalada, em geral não há evidências que sustentem a possibilidade de o tratamento etiológico impactar significativamente a evolução para morte ou a progressão da doença cardíaca, mesmo aumentando-se a probabilidade de negativação da parasitemia, avaliada por PCR. ^60 , 323 - 325^ Dessa forma, o tratamento antiparasitário não deve ser recomendado para pessoas com lesão orgânica avançada (formas cardíacas em estágios C e D) ou muito idosas. ^1 , 2 , 5 , 8 , 32 , 60 , 318 , 322 , 324 , 540 , 579^ Nesses casos, o tratamento etiológico não muda a história natural da doença, pode estar associado a risco aumentado de eventos adversos graves, além de induzir custos diretos e indiretos para as pessoas acometidas e suas famílias, ampliando-se, dessa forma, sua vulnerabilidade social. 

 Portanto, todos os esforços devem ser envidados para diagnóstico e tratamento etiológico oportuno de casos de DC com o objetivo de prevenir a progressão da doença. Ressalta-se que o risco anual de mortalidade na CCDC é considerável (7,9%; IC 95%: 6,3-10,1%) e associado principalmente a causas atribuíveis cardiovasculares, em especial quando da vigência de baixa FEVE e classificados como estágios C e C/D. ^448^

 Situação especial é a de pessoas com megaesôfago grave, impedindo a adequada absorção do agente tripanocida. Em tais situações clínicas, sem cardiopatia manifesta ou com cardiopatia pouco avançada, em que o tratamento etiológico objetiva prevenir a progressão da doença cardiovascular, esse pode ser indicado após o tratamento cirúrgico do megaesôfago. ^2^ A indicação do tratamento etiológico também teria recomendação ‘condicional’ e com nível de evidência C. 

 Não obstante essa concepção essencial, torna-se oportuno registrar que a análise meticulosa dos resultados obtidos com o ensaio clínico BENEFIT, o mais extenso ECR sobre terapêutica tripanocida em pacientes com CCDC (a maioria não avançada), permitiu identificar alguns aspectos relevantes a realçar. De fato, na população como um todo, envolvendo pacientes de cinco países da América Latina (Brasil, Argentina, Colômbia, Bolívia e El Salvador), o tratamento etiológico com benznidazol não logrou impactar favoravelmente a evolução dos pacientes quanto a mortalidade e outros desfechos graves da cardiomiopatia. ^324^ Tampouco ocorreu benefício, comparativamente ao placebo, sobre a disfunção ventricular regional, alteração precoce e frequentemente detectada em tais indivíduos e dotada de real conotação de mau prognóstico. ^342^

 Entretanto, a análise global dos resultados tornou-se passível de críticas e, muito provavelmente, impediu a devida apreciação de alguns desacertos metodológicos com relevantes implicações potenciais para a aplicabilidade dos resultados da investigação. ^325^ Por exemplo, comparativamente ao grupo tratado com placebo, no grupo do tratamento tripanocida com o benznidazol, verificou-se redução estatisticamente significante da taxa de hospitalizações por causas cardiovasculares, aspecto bastante realçado em muitos estudos envolvendo pacientes com IC, mas que sequer foi discutido na análise primária do estudo BENEFIT. ^324^

 Entre outros aspectos merecedores de apreciação crítica, deve-se considerar que a análise de subgrupos inicialmente realizada no âmbito do estudo BENEFIT foi arbitrária, não pré-especificada e não obedeceu a critérios defensáveis, podendo ter sido inadequadamente enviesada. ^324 , 325^ Em contraste, análise *post-hoc* dos resultados desse estudo evidenciou a possibilidade de que o efeito do tratamento etiológico nos pacientes brasileiros (40% da amostra global estudada) possa ter sido positivo, particularmente quando se confrontam os resultados obtidos no subgrupo arrolado no Brasil com os observados nos quatro demais países em que a pesquisa foi realizada. ^325^

 Destaque-se que essa possibilidade deve ser encarada somente como geratriz de uma hipótese e com certeza mereceria estudo subsequente especificamente para comprová-la ou não. De toda forma, a hipótese corolário dessa interpretação, de que esse tratamento parasiticida seja mais eficaz quando aplicado em brasileiros já com a CCDC, é biologicamente plausível e pode estar embasada na predominância do genótipo parasitário TcII que se verifica no Brasil, que pode ser mais sensível ao tratamento com o benznidazol comparativamente a outras cepas de *T. cruzi* e ao nifurtimox. Em realidade, há razões científicas para que o tratamento de pacientes baseado em fármacos tripanocidas (inclusive aqueles ainda em fase de validação) seja lastreado em consideração tanto da diversidade genômica parasitária ^248^ como da complexa interação das diversas linhagens do parasito com o hospedeiro humano, que resultam em formas variadas de expressão clínica. ^250^

 Com base em todas essas considerações, abre-se a perspectiva de que no Brasil o grau de recomendação condicional de se oferecer o tratamento etiológico a indivíduos já com CCDC não avançada seja ponderada com mais ênfase no potencial benefício do que o que ocorreria em outros países. Finalmente, ressalte-se a expressiva gravidade da DC e a necessidade de diagnóstico e atenção integral à pessoa com cardiopatia de modo oportuno e com base em manejo clínico qualificado. ^1 , 2 , 44 , 60 , 324^

 Além disso, considerando-se as atuais evidências sobre o tratamento etiológico da doença bem como a relevância da vigilância epidemiológica, a notificação compulsória de casos crônicos de DC deve ser implementada, o que possibilitaria ampliar o acesso ao diagnóstico e tratamento a mais pessoas acometidas. ^8 , 44 , 56 , 91 , 94 , 113^

### 9.9. Reativação da Doença de Chagas

 A RDC consiste na agudização da infecção crônica por *T. cruzi* , caracterizada pelo aumento da parasitemia (semelhante à doença na fase aguda) e pela incapacidade de o sistema imune controlar a infecção, em geral associada à imunossupressão farmacologicamente induzida – transplantes, tratamentos imunossupressores – ou à coinfecção com HIV. ^1 , 2 , 8 , 83 , 84 , 580^

 A RDC está associada a elevada morbimortalidade em virtude da infecção no sistema nervoso central e da miocardite, impactando criticamente também a qualidade de vida. ^2 , 8 , 83 , 84^ A prevalência observada de RDC com base na parasitemia em pessoas com DC e imunossupressão, sem profilaxia com tripanocida, foi aproximadamente 28%, sendo: 1,8% em transplante de fígado, 23,3% em transplante de medula óssea, 27,3% em transplante de rim, 30,9% em transplante de coração e 39,6% na infecção por HIV/AIDS. ^60^

 Caso ocorra reativação, deve-se iniciar o tratamento etiológico indicado para a fase aguda da DC. ^2 , 8 , 83 , 84^ Apesar do nível de evidência moderado (B), a recomendação é classificada como forte, pois os medicamentos antiparasitários podem apresentar benefícios potenciais na prevenção da ocorrência de reativações e suas consequências, assim como no seu controle e mesmo quanto à sua recorrência. ^5 , 60 , 84 , 580 - 584^

 Na infecção por HIV, na vigência de DC crônica sem reativação e sem tratamento etiológico prévio, o tratamento deve ser realizado preferencialmente com benznidazol, avaliando-se o *status* imunológico, em virtude do risco aumentado de ocorrência de síndrome inflamatória de reconstituição imune. ^8 , 83 , 84^

 Para os casos com transplantes e RDC, o tratamento também está indicado com a mesma posologia utilizada para os casos não relacionados a transplantes, sendo o benznidazol a alternativa preferencial pelo melhor perfil de eventos adversos e maior experiência com utilização no país. ^2 , 8^ Não há evidência consistente para recomendar profilaxia secundária em casos submetidos a transplantes, mas pode ser indicada em casos selecionados, particularmente naqueles com maior grau de imunossupressão. ^8 , 60^

 De modo geral, o tratamento etiológico pode contribuir para a prevenção de complicações clínicas (a exemplo da cardiopatia), devendo ser considerado com as mesmas recomendações e níveis de evidência utilizados em outras situações relativas à DC crônica em pessoas sem imunossupressão. ^8 , 84^

 Tanto para pessoas infectadas por HIV quanto com transplantes, a qPCR pode contribuir no monitoramento clínico; entretanto, sua recomendação de rotina ainda está por ser definida. ^8 , 60 , 580^ Ressalta-se que os episódios de RDC podem ocorrer de forma repetitiva, devendo ser tratados quando documentados, o que justifica o monitoramento parasitológico regular enquanto estiver mantida a condição de imunossupressão. ^2 , 83 , 84^

### 9.10. Infecção Acidental

 Em acidentes com material biológico contaminado com *T. cruzi* e risco elevado para transmissão da doença, como instrumentos perfurocortantes ou por contato com mucosas ou pele com solução de continuidade ou manipulação de material biológico com parasitos vivos (amostras de cultura de *T. cruzi* , amostras biológicas de casos com elevada parasitemia e material de necropsia, vetores e animais de laboratório infectados), deve-se indicar a profilaxia primária, iniciando-se com benznidazol na dose de 7 a 10mg/kg imediatamente após o acidente e mantendo-o por 10 dias. ^1 , 2 , 558 , 585^ Trata-se de uma conduta com recomendação forte, apesar do limitado nível de evidência (C), mas que considera o princípio de assimetria. ^2 , 5 , 8 , 60 , 83^

 Devem ser realizados exames sorológicos antes de se iniciar o tratamento e no 20º, 40º e 60º dias pós-tratamento para monitoramento de eventual soroconversão. ^2^ Em caso de os exames sorológicos serem reagentes, o tratamento antiparasitário convencional deverá ser realizado como descrito previamente para a fase aguda. Em situações de risco mínimo, como apenas contato superficial com sangue de casos com a DC em fase crônica, a profilaxia medicamentosa não está indicada, recomendando-se a realização de exames sorológicos imediatamente após e no 20º, 40º e 60º dias após o acidente. ^2^ Havendo soroconversão, o tratamento convencional para a fase aguda da DC deverá ser instituído e o monitoramento pós-terapêutico deve ser realizado como preconizado para a fase aguda. Se a sorologia permanecer positiva após o tratamento, deve-se procurar documentar possível falha terapêutica para um novo tratamento com benznidazol ou nifurtimox. ^1 , 2 , 83 , 534^

### 9.11. Avaliação de Cura da Doença de Chagas Pós-Tratamento Etiológico 

 Em uma doença em que existem apenas duas opções terapêuticas com indicações consistentes para uso, não há evidências disponíveis sobre métodos complementares para avaliar, no contexto da rotina dos serviços de saúde, o efeito do tratamento etiológico na eliminação do parasito, particularmente na fase crônica. ^1 , 8 , 41 , 60^ A garantia de acesso ao tratamento é fundamental, tendo uma função social clara dado o caráter de negligência relacionado às pessoas acometidas pela doença. Muitas das vezes, argumentos associados a eventos adversos e não estabelecimento de cura são utilizados como justificativa para o não tratamento no SUS. Como condição crônica, a DC demanda a necessidade de uma atenção integral e longitudinal a todas as pessoas acometidas. 

 Não existe método complementar para confirmar a evolução para cura (que seria considerado padrão-ouro), o que torna os testes sorológicos e os testes moleculares, mesmo com todas as limitações técnicas, métodos potencialmente disponíveis e úteis para avaliar a resposta ao tratamento antiparasitário na fase crônica. ^8 , 38 , 60 , 379^

 Neste sentido, não existem evidências relativas à necessidade de seguimento com controle sorológico pós-tratamento ou retratamento após curso terapêutico completo. ^8 , 60^ A qualidade das evidências que sustentam o uso de negativação sorológica como substituto para desfechos clinicamente relevantes é ‘baixa’ ou ‘muito baixa’, representando, na realidade, um desfecho indireto. ^8 , 60^

 Além disto, a negativação sorológica pós-tratamento em adultos pode ser muito lenta e levar mais de duas décadas para se efetivar, ^38 , 379 , 557^ e ser alcançada por apenas aproximadamente 1/3 dos casos, na dependência de diferentes fatores como idade no momento do tratamento, tempo entre o tratamento e o acompanhamento e área em que ocorreu a infecção. ^586^ Para crianças e adolescentes, a negativação sorológica pode ocorrer dentro de cinco anos em 3/4 dos casos. ^376 , 553 , 556 , 560 - 567^ Análises em crianças e adolescentes no contexto amazônico com DC aguda indicam persistência de sorologias reagentes em quase 55% dos casos, em um período médio de seguimento de cada caso por aproximadamente 11 anos após tratamento, além de proporção de 17% de casos com respostas sustentadas de negativação sorológica. ^291^

 Apesar de alguns estudos sugerirem o uso da PCR para monitoramento e controle da resposta terapêutica, a sensibilidade da técnica é variável ^587^ e não há disponibilidade de métodos validados e pactuados no SUS, restringindo sua aplicabilidade a atividades de pesquisa. ^8 , 46^ Reconhece-se, entretanto, que a PCR sendo positiva ainda nos primeiros 24 meses após o tratamento indica possibilidade de falha terapêutica. ^562 , 587^

 Os percentuais de cura verificados por diversos estudos após o tratamento antiparasitário da DC apresentam divergências, mas mesmo assim, reconhece-se a importância do tratamento etiológico tanto na fase aguda quanto em algumas formas clínicas da doença crônica. ^2 , 8 , 557^

 Além disto, mesmo com todas as limitações já mencionadas da terapêutica antiparasitária vigente, pode-se alcançar a supressão da parasitemia em muitos cenários, ^1 , 2 , 8 , 46 , 320 , 324 , 540 , 574 , 579^ o que torna inquestionável a utilidade do tratamento etiológico da DC em parte considerável das situações clínicas, independentemente da demonstração de cura, à exceção da DC aguda. Portanto, para a fase crônica da DC, a definição de critério para cura perde o sentido prático e contribui sobremaneira como forte barreira para o acesso. 

#### 9.11.1. Onde Realizar Tratamento da Pessoa Acometida 

 Para além da liderança técnico-científica sobre a DC, o Brasil tem um grande diferencial em relação à maioria dos países endêmicos para DC: a existência do SUS, de caráter público, universal e de base democrática, dentro dos referenciais de direito à saúde da Constituição Federal de 1988. Amplia-se, assim, a possibilidade de garantia de acesso a diagnóstico e tratamento da DC no país, ^2 , 49 , 113^ como tem sido verificado em países não endêmicos. 

 Entretanto, apesar de um contexto favorável e dos referenciais disponíveis a partir de portarias, diretrizes, consensos e do próprio PCDT, ^1 - 3 , 8 , 60^ com benefícios clínicos demonstrados a curto, médio e longo prazos, não se tem conseguido implementar de modo consistente o diagnóstico e o tratamento, nem a vigilância de casos de DC crônica no território nacional. ^113^ Questões como centralização das ações de atenção, vigilância e controle da DC contribuem para essa situação. Portanto, uma visão global unificada sobre o atual estágio de desenvolvimento de iniciativas para controle da DC no Brasil, apesar de reconhecer as conquistas alcançadas ao longo desses quase 120 anos, indigita a premente necessidade de implementação e integração das medidas englobadas no PCDT com vigilância sustentada da DC e adesão a diretrizes nacionais e internacionais. ^113^

 Devem-se considerar as especificidades da rede de atenção do SUS, reconhecendo-se, entretanto, que o tratamento etiológico da infecção por *T. cruzi* é factível, seguro e operacionalmente viável na APS. ^2 , 8 , 44 , 58 , 94 , 113 , 529 , 533 , 536^ Reconhece-se a possibilidade de que a rede de APS assuma a condução de casos com DC na fase aguda não grave, com a FIDC, ou mesmo com formas crônicas (cardíaca, digestiva ou cardiodigestiva) na vigência de doença estável e não grave, bem como de gestantes com DC em fase crônica sem comorbidades. ^8 , 44^ Há ainda evidências de que médicos de família e comunidade e suas equipes, conhecendo as particularidades dos medicamentos e da doença, podem manejar clinicamente os casos. ^2 , 44 , 58 , 533^

 Dependendo da gravidade das condições clínicas de cada caso, principalmente na vigência de fase aguda ou RDC, assim como de formas crônicas descompensadas, pode haver a necessidade de apoio matricial para o plano de cuidado ou de efetivação do encaminhamento para unidades de saúde mais especializadas ou de referência, ou até mesmo de internação hospitalar, em condições esporadicamente configuradas. ^2 , 8 , 44 , 94^

 A Tabela 9.1 sintetiza as recomendações para tratamento etiológico da infecção por *T. cruzi* em diferentes contextos da DC, segundo força de recomendação e nível de evidência, com base nos referenciais do sistema GRADE. 

**Tabela 9.1:** – Recomendação de tratamento etiológico em diferentes contextos da doença de Chagas, segundo força de recomendação e nível de evidência (adaptado do sistema GRADE)

TRATAMENTO ETIOLÓGICO DA DOENÇA DE CHAGAS Infecção por *Trypanosoma cruzi*	Grau de recomendação	Nível de evidência
Crianças com infecção aguda	Forte	B
Crianças com infecção congênita	Forte	B
Adolescentes e adultos em geral com infecção aguda ou recentemente adquirida	Forte	B
Crianças e adolescentes com infecção crônica	Forte	B
Mulheres em idade fértil com infecção crônica	Forte	B
Pessoas em geral com reativação da infecção crônica (HIV/AIDS ou outras condições imunossupressoras, incluindo transplantes)	Forte	B
Pessoas em geral com infecção por acidente com material biológico em contextos laboratoriais ou de atenção à saúde	Forte	C
Gestantes com síndrome clínica aguda grave - miocardite ou meningoencefalite	Forte	C
Adultos < 50 anos de idade com infecção crônica (forma crônica indeterminada)	Forte	B
Adultos ≥ 50 anos de idade com infecção crônica (forma crônica indeterminada)	Ponderado	C
Adultos em geral com infecção crônica (formas crônicas determinadas em fases iniciais - cardíaca e digestiva não avançadas)	Ponderado	C
Pessoas em geral com infecção crônica e lesão orgânica avançada na forma digestiva (não associada à doença cardíaca avançada), após correção cirúrgica	Ponderado	C
Pessoas em geral com infecção crônica e lesão orgânica avançada na forma crônica cardíaca ou digestiva (associada à doença cardíaca avançada) não devem ser tratadas	Forte	C

 Vale finalmente ressaltar que, quando da elaboração final do presente capítulo, publicou-se atualização de antiga revisão sistemática e respectiva meta-análise relativamente a estudos de tratamento etiológico com benznidazol para pessoas com infecção por *T. cruzi* . ^588^ As conclusões essenciais dessa publicação são de molde a ter coerência com as recomendações aqui exaradas na Diretriz Brasileira. Entretanto, reitera-se que, no atual momento, para além da busca de evidências científicas mais robustas, todos os esforços devem ser envidados para a garantia de acesso a diagnóstico e tratamento etiológico da DC nos sistemas nacionais de saúde. 

## 10. Condutas Terapêuticas na Disfunção Ventricular e Insuficiência Cardíaca 

### 10.1. Recursos Farmacológicos

#### 10.1.1. Classificação da Insuficiência Cardíaca

 Nossas recomendações priorizam pacientes com FEVE reduzida, visto que a maioria das condutas farmacológicas foram validadas nesse cenário. Nesse contexto, devemos compreender a diferença entre critérios de inclusão de um estudo científico e indicação clínica. Estudos primam por selecionar pacientes com menor FEVE (< 35% ou < 40%) a fim de otimizar a incidência do desfecho de interesse, aumentando-se o poder estatístico. Pelo fato de a magnitude do efeito absoluto (NNT) ser mais relevante em pacientes de maior risco (ou seja, devo tratar poucos pacientes para obter um benefício) e não se identificar motivo plausível para a ocorrência de interação qualitativa (desaparecimento do efeito) quando um determinado ponto de corte de FEVE é ultrapassado, optamos por generalizar nossas recomendações para o uso dos principais fármacos destinados ao tratamento da IC em pacientes com FEVE < 55%, evitando excesso de categorização. 

 No entanto, deve-se considerar que existe um *continuum* de relação (e que é inversa) entre FEVE e benefício terapêutico, de tal forma que, quanto menor for o valor da FEVE, maior será o benefício absoluto da terapia proposta. Para fins de simplificação, recomendações fortes para FEVE ≤ 40% se tornarão ponderadas para FEVE entre 41% e 54%. Julgamos também que há maior possibilidade de modificação de efeito em pacientes com alterações de contratilidade segmentar, porém sem disfunção ventricular global, os quais se encaixam no estágio B de IC. Durante a elaboração desta diretriz, predominou a noção de que evidências para esses pacientes têm importância na dimensão científica, mas ainda são insuficientes para promover qualquer recomendação. 

#### 10.1.2. Dose Máxima de Medicações

 Esta diretriz não respalda a obstinação por se atingir a dose máxima das medicações em detrimento da polifarmácia, preferindo enfatizar a individualização da melhor dose de cada fármaco para cada paciente. A racionalidade dessa posição baseia-se em algumas justificativas. A dose proposta ou mesmo aquela atingida pelos pacientes nos ensaios clínicos faz parte de uma estratégia científica, com objetivo de gerar contraste entre grupos e testar hipóteses conceituais. Uma vez demonstrado o conceito, esse deve ser aplicado de forma individualizada, ponderando benefícios e danos. Assim, a escolha da dose de um medicamento diz mais respeito à dimensão do raciocínio clínico do que da evidência. Segundo, não há dados científicos convincentes sobre a magnitude de efeito incremental relacionado à dose máxima ( *versus* dose ponderada) e se aquela supera consequências não intencionais. Terceiro, tolerabilidade e efeitos adversos são subestimados em ECR de eficácia, pois, usualmente, são selecionados candidatos ideais para o tratamento em questão e as condutas são mais bem controladas. Portanto, não transformamos eficácia em efetividade com padronização do máximo. O incremento de efetividade decorrerá de judiciosa individualização. 

#### 10.1.3. O Paciente Contemporâneo

 À medida que se prolonga a vida do paciente com CCDC e IC, ele tende a sofrer de outras doenças acumuladas com o envelhecimento. 

 Recentemente, em ECR de pacientes com CCDC de centro único (FIOCRUZ), observou-se média de idade de 65 anos, com índice de massa corporal médio de 27,4kg/m ^2^ e 1/3 com HAS, ^589^ diverso, portanto, de casuísticas que mostravam indivíduos mais jovens e frequentemente sem comorbidades. Outro ponto a ser notado é a possibilidade de o curso clínico da IC de etiologia da DC ser distinto daqueles de etiologias isquêmica e dilatada idiopática, ^590^ por possuir grau mais acentuado de disfunção autonômica, maior densidade de arritmia ventricular e bloqueios intracardíacos, mais elevada carga de fibrose miocárdica, comprometimento mais frequente de VD e maior grau de esfericidade/remodelamento cardíaco e inflamação miocárdica – todos fatores que poderiam interferir com a resposta ao tratamento farmacológico padrão. ^591 , 592^

 Uma pior trajetória clínica, do ponto de vista meramente estatístico, sugere maior benefício absoluto de tratamentos com nível B de evidência se comparados às populações-alvo dos estudos, não devendo implicar em violação do princípio da evidência indireta, ou seja, por extrapolação. 

 É oportuno mencionar que o estudo da FIOCRUZ acima citado ^589^ incorpora-se em iniciativa abrangente de pesquisas translacionais destinadas a explorar, em caráter experimental e também clínico, hipóteses de potencial benefício com suplementação de nutrientes, como selênio, e antagonismo de fatores inflamatórios para modificar a evolução da CCDC. ^593^ Talvez o mérito primordial dessas investigações incipientes resida no apelo de sua hipótese fortemente embasada, fisiopatologicamente, no caráter inflamatório da CCDC e somente a pesquisa dirigida poderá responder no futuro quanto ao êxito dessas intervenções. 

#### 10.1.4. Revisão da Literatura

 Para cada fármaco ou classe de fármacos utilizados no tratamento da IC, foi realizada uma revisão sistemática da literatura até 22/08/2021, visando responder à seguinte questão PICO da medicina embasada em evidência: “Esses fármacos são eficazes ou efetivos para alívio de sintomas e/ou redução de mortalidade em pacientes sintomáticos com IC sistólica secundária à CCDC, com perfil de segurança similar àquele para as outras etiologias da síndrome?”. Foram utilizados os seguintes termos padrão ou *Medical Subject Headings* (MESH): “ *beta-blockers, spironolactone, sacubitril-valsartan, ivabradine, sodium-glucose transporter 2 inhibitors* ”, “ *heart failure* ” ou “ *Chagas disease* ”, com limite para tipo de publicação (“ *clinical trial* ”). As bases de dados MedLine/PubMed, Lilacs, Web of Science e EMBASE foram usadas como fonte de busca. 

#### 10.1.5. Terapia Farmacológica

***10.1.5.1. Diuréticos***

 A terapia promotora de diurese na IC é incompreendida em sua magnitude de efeito. A ausência de ECR que compare diurético *versus* placebo pode gerar a equivocada impressão de que, diferentemente de betabloqueadores ou IECA, diuréticos de alça não reduzem a mortalidade. Essa visão ressente-se da percepção de que a carência desses estudos se deva justamente à ausência de *equipoise* para o tipo de paciente em que se validou benefício prognóstico com as demais terapias. Ou seja, na IC, a administração de diurético constitui terapia de plausibilidade extrema, o que corresponde ao paradigma do paraquedas, ^31^ representando justificativa desta diretriz para o nível de evidência C em indicação farmacológica. Assim, recomendamos fortemente terapia com intuito diurético para IC com moderada a importante redução de fração de ejeção e para casos com redução leve da fração de ejeção. 

***10.1.5.2. Inibidores do Sistema Renina-Angiotensina-Aldosterona***

 Está cabalmente demonstrado, por inúmeros ensaios clínicos de qualidade, que em pacientes com IC e FEVE reduzida, diversos IECA reduzem desfechos relevantes de morbimortalidade. ^594 - 596^ Além disso, esses fármacos podem ser substituídos pelos bloqueadores de receptores da angiotensina II (BRA) em casos de má tolerabilidade. ^597^ Entretanto, na IC da CCDC, não há evidências diretas de benefício por meio de ECR realizados especificamente nessa população. Sendo assim, julgamos que a evidência a respeito do uso de IECA na CCDC é indireta, proveniente de estudos de ótima qualidade que testaram a eficácia desse tratamento nos tipos mais comuns de miocardiopatia (isquêmica e dilatada idiopática, por exemplo) (nível B). Acompanhando o racional de que fração de ejeção é um *continuum* prognóstico (ao invés de uma variável binária, dicotômica), quanto maior o grau de disfunção ventricular, maior o benefício absoluto. Portanto, a recomendação é definida como forte para pacientes com IC e FEVE ≤ 40% e ponderada para pacientes com IC e FE levemente reduzida (ICFElr). 

 Estudos com número bastante reduzido de pacientes, avaliando captopril e enalapril na IC da CCDC, evidenciaram diminuição da ativação neuro-humoral simpática e dos níveis de angiotensina plasmática, além de melhora da disfunção diastólica e do remodelamento ventricular. ^598 - 600^ Esses pacientes frequentemente cursam com pressão arterial sistólica diminuída, podendo se tornar sintomáticos com a introdução dos IECA ou BRA que, por sua vez, devem ser titulados de forma gradual, buscando-se diminuir as doses dos diuréticos, quando o paciente não mais apresentar edema. 

 Vale destacar que, nas últimas décadas, as diretrizes internacionais têm enfatizado a busca da dose-alvo terapêutica de IECA ou BRA nos pacientes com IC e fração de ejeção reduzida (ICFEr), algo que pode ser elusivo e consistir em limitação para a prática clínica, considerando-se que os pacientes com CCDC estão mais propensos a apresentar hipotensão arterial sintomática. Portanto, aqui devemos buscar a melhor dose tolerada e particularmente proceder à titulação lenta nesse grupo particular de pacientes sujeitos a dificuldades posológicas. ^601^

***10.1.5.3. Betabloqueadores***

 As primeiras experiências usando betabloqueadores para tratamento de pacientes com IC datam da década de 70, quando alguns pesquisadores investigaram o efeito do fármaco em sete pacientes com cardiomiopatia, IC avançada e taquicardia. ^602^ Na ocasião, um paciente recebeu alprenolol 50 mg duas vezes ao dia e os demais receberam practolol, nas doses que variaram entre 50 mg e 400 mg, duas vezes ao dia. Os autores observaram melhora clínica, redução da cardiomegalia e melhora da função ventricular avaliada pelo fonocardiograma, ECO, apexcardiograma e pela curva do pulso carotídeo. Apesar dos resultados promissores reportados pelo grupo sueco, só na década de 90 os betabloqueadores foram adequadamente investigados na IC. 

 O estudo seminal que sugeriu benefício do betabloqueador em ICFEr foi o *U.S. Carvedilol Heart Failure Study* , ^603^ que randomizou 1.094 pacientes para carvedilol ou placebo e demonstrou redução de mortalidade. 

 Ao longo desses últimos 25 anos de investigação clínica, os betabloqueadores se consolidaram no tratamento da IC. Em meta-análise ^604^ envolvendo 10 ensaios clínicos e 18.254 pacientes com IC e FEVE reduzida, os betabloqueadores reduziram a mortalidade global em 27%. 

 No aspecto prático, é importante destacar que os pacientes com ICFEr podem piorar na fase inicial do uso do medicamento. ^21^ Impõe-se, portanto, vigilância quanto à piora, aparecimento de bradicardia, bloqueio cardíaco e hipotensão, em especial nas primeiras semanas de ajuste do tratamento. 

 No contexto da IC, esse fato é especialmente importante, pois os pacientes com CCDC são mais susceptíveis à ocorrência dessas manifestações adversas quando em uso de betabloqueadores. Ainda que a CCDC não tenha sido incluída nos grandes estudos multicêntricos que investigaram betabloqueador e mortalidade e que haja peculiaridades da síndrome com essa etiologia, que é associada com notória desregulação do sistema nervoso autonômico, como revisto em outros capítulos desta diretriz, não há plausibilidade biológica em se questionar o benefício do bloqueio beta-adrenérgico no tratamento da ICFEr de etiologia da CCDC. 

 Análise de pequeno grupo de pacientes (n = 68) com IC de etiologia da DC do estudo REMADHE ^605^ comparou os que estavam em uso de betabloqueador com aqueles que não faziam uso do medicamento. Apesar da limitação inerente ao pequeno tamanho amostral para comparações diretas, segundo os autores, os resultados sugerem efeitos benéficos dos betabloqueadores relacionados ao aumento de sobrevida (valor de p não ajustado = 0,05, ou seja, limítrofe). Deve-se salientar que nesse estudo o uso de betabloqueador não foi randomizado, havendo alto risco de viés de confusão por indicação. 

 Sendo assim, julgamos que a evidência a respeito do uso de betabloqueador para pacientes com ICFEr de etiologia da DC é indireta, proveniente de estudos de ótima qualidade que testaram a eficácia desse tratamento nos tipos mais comuns de miocardiopatia (nível B). Acompanhando o racional de que fração de ejeção é um *continuum* prognóstico (ao invés de uma dicotomização), disfunções sistólicas de maior gravidade tendem a ser associadas a maior benefício absoluto. Portanto, a recomendação é definida como forte para pacientes com IC e FEVE ≤ 40% e ponderada para pacientes com ICFElr. 

 Um caso especial ocorre na presença de arritmia ventricular grave que requer considerar-se a prescrição de amiodarona. Eventualmente torna-se inadequada a associação de betabloqueador e amiodarona devido à bradicardia e/ou prolongamento do intervalo QT. Consideramos que nesse contexto não existe comprovação de que betabloqueador deva ser o medicamento prioritário. É o caso de se flexibilizar a decisão pelo julgamento clínico, cabendo ao médico decidir pelo medicamento inicial a ser prescrito, com base na gravidade da arritmia (favorece amiodarona) *versus* gravidade da IC (favorece betabloqueador). Esse é um momento raro em que a diretriz reconhece a limitação de recomendações estáticas e abre espaço para o dinamismo do pensamento médico baseado em racionalidade e em lastro de evidências (não confundir com conceitos de eficácia pretensamente baseados apenas, de forma ingênua e inconsequente, no famigerado “olho clínico”). 

***10.1.5.4. Espironolactona***

 Espironolactona é o antagonista preferencial do receptor de mineralocorticoide, sítio principal de ligação da aldosterona e responsável por suas ações fisiológicas e com envolvimento direto no tocante à fisiopatologia da IC. 

 De maneira geral, a espironolactona é indicada para todos os pacientes com IC sintomáticos e com FEVE ≤ 35%, a despeito do uso concomitante ou não dos IECA, BRA ou betabloqueadores, excetuando-se aqueles pacientes com creatinina sérica > 2,5mg/dL ou *clearance* de creatinina < 30mL/min/1,73m ^2^ , ou nível de potássio sérico > 5,0mEq/L. 

 O estudo que respalda essa indicação respondeu pelo acrônimo RALES, randomizado, duplo-cego, placebo-controlado, publicado em 1999, e testou se o uso de espironolactona, em dose variando de 25mg a 50mg, seria superior ao placebo na ICFEr (≤ 35%) e classe funcional III-IV, em uso concomitante de IECA e furosemida. ^606^ O estudo foi interrompido precocemente após 24 meses, com número de desfechos satisfatórios para indicar precisão e com a análise interina prevista demonstrando 35% de redução relativa do risco de morte. 

 Ressalte-se que pacientes com creatinina > 2,5mg/dL foram excluídos e a incidência de hipercalemia foi mínima nos dois grupos. Esse fato deve ser destacado, visto que estudo canadense de vigilância epidemiológica relatou que a taxa de prescrição da espironolactona elevou-se substancialmente após a publicação do estudo RALES e foi acompanhada de aumento na taxa de morbimortalidade associada à hipercalemia. ^607^ Portanto, respeitar os critérios de contraindicação para uso da espironolactona e vigilância judiciosa são essenciais na condução clínica de pacientes em uso desse fármaco. 

 Ainda que a CCDC tenha sido minimamente representada no estudo RALES (apesar de tal fato não ter sido especificado em sua Tabela de base), não há plausibilidade biológica para se questionar o benefício potencial do bloqueio da aldosterona quanto à progressão da ICFEr também nessa entidade nosológica. Portanto, consideramos uma boa aplicação do nível de evidência B (indireta de boa qualidade). Quanto à recomendação, guardadas as devidas indicações e contraindicações, consideramos deva ser forte para os pacientes com CCDC sintomática, FEVE ≤ 40%, creatinina ≤ 2,5mg/dL e potássio sérico ≤ 5,0mEq/dL e ponderada para pacientes com ICFElr. 

***10.1.5.5. Ivabradina***

 A ivabradina é um bloqueador seletivo da corrente If (canais funny) e, portanto, inibidor da atividade de MP no nó sinusal, resultando em redução seletiva da FC sem alterar parâmetros hemodinâmicos, como pressão arterial ou contratilidade miocárdica, e sem interferir na condução elétrica intracardíaca. 

 Na IC, o estudo que respalda o uso da ivabradina responde pelo acrônimo SHIFT. ^608^ Nesse ECR, duplo-cego, placebo-controlado, publicado em 2010, a ivabradina foi testada na dose máxima de 7,5mg 2 vezes ao dia em pacientes com IC (FEVE ≤ 35%), ritmo sinusal e FC> 70bpm, a despeito do uso de betabloqueadores quando tolerados. Relatou-se redução relativa do risco de hospitalização de 26% e mortalidade por IC também de 26%. 

 Em subestudo do SHIFT, ^215^ por análise *post-hoc* , avaliou-se desempenho de 38 pacientes com IC de etiologia da DC. Nessa subamostra, 20 pacientes tinham sido alocados para o grupo ivabradina e 18 para o grupo placebo. Apesar de os pacientes com CCDC apresentarem pior prognóstico em geral, com maior prevalência de BRD, menor nível de pressão arterial, maior taxa de uso de diuréticos, espironolactona, digoxina e menor taxa de uso de IECA/BRA ou betabloqueadores, comparativamente à população geral do estudo SHIFT, a ivabradina não foi associada a maior prevalência de bradicardia grave, BAV, hipotensão ou síncope. Ademais, relatou-se que a ivabradina foi eficaz em reduzir a FC desses pacientes e melhorar a classe funcional da IC. 

 A tradução das evidências para recomendação terapêutica não deve ser baseada em trabalhos exploratórios. É bastante claro que a etiologia da CCDC não foi bem representada no estudo SHIFT. Por outro lado, generalização não depende apenas de representatividade e não reconhecemos qualquer provável mecanismo de interação que nos faça suspeitar que a etiologia da CCDC modifique o efeito da terapia com ivabradina, a ponto de perda da eficácia demonstrada no conjunto geral dos pacientes incluídos no SHIFT. Por esse motivo, definimos que há nível de evidência B, o que representa evidência indireta de boa qualidade para uso de ivabradina em pacientes com CCDC e IC. Quanto à força de recomendação, essa deve ser ponderada, pois depende da percepção de que a FC esteja elevada na impossibilidade de aumento da dose do betabloqueador. Dada essa especificidade, optamos por não estender a indicação para pacientes com FEVE superior a 40%. 

***10.1.5.6. Digoxina***

 Ao revisar a literatura, não identificamos nenhum estudo avaliando a segurança e eficácia do medicamento nesse contexto específico. Portanto, utilizaremos evidência científica indireta de que a digoxina mostrou efeito para melhora sintomática e redução de internações hospitalares. ^609 , 610^ Na prática clínica, o medicamento pode ser indicado para pacientes em classe funcional III e IV da NYHA, a despeito do tratamento medicamentoso otimizado com os outros fármacos, e especialmente quando há FA com elevada resposta ventricular. 

 Com o digital, há bastante proximidade entre a dose terapêutica e a tóxica, elevando-se o potencial de efeitos adversos, devido ao acometimento do sistema excito-condutor, e ocasionando bradiarritmias, BAV e outras manifestações clínicas gerais. 

***10.1.5.7. Sacubitril-Valsartana***

 Sacubitril-valsartana é uma combinação medicamentosa composta por um fármaco inibidor da neprilisina (substância catalisadora da degradação dos peptídeos atriais natriuréticos), o sacubitril, em associação com um tradicional bloqueador da angiotensina II tipo-1, a valsartana. O principal estudo para validação científica dessa combinação medicamentosa foi o PARADIGM-HF, ^611^ que a comparou com enalapril. Embora esse possa ser considerado um estudo preciso e com baixo risco de viés, demonstrando redução relativa do risco de 20% com a associação medicamentosa para o desfecho combinado primário de hospitalização por IC e morte cardiovascular, houve margem para questionamento científico de sua concepção conceitual. ^612^ Com comparador heterodoxo, a rigor, o estudo não foi capaz de esclarecer se o benefício encontrado deveu-se à molécula inovadora (sacubitril) ou se decorreu de diferença inadequada quanto às doses dos inibidores tradicionais do sistema da angiotensina (a valsartana em dose diária maximizada de 320 mg *versus* enalapril em dose submáxima, talvez insuficiente, de 20mg ao dia). Outro aspecto a ressaltar, a existência de uma fase *run-in* em estudo de fase III, que superestima a aplicabilidade do tratamento, pois seleciona previamente os pacientes que toleram a terapia vasodilatadora mais intensa. 

 A partir da publicação do estudo PARADIGM-HF, passou a existir percepção por parte de muitos cardiologistas de que a combinação sacubitril-valsartana tenha eficácia superior à vasodilatação tradicional com IECA, o que tem influenciado recomendações de *guidelines* e diretrizes de IC. No Brasil, o uso da sacubitril-valsartana foi aprovado em maio de 2017 pela ANVISA (Agência Nacional de Vigilância Sanitária) e, em agosto de 2019, incorporado ao SUS. ^613^

 Há também indícios de que, diversamente do benefício homogeneamente verificado com inúmeros inibidores do sistema da angiotensina-II estudados, a combinação sacubitril-valsartana não se mostrou superior em outros contextos. Assim foi no estudo PARAGON-HF, ^614^ de pacientes com IC e FEVE ≥ 45%, e no estudo de IC complicando o infarto agudo do miocárdio que correspondeu ao acrônimo PARADISE-MI. ^615^ Em ambos os cenários, os resultados não foram capazes de rejeitar a hipótese nula configurada nas suas análises primárias. Vale ressaltar que o estudo PARADISE-MI foi o único que comparou o sacubitril-valsartana com dose adequada de IECA, no caso 10 mg/dia de ramipril. 

 Portanto, julgamos inadequada uma indicação baseada na expectativa de que essa combinação medicamentosa traga superioridade à terapia tradicional. Por outro lado, não há indícios de que essa terapia seja prejudicial, fazendo desse tratamento uma alternativa terapêutica válida, caso o médico deseje modificar um tratamento-padrão por motivo clínico ou logístico. É importante salientar que o relatório que respaldou a incorporação do sacubitril-valsartana no SUS estimou razão de custo-efetividade incremental de R$ 22.769 por ano de vida ganho com qualidade. ^613^

 Quanto à indicação de uso em pacientes com IC causada pela CCDC, além da técnica de revisão da literatura mencionada anteriormente, utilizou-se também a ferramenta do google acadêmico para buscar na literatura cinzenta alguma referência que pudesse trazer luz à questão de interesse aqui tratada e avaliamos os anais de congressos em busca dessa informação. 

 Assim, relatou-se série de pacientes com CCDC tratados com sacubitril-valsartana em hospital de referência para essa doença no Brasil, referindo-se, após 6 meses, melhora sintomática desses indivíduos. ^616^

 Em estudo prospectivo e observacional de 136 pacientes consecutivos com IC em único centro hospitalar universitário, incluindo as etiologias CMI, CCDC e cardiomiopatia idiopática, ^617^ os autores verificaram que até 44% dos pacientes desse registro unicêntrico apresentavam os principais critérios de exclusão do PARADIGM-HF. Observaram ainda que níveis pressóricos mais baixos, comuns na CCDC, poderiam ter levado à subutilização de alguns medicamentos nesse contexto. 

 Outro estudo avaliou a proporção de pacientes com CCDC randomizados em dois ensaios clínicos recentes (PARADIGM-HF e ATMOSPHERE), reportando que apenas 7,6% dos pacientes randomizados na América Latina tinham essa etiologia. ^592^

 Análise de subgrupo *post-hoc* do PARADIGM-HF sugeriu que o sacubitril-valsartana, em comparação com o enalapril, poderia levar a redução semelhante ou até maior (37%) de morte e hospitalização em pacientes com CCDC, comparativamente àqueles sem essa etiologia de IC, apesar de ausência de significância estatística e imprecisão de estimativa de efeito. ^618^ Sob o acrônimo PARACHUTE (ClinicalTrials.gov Identifier: NCT04023227), está em andamento estudo exclusivo de pacientes com IC de etiologia da CCDC. Infelizmente, como no próprio PARADIGM, ^611^ os comparadores não são os ortodoxos e o efeito de sacubitril associado à dose maximizada de valsartana será cotejado ao do enalapril em dose não máxima, de 20 mg diariamente. 

 Em síntese, fica claro que os pacientes com CCDC não foram bem representados nos estudos do sacubitril-valsartana. Então, embora tenhamos trazido algumas evidências a respeito da utilização da terapia em questão em pacientes com IC causada pela CCDC, elas não nos servem para induzir recomendação, pois são de caráter exploratório. Porém servem para exemplificar o princípio da evidência indireta: generalização não depende apenas de representatividade e não reconhecemos nenhum provável mecanismo de interação que nos faça suspeitar que a etiologia da cardiopatia modifique o efeito dessa terapia. Por esse motivo, definimos que há nível de evidência B para o paciente com ICFEr e CCDC, no sentido de que o tratamento com a combinação sacubitril-valsartana seja alternativa possível, porém não uma inovação superior ao tratamento tradicional. Quanto à recomendação, essa não é a de se preferir esse tratamento, mas apenas considerá-lo como alternativa quando o julgamento clínico sugere a necessidade de mudança terapêutica (recomendação ponderada). Também não estendemos essa indicação para paciente com FEVE > 40%. 

***10.1.5.8. Inibidores do Cotransportador de Sódio e Glicose do Tipo 2***

 Nos últimos anos, essa classe de medicamentos suscitou muito entusiasmo na comunidade científica a partir de demonstrações de benefício incremental ao tratamento tradicional, em termos de melhora do prognóstico da IC e da disfunção renal. Neste capítulo, revisaremos se o nível de evidências é proporcional ao entusiasmo e traduziremos para a tomada de decisão no contexto da IC da CCDC. 

 Os inibidores do cotransportador de sódio e glicose do tipo 2 (SGLT2) são medicamentos originalmente testados para tratamento de hiperglicemia em pacientes com diabetes *mellitus* do tipo 2. O SGLT2 age fisiologicamente no túbulo contornado proximal e responde por 90% da reabsorção da glicose filtrada no glomérulo. Os inibidores de SGLT2 promovem excreção renal de glicose, sendo esse o mecanismo de seu efeito redutor de glicemia. ^619^ Duas observações iniciais foram percebidas quanto a efeitos intermediários: primeiro, a eficácia desses inibidores como redutores de glicemia em diabéticos é modesta, com reduções médias de hemoglobina glicada variando entre 0,4% e 1,1%, em comparação ao placebo; ^619 - 621^ segundo, promovem consistente redução de peso, quando comparados a outros antidiabéticos. ^622 , 623^

 Diversamente do mais tradicional, a estratégia inicial dos produtores industriais dessa classe de medicamentos foi a de avaliar sua segurança em diabéticos, focando em desfechos macrovasculares (morte cardiovascular, infarto do miocárdio e AVC) e utilizando abordagem contraintuitiva de testar não inferioridade relativamente ao placebo. Embora contraintuitiva, o desvio da hipótese nula para um valor diferente de zero é método adequado para testar segurança, visto que um intervalo de tolerância para efeito adverso pode se justificar com base em um benefício demonstrado. 

 A não inferioridade em comparação ao placebo (segurança) foi confirmada por diversos estudos dessa classe de fármacos, ^624 - 626^ o que cientificamente tem validade. Por outro lado, restava a questão clínica: na ausência de um benefício incremental constatado, apenas a demonstração de segurança não seria justificativa para recomendar a adição desse tratamento para pacientes diabéticos? 

 Foi então que se percebeu que desfechos relacionados a IC aparentavam redução nos grupos tratados. Esses foram desfechos secundários dos estudos, exceto para o ensaio clínico DECLARE–TIMI 58, ^624^ onde compuseram desfecho de eficácia primário, caso a hipótese de não inferioridade para eventos graves fosse demonstrada. Vale salientar que inibidores de SGLT2 promovem natriurese, diurese osmótica (pela glicosúria) e perda de peso, mecanismos que aumentam a probabilidade *a priori* do benefício demonstrado. A partir desses resultados, investiu-se no teste da hipótese de que esses inibidores de SGLT2 melhorem o prognóstico em pacientes com ICFEr. 

 Assim, os ensaios clínicos com inibidores de SGLT2 voltaram-se para pacientes com IC sintomática, independentemente da presença de diabetes *mellitus* tipo 2. Os estudos DAPA-HF ^627^ e EMPEROR-Reduced ^628^ avaliaram o efeito de dapagliflozina e empagliflozina, respectivamente, na incidência de desfecho combinado de morte por causa cardiovascular e internação por IC, em comparação ao placebo, nos pacientes com ICFEr. O primeiro a ser publicado, o estudo DAPA-HF, incluiu 4.744 pacientes com IC e FEVE ≤ 40%, em classe funcional II a IV (NYHA), já em uso de terapia farmacológica otimizada, e elevação dos níveis de NT-proBNP. Diabetes *mellitus* estava presente em 42% da amostra e 99% dos casos estavam em classe funcional II ou III na randomização. A etiologia da IC foi não isquêmica em 44% dos casos, sem menção a DC, embora esse tenha sido um ensaio clínico multicontinental, no qual cerca de 17% dos participantes foram recrutados em centros da América Latina. ^627^

 Os pacientes foram randomicamente alocados para uso de dapagliflozina 10 mg/dia ou placebo, em razão 1:1. Após seguimento mediano de 18 meses, dapagliflozina associou-se a redução do risco para o desfecho primário, que incluía morte de causa cardiovascular e internação por IC (386 *versus* 502 eventos, respectivamente; HR 0,74; IC 95%: 0,65-0,85). O benefício foi observado em ambos os componentes do desfecho primário, bem como mostrou-se consistente nas análises pré-especificadas em diferentes subgrupos, inclusive conforme a presença ou não de diabetes *mellitus* tipo 2. ^627^ Observou-se também redução no risco de morte por todas as causas no grupo tratado com dapagliflozina *versus* o grupo placebo (276 vs 329; HR, 0,83; IC 95%: 0,71-0,97). 

 Um subestudo do DAPA-HF avaliou mais detalhadamente a potencial influência da etiologia da IC, classificada como isquêmica e não isquêmica (causa hipertensiva, idiopática, “outras” e causa desconhecida), quanto ao benefício da dapagliflozina sobre o desfecho primário e não encontrou modificação de efeito. ^629^

 O perfil de segurança da dapagliflozina foi satisfatório, com baixa incidência de eventos adversos sérios. É importante salientar, porém, que, na avaliação de elegibilidade do DAPA-HF, pressão arterial sistólica < 95mmHg e taxa de filtração glomerular < 30mL/min/1,73m ^2^ constituíram critérios de exclusão para participação no estudo. 

 O estudo correspondente ao acrônimo EMPEROR-Reduced, publicado em 2020, ^628^ investigou o efeito da empagliflozina, comparada a placebo, em amostra de pacientes com ICFEr (≤ 40%) em terapia médica otimizada, e definiu perfil de elegibilidade e desfecho primário semelhantes aos do ensaio DAPA-HF. No entanto, os 3.730 participantes do estudo (50% com diabetes *mellitus* tipo 2) apresentaram valores médios mais altos de peptídeos atriais natriuréticos e média de FEVE mais baixa, em relação à amostra do estudo da dapagliflozina. Novamente, a DC não foi representada como etiologia da IC, ainda que 34% dos participantes do estudo tivessem sido recrutados em países da América Latina. Após mediana de seguimento de 16 meses, empagliflozina reduziu em 25% o risco combinado de internação por IC e morte cardiovascular em relação ao placebo (19,4% *versus* 24,7%; HR 0,75; IC 95%: 0,65-0,86), porém, diferentemente do DAPA-HF, esse benefício pareceu decorrer basicamente da redução de internações por IC. Nas análises de subgrupo pré-especificadas, o efeito da empagliflozina para o desfecho primário manteve-se consistente. ^628^

 Assim como observado no DAPA-HF, no EMPEROR-Reduced, pacientes em uso do inibidor de SGLT2 evoluíram com menores valores de pressão arterial sistólica, peso corporal e NT-proBNP após um ano de seguimento, em comparação aos valores basais. 

 Mais recentemente, o EMPEROR-Preserved trial ^630^ estendeu a investigação com empagliflozina para pacientes com ICFElr (> 40%). A redução relativa do risco de eventos foi semelhante àquela verificada nos pacientes com FEVE ≤ 40%, o que é esperado, pois um limite arbitrário de fração de ejeção não define duas doenças diferentes. Cabe enfatizar que pacientes com ICFElr possuem melhor prognóstico, o que naturalmente reduz a magnitude absoluta do benefício: NNT de 19 nos dois primeiros estudos com FEVE < 40% e NNT de 30 no EMPEROR-Preserved trial. 

 Esses estudos possuem precisão estatística satisfatória e baixo risco de viés, parecendo, portanto, adequado afirmar que existe efeito de benefício, cuja magnitude representada por 25% de redução relativa do risco se situa no nível (marginal) da maioria das terapias reconhecidas em IC. Portanto, do ponto de vista pragmático, esses fármacos são seguros e moderadamente benéficos. 

 Quanto à custo-efetividade, recente incorporação da dapagliflozina no SUS baseou-se em relatório da CONITEC que apresenta modelo econômico com razão de custo-efetividade incremental da ordem de R$ 9.296 por ano de vida salva com qualidade e situa-se dentro de uma definição aceitável para eficiência 

 No entanto, resta uma questão conceitual: o quanto do benefício desses fármacos deriva do aprimoramento da terapia diurética *versus* o quanto deve-se especificamente à inovação da molécula? Há descrição de efeitos favoráveis dessas medicações em desfechos intermediários, de ordem metabólica e neuro-humorais, como aumento de níveis circulantes de substâncias vasodilatadoras e redução dos níveis de vasoconstritores. Todavia, os ensaios clínicos não focalizaram a pertinente prova de conceito de que são esses os efeitos que medeiam o benefício clínico no contexto. Nenhum deles gerou um contrafactual (segundo grupo controle) baseado na terapia diurética para responder à questão: se um paciente que não recebesse o fármaco inovador tivesse tido um mesmo nível de melhora da diurese, o seu desfecho seria diferente? Essa pergunta também poderia ser explorada por análise de mediação (inferência causal), utilizando-se dados dos ensaios clínicos e de uma variável mediadora pós-randomização que representasse o efeito na diurese. Não detectamos na literatura esse tipo de abordagem. 

 Finalmente, como traduzir nossa interpretação das evidências para recomendação de terapêutica com gliflozinas em indivíduos com IC de etiologia da DC? De novo, essa não foi uma subpopulação representada nos ensaios clínicos. Consoante o já exposto para outros contextos, generalização não depende apenas de representatividade e não reconhecemos um provável mecanismo de interação que nos faça suspeitar que a etiologia da CCDC modifique o efeito dessa terapia a ponto de perda da eficácia demonstrada. Por esse motivo, definimos que há nível de evidência B para a IC causada pela CCDC, ou seja, ela é indireta e de boa qualidade. Quanto à força de recomendação, na ausência do contrafactual de que o benefício exista além do efeito diurético, optamos por uma recomendação ponderada para pacientes com IC cursando com FE reduzida, devendo a justificativa para esta nova prescrição ser mediada por um quadro clínico que sugira necessidade de incremento terapêutico. 

**Figura 10.1:**
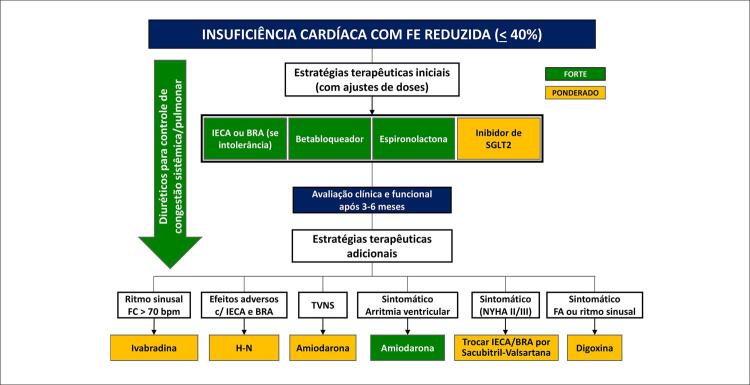
– Algoritmo para o tratamento farmacológico de pacientes com insuficiência cardíaca e fração de ejeção reduzida.

BRA: bloqueador de receptor de angiotensina II; FA: fibrilação atrial; FC: frequência cardíaca; FE: fração de ejeção; H-N: hidralazina-nitrato; IECA: inibidor da enzima de conversão da angiotensina; NYHA: New York Heart Association; SGLT2: cotransportador de sódio-glicose tipo 2; TVNS: taquicardia ventricular não sustentada.

 As recomendações para o tratamento farmacológico da IC na CCDC estão expressas na Tabela 10.1 e nas Figuras 10.1 e 10.2 . 

**Tabela 10.1:** – Recomendações para o manuseio farmacológico da insuficiência cardíaca na CCDC

Insuficiência Cardíaca com Fração de Ejeção Reduzida (ICFEr): ≤ 40%	Grau de recomendação	Nível de evidência
Diurético de alça para controle de congestão sistêmica ou pulmonar	Forte	C
Diurético tiazídico associado ao diurético de alça para controle de congestão sistêmica ou pulmonar persistente	Forte	C
IECA para reduzir morbidade e mortalidade	Forte	B
BRA nos pacientes intolerantes a IECA por tosse/angioedema para reduzir morbidade e mortalidade	Forte	B
Sacubitril-valsartana em substituição ao IECA/BRA para pacientes já em uso de terapêutica tripla otimizada, que permanecem sintomáticos (NYHA II ou III) para reduzir morbidade e mortalidade	Ponderado	B
Carvedilol, succinato de metoprolol ou bisoprolol para reduzir morbidade e mortalidade em pacientes hemodinamicamente estáveis	Forte	B
Espironolactona associada ao tratamento-padrão com IECA (ou BRA) e BB para reduzir morbidade e mortalidade (CCDC sintomática, creatinina ≤ 2,5mg/dL e potássio sérico ≤ 5,0 mEq/dL)	Forte	B
Associação de hidralazina e nitrato nos pacientes com contraindicação ao IECA/BRA (insuficiência renal e/ou hipercalemia) para redução de morbidade e mortalidade	Ponderado	B
Ivabradina para pacientes com terapêutica otimizada, em ritmo sinusal e com FC maior que 70 bpm para redução de morbidade e mortalidade	Ponderado	B
Inibidores de SGLT2 (dapagliflozina ou empagliflozina) para pacientes diabéticos ou não, com terapêutica tripla otimizada, para redução de desfechos cardiovasculares e progressão de disfunção renal	Ponderado	B
Digoxina para pacientes sintomáticos com FA e resposta ventricular elevada, apesar do uso de BB, para reduzir sintomas e hospitalizações	Ponderado	B
Digoxina para pacientes sintomáticos em ritmo sinusal, apesar de terapêutica tripla otimizada, para reduzir sintomas e hospitalizações	Ponderado	B
**Insuficiência Cardíaca com Fração de Ejeção Levemente Reduzida (ICFElr): 41-54%**	**Grau de recomendação**	**Nível de evidência**
Diurético de alça para controle de congestão sistêmica ou pulmonar	Forte	C
Diurético tiazídico associado ao diurético de alça para controle de congestão sistêmica ou pulmonar persistente	Forte	C
IECA para reduzir morbidade e mortalidade	Ponderado	B
BRA nos pacientes intolerantes a IECA por tosse/angioedema para reduzir morbidade e mortalidade	Ponderado	B
Carvedilol, succinato de metoprolol ou bisoprolol para reduzir morbidade e mortalidade	Ponderado	B
Espironolactona associada ao tratamento padrão com IECA (ou BRA) e BB para reduzir morbidade e mortalidade	Ponderado	B
Digoxina para pacientes sintomáticos com FA e resposta ventricular elevada, apesar do uso de BB, para reduzir sintomas e hospitalizações	Ponderado	B

BB: betabloqueador; BRA: bloqueador de receptor de angiotensina II; CCDC: cardiomopatia crônica da doença de Chagas; FA: fibrilação atrial; FC: frequência cardíaca; IECA: inibidor da enzima de conversão da angiotensina; NYHA: New York Heart Association; SGLT2: cotransportador de sódio-glicose tipo 2.

**Figura 10.2:**
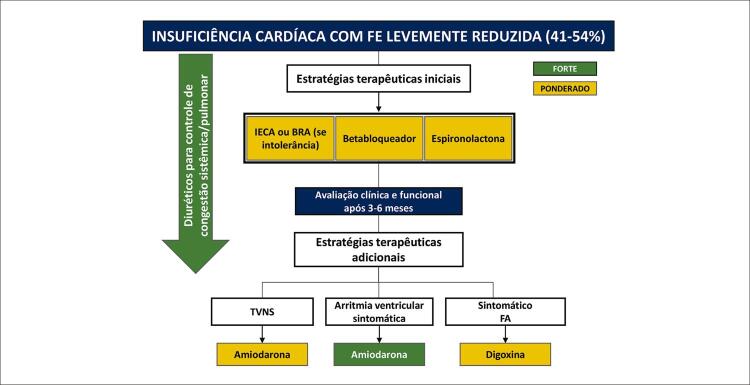
– Algoritmo para o tratamento farmacológico de pacientes com insuficiência cardíaca e fração de ejeção levemente reduzida. BRA: bloqueador de receptor de angiotensina II; FA: fibrilação atrial; IECA: inibidor da enzima de conversão da angiotensina; TVNS: taquicardia ventricular não-sustentada.

## 10.2. Recursos Não Farmacológicos

### 10.2.1. Transplante Cardíaco

 Apesar dos avanços observados no tratamento medicamentoso, nos cuidados de terapia intensiva e nas estratégias cirúrgicas, inclusive com uso de dispositivos cardíacos implantáveis para tratamento da IC, essa síndrome clínica ainda persiste com elevada morbidade e mortalidade e considerável impacto econômico sobre o sistema de saúde, principalmente em suas fases mais avançadas. ^631^

 O TC ainda é reconhecido como a melhor forma de tratamento para a IC refratária, com influência evidente no aumento de sobrevida e melhora da qualidade de vida dos pacientes, especialmente na CCDC, que têm prognóstico mais reservado quando comparada às outras etiologias. ^632 - 634^ Portanto, o tratamento com TC na CCDC avançada é considerado uma recomendação forte, com nível de evidência B, à semelhança do que ocorre em outras doenças cardíacas com indicações clássicas, desde que, obviamente, não haja contraindicações ao procedimento e que se considerem algumas peculiaridades, tais como, condições socioeconômicas desfavoráveis e presença de megacólon e/ou megaesôfago, que podem aumentar os riscos de complicações no pós-operatório e comprometer o resultado do TC. ^20^

***10.2.1.1. Estratégias de Imunossupressão***

 Os regimes imunossupressores instituídos após o TC podem ser classificados como de indução e de manutenção e independem da etiologia da IC que resultou na indicação do TC. Os regimes de indução propiciam intensa supressão imunológica pós-operatória precoce, enquanto os regimes de manutenção são usados ao longo da vida do paciente para prevenir a rejeição. ^635 , 636^

***10.2.1.2. Terapia de Indução***

 A terapia de indução no paciente transplantado consiste no tratamento imunossupressor de forma intensa, durante o transplante ou no pós-operatório imediato, sendo utilizada em pacientes de alto risco para rejeição na tentativa de reduzir o risco agudo desse evento ou de retardar o uso de doses maiores de inibidores da calcineurina, minimizando o dano renal, particularmente em pacientes com função renal comprometida. ^635^ São considerados de alto risco de rejeição fatal, podendo, portanto, beneficiar-se da terapia de indução os pacientes com altos títulos de anticorpos anti-HLA no painel imunológico (PRA = panel reactive antibody > 10%), sendo considerados mais vulneráveis: mulheres jovens com história prévia de gravidez, pacientes com transfusões pregressas múltiplas e usuários de suporte circulatório mecânico. Os principais agentes indutores são as imunoglobulinas antitimócitos policlonais (anticorpo policlonal - timoglobulina) e os inibidores dos receptores de IL-2, os quais têm baixa imunogenicidade, como o daclizumabe e basiliximabe. ^635^

 Embora esses agentes possam reduzir o risco de rejeição precoce e/ou minimizar o dano renal, estão associados a risco aumentado de infecção e, portanto, têm potencial para reativar a infecção pelo *T. cruzi* . A terapia de indução ainda é controversa e, apesar de ser utilizada em 50% dos receptores cardíacos em geral, não foram realizados, até o momento, grandes ECR demonstrando o benefício da terapia de indução *versus* nenhuma terapia de indução. ^636 , 637^ Não existem dados disponíveis acerca de seus efeitos no receptor com CCDC. 

***10.2.1.3. Terapia de Manutenção***

 A terapia imunossupressora básica de manutenção nos pacientes transplantados cardíacos, em geral, inclui necessariamente um agente inibidor de calcineurina, qual seja a ciclosporina A ou o tacrolimus. Esses agentes devem ser associados ao micofenolato de mofetil (MMF) ou micofenolato sódico ou azatioprina ou rapamicina ou everolimus. A prednisona também é associada a esse esquema-padrão, sendo que, na maioria dos pacientes, pode e deve ser suspensa cerca de 6 meses após o transplante, na ausência de rejeição. ^635^

 No contexto da CCDC, a terapia imunossupressora de indução e/ou de manutenção pode reativar a infecção pelo *T.cruzi* . ^2 , 20^ Não existem estudos comparando os vários esquemas de imunossupressão nos pacientes com CCDC; entretanto, um maior número de reativações foi diagnosticado com uso de MMF *versus* azatioprina. ^638^ Portanto, estratégias para alterar a imunossupressão, como a substituição do MMF pela azatioprina ou a redução da dose do MMF, têm sido propostas, mas essas estratégias não foram testadas em ECR. 

 Uma redução precoce dos agentes imunossupressores, especialmente corticosteroides, é recomendada para prevenir a RDC, mas essa abordagem pode facilitar os episódios de rejeição. Sendo assim, seria recomendável que o paciente com CCDC receba a terapia imunossupressora com a menor intensidade possível, desde que não tenha rejeição. ^2 , 20^

 A Tabela 10.2 resume as estratégias de imunossupressão após TC no contexto da DC. 

**Tabela 10.2:** – Recomendações relacionadas à terapia imunossupressora em receptores de TC com CCDC

Sumário de recomendações	Grau de recomendação	Nível de evidência
A terapia de indução só deve ser utilizada em pacientes de alto risco para rejeição aguda e ou piora da função renal: PRA > 10%; função renal reduzida; jovens com gravidez prévia; múltiplas transfusões prévias; usuários de SCM	Forte	C
Utilizar imunossupressores na menor dose possível	Forte	B
Preferir a utilização de azatioprina ou micofenolato em dose reduzida em associação com um inibidor de calcineurina (CYA ou Tacrolimus)	Ponderado	B
Na ausência de rejeição, suspender os corticosteroides gradativamente, após 6 meses do TC	Ponderado	C
Na presença de rejeição, reajustar o esquema e a dose de imunossupressores	Forte	B

CCDC: cardiopatia crônica da doença de Chagas; CYA: ciclosporina A; PRA: panel reactive antibody; SCM: suporte circulatório mecânico; TC: transplante cardíaco.

### 10.2.2. Diagnóstico e Tratamento da Rejeição

 A incidência de rejeição com necessidade de tratamento vem se reduzindo progressivamente ao longo dos anos, acometendo apenas 12,6% dos receptores no primeiro ano após o TC na atualidade. ^636^ A rejeição é classificada em hiperaguda, mediada por anticorpos, e rejeição celular aguda, que representa a forma mais prevalente de rejeição. Histologicamente, é definida por infiltrados inflamatórios, em que tipicamente predominam linfócitos, e lesão associada aos miócitos. A *International Society for Heart & Lung Transplantation* (ISHLT) revisou as categorias de rejeição celular aguda (R) como segue: 0R (sem rejeição), 1R (leve), 2R (moderado) ou 3R (grave). ^639^

 A rejeição hiperaguda constitui evento pouco comum, é mediada por anticorpos pré-formados nos receptores e se manifesta como uma falência grave do enxerto dentro de minutos ou poucas horas após o procedimento de TC. ^639^ A frequência de rejeição hiperaguda e rejeição mediada por anticorpos após TC devido à DC ainda não foi relatada. 

 A rejeição celular aguda ocorre em 10% a 14% dos receptores com CCDC e não há diferença na incidência de episódios de rejeição celular aguda (grau 2R ou 3R) entre receptores de TC com ou sem DC. ^636 , 639 - 643^

 A biópsia endomiocárdica ainda constitui o método-padrão para o diagnóstico de rejeição, sendo a frequência das biópsias variável conforme o protocolo do centro de transplante. Pode ocorrer miocardite secundária à reativação da infecção pelo *T. cruzi* no coração transplantado, o que torna o diagnóstico diferencial entre rejeição e RDC um grande desafio. ^390 , 635 , 643 , 644^

 A definição de uma dessas duas condições ainda é difícil se parasitas não forem encontrados nos fragmentos de biópsia. De acordo com as técnicas de coloração histopatológica de rotina, se os parasitas não forem vistos, as características histopatológicas inflamatórias encontradas na rejeição (grau 2R ou 3R) ou na RDC são bastante semelhantes. Assim, a detecção de infiltrado mononuclear inflamatório nas lâminas de biópsia endomiocárdica não é suficiente para descartar o diagnóstico de RDC e representa um dilema médico, pois o tratamento imunossupressor agressivo para abortar a rejeição pode facilitar e intensificar a RDC. ^390 , 644^ A presença de ninhos de amastigotas de *T. cruzi* com infiltrados mononucleares inflamatórios nos fragmentos de biópsia endomiocárdica não exclui rejeição concomitante do enxerto, pois as duas condições podem ocorrer simultaneamente. ^390 , 644^

 A terapia de rejeição em transplantados com e sem DC é semelhante. Em geral, o grau leve de rejeição (1R), na ausência de comprometimento clínico ou hemodinâmico, não requer intervenção adicional. No entanto, graus mais elevados (≥ 2R) requerem terapia imunossupressora suplementar agressiva. ^635 , 636^ A rejeição constitui um fator de risco para a RDC, sendo que mais de 85% dos pacientes apresentam pelo menos um episódio de rejeição antes de ocorrer a reativação. ^645^

### 10.2.3. Diagnóstico e Tratamento da Reativação da Infecção pelo T. cruzi 

***10.2.3.1. Apresentação Clínica***

 A terapia imunossupressora instituída aumenta o risco de reativação da infecção pelo *T. cruzi* , cuja incidência após TC varia de 19,6% a 90%. ^2 , 638 , 642 , 643 , 646 - 649^ Considerando a morbidade e a mortalidade potencial, o diagnóstico e manejo apropriado da RDC no contexto de transplante de órgãos é extremamente importante. 

 Portanto, esse procedimento deve ser realizado dentro de um protocolo clínico e laboratorial estruturado para monitorar a reativação da infecção e seu subsequente tratamento. ^2 , 20 , 648 - 651^ O diagnóstico da reativação baseia-se em sinais e sintomas clínicos e na presença de parasitos em sangue, líquor e outros fluidos, medula óssea ou tecidos. ^2^

 A monitoração tem como objetivo identificar os primeiros sinais de reativação e estabelecer tratamento anti- *T. cruzi* prontamente. A reativação clínica tem manifestações cardíacas e extracardíacas incluindo: miocardite, disfunção ventricular, arritmias, bloqueios atrioventriculares/intraventriculares novos no ECG, lesões cutâneas (nódulos subcutâneos, paniculite), febre, acometimento de medula óssea ou manifestações neurológicas, tais como meningoencefalite, chagoma, abcesso cerebral ou AVC. ^2 , 640 , 642 , 643 , 651 - 653^ A miocardite da reativação pode ser equivocadamente diagnosticada como rejeição do enxerto e tratada com intensificação do tratamento imunossupressor, o que vai agravar a reativação. ^644^ O diagnóstico diferencial entre a miocardite da rejeição e da reativação ainda constitui um grande desafio. ^390 , 644 , 654^ Na presença de infiltrado inflamatório, ninhos de amastigotas e/ou PCR positiva para *T. cruzi* no miocárdio, podemos afirmar que existe reativação, mas não é possível excluir, com segurança, rejeição do enxerto associada. Apesar dessa complexidade, a taxa de sobrevida de receptores com CCDC submetidos ao TC não difere das de outras etiologias. ^2 , 636 , 640 , 643^

***10.2.3.2. Diagnóstico Parasitológico da Reativação***

 O objetivo da monitoração laboratorial é identificar qualquer sinal subclínico de RDC antes dos sintomas cardíacos e extracardíacos, bem como de disfunção do enxerto. ^2 , 649 - 651 , 655^ As provas sorológicas têm utilidade somente em potenciais doadores, diagnóstico de CCDC em potenciais receptores e em receptores soronegativos que recebem órgãos de doadores soropositivos. ^2^ Não têm papel no diagnóstico da RDC. 

 Tradicionalmente, a monitoração laboratorial utilizava métodos parasitológicos (pesquisa direta do *T. cruzi* e hemoculturas) e exames histológicos seriados de biópsia endomiocárdica, na procura de amastigotas de *T. cruzi,* testes esses com baixa sensibilidade. ^2^ Nos últimos anos, vários estudos demonstraram o valor do teste da PCR no sangue periférico e no miocárdio para detectar RDC precoce antes do surgimento de sintomas e/ou disfunção do enxerto. ^388 , 390 , 391 , 401 , 403 , 656 - 658^

 Quanto à frequência das visitas clínicas e da monitoração laboratorial, ainda não há consenso na literatura. A Tabela 10.3 constitui sugestão de um protocolo de monitoração clínica, laboratorial e histológica para pacientes com CCDC submetidos ao TC e ainda sugestão de tratamento etiológico com base nas principais diretrizes disponíveis. ^2 , 20 , 647 , 650 , 651^

**Tabela 10.3:** – Monitoração clínica, histológica e laboratorial da reativação da infecção pelo T. cruzi após TC na doença de Chagas e tratamento etiológico

Procedimento	Grau de recomendação	Nível de evidência
**Antes do transplante**		
Testes sorológicos para doença de Chagas em doador	Forte	C
Testes sorológicos para doença de Chagas em potencial receptor com alguma possibilidade de CCDC	Forte	C
**Após o transplante**		
Consultas clínicas periódicas com atenção para sinais/sintomas de reativação, incluindo realização de ECG e ecocardiograma	Forte	C
Pesquisa de *T. cruzi* de rotina no sangue (esfregaço, hemocultura) para diagnóstico de reativação da infecção	Forte	C
Pesquisa de *T. cruzi* de rotina no sangue por PCR	Forte	C
Biópsias endomiocárdicas periódicas de rotina, com pesquisa de *T. cruzi* (histologia e imunohistoquímica)	Forte	C
Biópsias endomiocárdicas periódicas de rotina, com pesquisa de *T. cruzi* por PCR, se disponível	Forte	C
Pesquisa de *T. cruzi* em tecidos (pele, medula, etc) em quadro compatível com reativação da infecção por *T. cruzi*	Forte	C
Frequência dos procedimentos após o transplante:	Ponderado	C
• Primeiro mês: semanal • Segundo mês: a cada duas semanas • Terceiro ao sexto mês: mensal • Sétimo ao 12º mês: a cada 3 meses • Após 12 meses: a cada 6 meses		
**Tratamento etiológico da reativação**		
Benznidazol 5mg/Kg/dia durante 60 dias	Forte	C

CCDC: cardiopatia crônica da doença de Chagas; PCR: polymerase chain reaction; TC: transplante cardíaco.

***10.2.3.3. Tratamento Etiológico da Reativação***

 Na presença de sinais/sintomas e/ou identificação do parasito no sangue, líquor ou tecido, recomenda-se iniciar tratamento etiológico imediatamente. ^2 , 20^ O benznidazol é o medicamento recomendado como tratamento de primeira linha. ^2 , 563^ Os comprimidos têm 100mg da substância ativa. Sua absorção se dá pelo trato gastrintestinal, sendo a excreção predominantemente renal, com meia vida de 12 horas. A dose recomendada é de 5mg/kg/dia, por 60 dias de tratamento, sendo a dose diária dividida em duas ou três vezes. ^2 , 563^ Seu efeito colateral mais importante é a dermatite, do tipo urticariforme, que ocorre em cerca de 30% a 60% dos pacientes, já no final da primeira semana de tratamento, mas apresentando boa resposta terapêutica com o uso de anti-histamínicos ou pequenas doses de corticosteroides. Poucos são os casos que se acompanham de febre e adenomegalia, quando a medicação deve ser suspensa. Outros efeitos adversos incluem polineuropatia (mais tardia), com dor e/ou parestesia em membros inferiores, anorexia. Leucopenia significativa e agranulocitose são raras e, quando presentes, determinam interrupção do tratamento. ^2 , 563^ O nifurtimox não está disponível rotineiramente no Brasil. Essas medicações tripanossomicidas estão contraindicadas em gestantes e pacientes com insuficiência renal ou hepática importante. ^2^ Não existe evidência suficiente que suporte a estratégia de tratamento anti *T. cruzi* profilático da RDC. É importante considerar que esses fármacos têm efeitos colaterais importantes, nem todo receptor é acometido de RDC e que um paciente pode ter mais de um episódio de RDC após tratamento. Recomenda-se manter a monitoração da reativação mesmo após tratamento anti *T. cruzi* . ^2 , 20 , 650 , 651^ ( Tabela 10.3 ) 

***10.2.3.4. Complicações Pós-Transplante Cardíaco e Sobrevivência***

 Os desfechos clínicos, morbidade e mortalidade em receptores de TC com e sem DC são semelhantes. ^636 , 640 - 642^ Em ambas as classes de pacientes, as principais complicações relatadas após o TC são praticamente as mesmas: disfunção do enxerto (20%); rejeição 2R ou 3R (10%-14%); sangramento no pós-operatório (10%); infecção não relacionada ao *T. cruzi* (20%-30%); e insuficiência renal aguda (até 70%). Por sua vez, em receptores com CCDC, a doença vascular coronária do enxerto parece ser menos frequente, enquanto a incidência de neoplasias parece ser maior, embora nenhuma dessas diferenças relatadas tenha sido confirmada em todas as séries. ^584 , 640 - 642 , 659^

 Apesar de toda a complexidade da DC no contexto de TC, os resultados finais são bons. No Brasil, a taxa de sobrevida de pacientes com CCDC submetidos ao TC é de 76%, 71% e 46% em 6 meses, 5 e 10 anos, respectivamente, superior àquela da coorte de pacientes submetidos ao TC por outras etiologias. ^641 , 642^ Uma explicação para esse melhor desempenho seria que, em decorrência de características de base, os pacientes com CCDC, em geral, são mais jovens, com menos comorbidades e menos cirurgias cardíacas prévias. ^584 , 640 - 642^ Em aparente contraste com esses dados, publicação recente relatou a evolução de 376 transplantados entre 1997 e 2019 em única instituição do Nordeste brasileiro, comparando as seguintes etiologias de IC: CCDC, CMI e cardiomiopatia não isquêmica em geral. Evidenciou-se, após seguimento médio de 5 anos, estabilidade na sobrevida dos indivíduos com CCDC, enquanto ocorreu melhora subsequente desse parâmetro nos outros dois grupos. ^660^

### 10.2.4. Assistência Circulatória Mecânica

 Os dispositivos de assistência circulatória mecânica (DACM) são utilizados para restaurar a perfusão tecidual em pacientes com IC avançada ou choque cardiogênico, refratários à terapia clínica otimizada, incluindo o uso de medicações inotrópicas. Podem oferecer suporte ao VE, ao VD, ou a ambos. ^661^

 Os DACM podem ser indicados como ponte para o TC ou como ponte para recuperação, quando há perspectiva de melhora da função ventricular após insulto agudo, ou ainda como ponte para decisão em pacientes críticos, na incerteza quanto à probabilidade de melhora do quadro clínico. Em situações específicas, sobretudo na presença de contraindicação ao TC, os DACM podem ser utilizados como terapia de destino. ^21^

 As evidências sobre o uso de DACM em pacientes com DC limitam-se a poucos relatos ou séries de casos na literatura, predominantemente como estratégia de ponte para transplante. ^662 - 666^ Os critérios de indicação e contraindicação à utilização dos DACM em pacientes com DC podem ser os mesmos utilizados para outras etiologias. ^661^ Embora a disfunção sistólica ventricular direita seja relativamente comum em pacientes com CCDC, em especial nos indivíduos que também apresentam disfunção sistólica do VE, não há consenso quanto à escolha do tipo de dispositivo mais apropriado nessa condição. ^667^

 O primeiro relato da utilização bem-sucedida de um DACM como ponte para transplante em paciente com CCDC ocorreu em 1994. ^662^ Posteriormente, em ensaio clínico não controlado de fase I, incluindo seis pacientes com IC biventricular avançada, Moreira *et al* . reportaram sucesso com a utilização de DACM esquerda como ponte para transplante em apenas dois casos. ^663^

 Em países desenvolvidos, o DACM tem sido implantado em imigrantes com IC por DC. A utilização de DACM biventricular em pacientes com DC foi relatada por Kransdorf *et al* . em dois indivíduos de uma coorte de pacientes submetidos a TC nos EUA. ^664^ Ruzza *et al* . descreveram um caso bem-sucedido de coração artificial total como ponte para transplante em paciente com CCDC. ^665^ Na Holanda, foi implantado DACM como ponte para TC em paciente com IC refratária de etiologia da DC. ^666^ Mais recentemente, Atik *et al* . relataram outro caso de sucesso utilizando DACM esquerda de fluxo axial em paciente com DC e disfunção sistólica biventricular. ^667^

 Em geral, considera-se que o suporte cardíaco mecânico tem elevado potencial de êxito como estratégia de ponte para transplante, recuperação, tomada de decisão ou terapia de destino em pacientes com CCDC. Contudo, atualmente, as maiores limitações à sua aplicabilidade são o alto custo, a disfunção de VD e a necessidade de equipe especializada para o implante e manejo dos dispositivos. A Diretriz de Assistência Circulatória Mecânica da SBC recomenda avaliação criteriosa da função de VD como mandatória antes do implante, sendo que, na presença de disfunção moderada a importante, deve-se estar preparado para o implante de suporte biventricular. ^661^

 Os principais índices para avaliar dimensões e função de VD são: avaliação semiquantitativa da contratilidade longitudinal e radial do VD, cálculo da variação fracional da área, deslocamento sistólico do plano do anel tricúspide (TAPSE) pelo modo M, velocidade sistólica máxima do anel tricúspide lateral estimado pelo Doppler tecidual (s’) e índice de performance do VD. O implante de DACM univentricular esquerdo é motivo de restrições em pacientes com CCDC e dilatação importante do VD, insuficiência tricúspide moderada a grave, anel da válvula tricúspide > 45mm e pressão venosa central (PVC) > 15mmHg. ^661^ Os parâmetros hemodinâmicos considerados ótimos em relação à função ventricular direita e que reduziriam o risco de disfunção de VD após implante são: PVC ≤ 8mmHg, pressão capilar pulmonar (PCP) ≤ 18mmHg, PVC/PCP ≤ 0,66, resistência vascular pulmonar < 2 UW e trabalho indexado de VD ≥ 400mL/m ^2^ . ^661^

## 11. CONDUTAS TERAPÊUTICAS NAS ARRITMIAS CARDÍACAS

### 11.1. Recursos Farmacológicos

#### 11.1.1. Introdução

 A literatura médica relacionada ao tratamento das arritmias e prevenção de morte súbita na CCDC é relativamente escassa e insuficiente para a formulação de recomendações fortes que se apoiem em evidências diretamente obtidas em estudos aleatorizados, indiscutivelmente comprovando eficácia terapêutica (nível A de evidência). ^1 , 668^ No entanto, a CCDC compartilha várias similaridades com diversas cardiopatias extensamente estudadas, em particular aquelas que cursam com fibrose miocárdica e disfunção sistólica (global ou segmentar), tais como a CMI e a CMD, ^669^ permitindo que o raciocínio terapêutico siga bases fisiopatológicas semelhantes. Dessa forma, o tratamento e a prevenção das arritmias ventriculares e supraventriculares na CCDC tendem a seguir, em linhas gerais, orientações semelhantes às das demais cardiopatias. 

 Por outro lado, vale destacar que algumas particularidades específicas, que podem influenciar o tratamento antiarrítmico, costumam ser mais marcantes na CCDC. Disfunção do nó sinusal, distúrbios da condução atrioventricular e intraventricular e arritmias ventriculares são frequentemente encontrados tanto em pacientes assintomáticos como nas formas mais avançadas da doença. ^669 - 672^ Embora exista a clássica relação direta entre o grau de disfunção ventricular e a maior frequência de arritmia ventricular, a prevalência de arritmias ventriculares na CCDC é maior quando comparada às de outras cardiopatias. ^672 , 673^

 Além disso, o acometimento do VD, ^343^ a presença de trombos intracardíacos ^674^ e a disautonomia cardíaca por lesão neuronal parassimpática ^222^ são mais frequentes na CCDC. Todos esses fatores também poderiam justificar a menor sobrevida de pacientes com CCDC em relação a pacientes com cardiomiopatias de outras etiologias para um grau semelhante de dano miocárdico. ^675^

 A disfunção do nó sinusal e os distúrbios da condução atrioventricular e intraventricular requerem maior cautela no emprego, por exemplo, de betabloqueadores, digital e amiodarona, devido ao risco de bradicardias excessivas e aparecimento ou agravamento de bloqueios preexistentes. 

 Além disso, as taquiarritmias ventriculares demandam tratamento com fármacos frequentemente associados a efeitos colaterais graves. O acometimento do VD, presente em 42% dos pacientes com disfunção do VE, ^343^ tende a provocar mais congestão sistêmica, requerendo doses maiores de diuréticos e podendo induzir hipopotassemia acentuada, que implica risco inerente de morte global, súbita e cardiovascular. ^676^ Nesse contexto, além do uso rotineiro de inibidores de aldosterona (espironolactona/eplerenona), ^606 , 677^ suplementação com potássio por via oral, visando a manter seus níveis séricos entre 4,0 e 5,0mEq/L, pode ser necessária. 

#### 11.1.2. Prevenção da Morte Súbita com Fármacos Não Antiarrítmicos 

 A morte súbita, muitas vezes inesperada e acometendo indivíduos com boa capacidade de esforço e durante a realização de exercícios, predomina, nitidamente, em subpopulações de indivíduos ambulatoriais com CCDC. ^352 , 408^ O escore de RASSI, desenvolvido nesse subgrupo populacional da CCDC, estratifica adequadamente o risco de mortalidade total, aí incluída a predominante ocorrência de morte súbita. ^408^

 Todavia, é constatação frequente que muitos desses pacientes apresentam graus variáveis de disfunção ventricular e IC clinicamente manifesta. Como corolário disso, é plausível admitir-se que o tratamento otimizado da IC em pacientes com CCDC possa redundar em potencial benefício coadjuvante para evitar a arritmia ventricular maligna e sua mais temível consequência, a morte súbita. Isso não tem ainda comprovação específica, sendo que somente alguns poucos estudos de IC incluíram amostras diminutas de pacientes com a etiologia da DC. 

 Portanto, a aplicação de alguns tratamentos farmacológicos para a CCDC complicada por IC, visando à redução de morte súbita, é extrapolada de resultados obtidos em pacientes com outras etiologias de IC, assumindo-se que existam similaridades clínicas e fisiopatológicas entre elas. 

 Doses otimizadas de IECA ou BRA, bem como de betabloqueadores (carvedilol, bisoprolol e succinato de metoprolol) e de espironolactona, devem ser visadas na IC de etiologia da DC com aquela perspectiva antiarrítmica coadjuvante. ^678^ Por exemplo, o estudo MERIT-HF, que comparou o succinato de metoprolol *versus* placebo em pacientes com IC e fração de ejeção ≤ 40%, foi interrompido prematuramente (após seguimento médio de 12 meses) devido à redução de 40% a 60% na mortalidade global e por agravamento da IC e também por morte súbita. ^679^ Resultados similares foram observados com carvedilol e bisoprolol em pacientes com ICFEr. ^680 , 681^

 Importante ressaltar que no estudo observacional REMADHE, houve menor utilização dos betabloqueadores nos pacientes com CCDC do que naqueles com outras etiologias. ^605^ Em estudo de pequenas dimensões, mas específico para CCDC, o carvedilol foi bem tolerado e associado à tendência de aumento da FEVE. ^682^ Mais recentemente, no estudo PARADIGM-HF, em pacientes com ICFEr, o sacubitril-valsartana reduziu significativamente a incidência de morte súbita em comparação ao enalapril em dose não otimizada, tanto no grupo que recebeu CDI (redução de 51%) como naqueles não submetidos ao implante de CDI (redução de 19%). ^683^ É então plausível que outras recomendações de diretrizes internacionais sejam potencialmente aplicáveis para redução de morte total e súbita na CCDC cursando com IC. ^684^

#### 11.1.3. Arritmias Ventriculares em Cardiopatias de Outras Etiologias 

 As arritmias ventriculares podem ocorrer em qualquer cardiopatia e são proteiformes na essência, podendo se manifestar como: EV monomórficas ou polimórficas, isoladas, bigeminadas, trigeminadas e pareadas; TVNS ou TVS, que também podem ser monomórficas ou polimórficas. As arritmias ventriculares podem ser assintomáticas e, em suas formas mais graves (TVS e FV), causar síncope, baixo débito e morte súbita. 

 A prevalência de episódios de TVNS ao Holter de 24 horas variou de 21% a 25% no estudo SCD-HeFT, que avaliou a mortalidade em pacientes com IC de etiologias isquêmica e não isquêmica. ^685^ O estudo EMIAT, que analisou pacientes pós-infarto do miocárdio com FEVE < 40%, reportou prevalência de arritmia ventricular (definida como 10 ou mais EV/h ou TVNS no Holter) em 39% a 41% dos indivíduos. ^686^ Já o estudo GESICA, em pacientes com IC grave de diversas etiologias, reportou alta ocorrência de EV > 10/h (71%), EV pareadas (56%) e TVNS (33%) ao Holter. ^687^

#### 11.1.4. Amiodarona em Pacientes com Cardiopatias de Outras Etiologias: Prevenção Primária 

 A amiodarona possui os quatro efeitos antiarrítmicos da classificação de Vaughan-Williams: bloqueio de canais de sódio (classe I); inibição não competitiva alfa- e beta-adrenérgica (classe II); interferência com os canais de potássio, levando a prolongamento do potencial de ação, da repolarização e da refratariedade (classe III); e bloqueio dos canais de cálcio (classe IV). 

 O uso de amiodarona ( *versus* placebo, outro fármaco antiarrítmico ou grupo controle) na prevenção primária de mortalidade total e súbita foi avaliado por meio de várias meta-análises. Em 1997, o estudo ATMA, ^688^ utilizando dados individuais de pacientes de oito ECR após infarto agudo do miocárdio (EMIAT, CAMIAT, GEMICA, PAT, SSSD, BASIS, Hockings *et al* . e CAMIAT-P) e de cinco estudos incluindo pacientes com IC congestiva (CHF-STAT, GESICA, EPAMSA, Nicklas *et al* . e Hamer *et al* .), mostrou redução de 13% no risco de óbito total (p = 0,03) e de 29% no risco de morte súbita de causa arritmogênica (p = 0,0003) com a amiodarona. Não houve excesso de mortes não arrítmicas com a amiodarona e ambos os grupos de pacientes (após infarto agudo do miocárdio e IC congestiva) se beneficiaram do tratamento antiarrítmico. 

 No mesmo ano, outra meta-análise, ^689^ utilizando metodologia hierárquica bayesiana e dados publicados dos mesmos 13 estudos incluídos no ATMA e de 2 estudos adicionais (CASCADE e ASSG) envolvendo pacientes recuperados de parada cardíaca ou com TVS, concluiu, de forma semelhante, que a amiodarona reduz a mortalidade por todas as causas em cerca de 19% (p < 0,01), com reduções um pouco maiores na mortalidade cardíaca (23%, p < 0,001) e na morte súbita (30%, p < 0,001). Houve uma tendência de redução de risco de óbito também maior nos estudos que exigiram evidência de ectopia ventricular frequente ou complexa como critério de inclusão (25%) em comparação aos demais estudos (10%). 

 Com os resultados animadores com a amiodarona e o advento do CDI, o passo seguinte mais provável seria a comparação focalizada na alocação aleatória a amostras de pacientes tratados com amiodarona ou CDI ou placebo na prevenção primária de morte total. Esse foi o objetivo principal do estudo SCD-HeFT, publicado em 2005, que incluiu 2.521 pacientes com fração de ejeção ≤ 35%, em classe funcional II ou III da NYHA, sendo a IC de origem isquêmica em 52% dos pacientes e não isquêmica nos demais. ^685^ Após seguimento mediano de 45,5 meses, a mortalidade total foi de 29% no grupo placebo, 28% no grupo amiodarona e 22% no grupo CDI, ou seja, enquanto a amiodarona apresentou efeito nulo na mortalidade total, em comparação ao placebo, a terapia com CDI causou uma redução relativa de risco de 23% (p = 0,007). 

 Vale ressaltar que, com base em análise pré-especificada de subgrupos, os resultados não variaram de acordo com a etiologia da IC, mas variaram de acordo com a classe funcional da NYHA. Assim, em pacientes na classe III, observou-se aumento de mortalidade com a amiodarona (comparada ao placebo) e nenhuma diferença entre os tratamentos CDI e placebo. Apesar de a interação entre CDI e classe funcional ter sido extremamente significativa (p < 0,001), os autores ignoraram esses resultados e concluíram que em ambas as classes (II e III), a terapia com CDI unicameral ventricular foi capaz de reduzir a mortalidade total. 

 Seguindo esse mesmo paradigma, todas as diretrizes passaram a recomendar o implante de CDI, profilaticamente, a pacientes com fração de ejeção ≤ 35%, em classe funcional II e também III da NYHA. Apesar dos resultados incontestes proporcionados pelo estudo SCD-HeFT, duas ressalvas devem ser feitas. Primeiro, o critério de inclusão foi disfunção ventricular e não a documentação de arritmia ventricular complexa e frequente ao Holter. Segundo, dentre as várias análises de subgrupos realizadas, a principal delas, a nosso ver, comparando amiodarona com placebo em pacientes que apresentavam TVNS documentada (22% da população do estudo), por motivos desconhecidos, não foi contemplada. 

 É importante destacar que, no estudo CHF-STAT, ^690^ randomizando 674 pacientes com IC (fração de ejeção ≤ 40%) de etiologia isquêmica e não isquêmica e pelo menos 10 EV/hora ao Holter de 24 horas para receber amiodarona ou placebo, após seguimento mediano de 45 meses, a amiodarona reduziu significativamente a frequência da arritmia ventricular e melhorou a função ventricular, mas não foi capaz de aumentar a sobrevida. 

 Entretanto, em análise de subgrupo pré-especificada e com base em randomização estratificada pela etiologia da IC, houve tendência de menor mortalidade com a amiodarona nos pacientes não isquêmicos (p = 0,07). À época da publicação do estudo SCD-HeFT, também já eram conhecidos os resultados de dois pequenos ECR, EPAMSA (127 pacientes) ^691^ e AMIOVIRT (103 pacientes), ^692^ ambos abertos. O primeiro comparou amiodarona com grupo controle em cardiopatas isquêmicos e não isquêmicos com fração de ejeção < 35% e arritmia ventricular graus 2 ou 4 de Lown ao Holter, e o segundo comparou amiodarona com CDI em IC exclusivamente não isquêmica, com fração de ejeção ≤ 35% e TVNS ao Holter. No estudo piloto argentino EPAMSA, que incluiu 24 pacientes com CCDC, após 1 ano de seguimento, as reduções de morte total e súbita com amiodarona foram de 71% (p = 0,02) e 71% (p = 0,04), respectivamente. ^691^

 Já no estudo AMIOVIRT, interrompido precocemente pelo critério de futilidade, as sobrevidas após 1 ano (90% *versus* 96%) e 3 anos (88% *versus* 87%) não foram estatisticamente diferentes entre os grupos amiodarona e CDI (p = 0,8). ^692^

 Resultados positivos com a amiodarona também foram observados em outro estudo argentino (GESICA), ^687^ que incluiu 516 pacientes com IC grave de etiologia isquêmica e não isquêmica (48 pacientes com CCDC), em classe funcional predominantemente III ou IV da NYHA, apresentando pelo menos dois de três índices de disfunção ventricular sistólica: ICT > 0,55, fração de ejeção ≤ 35% e DDVE ≥ 32cm/m ^2^ . Os pacientes foram randomizados para grupo amiodarona ou controle e, após um tempo médio de acompanhamento de 13 meses, a mortalidade total foi de 41,4% no grupo controle e de 33,5% no grupo amiodarona, uma redução relativa de risco de 28% (p = 0,024). Os pacientes foram randomizados de acordo com a presença de TVNS ao Holter de admissão, que foi observada em 33,5% da população global do estudo. A redução do risco de óbito com a amiodarona ocorreu independentemente da presença de arritmia ventricular, mas foi numericamente maior nos pacientes com TVNS documentada (34% *versus* 24,5%). 

 Mais recentemente, foram publicados os resultados de longo prazo do estudo SCD-HeFT. ^693^ Após acompanhamento de 11 anos, o benefício do CDI em comparação ao placebo permaneceu estatisticamente significativo, mas houve atenuação de efeito, com redução relativa do risco de óbito diminuindo de 23% (após 45,5 meses) para 13% (após 11 anos, p = 0,028) e interação significativa entre tempo de seguimento (antes e após 6 anos) e benefício do CDI (p < 0,0015). Curiosamente, a análise de subgrupo, de acordo com a etiologia da IC, também mostrou resultados heterogêneos a longo prazo. Enquanto o efeito benéfico do CDI se manteve nos pacientes com IC de etiologia isquêmica (RRR de 19%, p = 0,009), naqueles com IC de etiologia não isquêmica, a redução de mortalidade com o CDI não mais foi observada (RRR de 3%, p=0,802). Como, após a publicação do ensaio original, mais da metade dos pacientes alocados para placebo ou amiodarona receberam implante de CDI ou dispositivo ressincronizador e a análise estatística preconizada foi a “intenção de tratar”, esse *crossover* pode ter interferido nos resultados. Todavia, esses resultados não sofreram alterações quando se utilizou a metodologia de análise “ *as treated* ”, que compara os grupos de acordo com o tratamento recebido e não conforme a alocação inicial. 

 Após a publicação do estudo SCD-HeFT, mais algumas meta-análises foram realizadas, adicionando os resultados desse e de alguns outros trabalhos. A primeira delas ^694^ identificou 15 estudos (apenas 1 de prevenção secundária, o OPTIC), totalizando 8.522 pacientes, que foram randomizados para amiodarona ou placebo/controle. A amiodarona reduziu o risco de morte súbita em 29% (p < 0,001) e de morte cardiovascular em 18% (p = 0,004). A redução de risco de mortalidade por todas as causas (13%) não atingiu significância estatística (p = 0,093). Análise de subgrupo pré-especificada mostrou redução do risco de morte total de 19% (IC 95%, 2% a 32%), com doses de amiodarona > 200mg/dia. Já doses ≤ 200mg/dia não foram eficazes (redução de 1%; IC 95%: -31% a 25%). Por outro lado, o uso de amiodarona esteve associado a um aumento de duas e cinco vezes, respectivamente, no risco de toxicidade pulmonar e tireoidiana. Os autores concluíram que a amiodarona representa uma alternativa viável para prevenir a morte súbita cardíaca em pacientes não elegíveis ou que não têm acesso à terapia com CDI. ^694^

 Outra revisão sistemática com meta-análise, seguindo as recomendações da colaboração do sistema Cochrane, foi publicada em 2015 ^695^ e incluiu 24 ECR totalizando 9.997 pacientes, com o objetivo de comparar amiodarona *versus* placebo/controle ou outros fármacos antiarrítmicos na prevenção primária (pacientes de alto risco para morte súbita) e secundária (pacientes recuperados de parada cardíaca ou com TVS sincopal). 

 Nos estudos de prevenção primária (total de 18), a amiodarona reduziu significativamente a mortalidade súbita, cardiovascular e global, mas a qualidade da evidência foi considerada baixa (comparação com placebo) ou moderada (comparação com outros fármacos antiarrítmicos). Na prevenção secundária (total de 6 estudos), não se observou redução da mortalidade súbita e global, sendo a evidência considerada de muito baixa ou baixa qualidade. ^695^

 Com base nesses resultados: 1) é razoável concluir que, comparada com placebo, grupo controle ou outro fármaco antiarrítmico, no que concerne à prevenção primária, a amiodarona reduz modestamente a morte por todas as causas, com efeito mais expressivo na redução de morte súbita, tanto na IC de etiologia isquêmica quanto (e principalmente) na IC de etiologia não isquêmica; 2) é plausível especular que o efeito benéfico da amiodarona seja maior quando se consegue documentar a presença de TVNS e de elevada densidade da arritmia ventricular ao Holter, fato que parece ser de maior relevância em presença de disfunção ventricular. Corroborando essa suposição, meta-análise ^696^ incluindo 11 estudos de pacientes com IC (isquêmica e não isquêmica) ou CMD não isquêmica associada a disfunção ventricular esquerda mostrou que a presença de TVNS ao Holter é um preditor independente de morte súbita cardíaca (OR 3,03; IC 95%: 2,44–3,77); 3) o único ECR que comparou diretamente amiodarona com CDI em prevenção primária (AMIOVIRT) ^692^ não mostrou superioridade do CDI, mas é limitado pelo seu pequeno tamanho amostral. 

 O estudo SCD-HeFT, ^685 , 693^ apesar de não comparar amiodarona com CDI, mas cada um dos dois contra placebo, não exigiu a presença de arritmia ventricular como critério de inclusão, não procedeu a análise de subgrupos baseada na presença de TVNS, mostrou benefício do CDI apenas em pacientes em classe funcional II da NYHA e teve esse benefício mantido a longo prazo apenas nos pacientes com IC de etiologia isquêmica, aspectos que devem ser considerados ao se tentar extrapolar seus resultados para a CCDC. 

#### 11.1.5. Amiodarona em Pacientes com Cardiopatias de Outras Etiologias: Prevenção Secundária 

 A prevenção secundária da morte súbita diz respeito a pacientes que foram recuperados de uma parada cardíaca por FV ou TV sem pulso, ou que já apresentaram pelo menos um episódio documentado de TVS. Fazem parte desse grupo os pacientes com síncope de etiologia provavelmente cardíaca, que, uma vez levados ao EEF, apresentam indução de FV ou de TVS hemodinamicamente instável (ou mesmo estável segundo alguns autores). 

 As taquiarritmias ventriculares sustentadas têm sido tipicamente agrupadas em uma única categoria e coletivamente denominadas “ameaçadoras à vida” ou “malignas”. Embora a FV previsivelmente precipite a parada cardíaca, a não ser que seja de curta duração e reverta espontaneamente (evento muito raro e mal documentado), a TVS, por sua vez, cursa com ampla gama de manifestações hemodinâmicas e clínicas. 

 Assim, deve-se evitar o agrupamento indiscriminado dessas várias entidades arrítmicas, pois diferenças relacionadas aos seus prognósticos e tratamentos devem existir. Certamente o grau de disfunção ventricular (expresso pela FEVE) e ainda os sintomas associados à arritmia e o tipo de cardiopatia estrutural são elementos que devem ser considerados durante avaliação desses pacientes. 

 O ponto de corte dicotomizante para a FEVE tem sido geralmente de 35% ou 40% e uma das gradações de sintomas propõe quatro classes: I - sem sintomas ou apenas palpitações; II - lipotimia, dor no peito ou dispneia; III - síncope, estado mental alterado ou outra evidência de comprometimento hemodinâmico importante (sinais e sintomas de baixo débito, edema pulmonar agudo, etc.); e IV - parada cardíaca (pulso e respiração ausentes). ^697^

 É bastante provável que o prognóstico e o tratamento de um paciente com cardiopatia isquêmica, recuperado de parada cardíaca por FV e com FEVE de 30%, sejam diferentes daqueles de um paciente com CMD (p. ex. a própria CCDC), TVS hemodinamicamente estável, FEVE relativamente preservada e sintoma de palpitações. 

 Três ECR (AVID, CIDS e CASH) ^698 - 700^ compararam amiodarona (ou outro fármaco antiarrítmico) com CDI na prevenção secundária de morte total. 

 O primeiro e maior deles (AVID) ^698^ randomizou 1.016 pacientes (81% com cardiopatia isquêmica), recuperados de parada cardíaca por FV (45% dos pacientes), com TVS sincopal (21%) ou ainda com TVS, FEVE ≤ 40% e sintomas sugestivos de comprometimento hemodinâmico grave associados (34%), para terapia com CDI ou fármacos antiarrítmicos (amiodarona, em 96% dos casos). A média de idade foi de 65 anos, a FEVE média foi de 32% no grupo CDI e de 31% no grupo antiarrítmico e 79% dos pacientes eram do sexo masculino. Após seguimento médio de 18,2 meses, o estudo foi encerrado precocemente devido à superioridade do CDI em reduzir morte total (15,8% *versus* 24%), com reduções relativas de risco de 39%, 27% e 31%, após 1, 2 e 3 anos de acompanhamento, respectivamente (p < 0,02, ajustado para múltiplas análises). 

 O estudo canadense CIDS ^699^ randomizou 659 pacientes (83% com cardiopatia isquêmica) para CDI ou amiodarona, sendo 48% deles recuperados de parada cardíaca por FV, 13% com TVS sincopal, 25% com TVS (FC ≥ 150 bpm), FEVE ≤ 35%, causando pré-síncope ou angina, e 14% deles com síncope e TVS induzida pela estimulação elétrica programada. A média de idade foi de 64 anos, a FEVE média foi de 34% no grupo CDI e de 33% no grupo amiodarona, e 85% dos pacientes eram do sexo masculino. Após tempo médio de acompanhamento de 3 anos, houve redução não significativa do risco anual de morte total (10,2% *versus* 8,3%, p = 0,142) e de morte arrítmica (4,5% *versus* 3,0%, p = 0,094) com o CDI. 

 O menor dos três estudo, CASH, ^700^ foi realizado na cidade de Hamburgo, na Alemanha, e comparou o CDI com diferentes antiarrítmicos (amiodarona, metoprolol e propafenona). Ao contrário dos ensaios anteriores, incluiu apenas pacientes recuperados de parada cardíaca (por FV em 84% e por TV em 16%). O braço da propafenona (58 pacientes) foi descontinuado após seguimento médio de 11,3 meses por apresentar taxa de mortalidade 61% maior que a observada no grupo do CDI. Os demais pacientes, num total de 288 distribuídos igualmente entre os grupos CDI, amiodarona e metoprolol, permaneceram no estudo. A média de idade foi de 58 anos, a FEVE média foi de 46%, 80% dos pacientes eram do sexo masculino e 73% tinham cardiopatia isquêmica. Ao longo de seguimento médio de 57 meses, a mortalidade total foi menor no braço CDI em comparação com o braço amiodarona/metoprolol (36,4% *versus* 44,4%), embora a diferença não tenha atingido significância estatística (p = 0,08 unicaudal). 

 O término prematuro do estudo AVID, podendo superestimar o benefício do CDI, assim como o número menor de pacientes arrolados no CIDS e no CASH, podendo diminuir o poder dos testes estatísticos em detectar um real benefício do tratamento com CDI, motivaram a realização de uma meta-análise, comparando o CDI exclusivamente com a amiodarona. ^701^ Essa meta-análise, explorando dados individuais dos pacientes dos três estudos, foi colocada em um banco de dados com protocolo pré-especificado e teve seus resultados publicados em 2000. Foram incluídos 1.866 pacientes (CDI = 934; amiodarona = 932), com média de idade de 63,5 anos, FEVE média de 33,5%, a grande maioria do sexo masculino (81,5%) e com diagnóstico de cardiopatia isquêmica (82%). As estimativas de benefício do CDI observadas nos três estudos foram consistentes entre si (p, heterogeneidade = 0,306) e, em conjunto, resultaram em redução relativa de risco de óbito total de 28% (HR = 0,72; IC 95%: 0,60-0,87; p = 0,0006) e de morte arrítmica de 50% (HR = 0,50; IC 95%: 0,37-0,67; p < 0,0001) com o CDI, traduzindo-se em ganho médio de sobrevida de 4,4 meses, após seguimento médio de 6 anos. 

 Entretanto, na análise de subgrupos, o benefício relacionado ao aumento de sobrevida com CDI foi observado apenas em pacientes com FEVE ≤ 35% (HR = 0,66) e não naqueles com FEVE > 35% (HR = 1,2; p, interação = 0,01). Embora o uso de betabloqueadores tenha sido maior no grupo CDI (42% *versus* 19%) e o benefício da terapia com CDI também fosse maior naqueles em uso de betabloqueador (HR = 0,58 *versus* HR = 0,88), essa diferença não foi estatisticamente significativa (p, interação = 0,095). ^701^

 Com base nos resultados desses ECR, ^698 - 700^ da meta-análise dos mesmos ^701^ e de alguns estudos observacionais, ^702^ as principais diretrizes internacionais ^684 , 703^ passaram a recomendar fortemente a terapia com CDI a todo paciente recuperado de parada cardíaca por FV ou TV e a todo paciente com cardiopatia estrutural e TVS, independentemente da FEVE e da presença e do tipo de sintomas relacionados à arritmia, excetuando-se, obviamente, os casos de arritmias por prováveis causas secundárias ou reversíveis (p. ex. distúrbios eletrolíticos, isquemia miocárdica e pró-arritmia) ou com expectativa de vida menor que 1 ano. 

 Vale ressaltar que nenhum dos três ECR incluiu pacientes com TVS hemodinamicamente estável ou bem tolerada e que a análise de subgrupos da meta-análise referida anteriormente não mostrou benefício do CDI em relação à amiodarona em pacientes com FEVE > 35%. Ainda, de acordo com essas diretrizes (potencialmente enviesadas quando generalizam, apesar das evidências de problemas com as análises dos resultados), apenas nos casos de indisponibilidade ou de contraindicação para a terapia com CDI ou quando essa for recusada pelo paciente, a amiodarona poderia ser utilizada com o objetivo de se tentar reduzir morte súbita (classe IIb). ^684 , 703^

#### 11.1.6. Arritmias Ventriculares em Pacientes com Cardiomiopatia Crônica da Doença de Chagas: Características e Tratamento 

 Conforme já assinalado acima, embora mais comumente encontradas em fases mais avançadas da CCDC, as arritmias ventriculares podem ocorrer já em estágios iniciais da doença e mesmo na ausência de comprometimento significativo da função ventricular sistólica global. ^672^

***11.1.6.1. Extrassístoles Ventriculares***

 Estão presentes em 86% a 88% dos pacientes com CCDC sem IC (classes funcionais I e II) e em praticamente todos os pacientes com IC (classes funcionais III e IV) ao Holter de 24 horas. ^672 , 704^ A densidade de EV também é elevada, de tal forma que 45% e 89% dos pacientes sem e com IC, respectivamente, apresentam mais do que 1000 EV/h ao Holter. ^672^

 Quando ocorrem em pacientes assintomáticos e com função ventricular preservada, as EV não requerem tratamento. No entanto, em pacientes assintomáticos e com alta densidade arrítmica (> 16-20% de EV ao Holter de 24h), há possibilidade de desenvolvimento de taquicardiomiopatia, ^705^ isso é, disfunção ventricular sistólica causada ou agravada pela arritmia e que pode ser atenuada ou revertida com a supressão das EV e, assim, cursar com aumento de sobrevida. ^706^

 Quando as EV são muito sintomáticas, mesmo na ausência de disfunção ventricular ou de realce tardio (fibrose) à RMC, o uso de medicamentos antiarrítmicos se impõe, mas esse deve ser individualizado, evitando-se, a princípio, a amiodarona, em virtude de seus efeitos adversos. Nessa situação, sugere-se o uso de um betabloqueador (e.g. nadolol ou sotalol) ou propafenona. Por outro lado, se houver disfunção ventricular ou substrato arritmogênico de fibrose detectável à RMC, antiarrítmicos da classe I não devem ser utilizados, devido aos seus efeitos pró-arrítmicos e eventual efeito inotrópico negativo, podendo ainda aumentar a mortalidade, conforme descrito em outras cardiopatias. ^707^ Então, a amiodarona pode ser indicada, em doses de 200 a 600 mg/dia, pela sua elevada eficácia em diminuir significativamente a densidade de EV. ^708^

***11.1.6.2. Taquicardia Ventricular Não Sustentada***

 A TVNS acomete 42% dos pacientes com CCDC sem IC e 89% daqueles com IC, ^672^ uma prevalência muito maior se comparada à de outras cardiopatias. Pode ser observada mesmo em pacientes com função ventricular normal e sua detecção no Holter ou durante teste ergométrico constitui marcador independente de mau prognóstico. ^408 , 436 , 458^ Quando associada à disfunção ventricular esquerda (global ou segmentar), achado relativamente comum na CCDC, aumenta o risco de óbito em 15 vezes se comparada a pacientes sem TVNS e com função ventricular normal. ^458^ Na ausência de dados disponíveis para avaliação de desfechos relevantes, como mortalidade e internação hospitalar, uma meta-análise de estudos antigos com a amiodarona na CCDC mostrou importante redução da densidade de arritmia ventricular em registros seriados de Holter (93% das EV isoladas, 79% dos pares e 100% dos episódios de TV). ^708^

 Conforme mencionado anteriormente, os ECR argentinos GESICA ^687^ e EPAMSA ^691^ incluíram pacientes com CCDC e mostraram redução de mortalidade com a amiodarona. Entretanto, o pequeno número de indivíduos com CCDC elencados nos dois estudos (total de apenas 72) impede uma conclusão definitiva. Como então conduzir os pacientes com TVNS? Na ausência de disfunção ventricular, o tratamento farmacológico deve seguir, em linhas gerais, as mesmas orientações preconizadas para o tratamento das EV. Em presença de disfunção ventricular, três opções estão disponíveis: betabloqueador, amiodarona e CDI, esse último a ser discutido mais adiante. 

 O objetivo do tratamento deve ser o alívio de sintomas (caso estejam presentes), a melhora da função ventricular e a prevenção da morte súbita. Como não existem dados convincentes, por meio de ECR, para apoiar qualquer uma das três opções, as recomendações para o tratamento desses pacientes baseiam-se na extrapolação de resultados de estudos realizados em outras doenças cardíacas e em dados observacionais (que são limitados) relacionados à CCDC. 

 Após análise detalhada da literatura, optou-se pela indicação preferencial de um betabloqueador seletivo (succinato de metoprolol, carvedilol ou bisoprolol) associado ou não à amiodarona, decisão que deverá ser individualizada e compartilhada com o paciente. 

***11.1.6.3. Taquicardia Ventricular Sustentada e Fibrilação Ventricular***

 Apesar de a prevalência das arritmias ventriculares sustentadas não ser amplamente conhecida, pacientes com CCDC, independentemente da função ventricular, podem apresentar TVS monomórfica, TVS polimórfica ( *torsades de pointes* ) e FV, que devem ser prontamente revertidas nas salas de emergência. A amiodarona constitui a melhor opção medicamentosa nos casos de TVS estável ou de FV refratária ou recorrente. 

 Quando houver instabilidade hemodinâmica, a cardioversão elétrica imediata é recomendada, segundo protocolo do Suporte Avançado de Vida Cardiovascular (ACLS). Em caso de recidivas imediatas (“tempestades elétricas”), deve-se considerar a administração de antiarrítmicos (preferencialmente amiodarona), com suporte de oxigênio adequado, monitorização cardíaca e correção de possíveis distúrbios eletrolíticos. 

 Segundo o protocolo do ACLS, dois fármacos são indicados para o tratamento de arritmias ventriculares sustentadas na sala de emergência: a amiodarona e a procainamida, ambas administradas por via endovenosa. As doses preconizadas estão apresentadas no Quadro 11.1 . 

**Quadro 11.1:** – Tratamento de arritmias ventriculares sustentadas na sala de emergência.

**AMIODARONA** (1 ampola = 150mg)	**TVS estável:** 1 ampola EV durante 10 min, podendo ser repetida em caso de não reversão. A administração de 1 mg/min EV em 6 horas seguido por 0,5 mg/min EV em 18 horas subsequentes pode ser realizada para estabilização da arritmia ventricular.
	**Parada cardiorespiratória por FV/TV sem pulso refratária:** 2 ampolas (300mg) EV em bolus após o terceiro choque, e repetir mais 1 ampola (150mg) EV em caso de insucesso aós o quinto choque
**PROCAINAMIDA** (1 AMPOLA = 500mg)	**TVS estável:** Dose de ataque: 10 a 17mg/kg (20-50mg/min) EV Dose de manutenção: 1–4 mg/min EV

 É importante lembrar que a administração endovenosa de amiodarona pode causar flebite e hipotensão arterial, reduzir a frequência sinusal, aumentar a duração do complexo QRS e do intervalo QT, aumentar a refratariedade do nó atrioventricular, reduzir a FC (alentecimento) da TVS e melhorar o limiar de desfibrilação do CDI. 

 A procainamida pode aumentar o intervalo PR, a duração do complexo QRS e o limiar de desfibrilação do CDI. Também pode causar hipotensão arterial e redução da FEVE, provocar diarreia e náuseas e desencadear sintomas e sinais da síndrome lúpica eritematosa. 

 Outros fármacos antiarrítmicos de administração endovenosa como lidocaína, verapamil, betabloqueadores (metoprolol, esmolol ou propranolol) e sotalol apresentam baixa eficácia para reverter taquiarritmias ventriculares sustentadas e devem ser evitados na CCDC ou usados apenas como opções secundárias em escassos contextos. 

 Uma vez controlada a taquiarritmia ventricular sustentada ou revertido o quadro de parada cardíaca, o tratamento subsequente tem como objetivos principais prevenir as recorrências e, principalmente, evitar a morte súbita. Apesar da elevada prevalência da CCDC na América Latina e da alta taxa de letalidade decorrente dessas arritmias, é lastimável que inexistam ECR devidamente controlados, com contingente amostral adequado para finalmente esclarecer qual deve ser a melhor conduta a ser adotada caso a caso. Dentre as principais opções estão o uso de amiodarona, o implante de CDI, a ablação por cateter ou uma associação dessas terapias, com a escolha tendo então que se basear nos resultados de estudos observacionais ou de registros realizados na CCDC, que são bastante heterogêneos e conflitantes, ou ainda na extrapolação de dados oriundos de ECR ou de diretrizes aplicáveis em outras cardiopatias e que apresentam algumas inconsistências e podem não se aplicar à CCDC devido a várias de suas peculiaridades. 

 Assim, a estratégia *“One-size fits all: what’s good for the gander is good for the goose”* , ou seja, CDI para todos os pacientes como terapêutica ideal para prevenir morte súbita, parece não ser a mais apropriada na CCDC. Obviamente que, quando se fala em prevenção de morte súbita, na verdade estamos nos referindo à morte por todas as causas, pois, muitas vezes, não se consegue distinguir o mecanismo exato do óbito (pode haver erro na adjudicação) e, além disso, de nada adianta um tratamento prevenir a morte súbita sem reduzir a mortalidade total, pois estaria apenas modificando o modo de óbito. 

 Vale destacar que, em comparação à cardiopatia isquêmica e não isquêmica de outras etiologias, pacientes com CCDC tratados com CDI para prevenção secundária tendem a apresentar FEVE mais alta, maior densidade e complexidade de arritmia ventricular espontânea, maior número de terapias apropriadas (choque e terapia antitaquicardia) e também das inapropriadas pelo CDI, tempo mais curto para o primeiro choque apropriado após o implante, tempestades elétricas mais frequentes e menor sobrevida livre de terapias do CDI, fatores que podem influenciar na escolha do tratamento. A FEVE, independentemente do tipo de cardiopatia, é fator primordial na determinação do prognóstico e seleção da terapia mais adequada. 

 Devido à sua elevada eficácia antiarrítmica, baixa incidência de pró-arritmia e de efeitos colaterais intoleráveis, principalmente quando utilizada em doses mais baixas, e bom perfil de segurança, mesmo quando administrada a pacientes com disfunção ventricular, a amiodarona (introduzida em nosso meio há mais de 4 décadas) é considerada o fármaco de primeira escolha no tratamento de pacientes com CCDC e arritmias ventriculares de alto risco. 

 Rassi Jr *et al* . ^352^ foram os primeiros a estudar o impacto do tratamento medicamentoso antiarrítmico na evolução de pacientes com CCDC. Ao analisarem a curva atuarial de sobrevida de 34 pacientes com TVS monomórfica tratados empiricamente com amiodarona, de maneira isolada ou em associação com outros antiarrítmicos, e compararem-na com a curva de outra coorte de 42 pacientes não tratados ou que fizeram uso de procainamida ou quinidina, únicos medicamentos disponíveis à época, constataram sobrevida significativamente maior no grupo tratado com amiodarona. Após 1, 4 e 8 anos de acompanhamento, a sobrevida foi de 87%, 65% e 59%, respectivamente, para o grupo tratado com amiodarona e de 57%, 22% e 7%, respectivamente, para o grupo não tratado ou tratado com aqueles antiarrítmicos da classe I (p < 0,01). 

 Scanavacca *et al* . ^709^ também relataram resultados a longo prazo sobre o uso empírico de amiodarona no Brasil em coorte de 35 pacientes com CCDC e taquiarritmia ventricular sustentada (recuperados de parada cardíaca = 8,5%; TVS com síncope ou pré-síncope = 77%; TVS com palpitações taquicárdicas bem toleradas = 14,5%). A média de idade foi de 50 anos, a FEVE média foi de 41%, 68,5% dos pacientes eram do sexo masculino e 86% estavam em classe funcional I/II. Após 27 meses de acompanhamento, a probabilidade de recorrência de TVS foi de 38%, 44% e 56% no seguimento de 1, 2 e 3 anos, respectivamente. A taxa de mortalidade cardíaca foi de 4%, 11% e 18% e a de morte súbita foi de 0%, 4% e 11% no seguimento de 1, 2 e 3 anos, respectivamente. De relevância, todos os pacientes em classe funcional III ou IV e com FEVE < 30% tiveram recorrência de TVS, sendo a mortalidade cardíaca nesse grupo de 80%. Por outro lado, apenas 30% dos pacientes em classe funcional I/II da NYHA e com FEVE > 30% apresentaram TVS recorrente (p < 0,05) e nenhum foi a óbito. A dose média de amiodarona, ao término do estudo, foi de 356 mg/dia e 15 (43%) pacientes relataram efeitos colaterais. 

 Leite *et al* . ^481^ avaliaram o papel da estimulação ventricular programada em predizer a eficácia a longo prazo de fármacos antiarrítmicos da classe III. Foram estudados 115 pacientes com CCDC e taquiarritmia ventricular sustentada (TVS recorrente com síncope ou pré-síncope = 54%; TVNS com síncope ou pré-síncope e indução de TVS ao EEF = 32%; e TVS hemodinamicamente estável com sintomas toleráveis = 14%). A média de idade foi de 52 anos, a FEVE média foi de 49%, 60% dos pacientes eram do sexo masculino e 83% estavam em classe funcional I/II. Com base nos resultados do EEF, após impregnação com amiodarona (78 pacientes) ou sotalol (37 pacientes), os pacientes foram divididos em três grupos: grupo 1 – sem indução de TVS (20%); grupo 2 – indução de TVS hemodinamicamente estável (39%); e grupo 3 – indução de TVS hemodinamicamente instável (41%). 

 Após seguimento médio de 52 meses, a mortalidade global foi de 39,1% (9%/ano), sendo significativamente maior no grupo 3 do que nos grupos 2 e 1 (69%, 22,2% e 26%, respectivamente, p < 0,0001). Já a recorrência de TVS foi significativamente menor no grupo 1 do que nos grupos 2 e 3 (39,1%, 62,2% e 74,5%, respectivamente, p = 0,005). Portanto, em pacientes com TVS e FEVE relativamente preservada tratados com antiarrítmicos de classe III, o EEF parece identificar aqueles com menor risco de óbito que poderiam permanecer em tratamento medicamentoso. Já nos de pior prognóstico, o CDI poderia ser a opção mais adequada à luz dessas observações. ^481^

 Sarabanda *et al* . ^710^ estudaram os preditores de mortalidade em 56 pacientes com CCDC e TV (TVS em 28 e TVNS em 28) e identificaram apenas a FEVE como marcador independente de mau prognóstico, de tal forma que FEVE < 40% aumenta o risco de óbito em 12 vezes (p = 0,0001). Os pontos de corte de maior acurácia para morte súbita e morte total foram FEVE de 40% e 38%, respectivamente. Quanto à evolução clínica da coorte de 28 pacientes com TVS, todos tratados empiricamente com amiodarona (quando o contexto era de indisponibilidade de implante de um CDI), esse grupo tinha média de idade de 54 anos, FEVE média de 42%, 64% dos pacientes eram do sexo masculino, 100% estavam em classe funcional I/II e 43% tinham história de síncope, tendo sido relatada taxa de sobrevida de 85% e 67% após 1 e 3 anos de acompanhamento, respectivamente. 

#### 11.1.7. Cuidados Durante Utilização de Amiodarona

 Efeitos adversos com a amiodarona incluem microdepósitos corneanos, bradicardia sinusal, BAV, aumento do intervalo QT, efeitos dermatológicos (fotossensibilidade e coloração cinzento-azulada da pele), disfunção tireoidiana (hipotireoidismo, mais frequente, e hipertireoidismo, mais raro), toxicidade pulmonar e, menos comumente, hepatotoxicidade. Efeitos colaterais neurológicos tardios como tremores, parestesias e ataxia também podem ocorrer. ^711^

 A toxicidade pulmonar é a complicação mais séria e potencialmente fatal do uso de amiodarona. O comprometimento pulmonar secundário à amiodarona se manifesta como pneumonia intersticial (mais frequente) ou pneumonia eosinofílica, pneumonite organizada, insuficiência respiratória aguda ou hemorragia alveolar difusa. Os sintomas iniciais são dispneia e tosse não produtiva, com ou sem febre. A radiografia de tórax mostra opacidades difusas ou localizadas, reticulares ou consolidadas. A tomografia de tórax revela comprometimento intersticial e opacidades difusas bilaterais. 

 Os primeiros relatos de toxicidade pulmonar referiam prevalência de 5% a 15% quando doses de manutenção ≥ 400mg/dia eram usualmente administradas. Atualmente, com as doses reduzidas para 200mg/dia, a incidência varia de 1% a 5%. Os fatores de risco mais importantes para a toxicidade pulmonar são, além de altas doses diárias de amiodarona (≥ 400mg), maiores doses cumulativas (longos períodos de administração), doença pulmonar pré-existente, cirurgia torácica e angiografia pulmonar. 

 Em meta-análise que reuniu quatro estudos com total de 1.465 pacientes, não houve diferença significativa na ocorrência de toxicidade pulmonar em pacientes que receberam amiodarona em dose baixa (definida como 150 a 330 mg/dia) em comparação com placebo. ^712^

 Em outra meta-análise, que reuniu 43 estudos e 11.395 pacientes, o risco relativo para eventos adversos pulmonares secundários ao uso de amiodarona foi de 1,77, com doses ≥ 300mg/dia e com seguimento clínico > 12 meses. Doses inferiores a 300 mg/dia não se associaram a incidência aumentada de complicações pulmonares em comparação com placebo. ^713^

 Em outro estudo, no entanto, mesmo doses inferiores a 200mg/dia foram associadas a aumento de alterações pulmonares. Assim, pacientes em uso de amiodarona devem receber a menor dose que seja eficaz, além de se submeter a monitorização clínica e laboratorial de forma periódica e sistemática. ^714^

 As doses iniciais da amiodarona, por via oral, em pacientes ambulatoriais, devem ser de 400 a 600 mg/dia, até se completar a dose de ataque cumulativa de 6 a 10 gramas. Em pacientes internados, as doses de ataque podem ser de 400 até 1200 mg/dia. Em seguida, a manutenção deve ser individualizada e a menor dose eficaz determinada. 

 A Tabela 11.1 resume as recomendações de monitorização clínica e laboratorial em pacientes recebendo amiodarona. 

**Tabela 11.1:** – Recomendações de monitorização clínica e laboratorial em pacientes usando amiodarona.

Sistema	Exame	Seguimento	Efeitos adversos	Recomendação
Cardiovascular	ECG	Semestral	BAV, TdP	Reduzir ou parar
Dermatológico	Ectoscopia	Se necessário	Fotossensibilidade	Evitar exposição solar
Endócrino	T4 livre / TSH	Semestral	Hipo/Hipertiroidismo	Tratamento endocrinológico
Hepático	TGO / TGP	Semestral	Elevação > 3x	Reduzir ou parar
Neurológico	Ex. físico	Se necessário	Tremores e ataxia	Reduzir ou parar
Oftalmológico	Ex. oftálmico	Se necessário	Micro depósitos na córnea Neuropatia óptica	Orientação oftalmológica
Pulmonar	RXT / TC / CDMO	Se tosse ou dispneia	Toxicidade pulmonar	Suspender amiodarona Usar corticosteroide

BAV: bloqueio atrioventricular; CDMO: capacidade de difusão pelo teste com monóxido de carbono; RXT: RX de tórax; TC: tomografia computadorizada dos pulmões; TdP: torsades de pointes com aumento do intervalo QT.

#### 11.1.8. Prevenção de Choques Elétricos Recorrentes em Pacientes Tratados com Cardioversor-Desfibrilador Implantável 

 Em portadores de CDI, múltiplos choques, apropriados ou não, e tempestades elétricas são comuns na CCDC e afetam o prognóstico e a qualidade de vida dos pacientes. Estudo observacional descreveu a evolução de 89 pacientes com CCDC e CDI, a maioria devido à prevenção secundária, por período médio de 12 meses. ^715^ Nesse curto período de acompanhamento, 42% dos pacientes receberam choques apropriados e 15,7% foram acometidos por tempestades elétricas, números muito mais elevados quando comparados aos de portadores de CDI com outras cardiopatias (choques apropriados em 8,4% e 12,1% dos casos de prevenção primária e secundária, respectivamente). Vale ainda lembrar que a FEVE média dos pacientes com CCDC tratados por CDI foi de 40 ± 11%, indicando que significativa parcela dessa subpopulação não tinha disfunção ventricular sistólica grave. ^715^

 A associação de amiodarona com betabloqueadores (preferencialmente propranolol, nadolol ou atenolol, se função ventricular satisfatória, e metoprolol ou carvedilol, se ruim) é considerada a de maior potencial para reduzir a morte arrítmica e o número de terapias elétricas apropriadas ou não, desencadeadas pelo CDI. ^716^ O estudo OPTIC, que não incluiu pacientes com CCDC, mostrou superioridade da associação entre amiodarona e betabloqueadores na prevenção de choques, comparativamente ao sotalol ou a outros betabloqueadores utilizados isoladamente. ^716^ Empiricamente, essa associação farmacológica pode ser indicada para prevenção de recorrência de choques em pacientes com CDI e CCDC. 

**Figura 11.1:**
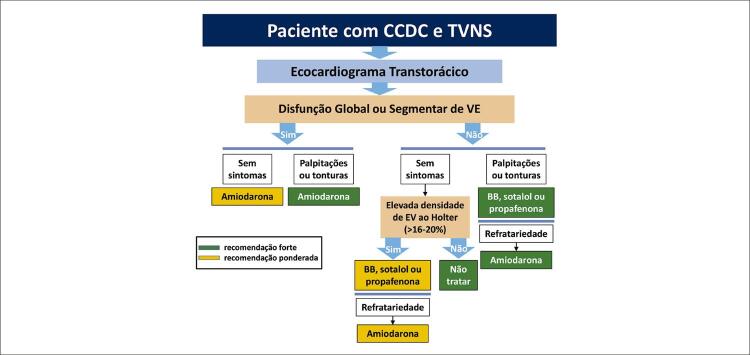
– Algoritmo para abordagem de pacientes com CCDC e TVNS.

 As recomendações para o manuseio farmacológico das arritmias cardíacas e prevenção de morte súbita na CCDC estão representadas na Tabela 11.2 e Figura 11.1 . 

**Tabela 11.2:** – Recomendações para o manuseio farmacológico das arritmias cardíacas e prevenção de morte súbita na CCDC

Sumário das recomendações	Grau de recomendação	Nível de evidência
Tratamento otimizado da IC de acordo com as recomendações desta diretriz para prevenção de arritmias e morte súbita na CCDC	Forte	B
Doses adequadas de IECA, betabloqueadores e inibidores de receptores de mineralocorticoides a pacientes com IC e FE ≤ 40% para redução de morte total e súbita	Forte	B
Associação de betabloqueadores, inibidores de receptores de mineralocorticoides e sacubitril-valsartana em pacientes com IC e FE ≤ 40% para redução de morte total e súbita	Ponderado	B
Correção de hipopotassemia durante tratamento das arritmias ventriculares	Forte	C
Betabloqueadores (succinato de metoprolol, carvedilol ou bisoprolol) associados ou não a amiodarona no tratamento de pacientes com TVNS sintomática e FE ≤ 40%	Forte	B
Betabloqueadores (succinato de metoprolol, carvedilol ou bisoprolol) associados ou não a amiodarona no tratamento de pacientes com TVNS assintomática e FE ≤ 40%	Ponderado	C
Betabloqueadores, sotalol, propafenona e amiodarona (casos refratários) no tratamento de ectopias ventriculares assintomáticas muito frequentes (> 16-20% dos batimentos em Holter 24h), os 3 primeiros na ausência de distúrbios de condução AV, disfunção ventricular, alterações segmentares ou fibrose miocárdica detectável na RMC	Ponderado	C
Betabloqueadores, sotalol, propafenona ou amiodarona (casos refratários) no tratamento de EV sintomáticas, os 3 primeiros na ausência de distúrbios de condução AV, disfunção ventricular, alterações segmentares ou fibrose miocárdica detectável na RMC	Forte	C
Amiodarona no tratamento da TVS hemodinamicamente estável e com FE > 40%	Ponderado	B
Amiodarona no tratamento da TVS com síncope e FE > 40%	Ponderado	B
Amiodarona no tratamento de pacientes com síncope, TVS monomórfica induzida ao EEF e FE > 40%	Ponderado	B
Amiodarona no tratamento de TVS espontânea ou de TVNS sintomática com indução de TVS ao EEF e posterior não indução de TVS após impregnação com fármacos antiarrítmicos (amiodarona ou sotalol)	Ponderado	B
Amiodarona para pacientes com recomendação forte para CDI, mas com expectativa de vida limitada ou sem acesso ao dispositivo	Ponderado	C
Quando utilizada empiricamente, a amiodarona deve ser administrada na menor dose eficaz e os pacientes devem ser submetidos a controle clínico e laboratorial periódico para avaliação de efeitos adversos	Forte	B
Pacientes com EVs assintomáticas e função ventricular esquerda sistólica preservada não requerem tratamento antiarrítmico	Forte	C

CCDC: cardiomiopatia crônica da doença de Chagas; IECA: inibidor da enzima de conversão da angiotensina; FE: fração de ejeção; IC: insuficiência cardíaca; TVNS: taquicardia ventricular não sustentada; TVS: taquicardia ventricular sustentada; RMC: ressonância magnética cardíaca; AV: atrioventricular; EV: extrassístole ventricular; CDI: cardioversor-desfibrilador implantável; EEF: estudo eletrofisiológico.

#### 11.1.9. Tratamento Medicamentoso da Fibrilação Atrial na Cardiomiopatia Crônica da Doença de Chagas 

 A FA e a IC frequentemente coexistem. De acordo com o Framingham Heart Study, aproximadamente 40% dos pacientes com FA desenvolverão IC e vice-versa. ^717^ Na ICFEr, a prevalência de FA aumenta com o agravamento da classe funcional (NYHA), variando entre 4,2% na classe I e 49,8% na classe IV. ^718^ O surgimento de FA associa-se ao aumento da mortalidade por todas as causas em pacientes com IC de qualquer etiologia, incluindo a CCDC. 

 A prevalência de FA na CCDC está aumentada em comparação com a de FA na população geral. Meta-análise de 49 estudos, incluindo 34.023 pacientes, revelou que a prevalência de FA na CCDC era duas vezes maior do que na população geral. ^719^ Mas essa prevalência na CCDC não parece ser superior àquela em pacientes com outras cardiomiopatias estruturais. ^720^

 O tratamento farmacológico da FA no paciente com CCDC é dificultado pelo comprometimento da função sistólica biventricular e por distúrbios do automatismo e dromotropismo elétrico. Por isso, a otimização da terapêutica para IC é mandatória e o uso de IECA ou BRA na ICFEr pode reduzir a incidência de FA. ^721^

#### 11.1.10. Tratamento na Sala de Emergência

 A conduta inicial em pacientes admitidos na sala de emergência com FA de alta resposta ventricular é o controle da FC e a anticoagulação com medicações apropriadas. Em seguida, avalia-se a indicação da reversão da arritmia. 

 A frequência ventricular da FA em pacientes com CCDC muitas vezes é baixa, mas, se houver instabilidade hemodinâmica com taquicardia, a conduta mais apropriada pode ser a anticoagulação imediata, seguida da cardioversão elétrica. Pacientes sintomáticos, porém estáveis, e com FA de duração < 48 horas, sem trombose mural detectável por ecocardiografia transesofágica, podem ser cardiovertidos com propafenona ou amiodarona. Pacientes com duração de FA ≥ 48 horas ou desconhecida, ou ainda com histórico de FA refratária, devem, inicialmente, ser anticoagulados e medicados para controle da FC. Pacientes assintomáticos e/ou com FC baixa e aqueles com intensa dilatação atrial devem, em geral, ser somente anticoagulados. ^722^

#### 11.1.11. Tratamento Ambulatorial

***11.1.11.1. Reversão para Ritmo Sinusal***

 A estratégia de reversão da FA é usualmente mais apropriada quando a FA é de início recente, ocorre em pacientes mais jovens, muito sintomáticos, com átrios pouco dilatados e com resposta ventricular elevada. Quando a IC se desenvolve ou agrava, pode também indicar a necessidade de reversão do ritmo com amiodarona ou mesmo ablação por cateter. ^723^ A amiodarona pode ser especialmente indicada quando, além da FA, os pacientes com CCDC apresentarem arritmias ventriculares, o que é comumente observado no contexto. 

***11.1.11.2. Controle da Frequência Cardíaca***

 A estratégia de controle cronotrópico, sem reversão a ritmo sinusal, é geralmente mais indicada quando há FA de longa duração ou com muita dilatação de câmaras e em pacientes muito idosos, com múltiplas comorbidades e recorrências da arritmia. Quando os betabloqueadores são insuficientes para o controle da resposta ventricular, pode-se considerar a adição de digoxina. Deve-se ressaltar que os bloqueadores de canais de cálcio são contraindicados em pacientes com ICFEr. A amiodarona pode ser ocasionalmente usada para controle cronotrópico se houver contraindicação para betabloqueadores e bloqueadores de canais de cálcio, sem possibilidade de ablação por cateter. ^722^

 As recomendações para o tratamento farmacológico da FA na CCDC estão representadas na Tabela 11.3 . 

**Tabela 11.3:** – Tratamento farmacológico da fibrilação atrial na CCDC

Sumário de recomendações	Fármaco antiarrítmico	Grau de recomendação	Nível de evidência
Reversão de FA de início recente em pacientes sem evidências de alterações cardíacas estruturais ou funcionais	Propafenona (ou outros fármacos do grupo I)	Forte	B
Reversão de FA de início recente, mas já com alterações estruturais e/ou funcionais cardíacas	Amiodarona	Forte	B
Manutenção do ritmo sinusal pós-reversão de FA persistente ou na FA paroxística, na ausência de evidências de alterações cardíacas	Propafenona (ou outros fármacos do grupo I)	Ponderado	B
Manutenção do ritmo sinusal pós-reversão de FA persistente ou na FA paroxística, mas já com alterações cardíacas estruturais ou funcionais	Amiodarona	Ponderado	B
	Não utilizar propafenona (ou outros fármacos do grupo I)	Forte	B
Controle da FC na FA com ou sem IC associada, desde que não haja contraindicação	Betabloqueadores	Forte	B
Controle da FC em pacientes sintomáticos com alta FC, impossibilitados de ablação por cateter e com contraindicação a betabloqueadores e bloqueadores de canais de cálcio	Amiodarona	Ponderado	B
	Digoxina	Forte	B

CCDC: cardiomiopatia crônica da doença de Chagas; FA: fibrilação atrial; FC: frequência cardíaca; IC: insuficiência cardíaca.

## 11.2. Marca-passo, Cardioversor-Desfibrilador e Ressincronizador 

### 11.2.1. Marca-passo Cardíaco Artificial

 Os processos de inflamação, necrose e reação fibrótica que acompanham a desorganização grave da arquitetura e estrutura do miocárdio na CCDC acometem não apenas as fibras contráteis, mas também o sistema nervoso autonômico e o tecido gerador e condutor do impulso elétrico no coração. ^724 , 725^

 O acometimento do nó sinusal ocorre precocemente no curso da CCDC e sua substituição pela reação fibrótica provoca diferentes expressões de doença do nó sinusal. A manifestação mais frequente é a bradicardia sinusal. A CCDC também provoca bloqueios intraventriculares, dentre os quais predomina o BRD isolado ou associado ao BDASE. ^724 , 726^ O BAV também é comum, apresentando-se sob todos os graus e podendo ser assintomático ou causar lipotimia, síncope e mesmo IC ou morte súbita. ^727^ De acordo com o Registro Brasileiro de Marca-passos, a CCDC é a primeira causa de BAV na América Latina, sendo responsável por cerca de 25% das indicações de MP. ^728^

 A prevalência do uso de MP em pacientes com CCDC foi relatada em poucas coortes, que mostraram taxas variando entre 3,5% e 14,1%. ^330^ Em síntese, doença do nó sinusal e BAVT são as bradiarritmias mais comumente tratadas com implante de MP em pacientes com CCDC. ^729 , 730^ A indicação de implante de MP em pacientes com BAVT de etiologia da DC, desde seu início na década de 1970, pode ser considerada como obedecendo ao princípio de plausibilidade extrema. De fato, a evidência de nítido benefício pelo implante do MP consistiu tão somente no estudo observacional das curvas de sobrevida historicamente comparadas de 147 pacientes seguidos antes (sobrevida de apenas 70%, 37% e 6% após 1, 5 e 10 anos de acompanhamento, respectivamente) com as de 74 pacientes seguidos após o advento do dispositivo (sobrevida significativamente maior de 86%, 57% e 44% após 1, 5 e 10 anos de acompanhamento, respectivamente, p < 0,05). ^352^

 Poucos estudos reportaram características antropométricas e epidemiológicas ou os preditores de mortalidade de pacientes com MP e CCDC. Um estudo de coorte prospectiva, publicado em 2018, incluiu 396 portadores de MP que foram acompanhados por, pelo menos, 24 meses. A média de idade foi de 62,5±12,0 anos, sendo a maioria do sexo feminino (64%). Cerca de 95% dos pacientes estavam em classe funcional I ou II (NYHA). Aproximadamente 75% apresentavam BAV avançado como indicação para implante de MP, sendo que a estimulação de VD ocorreu em 82,2% dos casos. A taxa de mortalidade anual foi de 8,4%. ^731^

 É importante destacar o potencial papel protetor de se evitar estimulação ventricular desnecessária e considerar-se a indicação da estimulação direta do sistema de condução, uma modalidade mais fisiológica, mas ainda não testada adequadamente na CCDC. ^732 - 736^

 De modo geral, as indicações de MP na CCDC não diferem das clássicas aplicadas a cardiopatias de outras etiologias. ^13 , 737^ As Tabelas 11.4 , 11.5 e 11.6 incluem os critérios utilizados para implante desses dispositivos. 

**Tabela 11.4:** – Indicações para implante de marca-passo na CCDC: disfunção do nó sinusal

Sumário das recomendações	Grau de recomendação	Nível de evidência
DNS espontânea e irreversível com síncope, pré-síncope, tonturas ou IC claramente relacionadas a bradicardia sinusal (< 40 bpm) ou pausas sinusais > 3,0s em vigília	Forte	C
DNS induzida por fármacos essenciais com síncope, pré-síncope, tonturas ou IC claramente relacionadas a bradicardia (< 40 bpm) ou pausas sinusais > 3,0s em vigília	Forte	C
DNS com sintomas de BFC claramente relacionados à incompetência cronotrópica	Forte	C
Síndrome bradicardia-taquicardia sem indicação de ablação por cateter ou com recusa do paciente	Forte	C
Pausa sinusal > 6,0s em paciente com sintomas de BFC	Forte	C
DNS espontânea e irreversível em paciente com síncope, pré-síncope ou tonturas provavelmente relacionadas a bradicardia, mas cuja associação não foi claramente documentada	Ponderado	C
DNS induzida por fármacos essenciais em pacientes com síncope, pré-síncope ou tonturas provavelmente relacionadas a bradicardia, mas cuja associação não foi claramente documentada	Ponderado	C
Bradiarritmia sinusal que desencadeia ou agrava IC, angina de peito ou taquiarritmias	Ponderado	C
Bradicardia (FC < 40 bpm) em vigília com sintomas leves, não definitivamente associados a bradiarritmia	Ponderado	C
Paciente com pausa assintomática > 6,0s	Ponderado	C
DNS assintomática ou com sintomas comprovadamente não relacionados a bradicardia – MP não indicado	Forte	C
Bradicardia sinusal ou pausas sinusais por uso de fármacos não essenciais ou substituíveis – MP não indicado	Forte	C
Pausas sinusais ou bradiarritmia sinusal exclusivamente durante o sono – MP não indicado	Forte	C

BFC: baixo fluxo cerebral; CCDC: cardiomiopatia crônica da doença de Chagas; DNS: disfunção do nó sinusal; FC: frequência cardíaca; IC: insuficiência cardíaca; MP: marca-passo.

**Tabela 11.5:** – Indicações para implante de marca-passo na CCDC: bloqueios atrioventriculares

Sumário das recomendações	Grau de recomendação	Nível de evidência
BAVT, BAV avançado, BAV de 2º grau Mobitz II, irreversíveis, permanentes ou intermitentes, independentes de sintomas e duração de QRS	Forte	C
BAV de 2º grau Mobitz I, de causa irreversível, permanente ou intermitente, com sintomas definidos de BFC, consequentes a bradicardia	Forte	C
FA ou *flutter* atrial com FC < 40 bpm, irreversível, com sintomas definidos de BFC, consequentes a bradicardia	Forte	C
BAVT ou BAV avançado intra ou infra-his, induzido por estimulação atrial ou teste farmacológico	Forte	C
FA ou *flutter* atrial com FC média < 40 bpm, em vigília, irreversível, ou consequente a fármacos essenciais, em pacientes assintomáticos	Ponderado	C
BAV de 2º grau Mobitz I (até períodos de condução 2:1), irreversível, com indicação de antiarrítmico ou betabloqueador, em pacientes assintomáticos	Ponderado	C
BAVT, BAV avançado, intermitentes e reversíveis, ou consequentes a medicação não essencial – MP não indicado	Forte	C
BAV de 1º grau, BAV de 2º grau tipo Mobitz I e BAV 2:1 assintomáticos e supostamente nodal AV – MP não indicado	Forte	C

AV: atrioventricular; BAV: bloqueio atrioventricular; BAVT: bloqueio atrioventricular total; BFC: baixo fluxo cerebral; CCDC: cardiomiopatia crônica da doença de Chagas; FA: fibrilação atrial; FC: frequência cardíaca; MP: marca-passo.

**Tabela 11.6:** – Indicações para implante de marca-passo na CCDC: bloqueios intraventriculares

Sumário das recomendações	Grau de recomendação	Nível de evidência
Bloqueio de ramo alternante documentado, independente da presença de sintomas	Forte	C
BIV com intervalo HV > 70ms espontaneamente ao EEF em paciente com síncope, pré-síncope ou tontura de etiologia desconhecida	Forte	C
BIV com intervalo HV > 100ms espontaneamente ao EEF em paciente assintomático	Forte	C
Bloqueio bifascicular sem documentação de BAVT intermitente em paciente com síncope, pré-síncope ou tontura de repetição, de etiologia desconhecida	Ponderado	C
Bloqueio de ramo ou bifascicular com ou sem BAV de 1º grau associado em paciente assintomático – MP não indicado	Forte	C

BAV: bloqueio atrioventricular; BIV: bloqueio intraventricular; BAVT: bloqueio atrioventricular total; CCDC: cardiomiopatia crônica da doença de Chagas; EEF: estudo eletrofisiológico; MP: marca-passo.

### 11.2.2. Cardioversor-Desfibrilador Implantável na CCDC 

***11.2.2.1. Prevenção Primária de Morte Súbita Cardíaca***

 O sucesso da prevenção primária de morte súbita cardíaca está atrelado ao reconhecimento dos indivíduos de risco mais elevado para esse evento. Nesse sentido, a estratificação de risco de mortalidade geral, que é predominantemente súbita no paciente com CCDC, conta com um instrumento de uso simples e rápido, o escore de RASSI, ^408^ conforme discutido em outro capítulo desta diretriz. 

 Recentemente, adicionaram-se evidências relevantes a respeito do papel da fibrose miocárdica na identificação de indivíduos de alto risco na CCDC. A quantificação de fibrose miocárdica > 12,3g foi reportada como fator de risco independente para o desfecho combinado de mortalidade por todas as causas, TC, estimulação antitaquicardia ou choque apropriado do CDI e morte súbita cardíaca abortada. ^424^ O impacto desse novo fator também se encontra esmiuçado naquele capítulo desta diretriz, em contexto geral da estratificação do risco e sua relação com o escore de RASSI. 

 O estudo da correlação entre estágios da CCDC e causas de mortalidade revela que a morte súbita cardíaca acomete em geral pacientes a partir do estágio B da doença, sendo mais relevante no estágio C e um pouco menos no estágio D, no qual a IC refratária é causa da maioria dos óbitos. Em termos gerais, o principal mecanismo de morte súbita na CCDC é arritmogênico, sendo que a TVS (FV subsequente) é responsável pela imensa maioria dos eventos letais. ^352^ Nesse sentido, as anormalidades estruturais da CCDC, caracterizadas por inflamação, morte celular e fibrose reativa ou reparativa, constituem-se no substrato anatômico mais propício para desencadear a morte súbita cardíaca. Isso porque se criam áreas de condução lenta e se promovem bloqueios unidirecionais propícios à ocorrência de reentrada elétrica. As EV, frequentes na CCDC, atuam como disparadores desses circuitos, desencadeando a TV/FV. ^738^

 As evidências científicas a respeito da prevenção primária de morte súbita cardíaca na CCDC com uso de fármacos antiarrítmicos (basicamente amiodarona) são escassas e já foram discutidas anteriormente. Com relação ao CDI, existe apenas o relato dos achados de uma série de 13 casos, que não permite conclusões sobre eficácia terapêutica. ^739^

 Embora o papel da estimulação ventricular programada na estratificação de risco de pacientes com CCDC ainda não esteja bem estabelecido, Silva *et al* . ^480^ demonstraram, em estudo com 78 pacientes com TVNS e síncope ou pré-síncope (média de idade de 46 anos, FEVE média de 47%, 58% dos pacientes do sexo masculino e 85% em classe funcional I/II), durante seguimento médio de 56 meses, que a indução de TVS monomórfica em 25 pacientes (32%), todos posteriormente tratados com amiodarona, foi preditora da ocorrência de TV espontânea e de mortalidade cardíaca e total. 

 Conforme também já relatado nesta diretriz, Leite *et al* . ^481^ demonstraram que, em pacientes com TVNS e indução de TVS (n = 37) ou naqueles com TVS espontânea (n = 78), o EEF poderia predizer a eficácia de antiarrítmicos da classe III (principalmente amiodarona) a longo prazo. Imediatamente após impregnação oral com os fármacos antiarrítmicos, a indução de TVS hemodinamicamente instável esteve relacionada à maior mortalidade total, cardíaca e súbita, quando se comparou esses pacientes com aqueles nos quais não se conseguiu induzir a arritmia ou a arritmia induzida foi a TVS bem tolerada. 

 Esses dois estudos, apesar de observacionais, sugerem que o EEF poderia identificar pacientes com taquiarritmias ventriculares, que, uma vez tratados com fármacos antiarrítmicos, evoluiriam com pior prognóstico e maior risco de óbito e, nesses casos, o CDI poderia ser alternativa viável. 

 Sumariamente, pode-se afirmar que, até o momento, não há evidências científicas que lastreiam o uso do CDI, com recomendação forte na prevenção primária de morte súbita cardíaca na CCDC. Nesse sentido, o estudo CHAGASICS, em andamento, deverá fornecer brevemente, informações relevantes. ^460^ Trata-se de ECR, multicêntrico e aberto, desenhado para comparar os efeitos do CDI com a amiodarona na prevenção primária de mortalidade na CCDC, em pacientes com TVNS ao Holter de 24 horas e escore de RASSI ≥ 10 pontos. As indicações para implante de CDI em prevenção primária de morte súbita cardíaca estão listadas na Tabela 11.7 . 

**Tabela 11.7:** – Indicações para implante de CDI na CCDC: prevenção primária de morte súbita cardíaca

Sumário das recomendações	Grau de recomendação	Nível de evidência
TVNS com síncope ou pré-síncope de provável etiologia cardíaca e indução de TVS hemodinamicamente instável ao EEF	Ponderado	B
TVNS com indução de TVS ao EEF, seguida de impregnação com amiodarona e repetição de EEF com indução de TVS hemodinamicamente instável	Ponderado	B

CCDC: cardiomiopatia crônica da doença de Chagas; CDI: cardioversor-desfibrilador implantável; EEF: estudo eletrofisiológico; TVNS: taquicardia ventricular não-sustentada; TVS: taquicardia ventricular sustentada.

***11.2.2.2. Prevenção Secundária de Morte Súbita Cardíaca***

 Considera-se, em geral, que o CDI seja recurso aplicável a alguns contextos de prevenção secundária de morte súbita cardíaca para pacientes com CCDC. Sua eficácia consiste na interrupção do evento arrítmico ameaçador da vida por meio de eletrochoque ou estimulação ventricular rápida (antitaquicardia), evitando a ocorrência de parada cardíaca e óbito subsequente, embora algumas arritmias abortadas pelo CDI pudessem reverter espontaneamente, não necessariamente culminando em óbito. A escolha dessa opção terapêutica envolve a análise rigorosa de cinco fatores essenciais: 1. adjudicação da parada cardíaca ou evento arrítmico (TVS ou FV) devidamente documentado e sua correlação com irreversibilidade da causa; 2. convicção de que a terapêutica clínica e/ou procedimentos menos invasivos, de similar eficácia, estão esgotados; 3. certificação de que o tratamento pleno da cardiopatia de base está sendo implementado; 4. valorização da estratificação de risco da cardiomiopatia de base; e 5. condição clínica do paciente, expressa principalmente pelo grau de disfunção ventricular (FEVE) e tipo de sintoma relacionado à arritmia. 

 Esses fatores foram pouco contemplados nos estudos de prevenção secundária de morte súbita cardíaca na CCDC. Não existem ECR nessa população e as evidências científicas se restringem a dados de registros de empresas de dispositivos implantáveis, ^740 , 741^ de estudos clínicos observacionais de centros únicos que avaliaram amostras populacionais pouco extensas ^355 , 742 - 750^ e de meta-análises desses estudos. ^751 , 752^

 A maior coorte de pacientes com CCDC tratados por implante de CDI para prevenção secundária, em centro único, arrolou 116 pacientes consecutivos, com média de idade de 54 anos, sendo 62% do sexo masculino. A FEVE média foi de 42%, 83% dos pacientes estavam em classe funcional I/II da NYHA e o motivo do implante de CDI foi a reversão de parada cardíaca em 18% e TVS sintomática em 82% dos casos. Em seguimento médio de 45 meses, foram reportados: taxa de mortalidade total anual de 7,1%; terapias apropriadas em 50% e de inapropriadas em 11% da população. Os fatores independentes de pior prognóstico foram classe funcional III da NYHA e baixa FEVE. Pacientes com taxa de estimulação do VD superior a 40% também tiveram menor sobrevida. ^746^

 Por outro lado, em coorte retrospectiva de 90 pacientes consecutivos com CCDC (68% do sexo masculino, média de idade de 59 anos e FEVE média de 47%) tratados por implante de CDI, cerca de 30% dos quais tinham função cardíaca preservada, foi surpreendente observar que, em seguimento médio de 756 dias, a taxa de mortalidade anual foi elevada (16,1%), ainda que, dos pacientes que faleceram, 88% estivessem em classe funcional I no momento do implante de CDI. Apesar de 65% dos pacientes receberem choque apropriado e terapia antitaquicardia, a taxa mensal de choques foi o único preditor independente de mortalidade. ^743^

 A taxa de mortalidade de outra coorte retrospectiva de 76 pacientes com CCDC, portadores de CDI, foi comparada com a de uma série histórica de 28 pacientes com TVS tratados apenas com amiodarona. ^747^ Reportou-se 72% de redução de mortalidade total e 95% de redução de morte súbita cardíaca na coorte tratada com CDI. Entretanto, quando se realizou a análise de subgrupo, houve importante interação entre a FEVE e o benefício do CDI. Enquanto pacientes com FEVE reduzida (< 40%) obtiveram benefício significativo e expressivo com o CDI, aqueles com FEVE relativamente preservada (≥ 40%) obtiveram pouco ou nenhum benefício. ^747^

 Esses dados são consistentes com os resultados da meta-análise de ECR de prevenção secundária em outras cardiopatias (AVID, CIDS e CASH), que mostrou redução de mortalidade total e súbita com o CDI (em comparação à amiodarona) apenas em pacientes com FEVE < 35%. ^701^

 Vale ressaltar que meta-análise incluindo esse estudo e outros cinco observacionais na CCDC não demonstrou diferença de mortalidade total entre uso de amiodarona (9,6%/ano) e CDI (9,7%/ano). ^751^

 Recentemente, foi publicada revisão sistemática e meta-análise de 13 estudos observacionais de pacientes com CCDC para reavaliar a eficácia global do CDI na prevenção de morte total e súbita. Foram incluídos 1.041 pacientes, 92% de prevenção secundária e apenas 8% de prevenção primária, com idade média de 57 anos, 64% do sexo masculino, FEVE média de 38%, 79% em classe funcional I/II, 79% em uso de amiodarona e 44% em uso de betabloqueador. Em seguimento de 2,8 anos, a taxa de mortalidade total foi de 9,0% ao ano e a taxa de morte súbita cardíaca foi de 2,0% ao ano, em 2,6 anos de seguimento. Terapias do CDI apropriadas (choques ou intervenções antitaquicardia) ocorreram em 24,8% dos pacientes, anualmente. Taxas elevadas de choques inapropriados (4,7%/ano) e de tempestades arrítmicas (9,1%/ano) também foram observadas. ^752^

 Em relação ao prognóstico dos tipos de arritmias que usualmente indicam implante de CDI, Lima *et al* . ^753^ compararam o comportamento clínico evolutivo de dois grupos de pacientes: grupo 1, constituído de 318 pacientes, dos quais 36% com CCDC, cujo motivo do implante foi a TVS sintomática (síncope e/ou instabilidade hemodinâmica) ou a indução de TVS ao EEF em pacientes com síncope recorrente de etiologia não esclarecida; e grupo 2, constituído de 97 pacientes, dos quais 15% com CCDC, cujo motivo do implante foi a recuperação de parada cardíaca por FV ou TVS sem pulso. Enquanto sexo masculino (75% *versus* 73%) e classe funcional I/II da NYHA (77% *versus* 76%) não diferiram entre os pacientes dos grupos 1 e 2, a média de idade foi maior (57 *versus* 51 anos, p = 0,0004) e a FEVE média menor (38% *versus* 43%, p = 002) nos pacientes do grupo 1. Após seguimento médio de 24 meses para o grupo 1 e de 26 meses para o grupo 2, houve maior mortalidade no grupo 2 (24,7% *versus* 13,5%, p < 0,005), com ocorrência similar de choques apropriados pelo CDI (31% dos pacientes do grupo 1 *versus* 26% daqueles do grupo 2, p = 0,09), denotando, possivelmente, maior gravidade da arritmia no subgrupo de pacientes recuperados de parada cardíaca. 

 Leite *et al* ., ^754^ por sua vez, avaliaram o impacto da presença de síncope na mortalidade total e cardíaca de 78 pacientes com TVS monomórfica (média de idade de 53 anos, 58% do sexo masculino, FEVE média de 50%, 88% em classe funcional I/II). Síncope durante TVS foi observada em 45 pacientes (58%) e esteve ausente em 33 (42%). Após seguimento médio de 49 meses, não houve diferença na mortalidade total (33% *versus* 39%) e cardíaca (27% *versus* 30%), nem na recorrência de TVS não fatal (58% *versus* 54%) entre os pacientes com e sem síncope, respectivamente. Entretanto, a presença de síncope durante as recorrências foi significativamente maior entre os pacientes que apresentaram o sintoma inicialmente (65% *versus* 18%, p < 0,01). Assim, na CCDC, síncope durante apresentação clínica da TVS monomórfica parece não estar associada a um aumento de mortalidade total e cardíaca. 

 Com base no conjunto dos resultados sumarizados acima, pode-se concluir que o uso de CDI para prevenção secundária de morte súbita cardíaca em pacientes com CCDC ainda carece de embasamento mais sólido em evidências científicas. Esse cenário negativo, idealmente, deveria ser resolvido pela execução de um ECR. Todavia, vários investigadores alegam impedimentos de ordem ética para adoção desse caminho científico e não há, nos dias atuais, perspectiva para tal. 

 Por outro lado, também se alega que existe ampla experiência positiva acumulada ao longo dos anos com o uso de protocolos referendados por diretrizes internacionais e de âmbito nacional para pacientes com CMI ou CMD tratados com implante de CDI. Isso criou um cenário favorável à extrapolação dessas regras na prática clínica, no sentido de pacientes com CCDC serem mais liberalmente tratados com CDI. Em contraposição, deve-se reafirmar que a prevenção secundária com implante de CDI na CCDC deve ser sempre respaldada em criteriosa decisão individualizada paciente a paciente, de análise de risco/benefício. 

 Esse princípio geral, por sua vez, deriva de duas noções essenciais: a primeira é que, mesmo para os cenários mais consolidados em diretrizes internacionais de pacientes com outras cardiopatias, o benefício do CDI torna-se relativamente restrito à vigência de grave disfunção ventricular sistólica, sendo muito menos significativo na ausência desse fator. A outra noção já ressaltada acima é que a complexa e peculiar fisiopatologia da CCDC implica em que, dificilmente, princípios terapêuticos apenas em parte validados em contextos de outras cardiopatias possam ser adequadamente extrapolados para a própria CCDC. Assim, tanto a FEVE, tomando como ponto de corte ideal o valor de 40%, quanto o tipo de arritmia e sintoma associado foram valorizados para balizar melhor as indicações de CDI na prevenção secundária de morte súbita cardíaca. 

**Figura 11.2:**
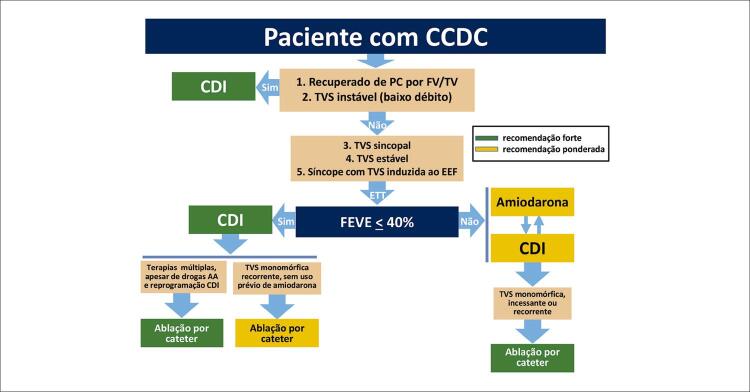
– Algoritmo para abordagem de pacientes com CCDC e taquiarritmias ventriculares sustentadas. AA: antiarrítmicas; CCDC: cardiomiopatia crônica da doença de Chagas; CDI: cardioversor-desfibrilador implantável; EEF: estudo eletrofisiológico; FEVE: fração de ejeção ventricular esquerda; FV: fibrilação ventricular; PC: parada cardíaca; TVS: taquicardia ventricular sustentada.

 É oportuno mencionar que, quando esta diretriz estava sendo finalizada, a recente publicação da *European Society of Cardiology* ^755^ para tratamento de arritmias ventriculares dedicou explícita menção à CCDC e restringiu sobremaneira as indicações de CDI no contexto, de forma praticamente análoga às nossas recomendações. As recomendações desta diretriz estão listadas na Tabela 11.8 e na Figura 11.2 . 

**Tabela 11.8:** – Indicações para implante de CDI na CCDC: prevenção secundária de morte súbita cardíaca

Sumário das recomendações	Grau de recomendação	Nível de evidência
Recuperado de parada cardíaca por FV ou TVS sem pulso documentada (excluindo-se causas reversíveis e expectativa de vida < 1 ano), independente da FE	Forte	B
TVS hemodinamicamente instável (baixo débito), independente da FE	Forte	B
TVS com síncope e FE ≤ 40%	Forte	B
Síncope com TVS monomórfica induzida ao EEF e FE ≤ 40%	Forte	B
TVS hemodinamicamente estável e FE ≤ 40%	Forte	B
TVS com síncope e FE > 40%	Ponderado	B
Síncope com TVS monomórfica induzida ao EEF e FE > 40%	Ponderado	B
TVS hemodinamicamente estável e FE > 40%	Ponderado	B

CCDC: cardiomiopatia crônica da doença de Chagas; CDI: cardioversor-desfibrilador implantável; EEF: estudo eletrofisiológico; FE: fração de ejeção; FV: fibrilação ventricular; TVS: taquicardia ventricular sustentada.

### 11.2.3. Terapia de Ressincronização Cardíaca

 Também não existem dados robustos a partir de ECR para embasar a utilização da terapia de ressincronização cardíaca (TRC) na CCDC. A TRC tem sido recomendada a pacientes com CMD e CMI, apresentando IC avançada, disfunção sistólica grave e dissincronia ventricular, traduzida particularmente por complexo QRS alargado. Nesse contexto, essa modalidade tem sido descrita como agindo positivamente sobre o remodelamento ventricular esquerdo e promovendo redução significativa da classe funcional de IC e melhora da qualidade de vida, com base em diversos outros parâmetros funcionais. ^756 - 760^

 Alguns estudos evidenciaram benefício do procedimento quanto à redução de mortalidade por IC, ^756 , 761 , 762^ especialmente na vigência de BRE, FEVE ≤ 35%, duração de QRS ≥ 130ms e insuficiência mitral. ^763 , 764^ Entretanto, como na CCDC a prevalência de BRE é baixa, tal fato limita a indicação formal para TRC nesse cenário. A presença e a extensão da fibrose miocárdica, que se associa a pior prognóstico independentemente da FEVE, ^424 , 428 , 765^ as arritmias ventriculares frequentes, a regurgitação tricúspide e a disfunção de VD são exemplos de outros fatores desfavoráveis à TRC na CCDC, que podem colocar os pacientes em maior risco de não resposta. 

 Ademais, é importante ressaltar que o implante de MP convencional (unicameral), muito utilizado na CCDC, provoca inerente dissincronia do VE (“BRE induzido”), sobretudo quando o cabo-eletrodo está localizado na região apical do VD. Isso se associa a prejuízos hemodinâmicos e agrava o prognóstico do paciente com IC tratado com MP. ^766 , 767^

 Até o momento, apenas cinco estudos observacionais, ^768 - 772^ todos de centros únicos, avaliaram a evolução clínica de pacientes com CCDC submetidos à TRC e três deles ^770 - 772^ compararam o efeito dessa terapia entre pacientes com CCDC e com outras cardiopatias ( Tabela 11.9 ). Redução estatisticamente significativa do DDVE e melhora significativa da classe funcional (NYHA) e da FEVE foram observadas com a TRC nos dois estudos que incluíram apenas pacientes com CCDC. ^768 , 769^ A mortalidade anual por todas as causas nesses dois estudos variou entre 9,0% e 9,2%. Nos outros três estudos, ^770 - 772^ a sobrevida de pacientes com CCDC foi significativamente menor e o percentual de pacientes não respondedores à TRC foi significativamente maior quando comparados aos de pacientes com as demais cardiomiopatias. Em pacientes submetidos à TRC, a presença de CCDC aumentou o risco de óbito em cerca de 2 a 4 vezes. ^770 , 772^

**Tabela 11.9:** – Estudos observacionais de terapia de ressincronização cardíaca na CCDC.

CARACTERÍSTICAS	Araujo et al. 2014 ^**55**^	Menezes et al. 2018 ^**56**^	Martinelli et al. 2018 ^**57**^			Scorzini et al. 2018 ^**58**^			Passos et al. 2019 ^**59**^	
População	CCDC	CCDC	CCDC	CMI	CMD	CCDC	CMI	Outras	CCDC	Não CCDC
Nº de pacientes	72	50	115	134	177	42	13	43	13	41
Sexo masculino (%)	ND	56	65	83	51	59,5	92	56	31	66
Média de idade (anos)	ND	63	57	68	60	60	66	58	65	62
Tipos de bloqueios:										
• BRE induzido (%)	15	30	74	31	17	21	0	5	ND	ND
• BRE espontâneo (%)	47	30	11	63	78,5	39	92	87	ND	ND
• Não BRE (%)	38	40	15	7	4,5	39	8	8	ND	ND
TRC-CDI (%)	ND	74	23,5	33	26	31	31	26	0	0
Fibrilação ou flutter atrial (%)	0	16	25	16	15	14	15	14	0	0
Classe funcional III/IV (%)										
• Pré-implante	100	82	82	82	88	87,5	67	80	77	63
• Pós-implante	13	18	43,5	26	26	50	33	24	ND	ND
FEVE média (%)										
• Pré-implante	27	29	26	26	24	26	27	24	27	26
• Pós-implante	44	39	27	28	29	26	34	30	ND	ND
Largura média do QRS (ms)										
• Pré-implante	148	150	163	164	162,5	161	154	160	ND	ND
• Pós-implante	ND	116	ND	ND	ND	139	134	135	ND	ND
DDFVE (mm)										
• Pré-implante	66	ND	66	69	74	68	68	73	ND	ND
• Pós-implante	65	ND	68	68	71	65	65	69	ND	ND
Seguimento médio (meses)	47	61	29	29	29	27	42	35	15	15
Não respondedores (%)	33	34	43,5	26	26	47	33	35	ND	ND
Mortalidade anual (%)	9,0	9,2	25,4	11,3	10,4	25,6	4,8	13,9	18,4	3,2

BRE: bloqueio completo de ramo esquerdo; CCDC cardiomiopatia crônica da doença de Chagas; CMD cardiomiopatia dilatada; CMI cardiomiopatia isquêmica; DDFVE diâmetro diastólico final de ventrículo esquerdo; FEVE fração de ejeção de ventrículo esquerdo; ND não disponível; TRC-CDI terapia de ressincronização cardíaca associada a cardioversor-desfibrilador implantável.

 Embora plausível, a utilidade da TRC como *upgrade* de MP também permanece controversa. Enquanto o estudo COMBAT, cuja casuística contou com 51,6% de pacientes com CCDC, descreveu melhora significativa na qualidade de vida e aumento da FEVE em pacientes submetidos à TRC em relação à estimulação simples de VD, ^773^ o estudo RAFT, que não incluiu pacientes com CCDC, não evidenciou benefício com relação à mortalidade quando 54 pacientes receberam TRC em comparação com outros 81 tratados apenas com estimulação de VD. ^762^

 Nesse contexto, ECR de pequena dimensão incluiu 50 pacientes e relatou benefício em termos de qualidade de vida, quando a TRC foi adicionada à estimulação isolada do VD em pacientes obrigatoriamente tratados com implante de MP. ^774^

 Em síntese, inexistem evidências científicas específicas e consistentes para apoiar a indicação da TRC na CCDC, o que somente será possível por meio da realização de ECR comparando TRC associada à otimização do tratamento clínico *versus* grupo controle apenas otimizado medicamentosamente. Por ora, esse recurso deve ter sua aplicação derivada de extrapolação a partir de estudos realizados em pacientes com CMI e CMD mediante criteriosa seleção e individualização baseada em análise de risco/benefício para o paciente com CCDC ( Tabela 11.10 ). 

**Tabela 11.10:** – Indicação de TRC na CCDC

Sumário das recomendações	Grau de recomendação	Nível de evidência
IC sintomática, classe funcional II e III, com FEVE ≤ 35%, ritmo sinusal, com morfologia de BRE e duração de QRS ≥ 130ms, apesar de terapêutica otimizada, para reduzir morbidade e mortalidade	Ponderado	B

BRE: bloqueio de ramo esquerdo; CCDC: cardiomiopatia crônica da doença de Chagas; FEVE: fração de ejeção ventricular esquerda; IC: insuficiência cardíaca; TRC: terapia de ressincronização cardíaca.

## 11.3. Métodos de Ablação

### 11.3.1 Taquicardia Ventricular Sustentada: Apresentação Clínica, Mecanismos Eletrofisiológicos e Localizações 

 As manifestações clínicas das arritmias ventriculares na CCDC são heterogêneas, desde formas assintomáticas, ou com discretos sintomas, até quadros de taquicardias incapacitantes com síncope, choques mal tolerados do CDI, tempestade elétrica e, eventualmente, a própria morte súbita. ^46 , 354 , 442 , 747 , 752 , 775^ Como destacado em outros capítulos desta diretriz, embora o mais comum seja a coexistência de manifestações arrítmicas com quadro de IC, é também muito característico da CCDC que ocorram arritmias graves como manifestação inicial ou predominante sem IC. ^353^

 Diversos mecanismos patogenéticos (incluindo a lesão miocárdica causada diretamente pelo parasita ou imunomediada, a denervação autonômica e os distúrbios da microcirculação) ocasionam dano miocárdico e variados distúrbios em todos os níveis de geração e condução da eletricidade cardíaca. ^176 , 270 , 326^

 O mecanismo eletrofisiológico fundamental da TVS na CCDC geralmente é a reentrada do estímulo elétrico em região de cicatriz ventricular, constituída por extensa fibrose intersticial entremeada por fibras miocárdicas viáveis. Isso ocorre mais frequentemente em região inferolateral do VE (70% dos pacientes), podendo localizar-se também na região apical do VE e no VD. ^776 - 779^ Tais áreas de fibrose (cicatrizes) podem ter localização subendocárdica, intramiocárdica ou subepicárdica do VE. ^424 , 428 , 778 - 781^ Adicionalmente, um istmo de miocárdio viável entre o anel mitral e uma cicatriz na região inferolateral do VE pode formar um circuito macrorreentrante de TVS. ^782^ Por fim, um circuito de macrorreentrada envolvendo os ramos direito e esquerdo (reentrada ramo a ramo) pode ser a causa menos comum de TVS. ^783^

 De forma genérica, os diferentes mecanismos reentrantes da TVS têm sido amplamente investigados pelo EEF invasivo, no qual a estimulação ventricular programada é capaz de reproduzir essa arritmia em mais de 80% dos pacientes com história clínica de TVS ou síncope e CCDC. Além disso, o mapeamento endocárdico e/ou epicárdico tem demonstrado a presença de eletrogramas diastólicos anormais, pré-sistólicos e mesodiastólicos, predominando nas regiões de acinesia ou discinesia do VE. ^481 , 776 , 778 , 780 , 781^

 Durante o EEF utilizando técnicas de estimulação ventricular (encarrilhamento oculto), é possível diferenciar o istmo crítico do circuito de reentrada de outras regiões não envolvidas no mecanismo da TV, o que pode ser confirmado pela interrupção da TV durante a ablação por radiofrequência. ^481 , 776 , 778 , 780 , 781^ Além da fibrose em regiões circunscritas da parede ventricular, as lesões do sistema nervoso autônomo intracardíaco, caracterizadas pela depleção neuronal ganglionar e disautonomia cardíaca, e a inflamação miocárdica crônica são alterações fisiopatológicas que podem contribuir para a instabilidade elétrica miocárdica e gênese das taquiarritmias ventriculares. ^193 , 208 , 222 , 784 - 788^

### 11.3.2. Avaliação Clínica e Laboratorial Antes da Ablação 

 Os pacientes com CCDC e TVS geralmente apresentam doença cardíaca avançada ^430^ e IC (que deve ter seu tratamento específico otimizado), demandando, para que se programe a ablação, a avaliação da função renal, ocorrência de infecção e necessidade de medicamentos vasoativos em casos de tempestade elétrica. Em geral, a presença de comorbidades não deve contraindicar a ablação, principalmente nos casos de tempestade elétrica e choques recorrentes, pois, sem a intervenção, a mortalidade é muito elevada. ^743^

 O escore *PAINESD* [doença **P** ulmonar obstrutiva crônica, idade ( ***A*** *ge* ) > 60 anos, cardiomiopatia **I** squêmica, **N** YHA III ou IV, fração de **E** jeção < 25%, tempestade elétrica ( **S** torm) e **D** iabetes mellitus] foi desenvolvido para identificar pacientes que podem apresentar descompensação hemodinâmica durante a ablação de TV ^789^ e maior mortalidade precoce após o procedimento. Como esse escore foi desenvolvido para pacientes com cardiopatias isquêmica e não isquêmica, mas não se incluiu a CCDC, não é aplicável como preditor de mortalidade em 30 dias após a ablação de TV. ^790 , 791^

 Pacientes com CCDC podem também apresentar megaesôfago e/ou megacólon. ^792 , 793^ Como a abordagem na ablação deve ser preferencialmente epicárdica, ^794^ na presença de megacólon, o acesso ao espaço pericárdico pode ser obtido através de janela cirúrgica ou através da punção guiada por laparoscopia. ^795^

 A RMC pelo método de contraste com gadolínio para realce tardio é útil para identificar as áreas de fibrose ^796^ e avaliar se o substrato-alvo está localizado na superfície epicárdica e endocárdica. ^797^ A angiotomografia de coronárias pode mostrar áreas de afilamento ^798^ e hipoperfusão, que estão associadas ao substrato da arritmia. Tanto a RMC quanto a angiotomografia de coronárias avaliam a espessura da gordura epicárdica local e a localização das artérias coronárias, permitindo integração com os sistemas de mapeamento eletroanatômico. ^799^

 Recentemente, foram desenvolvidos *software* de processamento de imagem 3D da RMC, que permitem a definição dos potenciais circuitos das arritmias. ^800^ Essas imagens possibilitam integração com os sistemas de mapeamento eletroanatômico e contribuem para o sucesso da ablação, ^801^ que se torna mais rápida e eficaz, dispensando assim a reconstrução do mapeamento eletroanatômico. ^802^

 Outro ponto importante no planejamento da ablação é a avaliação do ECG de 12 derivações durante a TV clínica, o qual, sempre que possível, deve ser registrado. Isso permite a comparação com as TV induzidas no procedimento, sendo importante na busca da eliminação, pelo menos, da TV clínica, visto que geralmente os pacientes com CCDC apresentam múltiplas morfologias de TV. ^781^ Esse conceito é válido apesar de o ECG apresentar limitações na definição de TV epicárdica. ^803^

 Frequentemente, pacientes com CCDC apresentam recorrências após ablação de TV, sendo comum a realização de múltiplos procedimentos. A informação dos procedimentos anteriores é fundamental no planejamento de nova ablação. Devem-se avaliar os mapas realizados anteriormente para comparar com o mapeamento atual, avaliar se alguma área cicatricial endo- ou epicárdica não foi abordada no procedimento anterior e obter-se a informação de o acesso epicárdico ter sido realizado com sangramentos, pois, nesses casos, pode ocorrer a complicação de aderências epicárdicas. 

### 11.3.3. Técnicas de Mapeamento das Taquicardias Ventriculares 

 Episódios de TV em indivíduos com CCDC apresentam altas taxas de recorrência, mesmo após terapia medicamentosa otimizada. Por exemplo, meta-análise recente relatou taxas de terapias apropriadas e tempestade elétrica de 9% e 25% ao ano, respectivamente, em portadores de CDI por profilaxia secundária. ^752^ Assim, a ablação por radiofrequência torna-se indicada em muitos casos refratários ao tratamento clínico. ^794^

 A cicatriz miocárdica que propicia reentrada e TVS usualmente se localiza nas porções basais das paredes inferior e lateral do VE e o acometimento mesocárdico e epicárdico é frequente. Logo, os resultados iniciais da ablação de TV com abordagem endocárdica apresentaram-se frustrantes, com taxas de sucesso em torno de 17%. ^777 , 804^

 O acesso epicárdico por punção percutânea subxifoide, com agulha de Tuohy guiada por fluoroscopia, foi descrito em 1996 ^804^ e contribuiu para a otimização dos resultados das ablações de TV em pacientes com CCDC. Em ECR, observou-se que a abordagem endocárdica/epicárdica combinada, comparada à abordagem endocárdica exclusiva, correlacionava-se com menor taxa de recorrência, 40% e 80% respectivamente, em 2 anos de seguimento. ^781^

 A complicação mais temível relacionada ao acesso epicárdico percutâneo é o sangramento, que pode ocorrer em cerca de 10% dos casos. A maioria é de pequena monta e está relacionada à punção acidental do VD. Sangramento vultuoso com necessidade de abordagem cirúrgica ocorre em 2% dos casos. Lesões hepáticas e intestinais podem ocorrer durante a punção epicárdica na presença de hepatomegalia significativa e megacólon. Nesses casos, pode-se optar pelo acesso pericárdico cirúrgico ou através de punção subxifoide guiada por videolaparoscopia. ^795^

 Nos últimos anos, surgiram variações da técnica original de punção epicárdica que incluem: micropunção, ^805^ insuflação de dióxido de carbono (CO _2_ ) no apêndice atrial direito ^806^ ou seio coronário, ^807^ agulha com sensor de pressão, ^808^ tomografia computadorizada, ^809^ RMC ^810^ e punção guiada por mapeamento eletroanatômico. ^811^ Dentre essas, vale destacar que, em estudo observacional multicêntrico, a micropunção demonstrou menores taxas de derrame pericárdico volumoso e de necessidade de correção cirúrgica de sangramento, quando comparada à técnica de punção com agulha de maior calibre. ^805^

 Algumas situações podem limitar a eficácia da ablação na superfície epicárdica, como nos casos em que a região-alvo para ablação se localiza sob a gordura epicárdica ou possui proximidade com o trajeto do nervo frênico ou com as artérias coronárias. ^812^

 Devido à gravidade da doença e à complexidade do procedimento, cuidados perioperatórios são importantes para a redução do risco de complicações. A pesquisa prévia de trombos intracavitários é mandatória e a monitorização invasiva da pressão arterial, a infusão de fármacos vasoativos previamente à indução anestésica e o suporte circulatório mecânico em casos selecionados são úteis à otimização hemodinâmica perioperatória. 

 A ablação por cateter pode ser realizada com o paciente em TV ou em ritmo sinusal. Cada estratégia possui vantagens e desvantagens e não existem estudos comparando seus resultados na população com CCDC. Apesar de o procedimento realizado com o paciente em TV favorecer a identificação dos istmos das taquicardias com maior acurácia, a maioria das TV induzidas é mal tolerada hemodinamicamente e necessita de cardioversão elétrica imediata. 

 Porém, mesmo em TV hemodinamicamente estáveis, o tempo de mapeamento deve ser abreviado ao máximo pelo risco de baixo débito pós-intervenção. A ablação por cateter com o paciente em ritmo sinusal tem como objetivo a modificação do substrato que consiste na identificação e eliminação dos possíveis istmos responsáveis pelas taquicardias. Essas áreas estão relacionadas à cicatriz miocárdica, que é identificada como região de baixa voltagem no sistema de mapeamento eletroanatômico e representada pelos potenciais tardios, fragmentados e de baixa amplitude. Embora menos específica, essa técnica tem a vantagem de manter o paciente hemodinamicamente estável por mais tempo durante o procedimento, quando há grave disfunção ventricular. ^813 , 814^

 Vale ressaltar que a evolução tecnológica do sistema de mapeamento eletroanatômico, principalmente com os cateteres de mapeamento de alta definição, aumentou significativamente a acurácia da definição anatômica das regiões de cicatriz, além de sua correlação funcional com a propagação elétrica. Entretanto, estudos relacionados à ablação de TV em pacientes com CCDC são escassos e praticamente não contemplam a tecnologia atualmente disponível. 

### 11.3.4. Desfechos e Complicações Durante o Procedimento de Ablação da Taquicardia Ventricular 

 Historicamente, a estimulação ventricular programada tem sido utilizada como a principal ferramenta para a avaliação da efetividade imediata do procedimento de ablação da TV. ^815^ Entretanto, variadas definições de não indutibilidade (protocolos de estimulação heterogêneos e relevância de indução de TV rápida ou “não clínica”) associadas a uma variação diária espontânea nos resultados da estimulação ventricular programada representam relevantes limitações e deficiências na acurácia dessa ferramenta em predizer o sucesso da ablação no curto e longo prazo. ^816^

 Apesar dessas limitações, a estimulação ventricular programada ao final do procedimento ainda permanece como a principal ferramenta para avaliação do sucesso agudo. ^815^ Os pacientes que permanecem com TV lenta (ciclo > 300ms) induzível ao final do procedimento exibem mais recorrência do que aqueles sem TV indutível. ^817^ Outras estratégias para avaliar o resultado durante o procedimento incluem verificar a eliminação da excitabilidade, ^818^ dos potenciais tardios, ^819 , 820^ dos eletrogramas locais anormais (LAVA), ^821^ ou dos canais da cicatriz, ^822^ bem como constatar a homogeneização do substrato, ^823^ o isolamento central da cicatriz ^824 , 825^ e a lesão guiada por imagem. ^826 , 827^

 As complicações agudas incluem as de natureza vascular, o derrame pericárdico, tamponamento cardíaco, dissociação eletromecânica, BAVT, paralisia do nervo frênico, AVC e morte. ^781 , 828^

### 11.3.5. Resultados da Ablação e Seguimento dos Pacientes 

 Recentemente, reportou-se ECR prospectivo de ablação de TV em pequeno grupo de pacientes com CCDC, sendo a abordagem sistemática epicárdica e endocárdica superior à endocárdica exclusiva, havendo no primeiro grupo recorrência de TV durante seguimento médio de 19 meses da ordem de 40%. ^781^ Essa taxa de recorrência mostrou, portanto, resultado similar à ablação de TV em pacientes com cardiopatias não isquêmicas em geral. ^828^

 A recorrência de TV no período pós-ablação depende de vários fatores, os mais comuns sendo relacionados à utilização de antiarrítmicos, à programação dos dispositivos cardíacos implantáveis e à gravidade da cardiomiopatia. ^815 , 829^ Todos os meios disponíveis para detecção de episódios de TVS devem ser empregados, incluindo uma zona de monitorização pelo CDI, capaz de detectar as TVS lentas induzidas durante a ablação. Além da recorrência em si de qualquer TVS, o seguimento deve registrar a densidade de arritmias, a ocorrência de tempestade elétrica, internações hospitalares e morte cardíaca e não cardíaca. 

 As Tabelas 11.11 e 11.12 mostram as recomendações e níveis de evidência para indicação de ablação por cateter de TVS, assim como os métodos utilizados durante o procedimento em pacientes com CCDC. 

**Tabela 11.11:** – Indicações de ablação por cateter de TVS na CCDC

Sumário das recomendações	Grau de recomendação	Nível de evidência
TVS monomórfica incessante ou recorrente, refratária ao tratamento com fármacos antiarrítmicos	Forte	B
Paciente com CDI e TVS monomórfica recorrente deflagrando choques múltiplos, apesar de tratamento com drogas antiarrítmicas e reprogramação do CDI	Forte	B
Paciente com CDI e TVS monomórfica recorrente deflagrando choques múltiplos, quando o tratamento com fármacos antiarrítmicos é contraindicado ou mal tolerado, apesar da reprogramação do CDI	Forte	C
TVS monomórfica induzida ao EEF para esclarecimento de síncope	Ponderado	C
Paciente com CDI e primeiro episódio de TVS monomórfica espontânea documentada em ECG ou interrompida pelo CDI	Ponderado	C
TVS monomórfica, recorrente, sem uso prévio de amiodarona	Ponderado	C
TVS monomórfica, mal tolerada, induzida em EEF para esclarecimento de síncope	Ponderado	C
A estratégia de ablação endocárdica/epicárdica combinada é preferível à estratégia endocárdica isolada para prevenir recorrência da TV	Forte	B
Ablação está contraindicada em casos de TVS polimórfica ou FV secundárias a distúrbios metabólicos graves ou a efeito pró-arrítmico de fármacos	Forte	C

CCDC: cardiomiopatia crônica da doença de Chagas; CDI: cardioversor-desfibrilador implantável; ECG: eletrocardiograma; EEF: estudo eletrofisiológico; FV: fibrilação ventricular; TV: taquicardia ventricular; TVS: taquicardia ventricular sustentada.

**Tabela 11.12:** – Métodos utilizados na ablação por cateter de TVS na CCDC

Sumário das recomendações	Grau de recomendação	Nível de evidência
Registro da TVS em ECG de 12 derivações antes da ablação, sempre que a condição hemodinâmica permitir	Forte	C
Avaliação clínica, laboratorial e por meio de ECG, Rx de tórax e ECO transtorácico (transesofágico nos pacientes em FA)	Forte	C
RMC com gadolínio e realce tardio antes da ablação	Ponderado	C
Angio TC das coronárias e TC do tórax e abdome superior (identificação de megacólon)	Ponderado	C
Reservar sala cirúrgica, banco de sangue e recuperação em unidade intensiva quando a abordagem epicárdica for considerada	Forte	B
Cineangiocoronariografia na impossibilidade de realização de angio TC das coronárias	Ponderado	C
Monitorização hemodinâmica invasiva durante a ablação da TVS	Forte	C
Reprogramar o CDI antes e no final do procedimento	Forte	B
Anestesia geral	Ponderado	C
Anticoagulação sistêmica após acesso às câmaras esquerdas, mantendo TCA acima de 350s durante a ablação endocárdica do VE	Forte	B
Avaliação eletrofisiológica com estimulação ventricular programada antes e depois da ablação	Ponderado	B
Acesso e mapeamento epicárdico em caso de insucesso da ablação endocárdica	Forte	B
Acesso e mapeamento epicárdico após recorrência de ablação endocárdica	Ponderado	B
Mapeamento e ablação endocárdica e epicárdica no primeiro procedimento	Ponderado	B
Mapeamento e ablação epicárdica exclusiva no primeiro procedimento ou após insucesso de ablação endocárdica	Ponderado	C
Limitar o procedimento para o máximo de 6h, exceto se o paciente persistir em TV incessante	Ponderado	B
Avaliação clínica, laboratorial e por meio de ECG e ECO 24h após o procedimento	Forte	B

CCDC: cardiomiopatia crônica da doença de Chagas; ECG: eletrocardiograma; ECO: ecocardiograma bidimensional; FA: fibrilação atrial; RNM: ressonância nuclear magnética; TC: tomografia; TCA: tempo de coagulação ativado; TV: taquicardia ventricular; TVS: taquicardia ventricular sustentada; VE: ventrículo esquerdo.

## 12. Condutas para Prevenção e Tratamento de Complicações Tromboembólicas 

### 12.1. Introdução

 As complicações tromboembólicas representam grupo heterogêneo de manifestações clínicas associadas à CCDC, correspondendo a um dos três mecanismos essenciais de morte dessa cardiopatia ao lado de IC e morte súbita. ^1^ Na maioria dos casos, a mortalidade pelos fenômenos tromboembólicos está relacionada a embolias encefálicas e pulmonares. Considerando que os eventos neurológicos são as manifestações clínicas usualmente mais expressivas, as complicações tromboembólicas encefálicas são detectadas de forma mais frequente na prática médica. ^830^

 O AVC, do tipo cardioembólico, pode ser a primeira manifestação clínica da CCDC e ocorrer mesmo em estágios precoces da história natural da doença, acometendo indivíduos de diversas faixas etárias e apresentando-se com recorrência frequente quando a profilaxia secundária não é estabelecida. As manifestações clínicas são usualmente decorrentes da embolização de trombos cardíacos intracavitários que, por sua maior dimensão, apresentam elevado potencial de obstrução da circulação proximal no sistema nervoso central, sendo geralmente associados a graves e incapacitantes sequelas neurológicas, quando não levam diretamente a morte. ^1^

### 12.2. Epidemiologia dos Eventos Tromboembólicos

 Estudos necroscópicos revelam frequência variável de trombose cardíaca na DC, com prevalência entre 27% e 79%, com leve predomínio de acometimento de câmaras direitas (22% a 54%, sendo 21% a 46% de câmaras esquerdas). ^831 - 834^ Nesses estudos, os fenômenos tromboembólicos foram mais comuns na circulação sistêmica, embora tenham causado relativamente mais mortes por embolia pulmonar. ^831^ A incidência de trombos cardíacos foi maior na síndrome clínica de IC (36%) do que em casos de morte súbita (15%), sem relação com idade ou sexo. 

 Lesões inflamatórias do endocárdio e estase sanguínea intracavitária são considerados fatores importantes na patogênese da trombose parietal cardíaca, relacionados à ocorrência de múltiplos fenômenos tromboembólicos e elevado risco de morte por embolia. ^831^ O aneurisma apical também é um fator relevante, estando presente em 53,2% dos casos em série de 148 autópsias, dos quais 36,8% seriam complicados por trombose localizada, enquanto apenas 11,1% dos corações sem aneurisma apical apresentavam trombos intracavitários. ^835^ Outro estudo, envolvendo 1.153 autópsias, constatou presença de aneurisma apical em 52% dos casos, ^836^ com predomínio no sexo masculino. 

 Em estudo observacional prospectivo de 55 pacientes com CCDC e aneurisma apical avaliados por ventriculografia de contraste radiológico, Albanesi Filho *et al* . ^837^ descreveram a presença de trombos intraventriculares em apenas 14,5% dos casos estudados. A baixa frequência de trombos descrita nesse estudo pode ser atribuída à menor sensibilidade do método de avaliação em relação aos estudos de necrópsia, que, além disso, provavelmente foram realizados em fase mais avançada da doença. 

### 12.3. Fatores de Risco e Mortalidade

 A presença de disfunção miocárdica grave, lesão apical do VE, trombos intracavitários e fenômenos tromboembólicos prévios, assim como dilatação das câmaras cardíacas e vigência de IC, tem sido associada a maior risco de acidentes tromboembólicos em estudos anatomopatológicos e clínicos. ^838^ Discinesias ventriculares regionais, em especial apicais, são condições características da CCDC, com maior prevalência em relação a outras etiologias, predispondo, assim, à formação de trombos murais e eventos embólicos, especialmente os sistêmicos. ^830^ Como ocorre em outras cardiopatias, dilatação cardíaca e IC constituem fatores de risco reconhecidos para ocorrência de eventos tromboembólicos. A FA, manifestação considerada relativamente tardia e em geral associada à disfunção ventricular, constitui fator trombogênico adicional nessa cardiopatia. ^830^

 A mortalidade associada aos eventos tromboembólicos na CCDC está em geral relacionada a embolias encefálicas e pulmonares, com mais de um território arterial comumente afetado. ^839^ Em relação à embolia pulmonar, a maior parte dos eventos origina-se nas próprias cavidades cardíacas direitas, diferindo das demais cardiopatias, nas quais os trombos comumente provêm dos membros inferiores. ^839^ Admite-se que fenômenos embólicos pulmonares sejam clinicamente subestimados na CCDC, a se considerar sua elevada prevalência em material de necropsias, ^830^ o mesmo ocorrendo com as embolizações sistêmicas não encefálicas. Tromboembolismo pulmonar pode acometer até 37% dos pacientes com IC, mas poucas vezes é relatado em pacientes sem essa síndrome. Em 85% dos casos, associa-se à trombose mural das câmaras cardíacas direitas. ^363^

 O tromboembolismo sistêmico afeta principalmente o cérebro, podendo constituir manifestação clínica inicial da DC, associando-se à presença de trombos murais e aneurisma da ponta do VE. Devido à sua maior expressão clínica, o AVC tem sido alvo de muitas investigações. Os primeiros registros de AVC embólico na CCDC foram feitos por Nussenzveig *et al* . em 1953. ^840^ Posteriormente, em 1955, Rocha & Andrade descreveram fenômenos tromboembólicos sistêmicos em pacientes com CCDC. ^841^

 A presença de CCDC é considerada fator independente de risco para ocorrência de AVC isquêmico. Estudos de casos-controles mostraram que IC, arritmias ao ECG, gênero feminino e aneurisma da ponta de VE constituem fatores de risco independentes para tromboembolismo cerebral em pacientes com DC. ^842 , 843^

 Em estudo utilizando ECO transtorácico e transesofágico, avaliando 75 pacientes, foram encontrados trombos murais de VE em 23% dos casos, em flagrante associação com história pregressa de AVC. Aneurisma apical foi identificado em 47% dos pacientes, significativamente relacionado à trombose mural e ocorrência de AVC. Trombose do apêndice atrial esquerdo foi constatada em 4 pacientes e trombose do apêndice atrial direito em 1 paciente. Houve 13 mortes em 24 meses de seguimento, sendo 7 subitamente, 5 por progressão de IC e 1 por AVC. Diferentemente de outras cardiopatias, na CCDC, o AVC ocorreu de forma mais frequente em pacientes com disfunção sistólica ventricular esquerda leve e classe I pela NYHA. ^363^

 Entretanto, em séries hospitalares, a incidência anual de fenômenos tromboembólicos em pacientes com CCDC e disfunção ventricular leve a moderada mostrou-se baixa (1% a 2%). ^363 , 358^ Em contraste, essa incidência revelou-se expressivamente maior, de 60% ao ano, nos pacientes com IC manifesta, nos quais, aneurisma da ponta do VE e trombose mural do VE foram observados em 23% e 37% dos casos, respectivamente. No conjunto de todas as séries descritas, a prevalência de trombose de câmaras direitas (53%) superou a de câmaras esquerdas (43%). ^831 - 834^

 Ao longo da pandemia de COVID-19, doença causada pelo SARS-CoV-2, constatou-se maior predisposição dos pacientes infectados às complicações trombóticas arteriais e venosas, devido a alterações inflamatórias, da microcirculação endotelial e estase sanguínea, dentre outros fatores. ^844^ Como a CCDC também cursa com um estado pró-inflamatório e pró-trombótico, a associação dessas duas doenças poderia atuar, de forma sinérgica, para potencializar o aparecimento de eventos tromboembólicos. 

 Entretanto, até o momento, não existe evidência clara dessa associação em relação a eventos clínicos mais relevantes. Todavia, ambientes intervencionistas relatam que síndromes coronarianas agudas em pacientes com COVID-19 tendem a se apresentar mais tardiamente após início dos sintomas e com maior gravidade clínica. ^845^ Em decorrência, há estudos em andamento testando terapêuticas antitrombóticas mais agressivas em tais contextos. A conduta frente à coexistência das duas infecções é abordada em subtópico específico desta diretriz. 

### 12.4. Avaliação de Risco de Acidente Vascular Cerebral 

 Como exposto, pacientes com formas mais avançadas da CCDC têm maior risco de desenvolver episódios tromboembólicos por apresentarem condições favoráveis à formação de trombos, como estase venosa e baixo fluxo sanguíneo, dilatação das câmaras cardíacas, disfunção sistólica do VE e fenômenos inflamatórios vasculares. Outros fatores, como alterações parietais por hipocontratilidade segmentar e arritmias, especialmente FA, contribuem para aumentar o risco de tromboembolismo. ^832^ Mesmo pacientes com CCDC sem disfunção ventricular global podem apresentar aumento significativo dos marcadores de risco de trombose, sugerindo estado pró-trombótico em fases mais precoces da doença. ^846^

 Revisão sistemática de oito estudos observacionais, abrangendo um total de 4.158 pacientes, permitiu evidenciar associação clara entre CCDC e risco de AVC. Esse estudo indicou que pacientes cronicamente infectados por *T. cruzi* , quando comparados aos não infectados, apresentavam excesso de risco de AVC, da ordem de 70% (RR = 1,70; IC 95%: 1,06-2,71). ^361^

 Em coorte prospectiva de 1.043 pacientes com DC (com e sem cardiopatia), seguidos por tempo médio de 5,5 anos, encontrou-se incidência de 3% de AVC cardioembólico, ou seja 0,56% ao ano. ^364^ Um escore de risco ( *IPEC-FIOCRUZ* ) para ocorrência de AVC baseado em pontos e a indicação de profilaxia de eventos embólicos foram propostos pelos autores, considerando-se a presença de disfunção sistólica do VE (em qualquer grau e localização - 2 pontos), aneurismas apicais (1 ponto), alterações primárias da repolarização ventricular ao ECG (1 ponto) e idade > 48 anos (1 ponto). Pacientes com 4-5 pontos foram considerados de alto risco para AVC cardioembólico. Para essa análise foram excluídos os fatores de risco clássicos associados a complicações cardioembólicas em outras cardiopatias, para os quais já estaria assegurada a indicação de profilaxia, como FA, trombos intracavitários e eventos cardioembólicos prévios. Ainda assim, a frequência de eventos foi comparativamente maior que em outras cardiopatias em análises pareadas para o mesmo grau de disfunção sistólica, demonstrando que a CCDC seja de fato uma entidade mais trombogênica. ^364^

 Estudos recentes sugerem que o *flutter* e a FA possam ser mais frequentes na CCDC do que inicialmente se descrevia, com aumento da prevalência dessas arritmias em estágios mais avançados da doença, acompanhando o agravamento da disfunção ventricular, e constituindo-se em fator trombogênico adicional. ^847^

 Entretanto, embora as complicações cardioembólicas sejam muito frequentes na CCDC, devendo ser sempre avaliadas como potencial fator causal para o AVC isquêmico, outros mecanismos, como eventos aterotrombóticos ou lacunares, e, mais raramente, etiologias diversas de vasculites e coagulopatias ^848^ podem ser implicados na gênese do AVC isquêmico em pacientes com DC. O recente aumento da expectativa de vida dessa população e mudanças em seu estilo de vida, com eventual incorporação dos fatores de risco cardiovasculares clássicos para aterosclerose (como HAS, dislipidemia e diabetes *mellitus* ), fazem com que o AVC isquêmico seja considerado uma das principais causas de morte em coortes históricas de pacientes com DC, ^849^ embora nem sempre o mecanismo cardioembólico seja implicado. 

 Eventualmente, mesmo a condição de risco cardioembólico pode não significar de fato manifestação da CCDC, mas estar associada à própria evolução da cardiopatia do idoso, também responsável por aumento da incidência de FA. Nem sempre é possível estabelecer o nexo causal preciso do AVC em idosos e pacientes com múltiplas comorbidades clínicas. Entretanto, o cuidado integral ao paciente deve ser considerado o tema mais relevante, estabelecendo-se tratamento e/ou profilaxia apropriados em cada situação. 

### 12.5. Quadro Clínico e Investigação Diagnóstica do Acidente Vascular Cerebral Isquêmico na Doença de Chagas 

 O AVC é definido como um déficit neurológico, em geral focal, de início súbito, com duração de pelo menos 24 horas, de causa presumivelmente vascular, eventualmente seguido de morte. A presença de sinais e sintomas neurológicos focais, que desaparecem em menos de 24 horas, caracteriza o ataque isquêmico transitório (AIT). O diagnóstico de AVC é baseado nas manifestações clínicas apresentadas pelo paciente, com a presença de pelo menos uma das seguintes alterações neurológicas: déficit motor ou sensitivo, afasia ou disfasia, hemianopsia, desvio conjugado do olhar, ou início súbito de apraxia, ataxia ou déficit de percepção. ^850^

 Em pacientes com DC, devido ao predomínio de AVC isquêmico de etiologia cardioembólica, sintomas de manifestação cortical são frequentemente observados e as síndromes da circulação anterior são as mais comuns, relacionadas ao território de irrigação das artérias cerebrais médias e anteriores. ^843^ Essas caracterizam-se por sinais de disfunção cortical superior (alterações da linguagem, da função visuoespacial ou do nível de consciência), hemianopsia homônima (déficit visual que acomete igualmente ambos os olhos) e déficit motor e/ou alteração sensitiva de pelo menos duas áreas do corpo (face, membros superiores e membros inferiores). Lesões corticais extensas geralmente ocasionam todos esses distúrbios neurológicos, caracterizando assim a síndrome da circulação anterior total. Lesões corticais menos extensas podem levar à síndrome da circulação anterior parcial, com presença de dois desses três conjuntos de manifestações neurológicas. 

 Já as síndromes de circulação posterior são menos frequentes, afetando áreas de irrigação da artéria cerebral posterior, como cerebelo e tronco cerebral. Essas manifestam-se com pelo menos uma das seguintes alterações: paralisia de nervos cranianos associada a déficit sensitivo-motor contralateral, déficit sensitivo-motor bilateral, alterações dos movimentos conjugados dos olhos, disfunção cerebelar sem déficit de trato longo ipsilateral, hemianopsia isolada ou cegueira cortical. ^851^

 Os sinais e sintomas de AVC isquêmico secundário à aterosclerose de grandes artérias podem ser semelhantes e, portanto, indistinguíveis daqueles presentes nos eventos cardioembólicos quanto ao comprometimento motor ou sensitivo, porém sem alterações das funções corticais, como linguagem ou funções cognitivas. 

 Alguns pacientes podem demonstrar síndromes lacunares, geralmente relacionadas à presença de outros fatores de risco cardiovascular em concomitância com a DC, como HAS e diabetes *mellitus.* Os infartos cerebrais lacunares caracterizam-se pela presença de déficits motores e sensitivos puros, que podem ocorrer de forma isolada ou combinada, ou pela hemiparesia atáxica. 

 O AVC isquêmico silencioso pode ocorrer em proporção significativa de pacientes com CCDC, tendo sido relatado em 18% dos indivíduos incluídos em estudo do tipo caso-controle posteriormente identificados com DC. ^852^

 A realização de tomografia computadorizada ou ressonância magnética (RM) de encéfalo é recomendada para a confirmação diagnóstica de lesão estrutural decorrente de eventos vasculares, classificação do tipo de evento e exclusão de diagnósticos diferenciais. ^853^ Na avaliação do AVC agudo, a tomografia computadorizada é considerada a estratégia mais custo-efetiva, por se tratar de método de rápida execução e ampla disponibilidade na maioria dos serviços de emergência médica. A RM é particularmente útil para a avaliação de lesões da circulação posterior, de pequenos infartos corticais, infartos lacunares e, sobretudo, para a análise de imagens não usuais quando há dúvidas sobre o diagnóstico de AVC. ^854^

 Em pacientes com DC e diagnóstico de AVC, os exames de imagem mostram predomínio de lesões cerebrais em topografia de irrigação das artérias cerebrais médias, que são as áreas geralmente mais acometidas nos indivíduos com AVC isquêmico. Pacientes com DC e AVC isquêmico de etiologia indeterminada apresentam proporção elevada de acometimento estrutural em território de irrigação dos ramos inferiores da artéria cerebral, que podem estar associados a êmbolos cardíacos, possivelmente devidos a fatores anatômicos e hemodinâmicos. ^855^

 Os sinais e sintomas neurológicos decorrentes do AVC podem ser as primeiras manifestações clínicas de pacientes com DC. ^856^ Dessa forma, a pesquisa para infecção pelo *T. cruzi* mediante testes sorológicos deve ser considerada nos casos de AVC isquêmico em pacientes provenientes de áreas endêmicas para a DC ou filhos de mães com a mesma condição de risco endêmico, especialmente em casos de eventos cerebrovasculares secundários a tromboembolismo ou de etiologia indeterminada. ^857^

 Reconhecidamente, a maioria dos pacientes com DC que desenvolvem AVC apresenta primariamente sinais de cardiomiopatia. ^361^ Arritmias cardíacas, sobretudo *flutter* e FA, disfunção sistólica do VE, dilatação atrial esquerda, aneurisma apical e trombose intracavitária são fatores associados à ocorrência de AVC em pacientes com a CCDC. ^358 , 360 , 364 , 849^ Dessa forma, ECG e ECO transtorácico de repouso são os exames complementares recomendados para a investigação desses fatores de risco. Em pacientes com janela ecocardiográfica imprópria para a adequada avaliação do ápice ventricular esquerdo, a ecocardiografia com contraste de microbolhas e a RMC podem ser úteis para a identificação de aneurismas e trombos murais nessa região. ^858^

 Nos casos de AVC isquêmico embólico de fonte trombogênica indeterminada após avaliação inicial, pode-se considerar a complementação diagnóstica com o monitoramento eletrocardiográfico contínuo (Holter) de 24 horas e com a ecocardiografia transesofágica. Se o paciente é portador de MP ou CDI, pode-se interrogar o registro de eventos do dispositivo, com o mesmo intuito de se identificar arritmias com potencial emboligênico. 

 A concomitante prevalência de outros fatores de risco para doenças cardiovasculares, como HAS, diabetes *mellitus* , dislipidemia e tabagismo, em pacientes com DC pode ser elevada, principalmente naqueles que apresentam AVC isquêmico. ^856^ Nesses casos, a investigação não invasiva de doença ateromatosa carotídea e das artérias vertebrais por meio de exame ultrassom-Doppler, angiotomografia ou angiorressonância é recomendada, especialmente para os pacientes que demonstram infarto cerebral relacionado à circulação cerebral anterior. Doppler transcraniano também pode ser útil nesses casos. 

 Diagnóstico diferencial com outras condições clínicas raras como vasculites ou trombofilias deve ser buscado nos casos de suspeita clínica ou quando o diagnóstico permanece indeterminado por meio da realização de coagulograma, com avaliação do tempo de protrombina (relação normatizada internacional - RNI) e contagem de plaquetas ou pesquisa específica de outras etiologias mais incomuns. 

 Na Tabela 12.1 , encontram-se resumidas as recomendações para investigação de AVC isquêmico na DC. 

**Tabela 12.1:** – Investigação de AVC isquêmico na CCDC

Indicação	Exame	Grau de recomendação	Nível de evidência
Confirmação diagnóstica de evento neurológico agudo, classificação do tipo de evento e avaliação de diagnósticos diferenciais ou complicações	TC cerebral	Forte	B
	RNM cerebral (particularmente para avaliação de pequenos infartos ou acometimento da circulação posterior)	Ponderado	B
Pesquisa de infecção por *T. cruzi* em casos de AVC isquêmico e risco epidemiológico identificado	Sorologia para doença de Chagas (duas técnicas sorológicas distintas)	Forte	C
Pesquisa de acometimento cardíaco e fonte emboligênica associada à CCDC	ECG e ECO transtorácico	Forte	B
	RNM cardíaca ou ECO com microbolhas (se janela imprópria para avaliação apical de VE)	Ponderado	B
	Holter de 24h (fonte trombogênica indeterminada após avaliação inicial)	Forte	B
	ETE (fonte trombogênica indeterminada após avaliação inicial)	Ponderado	C
Diagnóstico diferencial com etiologia aterotrombótica em pacientes com fatores de risco cardiovascular	Doppler arterial de carótidas e vertebrais	Forte	B
	AngioTC ou AngioRNM cerebral	Ponderado	B
	Doppler transcraniano	Ponderado	C
Diagnóstico diferencial com outras etiologias	Coagulograma com tempo de protrombina (RNI) e contagem de plaquetas	Forte	B

AVC: acidente vascular cerebral; CCDC: cardiopatia crônica da doença de Chagas; ECG: eletrocardiograma; ECO: ecocardiograma; ETE: ecocardiograma transesofágico; RNM: ressonância nuclear magnética; RNI: relação normatizada internacional; TC: tomografia computadorizada; VE: ventrículo esquerdo.

### 12.6. Tratamento do Acidente Vascular Cerebral Isquêmico na Doença de Chagas 

 As condutas terapêuticas dependem do tempo de início dos sintomas, da vigência de comorbidades e da gravidade e extensão da área acometida pela isquemia cerebral. Essa última pode se apresentar como AIT, infartos cerebrais silenciosos ou AVC isquêmicos, com sequelas motoras leves ou graves e transformação hemorrágica, causando morte, comprometimento crônico da cognição ou drástica limitação física. ^859 - 861^

 A abordagem terapêutica inicial na CCDC é semelhante à de outras etiologias e objetiva estabilizar, reduzir danos e prevenir complicações por meio de internamento em terapia intensiva específica para pacientes com AVC, onde as seguintes medidas gerais devem ser observadas: ^862^ 1) Controle de funções vitais e temperatura; 2) Manejo de HAS, mas evitando causar hipotensão e consequente agravamento da isquemia cerebral; 3) Controle de hiper ou hipoglicemia; 4) Hidratação cuidadosa e controle do nível de sódio sérico; 5) Proteção de vias aéreas e de deglutição, evitando infecção por broncoaspiração; 6) Identificação precoce de hipoventilação, evitando retenção de CO _2_ e hipoxemia, por meio da suplementação de oxigênio; 7) Prevenção de trombose venosa profunda, utilizando heparinas ou seus sucedâneos orais ou métodos mecânicos de compressão pneumática quando indicados; e 8) Determinação da extensão da lesão cerebral por tomografia computadorizada ou RM do crânio para tratamento do edema cerebral e identificação do risco ou presença de transformação hemorrágica, avaliando sintomas sugestivos como cefaleia intensa e persistente, sonolência, rebaixamento do nível de consciência, além de piora dos déficits motores/sensoriais. 

 Nos casos agudos e mais graves, estando em janela terapêutica e não havendo contraindicações, a trombólise deve ser instituída (tempo de apresentação < 4,5 horas), sendo habitualmente utilizado o rt-PA intravenoso. Se a tomografia computadorizada de crânio inicial sugerir hipodensidade precoce, igual ou maior do que um terço do território da artéria cerebral média, a trombólise é contraindicada, devido ao elevado risco de transformação hemorrágica. Em casos específicos, a trombectomia endovascular permite o tratamento com janela terapêutica maior, mas ainda inferior a 24 horas. ^862^

 Passada a fase aguda do evento isquêmico, a anticoagulação oral com varfarina é a terapêutica estabelecida para a profilaxia secundária de complicações tromboembólicas originadas do coração, seja na vigência de arritmias ou evidência de trombose intracavitária. ^1 , 2 , 722 , 862^ Ajustes frequentes da anticoagulação são necessários para manutenção da faixa terapêutica ideal (RNI entre 2 e 3) e o tratamento deve continuar por toda a vida. 

 Como opção mais simples, por não serem necessárias consultas recorrentes para ajustes da anticoagulação, os novos anticoagulantes orais de ação direta ou indireta (rivaroxabana, edoxabana, apixabana e dabigatrana) podem ser empiricamente usados em pacientes com arritmias atriais crônicas do tipo *flutter* ou FA, com resultados potencialmente benéficos, eventualmente até superiores em relação à varfarina. ^722^ Mais recentemente, meta-análises comparando os novos anticoagulantes à varfarina em indivíduos com trombose de VE associada à CMI ou CMD sugerem que esses fármacos teriam eficácia semelhante à do antagonista da vitamina K quanto à frequência de resolução do trombo, prevenção de AVC ou outros eventos tromboembólicos e complicações hemorrágicas. ^863^ Essa plausibilidade talvez se aplique a pacientes com a CCDC, mas ainda permanece por se demonstrar, e o custo do tratamento de longo prazo pode limitar seu uso em população com reconhecida vulnerabilidade e marginalização social. 

 O tempo para início da anticoagulação oral crônica após o AVC isquêmico é controverso, não tendo sido estudado de maneira sistemática. Aceita-se que, para pacientes com AIT, seja razoável o início de anticoagulantes 24 horas após o início dos sintomas; para pacientes com déficits leves, após 3 dias; para pacientes com déficits moderados, após 6 a 8 dias; e para pacientes com déficits graves, após 12 a 14 dias, desde que, em todas essas situações, se exclua transformação hemorrágica após avaliação por exame de neuroimageamento. ^862^

 Escores de risco, como CHADS2 ou CHA2DS2-VASc, são empregados para orientação de profilaxia primária e secundária de AVC cardioembólico na vigência de FA e de outros fatores de risco cardiovasculares, ^2 , 722 , 862^ independentemente da etiologia da cardiopatia. A princípio, todos os pacientes com 2 pontos ou mais (talvez também homens com 1 ponto ou mais) se beneficiariam da profilaxia com anticoagulantes; entretanto, o risco de complicações hemorrágicas deve ser sempre avaliado durante o uso crônico desses medicamentos. 

 O escore HAS-BLED (baseado em hipertensão não controlada; função renal/hepática anormal; AVC prévio; história ou predisposição a sangramento, como anemia; RNI lábil; idade > 65 anos; uso concomitante de drogas/álcool) foi validado em diferentes coortes (não de indivíduos com DC) para definir o risco de complicações hemorrágicas, com alto risco de sangramento identificado por um escore ≥ 3. ^722 , 862^ Nesse contexto, a avaliação de risco *versus* benefício deve ser definida de modo individual e compartilhada com o paciente e seus familiares. 

### 12.7. Prevenção de Eventos Cardioembólicos na Doença de Chagas 

 A prevenção de eventos cardioembólicos no paciente com CCDC é de extrema importância pelo elevado impacto negativo potencial dessas complicações na morbimortalidade e qualidade de vida. ^2^ Durante o seguimento clínico, faz-se necessário rastrear, de forma periódica, as potenciais condições de risco para eventos cardioembólicos, tais como: disfunção sistólica ventricular e IC, presença de aneurismas ventriculares ou trombos murais e arritmias (especialmente a FA). ^1 , 364^

 Recomenda-se que todos os pacientes com DC sejam submetidos a ECG e ECO com periodicidade recorrente em seu seguimento ambulatorial e na avaliação clínica de um evento cardioembólico agudo ou prévio. ^1 , 2^ O ECG, idealmente deve ser realizado com um traçado longo de pelo menos 30s, permitindo a identificação de arritmias atriais. ^1^ O ECO permite a visibilização das cavidades cardíacas, identificando graus variados de disfunção sistólica ventricular esquerda, áreas regionais de discinesia, aneurismas (principalmente apicais), contraste espontâneo e trombos murais, caracterizando-os como móveis ou sésseis, com elevado potencial de embolização, ou organizados. ^858^

 A busca ativa de arritmias com alto potencial emboligênico, como *flutter* ou FA, também deve ser feita de forma seriada no seguimento clínico de pacientes com DC por meio da realização de ECG anual, estratégia que também permite a identificação da progressão da forma crônica indeterminada para a de cardiopatia. ^2^

 Além disso, a anamnese e o exame físico são primordiais, avaliando sintomas como palpitação, taquicardia, dor precordial, tontura, lipotimia, mal-estar, fraqueza, dispneia, piora da classe funcional, dentre outros, que levam à suspeita clínica de uma arritmia. No exame físico, o mais notório é a identificação de pulso ou ritmo cardíaco irregular à ausculta. Como os eventos arrítmicos podem ocorrer de forma paroxística, a arritmia pode não ser identificada no momento da avaliação clínica. Nos casos de suspeição clínica persistente, faz-se necessário realizar o monitoramento eletrocardiográfico contínuo utilizando o Holter de 24 horas. ^1 , 2 , 722^

 Em pacientes com dispositivos implantados como MP, CDI ou ressincronizador, eventualmente a irregularidade do ritmo deixa de ser percebida ao exame físico, sendo necessária a avaliação do ECG ou, mais apropriadamente, recorrer-se ao próprio registro de eventos, presente nesses aparelhos. ^2 , 722^ Recomenda-se interrogar os dispositivos intracardíacos, de forma sistemática, em cada avaliação prevista, buscando o registro de episódios silenciosos de FA. Uma adequada interface de atuação entre as equipes de seguimento clínico (cardiologia clínica e arritmologia) faz-se necessária para que intervenções apropriadas sejam recomendadas, como o início de anticoagulação para prevenção primária. 

 De acordo com diretrizes recentes de IC e arritmologia, considerando cardiopatias de diversas etiologias, a constatação de trombose mural, fenômenos tromboembólicos prévios e FA com CHA2DS2-VASc ≥ 2 já indicaria a anticoagulação como estratégia de profilaxia para eventos cardioembólicos. ^722 , 863^ É plausível admitir que as mesmas recomendações seriam aplicáveis empiricamente, por extrapolação, a pacientes com CCDC. 

 Como assinalado acima, reconhecendo o maior potencial emboligênico da CCDC, desenvolveu-se escore de risco específico de AVC cardioembólico para essa etiologia, ampliando as recomendações classicamente estabelecidas para outras cardiopatias. ^364^ Por meio da análise de risco-benefício, os investigadores proponentes desse escore *IPEC-FIOCRUZ* também sugeriram que, para indivíduos com a máxima pontuação (4-5 pontos), a incidência de AVC de 4,4% ao ano superaria a taxa estimada de 2,0% ao ano de sangramento grave associada ao uso de varfarina. ^364^

 Embora diretrizes anteriores tenham referendado o uso desse escore, ^1 , 2^ torna-se hoje imperativo que ele seja revisitado, para ser aplicado especificamente a pacientes com CCDC (isso é, não se englobando o subgrupo com FIDC, que praticamente não apresenta risco de AVC), com eventual correção dos pontos atribuíveis às variáveis (por exemplo, para a variável independente disfunção sistólica, com coeficiente beta de regressão de 2,6, foram atribuídos 2 pontos, quando o correto seria um arredondamento para 3 pontos), definição mais adequada de faixas etárias e, sobretudo, para ser respaldado por validação externa. 

 Essa questão da validade externa de escores de risco assume especial relevância para serem recomendados em aplicações práticas, no contexto geral da CCDC, à luz dos conceitos atuais. ^473 , 864 , 865^ Com implementação desses princípios metodológicos, o escore poderá ser revigorado e, coerente com seu inegável e histórico papel científico no contexto, recuperar mais abrangência e aplicabilidade do que atualmente se verifica. ^474 , 866^ Ademais, as empíricas condutas terapêuticas sugeridas quando de sua formulação ^364^ idealmente deverão ser lastreadas em estudos aleatorizados de comprovação de eficácia. ^474 , 866 , 867^

 Considerando que recentemente se observa nítida tendência à maior sobrevida de pacientes com DC e, consequentemente, fatores de risco cardiovascular ocorram de forma mais frequente nessa população, amplia-se a prevalência de FA (não necessariamente relacionada à própria CCDC, mas eventualmente associada à cardiopatia do idoso ou outras comorbidades clínicas), com consequente risco adicional de AVC cardioembólico. Dessa forma, recomendações de mudanças no estilo de vida com controle da HAS, diabetes *mellitus* , dislipidemia, cessação do tabagismo, perda de peso e atividade física regular ^722^ também são importantes para redução de eventos cardioembólicos nessa população. As recomendações de tratamento e prevenção de AVC cardioembólico na CCDC estão resumidas na Tabela 12.2 . 

**Tabela 12.2:** – Tratamento e prevenção de eventos cardioembólicos na CCDC

Indicação	Conduta	Grau de recomendação	Nível de evidência
Tratamento (∆t ≤ 4,5h) excluídas contraindicações e risco potencial de transformação hemorrágica	Trombólise IV (rt-PA)	Forte	B
Anticoagulação oral (prevenção primária ou secundária)	FA com CHADS-VASc ≥ 2 (mulher) e ≥ 1 (homem)	Forte	B
	Trombo mural	Forte	C
	AVC isquêmico prévio	Forte	C
Pesquisa de acometimento cardíaco e fonte emboligênica associada à CCDC	ECO transtorácico	Forte	C
	Holter de 24h	Forte	C
	Interrogar Holter de eventos (portadores de MP/CDI)	Forte	C
Redução do risco de FA (independentemente da predisposição intrínseca à CCDC)	Mudança de estilo de vida (controle HAS, DM, dislipidemia, perda de peso, atividade física, cessação do tabagismo)	Forte	C

CCDC: cardiopatia crônica da doença de Chagas; CDI: cardioversor-desfibrilador implantável; DM: diabetes mellitus; HAS: hipertensão arterial sistêmica; ECO: ecocardiograma; FA: fibrilação atrial; IV: intravenosa; MP: marca-passo; rt-PA: ativador do plasminogênio tissular recombinante; ∆t: tempo desde início dos sintomas.

## 13. Condutas em Subgrupos Especiais e Abordagem de Problemas Relativos a Gravidez, Atividade Física, Risco Cirúrgico, Anestesia Geral e Covid-19 

### 13.1. Coinfecção *T. cruzi* -HIV 

 Com estimativa de 37 milhões de pessoas vivendo com HIV/AIDS no mundo todo, o risco da coinfecção *T. cruzi-* HIV ^868^ é uma realidade em áreas endêmicas e não endêmicas que albergam imigrantes infectados pelo parasito. ^869 , 870^

 A coinfecção *T. cruzi-* HIV foi registrada inicialmente em 1990 ^871^ como RDC, tendo sido citada em 1988 no Brasil pela identificação do parasito no líquor de paciente com AIDS. ^83^ Descrita principalmente no Brasil e na Argentina, mas também em outros países (Bolívia, Chile, Espanha, EUA, Colômbia, Venezuela, Jamaica, Alemanha e Suíça), a RDC caracteriza-se por elevadas morbimortalidade e transmissibilidade materno-fetal, ^83 , 84 , 580 , 872 , 873^ interferindo na evolução tanto da DC como da infecção por HIV. Em geral, acomete pacientes infectados por HIV com grave deficiência imunológica (células CD4 ^+^ < 200/mm ^3^ ) e carga viral detectável por falta de resposta à terapêutica antirretroviral efetiva. Na infecção ativa por HIV, a acentuada redução de células CD4 ^+^ expressa a deficiência de resposta TH1, ^874^ responsável pela ativação de CD4 ^+^ e de macrófagos capazes de secretar IFN-γ e destruir os parasitos, assim ocorrendo aumento de parasitemia e parasitismo tecidual. ^875^

 A RDC apresenta-se como meningoencefalite em cerca de 2/3 dos casos, seguindo-se miocardite, meningoencefalite mais miocardite, pericardite, duodenite, gastrite, eritema nodoso e colpite. ^83 , 580 , 872 , 873^ Na forma congênita de coinfecção *T. cruzi-* HIV, ^872 , 873^ ocorrem abortos, baixo peso ao nascer, sepse e meningoencefalite. Mais raramente, formas oligossintomáticas manifestam-se como quadros febris, eritema nodoso, mielite e puérpera assintomática, mas com natimorto por DC congênita. ^872^

 A meningoencefalite causada por *T. cruzi* deve ser diferenciada de toxoplasmose e de doenças infecciosas, tumorais e degenerativas. A miocardite aguda na RDC deve ser diferenciada da CCDC descompensada. Níveis de CD4+ ≤ 200/mm ^3^ são observados em cerca de 2/3 dos casos, sendo menores na RDC do que em pacientes sem reativação. A mortalidade na RDC foi de 63 pacientes em 120 casos (52,5%). ^83^ RDC é descrita em 10%-15% dos casos de coinfecção em estudos retrospectivos e em 10% em estudos prospectivos de pacientes em acompanhamento prévio. ^83 , 580 , 873^

 A prevalência da coinfecção tem sido estimada em 1,5%-5,0% no Brasil ^83 , 580^ e 4,2% na Argentina, ^876^ sendo mais elevada em usuários de drogas ilícitas. ^877^ Estimam-se cerca de 4.570-15.360 casos de coinfecção com base no número de pacientes com infecção por *T. cruzi* e HIV no Brasil e Argentina, sugerindo-se um número muito subestimado na literatura em geral. 

 Entre as causas de mortalidade na coinfecção, ^878^ AIDS foi a causa básica de morte em 2/3 dos casos e DC em 17,5%. A FIDC predomina em cerca de metade dos casos de coinfecção, a forma cardíaca ocorre em 37%, seguindo-se as formas digestiva e cardiodigestiva em 5% e 6%, respectivamente. ^873^ Têm-se associado níveis reduzidos de CD4 ^+^ (no diagnóstico da coinfecção) ao prognóstico da reativação e mortalidade por reativação. A presença de parasitemia também tem sido associada à resposta TH2, sugerindo desequilíbrio a favor do parasito. 

 Dessa forma, recomenda-se que casos de infecção por HIV ou de DC sejam investigados ativamente do ponto de vista clínico e epidemiológico com indicação de triagem sorológica, visando ao diagnóstico precoce e controle de ambas as infecções. 

 O diagnóstico da coinfecção é realizado mediante positividade em duas provas sorológicas para ambas as infecções e/ou provas parasitológicas para o diagnóstico da DC. ^580 , 873^ Na DC, em caso de provas discordantes (ELISA, IFI ou HAI), uma prova confirmatória (imunoblot/imunocromatográfica) ou imunoenzimática com antígeno recombinante ou imunofluorescência é indicada. Para a infecção por HIV, ELISA ou CLIA positiva para antígenos HIV1 e HIV2 deve ser confirmada por prova imunoblot/imunocromatográfica para antígenos HIV1 e HIV2. ^580 , 873^ Provas parasitológicas de *T. cruzi* indiretas e PCR são específicas, mas com sensibilidades baixas para diagnóstico (cerca de 50% na forma crônica), embora mais elevadas na coinfecção. ^879 - 881^

 O diagnóstico da RDC deve ser efetivado por métodos padrão-ouro de pesquisa direta do parasito por microscopia no sangue e fluidos biológicos (líquor, líquido pericárdico) e/ou em tecidos corados. ^83 , 580^ Métodos de concentração (creme leucocitário, microhematócrito, Strout) são mais sensíveis do que o simples exame no esfregaço do sangue periférico ou a pesquisa do parasito a fresco em sangue periférico. A biópsia pode ser indicada quando outros métodos não invasivos falharem. ^83 , 580^ Em pacientes com RDC, as provas sorológicas para diagnóstico de DC podem ser negativas, ^876^ não invalidando o prosseguimento da investigação por métodos de microscopia direta. A PCR qualitativa e provas parasitológicas indiretas de enriquecimento, como hemocultura e xenodiagnóstico, têm baixo valor preditivo positivo para o diagnóstico da RDC, uma vez que podem ser positivas em pacientes crônicos sem reativação. ^879^ Por outro lado, provas semiquantitativas, como contagem de ninfas no xenodiagnóstico ^580^ e de qPCR, costumam ser úteis no monitoramento da RDC. ^880 , 881^

 O tratamento antiparasitário com benznidazol é obrigatório em pacientes com RDC, ^83 , 580 , 873^ na dose de 5mg/kg/dia por 60 dias. O derivado nitroimidazólico (nifurtimox) é indicado como segunda escolha quando o primeiro não estiver disponível ou houver evento adverso que impeça a sua continuidade. ^83 , 580^ Nas primeiras semanas pós-tratamento, a pesquisa direta do parasito por creme leucocitário ajuda a monitorar a falha terapêutica em casos de resultado positivo; resultados negativos não indicam sucesso terapêutico em curto tempo. O período de acompanhamento para o controle de cura deve ser realizado com PCR qualitativa ou exames parasitológicos indiretos (hemocultura) a partir de 3, 6, 9, 12, 24 meses e provas sorológicas aos 6, 12, 24 meses do início da terapêutica. 

 Em pacientes coinfectados sem RDC, tem-se mostrado melhor resposta antiparasitária quando ocorre parasitemia patente ou em níveis mais elevados inicialmente. ^580^

 Recomenda-se o seguimento em unidades de referência para controle tanto da carga viral, com controle da terapêutica antirretroviral efetiva para restaurar a resposta TH1, como da DC, com monitoramento da parasitemia, para evitar a RDC ou permitir seu diagnóstico e tratamento precoces ( Tabela 13.1 ). 

**Tabela 13.1:** – Recomendações para diagnóstico e tratamento de infecção por *T. cruzi* /HIV

Sumário das recomendações	Grau de recomendação	Nível de evidência
Busca ativa do diagnóstico da coinfecção mediante suspeita clínico-epidemiológica por exames sorológicos	Forte	B
Monitoramento de pacientes com coinfecção quanto à parasitemia por métodos moleculares e parasitológicos para evitar a reativação, de preferência a cada 3 meses	Forte	B
Uso de terapêutica antirretroviral efetiva para manter a resposta imune adequada	Forte	A
Diagnóstico da reativação por métodos de concentração mediante microscopia direta no sangue e fluidos biológicos ou biópsia	Forte	B
Uso de PCR quantitativo para diagnóstico da reativação após estabelecer os limites entre reativação e não reativação na região endêmica, a partir de diferenças entre número de cópias na reativação e não reativação em grande número de pacientes, mediante uso de iniciadores conhecidos e linhagens moleculares prevalentes	Ponderado	C
Não utilizar métodos parasitológicos indiretos ou métodos qualitativos positivos na fase crônica da doença de Chagas para diagnóstico da reativação	Ponderado	B
Tratamento da reativação com benznidazol imediatamente após o diagnóstico, com hospitalização em casos graves com comprometimento encefálico, miocárdico e/ou medular	Forte	C
Tratamento preemptivo na coinfecção em pacientes com parasitemia elevada por xenodiagnóstico (>20% de ninfas +) ou PCR quantitativo bem padronizado com valores mais elevados que a mediana dos casos na região	Ponderado	B
Manter tratamento antirretroviral eficaz para garantir resposta imune adequada, níveis de CD4+ >200 cel/mm ^3^ e carga viral indetectável para evitar a reativação	Ponderado	B
Profilaxia secundária 2x/semana com benznidazol em pacientes com CD4 ^+^ <200 cel/mm ^3^	Ponderado	C

 A indicação de profilaxia secundária com benznidazol (5mg/kg/dia 3x/semana) em pacientes com CD4 ^+^ < 200 células/mm ^3^ , em similaridade à prevenção de outras infecções oportunísticas, é controversa, não havendo estudos prospectivos ou séries retrospectivas confiáveis a respeito na DC. 

### 13.2. Soropositividade em Doadores Potenciais nos Bancos de Sangue 

 No Brasil, o rastreamento sorológico para DC é obrigatório para todos os doadores de sangue desde 1969. ^882^ Após o surgimento da AIDS na década de 1980, diversas medidas e legislações foram desenvolvidas e adotadas para aumentar o controle dos bancos e doadores de sangue, particularmente com a criação de hemocentros e a centralização das atividades de controle e vigilância sob a responsabilidade das Secretarias Estaduais de Saúde. ^883^

 A portaria do Ministério da Saúde nº 158 de 2016 estabelece como inapto para doação de sangue indivíduo com histórico de contato domiciliar com triatomíneos em áreas endêmicas e quem apresenta diagnóstico clínico ou laboratorial para DC. ^884^ Na triagem sorológica são utilizados testes sorológicos automatizados de alta sensibilidade e especificidade para detecção de anticorpos da classe IgG anti- *T. cruzi* , sendo os mais utilizados os testes de ELISA e, mais recentemente, a CLIA. ^885 , 886^ Na triagem sorológica dos bancos de sangue, apenas um teste sorológico se faz necessário, podendo ser repetido se a amostra apresentar resultado positivo. ^887^ Caso positivo, o sangue doado não poderá ser utilizado e o doador deverá ser contactado e encaminhado para esclarecimento diagnóstico em centros de referência em DC. 

 Com o controle da transmissão vetorial e transfusional, a prevalência média da DC entre doadores de sangue vem se reduzindo rapidamente. Projeções mais recentes estimam a prevalência para DC no Brasil em 0,18% dos potenciais doadores de sangue. ^2^ No entanto, essas taxas podem sofrer variações de acordo com as áreas onde são realizadas as doações e a idade dos doadores, sendo habitualmente maiores em regiões historicamente endêmicas e em faixas etárias mais altas. ^888^

 Pesquisas recentes desenvolvidas em doadores de sangue na região Nordeste apuraram prevalências de 0,17% a 0,57% no estado do Ceará e de 0,18% a 2,4% no estado do Piauí. ^889 , 890^ Estudo desenvolvido em Uberaba com grande número de doadores por período de 15 anos demonstrou queda da taxa de prevalência de 0,03% ao ano, observando-se, no último ano estudado, apenas 0,08% dos doadores inelegíveis por soropositividade. ^891^

 Com a queda da prevalência entre os doadores mais jovens, tem-se observado o aumento de casos inconclusivos ou indeterminados, sendo a maior parte resultante de testes falsos-positivos. ^885^ Todos os casos positivos devem ser encaminhados para centros de referência em DC para repetição e realização de novos testes para confirmação ou descarte do diagnóstico da doença. 

### 13.3. Atividade Física

 A prática de atividade física é uma importante estratégia de intervenção para a prevenção e tratamento de inúmeras doenças crônicas, principalmente aquelas relacionadas ao sistema cardiovascular. ^892^ Recentemente, a OMS publicou recomendações sobre a prática de atividade física em indivíduos saudáveis e com condições específicas de saúde e doença. Em geral, recomenda-se a realização de 150 minutos de atividades físicas de moderada intensidade e/ou 75 minutos de atividades físicas vigorosas por semana para obtenção de benefícios em termos de saúde cardiovascular. 

 Além disso, devem ser realizados exercícios de fortalecimento para os principais grupamentos musculares pelo menos duas vezes por semana, com intensidade moderada, avaliada por meio de escala de esforço percebido. Exercícios de flexibilidade e de equilíbrio devem também ser realizados, principalmente em indivíduos idosos, com intuito de manutenção da amplitude de movimento e autonomia para a realização das atividades da vida diária. 

 Benefícios de saúde podem ser obtidos mesmo para níveis de atividade física inferiores a essa recomendação, devendo a prática de atividade física ser iniciada de forma gradual em indivíduos previamente inativos. ^893^ Realizar pequenos volumes de atividade física traz mais benefícios à saúde em comparação com ser inativo, sendo que maiores volumes de atividade física podem trazer melhores benefícios por importante relação dose-resposta. ^893 , 894^

 Entretanto, os benefícios da atividade física na saúde física e mental em indivíduos com DC ainda não foram plenamente explorados. Alguns trabalhos apresentam resultados promissores para a melhora da capacidade funcional e da qualidade de vida. ^895 - 897^ Tais estudos, porém, incluíram apenas pacientes com a forma cardíaca da doença, não havendo trabalhos adequados na literatura que avaliem a influência dessa estratégia na FIDC. 

 Assim, em linhas gerais, as recomendações de exercícios para pessoas com FIDC devem ser idênticas às da população em geral, objetivando melhora dos parâmetros de aptidão física, controle de comorbidades e melhora da qualidade de vida. Intervenções no estilo de vida que aumentem gradualmente os níveis de atividade física devem ser estimulados, levando sempre em consideração a capacidade física e funcional de cada indivíduo para a realização das atividades propostas. Alguns trabalhos têm demonstrado que a prática de atividade física está associada à melhoria do trânsito intestinal, entretanto seus efeitos em indivíduos com a forma digestiva da DC ainda não foram investigados. ^898^

 Os efeitos da atividade física na CCDC foram objeto de trabalhos recentes, principalmente por meio de programas de reabilitação cardiovascular. ^895 - 897^ Em trabalho pioneiro sobre o assunto, ECR investigou os efeitos de programa de reabilitação cardiovascular em pacientes com CCDC acompanhados por 3 meses, tendo o treinamento físico promovido melhora da capacidade funcional e da qualidade de vida. ^895^

 Posteriormente, estudo de intervenção relatou que um programa de reabilitação cardiovascular em pacientes com IC por DC foi associado à melhora da função cardíaca avaliada pela FEVE, da força da musculatura respiratória e da qualidade de vida após 8 meses de acompanhamento. ^896 , 899^

 Mais recentemente, o ECR PEACH observou melhora da capacidade funcional e da microcirculação após 6 meses de programa de reabilitação cardiovascular em pacientes com CCDC com e sem IC. ^897 , 900^ Dessa forma, o exercício físico tem se mostrado como estratégia de intervenção bastante eficaz na melhora de diversos parâmetros clínicos e da qualidade de vida na CCDC ( Tabela 13.2 ). 

**Tabela 13.2:** – Recomendações para prática de atividade física em indivíduos com doença de Chagas

Sumário das recomendações	Grau de recomendação	Nível de evidência
Benefícios da atividade física em indivíduos com a forma indeterminada da doença de Chagas	Forte	C
Benefícios da atividade física em indivíduos com a forma cardíaca da doença de Chagas	Ponderado	B
Benefícios da atividade física em indivíduos com a forma digestiva da doença de Chagas	Ponderado	C

### 13.4. Gestantes

 A prevalência de infecção por *T. cruzi* entre mulheres grávidas varia de < 1% a 70,5% dependendo do país, da área geográfica e da localidade (rural ou urbana), enquanto a taxa de transmissão vertical em países endêmicos varia de 0% a 18,2%. ^38 , 39^ A taxa de transmissão vertical por *T. cruzi* apresenta diferenças regionais, variando em torno de 1,0% no Brasil e de 4% a 12% em outros países do Cone Sul, e parece depender de fatores ligados ao parasito e ao hospedeiro. ^40^

 A transmissão congênita da DC pode ocorrer durante qualquer fase da doença materna; entretanto, a maior taxa de transmissão ocorre em gestantes com a fase aguda da doença, aproximadamente 30%, enquanto a taxa geral é de 4,7%. ^88 , 901^ No Brasil, a frequência da transmissão congênita varia de 0% a 5,2%; entretanto, há grande heterogeneidade dependendo da região avaliada. A taxa mais alta de transmissão congênita regional foi observada na região Sul-Sudeste (2,1%), seguida pelas regiões Nordeste (1,6%) e Centro-Oeste (0,9%). ^87^

 As evidências de risco geral aumentado de aborto ou prematuridade em gestantes soropositivas são inconclusivas. No entanto, estudos sugerem que a infecção crônica materna não influencia o curso clínico da gravidez ou a saúde dos recém-nascidos, desde que não haja transmissão vertical. Porém, a infecção do feto aumenta a possibilidade de parto prematuro, baixo peso ao nascimento e natimortalidade. ^902^

 A transmissão congênita do *T. cruzi* é processo complexo, resultante da interação de múltiplos fatores relacionados ao parasita, à placenta e à resposta imune do feto e da mãe. ^903^ A carga parasitária de mulheres infectadas durante a gravidez é fator fundamental para a transmissão congênita. ^904^ A parasitemia pode reaparecer com a RDC geralmente associada à imunossupressão fisiológica transiente que ocorre durante a gravidez. ^905^ Adicionalmente, o papel da idade da mãe e do número de gestações no aumento do risco de transmissão ainda precisa ser melhor investigado. Por outro lado, evidências sugerem que a ativação da resposta imunológica inata em gestantes pode contribuir para a redução da ocorrência e gravidade da infecção congênita, mediante a regulação de mediadores pró e anti-inflamatórios. ^906^

 O impacto da DC no transcurso da gravidez é controverso. Alguns trabalhos apontam no sentido da benignidade dessa associação, enquanto outros relatam elevada incidência de complicações na gestação e de mortalidade perinatal, bem como hipotrofia neonatal, considerando as gestantes infectadas pelo *T. cruzi* como grupo de alto risco obstétrico. ^907^

 Gestantes com CCDC têm prognóstico estreitamente relacionado à gravidade da disfunção ventricular e classe funcional no início da gravidez. Pacientes que iniciam a gestação em classe funcional I e II geralmente chegam ao parto sem intercorrências; já aquelas em classe funcional III ou IV têm probabilidade de 25% a 50% de morte. ^908^ A cardiopatia, desde que assistida e sem maior gravidade, não contraindica a gravidez. Pacientes com IC e/ou arritmias graves devem ser desaconselhadas a engravidar, mas, caso engravidem, requerem acompanhamento e cuidados especiais. 

 O tratamento etiológico não deve ser instituído em gestantes nem em mulheres em idade fértil que não estejam em uso de contraceptivos. No entanto, já há evidências de que o tratamento etiológico reduz o risco de transmissão congênita numa gravidez subsequente. ^569 - 571^

 Além disso, no caso exclusivo de DC aguda, o tratamento etiológico pode ser instituído na gestante, levando-se em consideração a morbimortalidade materna, risco mais elevado de transmissão congênita e de impacto na saúde do recém-nato. As gestantes com DC aguda grave (miocardite ou meningoencefalite) devem ser tratadas independentemente da idade gestacional pela alta morbimortalidade materna, além do alto risco de transmissão congênita da DC (22% a 71%) e do potencial impacto na saúde dos neonatos. Gestantes com DC aguda não grave devem ser tratadas idealmente a partir do segundo trimestre de gestação, devido ao risco potencial de malformação congênita relacionado ao benznidazol. ^8^

 O uso de medicamentos com ação sobre o sistema cardiovascular pela gestante com DC deve seguir indicação médica seletiva e individualizada, devido ao risco potencial de efeitos colaterais sobre o feto. As mães infectadas deverão ser tratadas após o parto e o período de lactação para evitar a interrupção da lactação como resultado de possíveis reações adversas. A DC deve ser investigada sistematicamente em parentes e outras crianças nascidas de mães infectadas (diagnóstico sorológico) e os casos positivos devem ser avaliados clinicamente e tratados de acordo com os princípios já expostos. ^86^

### 13.5. Recém-natos

 Em áreas livres de vetores dentro e fora da América Latina, a transmissão vertical congênita ou perinatal é atualmente a principal forma de infecção pelo *T. cruzi* , superando aquelas por transfusão de sangue e transplante de órgãos. Apesar da subnotificação e subestimação evidentes globalmente, mais de dois milhões de mulheres em idade fértil já estão infectadas com *T. cruzi* e 1%-10% dos bebês de mães infectadas nascem com DC. Com base nas recentes demonstrações de que a transmissão congênita pode ser evitada, a OMS mudou seu objetivo em 2018, do controle para a eliminação da DC congênita. ^86 , 569 , 571^

 A gravidade da DC congênita varia amplamente, desde casos assintomáticos até infecção fatal, que está relacionada ao nível de parasitemia no nascimento. ^904^ Em trabalhos realizados no Brasil, Argentina, Chile e Paraguai foi demonstrado que 60% a 90% dos recém-natos com infecção congênita são assintomáticos. Nos sintomáticos, as alterações clínicas mais frequentes foram prematuridade, baixo peso, febre e hepatoesplenomegalia. ^86^

 O diagnóstico de infecção congênita deve ser pesquisado em todas as crianças nascidas de mães soropositivas, não apenas no primeiro mês de vida, mas também aos 6 e 12 meses de idade. O acompanhamento por 1 ano é essencial, pois proporção significativa de casos é inicialmente negativa e a doença só é detectada em um estágio posterior. ^86^

 Os métodos de diagnóstico mais recomendados no primeiro mês após o nascimento baseiam-se na pesquisa direta do *T. cruzi,* mediante a utilização de métodos de concentração por centrifugação, como a técnica do microhematócrito. ^908^ Quando positivos, esses testes oferecem um diagnóstico indiscutível e definitivo da infecção; contudo, nas situações em que a carga parasitária é baixa, principalmente quando a transmissão ocorre no último trimestre da gestação ou durante o parto, os exames podem gerar resultados negativos falsos. Dessa forma, testes mais sensíveis e automatizados são necessários para a detecção precoce da infecção congênita. O resultado positivo determina o início imediato do tratamento etiológico. ^86^ A DC congênita é considerada aguda e, portanto, de notificação obrigatória. 

 Em caso de exame parasitológico negativo, deve-se completar a investigação diagnóstica com testes sorológicos (com duas técnicas distintas), após o 7º mês de vida. Estudo sorológico antes do 6º mês não é útil, devido à passagem passiva de anticorpos maternos para a criança. Após o 10º mês, tais anticorpos desaparecem e o diagnóstico de DC congênita é mais preciso; entretanto, o atraso no diagnóstico diminui a eficácia do tratamento e aumenta o risco de perda de acompanhamento do bebê. ^86^ A sorologia negativa após o período acima referido permite a exclusão do diagnóstico de infecção pelo *T. cruzi* . 

 Os métodos moleculares representam alternativa promissora e têm sido amplamente utilizados para a detecção precoce das infecções congênitas, especialmente na Europa. Contudo, são métodos dispendiosos, exigem considerável treinamento técnico e necessitam de cuidadosa padronização, o que dificulta a sua implementação na rotina laboratorial. Como consequência, os métodos moleculares requerem validações clínicas mais amplas, antes de serem considerados padrão-ouro para diagnosticar infecções congênitas. ^86^

 O tratamento da infecção pelo *T. cruzi* no recém-nato é altamente eficaz e pode ser realizado com benznidazol (primeira opção no Brasil) ou nifurtimox, por 30 a 60 dias, com menos eventos adversos do que aqueles descritos em adultos, sendo a taxa de cura superior a 90%. As doses preconizadas para crianças são benznidazol 10mg/kg/dia em 3 ou 2 tomadas e nifurtimox 15 mg/kg/dia em 3 tomadas, sendo que o benznidazol tem apresentação de comprimidos de 12,5mg, que podem ser diluídos em água. O benznidazol é disponibilizado pelas secretarias estaduais de saúde e o nifurtimox deve ser solicitado à OPAS, via grupo técnico de DC da Secretaria de Vigilância Sanitária do Ministério da Saúde. 

 Ensaio clínico recentemente publicado utilizou nifurtimox para tratar crianças (0 a 17 anos de idade) na Argentina, Colômbia e Bolívia e comparou o tratamento de 30 dias contra 60 dias de duração. Ao final de 12 meses de seguimento, ambos os regimes demonstraram significativa soroconversão ou sororredução comparativamente a controles históricos, sendo o regime de 60 dias de duração superior ao de 30 dias de duração no grupo de 2-17 anos de idade. O nifurtimox foi bem tolerado, com efeitos adversos na maioria leves ou moderados e sem sequelas, sendo que apenas 4% desses eventos obrigaram à interrupção do tratamento. ^909^

 O acompanhamento do resultado da terapêutica etiológica deve ser realizado por testes parasitológicos e/ou moleculares nas semanas seguintes ao início do tratamento para neonatos com parasitemia. Após o término do tratamento, os pacientes devem ser acompanhados a cada 6 meses com testes sorológicos quantitativos. O paciente é considerado curado quando a sorologia se torna negativa em dois testes consecutivos. ^86^ O tempo necessário para ocorrer a negativação depende da idade e do início do tratamento. As crianças diagnosticadas nos primeiros meses de vida negativarão a sorologia entre o 2º e o 12º mês após o início do tratamento. Os sistemas de saúde devem avaliar e implementar estratégias que facilitem o diagnóstico mais precocemente possível da infecção congênita, considerando a frequente má adesão das mães aos atendimentos de acompanhamento nos centros de saúde. ^86^

 Considerando o risco de transmissão ao recém-nato ou lactente pelo contato com secreções maternas, recomenda-se suspender temporariamente a amamentação apenas nos casos de mães com RDC ou em fase aguda e, mais enfaticamente, aquelas que portem fissuras ou sangramentos mamilares. É importante a avaliação caso a caso considerando o grande benefício da amamentação nos primeiros meses de vida da criança. Conclusivamente, mães que já estejam utilizando o tratamento antiparasitário há pelo menos 30 dias, mesmo nos casos acima apontados, podem amamentar livremente. ^2^

### 13.6. Risco Cirúrgico e Anestesiológico

 Pacientes com CCDC têm riscos cirúrgico e anestésico aumentados por diversas razões, que devem ser consideradas nos períodos pré, intra e pós-operatório. 

 O cuidado pré-operatório mais importante é o controle da IC, com a otimização medicamentosa, e a correção de eventuais distúrbios hidroeletrolíticos. 

 A avaliação da função ventricular por meio do ECO deve ser feita sempre que possível. O ECG deve ser realizado em todos os candidatos a cirurgia e, em pacientes com arritmias ou sintomas compatíveis, a monitorização eletrocardiográfica dinâmica pelo sistema Holter pode ser necessária. Fármacos antiarrítmicos não devem ser suspensos, mas os anticoagulantes orais devem ser interrompidos. Os novos anticoagulantes orais inibidores diretos da trombina, como dabigatrana, ou os inibidores do fator Xa, como rivaroxabana, apixabana e edoxabana, podem simplesmente ser suspensos de 24 a 48 horas antes da cirurgia. No caso da varfarina, deve ser suspensa idealmente 5 dias antes da cirurgia, que poderá ser realizada quando o INR for inferior a 1,5. Durante o período de suspensão da varfarina, os pacientes com alto risco para eventos tromboembólicos devem receber anticoagulação com heparinas, por exemplo enoxaparina subcutânea em dose plena. Finalmente, a avaliação pré-operatória do paciente com DC deve considerar a eventual presença de megaesôfago, que acarreta risco aumentado de aspiração pelas vias aéreas durante os períodos intra- e pós-operatórios. ^910^

 Durante a cirurgia, o paciente com CCDC requer um manejo anestésico individualizado. O anestesista deve levar em consideração aspectos hemodinâmicos, como a disfunção miocárdica, muitas vezes biventricular, os quais limitam a infusão de volumes de líquidos e aumentam o risco de arritmias cardíacas. ^910^ A monitorização eletrocardiográfica contínua é essencial para controle de arritmias ventriculares malignas e bradiarritmias. A monitorização hemodinâmica invasiva arterial e venosa central é interessante e deverá ser implementada nos casos mais graves e em cirurgias de maior porte. O implante de MP cardíaco transvenoso temporário deve ser considerado nos pacientes com grau avançado de BAV, principalmente quando associado a distúrbios de condução intraventricular. O ECO transesofágico intraoperatório fornece valiosas informações sobre a resposta inotrópica à medicação anestésica e o estado volêmico do paciente, podendo ser muito útil em casos selecionados. 

 Os cuidados anestesiológicos são muito importantes. Nos pacientes com disfunção ventricular, a indução anestésica pode resultar em rápida deterioração hemodinâmica, que ocorre principalmente por vasoconstrição periférica e ação inotrópica negativa induzida pelos agentes anestésicos. ^910^ A infusão intraoperatória de volumes deve ser muito criteriosa. Pacientes em uso de vasodilatadores e diuréticos para o tratamento de IC comumente apresentam níveis pressóricos baixos, bem como precisam de um tempo maior para ação de anestésicos venosos, devido à circulação mais lenta. ^911^ Além disso, a insuficiência hepática, consequente à IC direita, e a insuficiência renal alteram a farmacocinética da maioria das medicações. 

 A disfunção autonômica do paciente com CCDC reduz a reserva contrátil e pode atenuar a ação de catecolaminas exógenas, requerendo doses acima das usuais para estabilização hemodinâmica. ^422^ O melhor esquema anestésico deve promover o menor grau de depressão miocárdica e de vasodilatação possível. Todo anestésico inalatório e a maioria dos anestésicos venosos são depressores do miocárdio, ^912^ necessitando de titulação e monitorização criteriosa por parte do anestesista. Sempre que possível, conforme o tipo de cirurgia, técnicas de anestesia regional isoladamente ou em associação com a anestesia geral devem ser utilizadas por apresentarem menor risco de instabilidade hemodinâmica. ^910^

 Pacientes com CCDC frequentemente são portadores de dispositivos eletrônicos implantáveis para o tratamento de arritmias e/ou IC. Em caso de utilização do termocautério durante a cirurgia, tais dispositivos requerem atenção específica. A produção de ruídos elétricos pelo termocautério leva a interpretação equivocada dos eventos elétricos do coração por parte do dispositivo, com consequente inibição de estímulos elétricos necessários ou liberação de terapias de choque elétrico inapropriadas. Os portadores de MP devem ter o dispositivo programado no modo DOO ou VOO. Os portadores de CDI devem ter as terapias desligadas durante a cirurgia ou utilizarem um imã sobre o dispositivo para inibição de eventuais choques inapropriados. O acesso venoso central deve ser feito com cuidado nesses pacientes pelo risco de o fio guia gerar ruídos pelo contato com o eletrodo de choque, levando a descargas inadequadas. ^913^ Independentemente do tipo de dispositivo, o termocautério deve ser programado no modo bipolar e com a menor potência efetiva, utilizado de forma intermitente e com a placa neutra localizada o mais distante possível da unidade geradora. 

 O pós-operatório de pacientes com CCDC deverá ser feito em unidade de terapia intensiva nos casos com disfunção ventricular ou arritmias cardíacas e nas cirurgias de grande porte. A medicação anticoagulante oral em uso anterior à cirurgia e que necessitou suspensão temporária no transoperatório, bem como as demais medicações para IC e arritmias, devem ser reintroduzidas assim que possível. 

### 13.7. Doença de Chagas e Infecção por Coronavírus

 A disseminação mundial da doença causada pelo novo coronavírus (SARS-Cov-2), a COVID-19, fez com que a OMS a declarasse pandemia em março de 2020. Seguindo o mesmo perfil epidemiológico global, os estudos demonstraram inter-relação entre potencial de gravidade e comorbidades com ênfase em doença cardiovascular e taxas de letalidade maiores em pacientes com essas doenças, comparativamente ao que ocorre na população geral. ^914^

 Dos pacientes com COVID-19, mais de 80% apresentam sintomas leves como febre, dor de garganta e tosse, ^915^ porém as taxas de mortalidade podem ir de 2,3% até 27% ^916 , 917^ em populações vulneráveis, incluindo idosos e pacientes com comorbidades, ^918 , 919^ devido a complicações graves, como pneumonia, tromboembolismo, sepse, insuficiência renal e cardíaca. ^920 , 921^ A infecção por SARS-CoV-2 pode afetar o sistema cardiovascular por diversos mecanismos, incluindo lesão miocárdica inflamatória (miocardite), tromboses intravasculares, síndrome de Takotsubo, causando IC, arritmias e choque circulatório. ^922^ Pacientes com IC têm maior mortalidade por COVID-19 do que pacientes sem IC, ^923^ podendo chegar a 40%. ^924^ Assim a preexistência de IC é fator de risco indubitável para mortalidade por COVID-19. ^923^

 A consequência da pandemia da COVID-19 sobre o estado de saúde de pacientes com DC ainda é, em grande parte, desconhecida. ^71^ Por muitos deles serem cardiopatas, são vulneráveis a infecções graves e podem ter complicações graves causadas pela COVID-19, ^925^ inclusive maior mortalidade quando apresentam IC. ^923^ Além disso, há alta prevalência de comorbidades na população com DC que está envelhecendo graças a medidas de controle da transmissão de DC e melhora global do sistema de saúde. ^926^ Assim, é possível, até provável, que haja maior morbidade/mortalidade relacionada à COVID-19 em pacientes com DC. No entanto, registro amplo recente no Brasil indicou que a mortalidade intra-hospitalar por COVID-19 foi similar entre pacientes com e sem DC, pareados por sexo, idade, hipertensão e diabetes *mellitus* , mesmo sendo a IC e a FA mais prevalentes no grupo com a infecção crônica por *T. cruz* i. ^104^

 A prevenção da COVID-19 para os pacientes com DC, cursando em qualquer fase da moléstia, segue as mesmas recomendações para a população em geral, contidas nas diretrizes do Ministério da Saúde do Brasil, porém com recomendações redobradas e atenção especial às indicações de vacinas, de acordo com a faixa etária, para profilaxia das infecções por pneumococos, vírus influenza e COVID-19. ^927^ Os pacientes com DC têm indicativo prioritário de vacinação anti-COVID-19 e configuram grupos de risco importantes nas estratégias vacinais, tanto para COVID-19 como para outras doenças imunopreveníveis por vacinas, com risco de desenvolvimento de pneumonias graves e/ou acometimento cardíaco. 

**Figura 13.1:**
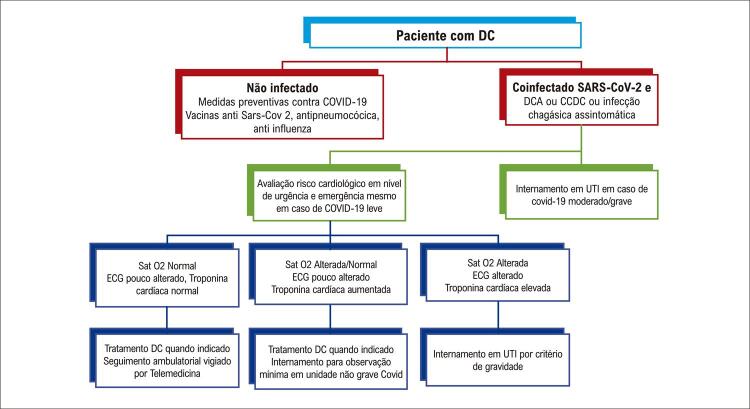
– Fluxograma recomendado para atenção médica ao paciente com doença de Chagas em duas situações: prevenção de COVID-19 ou coinfectados. CCDC: cardiomiopatia crônica da doença de Chagas; DC: doença de Chagas; DCA: doença de Chagas aguda

 Para indivíduos com DC que adquirem a infecção por SARS-CoV-2, recomenda-se que os cuidados médicos devam ser instituídos desde o nível de APS, com ênfase nas condições de risco associados à miocardite e aos fenômenos intravasculares tromboembólicos ( Figura 13.1 ). 

 Em casos moderados ou graves de COVID-19, pode-se usar corticoterapia, quase sempre indicada a partir do 6º dia de doença e utilizada por período curto de tempo. Não há qualquer contraindicação ao uso de corticoides nos pacientes com DC coinfectados com SARS-CoV-2, uma vez que seu efeito imunodepressor, que poderia ser danoso para a resposta do paciente ao *T. cruzi* , não é alcançado nas doses recomendadas e por períodos curtos de tempo. Contudo, o manejo e as indicações devem ser conduzidos por associação de infectologista e cardiologista. ^928^

 Nos pacientes com CCDC e COVID-19 leve, devem-se manter as medicações cardiovasculares e a anticoagulação anteriormente indicadas, já que não há indicativos de que sejam prejudiciais. Nos casos moderados ou graves, a anticoagulação oral será trocada por heparina de baixo peso molecular e a medicação cardiovascular deverá ser reavaliada, conforme a hemodinâmica do paciente. 

### 13.8. Transplante Não Cardíaco e Terapia Imunossupressora 

 A transmissão da DC por transplante de órgãos foi descrita pela primeira vez no Brasil em 1981 após transplante de rim. ^929^ Depois disso, vários relatos de transmissão de DC após doação de rim, fígado, coração ou medula óssea surgiram na literatura mundial, sendo que a taxa de transmissão variou conforme o órgão, por exemplo de 13% ^930^ a 16% ^931^ no caso de transplante de rim, 20% ^930^ a 22% ^932^ no de fígado e até 75% no de coração. ^930^ Além disso, foi detectada RDC em receptores de órgãos sólidos de pacientes com DC crônica. 

 A maior experiência é com transplante de rim, onde a RDC ocorre principalmente no primeiro ano, mas varia amplamente entre os centros, de 8% a 22%. ^933 , 934^ No caso do transplante de fígado, a experiência ainda é limitada, observando-se, porém, que a incidência de RDC varia conforme o centro de forma similar à do transplante de rim. ^934^ Outra situação é o transplante de medula óssea em pacientes com DC crônica assintomáticos, no qual o risco de RDC variou de 17% a 40%. ^583^

 No Brasil, a Portaria n ^o^ 2.600 de 2009 determina a testagem para DC: (1) em todas as doações, seguindo-se os mesmos algoritmos utilizados para triagem de doadores de sangue; (2) para fins de inscrição dos potenciais receptores de órgãos no Cadastro Técnico Único; e (3) em todos os cadáveres potenciais doadores de órgãos, tecidos, células ou partes do corpo antes da alocação dos enxertos. A portaria também estabelece que o coração de doadores com DC não deva ser utilizado em transplante, enquanto rim, pâncreas, fígado e pulmão de doadores com DC podem ser transplantados, desde que autorizado pelo receptor e equipe de transplante, apesar do risco de transmissão e implicando em necessidade de monitorização após o procedimento. 

#### 13.8.1. Doador com Doença de Chagas e Receptor sem Doença de Chagas 

 O risco nesse caso é de transmissão da DC. No caso de doador vivo, idealmente ele deverá ser tratado com benznidazol por 60 dias antes do procedimento. Caso seja imperativo que o transplante ocorra antes de se completar o tratamento, o transplante poderá ser realizado após 14 dias de tratamento, ^2 , 651 , 934^ com base na queda da parasitemia nessa fase do tratamento. ^587^

 No caso de doador não tratado, a conduta mais recomendada é a monitorização da ocorrência de transmissão da DC e o tratamento dos casos diagnosticados, quando se observam bons resultados, com alta taxa de cura. ^2 , 583 , 931 , 935 , 936^ Já o uso profilático de benznidazol, que seria aplicável de rotina, é controverso, alegando-se a toxicidade do fármaco associada à baixa taxa de transmissão. 

 A monitorização é feita com pesquisa direta de *T. cruzi* no sangue periférico semanalmente, até 60 dias, e exames parasitológicos indiretos e sorológicos aos 30 e 60 dias após o transplante. A seguir, exames clínicos, sorológicos e parasitológicos (diretos/indiretos/PCR) devem ser realizados a cada 2 meses até 1 ano de seguimento; posteriormente, a cada 6 meses, enquanto persistir a imunossupressão (tempo dependente da modalidade e do tipo de transplante). Além dos controles habituais, qualquer sinal clínico suspeito de DC aguda deverá ser investigado por meio de exames parasitológicos. 

 O PCR pode ser utilizado em lugar de exames parasitológicos indiretos. ^2^ Em qualquer momento, caso seja detectada infecção aguda, o tratamento antiparasitário convencional deverá ser instituído. ^2^ Também é importante ressaltar que exames sorológicos podem não se positivar devido à imunossupressão vigente nesses pacientes. A monitorização é mais frequente no início, já que a maioria dos casos de transmissão com infecção aguda se dá entre 3 e 29 semanas (média de 8). ^930^

 Ao comparar 13 pacientes que não foram monitorados corretamente com 19 que fizeram monitorização semanal, viu-se que, no primeiro grupo, 5 pacientes tiveram diagnóstico de DC sintomática, dos quais 4 morreram, enquanto que, no outro grupo, 4 transmissões foram confirmadas e receberam tratamento antiparasitário e não desenvolveram doença sintomática. ^930^ Os testes parasitológicos devem ser feitos semanalmente durante o tratamento ou até que dois testes consecutivos negativos sejam obtidos. ^934^

#### 13.8.2. Receptor com Doença de Chagas

 A prevalência de DC entre candidatos a transplante de órgãos sólidos é maior no de coração, devido à própria característica específica de ela cursar com IC refratária em muitos casos. 

 Embora RDC possa ocorrer durante períodos de imunossupressão após qualquer transplante de órgão sólido, formas graves dessa complicação, como meningoencefalite ^937^ e lesões tumor-símile intracerebral (“chagomas”), ^938^ são incomuns. Em recipientes de transplante renal, RDC ocorre principalmente no primeiro ano após o transplante ou quando se intensifica a imunossupressão após episódios de rejeição. A RDC pode ser totalmente assintomática e, quando manifestações clínicas aparecem, elas são usualmente na forma de envolvimento subcutâneo (eritema nodoso-like, paniculite) em membros. Se o tratamento não é instituído, as lesões podem evoluir para úlceras dolorosas. Miocardite e encefalite também são descritas, mas menos frequentemente. A resposta ao tratamento é boa, com sobrevida adequada do paciente e do enxerto a longo prazo. ^933^

 Analogamente ao exposto acima, para outro contexto similar, há duas condutas debatidas para o receptor de transplante que já tem DC diagnosticada: fazer-se tratamento antes do transplante no receptor já assim infectado ou instituir-se conduta expectante para diagnóstico e tratamento da eventual RDC. O tratamento rotineiro de recipientes assintomáticos, mas com DC, antes de receberem o transplante poderia, teoricamente, reduzir a chance de RDC após a imunossupressão; não há evidência conclusiva, porém, a favor dessa assertiva e da correspondente conduta profilática. Ao contrário, falha dessa conduta já foi relatada. ^939^

 Portanto, a conduta preferida é a monitorização de rotina da parasitemia e de outras evidências de RDC, de forma a se poder instituir tratamento específico precocemente e aumentar o sucesso do tratamento com menor número de casos graves ou fatais. ^2 , 934^ Ademais, em geral, o resultado do tratamento da RDC é favorável, com altas taxas de cura e baixa mortalidade. 

 Todos os receptores infectados com *T. cruzi* devem ser acompanhados para investigar RDC semanalmente nos primeiros 2 meses, a cada 2 semanas do terceiro ao sexto mês e mensalmente depois disso até 1 ano, e semanalmente por 2 meses após intensificação de imunossupressão, ou a qualquer tempo se houver suspeita clínica de DC aguda. ^934 , 940^

 Os testes de laboratório de preferência são os parasitológicos diretos. O PCR a ser usado deve ser o quantitativo, já que o qualitativo pode ser positivo em pacientes assintomáticos. A vantagem do qPCR é ser mais sensível e positivar-se mais precocemente que os métodos parasitológicos diretos. ^401^ Ninhos de formas amastigotas de *T. cruzi* devem ser procurados em todas as biópsias. O diagnóstico de RDC é feito pela identificação de parasitas no sangue periférico por meio de métodos diretos ou por qPCR, conforme descrito anteriormente, ou identificação de *T. cruzi* em biópsias. RDC deve ser considerada em pacientes com febre inexplicada, dermopatia, miocardite ou encefalite. 

 Todos os recipientes infectados devem ser investigados uma vez por ano para as formas cardíaca e digestiva da DC. Todos com RDC devem ser tratados por 60 dias com benznidazol (5mg/kg/dia), sendo o nifurtimox (8mg/kg/dia) a segunda escolha. Durante o tratamento, testes parasitológicos devem ser feitos semanalmente até dois testes negativos serem obtidos. ^934^

 É importante frisar que os testes sorológicos não são úteis para diagnóstico de RDC e que a soroconversão negativa já foi descrita em pacientes com DC crônica após receberem transplantes, devido à imunossupressão. ^931^

 Outro ponto ainda incerto é o uso de protocolos específicos quimioterápicos podendo influenciar a RDC. Assim, evitar globulina antitimócitos e minimizar o uso de micofenolato parece recomendável. Alguns estudos sugerem que inibidores de mTOR ( *mechanistic target of rapamycin* ) poderiam favorecer o controle da replicação do *T.* cruzi, ^941^ assim constituindo um regime mais apropriado para pacientes em risco de DC. Porém, ainda não há um regime ótimo estabelecido. ^655^

#### 13.8.3. Doenças Autoimunes

 A experiência com DC associada a outras doenças com imunocomprometimento é escassa e limitada principalmente a relatos de casos. A maioria desses está relacionada a lúpus eritematoso sistêmico. ^942^ Sendo assim, recomenda-se vigilância para RDC e tratamento apropriado. Também não há evidência a favor do uso profilático de benznidazol antes do uso de corticoide em dose imunossupressora, sendo a monitorização da possibilidade de RDC a melhor conduta. 

## 13.9. Doença de Chagas e Senescência

 O sucesso de políticas públicas de controle da transmissão da DC, aliado à elevação da expectativa de vida do brasileiro, assim como a melhora das condições de moradia em regiões endêmicas, vem mudando o perfil desses pacientes, assim propiciando aumento da média de idade dos indivíduos infectados cronicamente pelo *T. cruzi.* ^926 , 943^ Contudo, a infecção por *T. cruzi* permanece como preditor independente de mortalidade por todas as causas e de AVC entre pessoas idosas. ^70 , 849^ Esse fato lança novos desafios para a atenção ao paciente com DC, quando as doenças degenerativas do idoso, a HAS, o diabetes *mellitus* , a dislipidemia e a doença coronariana se somam ao agravo ao coração causado pela DC, assim podendo influenciar o prognóstico e a qualidade de vida dessa população. 

 Porém, a informação sobre como a DC se apresenta entre indivíduos idosos é escassa, uma vez que a maioria dos estudos longitudinais prévios foi realizada há muito tempo, em populações com predomínio de adultos jovens. ^404^

 Estudos transversais conduzidos no Ceará, ^944^ em Campinas (SP) ^523^ e no Rio de Janeiro ^926^ em pacientes idosos com DC atendidos ambulatorialmente registraram a HAS como a mais frequente comorbidade. 

 Além da HAS, outras comorbidades também foram relatadas, como dislipidemia, osteoporose, osteoartrite, diabetes *mellitus* , IC, insuficiência coronariana, hipotireoidismo, dispepsia, depressão, AVC e insuficiência renal. Portanto, esses pacientes merecem especial atenção. Além disso, a presença de comorbidades crônicas pode resultar em frequentes consultas médicas e risco de interações medicamentosas, efeitos adversos, bem como uso diário de cinco ou mais medicações de difícil domínio de administração correta por parte do idoso. ^945^

 Nos estudos transversais citados, a forma clínica predominante da DC foi a de cardiomiopatia; porém, a informação sobre o valor prognóstico das alterações entre idosos ainda é escassa. ^946 , 947^ Em estudo realizado em Bambuí, em coorte de pacientes idosos com ou sem DC, as alterações eletrocardiográficas eram nitidamente mais frequentes em pacientes com DC. ^303^ Anormalidades do ECG significantemente associadas com DC foram bradicardia sinusal, extrassístoles ventriculares ou supraventriculares frequentes, FA, BRD, BDASE, BAV de 1º grau e intervalo QT prolongado. 

 O BRD, em especial associado ao BDASE, foi fortemente associado à presença de DC, sendo observado em 40% da população com DC e em apenas 8% dos idosos sem DC. Variáveis do ECG independentemente associadas com maior risco de morte em pacientes com DC foram extrassístoles ventriculares ou supraventriculares frequentes, FA, BRD, zona elétrica inativa, alterações primárias da repolarização ventricular e hipertrofia ventricular esquerda. Aqueles com ECG normal ou alterações menores não tinham maior risco de morte, quando comparados com a população não infectada. ^303^

 Muitos idosos nunca tiveram uma avaliação clínica inicial para a classificação da DC e receberem o acompanhamento e tratamento adequados. Pode-se constatar isso em estudo transversal conduzido em área endêmica de São João do Piauí, na região semiárida brasileira. Esse estudo evidenciou alta prevalência de DC nos idosos, chegando a 34% no grupo de 61 a 75 anos e 39% no grupo acima de 75 anos. Nessa região, apesar do controle de transmissão da doença, o diagnóstico e tratamento foram interrompidos e muitos idosos nunca tiveram avaliação clínica inicial. Essa região, assim como outras com características socioambientais semelhantes da região semiárida brasileira, continua sofrendo pela escassez de grupos de APS treinados para diagnosticar e tratar a população. ^948^

 Estudos clássicos em áreas endêmicas mostram que a FIDC é a mais prevalente e que 30% a 40% desses indivíduos podem persistir indefinidamente com essa variante clínica. ^300^ Em contraste com essas noções bastante fundamentadas, um estudo descreve que apenas 13% dos idosos têm ECG normal e sugerem que a gravidade da DC em idosos possa ser similar à observada em adultos jovens. ^303 , 947^ Tais informações, obviamente, carecem de mais substancialidade e comprovação. 

## 14. Recomendações para Constituição de Serviços Estruturados para Acompanhamento de Pessoas com Cardiomiopatia Crônica da Doença de Chagas 

 Considerando o impacto de fatores sociais, econômicos e culturais na gênese e na evolução da CCDC, o manejo clínico em serviços de saúde requer a conformação de uma rede de atenção em um modelo que transcenda dimensões biomédicas. Para tanto, deve garantir acesso à assistência integral, hierarquizada e descentralizada, contemplando o processo de determinação social que permeia essa doença negligenciada, causa e consequência de pobreza estrutural. 

 Como já descrito, pessoas com CCDC apresentam elevada carga de morbimortalidade quando comparadas a pessoas com outras cardiomiopatias. Em sua grande maioria, pertencem a classes sociais menos favorecidas com elevados graus de vulnerabilidade, o que dificulta, sobremaneira, o acesso a diagnóstico e tratamento. ^949^ Não raro, as pessoas com CCDC são as que se encontram em condições críticas para alcance da atenção, o que inclui, por exemplo, itinerário terapêutico longo, baixa resolutividade e diagnóstico tardio, muitas vezes em estágios avançados da doença. 

 Além disso, pacientes com CCDC enfrentam preconceito e estigma em diferentes contextos na sociedade, o que acaba por agravar mais ainda o seu sofrimento não apenas físico, mas também psicológico e social. A DC está incluída no rol das enfermidades mais negligenciadas em todo o mundo, especialmente na América Latina, segundo a OMS. Trata-se de uma condição crônica bastante desafiadora para qualquer sistema de saúde pública, uma vez que os acometidos podem demandar ações desde a baixa e média complexidade tecnológica no setor saúde, em aproximadamente 70% a 80% dos casos (em grande parte na APS), até situações que requerem acesso à atenção terciária e quaternária, ampliando os custos relacionados à saúde pública. Reitera-se também, sobremaneira, o crítico impacto negativo na qualidade de vida das pessoas acometidas, além de suas famílias e comunidades. ^950 - 952^

 No Brasil, entre 2000 e 2010, a carga da CCDC correspondeu a um total de 7.402.559 anos potenciais de vida comprometidos, sendo 9% desse total devido a anos de vida perdidos e 91% a anos de vida com incapacidade. ^953^

 O SUS, em sua concepção hierarquizada e descentralizada, foi pensado com a finalidade de alcançar a integralidade como referencial, particularmente a partir de territórios da APS, com apoio matricial inclusive por serviços de referência em casos mais complexos. Entretanto, requer investimentos aliados a uma gestão pública qualificada e amplamente engajada, que permita estruturação de uma rede de atenção fundamentada em linhas de cuidado em forte integração com ações de vigilância em saúde. 

 Apontam-se, no entanto, alguns fatores para que o débito sanitário com essas pessoas acometidas pela DC permaneça presente, mesmo 113 anos após a sua descoberta. Como exemplo desse ciclo de negligência, trata-se de uma doença que alcança uma população silenciosa e silenciada, com persistentes falhas da ciência, do mercado e da saúde pública. 

 Persistem questões básicas a serem respondidas nos contextos endêmicos: quem são essas pessoas? onde estão? como estão? ^951 , 954^

 Em contextos de maior complexidade no manejo clínico, ao se recomendar a constituição de serviços estruturados de acompanhamento a pessoas com CCDC, alguns aspectos precisam ser observados, como espaço ambulatorial apropriado, vinculado ou com retaguarda de um hospital terciário ou quaternário em cardiologia, com possibilidade de realizar exames complementares de média e alta complexidade para estadiamento adequado do comprometimento cardíaco. 

 Deve ser considerada também a necessidade do acompanhamento de casos residentes em regiões de difícil acesso a serviços com melhor estruturação, como, por exemplo, região Amazônica, áreas do país com características rurais, urbanas e de periferias de cidades. Para esses casos, pode ser necessária a utilização de meios tecnológicos diferenciados, como a consulta por meio de telemedicina, elaboração de laudos de ECG e radiografia de tórax à distância, dentre outros. 

 Serviços de saúde estruturados em CCDC podem tornar-se referência regional e estadual para casos com manejo clínico mais complexo, tendo como objetivo o esclarecimento diagnóstico e o estadiamento do comprometimento visceral. Além disso, podem apoiar matricialmente os programas estaduais e municipais no processo de educação permanente de profissionais das unidades de saúde da APS (considerando toda a equipe de saúde), o que inclui agentes comunitários de saúde e agentes de combate a endemias, no manejo clínico da DC, uma vez que, embora endêmica, ainda é subdiagnosticada. 

 Para que um serviço de saúde estruturado tenha seu pleno funcionamento, faz-se necessária a composição de equipe multiprofissional em caráter interdisciplinar, reconhecida como a melhor forma de atenção longitudinal e integral a condições crônicas. Para além do diagnóstico e tratamento oportunos, requer ações de reabilitação e prevenção quaternária. 

 Ao criar-se um serviço destinado e vocacionado a pessoas com CCDC, torna-se importante contemplar suas peculiaridades, procurando compreendê-las dentro de um contexto biopsicossocial, exercendo a medicina onde a atenção é centrada na pessoa acometida e não apenas na doença ou no órgão por ela afetado. 

 Nessa proposta de trabalho, a equipe deve reconhecer os elementos comuns que demandam forte interação entre cada profissional, mas também as especificidades do processo de trabalho delimitado por suas possibilidades e responsabilidades de atuação. É necessário que essa equipe tenha conhecimento acerca da CCDC, assim como da rotina de seu manejo, para que todos falem uma mesma linguagem. Dessa forma, busca-se evitar informações distorcidas ou mesmo inverídicas. ^951 , 955 , 956^

 O serviço estruturado para condução de casos de CCDC deve dispor idealmente dos seguintes profissionais: médico/a (cardiologia, clínica médica, infectologia, gastroenterologia), enfermeiro/a, psicólogo/a, nutricionista, farmacêutico/a, fisioterapeuta, educador/a físico/a e assistente social, podendo ser ampliado de acordo com a adoção de novas intervenções. A dimensão da equipe deverá ser ajustada à realidade local, às possibilidades de cada serviço de saúde e, acima de tudo, à demanda trazida pelas pessoas acometidas. ^951 , 955^

### 14.1. Atribuições dos Serviços Estruturados para Acompanhamento de Pessoas com Cardiomiopatia Crônica da Doença de Chagas

1. Acolher todos os casos vindos de: unidades da APS, unidades de atenção secundária [Unidades de Pronto-Atendimento Especializado (UPAE)], emergências cardiológicas e não cardiológicas, maternidades, hemocentros públicos ou privados, serviços de transplantes e serviços especializados em HIV/AIDS para esclarecimento diagnóstico e realização de estadiamento;
2. Para confirmar o diagnóstico da DC é necessária anamnese qualificada, dirigida ao contexto clínico epidemiológico, com confirmação sorológica preferencialmente pelo LACEN;
3. Realizar notificação compulsória dos casos crônicos diagnosticados de acordo com a publicação da Portaria n ^o^ 1.061, de 18 de maio de 2020 do Ministério da Saúde, que facilitará a melhor organização da rede de INSS da prevalência da DC crônica no Brasil (Ministério da Saúde do Brasil); ^957^
4. Estadiar, por meio da utilização de exames complementares, o grau de comprometimento cardíaco, mantendo diálogo permanente com as Unidades Básicas de Saúde (UBS) e UPAE, de forma descentralizada, para que o fluxo de referência e contrarreferência seja efetivado. Casos na FIDC ou com dano cardíaco não significativo poderão ser acompanhados nas UBS, próximo ao domicílio, diminuindo, assim, a necessidade de tratamento fora do domicílio;
5. Pessoas acometidas com indicação de tratamento etiológico deverão seguir as recomendações indicadas em capítulo específico destas diretrizes da SBC e poderão ser acompanhados nas UBS, desde que a equipe de saúde esteja habilitada ao manejo clínico desses casos; ^957^
6. Mulheres em idade fértil devem ser orientadas sobre a possibilidade de transmissão congênita da DC quando grávidas e orientadas quanto a métodos de contracepção. Caso desejem ou já estejam grávidas, devem ser acompanhadas pela equipe de APS em articulação com serviço de obstetrícia de referência e receber tratamento de acordo com as diretrizes vigentes; ^957^
7. Casos de DC com IC, arritmias complexas, necessidade de implantes de MP, CDI e TC deverão permanecer em acompanhamento em serviço de maior complexidade. Em alguns casos, o uso de DACM pode ser necessário como intervenção intermediária para TC ou como alternativa ao TC com bons resultados; ^8^
8. Identificar comprometimento digestivo associado e, quando presente, orientar ou encaminhar para serviço de referência em DC; ^957^
9. Tratar as comorbidades ou avaliar a necessidade de encaminhar os casos para interconsulta em serviços especializados; ^951^
10. A reabilitação cardíaca deve estar integrada a serviços estruturados de atenção a pessoas com CCDC pelo benefício clínico comprovado do exercício físico sob supervisão para a saúde e qualidade de vida; ^897^
11. Pessoas com dificuldade no entendimento de prescrições da equipe de saúde podem ser auxiliadas por profissional farmacêutico compondo a equipe multiprofissional de assistência, com a finalidade de esclarecer a posologia, intervalo entre doses, eventos adversos, interações medicamentosas e estratégias para alcance de soluções; ^552^
12. Propiciar ações educativas (presenciais ou virtuais) permanentes com a pessoa acometida, familiares e cuidadores/as sobre a doença e o autocuidado, objetivando a identificação oportuna de sinais e sintomas de descompensação cardíaca, disponibilizando canal de comunicação (por exemplo, DISC Chagas, DISC IC) e diversas mídias sociais. Com a difusão de meios de comunicação, celulares tipo *smartphones* e internet, o atendimento remoto tem sido de grande importância na condução de pacientes mais graves que não podem aguardar por uma consulta ou comparecer presencialmente para pequenos ajustes, fato comprovado especialmente durante a pandemia da COVID-19;
13. Esclarecer sobre os dispositivos intracardíacos, função e necessidade de implante de MP ou CDI, bem como de TC, procurando desfazer mitos e crenças que podem impactar negativamente a qualidade de vida e a adesão aos tratamentos propostos, assim como sobre a impossibilidade em doar sangue, órgãos e tecidos; ^951^
14. Valorizar o conhecimento experiencial das pessoas acometidas sobre sua própria doença, convidando-as a participar de reuniões educativas, possibilitando a troca de vivências, potencializando a autonomia e o empoderamento, estimulando a mudança de postura de sujeito passivo a ativo no seu processo terapêutico e suas demandas; ^958^
15. Reuniões de grupo com abordagem de temas específicos como: aspectos nutricionais, atividade física, depressão, direitos das pessoas com doenças crônicas, aspectos médico-trabalhistas, auxílio-transporte, previdenciários, sexualidade, gestação, amamentação, mitos e verdades sobre DC; ^951^
16. Oferecer suporte psicológico objetivando diminuir o estigma, o autopreconceito, os tabus e as crenças inadequadas em relação à doença. Esclarecer sobre a prevenção de fatores agravantes como álcool, tabagismo, drogas lícitas e ilícitas em sua doença; ^951 , 958^
17. Desenvolver ações de educação permanente junto a profissionais da saúde, com enfoque específico sobre as peculiaridades da CCDC, estimulando o ensino, a pesquisa e a extensão multiprofissional; ^951 , 958^
18. Identificar pela busca ativa e o aprofundamento da relação médico-paciente, outros membros da família no mesmo contexto de risco e vulnerabilidade da exposição ao *T. cruzi* (inclusive diante da possibilidade de transmissão congênita) e, nos casos confirmados, incorporá-los ao serviço para determinar o fluxo terapêutico a ser seguido; ^951^
19. Estimular e apoiar a criação de novas associações de pessoas acometidas pela DC, visando a melhor integração entre elas, estabelecendo um canal de comunicação ativo e propositivo junto à sociedade, particularmente a comunidade científica, política e sanitária, a respeito de suas reivindicações baseadas no direito à saúde. Abre-se, portanto, um forte canal em busca da cidadania ativa, em prol de si próprio e da coletividade, transformando a sua dor e o seu sofrimento em um ato político;
20. Apoiar sempre a luta contra preconceitos, a exemplo da necessária superação da adjetivação pelo termo “chagásico”, que reduz a pessoa acometida pela doença em si. Na prática clínica, significa substituir o termo “chagásico” por “pessoa acometida ou afetada pela DC”; ^951 , 954 , 959^
21. Divulgar a existência da FINDECHAGAS, criada em 2010, assim como do dia 14 de abril, como Dia Mundial da DC, reconhecido pela OMS em 2019; ^954^
22. Criar serviços de telemedicina para a realização de consultas e elaboração de laudos de exames complementares, como ECG e radiografia de tórax. De acordo com essa avaliação à distância para apoio matricial, encaminhar os casos selecionados para atendimento em serviços estruturados.

### 14.2. Benefícios Esperados dos Serviços Estruturados para Acompanhamento de Pessoas com Cardiomiopatia Crônica da Doença de Chagas 

 Serviços de referência estruturados para acompanhamento de pessoas com CCDC poderão comprovar o que tem sido descrito para outras doenças crônicas. ^955^ Espera-se que, uma vez estruturado, o serviço seja capaz de proporcionar:

Fortalecimento da relação entre o profissional de saúde e a pessoa acometida pela DC;
Desenvolvimento de escuta ativa e qualificada e de aconselhamento para a DC;
Melhor conhecimento sobre a doença entre profissionais de saúde e pessoas acometidas;
Favorecimento de maior adesão ao tratamento farmacológico e não farmacológico;
Menor morbimortalidade com diminuição de atendimentos de emergência e reinternações hospitalares;
Impacto positivo na qualidade de vida; diversos estudos recentes focalizam esse relevante conceito com dados fundamentados e coerentes; ^43 , 515 , 960^
Redução do estigma e do preconceito;
Maior empoderamento, autonomia e motivação das pessoas acometidas para desenvolverem ações de autocuidado e buscarem seus direitos (saúde, educação, dentre outros);
Redução dos custos para saúde pública.

Embora se saiba que aparentemente a implantação de um serviço estruturado implica em investimento (financeiro e técnico-operacional), acredita-se que a sua estruturação nas redes de atenção poderá ter uma relação favorável de custo e efetividade em médio e longo prazos. 

 Em suma, os serviços estruturados têm como missão precípua promover assistência que favoreça a estabilidade clínica, psicológica e social de todas as pessoas acometidas pela DC. 

## 15. Definição de Cardiopatia Grave e Avaliação Médico-Trabalhista 

### 15.1. Introdução

 A CCDC, ainda prevalente no Brasil, pode cursar com IC, arritmias ventriculares e distúrbios de condução do estímulo elétrico, AVC e outras complicações tromboembólicas, pulmonares e sistêmicas, configurando, portanto, situações graves, ^7^ muitas das quais com implicações sociais e trabalhistas. 

 O termo “cardiopatia grave”, cunhado por uma equipe multidisciplinar, foi referido pela primeira vez na legislação brasileira no ano de 1952 mediante o Estatuto dos Funcionários Civis da União, pela lei 1.711 (item 11, artigo 178), e definido como “doença que leva, em caráter temporário ou permanente, a redução da capacidade funcional do coração, a ponto de acarretar risco à vida ou impedir o servidor de exercer suas atividades laborais”. ^961^ De acordo com aquele documento, o médico perito tinha que se valer de dados subjetivos para concluir a sua avaliação diagnóstica. Todavia, com os avanços da medicina pericial, baseados em melhor conhecimento da evolução clínica e prognóstico de pacientes com CCDC, além dos avanços relacionados aos métodos complementares que diagnosticam a disfunção cardiovascular, a caracterização de cardiopatia como entidade mórbida evoluiu, tornando-se necessário que o diagnóstico seja respaldado por avaliação clínica rigorosa e comprovação laboratorial, conforme reza a II Diretriz Brasileira de Cardiopatia Grave da SBC, publicada em 2006. ^962^

### 15.2. Conceito e Âmbito

 Primeiramente, é preciso destacar que o termo “cardiopatia grave” pode ser encontrado em diversos processos judiciais, conforme explicitado na Lei Federal nº 7.713/1988, artigo 6º, inciso XIV. ^961^ Por cardiopatia grave entende-se um amplo grupo de enfermidades e condições clínicas, de origem cardiológica, em que ocorre redução significativa na perspectiva de sobrevida ou limitação significativa na capacidade funcional, ou ambas as situações. A tipificação de cardiopatia grave se destina precipuamente a atender questões na esfera trabalhista (tais como aposentadoria por invalidez, alteração de cargo e adaptação ao ambiente de trabalho) ou proporcionar benefícios financeiros (liberação do FGTS e de PIS/PASEP) e fiscais (isenção de imposto de renda, conforme descrito na portaria normativa Nº 1174/MD do Manual do Ministério da Defesa, de 06 de setembro de 2006, capítulo III) ou de aumento de proventos (adicional de 25% do valor da aposentadoria para condições em que haja a necessidade de um cuidador). 

 Em segundo lugar, é importante esclarecer que o *status* de cardiopatia grave é definido somente após a utilização apropriada de tratamento clínico ou cirúrgico, quando recomendados, e identificada a ausência de resposta satisfatória, ou ainda em situações em que não há recursos terapêuticos satisfatórios ou, se houver, eles não são suficientes para modificar a situação clínica e prognóstica do indivíduo. 

 Alterações ocasionais em exames complementares não implicam em diagnóstico automático de cardiopatia grave. De fato, a verificação das limitações funcionais e a avaliação do prognóstico decorrem de ampla investigação e contextualização do cenário clínico do paciente com cardiopatia. Dito de outra maneira, entre os principais critérios de inclusão no rol de cardiopatias graves, deve-se assegurar a realização de uma avaliação clínica completa, algo que permita obter informações sobre a capacidade funcional do paciente, e, paralelamente, obter informações acerca da taxa estimada de sobrevida para a referida situação. 

 A primeira parte é realizada mediante consulta médica com anamnese e exame físico detalhados, complementados pela realização de exames, tais como ECG, radiografia de tórax, ecodopplercardiograma, Holter de 24 horas, teste ergométrico ou ergoespirométrico, dentre outros. Em situações específicas, podemos recorrer a exames mais sofisticados ou invasivos, como cintilografia miocárdica, RMC, angiotomografia ou cineangiocoronagrafia. ^963^ A segunda parte decorre do grau de evidência de risco de morte e esse dado deve ser obtido, no caso específico da CCDC, mediante a utilização de escores validados e publicados em emblemáticos periódicos especializados. ^408 , 474 , 964^

### 15.3. Escore Capaz de Predizer o Risco de Óbito em Pacientes com Cardiomiopatia Crônica da Doença de Chagas 

 Infelizmente, a CCDC tem um curso variável e imprevisível, sendo uma das suas apresentações a morte, que pode ser súbita, por evolução progressiva de quadro de IC, ou decorrente de fenômeno tromboembólico. Estimar, portanto, o risco de morte de um paciente com CCDC é um desafio clínico relevante e foi deveras facilitado pela introdução de um escore desenvolvido com essa finalidade. 

 Trata-se do escore idealizado por Rassi Jr. *et al* ., publicado em 2006, acompanhando uma coorte de 424 pacientes com CCDC. ^408^ Durante o período do estudo, aproximadamente 8 anos, 130 pacientes evoluíram para óbito. Os autores identificaram seis variáveis associadas com morte: classe funcional III ou IV da NYHA = 5 pontos; evidência de cardiomegalia na radiografia de tórax = 5 pontos; disfunção ventricular esquerda global ou segmentar na ecocardiografia = 3 pontos; TVNS ao Holter de 24 horas = 3 pontos; QRS do ECG com baixa voltagem em todas as derivações do plano frontal = 2 pontos; e sexo masculino = 2 pontos. Embasados nessa pontuação, os autores definiram três categorias de risco: baixo risco (0 a 6 pontos); risco intermediário (7 a 11 pontos); e alto risco (12 a 20 pontos). Em 10 anos, a mortalidade dos três grupos foi, respectivamente, 10%, 44% e 84%. ^408^

 De posse desse escore, o trabalho do perito pode ser mais facilmente parametrizado, traduzindo em números a realidade clínica do paciente. Portanto, aquele que porventura contabilize ≥ 12 pontos no escore de RASSI seguramente será considerado paciente com cardiopata grave. Entretanto, deve-se destacar que a II Diretriz Brasileira de Cardiopatia Grave da SBC, publicada em 2006, ^962^ vigente e que ainda embasa o diagnóstico pericial de cardiopatia grave, lastreia-se muito mais na capacidade funcional/qualidade de vida do paciente após ter esgotado os recursos terapêuticos habituais, que em ciência de predição de risco. Em que pese a importância do quadro clínico e da classe funcional, a busca por novas ferramentas prognósticas que permitam refinar os dados clínicos será fundamental para subsidiar melhor as perícias médicas e suas conclusões. 

 É notória a necessidade urgente da revisão da referida diretriz para que tais avanços científicos possam ser debatidos com rigor sobre a sua utilidade durante a emissão de laudos periciais de pacientes com a DC. 

### 15.4. Aspectos Clínicos

 Entre os aspectos clínicos característicos da CCDC, são citados IC congestiva, arritmias ventriculares complexas necessitando de implante de CDI, fenômenos tromboembólicos e comprometimentos graves das funções hepática e renal, secundários à doença cardíaca de base. Vale ressaltar que é de importância capital avaliar a condição funcional desses pacientes associada à redução da expectativa de vida, a despeito do arsenal terapêutico otimizado para enquadrá-los na condição de cardiopatia grave causada pela DC. 

### 15.5. Função Pericial

 O perito médico-legal é o profissional capacitado para avaliar e conceder (ou não) o *status* de cardiopatia grave a indivíduos que busquem a Previdência Social, com intuito de receber benefícios decorrentes dessa tipificação. Para exercer essa função, o perito conta, além de sua formação acadêmica na área de saúde, com a realização de cursos de especialização. Existem também diversos manuais que orientam o exercício correto dessa função. Além desses aspectos, há que se considerar aquilo que recebe o nome de “amplexo das leis”. No caso da cardiopatia grave causada pela DC, ela se situa dentro do espectro da cardiopatia grave em geral. Essa se encontra amparada em três leis, que, por sua vez, se referem a respectivos regimes legais: regime jurídico único (lei nº 8.112/90); regime previdenciário (lei nº 8.213/91); e regime fiscal (lei nº 11.052/04). 

 Do ponto de vista didático, pode-se classificar cardiopatia grave nas seguintes subdivisões: 1) cardiopatias agudas, de evolução rápida, podendo transformar-se, progressivamente, em cardiopatias crônicas, caracterizadas por perda da capacidade física do indivíduo e funcional do coração; 2) cardiopatias crônicas, caracterizadas por limitar progressivamente a capacidade física e funcional do coração, ultrapassando os limites de eficiência dos mecanismos de compensação cardíacos, não obstante o tratamento clínico e/ou cirúrgico adequado adotado; 3) cardiopatias crônicas ou agudas que apresentam dependência total de suporte inotrópico farmacológico (dopamina, dobutamina) ou mecânico (balão intra-aórtico, *biopump* ); e 4) cardiopatia terminal, quando a expectativa de vida se encontra bastante reduzida, não responsiva a qualquer tipo de terapia. 

 Diferentemente da Junta Médica, a atuação pericial em saúde decorre rotineiramente da atuação de um único perito, designado para avaliar se o *status* de cardiopatia grave se aplica ao indivíduo em questão. A função pericial exige equilíbrio emocional (a fim de não se deixar influenciar por aspectos alheios aos critérios específicos) e discernimento (a fim de poder, em meio a grande número de documentos, extrair os elementos que permitam tipificar o quadro clínico). Cabe ao perito, de posse do relatório médico e dos exames complementares, reavaliar o indivíduo para o qual se pleiteia o *status* de cardiopatia grave, a fim de validar ou não essa condição. Uma FEVE inferior a 40%, com medicação otimizada, costuma ser um dos principais parâmetros funcionais adotados. De modo geral, é necessária uma avaliação mais ampla, a fim de encampar todos os aspectos do quadro clínico e dos exames complementares, uma vez que há situações limítrofes, em que se observa quadro clínico dissonante dos métodos diagnósticos, resultados divergentes entre exames ou direcionamento para outros dados de igual relevância para a classificação. 

 Em casos de discordância ou divergência nos critérios selecionados para a classificação, tendo o perito negado a presença dessa condição, a via judicial tem sido o recurso a seguir, naturalmente, havendo subsídio documental suficiente para deflagrar a via processual. 

 De maneira sucinta, além da pontuação do escore de RASSI ≥ 12 pontos, outras informações importantes a indicar possível diagnóstico de cardiopatia grave em pacientes com CCDC são: classe funcional NYHA III ou IV isoladamente; episódios de síncope de repetição, sem possibilidade de controle definitivo; presença de TV, principalmente se sintomática ou demandar atendimento emergencial; cardiomegalia acentuada; e presença de trombo no coração ou antecedentes tromboembólicos. ^965^

 Vale ressaltar que a presença de disfunção do nó sinusal sintomática ou de BAV avançado (Mobitz II, 3:1, 4:1, etc e BAVT) não implica necessariamente em limitação funcional permanente, uma vez que o implante de MP pode reverter o quadro clínico e melhorar significativamente o prognóstico, particularmente quando o paciente apresenta essas alterações de forma isolada. No entanto, na CCDC, principalmente nos estágios avançados, é comum a presença de bradiarritmias e bloqueios avançados associados à depressão da função miocárdica ou a arritmias ventriculares complexas, apontando para um maior comprometimento da função cardíaca sob outros aspectos, concomitantemente. Nesses casos, uma avaliação cardiológica ampla, conforme sugerida acima, permite ao perito médico identificar a real situação do paciente em termos de limitação definitiva, tanto no que diz respeito à situação funcional quanto ao prognóstico. 

 De modo análogo, a mera presença de sorologia positiva para DC ou a sua associação com uma alteração eletrocardiográfica, por exemplo, BRD, não é elemento suficiente para a caracterização de cardiopatia grave. Embora se saiba que uma fração desses indivíduos evolua para formas incapacitantes no futuro, a maioria pode permanecer décadas nesse estágio, sem sintomas, ou até completar seu ciclo de vida sem o agravamento clínico dessa enfermidade. 

 A fim de que a função pericial seja exercida em sua plenitude, cabe ao médico-assistente fornecer relatórios detalhados que descrevam com precisão e clareza a situação clínica do paciente e anexar exames que a comprovem. 

### 15.6. Conclusão

 A definição de cardiopatia grave nos tempos atuais encontra-se facilitada pelo avanço do conhecimento da evolução clínica parametrizada, terapêutica clínica e por exames complementares existentes, a grande maioria deles com respaldo científico em termos do prognóstico desses pacientes. A reunião dessas informações, qualificadas e organizadas em forma de escores desenvolvidos em indivíduos brasileiros, é de grande valia para subsidiar o perito em sua avaliação e definição dos mesmos. Entretanto, a capacidade de julgamento clínico do médico deve ser exercitada em toda sua plenitude, agregando sinais e sintomas característicos do paciente em questão, associados a dados dos exames complementares solicitados. 
