# Supplementary material for: Diretriz da SBC sobre Diagnóstico e Tratamento de Pacientes com Cardiomiopatia da Doença de Chagas – 2023
Source: Arq Bras Cardiol. 2023 Jun 16;120(6):e20230269. [Article in Portuguese] doi: 10.36660/abc.20230269 (PMC10344417; doi:10.36660/abc.20230269)
Supplement: Translation

# Translation: SBC Guideline on the Diagnosis and Treatment of Patients with Cardiomyopathy of Chagas Disease – 2023

**Authors:** José Antonio Marin-Neto, Anis Rassi, Gláucia Maria Moraes Oliveira, Luís Claudio Lemos Correia, Alberto Novaes Ramos, Alejandro Ostermayer Luquetti, Alejandro Marcel Hasslocher-Moreno, Andréa Silvestre de Sousa, Angelo Amato Vincenzo de Paola, Antônio Carlos Sobral Sousa, Antonio Luiz Pinho Ribeiro, Dalmo Correia, Dilma do Socorro Moraes de Souza, Edecio Cunha-Neto, Felix Jose Alvarez Ramires, Fernando Bacal, Maria do Carmo Pereira Nunes, Martino Martinelli, Maurício Ibrahim Scanavacca, Roberto Magalhães Saraiva, Wilson Alves de Oliveira, Adalberto Menezes Lorga-Filho, Adriana de Jesus Benevides de Almeida Guimarães, Adriana Lopes Latado Braga, Adriana Sarmento de Oliveira, Alvaro Valentim Lima Sarabanda, Ana Yecê das Neves Pinto, Andre Assis Lopes do Carmo, Andre Schmidt, Andréa Rodrigues da Costa, Barbara Maria Ianni, Brivaldo Markman, Carlos Eduardo Rochitte, Carolina Thé Macêdo, Charles Mady, Christophe Chevillard, Cláudio Marcelo Bittencourt das Virgens, Cleudson Nery de Castro, Constança Felicia De Paoli de Carvalho Britto, Cristiano Pisani, Daniela do Carmo Rassi, Dário Celestino Sobral, Dirceu Rodrigues de Almeida, Edimar Alcides Bocchi, Evandro Tinoco Mesquita, Fernanda de Souza Nogueira Sardinha Mendes, Francisca Tatiana Pereira Gondim, Gilberto Marcelo Sperandio da Silva, Giselle de Lima Peixoto, Gustavo Glotz de Lima, Henrique Horta Veloso, Henrique Turin Moreira, Hugo Bellotti Lopes, Ibraim Masciarelli Francisco Pinto, João Marcos Bemfica Barbosa Ferreira, João Paulo Silva Nunes, José Augusto Soares Barreto-Filho, José Francisco Kerr Saraiva, Joseli Lannes-Vieira, Joselina Luzia Menezes Oliveira, Luciana Vidal Armaganijan, Luiz Cláudio Martins, Luiz Henrique Conde Sangenis, Marco Paulo Tomaz Barbosa, Marcos Antonio Almeida-Santos, Marcos Vinicius Simões, Maria Aparecida Shikanai Yasuda, Maria da Consolação Vieira Moreira, Maria de Lourdes Higuchi, Maria Rita de Cassia Costa Monteiro, Mauro Felippe Felix Mediano, Mayara Maia Lima, Maykon Tavares de Oliveira, Minna Moreira Dias Romano, Nadjar Nitz Silva Lociks de Araujo, Paulo de Tarso Jorge Medeiros, Renato Vieira Alves, Ricardo Alkmim Teixeira, Roberto Coury Pedrosa, Roque Aras, Rosalia Morais Torres, Rui Manoel dos Santos Povoa, Sergio Gabriel Rassi, Silvia Marinho Martins Alves, Suelene Brito do Nascimento Tavares, Swamy Lima Palmeira, Telêmaco Luiz da Silva, Thiago da Rocha Rodrigues, Vagner Madrini, Veruska Maia da Costa Brant, Walderez Ornelas Dutra, João Carlos Pinto Dias

**Affiliations:** 1 Brasil Universidade de São Paulo, Faculdade de Medicina de Ribeirão Preto, Ribeirão Preto, SP – Brasil; 2 Brasil Hospital do Coração Anis Rassi, Goiânia, GO – Brasil; 3 Brasil Universidade Federal do Rio de Janeiro, Rio de Janeiro, RJ – Brasil; 4 Brasil Escola Bahiana de Medicina e Saúde Pública (EBMSP), Salvador, BA – Brasil; 5 Brasil Universidade Federal do Ceará, Faculdade de Medicina, Fortaleza, CE – Brasil; 6 Brasil Centro de Estudos da Doença de Chagas, Hospital das Clínicas da Universidade Federal de Goiás, Goiânia, GO – Brasil; 7 Brasil Instituto Nacional de Infectologia Evandro Chagas, Fundação Oswaldo Cruz, Rio de Janeiro, RJ – Brazil; 8 Brasil Universidade Federal de São Paulo, São Paulo, SP – Brasil; 9 Brasil Universidade Federal de Sergipe, São Cristóvão, SE – Brasil; 10 Brasil Hospital São Lucas, Rede D`Or São Luiz, Aracaju, SE – Brasil; 11 Brasil Universidade Federal de Minas Gerais, Belo Horizonte, MG – Brasil; 12 Brasil Universidade Federal do Pará, Belém, PA – Brasil; 13 Brasil Universidade de São Paulo, Faculdade de Medicina da Universidade, São Paulo, SP – Brasil; 14 Brasil Instituto do Coração do Hospital das Clínicas da Faculdade de Medicina da Universidade de São Paulo, São Paulo, SP – Brasil; 15 Brasil Universidade de Pernambuco, Faculdade de Ciências Médicas, Recife, PE – Brasil; 16 Brasil Instituto de Moléstias Cardiovasculares, São José do Rio Preto, SP – Brasil; 17 Brasil Hospital de Base de Rio Preto,São José do Rio Preto, SP – Brasil; 18 Brasil Secretaria de Saúde do Distrito Federal, Brasília, DF – Brasil; 19 Brasil Escola Superior de Ciências da Saúde, Brasília, DF – Brasil; 20 Brasil Hospital Universitário Professor Edgard Santos, Universidade Federal da Bahia, Salvador, BA – Brasil; 21 Brasil Instituto de Cardiologia do Distrito Federal, Brasília, DF – Brasil; 22 Brasil Hospital das Clínicas da Universidade Federal de Minas Gerais, Belo Horizonte, MG – Brasil; 23 Brasil Hospital das Clínicas da Universidade Federal de Pernambuco,Recife, PE – Brasil; 24 Brasil Hcor, Associação Beneficente Síria, São Paulo, SP – Brasil; 25 Brasil Hospital São Rafael, Fundação Monte Tabor, Salvador, BA – Brasil; 26 França Institut National de la Santé Et de la Recherche Médicale (INSERM), Marselha – França; 27 Brasil Universidade de Brasília, Brasília, Distrito Federal – Brasil; 28 Brasil Instituto Oswaldo Cruz, Fundação Oswaldo Cruz, Rio de Janeiro, RJ – Brasil; 29 Brasil Universidade Federal de Goiás, Faculdade de Medicina, Goiânia, GO – Brasil; 30 Brasil Hospital Universitário Antônio Pedro da Faculdade Federal Fluminense, Niterói, RJ – Brasil; 31 Brasil Hospital Universitário Walter Cantídio da Universidade Federal do Ceará, Fortaleza, CE – Brasil; 32 Brasil DentCor Clínica Médica e Odontológica, Santo André, SP – Brasil; 33 Brasil Instituto de Cardiologia do Rio Grande do Sul, Porto Alegre, RS – Brasil; 34 Brasil Hospital das Clínicas, Faculdade de Medicina de Ribeirão Preto, Universidade de São Paulo,Ribeirão Preto, SP – Brasil; 35 Brasil Instituto Dante Pazzanese de Cardiologia, São Paulo, SP – Brasil; 36 Brasil Grupo Fleury, São Paulo, SP – Brasil; 37 Brasil Universidade do Estado do Amazonas, Boca do Acre, AM – Brasil; 38 Brasil Fundação Zerbini, Instituto do Coração do Hospital das Clínicas da Faculdade de Medicina da Universidade de São Paulo, São Paulo, SP – Brasil; 39 Brasil Sociedade Campineira de Educação e Instrução, Campinas, SP – Brasil; 40 Brasil Universidade Estadual de Campinas, Faculdade de Ciências Médicas, Campinas, SP – Brasil; 41 Brasil Universidade Tiradentes, Aracaju, SE – Brasil; 42 Brasil Organização Social de Saúde VivaRio, Rio de Janeiro, RJ – Brasil; 43 Brasil Instituto Nacional de Cardiologia (INC), Rio de Janeiro, RJ – Brazil; 44 Brasil Secretaria de Vigilância em Saúde, Ministério da Saúde, Brasília, DF – Brasil; 45 Brasil Instituto René Rachou, Fundação Oswaldo Cruz, Belo Horizonte, MG – Brasil; 46 Brasil Hospital Universitário Clementino Fraga Filho, Instituto do Coração Edson Saad – Universidade Federal do Rio de Janeiro, RJ – Brasil; 47 Brasil Universidade Federal da Bahia (UFBA), Salvador, BA – Brasil; 48 Brasil Ambulatório de Doença de Chagas e Insuficiência Cardíaca do Pronto Socorro Cardiológico Universitário da Universidade de Pernambuco (PROCAPE/UPE), Recife, PE – Brasil; 49 Brasil Prefeitura Municipal de Goiânia, Goiânia, GO – Brasil; 50 Brasil Cardion - Cardiologia Preventiva e Avançada, Uberlândia, MG – Brasil; 51 Brasil Hospital Felicio Rocho, Belo Horizonte, MG – Brasil

**Table:** 

SBC Guideline on the Diagnosis and Treatment of Patients with Cardiomyopathy of Chagas Disease – 2023	
The report below lists declarations of interest as reported to the SBC by the experts during the period of the development of these statement, 2021-2023.	
Expert	Type of relationship with industry
Adalberto Menezes Lorga Filho	Nothing to be declared
Adriana de Jesus Benevides de Almeida Guimarães	Nothing to be declared
Adriana Lopes Latado Braga	Nothing to be declared
Adriana Sarmento de Oliveira	Nothing to be declared
Alberto Novaes Ramos Júnior	Nothing to be declared
Alejandro Marcel Hasslocher-Moreno	Nothing to be declared
Alejandro Ostermayer Luquetti	Nothing to be declared
Alvaro Valentim Lima Sarabanda	Nothing to be declared
Ana Yecê das Neves Pinto	Nothing to be declared
Andre Assis Lopes do Carmo	Financial declaration A - Economically relevant payments of any kind made to (i) you, (ii) your spouse/partner or any other person living with you, (iii) any legal person in which any of these is either a direct or indirect controlling owner, business partner, shareholder or participant; any payments received for lectures, lessons, training instruction, compensation, fees paid for participation in advisory boards, investigative boards or other committees, etc. From the brazilian or international pharmaceutical, orthosis, prosthesis, equipment and implants industry: - Biosense Webster; Abbott. Other relationships Funding of continuing medical education activities, including travel, accommodation and registration in conferences and courses, from the brazilian or international pharmaceutical, orthosis, prosthesis, equipment and implants industry: - Biosense Webster; Abbott.
Andre Schmidt	Nothing to be declared
Andréa Rodrigues da Costa	Nothing to be declared
Andréa Silvestre de Sousa	Nothing to be declared
Angelo Amato Vincenzo de Paola	Nothing to be declared
Anis Rassi Junior	Nothing to be declared
Antônio Carlos Sobral Sousa	Nothing to be declared
Antonio Luiz Pinho Ribeiro	Nothing to be declared
Barbara Maria Ianni	Nothing to be declared
Brivaldo Markman Filho	Nothing to be declared
Carlos Eduardo Rochitte	Nothing to be declared
Carolina Thé Macêdo	Nothing to be declared
Charles Mady	Nothing to be declared
Christophe Chevillard	Nothing to be declared
Cláudio Marcelo Bittencourt das Virgens	Financial declaration A - Economically relevant payments of any kind made to (i) you, (ii) your spouse/partner or any other person living with you, (iii) any legal person in which any of these is either a direct or indirect controlling owner, business partner, shareholder or participant; any payments received for lectures, lessons, training instruction, compensation, fees paid for participation in advisory boards, investigative boards or other committees, etc. From the brazilian or international pharmaceutical, orthosis, prosthesis, equipment and implants industry: - Daiichi-Sankyo: Lixiana e Benicar Triplo; Pfizer: Eliquis; Astrazeneca: Forxiga; Novo Nordisk: Ozempic. Other relationships Funding of continuing medical education activities, including travel, accommodation and registration in conferences and courses, from the brazilian or international pharmaceutical, orthosis, prosthesis, equipment and implants industry: - Novo Nordisk: Ozempic; Daiichi-Sankyo: Lixiana e Benicar Triplo.
Cleudson Nery de Castro	Nothing to be declared
Constança Felicia De Paoli de Carvalho Britto	Nothing to be declared
Cristiano Faria Pisani	Financial declaration A - Economically relevant payments of any kind made to (i) you, (ii) your spouse/partner or any other person living with you, (iii) any legal person in which any of these is either a direct or indirect controlling owner, business partner, shareholder or participant; any payments received for lectures, lessons, training instruction, compensation, fees paid for participation in advisory boards, investigative boards or other committees, etc. From the brazilian or international pharmaceutical, orthosis, prosthesis, equipment and implants industry: - Johnson & Jonhson; Biosense Webster.
Dalmo Correia Filho	Nothing to be declared
Daniela do Carmo Rassi	Nothing to be declared
Dário Celestino Sobral Filho	Nothing to be declared
Dilma do Socorro Moraes de Souza	Financial declaration B - Research funding under your direct/personal responsibility (directed to the department or institution) from the brazilian or international pharmaceutical, orthosis, prosthesis, equipment and implants industry: - Novartis: bioscience.
Dirceu Rodrigues de Almeida	Nothing to be declared
Edecio Cunha Neto	Nothing to be declared
Edimar Alcides Bocchi	Nothing to be declared
Evandro Tinoco Mesquita	Financial declaration A - Economically relevant payments of any kind made to (i) you, (ii) your spouse/partner or any other person living with you, (iii) any legal person in which any of these is either a direct or indirect controlling owner, business partner, shareholder or participant; any payments received for lectures, lessons, training instruction, compensation, fees paid for participation in advisory boards, investigative boards or other committees, etc. From the brazilian or international pharmaceutical, orthosis, prosthesis, equipment and implants industry: - AVE; AstraZeneca; Pfizzer; Bayer; Anylan; Boeringer; Novo Nordisk; Norvartis. Other relationships Any economically relevant equity interest in companies in the healthcare or education industry or in any companies competing with or supplying to SBC: - Partner at EC Tinoco. Employment relationship with the Brazilian or international pharmaceutical, orthosis, prosthesis, equipment and implants industry, as well as any employment relationship with health insurance companies or medical audit companies (including part-time jobs) in the year to which your declaration refers: - UnitedHealth Group (UHG). Performance, in the previous year, as a medical auditor for health insurance companies or the like: - Vice President of the Sociedad Interamericana de Cardiología (SIAC).
Felix Jose Alvarez Ramires	Financial declaration A - Economically relevant payments of any kind made to (i) you, (ii) your spouse/partner or any other person living with you, (iii) any legal person in which any of these is either a direct or indirect controlling owner, business partner, shareholder or participant; any payments received for lectures, lessons, training instruction, compensation, fees paid for participation in advisory boards, investigative boards or other committees, etc. From the brazilian or international pharmaceutical, orthosis, prosthesis, equipment and implants industry: - Novartis; Pfizer; AstraZeneca; Amgen.
Fernanda de Souza Nogueira Sardinha Mendes	Nothing to be declared
Fernando Bacal	Other relationships Funding of continuing medical education activities, including travel, accommodation and registration in conferences and courses, from the brazilian or international pharmaceutical, orthosis, prosthesis, equipment and implants industry: - Novartis: heart failure; Pfizer: amyloidosis.
Francisca Tatiana Pereira Gondim	Nothing to be declared
Gilberto Marcelo Sperandio da Silva	Nothing to be declared
Giselle de Lima Peixoto	Nothing to be declared
Gláucia Maria Moraes de Oliveira	Nothing to be declared
Gustavo Glotz de Lima	Financial declaration A - Economically relevant payments of any kind made to (i) you, (ii) your spouse/partner or any other person living with you, (iii) any legal person in which any of these is either a direct or indirect controlling owner, business partner, shareholder or participant; any payments received for lectures, lessons, training instruction, compensation, fees paid for participation in advisory boards, investigative boards or other committees, etc. From the brazilian or international pharmaceutical, orthosis, prosthesis, equipment and implants industry: - Abbott: atrial fibrillation; Bayer: atrial fibrillation; Sankyo: anticoagulation.
Henrique Horta Veloso	Nothing to be declared
Henrique Turin Moreira	Nothing to be declared
Hugo Bellotti Lopes	Other relationships Funding of continuing medical education activities, including travel, accommodation and registration in conferences and courses, from the brazilian or international pharmaceutical, orthosis, prosthesis, equipment and implants industry: - Abbott. Participation in procurement committees for supplies or drugs in health institutions or any similar roles taken: - Bidding of OPME materials by SUS.
Ibraim Masciarelli Francisco Pinto	Other relationships Participation in government-related regulatory authorities or advocacy authorities in cardiology: - Delegate CFM.
João Carlos Pinto Dias	Nothing to be declared
João Marcos Bemfica Barbosa Ferreira	Nothing to be declared
João Paulo Silva-Nunes	Nothing to be declared
Jose Antonio Marin Neto	Nothing to be declared
José Augusto Soares Barreto-Filho	Nothing to be declared
José Francisco Kerr Saraiva	Nothing to be declared
Joseli Lannes Vieira	Nothing to be declared
Joselina Luzia Menezes Oliveira	Nothing to be declared
Luciana Vidal Armaganijan	Nothing to be declared
Luis Cláudio Lemos Correia	Other relationships Participation in government-related regulatory authorities or advocacy authorities in cardiology: - CONASS representative at CONITEC.
Luiz Cláudio Martins	Nothing to be declared
Luiz Henrique Conde Sangenis	Nothing to be declared
Marco Paulo Tomaz Barbosa	Nothing to be declared
Marcos Antonio Almeida-Santos	Nothing to be declared
Marcus Vinicius Simões	Financial declaration B - Research funding under your direct/personal responsibility (directed to the department or institution) from the brazilian or international pharmaceutical, orthosis, prosthesis, equipment and implants industry: - MSD: heart failure; Novartis: hypercholesterolemia; Alnylam: amyloidosis; IONIS: amyloidosis; Behringer: heart failure. Other relationships Funding of continuing medical education activities, including travel, accommodation and registration in conferences and courses, from the brazilian or international pharmaceutical, orthosis, prosthesis, equipment and implants industry: - BMS: cardiomyopathies.
Maria Aparecida Shikanai Yasuda	Nothing to be declared
Maria Carmo Pereira Nunes	Nothing to be declared
Maria da Consolação Vieira Moreira	Nothing to be declared
Maria de Lourdes Higuchi	Nothing to be declared
Maria Rita de Cassia Costa Monteiro	Nothing to be declared
Martino Martinelli Filho	Financial declaration B - Research funding under your direct/personal responsibility (directed to the department or institution) from the brazilian or international pharmaceutical, orthosis, prosthesis, equipment and implants industry: - Impulse Dynamics Inc.
Mauricio Ibrahim Scanavacca	Financial declaration B - Research funding under your direct/personal responsibility (directed to the department or institution) from the brazilian or international pharmaceutical, orthosis, prosthesis, equipment and implants industry: - J&J: Catheter ablation of patients with ventricular tachycardia and Chagas disease; ABBOTT: autonomic denervation of patients with vasovagal syncope; Medtronic: cryoablation of patients with heart failure and reduced LV ejection fraction with atrial fibrillation.
Mauro Felippe Felix Mediano	Nothing to be declared
Mayara Maia Lima	Nothing to be declared
Maykon Tavares de Oliveira	Nothing to be declared
Minna Moreira Dias Romano	Nothing to be declared
Nadjar Nitz Silva Lociks de Araujo	Nothing to be declared
Paulo de Tarso Jorge Medeiros	Nothing to be declared
Renato Vieira Alves	Nothing to be declared
Ricardo Alkmim Teixeira	Financial declaration A - Economically relevant payments of any kind made to (i) you, (ii) your spouse/partner or any other person living with you, (iii) any legal person in which any of these is either a direct or indirect controlling owner, business partner, shareholder or participant; any payments received for lectures, lessons, training instruction, compensation, fees paid for participation in advisory boards, investigative boards or other committees, etc. From the brazilian or international pharmaceutical, orthosis, prosthesis, equipment and implants industry: - Boehringer-Ingelheim: Pradaxa, Jardiance; Daiichi-Sankyo: Lixiana; Abbott: Implantable Electronic Heart Devices; Biotronik: Implantable Electronic Heart Devices; Medtronic: Implantable Electronic Heart Devices; Biomedical: Lead Extraction from Implantable Electronic Cardiac Devices. Other relationships Funding of continuing medical education activities, including travel, accommodation and registration in conferences and courses, from the brazilian or international pharmaceutical, orthosis, prosthesis, equipment and implants industry: - Biomedical: Biomedical: Lead Extraction from Implantable Electronic Cardiac Devices.
Roberto Coury Pedrosa	Nothing to be declared
Roberto Magalhães Saraiva	Nothing to be declared
Roque Aras Junior	Financial declaration B - Research funding under your direct/personal responsibility (directed to the department or institution) from the brazilian or international pharmaceutical, orthosis, prosthesis, equipment and implants industry: - Novartis: Sacubitril.
Rosalia Morais Torres	Nothing to be declared
Rui Manuel dos Santos Povoa	Other relationships Funding of continuing medical education activities, including travel, accommodation and registration in conferences and courses, from the brazilian or international pharmaceutical, orthosis, prosthesis, equipment and implants industry: - Servier: Perindopril.
Sergio Gabriel Rassi	Nothing to be declared
Silvia Marinho Martins Alves	Other relationships Funding of continuing medical education activities, including travel, accommodation and registration in conferences and courses, from the brazilian or international pharmaceutical, orthosis, prosthesis, equipment and implants industry: - Novartis: Entresto.
Suelene Brito do Nascimento Tavares	Nothing to be declared
Swamy Lima Palmeira	Nothing to be declared
Telêmaco Luiz da Silva Júnior	Nothing to be declared
Thiago da Rocha Rodrigues	Nothing to be declared
Vagner Madrini Junior	Nothing to be declared
Veruska Maia da Costa Brant	Nothing to be declared
Walderez Ornelas Dutra	Nothing to be declared
Wilson Alves de Oliveira Junior	Financial declaration B - Research funding under your direct/personal responsibility (directed to the department or institution) from the brazilian or international pharmaceutical, orthosis, prosthesis, equipment and implants industry: - Entresto/Novartis: heart failure in Chronic Chagas Disease.

## List of Abbreviations

^123^I-MIBG – iodine-123-metaiodobenzylguanidine

^18^F-FDG – 18F-fluorodeoxyglucose+

ACEI – angiotensin-converting-enzyme inhibitor

ACLS – Advanced Cardiovascular Life Support

AF – atrial fibrillation

AIDS – acquired immunodeficiency syndrome

ANVISA – Brazilian Health Surveillance Agency

ARB – angiotensin II receptor blocker

ATP – adenosine triphosphate

AVB – atrioventricular block

BNP – brain natriuretic peptide

CCCD – chronic cardiomyopathy of Chagas disease

CCD – cardiomyopathy of Chagas disease

CD – Chagas disease

CI – confidence interval

CLIA – chemiluminescence immunoassay

CMIA – chemiluminescent microparticle immunoassay

CMRI – cardiac magnetic resonance imaging

CONITEC – National Commission of Technology Incorporation in the SUS

COVID-19 – disease caused by the new coronavirus

CRT – cardiac resynchronization therapy

CTI – cardiothoracic index

CTX – cardiac transplantation

CVP – central venous pressure

DCM – dilated cardiomyopathy

DTU – discrete typing units

EBM – Evidence-Based Medicine

ECG – electrocardiogram

ECHO – echocardiography

ECLIA – electrochemiluminescence immunoassay

ELISA – enzyme-linked immunosorbent assay

EPS – electrophysiological study

FDA – Food and Drug Administration

FINDECHAGAS – International Federation of Associations of Individuals With Chagas Disease

FIOCRUZ – *Fundação Oswaldo Cruz*

G-CFS – granulocyte-colony stimulating factor

GLS – global longitudinal strain

GRADE – Grading of Recommendations, Assessment, Development, and Evaluations

GWAS – Genome Wide Association Study

HF – heart failure

HFmrEF – heart failure with mildly reduced ejection fraction

HFrEF – heart failure with reduced ejection fraction

HIV – human immunodeficiency virus

HR – heart rate

HRV – heart rate variability

ICD – implantable cardioverter-defibrillator

ICM – ischemic cardiomyopathy

IDI – integrated discrimination index

IFCD – indeterminate form of Chagas disease

IFN-γ – interferon-gamma

IHA – indirect hemagglutination

IIF – indirect immunofluorescence

IL – interleukin

INR – international normalized ratio

INSS – National Institute of Social Security (in Portuguese, *Instituto Nacional do Seguro Social* )

IPEC-FIOCRUZ – *Instituto de Pesquisa Evandro Chagas-Fundação Oswaldo Cruz*

ISHLT – International Society for Heart & Lung Transplantation

LACEN – Public Health Central Laboratories

LAFB – left anterior fascicular block

LAVA – local abnormal ventricular activities

LBBB – left bundle branch block

LV – left ventricular

LVDD – left ventricular diastolic diameter

LVEF – left ventricular ejection fraction

MCSD – mechanical circulatory support device

MESH – Medical Subject Headings

miRNA – microRNA

MMF – mycophenolate mofetil

MRI – magnetic resonance imaging

mTOR – mechanistic target of rapamycin

NK – natural killers

NNT – number needed to treat

NRI – net reclassification index

NSVT – nonsustained ventricular tachycardia

NTD – neglected tropical diseases

NT-proBNP – N-terminal pro-brain natriuretic peptide

NYHA – *New York Heart Association*

PAHO – Panamerican Health Organization

PCDT – Clinical Protocol and Therapeutic Guidelines

PCP – pulmonary capillary pressure

PCR – polymerase chain reaction

PET/CT – positron emission tomography computed tomography

PHC – primary health care

PM – pacemaker

PRA – panel reactive antibody

qPCR – real time or quantitative PCR

RBBB – right bundle branch block

RCD – reactivation of Chagas disease

RCT – randomized clinical trial

RV – right ventricular

SAH – systemic arterial hypertension

SARS-CoV-2 – severe acute respiratory syndrome coronavirus 2

SBC – Brazilian Society of Cardiology (in Portuguese, *Sociedade Brasileira de Cardiologia* )

SDG – Sustainable Development Goals

SGLT2 – sodium-glucose cotransporter-2

SINAN – Notifiable Diseases Information System (in Portuguese, *Sistema de Informação de Agravos de Notificação* )

SNP – single nucleotide polymorphism

SPECT-CT – single photon emission computed tomography and computed tomography

SSFP – steady-state free precession

STE – speckle tracking echocardiography

SUS – Brazilian Unified Health System (in Portuguese, *Sistema Único de Saúde* )

SVT – sustained ventricular tachycardia

*T. cruzi* – *Trypanosoma cruzi*

TAPSE – tricuspid annular plane systolic excursion

TAVB – total atrioventricular block

TIA – transient ischemic attack

TNF-α – tumor necrosis factor alpha

Tregs – regulatory T cells

UBS – Basic Healthcare Units

UPAE – specialized emergency care units

USA – United States of America

VE – ventricular extrasystole

VF – ventricular fibrillation

VT – ventricular tachycardia

WHO – World Health Organization

## Table of Contents

1. Initial Considerations 12

**1.1. Methodology Used to Elaborate this Guideline** 14

**1.2. Scientific Rationale for Recommendations of Diagnostic Methods** 15

2. Epidemiology – 21st Century Update 16

**2.1. Introduction** 16

**2.2. World Distribution of Chagas Disease** 18

**2.3. Overview of Chagas Disease in Brazil** 19

**2.4. Epidemiological Surveillance in Brazil** 21

**2.5. Association of Chagas Disease with COVID-19** 21

**2.6. Final Reflection on the Current Chagas Disease Epidemiological Scenario** 23

3. Pathogenesis of Cardiomyopathy of Chagas Disease 23

**3.1. Introduction** 23

**3.2. Immune Dynamics and Differential Progression to Chronic Cardiomyopathy of Chagas Disease** 24

**3.3. Myocardial Mitochondrial Dysfunction and Chronic Cardiomyopathy of Chagas Disease** 25

**3.4. Genetics in Chronic Cardiomyopathy of Chagas Disease** 26

**3.5. Coronary Microvascular Disorder** 26

**3.6. Cardiac Denervation** 27

**3.7. Final Considerations** 28

4. Cardiomyopathy Pathophysiology – Acute and Chronic Phases 28

**4.1. Introduction** 28

**4.2. Myocardial Parasitism and Immune Response** 29

**4.2.1. Immune response in the acute phase** 29

**4.2.2. Immune response in the chronic phase** 29

4.3. Autonomic Nervous System Alterations in Chagas Disease: Evidence from Histopathological Studies 29

4.4. Chagas Disease Pathophysiology Dependent on the Parasite and Human Host Genetic Characteristics 30

4.5. Peculiar Histopathology of Chagas Disease 31

4.6. Coronary Microcirculation Injury 32

4.7. Potential Therapeutic Applications of Pathophysiological Targets in Chronic Cardiomyopathy of Chagas Disease 32

5. Natural History 33

5.1. Acute Myocarditis of Chagas Disease 33

5.2. The Indeterminate Form and Clinical Syndromes of Chronic Cardiomyopathy of Chagas Disease 34

**5.2.1. Natural History of the Chronic Phase of Chagas Disease** 34

**5.2.2. Indeterminate Form of Chagas Disease: Importance of the Concept and Abnormalities on More Sophisticated Additional Tests** 34

**5.2.3. Progression to Chronic Cardiomyopathy** 35

**5.2.4. Clinical Forms of Chronic Cardiomyopathy of Chagas Disease** 36

**5.2.4.1. Abnormalities on Complementary Tests** 36

**5.2.4.2. Cardiac Arrhythmias** 36

**5.2.4.3. Heart Failure Syndrome** 37

**5.2.4.4. Systemic and Pulmonary Thromboembolic Syndrome** 37

6. Diagnosis of Cardiomyopathy of Chagas Disease 38

6.1. Methods to Determine the *T. cruzi* Infection 38

**6.1.1. Introduction** 38

**6.1.2. Serological Tests Available and Which to Request** 38

**6.1.3. Interpretation of the Results** 38

**6.1.4. Special Situations** 39

**6.1.4.1. Inconclusive Serological Results** 39

**6.1.4.2. Test Result Not Corresponding to Clinical Expectation** 39

**6.1.4.3. Parasitemia** 39

**6.1.4.4. Negative Serology in Patients in the Chronic Phase** 39

**6.1.4.5. Spontaneous Cure** 39

**6.1.4.6. Acute Phase Diagnosis** 39

**6.1.4.7. Blood Services** 40

**6.1.4.8. Congenital transmission** 41

**6.1.4.9. Serology of Infected Individuals Treated with Chemotherapy Drugs** 41

**6.1.4.10. Rapid Serological Tests** 41

**6.1.4.11. Parasitological Tests** 41

**6.1.4.11.1. Indications for Parasitological Tests, particularly Polymerase Chain Reaction** 41

**6.1.4.11.2. Interpretation of the Results of Parasitological Tests** 42

**6.1.4.12. Polymerase Chain Reaction** 42

**6.1.4.13. Operational Procedures for PCR Use** 42

6.2. Diagnostic Methods for Structural and Functional Cardiac Abnormalities 43

**6.2.1. Electrocardiogram in Chagas Disease** 43

**6.2.2. Chest Radiography** 43

**6.2.3. Echocardiography** 45

**6.2.3.1. Left Ventricular Systolic Function** 45

**6.2.3.2. Segmental Changes in Ventricular Contractility** 46

**6.2.3.3. Left Ventricular Diastolic Function** 46

**6.2.3.4. Right Ventricular Assessment** 46

**6.2.3.5. Stress Echocardiography** 46

**6.2.4. Cardiac Magnetic Resonance Imaging** 46

**6.2.5. Nuclear Medicine** 47

**6.2.5.1. Radionuclide Ventriculography** 47

**6.2.5.2. Myocardial Perfusion** 47

**6.2.5.3. Sympathetic Innervation Assessment** 47

**6.2.6. Computed Tomography of Coronary Arteries** 48

**6.2.7. Ambulatory Electrocardiography (Holter)** 48

**6.2.8. Intracardiac Electrophysiological Study** 48

**6.2.9. Exercise and Cardiopulmonary Tests** 48

**6.2.10. Cardiac Catheterization** 49

7. Risk Stratification and Prognosis 49

8. Therapeutic Management in the Indeterminate Form of Chagas Disease 54

9. Etiological Treatment of Chagas Disease 56

9.1. Introduction 56

9.2. Drugs and Administration 57

9.3. Etiological Treatment of Individuals with Chagas Disease 60

9.4. Acute Infection 60

9.5. Congenital Infection 61

9.6. Children and Adolescents with Chronic Infection 61

9.7. Women of Reproductive Age with Chronic Infection 61

9.8. Adults with Chronic Infection 62

9.9. Reactivation of Chagas Disease 63

9.10. Accidental Infection 64

9.11. Assessment of Chagas Disease Cure after Etiological Treatment 64

**9.11.1. Where to Treat an Individual with Chagas Disease** 65

10. Therapeutic Management of Ventricular Dysfunction and Heart Failure 66

10.1. Pharmacological Resources 66

**10.1.1. Heart Failure Classification** 66

**10.1.2. Maximum Dosage of Medications** 66

**10.1.3. The Contemporary Patient** 66

**10.1.4. Literature Review** 66

**10.1.5. Pharmacological Therapy** 66

**10.1.5.1. Diuretics** 66

**10.1.5.2. Renin-Angiotensin-Aldosterone System Inhibitors** 67

**10.1.5.3. Beta-blockers** 67

**10.1.5.4. Spironolactone** 68

**10.1.5.5. Ivabradine** 68

**10.1.5.6. Digoxin** 68

**10.1.5.7. Sacubitril-valsartan** 68

**10.1.5.8. Sodium-Glucose Cotransporter-2 Inhibitors** 69

10.2. Non-Pharmacological Resources 71

**10.2.1. Cardiac Transplantatio** n 71

**10.2.1.1. Immunosuppression Strategies** 73

**10.2.1.2. Induction Therapy** 73

**10.2.1.3. Maintenance Therapy** 73

**10.2.2. Diagnosis and Treatment of Rejection** 73

**10.2.3. Diagnosis and Treatment of T. cruzi-Infection Reactivation** 74

**10.2.3.1. Clinical Presentation** 74

**10.2.3.2. Parasitological Diagnosis of Reactivatio** n 74

**10.2.3.3. Etiological Treatment of Reactivation** 74

**10.2.3.4. Post-Heart Transplant Complications and Survival** 75

**10.2.4. Mechanical Circulatory Support** 75

11. Therapeutic Management of Cardiac Arrhythmias 76

11.1. Pharmacological Resources 76

**11.1.1. Introduction** 76

**11.1.2. Sudden Death Prevention with Non-Antiarrhythmic Drug** s 76

**11.1.3. Ventricular Arrhythmias in Cardiopathies of Other Etiologies** 77

**11.1.4. Amiodarone for Patients with Cardiopathies of Other Etiologies: Primary Prevention** 77

**11.1.5. Amiodarone for Patients with Cardiopathies of Other Etiologies: Secondary Prevention** 79

**11.1.6. Ventricular Arrhythmias in Patients with Chronic Cardiomyopathy of Chagas Disease: Characteristics and Treatment** 80

**11.1.6.1. Ventricular Extrasystoles** 80

**11.1.6.2. Nonsustained Ventricular Tachycardia** 80

**11.1.6.3. Sustained Ventricular Tachycardia and Ventricular Fibrillation** 80

**11.1.7. Care During the Use of Amiodarone** 82

**11.1.8. Prevention of Electrical Shock Recurrence in Patients Treated with Implantable Cardioverter-Defibrillator** 82

**11.1.9. Pharmacological Treatment of Atrial Fibrillation in Chronic Cardiomyopathy of Chagas Disease** 83

**11.1.10. Treatment in the Emergency Room** 83

**11.1.11. Outpatient Treatment** 83

**11.1.11.1. Reversion to Sinus Rhythm** 83

**11.1.11.2. Heart Rate Control** 83

11.2. Pacemaker, Cardioverter-Defibrillator, and Cardiac Resynchronization Therapy 85

**11.2.1. Artificial Cardiac Pacemaker** 85

**11.2.2. Implantable Cardioverter-Defibrillator in CCCD** 85

**11.2.2.1. Primary Prevention of Sudden Cardiac Death** 85

**11.2.2.2. Secondary Prevention of Sudden Cardiac Death** 87

**11.2.3. Cardiac Resynchronization Therapy** 89

11.3. Ablation Methods 90

**11.3.1. Sustained Ventricular Tachycardia: Clinical Presentation, Electrophysiological Mechanisms, and Sites of Origin** 90

**11.3.2. Clinical and Laboratory Pre-Ablation Assessment** 90

**11.3.3. Mapping Techniques for Ventricular Tachycardias** 92

**11.3.4. Outcomes and Complications During Ventricular Tachycardia Ablation** 93

**11.3.5. Ablation Results and Patients’ Follow-up** 93

12. Managements to Prevent and Treat Thromboembolic Complications 93

12.1. Introduction 93

12.2. Epidemiology of Thromboembolic Events 94

12.3. Risk Factors and Mortality 94

12.4. Risk Assessment of Stroke 95

12.5. Clinical Findings and Diagnostic Investigation of Ischemic Stroke in Chagas Disease 96

12.6. Treatment of Ischemic Stroke in Chagas Disease 97

12.7. Prevention of Cardioembolic Events in Chagas Disease 98

13. Management in Special Subgroups and Handling of Issues Related to T. cruzi-HIV Coinfection, Pregnancy, Physical Activity, Surgical Risk, General Anesthesia, and COVID-19 100

13.1. T. cruzi-HIV Coinfection 100

13.2. Seropositivity in Potential Donors at Blood Banks 101

13.3. Physical Activity 101

13.4. Pregnant Women 102

13.5. Newborns 103

13.6. Surgical and Anesthetic Risk 104

13.7. Chagas Disease and Coronavirus Infection 104

13.8. Noncardiac Transplantation and Immunosuppressive Therapy 105

**13.8.1. Donor with Chagas Disease and Recipient without Chagas Disease** 105

**13.8.2. Recipient with Chagas Disease** 106

**13.8.3. Autoimmune Diseases** 107

13.9. Chagas Disease and Aging 107

14. Recommendations for Implantation of Structured Health Services for the Follow-Up of Individuals with Chronic Cardiomyopathy of Chagas Disease 107

14.1. Assignments of Structured Health Services for the Follow-up of Individuals with Chronic Cardiomyopathy of Chagas Disease 108

14.2. Expected Benefits of Structured Health Services for the Follow-up of Individuals with Chronic Cardiomyopathy of Chagas Disease 109

15. Definition of Severe Cardiopathy and Medico-Legal Assessment 110

15.1. Introduction 110

15.2. Concept and Scope 110

15.3. Score to Predict the Risk of Death in Patients with Chronic Cardiomyopathy of Chagas Disease 110

15.4. Clinical Aspects 111

15.5. Medico-Legal Expert Assessment 111

15.6. Conclusion 111

Acknowledgements 111

References 112

## 1. Initial Considerations

In 2021, the then president of the Brazilian Society of Cardiology (SBC), Dr. Marcelo Queiroga Cartaxo Lopes, commissioned us to coordinate the elaboration of the new guideline on Chagas disease (CD). The undertaking was justified because since 2011 the SBC had not directly been responsible for a guideline on that subject. Differently from the guideline published more than one decade ago in the *Arquivos Brasileiros de Cardiologia* ,^1^the current one would no longer be “Latin American” but would count essentially on an expressive number of Brazilian collaborators. The summoned group of notorious active researchers in the field would represent a comprehensive team of professionals from different parts of Brazil, involved in and directly contributing to the advance of the fight against CD. These researchers are responsible for this guideline authorship, as explained below.

In addition, considering that in 2015 we collaborated extensively with the Brazilian Society of Tropical Medicine guideline on the general context of CD,^2^the scope of the current one was limited only to aspects related to the diagnosis and treatment of the most frequent and severe manifestation of CD, the cardiomyopathy of Chagas disease (CCD).

Despite the large number of documents on the theme in its varied aspects ( Chart 1.1 ),^1 - 22^discrepancies exist mainly regarding the strength of the recommendations and levels of evidence related to the different types of treatment, as well as the appearance of new scientific evidence, corroborating the understanding that guidelines need to be periodically reviewed and updated.

**Chart 1.1:** – Consensus, guidelines, and relevant documents approaching patients with Chagas disease.

TITLE OF THE DOCUMENT	THEME	PUBLICATION YEAR	RESPONSIBLE
“Consenso Brasileiro em Doença de Chagas” ^3^	general	2005	Brazilian Ministry of Health
“Diagnosis, Management and Treatment of Chronic Chagas' Heart Disease in Areas Where *Trypanosoma cruzi* infection is not Endemic” ^4^	general	2007	SEMTSI
“Evaluation and Treatment of Chagas Disease in the United States: A Systematic Review” ^5^	general	2007	CDC and panel of experts on Chagas disease
“I Diretriz Latino Americana para o Diagnóstico e Tratamento da Cardiopatia Chagásica” ^1^	general	2011	SBC
“Consenso de Enfermedad de Chagas-Mazza” ^6^	general	2011	SAC
“II Consenso Brasileiro em Chagas disease” ^2^	general	2015	Brazilian Ministry of Health
“Chagas Cardiomyopathy: An Update of Current Clinical Knowledge and Management: A Scientific Statement From the American Heart Association” ^7^	general	2018	AHA
“Protocolo Clínico e Diretrizes Terapêuticas (PCDT) da Doença de Chagas” ^8^	general	2018	CONITEC
“Acuerdo Regional de los Expertos en Chagas de las Sociedades de Cardiología Sudamericanas” ^9^	general	2018	SSC
“Guía para el diagnosis y el Tratamiento de la Enfermedad de Chagas” ^10^	general	2018	PAHO
“Consenso Enfermedad de Chagas 2019” ^11^	general	2020	SAC
“Consenso do Comitê de Eletrofisiologia da USCAS sobre o Tratamento das Arritmias Ventriculares na Doença de Chagas” ^12^	arrhythmia	2002	SBC
“Diretrizes Brasileiras de Dispositivos Cardíacos Eletrônicos Implantáveis (DCEI)” ^13^	arrhythmia	2007	SBC
“Consenso de Prevención Primaria y Secundaria de Muerte Súbita” ^14^	arrhythmia	2012	SAC/SUC
“Diretrizes Brasileiras de Dispositivos Cardíacos Eletrônicos Implantáveis” ^15^	arrhythmia	2015	DECA/SBCCV
“II Diretrizes da Sociedade Brasileira de Cardiologia para o Diagnóstico e Tratamento da Insuficiência Cardíaca” ^16^	HF	1998	SBC
“Revisão das II Diretrizes da Sociedade Brasileira de Cardiologia para o Diagnóstico e Tratamento da Insuficiência Cardíaca” ^17^	HF	2002	SBC
“III Diretriz Brasileira de Insuficiência Cardíaca Crônica” ^18^	HF	2009	SBC
“Atualização da Diretriz Brasileira de Insuficiência Cardíaca” ^19^	HF	2012	SBC
“3ª Diretriz Brasileira de Transplante Cardíaco” ^20^	HF	2018	SBC
“Diretriz Brasileira de Insuficiência Cardíaca Crônica e Aguda” ^21^	HF	2018	SBC
“Consenso de Enfermedad de Chagas. Insuficiencia cardíaca en miocardiopatía chagásica crónica” ^22^	HF	2019	FAC

AHA: American Heart Association; CDC: US Centers for Disease Control and Prevention; CONITEC: Brazilian National Commission of Technology Incorporation in the SUS; DECA/SBCCV: Department of Artificial Cardiac Stimulation of the Brazilian Society of Cardiovascular Surgery; FAC: Federación Argentina de Cardiología; HF: heart failure; PAHO: Pan American Health Organization; SAC: Argentinian Society of Cardiology; SBC: Brazilian Society of Cardiology; SEMTSI: Sociedad Española de Medicina Tropical y Salud Internacional; SSC: Sociedad Sudamericana de Cardiología; SUC: Sociedad Uruguaya de Cardiología.

This guideline, particularly its usual framework naturally directed to the formulation of rules of conduct and scientific evidence that support the countless aspects of CCD diagnosis and treatment, has some characteristics conferred by the temporal context of its elaboration. In fact, we lived the distressful circumstance of adding to CCD, a markedly inflammatory illness, the problems from the disease caused by the new coronavirus (COVID-19) pandemic, with its own inflammation component. Thus, the scientific community, both worldwide and specially in Brazil, had to face at least three big obstacles to control the pandemic: first, it is a special virus, with peculiar behavior regarding the injury to the host’s organs; second, there were inherent and unpredictable difficulties regarding the epidemiological behavior of that virus; third, our national indigence, when we verify that, to overcome the pandemic, the appropriate measures bump into basic facts, such as the very precarious sanitary conditions of 30-40% of our population, lacking sewage, piped water, and minimally appropriate housing.

Denialist attitudes and dissemination of fake concepts, including by part of the medical community, represent an incremental obstacle to the performance of Science and Medicine in the fight against the pandemic.^23^To that set of challenges and obstacles, the Brazilian scientific community responded with notable readiness and efficiency, as exemplified by the large-scale development and application of vaccines against COVID-19. It is worth noting that the difficulties faced to widen the protection against contamination and to implement population vaccination were remains from the wars fought during the 20th century against the deleterious influence of the tobacco industry, which for a long time tried to hide the tobacco’s harms.^24^It is worth noting that some aspects of the concomitance of the infections by *Trypanosoma cruzi* ( *T. cruzi)* and by coronavirus in the same individual are properly addressed in specific topics of this guideline.

Thus, the sanitary conquests in fighting the pandemic in the 21st century achieved by the scientific community, well represented by FIOCRUZ, a historical heir of its first and unsurpassed epigone, Oswaldo Cruz, are reminders of his success with the vaccination campaigns against yellow fever in the beginning of the 20^th^century. In addition, a parallel can be drawn between the current scientific and medical community situation in the fight against the COVID-19 pandemic and the difficult context experienced by Carlos Chagas and his mentor Oswaldo Cruz during the first decades of the 20^th^century.

Similarly to today’s denialism, that great Brazilian, despite his scientifically epic discovery, had to confront the nihilism and misunderstanding of a considerable part of the medical community regarding his deeds in the history of Medicine, according to professor João Carlos Pinto Dias, and son of his direct collaborator, Emmanuel Dias, and also participating in this guideline. In addition, the early sudden death of Carlos Chagas might have been triggered emotionally, consequent to the obscurantist attack.

As it has been reported, “It is plausible that his great humanistic perspicacity had provided him with the vision of the tragically real social meaning of the disease he had just discovered, affecting literally millions of vulnerable individuals in large areas of Brazil. In contrast to the denial by part of the academic community to accept the existence of the new disease, Carlos Chagas might have envisioned the national tragedy of his discovery, which still unfolds in multiple socially deplorable acts and chapters”.^25^

It will never be too much to glorify Carlos Chagas’ memory. According to Alejandro Hasslocher-Moreno, another collaborator of this guideline, “Carlos Chagas was the right physician and scientist, at the right time, in the right place. The circumstances involving the disease’s discovery had as protagonist an individual prepared to face a known challenge and, at the same time, to discover the unknown. In the biomedical context, the Brazilian science was boosted after the discovery of CD, gaining international recognition, one of the major legacies of Carlos Chagas to the Brazilian Science and Medicine”.^26^

When revisiting and elaborating guidelines, it is justifiable to pay tribute to the emeritus physicians and scientists who either had left us right before the publication of the 2011 guideline or now, when we are finishing the 2022 guideline. We want to honor all of them for their legacy in CD research, and especially professors Joaquim Romeu Cançado 1913-2011 (Belo Horizonte), Aluízio Rosa Prata 1920-2011 (Uberaba), Zilton Araújo Andrade 1924-2020 (Salvador), José Rodrigues Coura 1927-2021 (Rio de Janeiro), and Anis Rassi 1929-2021 (Goiânia). To them we devote our gratitude and recognition. Their influence on this guideline kept us in the luminous scientific pathway trod by Carlos Chagas.

The authors, collaborators and coordinators of this document are aware that, in this phase of intensified perception of CD as a neglected disease, it is mandatory to rescue the affected individuals from their miserable conditions and their deplorable medical-social implications. Thus, we should strive to minimize the stigma of CD, beginning by banning the term “Chagasic” from this guideline. This is based on the recent understanding that instead of being an eponym to honor the historical trajectory of Carlos Chagas, that term, for some patients, would sound as if they had a true and painful incurable wound in their hearts.

Involved in the elaboration of this guideline, we are clearly aware that our responsibility has greatly increased recently. In addition to being mainly directed to physicians and paramedics, the principles herein contained should be useful to guide managers and agencies in charge of providing proper public health conditions nationally. And last but not least, the individuals currently affected by the disease are much more in need for safe guidance by health professionals than in the past. The number of individuals with CD increased in parallel with the democratization of the information provided in the web. Health professionals must provide better instructions on how to manage and decrease the problems deriving from CD.

We will use the introduction of this new guideline to describe the process that culminated in this document. Right from the beginning we realized that the timeline initially planned to be concluded in a few months did not correspond to our ambition to build a reflexive, scientifically deep document, with consistent clinical and population implications.

The starting point was the meeting where the coordinators discussed the scientific principles to guide the elaboration of the topics, introducing the idea that the knowledge of experts would be essential to interpret and judge the applicability of the evidence, but not to foster opinions based on individual preferences. Disagreements would be solved by deep analysis of evidence, not by majority vote. At that time, we planted the seed of a guideline to challenge our own intuitions and recognized that often the truth antagonizes our expectations, requiring the cultivation of doubt, which usually contrasts with the eloquence of a group of opinion makers in their respective areas. This was the seed of a guideline built based on intense debates, creative attrition, and learning for all, totaling seven virtual meetings and 28 hours of debates.

From conception to the last subject presented in this guideline, we tried to follow the most legit principles of the classic paradigm of Evidence-Based Medicine (EBM). Even though a comprehensive systematic review of the literature could not be performed, in some more polemic points we analyzed the evidence that should answer the so-called PICO question, which comprises the characteristics of the population (”P”), intervention (“I”), comparing control (“C”), and outcome (“O”).^27^We hope we were able to ‘rescue’ at least most of the recommendations and the analyses of evidence that support them from the bias and other management deviations identified in previous contexts. We are sure that, unfortunately, the paradigm of EBM itself is currently abused and distorted, paradoxically amid the exponential multiplication of research and knowledge generated and disclosed without proportional control by entities that should supervise the entire process of advances in such a noble area of human activity. One example of such distortions, fortunately not observed in the context of CD, but very clear in some areas of Medicine, is the profusion of inappropriate, inconsistent, routine, or redundant meta-analyses, resulting in “fake news”, as already suggested.^28^

Thus, we retrieve and emphasize the essential principle of the last section of the SBC 2011 guideline,^1^which briefly is “Cardiologists should improve their patients’ clinical management, wisely administering medications and interventions that respect as much as possible the peculiar pathophysiology of the disease, neither appealing to measures without proof of benefit nor wasting plausible therapeutic opportunities”.

### 1.1. Methodology Used to Elaborate this Guideline

The construction of a guideline for medical management is an underppreciated opportunity of reflection on the rationale that enables the translation from the scientific paradigm to clinical decision. This type of reflection can develop strategies to reduce the gap between evidence and recommendation.

Scientific evidence has two major functions: the epistemological function (counterfactual), relative to the construction of knowledge of causality, and the pragmatic function, which influences the decision-making process (consequentialist). In the first function, evidence of an exploratory character, whose quality is satisfactory, is valued by suggesting the scientific pathway. In the second function, the use of evidence with a high risk for bias or statistical imprecision servs more to justify the desire to act rather than to increase the likelihood that the action represents the best choice for the patient or population. It is the intuitive desire searching for scientific justification.

Guidelines usually make clinical recommendations based on the quality of the evidence found after careful search. Several systems have been proposed to classify evidence and to categorize the “strength” of the clinical recommendation, such as GRADE, CEBM, SIGN, NZGG, SORT, USPSTF, ACCF/AHA/ESC, ACCP, IDSA, and NICE.^29^The strength of the recommendation is usually related to the level of evidence. For this guideline, we adopted a simple classification based on the GRADE ( *Grading of Recommendations, Assessment, Development, and Evaluations* ) approach,^27^but with some modifications, grouping the studies into only 3 levels of quality of evidence (A = high level; B=moderate level; and C = low level), from which 2 grades of recommendation derive (1 = strong; and 2 = conditional). The starting point in assessing the quality of the evidence should be the delineation of the research. Evidence originating from analytical experimental studies, such as randomized clinical trials (RCTs), and systematic reviews with meta-analyses of those studies are less prone to biases, being, consequently, considered of better quality or high level (A). In contrast, evidence originating from analytical observational studies (case-control, cross-sectional, and cohort) are considered of moderate level (B), and those originating from descriptive observational studies (without a comparing group), such as case series, are considered of low quality or low level (C).

In the specific case of CCD, because we do not usually have high quality evidence deriving from RCTs and, in some situations, not even from observational studies with convincing results to generate endorsed recommendations, there is a natural tendency to resort to free “expert opinion” or “consensus”, abstract words of uncertain significance and consequences, which should not be formally characterized as evidence.^30^One solution for this question is the recognition of the value of indirect evidence, originating from results of RCTs performed on other cardiopathies, and the understanding of the difference between representative sample and generalizable sample.^27^

In observational descriptive studies, the sample representativeness is essential. For example, regarding the prognosis of a patient with heart failure (HF), what is observed in ischemic cardiomyopathy (ICM) might not apply to CCD. However, in analytical, observational, or experimental (of causality) studies, a nonrepresentative sample can become generalizable. For generalization to be justified, lack of interaction (effect modification) between the populations’ differences and the effect of a risk factor or a medical management is required.

Because biological interaction is a rare phenomenon, nonrepresentative samples generate generalizable concepts for different types of patients. This justifies a large part of the recommendations for elderly and children, subgroups usually not properly represented in RCTs. The prescription, for example, of an angiotensin-converting-enzyme inhibitor (ACEI) for a patient with CCD and reduced left ventricular ejection fraction (LVEF) is not based on preference or use of evidence of low quality in that type of population. It is based on evidence of high quality in patients with HF of other etiologies, in accordance with the perception that “effect modification” by the cardiomyopathy etiology (interaction) is unlikely. This is how scientific knowledge is built. For example, the theory supporting the knowledge that the speed of light is constant did not derive from its measure in all environments and circumstances. Only a few measures, in accordance with the notion of low probability of interaction between the environment and speed of light, enable us to generalize that the speed of light is, in fact, constant.

When making generalization, we should question if there is a characteristic in the population of interest that would change the study’s result. For example, is there any characteristic of the patient with CCD that can modify the beneficial effect (interaction) of the vasodilating therapy, which was confirmed in ICM or dilated cardiomyopathy (CDM)? Probably not.

In the absence of direct experimental evidence, which is obtained from the results of RCTs performed on CCD (level A), and of indirect evidence, which is obtained from extrapolation of the results of RCTs performed on other cardiopathies (level B), we chose to value the results obtained from observational analytical studies (level B) or from observational descriptive studies (level C), both performed on CCD, in addition to adopting the principle of extreme plausibility and the principle of asymmetry as level C of evidence.

It is worth noting that decision-making that dispense empirical evidence is common in Medicine. In the essential absence of *equipoise* , decisions are not made based on experimental data, but on natural data. There are situations that do not require “judgement” (mental measurement of the likelihoods) and it would be unethical to perform an experiment with a control group. One example is the use of diuretic in HF with marked congestion, whose benefit has never been specifically measured in a placebo-controlled clinical trial, because of its almost deterministic character. If it were, we would have a number needed to treat (NNT) of 1 for symptom improvement and a possibly very relevant NNT for reduction of mortality.

By failing to understand that statement, the medical community places diuretics at a lower level of benefit because of the lack of experimental proof of mortality reduction. Therefore, in the presence of HF with systemic and/or pulmonary congestion, the careful prescription of a diuretic should be considered based on essential evidence, leading to its strong recommendation. For situations like that, the parachute analogy can be used as a strategy to reduce mortality of individuals in free fall.^31^This is another circumstance in which the level C of evidence should be applied: absence of experimental evidence, but strong natural evidence. This should be emphatically differentiated from the paradigm of preference, contained in “consensus”, because the evidence regarding the use of parachute requires no consensus. It is indisputable.

Another principle that will be used as level C of evidence is that of asymmetry of effect, which can be applied to situations in which, despite the lack of proof of the efficacy of a certain intervention, there is great asymmetry between the magnitude of a potential benefit and the magnitude of an occasional harm, such as the use of masks to control COVID-19 and the etiological treatment in middle-aged adults with the indeterminate form of CD (IFCD).

Once the situations of extreme plausibility and asymmetry (level C) are resolved, we should solve the indications based on level B of evidence. This level should not be represented by evidence of dubious quality. The quality of the evidence should be of low risk for bias and high precision, here represented as indirect evidence of high level and direct evidence of satisfactory quality.

While the classification of the level of evidence is part of the scientific dimension, the strength of the recommendation involves and translates the dimension of clinical thinking: of the individual probability of benefit (magnitude of the effect) *versus* risk (harm/loss), of the doubt regarding feasibility (effectiveness), or even of cost-effectiveness questions (impact on the health system).

Thus, in analogy to the classification system adopted by ACC/AHA, we will name the grade of recommendation I and most of the time the grade of recommendation IIa as “strong”, which should be applied to those situations with little or no doubt regarding the ‘prescription’ process, which becomes almost a rule, except if specific contraindications apply. For example, the prescription of etiological treatment in cases of reactivation of CD (RCD). In contrast, the term “conditional” recommendation will apply to grade IIb (and occasionally to grade IIa), whose decision-making depends on a clinical analysis individualized in its magnitude of benefit and risk, patient’s values and preferences (shared decision-making), and aspects regarding the health system ( Chart 1.2 ).

**Chart 1.2:** – Grades of recommendation and levels of evidence.

TARGET AUDIENCE	GRADES OF RECOMMENDATION		LEVELS OF EVIDENCE	
	(1) STRONG	(2) CONDITIONAL	A	Direct evidence of good/high quality (RCTs with no important limitation or observational studies with indisputable expressive results conducted on CCCD).
Policy makers	The intervention should be adopted as a public health policy.	The intervention can be adopted as a health policy in some specific contexts, considering the risk-benefit analysis of this and other alternative interventions and health priorities.		
Health professionals	Physians are sure and confident to recommend the intervention.	Different choices can be adopted by physicians and the shared and informed decision-making process should consider the patients’ values and preferences.	B	Indirect evidence of good/high quality (extrapolation of results from RCTs without important limitation or observational studies with indisputable expressive results conducted on other cardiopathies) or Direct evidence of moderate quality (RCTs with limitations, subanalyses of RCTs including patients with CCCD, observational studies with satisfactory results conducted on CCCD).
Patients	Most patients, when well informed, would want the intervention; only a few would not.	Most patients, when well informed, would want the intervention, but many would not.	c	Absence of empirical evidence (case series, extreme plausibility, and principle of asymmetry).

CCCD: chronic cardiomyopathy of Chagas disease; RCT: randomized clinical trial.

### 1.2. Scientific Rationale for Recommendations of Diagnostic Methods

In parallel with the organization of the scientific thinking applied to recommendation on therapeutic management, a predominant theme in any guideline, we should widen the discussion for recommendation of diagnostic tests, because we also have chapters on such dimensions of medical decision.

In the context of diagnosis and differently from that of treatment, the grounding scientific concept of the level of evidence is not efficacy. Diagnosis involves the concept of accuracy, the ability to discriminate between sickness (sensitivity) and healthiness (specificity). Thus, the question is neither the conceptual proof of causality, nor the need for randomized experimental studies to minimize confounding factors. The need is to demonstrate enough accuracy so that the new information added by the test increases significantly the pretest diagnostic probability, within a structure of Bayesian thinking.

In that case, the best level of evidence for diagnostic accuracy derives from cross-sectional studies with proper methodology for patients’ selection, execution and reading of the predefined tests, performed to reduce systematic errors. It is worth noting that the studies of diagnostic accuracy are very sensitive to biases caused by retrospective observations from data banks (selection bias, spectrum bias, nonblind and non-standardized observation bias).

Thus, the quality of the evidence is essential, and recommendation based on preliminary information should be avoided. Similarly to what is used for treatment, the current guideline classifies as level of diagnostic evidence A and B those with satisfactory precision and low risk for bias, with level B refering to indirect evidence with high potential of generalization or to direct evidence of satisfactory quality. Level of evidence C is reserved for situations that do not require empirical evidence, incontrovertible situations. For example, the accuracy of the electrocardiogram (ECG) to define baseline cardiac rhythm.

Regarding the strength of the recommendation, the accuracy observed is a necessary, but not sufficient, condition. An accurate test has not necessarily strong indication. For that, three essential conditions apply: first, the diagnosis should be clinically useful, that is, should benefit the patient; second, the test’s additional information should be necessary and sufficient to increase the pretest diagnostic probability, which was previously undefined; and third, less complex, less invasive, of lower risk, or less expensive options should be absent. For example, although cardiac magnetic resonance imaging (CMRI) has better accuracy to assess systolic function, it is not strongly recommended because echocardiography (ECHO) is usually sufficiently accurate and widely available, unlike CMRI.

This analysis of the need for a certain diagnostic test and of its impact determines its strength of recommendation, which is usually defined based on clinical rationale. For example, in the case of a symptomatic patient, finding a defined problem is obviously useful, if a specific solution applies. However, the diagnostic usefulness becomes dubious in the case of screening tests, when there is strong *equipoise* between the intentional consequences of early diagnosis and the probability of harm. In such dubious circumstances, performing diagnostic tests by use of RCTs is suggested to substantiate the diagnostic effort.

Finally, it is worth emphasizing that the rationale described for diagnosis also applies to the definition of the level of evidence and strength of recommendation for risk factors and prognostic models.

## 2. Epidemiology – 21 st Century Update

### 2.1. Introduction

Chagas disease, also known as American trypanosomiasis, is a transmissible, potentially life-threatening illness caused by the protozoan parasite *T. cruzi* and belongs to the group of neglected tropical diseases (NTD) from the World Health Organization (WHO).^32 - 36^Discovered by Carlos Ribeiro Justiniano Chagas in 1909,^37^in the 21^st^century it continues to affect mainly socially vulnerable individuals and can generate severe physical (especially death and permanent disability), psychological (fear and stigma), and socioeconomic impacts, which reflect directly and indirectly on the quality of life.^1 , 2 , 36 , 38 - 43^

Political-institutional, economic, environmental (environmental degradation, climate changes – particularly temperature increase) and social (national and international human migrations, as well as precarious socioeconomic conditions, housing, education, sanitation, income, etc) factors are equally important central determinants of the global impact of *T. cruzi* transmission to the human species.^2 , 34 , 36 , 42 , 44^

For a deeper analysis of CD, it is essential to identify the different epidemiological scenarios and their transmission dynamics, involving not only individuals at risk for infection or already infected but also different *T. cruzi* strains, vector species, and reservoirs of the etiological agent, from the One Health perspective.^45^

*T. cruzi* is a hemoflagellate parasite, transmitted mainly through the contact of feces of different species of bugs of the *Hemiptera* order, *Reduviidae* family, *Triatominae* subfamily, whose habitat extends from Argentina and Chile to the southern half of the United States of America (USA), contaminated when sucking blood from infected individuals or animals.^2 , 36 , 38 , 39^

In addition, transmission can occur through: 1. ingestion of contaminated foods and beverages with triatomine bugs or their feces; 2. congenitally, from an infected mother to her fetus or newborn during pregnancy or delivery; 3. transfusion of blood or blood products from *T. cruzi-* infected individuals; 4. solid organ transplant from an infected person; and 5. accidents with biological materials, particularly in laboratories, in addition to sharing contaminated needles/syringes by individuals on illegal drugs.^2 , 32 , 34 , 36 , 38 , 39^From that perspective, the CD prevention and control actions are directly related to the *T. cruzi* transmission modes.^34 , 44^

Chagas disease is multisystemic and its natural history is characterized by an acute phase, which can last some weeks or months, usually asymptomatic or mildly symptomatic, and a chronic phase.^1 , 38 , 44 , 46 - 48^When not properly treated, the *T. cruzi* infection can last a whole life.^46^It is estimated that 30-40% of untreated infected individuals develop relevant clinical syndromes in the chronic phase, which can be life-threatening. In that phase, target-organs can be impaired, leading to cardiac, digestive, neurological, or mixed manifestations, which might require etiological treatment.^2 , 32 , 44^This aspect emphasizes the importance of the timely diagnosis, in initial phases of disease, particularly in individuals originating from poor or socially vulnerable communities.^2 , 42 , 44 , 49^

The economic burden generated by CD in the national health systems and society is significant, matching or even exceeding that of other diseases, such as rotavirus infection or cervical cancer, even in nonendemic areas.^36 , 50 , 51^A substantial proportion of the economic burden is consequent to loss of productivity due to early morbidity and mortality induced, particularly, by chronic cardiomyopathy.^34 , 50 , 51^Globally, the annual burden is US$ 627.46 million in health costs, with a current global net value of US$ 24.73 billion (annual costs per individual of US$ 4660 and, throughout life, per individual, of US$ 27 684). The global costs reached US$ 7.19 billion per year and US$ 188 billion throughout life. It is worth noting that approximately 10% of those costs are associated with areas where CD is not endemic, such as USA and Canada.^50^Thus, overcoming barriers to diagnosis and treatment access with the proper implementation of whole attention to individuals with CD would reduce the occurrence of chronic complication and the costs associated with the national health systems [for example, pacemaker (PM) implantation and repair surgeries], with a beneficial impact on the entire society.^34 , 44 , 50 , 51^

From that perspective, a comprehensive economic assessment regarding measures to expand access to CD diagnosis and treatment revealed the importance of the serological screening of candidates for blood donation and pregnant women, as the best cost-effective public health strategies.^52 , 53^Broader policies that recognize the different dimensions of social determination are fundamental to reduce that burden, requiring the involvement of other areas beyond the health sector.^2 , 33 , 45 , 54^Chagas disease is included in the 2030 Sustainable Development Goals (SDG) agenda, in its third objective, “ensure healthy lives and promote well-being for all at all ages”, aimed at “ending the epidemics of AIDS, tuberculosis, malaria, and neglected tropical diseases, and fighting hepatitis, waterborne diseases and other communicable disease” by 2030.^33 , 34 , 55^

Despite the high morbidity and mortality burden of CD and the elevated costs for the national health systems and mainly for society, 70-90% of the individuals with CD are reported to ignore their diagnosis, and only 1% effectively receives proper etiological treatment in the 21st century.^49 , 54 , 56^There is strong evidence that the diagnosis and proper etiological treatment of CD have several benefits, such as prevention of the congenital transmission in treated mothers, serological cure in babies and children, and a reduction in the progression to advanced clinical forms of CD in acutely and chronically infected individuals.^1 , 2 , 7 , 8 , 41 , 44 , 56 - 60^However, once the disease has progressed to a more advanced clinical phase, with severe heart impairment, the etiological treatment seems not to have any clinical benefit.^1 , 2 , 7 , 8 , 44 , 46 , 60^This strengthens the need to develop enhanced diagnostic methods in the local health services to guarantee access to early, safe, and effective treatment.^8 , 53 , 54 , 56 , 57 , 60^

In addition to the complex political, geographical, socioeconomic, cultural, technological, and legal challenges of the territories with higher endemicity for CD, barriers are known to persist regarding access to diagnosis, treatment, and longitudinal care.^44 , 54^Those barriers include: lack and inconsistency of data on the disease; limitation of integrated actions of surveillance, control, and care in the Primary Health Care (PHC) network; distance to health services; complicated diagnostic flowcharts and slow and costly processes (referral and counter-referral systems); limited integration of policies and actions for reproductive, maternal, neonatal, and child health; disproportional impact of CD on more vulnerable populations; limited knowledge of both the population and healthcare professionals about the disease; limited interest of the media and pharmaceutical industry; reduced health education initiatives; limited availability of tools and materials in healthcare centers; fear; stigma and discrimination against affected individuals; low capacity for social mobilization and limited political leadership of individuals at higher risk.^2 , 40 , 41 , 44 , 45 , 49 , 61^

It is worth noting that the limited knowledge of healthcare professionals about CD is one of the critical factors to ensure wide access to proper diagnosis and treatment in the national health systems.^2 , 61^In addition, there is clear need to overcome the access barriers related to the etiological treatment, currently limited to only two effective drugs - benznidazole and nifurtimox – which require relatively long periods of administration and may be associated with adverse reactions that can complicate treatment, requiring clinical and laboratory monitoring.^1 , 41 , 49 , 54 , 56 , 58 , 60^Furthermore, the antiparasitic drugs have limitations regarding their use by pregnant women and those with advanced disease with cardiac or cardiodigestive involvement.^2^However, for pregnant women with acute and severe clinical findings of CD (myocarditis or meningoencephalitis),^8^a decision about that ethical dilemma has to be made.^2^

In one of the eleven WHO public health global campaigns, the 72nd World Health Assembly approved, on May 24, 2019, the designation of a World Chagas Disease Day aimed at raising public awareness on this NTD.^36^In addition, the WHO’s road map for NTD identified three strategic actions to eliminate the disease: action 1 – engage with public institutions/agencies involved in prevention and control in different countries (ministries of health) to recognize CD as a public health problem and establish policies and effective actions of prevention, control, care, and surveillance in all endemic territories; action 2 – qualify medical care, from permanent education during service to integrated action across the entire care network; and action 3 – ensure that countries where within-household/extra-household vector transmission still occurs, comply with the protocols of prevention, control, and surveillance.^62^

It is noteworthy that society is increasingly participating in this endeavor. Social movements are engaging and taking a leading role in CD globally, as well as coordinating with other movements directed at NTD, in an effort to preserve fundamental rights such as access to health.^2 , 34^Those movements get together in a wider Social Forum to tackle NTD in Brazil.^49^In addition, those movements related to CD make up an international federation (FINDECHAGAS – *Federação Internacional de Associações de Pessoas Afetadas pela Doença de Chagas* [https://findeChagas.org/home-po/]) representing endemic and nonendemic countries.^2 , 34^

### 2.2. World Distribution of Chagas Disease

In the 21^st^century, CD maintains an epidemiological pattern of endemicity in 21 Latin-American countries, with approximately 70 million individuals at risk for exposure to *T. cruzi* infection. There is relative difficulty in establishing more precise estimates in the context of a NTD, raising uncertainty. However, the currently available estimates have been fundamental to subsidize agendas for CD control. The WHO estimates 6-7 million infected individuals around the world, most in Latin America, indicating a reduction of approximately 65% as compared to 1980 (17 million).

Approximately 63% of those cases are in countries of the Southern Cone Initiative, mainly Argentina (1.5 million), Brazil (1.2 million), Mexico (880 thousand) and Bolivia (610 thousand).^38 , 39 , 63^ Chart 2.1 shows the different patterns of epidemiological indicators of CD in Latin America at different times.

**Chart 2.1:** – Changes in mortality, prevalence, and incidence of Chagas disease (vector and congenital transmission) in 21 endemic countries in Latin America, in 1980–1985, 2005, and 2010.

PARAMETERS - ESTIMATES	1980–1985	2005	2010
**Number of deaths/year**	>45 000	12 500	12 000
Number and percentage of infected individuals	17 395 000 (4.3%)	7 694 500 (1.4%)	5 742 167 (1.1%)
New cases/year – total	700 000	55 585	38 593
New cases/year – vector transmission	>500 000	41 200	29 925
New cases/year – congenital transmission	7000 - 49 000	14 385	8668
Number and percentage of total population at risk	92 895 000 (25.0%)	108 595 000 (20.4%)	70 199 360 (12.9%)

Source: Adapted from Dias et al., 2016; ^2^ WHO, 2002; ^38^ PAHO, 2006; ^39^ and WHO, 2015. ^63^

However, those global data differ from the individual estimates in several countries, hindering the exact establishment of CD prevalence in the Americas.^2^In addition, under-reporting of cases and not reporting deaths due to CD represent critical obstacles, because they prevent the adoption of control measures more adjusted to local realities based on epidemiological surveillance.^34 , 64 , 65^

Despite these difficulties, the development of regional initiatives, coordinated to interrupt *T. cruzi* transmission, was able to expressively reduce the global prevalence of CD.^2 , 34 , 66 , 67^The agreed goals to eliminate transmission by the major vector species ( *Triatoma infestans* ) and by blood transfusion was achieved by several countries based on initiatives since the 1990 decade, with a significant reduction in the number of new cases; however, some critical areas of transmission still persist.^2 , 66 , 68 , 69^

The current challenges are even more important. Only 10%-30% of the individuals affected by CD are aware of their diagnosis, which contributes to the fact that only 1% of those requiring etiological treatment have actual access to it, maintaining morbidity, mortality, and social cost at high levels, with impairment of quality of life.^36 , 43 , 54 , 56 , 60^Furthermore, in most areas where vectorial interruption or transmission reduction was achieved, the population affected is getting older, increasing the burden of morbidity and mortality due to the coexistence of chronic-degenerative diseases, mostly cardiovascular diseases.^41 , 66 , 70 - 72^In the elderly population, CCD remains a strong predictor of higher risk for death.^70^

Despite the significant reduction in prevalence, approximately 10-15 thousand deaths related to CD are reported every year,^2 , 36 , 59^although a significant reduction has been observed, considering the recording of over 45 thousand deaths annually in the 1980 decade. However, mortality still remains high,^63 , 65 , 70 , 73^contributing to maintain CD a public health problem.^44 , 70 , 74 , 75^

Beyond the classically endemic areas in Latin America, CD has been progressively reported in nonendemic countries (some in Europe, USA, Australia, and Japan), because of the migration movements associated with political-institutional, sanitary, environmental, and economic crises in some countries.^2 , 42 , 67 , 76 - 81^

Those global estimates are supported by recent data originating from countries, such as Spain, where, although the disease is not endemic, there is active research and focus on public health measures for control. In Spain, they estimated for 2018 over 55 thousand of the almost 2.6 million migrants from endemic countries (54% from Bolivia) living with CD, an estimated prevalence of 2.1%. Approximately 70% of migrants did not have an established diagnosis and most were not treated, 83% were older than 15 years and 60% were children.^82^

In addition, those populations live in precarious conditions, are socially vulnerable because of social and economic restrictions that hinder access to healthcare, which is aggravated by the poor professional experience in the specific health sector.^42 , 61 , 67 , 77 , 80 , 81^It is worth noting that *T. cruzi* can act as an opportunistic microorganism in individuals with other immunosuppression-associated pathologies, resulting in life-threatening clinical syndromes due to RCD.^1 , 83 , 84^

In those nonendemic contexts, the likelihood of *T. cruzi* transmission through blood transfusion has been more and more recognized. Although CD is rarely defined as a public health problem in nonendemic countries, in the past 10 years several blood centers implemented measures to mitigate the risk related to blood safety based on the recognition of epidemiological risk factors associated with Latin-American immigrants and the adoption of serological tests for screening.^42 , 79^

In endemic contexts, the control of two transmission modes (vectorial and transfusion) puts into perspective the congenital mode, responsible for almost one third of the new infections in 2010.^41 , 63 , 69 , 85 - 87^In endemic regions, 1.12 million women at reproductive age are estimated to be infected,^59^and the mean congenital transmission rate is estimated as 5%, mainly in high-risk endemic areas.^59 , 88^Because the access to diagnosis of *T. cruzi* infection in mothers or newborn babies is limited in most endemic areas, the prevalence in pregnant women and newborns can be underestimated.^59 , 88^Even with such limitations, the incidence is estimated at 8000 to 15 000 cases of congenital transmission per year in Latin America.^85^Of note, that mode of transmission has played a central role in maintaining *T. cruzi* infection in nonendemic areas.^59 , 63 , 86 , 87^Thus, the occurrence of congenital infection can sustain *T. cruzi* transmission indefinitely, even in countries without the classic vectorial mode.^86 , 88^

To prevent congenital transmission in endemic areas, it is essential to guarantee access to diagnosis and etiological treatment to girls and women at reproductive age before pregnancy.^57 , 59 , 89^In addition, the diagnosis of *T. cruzi* infection in pregnant women during the prenatal period, favoring the early screening of the newborn infection, as well as the diagnosis of infection in newborns from infected mothers, enabling the implementation of etiological treatment, would be highly effective and safe measures.^41 , 57 , 59 , 85 , 86 , 88 , 89^

Oral transmission, on the other hand, has been reported particularly in the Amazonian region and subtropical Andes,^90^playing an important role in the appearance of acute cases in the Brazilian Amazonian region and Venezuela.^2 , 48 , 90^In those scenarios, mortality is higher during the acute phase as compared to that of acute cases caused by the classic vectorial transmission.^2 , 47 , 48^Acute CD orally transmitted has considerable lethality in the first year after infection,^48^as discussed in another chapter of this guideline.

### 2.3. Overview of Chagas Disease in Brazil

In the 21^st^century, it is unequivocally important to sustain CD surveillance and control in all its evolutionary clinical phases, considering as criteria, the magnitude, spread potential, transcendence, vulnerability, and Brazil’s international commitments.^2 , 34 , 69 , 91^As a country of continental dimensions, throughout this century Brazil has undergone demographic, social, economic, and environmental changes, failing to overcome the critical socioeconomic and regional inequalities.^2 , 33 , 52^

However, Brazil has a public, universal, and democratic unified health system (SUS), whose quality should continue to enhance constantly, aimed at ensuring the access to health for all individuals, a right established in the 1988 Federal Constitution 1988.^2 , 33 , 49 ,92^

In that context, CD remains the NTD with the highest morbidity and mortality burden, particularly among elderly men and those who had lived in important endemic areas of vectorial transmission.^73 , 74 , 75^Considering Brazil’s extension and diversity, with implications in the ecological, demographic, social, and economic dynamics of the regions, there are multiple clinical, epidemiological, and operational scenarios for disease control.^2 , 34^

Vectorial control in endemic areas had a significant impact on both blood transfusion and congenital transmissions,^64 , 69 , 87^but the current weakening of the entomological surveillance operations in endemic municipalities is worrisome. The certification of interruption of Chagas disease transmission by the major domestic vector, *T. infestans,* was issued in 2006 by the Pan American Health Organization (PAHO), within the Southern Cone Initiative.^2 , 34^Despite advances, the risk of CD transmission by vectors persists and has been assessed under different perspectives because of several factors, such as the existence of autochthonous triatomine species with a high potential of colonization, the presence of wild and domestic reservoirs of *T. cruzi* , the increasing proximity between human populations and those environments, in addition to the persistence of residual *T. infestans* foci, even in specific areas of the Bahia state, and the limitation of entomological surveillance actions.^2 , 34^

In Brazil, in 1980-1985, 6 180 000 (4.2%) individuals were estimated to be infected with *T. cruzi* , and, in 2000, 1 900 000 (1.0%).^38 , 39^The WHO’s most recent estimates indicate in 2010 1 156 821 infected individuals with *T. cruzi* (0.6%).^63^However, the limitation of population-based studies hinders more realistic assessments about the magnitude of CD in Brazil.^64^Thus, some studies based on systematic reviews and meta-analyses of data available in Brazil have estimated the number of infected individuals ranging from 1.9 to 4.6 million, figures that might be closer to the current variation from 1.0% to 2.4% of the population.^2 , 64^Based on those proportions, the number of Brazilians infected with *T. cruzi* estimated for 2020 is 1 365 585 to 3 213 142, of whom, 136 559 to 321 314 individuals with the chronic digestive form and 409 676 to 963 943 with the chronic cardiac form. Regarding the IFCD, the population estimated with *T. cruzi* infection ranged from 819 350 to 1 927 885 individuals.^2^ Chart 2.2 shows the projections of the numbers of *T. cruzi* infected individuals and cases in the chronic phase of CD with cardiac and digestive forms in Brazil from 2020 to 2055.

**Chart 2.2:** – Projections of the number of individuals infected with T. cruzi and of individuals with chronic Chagas disease in the cardiac and digestive forms in Brazil, 2020–2055.*

YEAR	ESTIMATE OF THE BRAZILIAN POPULATION	AGE GROUP OF REFERENCE			ESTIMATE OF THE NUMBER OF INFECTED INDIVIDUALS		ESTIMATE OF INDIVIDUALS WITH THE DIGESTIVE FORM		ESTIMATE OF INDIVIDUALS WITH THE CARDIAC FORM	
		AGE GROUP	POPULATION	%	INFECTION 1.02%	INFECTION 2.4%	INFECTION 1.02%	INFECTION 2.4%	INFECTION 1.02%	INFECTION 2.4%
2020	212 077 375	≥ 25	133 880 929	63.1	1 365 585	3 213 142	136 559	321 314	409 676	963 943
2025	218 330 014	≥ 30	127 334 466	58.3	1 298 812	3 056 027	129 881	305 603	389 644	916 808
2030	223 126 917	≥ 35	120 096 221	53.8	1 224 981	2 882 309	122 498	288 231	367 494	864 693
2035	226 438 916	≥ 40	112 013 898	49.5	1 142 542	2 688 334	114 254	268 833	342 763	806 500
2040	228 153 204	≥ 45	102 983 115	45.1	1 050 428	2 471 595	105 043	247 160	315 128	741 479
2045	228 116 279	≥ 50	92 984 144	40.8	948 438	2 231 619	94 844	223 162	284 531	669 486
2050	226 347 688	≥ 55	82 097 220	36.3	837 392	1 970 333	83 739	197 033	251 218	591 100
2055	222 975 532	≥ 60	70 485 475	31.6	718 952	1 691 651	71 895	169 165	215 686	507 495

*For infection rates of 1.02% and 2.4%, and considering 30% of patients with the cardiac form and 10% with the digestive form of Chagas disease. Source: Adapted from Dias et al., 2016 ^2^

The prevalence of *T. cruzi* infection in pregnant women in Brazil in 2010 was estimated as 1.1% (34 629 women), with a mean of 589 newborns with congenital infection (transmission rate of 1.7%),^87^similar to the WHO’s estimate (571 cases).^63^The congenital transmission rate is lower (1.5-2.0%) as compared to the mean of 5% observed in other Southern Cone countries, such as Argentina, Paraguay and Bolivia. Such findings suggest that the presence of TcII associates with lower transmission as compared to that of TcV, which predominates in the Southern region of Brazil and those countries.^2 , 57^

Based on data from the Notifiable Diseases Information System (SINAN), the occurrence of cases of acute CD has been the aim of epidemiological surveillance, according to the Brazilian Ministry of Health definition of “case”. From 2007 to 2019, 3060 cases of acute CD (mean of 222 cases/year) were confirmed in 219 municipalities.^34^In 2020, 146 cases were confirmed, mainly in the North region, with case fatality rate of 2% (3/146 - all deaths in the Pará state). The most frequently reported mode of transmission in Brazil in the past 15 years in cases of acute CD was the oral one,^34 , 93^revealing the operational limitations of the surveillance process in Brazil, which have induced changes in the epidemiological profile of the disease in the past decade.^2 , 90^

The CD mortality burden in Brazil persists significantly high, despite the control actions implemented. Mortality is known to be more expressive at ages 50 to 64 years and older cohorts, probably due to the effects of the period of intensification of vectorial control actions, in addition to demographic changes.^35 , 75^The differences observed between regions, especially with higher burden in the West-Central and Southeast regions, indicate socioeconomic inequities and different patterns of access to healthcare services in the SUS.^35 , 73^The South region shows a reduction in the trend of mortality, the North region, an increase, while the Northeast region has no defined trend.^35 , 74^

It is worth noting that the North region concentrates the majority of the new cases reported in the country.^34 , 47 , 93^In addition to the probable underreporting of cases not associated with domiciliary vectorial transmission, that region had little impact from the systematic actions for triatomine control. This is justified because the local cycle of *T. cruzi* transmission does not involve vectors with the ability of domiciliation, but is sustained in an enzootic cycle, with wild vectors implicated in cases associated with oral or extra-household vector transmission.^2 , 47 , 48 , 60 , 73^Thus, it is reasonable to estimate that the accumulation of hundreds or even thousands of cases of *T. cruzi* infection over time in the Amazonian region might be contributing to this specific epidemiological pattern.^47^

Chagas disease continues to have a strong impact on Social Security and the services of the National Institute for Social Security (INSS) in the Brazilian states with higher prevalence,^34^particularly as the affected population ages.^2 , 72^The global analysis for the 2030-2034 period indicates a progressive decline in mortality (over 75% as compared to that in 2010-2014), mainly among the youngsters, ranging from 86%, in the age group from 20 to 24 years, to 50% in those aged 80 years and more.^75^It is worth noting the significant reduction in the quality of life of individuals with CD and their families.^43^

Integrated actions of care, surveillance and control of CD in PHC have been fundamental and strategic to reduce the morbidity and mortality burden, mainly in endemic territories, expanding access to diagnosis and etiological treatment.^2 , 34 , 44 , 58 , 75^The PAHO document “ *Chronic Care for Neglected Infectious Diseases: Leprosy/Hansen’s Disease, Lymphatic Filariasis, Trachoma, and Chagas Disease – A guide for morbidity management and disability prevention for primary health care services* ” is a landmark, because it highlights several fundamental aspects in the care of individuals with CD, aiming at providing PHC teams with tools, and reinforces the importance of integrated surveillance actions.^44^

### 2.4. Epidemiological Surveillance in Brazil

Epidemiological surveillance for CD comprises necessarily integrated actions that involve approaching human cases, vectors, and reservoirs, maintaining an interface with the healthcare network and special emphasis on the PHC role.^2 , 34 , 91 , 93^

The major objectives of the epidemiological surveillance actions for CD in Brazil are as follows: 1) to early detect cases of acute CD for proper etiological treatment and application of measures to prevent the occurrence of new cases; 2) to conduct an epidemiological investigation of all acute cases, aiming to identify the mode of transmission and adopt proper control measures; 3) to monitor *T. cruzi* infection in the human population by use of PHC screening programs, periodical serological inquiries in strategic populations, and analysis of the screening process of candidates for blood donation in blood centers; 4) to monitor the morbidity and mortality profile of CD, outlining actions to strengthen the healthcare network for infected individuals; 5) to maintain transmission by *T. infestans* eliminated and that by other important species under monitoring/control; and 6) to integrate actions of sanitary, environmental, vectorial, and reservoir surveillance with epidemiological surveillance actions.^2 , 93^

The data available on the epidemiological surveillance of human cases do not enable estimating the nosological magnitude of American trypanosomiasis. Only 10-20% of the acute CD cases are estimated to be reported.^2 , 47^Up to May 2020, when the chronic phase of CD was included as an event of interest for epidemiological surveillance, by use of mandatory reporting of cases (Ordinance nº 1.061, of May 18, 2020), only the traditional surveillance of cases in the acute phase was performed and included in the National List of Notifiable Diseases of Mandatory and Immediate Reporting.^34 , 93^This enlargement in the surveillance scope is very important for our country to reach national recognition of patterns of disease occurrence and can be followed by other endemic countries. This new process of epidemiological surveillance is expected to be implanted in the entire Brazilian territory by 2022.

More recently, to recognize the magnitude of chronic CD in the country, the importance of rearticulating and integrating health surveillance actions have been discussed, aimed at the development of a large hierarchical network of health services in several territories to guarantee access for millions of *T. cruzi* infected individuals.^44 , 91 , 94^To elaborate a prioritization model of municipalities for chronic CD surveillance, a team of the Ministry of Health conducted a preliminary multicriteria decision analysis based on three indices built from the following indicators: (a) epidemiological, directly related to chronic CD; (b) resulting from the progression of chronic CD; and (c) related to access to health services. The model defined as the most suitable was composed by 1345 municipalities of intermediate priority, 1003 of high, and 601 of very high priority for chronic CD, mainly in the Southeast and Northeast regions of the country.^94^

After that, the Ministry of Health proposed the elaboration of an index of vulnerability for chronic CD to evidence areas at higher risk for morbidity and mortality in that phase of the disease, taking into consideration contexts of limited access to the healthcare service network, with low diagnostic suspicion and case detection and limitation of the quality of life of the individuals affected.^91^Thus, three subindices were developed from the three indicators integrated in the previous analysis.^91 , 94^The value of the index can range from 0 to 1, and the closer to the value ‘1’, the higher the vulnerability for chronic CD ( Figure 2.1 ).^91^

**Figure 2.1:** – Spatial distribution of chronic Chagas disease (CCD) vulnerability index according to the federative units (FU) and Distrito Federal (A) and health macroregions (B). Source: CGZV/Deidt/SVS

An additional perspective of CD surveillance in Brazil leads to the recommendation that an anti- *T. cruzi* antibody testing request should be made available to every infected individual with the human immunodeficiency virus (HIV) or with acquired immunodeficiency syndrome (AIDS), based on the existence of epidemiological antecedent. This recommendation has been debated more recently in other countries, such as the USA.^95^It is worth noting that, for the purpose of epidemiological surveillance, since 2004 Brazil has inserted RCD in the list of diseases indicative of AIDS in the presence of HIV infection, from the definitive diagnosis of meningoencephalitis and myocarditis associated with CD.^2 , 83 , 84^

### 2.5. Association of Chagas Disease with COVID-19

The emergence of COVID-19, caused by the severe acute respiratory syndrome coronavirus-2 (SARS-CoV-2), has brought serious and unprecedented global challenges for the national health systems and humankind.^42 , 80 , 96 , 97^Its pandemic character has been compounded by its high infectivity, even in its asymptomatic phases, leading to its rapid spread.^98 , 99^

As the COVID-19 global pandemic advances, it disproportionately impacts more and more socially vulnerable populations,^80 , 98 , 99^which already bear a significant morbidity and mortality burden for NTD. Thus, the analysis of the current context of NTD provides relevant possibilities to approach gaps in COVID-19 control, because it is an important referential for the progress in responding to the needs of more vulnerable populations. Success in responding to COVID-19 control without reducing the NTD burden concomitantly points to flaws in the sustainability of the national health systems to maintain that control.^97^

The concomitance of CD is particularly worrisome because it can cause cardiac, gastrointestinal, neurological and other complications, increasing susceptibility to COVID-19.^71 , 100 - 102^The highest prevalence of comorbidities seems to be related to a worse prognosis in coinfection.^102^Since the appearance of the pandemic caused by SARS-CoV-2, the cardiovascular involvement has been identified as a frequent complication of COVID-19.^96 , 100^However, there is little evidence on the effects of COVID-19 on individuals with CD.^100 , 102 - 104^Some studies have reported that COVID-19 may add new challenges in guaranteeing access to total healthcare (diagnosis and treatment, as well as throughout the full disease cycle) to those individuals and in the necessary development of new research analyzing the implications of SARS-CoV-2 coinfection.^97 , 103^

In addition, both diseases are similar regarding the susceptibility to risk factors, molecular patterns associated with the pathogen, recognition of glycosaminoglycans, inflammatory process, vascular hypercoagulability, microthrombosis and endotheliopathy, and, thus, may require treatments with similar principles.^105^However, it is worth noting the importance of considering the disease’s different clinical forms and specific pathophysiological mechanisms associated.^102^Thus, despite some similarity regarding pathophysiology, which involves high risk of thromboembolism in COVID-19 and in the chronic cardiomyopathy of Chagas disease (CCCD), cautiousness is required regarding the recommendation of immediate treatment for CD with anticoagulant drugs, whose potential benefit should be restricted to clinical scenarios in which there is a favorable adequate risk relation of hemorrhage *versus* thrombosis with the use of those drugs. Such principles are discussed in another chapter of this guideline.

Some studies have pointed to high levels of comorbidity in cases of CD associated with severe forms of COVID-19. It is important to emphasize that those comorbidities reflect the more advanced ages of the populations that are specially impacted by CD and COVID-19.^71 , 100 - 102^Although more than 80% of the cases of COVID-19 are mild or asymptomatic, severe cases have been more frequent among elderly and individuals with comorbidities, while, for CD, elderly with chronic cardiomyopathy are at a higher risk of death, which is partially justified by its association with age or other chronic conditions and by social poverty.^71 , 100^

Although coinfection might be associated with a higher risk for complications, with a worse clinical prognosis, a multicenter prospective study with 37 hospitals in 17 municipalities of 5 Brazilian states (Minas Gerais, Pernambuco, Rio Grande do Sul, Santa Catarina, and São Paulo) reported that there was no significant differences in the clinical presentation nor in the outcomes of cases with CD as compared to controls, despite the evidence in the beginning of the study of higher frequency of chronic HF and atrial fibrillation (AF). In addition, that study reported lower level of reactive C protein among participants with CD.^104^

The higher social vulnerability of individuals with CD in the context of poverty can be increased by COVID-19, because of its political-economic impacts.^80 , 97 - 99^The significant increase of extreme poverty around the world in the past decade compounds the challenge of access to healthcare for individuals with CD.^71 , 101 , 106^

In addition, individuals with CD might be afraid to search care because of fear of exposure to COVID-19, postponing the solution for complications related to disease and increasing the emotional burden of the disease due to the associated worries. And all that is compounded by the weakening, disorganization, and overload of the national health systems.^71^

Brazil is one of the countries with the highest COVID-19 morbidity and mortality burdens and has called negative attention in the international scenario because of its lack of coordination and leadership regarding the COVID-19 surveillance and control actions.^92, 99 , 107^Moreover, the inequity of COVID-19 expression in Brazil has been evidenced by the excessive mortality of black/mixed heritage individuals of all age groups.^99 , 108^These racial disparities can be justified by historically determined socioeconomic conditions, which usually define who can maintain social distancing and avoid exposure to SARS-CoV-2.^71 , 99 , 108^

In addition, the risk of death from COVID-19 showed a wide range in the pandemic initial stages, with increased vulnerability of the peripheral areas, where the most vulnerable communities are found, jeopardizing the health system’s ability to respond and increasing the inequities in health care.^99 , 106^

Through the informative note nº 9 from 2020 (CGZV/DEIDT/SVS/MS), recommendations from the Ministry of Health were established in Brazil to adapt surveillance and healthcare actions for individuals with CD considering the COVID-19 epidemiological situation.^109^Despite those orientations, the specific Epidemiological Bulletin from the Ministry of Health raised the possibility of impact of the COVID-19 pandemic on the morbidity and mortality profile and surveillance actions for CD in Brazil.^93^

That bulletin points to evidence of cardiovascular diseases as critical risk factors for higher severity of the clinical syndrome associated with COVID-19. Based on that, individuals with CD should be considered a population at higher risk for worse clinical progression of COVID-19, requiring more careful attention by the SUS during the pandemic.^93^

In addition, that epidemiological bulletin from March to August 2020 reported 1746 deaths in Brazil with CD as the underlying cause of death (data from the Brazilian Mortality Information System), 29 of which cited COVID-19 or severe acute respiratory syndrome as an aggravating condition or a condition contributing directly or indirectly to the causal chain of death (parts I and II of the certificate of death), with a higher proportion in the Southeast and Northeast regions.^93^During that period, 125 691 deaths from COVID-19 were reported, in 207 of which (0.2%) CD was mentioned as the condition contributing to death (part II of the certificate of death), with a higher proportion in the Southeast and Northeast regions. Most of those deaths occurred in female individuals (52.7%), of mixed heritage (42.0%), with a mean age of 74 years (SD±11.36), and in the age group over 75 years (53.0%).^93^

Some hypotheses point to *T. cruzi* and SARS-CoV-2 coinfection as an important uninvestigated causal binomial of death in CD endemic regions.^101^

The regionalized temporal trend analysis in the country, from 2009 to 2019, reveals propensity for a statistically significant reduction in the specific coefficient of mortality from the disease. However, a tendency to increase was observed in the coefficient of incidence of acute phase cases, which was statistically significant in the North region; however, in 2020, the number of cases reported was lower than that foreseen.^93^

Regarding diagnosis, a 24% reduction was observed in the number of requests for laboratory tests for CD diagnosis processed in 2020 as compared to the mean from 2017 to 2019.^93^In addition, a reduction in treatment was observed, evidenced by the reduction in benznidazole distribution and the entomological surveillance assessment in the state coordinations,^93^indicating a possible reduction in the sensitivity of the healthcare and surveillance network, probably related to directing municipal and state efforts to fight the COVID-19 pandemic.

Even with guidance on the need to readapt the entomological surveillance activities in the context of COVID-19,^109^the reports of state representatives indicate that, in many territories, the foreseen control activities for 2020 could not be performed, not even partially.^93^

Finally, because of the recent evidence that cardiovascular sequelae persist in the long term in individuals with COVID-19,^110^this could be even more ominous for those individuals already with CCCD when they get infected with SARS-CoV-2.

### 2.6. Final Reflection on the Current Chagas Disease Epidemiological Scenario

Recent publications by researchers and managers from both nonendemic^111 , 112^and endemic countries,^113 , 114^indicate the pressing need to adopt comprehensive policies regarding public health to effectively control the inter-human transmission of the *T. cruzi* infection and to reach an optimal level of care for already infected individuals, focused on providing both diagnostic and therapeutic opportunities.

## 3. Pathogenesis of Cardiomyopathy of Chagas Disease

### 3.1. Introduction

The CCCD pathogenesis is still object of intense debate. In the acute phase of CD, the intense tissue parasitism has always been recognized as an essential mechanism; however, in the chronic phase, this has not occurred and other pathogenetic hypotheses predominated during the second half of the 20^th^century. It was only from the year 2000 on that the notion of the persistence of the parasite in the myocardial has consolidated as the primordial mechanism for the CCCD installation. This rescued the concept of CD as a truly infectious entity and that of CCCD as caused by a low-intensity, but virtually incessant, focal inflammatory process. The tissue aggression, causing necrosis and reactive and repairing fibrosis, is directly stimulated by *T. cruzi* and the adverse immune reaction to parasite persistence.

The prognosis of CCCD is usually more ominous than that of non-inflammatory cardiomyopathies. The identification of prognostic factors and therapeutic targets is critically dependent on that knowledge. The direct lysis of infected cells is significant mainly during the acute phase of the infection, when intracellular parasites are abundant and myocarditis is usually diffuse and intense. On the other hand, chronically infected individuals have a clearly different disease progression. Decades after the infection, approximately 60% of infected individuals remain free from clinical manifestations of the disease for their entire life (stage A - IFCD), 10% develop gastrointestinal disease, and 30% develop CCCD, which can be classified into stages B1/B2 (less advanced cardiomyopathy) or C/D (severe cardiomyopathy), as detailed in another chapter of this guideline.

The major pathogenic hypotheses to explain the beginning and progression of CCCD include: 1) direct parasite-induced damage to the tissues; 2) indirect inflammatory/immune damage to the tissues; 3) neurogenic disorders; 4) microvascular disorders. The neurogenic hypothesis was based on intracardiac neuronal depletion and consequent dysautonomia, but there are unavoidable obstacles to the postulated cardiopathy resulting from parasympathetic deprivation (‘parasympathetic-deprived’ cardiopathy). Evidence from experimental models and human disease indicates that inflammatory infiltrates are the major cause of damage to the cardiac tissue. However, more recent evidence has shown that genetic susceptibility and mitochondrial damage are important parts in the CCCD pathogenesis. Cardiac microcirculatory lesions have been reported in CCCD, but microvascular ischemia can result from the action of inflammatory mediators and constitute a mechanism of positive feedback, potentializing the inflammatory and mitochondrial damages, as discussed in the following section.

### 3.2. Immune Dynamics and Differential Progression to Chronic Cardiomyopathy of Chagas Disease

In the acute phase of the infection, which has been investigated in more details in murine models, parasitemia and intense parasitism of the tissues trigger a strong immune response. Initially there is innate immune response, followed by that depending on cytotoxic T lymphocytes and T lymphocytes that produce inflammatory cytokines, such as interferon-gamma (IFN-γ) and tumor necrosis factor alpha (TNF-α), and specific anti- *T. cruzi* antibodies that partially control the parasitism, establishing persistent, although low-grade, infection.^115 , 116^

Different lineages of mice infected with the same lineage of *T. cruzi* show different severities of CCCD, characterized by electrocardiographic and echocardiographic changes, associated with varied serum levels of TNF-α and nitric oxide, suggesting that variations in the host’s genetics can determine chronic disease severity.^117 , 118^

Persistent parasitic stimulation induces the systemic production of IFN-γ and TNF-α in individuals with chronic CD, which is particularly intense in those with CCCD as compared to those with the IFCD.^119 , 120^A relation between the intensity of the acute phase of *T. cruzi* infection and the severity of its chronic phase has been proposed. Patients with CCCD have diffuse myocarditis (rich in macrophages, CD8+ cytotoxic lymphocytes and CD4+ T lymphocytes) with fibrosis and hypertrophy. Myocarditis is due to both *T. cruzi* -specific lymphocytes and autoimmune T lymphocytes, which produce large amounts of IFN-γ and TNF-α. In CCCD, IFN-γ plays a central pathogenic role by inducing cell damage via several mechanisms, while other inflammatory mediators also act.

Recent review on systemic and heart-specific immune alterations has shown that patients with CCCD have a characteristic inflammatory cytokine profile.^115^Significant systemic immune effects were observed in the peripheral blood of patients with chronic CD, which are associated with the distinct clinical forms. It is important to note that qualitative differences are clearly observed in the systemic cellular responses of patients with the IFCD and cardiac clinical form. Those differences are influenced by an immune-regulated cytokine network, which orchestrates the immune response. While individuals with IFCD have a balanced immune-regulatory profile modulated by the production of interleukin (IL)-10,^121^patients with CCCD have an increased frequency of CD4+ and CD4-CD8- T cells producing IFN-γ, as well as increased levels of circulating TNF-α in the peripheral blood.^122^

In addition, the expression of IFN-γ is elevated in patients with the DCM form of CD as compared to the still nondilated form of CCCD.^123 , 124^Also, patients with CCCD have reduced numbers of circulating Th17 T cells^125 , 126^and of monocytes producing IL-10,^127^CD4+CD25+ regulatory T cells (Tregs),^128 - 132^as well as reduced levels of Ebi/IL-27p28^133^as compared to individuals with the IFCD ( Figure 3.1 ). Such immune-regulatory alterations correlate with contractile depression, because the high frequency of cells producing IFN-γ and TNF-α is associated with low LVEF.^134 , 135^

**Figure 3.1:** – Pathogenic events in the progression of chronic cardiomyopathy of Chagas disease (CCCD). (A): events in the acute phase of Chagas disease (CD); (B): primary pathogenic events in the chronic phase of CD, with the fundamental stages and clinical manifestations (see the CD natural history chapter); (C) events and pathophysiological disorders in more advanced phases of CD. miRNA: microRNA.

On the other hand, higher numbers of cells producing IL-17 and IL-10 are associated with the preservation of LVEF within the normal range.^136 - 138^Self-reactive B cells^139^and subpopulations of B cells associated with potentially protective or pathogenic responses were identified in patients with CD.^140^In addition, the production of lytic antiparasite antibodies was proposed as a mechanism to control parasites.^141^Activation of monocytes,^127 , 142 , 143^CD4+ T cells with T-cell-specific receptors,^136 - 138^CD8+ T cells^144 - 147^and other less numerous but very active T cell populations, such as CD4-CD8- T cells, was shown in patients with CD.^135 , 148^

An exacerbated Th1 response in the peripheral blood of patients with CCCD reflects on the inflammatory infiltrate rich in Th1 secreting predominantly IFN-γ and TNF-α, with lower production of IL-4, IL-6, IL-7, IL-15, IL-18, as evidenced in immunohistochemistry and mRNA expression studies.^123 , 149 - 153^In the cardiac tissue of patients with CCCD, IFN-γ is the most upregulated cytokine. Thus, a significant expression of T-bet, Th1 transcription factor, is observed in the myocardium of patients with CCCD.^154^

In addition, there is a positive correlation between the T-bet gene expression and dilatation of the left ventricle, corroborating the pathogenic role of T cells producing IFN-γ. However, the mRNA expression of GATA3, RORγT and FoxP3, T cell subset that defines transcription factors of Th1, Th2, Th17, and Treg populations, with their cytokines IL-4, IL-13, IL-17, IL-10, and molecular markers (FoxP3 and CTLA4), was low or undetectable.^154^

The number of CCR5+ CXCR3+ Th1 T cells producing IFN-γ is higher in patients with CCCD than in those with the IFCD,^155^and those same cells were identified in the cardiac tissue of patients with CCCD, along with their chemokine receptors (CCL3-5, CXCL9 and CXCL10, respectively). CCL5 and CXCL9 were the most often expressed chemokines and the severity of myocardial inflammation correlated positively with the mRNA expression of CXCL9.^151 , 156^

In animal models of CCCD, in the acute and chronic phases of *T. cruzi* infection, CCL3, CCL4 and CCL5, acting via CCR1 or CCR5, control the migration of T cells and macrophages to the cardiac tissue, leading to cardiomyocyte damage, conduction abnormalities, and ventricular dysfunction.^157 , 158^This suggests that Th1 chemoattractive chemokines locally produced play a significant role in the selective accumulation of Th1 T cells in the heart in the CCCD. In addition, it indicates that there is essentially no regulation via either T cells or regulatory cytokines in the myocardium infiltrated with Th1 of patients with CCCD.

The low regulation could explain the inflammatory infiltrate destructiveness, most probably due to the excessive collateral damage caused by the T cells producing IFN-γ. The non-antagonism to IFN-γ in patients with CCCD might be related to the decreased number of the T cells producing IL10 and Ebi/IL27R and regulatory ones, all able to suppress the production of IFN-γ and/or differentiation of Th1 T cells.

### 3.3. Myocardial Mitochondrial Dysfunction and Chronic Cardiomyopathy of Chagas Disease

Wan *et al* . were the first to implicate myocardial mitochondrial dysfunction and oxidative stress in the pathogenesis of CCCD in murine models.^115 , 158^The remarkable similarity between cardiac, digestive, and autonomic disorders in the mitochondriopathies (15% develop gastrointestinal motility disorders and 40% develop cardiomyopathy and arrhythmia),^159 , 160^as well as the broad clinical spectrum of symptomatic CD,^161^suggest mitochondrial dysfunction as a fundamental component of the CCCD pathogenesis.

In CCCD, the myocardium shows signs of reduced mitochondrial activity and energy production. The reduction in mitochondrial ribosomal RNA^151^and mitochondrial DNA,^162^in addition to other observations (not published) and *in vivo* production of adenosine triphosphate (ATP),^163^has been described in the myocardium of patients with CCCD.

The myocardial levels and activity of the enzymes of the mitochondrial energy metabolism, ATP synthase and creatine kinase, are even lower than those in other cardiomyopathies,^164^which could contribute to the worse prognosis associated with CCCD. The discovery of the association of CCCD with rare variants of mitochondrial genes, described in more details in this chapter, supports the role played by mitochondrial dysfunction in the myocardial injury of patients with CCCD and can be a mechanism to perpetuate the inflammation and damage in cardiomyocytes.^115 , 161^

Recent studies have shown the modulation of the expression of some microRNAs (miRNAs), molecules that specifically control the translation of mRNA in the cardiac tissue of patients with CCCD^165 , 166^and in the murine acute infection by *T. cruzi* .^167^

In addition, the discoveries in *T. cruzi-* infected mice genetically deficient in microRNA-155 have supported the relationship of miRNA with infection control and production of inflammatory cytokines.^168^

### 3.4. Genetics in Chronic Cardiomyopathy of Chagas Disease

Finding that approximately 30% of the patients with CD develop chronic cardiomyopathy and the presence of family cases of CCCD^169^have suggested the participation of genetic factors in the different progressions of the disease. Patients with CCCD have more intense inflammatory response than those with the IFCD, who seem to have a better regulated immune response.

Given the importance of the inflammatory mechanisms in the pathogenesis of CCCD, several studies have focused the common or frequent polymorphisms in the genes related to inflammatory and immune responses, thus causing important variations in the expression of inflammatory cytokines and chemokines involved in the disease’s pathogenesis. Each common or frequent polymorphism is typically responsible for small phenotypic effects (approximately 10% of the population/phenotype).

A recent review of 145 association studies on candidate polymorphisms in 76 genes has revealed 62 single nucleotide polymorphisms (SNPs) of 44 genes to be associated with the CCCD phenotype.^115^Of those, SNP in 8 genes were associated with the severity of CCCD: SNPs in genes IL17a, IL18, IL27b/Ebi3, CCR2, CXCL9, CXCL10, and MICA were more frequent in patients with CCCD and significant left ventricular (LV) dysfunction (LVEF < 40%) as compared to other patients with CCCD.

Two genome association studies were performed using the GWAS (Genome Wide Association Study) technique, comparing CCCD and IFCD, one in 2013,^170^involving 600 patients with CD, and the other in 2021, involving 3413 individuals.^171^Only the latter revealed a significant single variant in the entire genome (p < 10^- 8^) close to the SAC3D1 gene.

A recent study has assessed the role of rare gene variants in the progression to CCCD in nuclear families with multiple cases of CD by using whole exome sequencing.^172^In the six families studied, 22 rare high-impact non-synonymous heterozygous pathogenic variants were found associated with CCCD, located in 20 genes. Only seropositive individuals with the pathogenic genetic variants developed CCCD, but neither seropositive individuals without the genetic variants nor seronegative siblings with the pathogenic genetic variants did. There was a significant accumulation of specific variants of CCCD (86%) in mitochondrial or inflammation-related genes. In addition, all families studied showed at least one gene variant linked to CCCD. The results of that study indicated that the gene contribution to cause CCCD is polygenic and mediated by several rare genetic variants that differ in the families, being related to mitochondrial changes and inflammation.

The results indicate that mitochondrial dysfunction and inflammation, key-processes in the CCCD pathophysiology, are at least partially genetically determined. This can depend on a double-aggression mechanism. Thus, IFN-γ and proinflammatory cytokines induced by chronic infection would trigger mitochondrial dysfunction and clinical disease in patients with gene variants that cause subclinical impairment of mitochondrial function in organs of high metabolic demand, such as heart and myenteric ganglion neuronal cells. Mitochondrial lesion can be the mechanism that perpetuates the tissue inflammatory changes because of the release of inner components of damaged mitochondria by innate immune response. Figure 3.2 shows the highlights of the inflammatory key points associated with CCCD progression.

**Figure 3.2:** – Interactions between immune, microvascular, and neurogenic mechanisms in the chronic cardiomyopathy of Chagas disease.

### 3.5. Coronary Microvascular Disorder

There is increasing evidence, both clinical and experimental, of coronary microvascular abnormalities in the pathogenesis of CCCD. Several studies have indicated that the myocardial damage might result from microvascular changes mainly associated with inflammation, which lead to myocardial ischemia and necrosis with occasional reparative fibrosis.^173 - 176^

The first evidence that coronary microcirculation disorders may play a role in the myocardial damage of CD in humans has been obtained from postmortem studies describing severe vascular changes, with intimal hyperproliferation, wall thickening, and obstruction of the small intramural coronary arterioles in hearts of patients with CCCD.^177 , 178^In addition, the myocardial fibers in the proximity of the vascular lesions showed myocytolysis, a cellular lesion closely related to myocardial ischemia.

In a more recent study, Higuchi *et al* . have described coronary microcirculatory changes with vascular dilation and rarefaction in hearts of patients with CCCD, which differed from those usually observed in patients with idiopathic DCM.^179^

Thus, observations from postmortem studies strongly suggest the participation of microvascular ischemia in the genesis of the inflammatory foci and myocytolysis, which lead to reparative fibrosis, the fundamental histopathological features of CCCD.

In the clinical scenario, studies using myocardial perfusion scintigraphy have shown high prevalence (30% to 50%) of perfusion defects in patients with CCCD and angiographically normal coronary arteries, strongly suggesting the presence of coronary microvascular dysfunction.^180 - 183^In addition, several studies have shown that the myocardial perfusion defects were topographically related to LV wall motion impairment occurring in patients in the early phases of CCCD with no other evidence of cardiac impairment,^183^suggesting that microvascular ischemia is an early disorder in the disease progression, preceding regional ventricular dysfunction and possibly related to hibernating or stunned myocardium induction. Similar results were obtained in studies with doppler ECHO, showing reduction in the coronary vasodilation reserve, a microvascular dysfunction index, in patients with the IFCD as compared to normal controls.^184^

A retrospective longitudinal study using myocardial perfusion scintigraphy in patients with CCCD has shown that microvascular ischemia is topographically related to areas that develop myocardial fibrosis during the disease progression. These results support the hypothesis that microvascular ischemia can be directly involved in the mechanism that leads to regional fibrosis and LV systolic dysfunction progression in CCCD.^181^

More recent studies in an experimental model of Syrian hamsters chronically infected by *T. cruzi* have shown, using high-resolution myocardial perfusion scintigraphy *in vivo* , a close topographic relation between myocardial perfusion defects at rest with histological evidence of inflammation and regional/global LV systolic dysfunction.^185^In addition, another study with that experimental model has confirmed, by use of positron emission tomography computed tomography (PET/CT) with^18^F-fluorodeoxyglucose (^18^F-FDG), that the regions with myocardial hypoperfusion at rest corresponded to areas with viable myocardium and inflammation.^186^

Another recent study with the same model of hamsters chronically infected by *T. cruzi* has shown that the prolonged use of dipyridamole, a vasodilating agent of the coronary microcirculation, was associated with a significant reduction in myocardial perfusion defects at rest, indirectly supporting the presence of viable, but hypoperfused myocardium caused by coronary microcirculation dysfunction in experimental CCCD.^187^

The mechanisms potentially involved in the genesis of coronary microvascular dysfunction in CCCD are: 1. Functional changes in the coronary tree, with increased vasoreactivity and spasm of small intramural arterial branches;^188 , 189^2. Endothelial lesions caused directly by parasitic aggression;^190^3. Functional and structural changes induced by substances secreted by the inflammatory infiltrate in the myocardial tissue close to the coronary microvessels, mainly endothelin and cytokines. This late mechanism is supported by studies evidencing that myocardial inflammation changes are associated with the occurrence of platelet plugs, obstructive proliferation of the vascular intimal layer, and microarteriolar spasm.^191^

### 3.6. Cardiac Denervation

Autonomic cardiac denervation is an important characteristic of CCCD and was first described in human postmortem studies showing intense intramural neuronal depopulation, greater than that observed in any other cardiovascular disease.^192 , 193^This discovery has been confirmed in studies with experimentally *T. cruzi-* infected animals, showing cardiac neuronal parasitism associated with periganglionitis and degenerative abnormalities in Schwann cells and nervous fibers.^194 , 195^It is worth noting that the neural depopulation also affects the intramural ganglia of several digestive system organs, mainly the esophagus and colon, which is clearly implicated in the pathophysiology of megaesophagus and megacolon of CD.

Neuronal depopulation in CCCD has been postulated to occur during the acute phase of the infection, secondary to the direct parasitism of neurons, the degeneration caused by periganglionic inflammation, and the antineuronal autoimmune reaction.^196 , 197^There is evidence that the damage can continue in the chronic phase because of the localized inflammation.

Several functional abnormalities of the autonomic reflex control of heart rate (HR) in patients with CCCD have been described as a consequence of the anatomically detected autonomic cardiac denervation.^198 - 201^Patients with CCCD have deprivation of the tonic inhibitory action of the parasympathetic system in the sinus node and also lack of the vagal-mediated mechanism to respond with rapid bradycardia or tachycardia to transient blood pressure or venous return changes.^202^Dysautonomia in patients with CCCD can be detected before the development of ventricular dysfunction, as well as in the early stage of the chronic phase and even in the indeterminate and digestive forms of CD.^203 , 204^

More recently,^123^I-MIBG myocardial scintigraphy has been used in patients with CD to provide accurate information on the integrity of the sympathetic nervous fibers within the LV myocardium.^183^In that study, 37 patients were investigated by use of^123^I-MIBG imaging and the results were correlated with myocardial perfusion and regional LV wall motion impairment. Defects in^123^I-MIBG uptake were observed in most patients: 33% of those with a normal ECG and normal ECHO and 77% of those with regional LV wall motion impairment. In addition, patients with more severe ventricular dysfunction had a higher prevalence of defects in the^123^I-MIBG uptake (92%). There was a clear topographic correlation of the myocardial sympathetic denervated areas, perfusion myocardial defects, and regional LV wall motion abnormalities.

Another study using^123^I-MIBG scintigraphy has shown strong topographic concordance between myocardial sympathetic denervated areas and myocardial hypoperfusion areas during stress.^205^Those results indicated that sympathetic denervation is an early disorder in the CCCD pathophysiology, preceding the development of LV regional contractile abnormalities or global contractile dysfunction. This hypothesis has been corroborated by the results of an independent study evidencing abnormal^123^I-MIBG absorption in most patients with CD without any sign of cardiac involvement.^206^

In addition, clinical studies have reported a quantitative relation between the extension of myocardial denervation, using^123^I-MIBG imaging, and the risk for malignant ventricular arrhythmias. This is a clinically relevant aspect because it associates the presence and extension of sympathetic denervation with severe arrhythmia in patients with CCCD and it is potentially implicated as the mechanism of sudden death.^207 , 208^

Despite the extensive documentation of conspicuous autonomic denervation in the early stages of CD and the recent demonstration of its potential participation in the mechanism that triggers severe ventricular arrhythmias, the “neurogenic theory” still lacks demonstration of the pathophysiological links between those phenomena and the essential myocardial lesions in CCCD.

Autonomic denervation has also been proposed to be associated with coronary microvascular spasm and trigger myocardial ischemia, leading to myocardial necrosis. However, that mechanism still awaits more clear evidence. Figure 3.2 shows the interaction of inflammation with microvascular and neurogenic mechanisms.

### 3.7. Final Considerations

The pathogenesis of CCCD is still an enigma consisting of multiple complex aspects related to the variety of pathogens, as well as to the host’s genetics and immune system, as shown in Figures 3.1 and 3.2 . In addition, there is recent evidence that the several gaps in the knowledge about the parasite’s life cycle in the human host and vector should be revisited and clarified, enabling the identification of more appropriate targets for more effective therapies, as well as their use in guided research.^209^

## 4. Cardiomyopathy Pathophysiology - Acute and Chronic Phases

### 4.1. Introduction

The pathophysiology of CCD can be briefly described as follows. In the acute phase, most *T. cruzi-* infected individuals can progress with diffuse myocarditis of low intensity, which does not associate with severe cardiovascular disorders, being not even diagnosed. In rare patients, acute inflammation can lead to a significant loss of myocardial contractility, with chamber dilatation and HF with biventricular ejection fraction reduction, sometimes with concomitant electrical disorders (conduction blocks, extrasystoles) and pericardial effusion. Such changes are usually self-limited to a few weeks, not causing clinically manifest sequelae.

Cardiac damage in the chronic phase, however, results from fundamental changes (inflammation, necrosis, and fibrosis) caused directly or indirectly by *T. cruzi* in the specialized conduction tissue, in the contractile myocardium, and in the intramural autonomic system.

The frequent impairment of sinus and atrioventricular nodes, as well as of the His bundle, due to inflammatory, degenerative, and fibrotic changes leads to sinus dysfunction and varied atrioventricular and intraventricular blocks. By being more individualized structures, the right branch and the left anterosuperior fascicle are more vulnerable and more frequently damaged. Inflammatory foci and fibrotic areas in the ventricular myocardium, especially in apical, posterolateral and inferobasal regions, can produce electrophysiological changes and favor the appearance of reentry, the major electrophysiological mechanism of malignant ventricular tachyarrhythmias, which cause sudden death even in patients without previous HF and without severe LV systolic dysfunction.

Another very common consequence of myocardial lesions is biventricular dysfunction, characteristic of CCCD. Initially, there is regional impairment, similar to that occurring in the cardiopathy due to coronary obstruction, but dilation and generalized hypokinesia, usually of both ventricles, gradually occur, conferring the hemodynamic pattern of DCM to CCCD. In more advanced phases of the natural history, there are global cardiac dilation and marked cardiac mass increase, due to the combination of myocardial hypertrophy and fibrosis at degrees varying from patient to patient.

From the earliest phases, dyssynergies or ventricular aneurysms predispose to thromboembolic complications. In advanced stages, global dilation, venous stasis, and AF are additional factors that propitiate thrombus formation and consequent pulmonary and systemic embolism, such as in the central nervous system, where they cause stroke. In addition to malignant arrhythmias and refractory HF, CCCD can cause embolism to the pulmonary circuit and several systemic organs, resulting in renal, splenic, and mesenteric infarctions, or embolism to the arteries of the limbs.

Such characteristics of the CCCD pathophysiology can be understood as resulting from important pathogenic mechanisms, such as those approached in the chapter of pathogenesis, with the additional emphasis on aspects described in the next section.

### 4.2. Myocardial Parasitism and Immune Response

The infectious-parasitic disease caused by *T. cruzi* , CD, has its natural history divided into acute and chronic phases.^37 , 46^The acute phase is usually oligosymptomatic with unspecific symptoms, but 5-10% of the cases can have more expressive symptoms in the presence of intense parasitemia,^210^with fever and lesion in the pathogen inoculation site. It can complicate with meningoencephalitis, myocarditis, and other manifestations.

Four to eight weeks after the infection, when the parasitemia drops to undetectable levels and the acute phase symptoms disappear, begins the chronic phase, which usually lasts for several decades. In the chronic phase, 60-70% of the individuals have no symptom, and the routine additional tests related to the heart and digestive system show no abnormality. That is the IFCD.^211^The other chronically infected patients develop the determined forms, with cardiac and/or digestive impairment.^46 , 212^

#### 4.2.1. Immune Response in the Acute Phase

Since the acute phase, CD has a multifactorial pathophysiology, in which the immune and primary inflammatory (triggered by *T. cruzi* itself) mechanisms play a fundamental role.^213 , 214^

In the acute phase, there is exposure of the *T. cruzi* surface molecules to the receptors of macrophages and dendritic cells, causing the activation of innate immunity cells, such as neutrophils and NK lymphocytes (natural killers), which will trigger intense inflammatory response aimed at controlling parasitemia. Innate immunity activation generates intense secretion of pro-inflammatory cytokines, such as TNF-α, IFN-γ, and several interleukins, especially IL-10.^119 , 215^That intense inflammatory response resulting from the activation of innate immunity cells and production of pro-inflammatory mediators, although crucial to control the infection, contributes to cause direct lesion to the cardiomyocytes – also attacked by the usually conspicuous tissue parasitism. This pathophysiologic set represents the typical diffuse myocarditis of the acute phase of CD, which, in most cases, has a benign and self-limited course.

After the intense inflammatory phase, causing a reduction in parasitemia and tissue parasitism, the macrophages and dendritic cells that phagocyted *T. cruzi* trigger the humoral and cellular immune responses, with activation of B and T lymphocytes. The chronic phase, thus, begins in most patients who did not eliminate the parasite in the acute phase window of opportunity.^216^

#### 4.2.2. Immune Response in the Chronic Phase

The presence of the parasite’s DNA in the myocardium,^149 , 217^as well as the cross-recognition by CD4+ T cells of *T. cruzi* antigens and amino acid sequences of cardiac myosin are important aspects of the pathophysiology of myocardial dysfunction during the chronic phase.^218^

Regarding the cellular immune response, infected macrophages present *T.cruzi* antigens from cross-reaction with the heart to CD4+ T lymphocytes, which migrate to the heart, producing inflammatory cytokines that increase the recruiting and activation of immune system cells, triggering late hypersensitivity reaction. Of the inflammatory cytokines, TNF-α and IFN-γ are significantly increased in patients with CCCD.^119 , 124^

A recent study has directly compared the T lymphocytic subpopulation in individuals with CCCD and idiopathic DCM, evidencing a clear difference in the immunoregulatory profile and higher immune activation in CCCD, although those two conditions have similar hemodynamic characteristics.^219^

Several factors are implicated in the etiopathogenesis of CD in the heart, but, regardless of the major mechanisms of tissue damage, the common final pathway is intense inflammatory infiltrate and reactive and reparative myocardial fibrosis. The structural, geometrical, and functional cardiac disorganization essentially results from myocardial necrosis and consequent replacement by fibrous tissue, damaging the perivascular and interstitial content, important histopathological markers of CD.

Such alterations are sufficient to cause dilation and consequent biventricular contractile dysfunction, and the myocardial fibrosis is far more intense as compared to that of other cardiomyopathies. Complex mechanisms activate the cascade of cellular and molecular response, intensifying the inflammatory response, oxidative stress, and progressive loss of cardiomyocytes through necrosis and/or apoptosis, in addition to promoting overload and further dysfunction of the residual myocardium.^200 , 220^

## 4.3. Autonomic Nervous System Alterations in Chagas Disease: Evidence from Histopathological Studies

Anatomical pathological and functional changes of the autonomic nervous system, at various severity levels, have been described in humans and experimental animals since the initial studies by Carlos Chagas and collaborators.^176 , 200 , 221 - 229^Such changes have been reported to be more conspicuous in patients with CD as compared to that occurring, at a lower level, in other cardiomyopathies.^230 , 231^However, despite being one of the most remarkable aspects in the CD pathophysiology, the actual etiopathogenetic role of those changes, including those described in the inter-trunk cardiac plexus, remains uncertain.

Directly dependent on the *T. cruzi* infection, those changes, such as ganglionitis, periganglionitis, neuritis, and perineuritis, cause a significant ganglionic density reduction and neural depopulation in experimental animal models^223^and patients with CD.^224^Based on studies with experimental models, those anatomical pathological changes in the inter-trunk cardiac plexus have been postulated to occur predominantly during the acute phase of the infection^225 , 226^and to continue in the chronic phase at lower intensity.^227 , 228^Such changes result from four factors acting in isolation or combination: direct parasitism of neurons,^229^intense periganglionic inflammatory process,^221^autoimmune antineural reaction,^232^and periganglionic microvascular dysfunction.^233^

Damage to the autonomic structures can be partially compensated, because the autonomic neurons, within limits, maintain a certain ability to functionally recover.^227^In addition, sympathetic reinnervation has been reported in humans during the chronic phase of CD after procedures, such as cardiac transplantation (CTX)^234^and stem-cell therapy.^235^

However, restoration of the functional neuroeffector junctions, due to axonal regeneration during the chronic phase, is disorganized, random, and incomplete. The parasympathetic innervation has a similar behavior: there is marked destruction of nervous fibers, with a reduction in the cardiac acetylcholine levels during the acute phase, followed by disorganized, random, and incomplete functional restoration during the chronic phase.^227^Several physiological and pharmacological tests have evidenced abnormal functional responses, supporting that pathophysiological hypothesis.^161^

Neuronal depopulation occurs predominantly in the parasympathetic intramural ganglia of the heart and myenteric plexuses.^221 - 228^Thus, a theory was initially formulated that a ‘parasympathetic-deprived’ cardiopathy would install in the heart, in other words, there would be an actual “cardioneuropathy induced by a relative non-antagonized excess of catecholamines”.^221 , 223^According to that pathophysiological theory, the heart, unprotected due to the absence of the moderator parasympathetic effect, would be subjected to the stress of the intense toxic stimulation of the adrenergic system.

However, several pieces of evidence hinder the confirmation that a “catecholamine-induced cardioneuropathy” decisively contributes to the pathogenesis of the cardiac form of CD. In contrast, it is virtually impossible to eliminate the possibility of the involvement of that mechanism in the process. Even more important, there would be signs that the vagal-cholinergic pathway plays a fundamental direct role in preventing the cardiac impairment that occurs in CD.^229^

Among the obstacles to the ‘parasympathetic-deprived’ theory and despite the predominant vagal dysfunction, there is the finding of the concomitant attenuation of the adrenergic regulation of the sinus node-mediated cardiac chronotropism.^176 , 236^Furthermore, at the myocardial level, sympathetic denervation is described in I-MIBG scintigraphy of the heart.^123^Disorders in that radiotracer uptake, which reflects the adrenergic integrity at ventricular level, tend to intensify as the disease progresses.^205^Those studies evidence strong association between the sympathetic denervation areas, wall motion abnormalities, and myocardial hypoperfusion in many patients, contributing to the installation of potentially fatal arrhythmias. Studies on *T. cruzi* infection in humans and experimental models with Syrian hamsters have suggested that the sympathetic autonomic denervation and microvascular dysfunction are closely related and active in the initial stages of CCCD.^183 , 205^

Additional aspects related to the complex dysautonomic pathophysiology observed in CD involve the so-called anti-inflammatory cholinergic pathway. The conceptual base resides in the evidence that the inflammatory process in CD influences and is influenced by the immune-mediated autonomic balance.^237 - 239^Thus, attenuation of the cytotoxicity of T lymphocytes by cholinergic-muscarinic stimulation was observed, and afferent and efferent signs that would compose an arch, the “neuroimune” or “inflammatory” reflex, were postulated.

According to that concept, the nervous and immune systems communicate in a two-way manner and use that interaction to mediate the cytokines and neurotransmitters they have in common. The efferent pathway of the central nervous system would act on the immune system through its parasympathetic component, making the anti-inflammatory cholinergic pathway. The parasympathetic system innervates the organs of the immune system and its mediator, acetylcholine, acts on that system cells, especially macrophages, by activating the acetylcholine receptor.^240^In the CD context, it was hypothesized that the cardiac parasympathetic tonus depression could contribute to exacerbate inflammation during the chronic phase, a pathophysiological conception that goes back to the beginning of the investigations about CD.^229^

The mechanisms that induce autonomic dysfunction in CD include the production of circulating autoantibodies, particularly against cholinergic receptors (Ac-M), as well as against adrenergic receptors (Ac-β).^240^Such antibodies are postulated to result from antigenic mimicry (cross-reaction between the P ribosomal protein of *T. cruzi* and the human ribosomal protein),^241 - 243^and the disorders mediated by circulating autoantibodies can confer particular characteristics to the CD dysautonomia as compared to other neuronal affections.^233 , 244 - 246^

## 4.4. Chagas Disease Pathophysiology Dependent on the Parasite and Human Host Genetic Characteristics

The development of an infectious disease is usually a complex phenomenon related to several factors of the environment, the infectant pathogen, and the host. Thus, the assessment of the genetic characteristics of the host and pathogen can contribute to solve the enigma: why approximately 30% of the infected individuals develop CCCD, while the rest remains asymptomatic and without clinical manifestations for the entire life.

The genetic diversity of *T. cruzi* recognizes seven discrete typing units (DTU), TcI-TcVI and Tcbat.^247^This genetic diversity is a potential target for innovation in new trypanocidal drugs.^248^

Recent research has indicated that the parasite strains detected in patients, regardless of their clinical presentation, reflect the major circulating DTU in the domestic transmission cycles of a certain region. Recent systematic review and meta-analysis of *in vitro* studies has evidenced that, despite the preliminary signs of relevant differences in the parasite’s sensitivity to the etiological treatment, there is significant heterogeneity in the results, even considering only studies on the sensitivity of several *T. cruzi* DTU to a single trypanocidal drug, benznidazole, which hinders the accurate identification of more and less sensitive parasite’s strains to treatment.^249^

In several studies of micro-outbreaks of orally transmitted parasites, wild strains are implicated. Because of the genotypic and phenotypic differences of *T. cruzi* strains and the different geographical distribution of DTU in humans, there are regional variations in the sensitivity of the serological tests, causing potential implications in the response to parasiticide treatment options.^250^

Such genotypic characteristics have been recently summarized to clarify their potential associations with clinical manifestations of CD, emphasizing the persistence of significant uncertainties regarding knowledge as well as of relevant challenges in those research lines.^251^

Similarly, studies of genetic polymorphism have focused the host’s characteristics that influence the development and severity of the clinical presentations. In that context, the SNPs are defined when at least two alternative nucleotides occur in the genome at significant frequency (usually > 1%). The SNPs show Mendelian inheritance and are used as genetic markers.^252^

Several studies have assessed the human genetic polymorphism and included correlations with elements of the immune response, adaptive and regulatory, during *T. cruzi* infection.^253^The TNF polymorphism is one of the most studied in CD. In Brazil, a reduction in the survival of patients with the TNF-308A allele or TNFa2 microsatellite allele has been reported,^254^but no association was found between the TNF-308 polymorphism and the CD clinical presentations.^255^Similarly, another study in Peruvian patients, comparing those with CD *versus* control individuals without *T. cruzi* infection, has shown no major association of the TNF-308, -244, and -238 polymorphisms with CD.^256^

In contrast to that described for mediators related to the immune profile, the genetic assessment related to the angiotensin-converting-enzyme system evidenced some disagreement, but the DD genotype has been associated with a higher risk for HF and mortality in the myocardial disease of ischemic etiology.^257^

In another cohort study, in HF due to idiopathic cardiomyopathy, the DD genotype has been shown to remain a predictor of mortality.^258^In two different populations with CD, including a Brazilian one, no valid association regarding those polymorphisms has been observed.^259 , 260^However, in another population from the Brazilian northeastern region, a higher prevalence of the I/D polymorphism has been reported in patients with HF as compared to asymptomatic patients with CD.^261^Such discrepancies can be due to the fact that final phenotypes are considered dependent on environmental factors,^262^requiring large samples to demonstrate effects of the genes involved in complex traces, such as those in the complicated clinical syndromes, such as HF of CD etiology.

More recent studies on genetic aspects and using GWAS technology have involved bigger samples and generated more consistent and relevant information. For example, previously the SNPs have not been strongly associated with CCCD.^170^But a recent meta-analysis has revealed the association with CCCD development in rs2458298, nearby the SAC3D1 gene, indicating the host’s genetic variability as a susceptibility factor to CCCD development after *T. cruzi* infection.^171^

## 4.5. Peculiar Histopathology of Chagas Disease

In the acute phase of the infection, the parasite’s adhesion to and penetration into the host’s cells occur via lecithins that bind to carbohydrate residues attached to the host cell membrane, mainly the sialic acid. Recent review about the family of human galactoside-binding proteins, called galectins, has reported on their significant participation in the innate and adaptive immunomodulation to *T. cruzi* infection, with potential pathophysiological and therapeutical implications.^263^

Inside the host’s cells, trypomastigote forms transform into amastigote forms, but, for as long as the parasitized cells remain intact, no local inflammatory reaction is observed. When the parasitized cell ruptures, epi-, trypo-, and amastigote forms of the parasite, intact or degenerated, are released, along with cell components that act as immunogens (mitochondria, myofibril remnants), to the extracellular medium, stimulating the presence of inflammatory mediators, which cause vasodilation and increase vascular permeability, factors typically implicated in the inflammatory process exacerbation.

In the early acute phase, the inflammation is focal, topographically associated with intense parasitism, and can coalesce and become diffuse. In contrast, the chronic phase is more obscure and complex, because, although there is active inflammatory reaction, the parasitism is scarce and cannot completely explain the inflammatory foci. Thus, the hypothesis of late hypersensitivity and autoimmunity has been raised to explain the maintenance of inflammation and lesions in the chronic phase of the disease by: (1) molecules of the parasite and of the cardiomyocytes are structurally similar, which could explain common antigenic properties and immune cross-reaction: *in vitro, T. cruzi-* sensitized lymphocytes have a cytotoxic action against cardiomyocytes; (2) the mononuclear inflammatory infiltrate and occasional formation of granulomas suggest the possibility of late hypersensitivity reaction.

These more controversial pathophysiological aspects of the inflammatory lesions of the CCCD have been partially clarified in recent studies using more sensitive tests to detect the parasite. Such tests suggest that, even scarce, the parasite persistence in the tissues is a continuous source of antigens, which can mediate the low-grade, but incessant, inflammatory response.

Techniques of molecular biology, such as polymerase chain reaction (PCR), applied to myocardial fragments of patients with CCCD have shown *T. cruzi* DNA in inflammatory foci in almost all cases studied. In addition, the accumulation of CD8+ T lymphocytes, which predominate in chronic myocarditis, correlates with the focal presence of parasite antigens. In addition to the degenerated trypanosomes, the cell rupture can cause the release of microorganisms that were in the *T. cruzi* cytoplasm. This hypothesis is based on the observation of endomyocardial biopsies of patients with CCCD showing electron-lucent microparticles and nanovesicles containing DNA from archaea – the oldest microorganisms in nature that can parasitize trypanosomes – in the region of inflammatory foci.^264^

Archaea, numerous in the serum of patients with HF due to CD, are associated with inflammation, because they uptake interstitial proteins and generate immune response with CD8+ T lymphocytes, with no response of CD4+ T cells. Lipidic archaea are increased in the IFCD, as well as protecting exosomes that capture AMZ1 (archaea-specific metalloprotease) from the external medium, preventing the activation of enzymes and protecting against collagen degradation and inflammation. Thus, according to that hypothesis, archaea could play a fundamental role in the myocardial inflammation and microcirculation dilation.^265^

Still, from the histopathological viewpoint, former studies have evidenced that *T. cruzi* has tropism for the adipose tissue, which can be another pathophysiological link of the extensive inflammatory changes present in the chronic phase and explored as a therapeutic target.^266 - 268^

## 4.6. Coronary Microcirculation Injury

Several clinical manifestations in patients with CCCD mimic those of coronary obstructive disease: 30-40% of those patients have chest pain, which is usually atypical with no clear relation to physical exertion and variable duration, for long symptomatic periods. Those patients’ ECG usually show ST-T alterations and areas of electrical inactivity, simulating changes due to ischemia and/or myocardial infarction. More characteristically, patients with CCCD usually show ventricular wall motion abnormalities similar to those due to necrosis and infarction associated with coronary obstructions. Finally, several myocardial perfusion disorders have been described in the different phases of the CCCD.

Of note, all those structural and functional changes are found in the presence of angiographically normal subepicardial coronary arteries with no early detectable atherosclerosis on computed tomography angiography.^269^These pathophysiological changes are attributed to coronary structural and regulatory abnormalities at microvascular level. Histologically, extreme vasodilation, not seen in other DCMs, has been described, with a reduction in distal perfusion pressure, myocytolysis, and ischemia in borderline regions of double coronary irrigation (vascular watershed zones, such as the *crux cordis* region, in which the septal artery, a branch of the anterior descending artery, competes with a branch originating from the right coronary artery), postulated as more susceptible to ischemia.^270^Such ischemic lesions are believed to contribute to the installation of akinetic areas and ventricular aneurysms, such as apex thinning and typical inferolateral fibrosis frequently detected as the origin of sustained ventricular tachycardia (SVT).

A common consequence of those microcirculatory disorders is fibrosis, which develops slowly and progressively, with interstitial deposition of fibronectin, laminin, and collagen, leading to expansion and distension of the extracellular matrix, contributing to the progressive loss of myocardial contractile activity and appearance of cardiac arrhythmias. There is no other human myocarditis in which fibrosis develops so intensely and with so peculiar characteristics as that of the CCCD.^271^

## 4.7. Potential Therapeutic Applications of Pathophysiological Targets in Chronic Cardiomyopathy of Chagas Disease

Several recent investigations have focused on some pathophysiological changes that can be therapeutic targets to favorably influence the *T. cruzi-* infection natural history or even CCCD. The parasite life cycle itself, through new knowledge of its interaction with the human host, and the vector as an intermediate host, with the better understanding of its genetic characteristics, can be revisited to assess the therapeutic possibilities of the trypanocidal effect.^209^

However, the most recently identified perspectives reside in the possibility of inflammatory response modulation. In the IFCD and CCCD, several mechanisms of inflammatory activation of IL-1Beta have been shown.^143^Thus, after a preclinical study evidencing a reduction in fibrosis with the transforming growth factor-beta inhibitor,^157^that cytokine antagonism has become an important therapeutic target in the context.^272^

In addition, there is evidence that the clinical forms of CD (IFCD and CCCD) involve different subpopulations of CD4- and CD8- immune memory cells, raising the possibility of a new anti-inflammatory strategy to control CD in the heart.^273^An extensive analysis of several aspects hypothetically related to multiple strategies to control the parasite and its inflammatory consequences has been recently reported to improve the prognosis of infected individuals.^274^

In another line of research about natural drugs with strong anti-inflammatory and antioxidant activity, such as curcumin and resveratrol, the results with experimental animals have been reviewed, encouraging future initiatives in humans.^275^A pioneer randomized study with a small sample of 37 patients with CCCD has assessed the therapy with granulocyte-colony stimulating factor (G-CSF), which is clinically used to support chemotherapy and bone marrow transplantation and has shown promising results in *T. cruzi* -infected mice. That study has reported good tolerability to treatment for 1 year, suggesting that further studies with G-CSF should be conducted in humans with CCCD.^276^

Finally, the genetic polymorphism, which pathophysiologically regulates the levels of pro- and anti-inflammatory factors (as exemplified by IL-10), has been recently revisited in a meta-analysis of several studies with some subpopulations of *T. cruzi* -infected individuals. The aim was to identify biomarkers to predict the risk of developing CCCD and to monitor the evolution and therapeutic interventions in that context.^277^

## 5. Natural History

### 5.1. Acute Myocarditis of Chagas Disease

The incidence of the acute myocarditis of CD depends on the parasite load and strain, host, and infection transmission mode (oral or classic vectorial mainly). Depending on the diagnostic tool, the detection of myocarditis can vary from 40% to 100% in the acute phase of *T. cruzi* infection.^278 - 281^

As already discussed in the chapter on the pathogenesis of CD, the anatomical pathology of the acute phase is directly related to the parasitism of the cardiac cells, the inflammatory reaction immediately evoked by the infectious process, and the consequent microcirculatory impairment.^282^There are inflammatory lesions in the myocardium, endocardium, pericardium, and intramural autonomic nervous system of the heart and several other organs, similarly to those observed in viral myocarditis. The hematoxylin-eosin and Giemsa stains can easily evidence amastigote forms of the parasite.^283 , 284^

A characteristic finding is the presence of small nodules lined up as beads in a rosary, which is called moniliform epicarditis. Despite the true pancarditis, the cardiac valves, typically avascular structures, are often spared. The severity of the cardiac lesions is influenced by the infection transmission mode (oral or classic vectorial). In most cases, the infection has a benign course, being virtually oligosymptomatic, or, less often, very severe, leading to death.^283 - 285^

Recently, the most studied clinical aspects have been those related to the myocarditis caused by oral *T. cruzi* transmission (ingestion of foods not sanitarily prepared and contaminated by macerated triatomine bugs or by their excrementa). Subclinical aspects have been frequent. Acute inflammation can begin right before the fever subsides, which usually occurs within 15 to 20 days from the disease beginning.^2^

Some patients, similarly to that occurring in viral myocarditis, can manifest chest pain, dyspnea, and palpitations, sometimes imitating coronary artery disease symptoms.^286^Tachycardia is usually present, and, in most severe cases, symptoms and signs of acute HF can be observed, particularly the C hemodynamic profile (poor tissue perfusion and pulmonary and/or systemic congestion).^287^The ECG shows unspecific ventricular repolarization changes, low-voltage QRS complexes, supra- or ventricular extrasystoles, and even sustained ST-segment elevation. Atrioventricular or even intraventricular conduction disorders, common in the chronic phase, are less frequent in the acute myocarditis.^279 , 288^

The ECHO frequently detects pericardial effusion of variable proportions, with diffuse hypocontractility of both ventricles, which is an attribute of the most severe cases of myocarditis.^289^

The most accepted hypothesis for the fatal outcome of a significant number of orally infected patients is due to large inoculation, with ingestion of a high parasite load, in addition to the easy intense penetration of parasites through the gastrointestinal mucosa, very permeable to *T. cruzi* .^48 , 290^In a study involving 126 individuals aged < 18 years, most of whom (68.3%) diagnosed with the acute form after oral transmission and followed up for 10.9 years, the evolution was considered benign, although 2.4% persisted with cardiac changes.^291^

The natural history of the acute phase of CD caused by the classic vector transmission (excrementa of the hematophagous bug) includes a high number of individuals whose infection is not even diagnosed because they are asymptomatic or oligosymptomatic and progress to practically spontaneous remission. In a reduced number of cases, the acute *T. cruzi* infection can be fatal (estimated as 3-5% of the symptomatic cases, due to myocarditis and/or fulminant meningoencephalitis).

The natural history of the acute phase myocarditis caused by classic vectorial transmission^292 , 293^is less clear than that registered for the micro outbreaks recently observed after oral transmission. However, it is evident that more symptomatic cases have unfavorable outcomes because of their association with a more intense acute myocarditis.^294^But, most importantly, the large majority of individuals acutely infected by *T. cruzi* usually progress to the chronic phase, being characterized as having the IFCD.

The natural history of the acute myocarditis of CD and IFCD is yet to be clarified. Some studies have properly assessed that evolution. However, several influences can bias the results, such as the affected population’s age range, transmission mode, parasite load and strain, follow-up duration, and previous etiological treatment, which can completely and favorably change the natural history.

A cross-sectional study conducted in the municipality of Bambuí, Minas Gerais state, in the 1940-1950 decades, on the acute phase of CD diagnosed after classic vectorial transmission, has reported 8.3% of case-fatality in the acute phase of children < 10 years of age. Of 130 individuals followed up from 1 to 3 years after the acute phase, 71.5% showed no ECG changes and 30% had normal cardiac dimensions. After 3 to 5 years, those numbers were 65.7% and 87.5%, respectively. It is worth noting that the population sample was basically formed by children in the post-world war-II period, who received no etiological treatment.^293 , 295 , 296^

A more recent study on the natural history of CD has assessed two groups of patients: one followed up since the diagnosis in the acute phase and the other followed up from the IFCD onward. The study has assessed the risk of developing CCCD by use of systematic review and meta-analysis of 32 studies. The following were considered for the diagnosis of CCCD: appearance of arrhythmias or ECG changes; evidence of ventricular contractile abnormalities on ECHO; and death associated with CD. After the acute phase, the estimated annual risk of progression to chronic cardiopathy was elevated, 4.6% (95% CI, 2.7%-7.9%; I2 = 86.6%; τ2 [ *ln scale* ] = 0.4946). For the individuals followed up from the IFCD on, that risk was 1.9% (95% CI, 1.3%-3.0%; I2 = 98.0%; τ2 [ *ln scale* ] = 0.9992).^297^

Observational studies characterize myocarditis either as a potential underdiagnosed cause of acute HF that could progress to sudden death or, more commonly, as DCM. The prognosis is considered good in the short term for the group of individuals with no clinical manifestation of cardiac impairment, or when there is complete remission of the biventricular myocardial depression. There is no evidence of how many patients with clinically detected myocarditis in the acute phase recover ventricular function, but develop DCM in the long term. However, much older studies have reported a considerably poorer prognosis of patients whose acute phase was manifested and disease diagnosed than that of patients whose acute myocarditis passed unnoticed.^293 , 295 , 296^

There is evidence through anecdotal cases and WHO records that some individuals with acute myocarditis progress directly to the chronic phase with severe clinical manifestations, without the characteristically long period of IFCD.^38^Finally, similarly to myocarditis of other etiologies, access to low-cost diagnostic tools is currently being assessed to predict the risk of cardiovascular events and guide therapy. In addition to the limited access, the actual role of myocardial biopsy and CMRI to approach patients with suspicion of acute myocarditis of CD remains to be determined.^298^

### 5.2. The Indeterminate Form and Clinical Syndromes of Chronic Cardiomyopathy of Chagas Disease

#### 5.2.1 Natural History of the Chronic Phase of Chagas Disease

After the acute phase, untreated *T. cruzi* -infected individuals progress to the indeterminate form of the chronic phase, also called IFCD.^299 , 300^This form is classically defined by evident *T. cruzi* infection, confirmed by serology or parasitology test, in the absence of the disease’s symptoms and physical signs, in addition to lack of abnormalities on ECG at rest and on chest, esophagus, and colon radiologic study.^301^Patients with the IFCD have an excellent medium-term prognosis (5 to 10 years of follow-up), and deaths among them are very rare and probably not more frequent than those occurring in sex- and age-matched individuals not infected by *T. cruzi* .^299 , 300^

Although several individuals can remain indefinitely in the IFCD, in others, some decades after the acute infection, CD becomes clinically evident with disorders in specific organs, mainly heart, esophagus, and colon, characterizing the determined chronic clinical forms: cardiac, digestive, or mixed (cardiodigestive).

Epidemiological studies in endemic areas, observation in blood donors, and meta-analysis results, after systematic review, have shown that approximately 2% of the patients progress annually from IFCD to a clinical form of CD.^297 , 302^In Brazil, 20% to 30% of the patients are estimated to develop the cardiac form, 5% to 8%, esophageal disorders, and 4% to 6%, colonic disorders. With population aging, a greater part of infected individuals tends to develop the cardiac form, although recognition of the real prevalence is hindered by the coexistence of other cardiovascular diseases associated with aging.^303^The clinical manifestations of CD differ significantly in different Latin-American regions, and digestive syndromes are less often reported outside Brazil. From the epidemiological and clinical viewpoints, chronic cardiomyopathy is the most important form of CD because of its high morbidity and mortality and consequent medical and social impacts.

#### 5.2.2. Indeterminate Form of Chagas Disease: Importance of the Concept and Abnormalities on More Sophisticated Additional Tests

The term “indeterminate form” was used, for the first time, by Carlos Chagas in 1916 to designate *T. cruzi* infection in the “absence of any clinical syndrome” of CD.^229^Its possibility for progression to cardiac and/or digestive disease was originally described by Eurico Villela and Carlos Chagas in 1923^304^and highlighted in the 1950 decade by Laranja *et al* .^305^Those authors defined IFCD as the 10-to-30-year asymptomatic period extending from the end of the acute phase to the late initiation of cardiopathy from chronic infection.

Since then, several authors have used different terms to refer to that stage of CD, such as latent, asymptomatic, subclinical, laboratory, or “potentially cardiac” form, with no strict standardization of the diagnostic criteria, leading to different and even conflicting interpretations of the real meaning of the IFCD.

In 1984, a group of experts met in the city of Araxá, Minas Gerais state, to elaborate a consensus document reassuring the IFCD concept validity, as well as defining the objective diagnostic criteria previously cited.^301^The consensus emphasized that the presence of alterations identified on the most sophisticated investigative tests does not invalidate the previously presented concept, reinforcing the good medium-term prognosis of the cases, as confirmed by clinical follow-up as well as by ECG and ECHO.^211^

As there is still criticism about the IFCD concept, some suggestions for its modification have been made, such as replacement of ‘normal findings on chest X-ray’ by ‘normal findings on ECHO’ to define IFCD presence.^306^Elimination of the term has been even suggested, with its replacement by ‘chronic CD with no evident pathology’, when not only conventional ECG and chest radiography, but routinely performed doppler ECHO, Holter, and exercise testing resulted normal.^11^However, the classical concept of IFCD has been reassured in national and international guidelines.^2 , 7^

It is worth noting that, in clinical practice and epidemiological studies, patients with CD, normal findings on ECG and chest X-ray, and no digestive manifestations, do not routinely undergo gastrointestinal tract radiological assessment. This has led to the operational concept of “chronic CD with no apparent cardiopathy”, because the classical definition of IFCD requires the radiological assessment of the esophagus and colon.^299^

As the investigative methods became more sophisticated, several alterations, usually discrete and with no prognostic implication, could be detected in those individuals, as reported in studies with Doppler ECHO, radionuclide ventriculography, exercise test, cardiopulmonary exercise test, autonomic tests, and ambulatorial ECG.^204 , 299 , 307 , 308^In addition, invasive methods, such as endomyocardial biopsy, have shown histological changes in a substantial number of patients with IFCD, but at low intensity. Of 33 patients with IFCD undergoing endomyocardial biopsy, 60% have shown degenerative changes, fiber volume alterations, interstitial edema, inflammatory infiltrate, and fibrosis in small amount.^309^

Currently, CMRI provides the same data, with the advantage of being noninvasive.^310 , 311^In addition, more sensitive echocardiographic methods, such as tissue Doppler^312^and myocardial global longitudinal strain (GLS)^313^with speckle tracking echocardiography (STE), have shown alterations in patients with IFCD. However, such studies have not had sufficient follow-up to define if patients with those mild alterations would progress in a different way, and, eventually, to ventricular dysfunction.

#### 5.2.3. Progression to Chronic Cardiomyopathy

For the past 60 years, the risk of chronic cardiomyopathy development has been assessed in cohort studies, which have been gathered in a recent systematic review and meta-analysis.^297^The following cardiac primary outcomes were considered in that systematic review: (1) development of symptoms, in general, or of HF, specifically; (2) development of structural cardiomyopathy or cardiac arrhythmias, as observed in abnormal ECG or echocardiographic findings; and (3) presence of complications due to severe cardiomyopathy, such as sudden death, death associated with advanced HF, pulmonary embolism, or stroke. Twenty-three studies have reported observational longitudinal findings for patients with IFCD. Most of them were prospective cohorts conducted in Brazil or Argentina between 1960 and 2005. In studies with information on age group, the means ranged from 10 years to 44 years, the general mean being 31 years. The mean follow-up duration was 8.5 years, varying from 3 years to 18 years. That study concluded that the estimated annual combined rate of progression to CCCD was 1.9% (95% CI, 1.3% – 3.0%). The cumulative probabilities of cardiomyopathy evidence were approximately 17% and 31% in 10 years and 20 years, respectively.^297^

Although the rate of progression to cardiomyopathy is well defined, several doubts persist about the mechanisms involved in disease progression. In that same systematic review,^297^the authors found no difference regarding the rate of progression based on the investigation year (before or after 1985), study size (> or < 200 participants), participants’ mean age (< or > 32 years), or sex predominance. However, in studies originated from Brazil, the annual rate of progression to cardiomyopathy was significantly higher (2.3%; 95% CI, 1.2% – 4.3%) as compared to those from studies from other South-American countries (1.1%; 95% CI, 0.5% – 2.4%; p = 0.05), reinforcing the importance of regional differences in disease course.

In addition, the authors have reported that the subgroup receiving antiparasite treatment had an estimated annual combined rate of progression to cardiomyopathy significantly lower (1.0%; 95% CI, 0.5% – 1.9%) as compared to the subgroup not receiving etiological treatment (2.3%; 95% CI, 1.5 % - 3.5%; p = 0.03).^297^

Those results corroborate with the general pathophysiological notion that there is substantial evidence that parasite persistence (load) is a fundamental factor for IFCD progression to CCCD. A reference study^314^with a murine model of *T. cruzi* infection has shown that parasite persistence correlates with the presence of cardiac disease, while parasite elimination from the tissues is associated with inflammation improvement.

Subsequent studies have shown that inflammation and fibrosis extension, as well as disease severity, are associated with persistence of the parasite DNA in cardiac lesions observed in patients with CD.^315 , 316^The presence of parasitemia correlates significantly with known markers of disease progression, such as prolonged QRS, reduced LVEF, and higher levels of troponin and N-terminal pro-brain natriuretic peptide (NT-proBNP).^317^

In a cohort with 1813 patients with CCCD, those previously treated with benznidazole showed a significantly reduced parasitemia, lower prevalence of severe cardiomyopathy markers, and lower mortality after a 2-year follow-up.^318^Additional results of the NIH-REDS2 cohort, with a mean 8.7-year follow-up of the original cohort,^319^have shown that the incidence of cardiomyopathy in *T. cruzi* seropositive blood donors was 13.8 (95% CI, 9.5-19.6) events/1000 per year (32/262, 12%) as compared to 4.6 (95% CI, 2.3-8.3) events/1000 per year (11/277, 5%) in seronegative controls, with a difference in the absolute incidence of *T. cruzi* infection of 9.2 (95% CI, 3.6-15.0) events/1000 per year. Anti- *T. cruzi* antibody levels at the beginning of the study, an indirect measure of parasite load, were associated with the development of cardiomyopathy, with adjusted odds ratio of 1.4 (95% CI, 1.1-1.8) per unit of increase in antibody levels.^319^

The importance of parasite persistence for the development of CCCD is corroborated by an extensive nonrandomized clinical trial by Viotti *et al* .^320^showing that treatment with benznidazole, as compared to absence of etiological treatment, was associated with a reduction in the progression of CD and an increase in negative seroconversion. Other observational studies have reported similar results.^321 - 323^

There is yet evidence that, once the cardiopathy is established, tissue parasitism loses importance for the disease’s clinical course, and immune damages to the tissues predominate. According to this hypothesis, once the cardiopathy is established and the tissue parasite factor is eliminated, there would no longer be a chance of reversion to a less ominous natural history, because irreversible lesions would already be installed. Thus, the prospective, multicenter, randomized BENEFIT study, involving 2854 patients with CCCD receiving benznidazole or placebo for as long as 80 days and with a mean follow-up of 5.4 years, has shown that the use of the trypanocidal drug reduced parasitemia, but did not significantly influence clinical cardiac deterioration as compared to a control group.^324^Those results have been object of divergent and complementary discussions and interpretations,^325^and the importance of parasite persistence in patients with established cardiopathy remains controversial, as detailed in the chapter of etiological treatment in this guideline.

#### 5.2.4. Clinical Forms of Chronic Cardiomyopathy of Chagas Disease

The natural history of CCCD is characteristically slow and progressive, although occasionally more abrupt. Its clinical manifestations vary from asymptomatic (‘silent’ cardiopathy) to severe findings, with refractory HF, rhythm disorders, and thromboembolic phenomena, the three major clinical syndromes.^326^The most important symptoms are: dyspnea on exertion, fatigue, palpitations, dizziness, syncope, chest pain (angina, usually atypical), and lower limb edema.

The physical examination reveals one or more abnormalities: systolic murmur of mitral and/or tricuspid regurgitation; splitting of the second heart sound, usually associated with right bundle branch block (RBBB); diffuse and displaced apical thrust; and arrhythmias, extrasystoles being the most common ones.

5.2.4.1 Abnormalities on Complementary Tests

The ECG in CD has fundamental diagnostic and prognostic values.^327 , 328^The absence of electrocardiographic changes, however, is no absolutely reliable indicator of the cardiac impairment absence.^299^The RBBB, isolated or in association with other changes, is the most common electrocardiographic abnormality.^329 , 330^It is more typically associated with left anterior fascicular block (LAFB) and ventricular extrasystoles (VE). The QRS duration is directly related to the LV size and inversely related to LVEF.^331^A QRS duration > 120ms and QT interval > 440ms have moderate accuracy to predict reduced LVEF in patients with CD.^332^

The ECG abnormalities most frequently associated with LVEF reduction in CD are frequent supraventricular and ventricular extrasystoles, AF, intraventricular blocks, pathologic Q waves, and ST-T alterations.^332 , 333^The combination of intraventricular conduction disorders with extrasystoles or sinus bradycardia associates with both LVEF reduction and LV volume increase.^334^It is worth noting that the electrocardiographic changes caused by CD tend, in older individuals, to add to those caused by the biological aging process itself.^303^A more detailed presentation of the ECG changes of CCCD and of those not sufficient to make that diagnosis is found in other chapters of this guideline.

Chest radiography is an important test to diagnose patients with CCCD, enabling the assessment not only of the enlargement of cardiac chambers, but, especially, of the pulmonary congestion grade, an imperceptible change on usual ECHO.^335 , 336^There is low correlation between the cardiac silhouette enlargement on chest radiography and the systolic ventricular dysfunction degree.^335^However, cardiomegaly detected based on cardiothoracic index (CTI) > 0.5 on radiography correlates better with LV diastolic diameter (LVDD) increase, suggesting the presence of LV systolic dysfunction.^337^

In CCCD, the radiological findings are similar to those detected in other DCMs. However, an interesting particularity refers to a fact described by clinicians decades ago: several patients with evident systemic congestion, including ascites, hepatomegaly, and anasarca, show a clear disproportion between the advanced cardiomegaly and pulmonary congestion that is usually mild or even absent.^336^

For decades, transthoracic ECHO has been an important tool for the diagnosis and follow-up of patients with the different forms of CD.^338 , 339^It is the most often used noninvasive test to assess cardiac function because of its wide availability and high reliability regarding performance and interpretation, as well as its relatively low cost. It enables determining the evolutionary status of the disease, as well as the most subtle cardiac impairment alterations, especially in the less advanced phases of cardiomyopathy. In CCCD, up to 13% of the patients in stage B (see HF classification) have a characteristic regional deficit, despite preserved global biventricular systolic function.^340^It is worth noting that such isolated regional LV motion alterations evidence poor prognosis, as observed in serial studies with ECHO.^341 , 342^

Of several parameters analyzed, the most important ones are: LVEF, left atrial diameter, left atrial volume, systolic and diastolic LV diameters, diastolic function, right ventricular (RV) systolic function, global and regional LV contractility, global RV contractility, and presence of apical aneurysm.

The RV echocardiographic analysis is hindered by technical difficulties regarding the RV chamber itself and the essence of the ultrasonographic method. Thus, RV dysfunction is more often evidenced when there is concomitant and significant LV involvement.^343 , 344^Despite this pathophysiological notion, evidence derived from studies using other methods, such as radionuclide ventriculography, CMRI, and more specialized ECHO, indicates that some patients with CCCD have early important isolated RV morphofunctional changes.^203 , 345 - 348^In such conditions, in the absence of concomitant LV pathological involvement and while the impedance of the pulmonary circuit remains reduced, the RV dysfunction might remain unnoticed because the LV *vis-a-tergo* is sufficient to maintain the flow and pulmonary vascular resistance normal, as reported in a publication on the subject.^349^

Finally, when present in the natural history of the disease, the clinically manifest RV systolic dysfunction significantly worsens the prognosis of patients with CCCD.^350^

5.2.4.2. Cardiac Arrhythmias

Cardiac arrhythmia is an extremely common manifestation of CCCD, and ventricular ectopic activity predominates since the early phases of its natural history. Ventricular extrasystoles have been shown in 15% to 55% of individuals with positive serology for *T. cruzi* . When patients with ECG changes at rest and manifest HF undergo dynamic electrocardiography assessment, almost all of them (99%) have VEs, which, in 87% of the patients, are multiform or present as repetitive (paired) forms or even as nonsustained ventricular tachycardia (NSVT), that is, three or more successive ventricular ectopic beats, lasting less than 30 seconds.^351^

In addition, involvement of the sinus node and the atrioventricular conduction system is very frequent in patients with CCCD. Sinus node dysfunction can manifest as bradycardia or even sinus arrest, second-degree sinoatrial block, junctional rhythm, and accelerated idioventricular rhythm. First-degree atrioventricular block (AVB) is one of the most frequent atrioventricular conduction disorders, and can be transient or permanent. Second-degree AVB is less frequent and classified into the following types: Mobitz I (Wenckebach), Mobitz II or advanced-degree. Third-degree or total AVB (TAVB) can occur in 10% of the patients, being more frequent than in any other acquired cardiopathy. Atrial fibrillation tends to manifest later, usually associated with more advanced degrees of systolic dysfunction and ventricular dilation.

The arrhythmias can be asymptomatic or cause palpitation, dizziness, dyspnea, weakness, presyncope, syncope, or cardiac arrest. Sudden death accounts for 50% to 65% of the deaths due to CD.^352^Sudden death is usually precipitated by physical exercises and can be associated with SVT or ventricular fibrillation (VF) and, less frequently, asystole or TAVB. Approximately 40% to 50% of the cases of sudden death are asymptomatic prior to the fatal episode, but most patients have concomitant severe impairment of the ventricular systolic function and of the conduction system. The severity of ventricular arrhythmias tends to correlate with the ventricular dysfunction degree. However, differently from other diseases, patients with CCCD and malignant ventricular arrhythmias may present relatively preserved LV global function, but with regional dyskinesias indicating localized fibrosis.^353^Episodes of malignant ventricular arrhythmias are much more frequent in patients with CCCD than in those with other forms of cardiopathy, such as the one resulting from coronary artery disease or DCM of other etiologies.^354 - 356^

5.2.4.3. Heart Failure Syndrome

Heart failure manifests in many patients throughout the natural history of CCCD, usually with biventricular dysfunction, including early symptoms, such as dyspnea, fatigue, lower limb edema, and atypical chest pain. Diastolic dysfunction can be observed in the early stages of CCCD, in the absence of regional or global LV systolic dysfunction, and can be explained by a certain degree of LV diffuse fibrosis.^357^

As already mentioned, in some patients, right HF can be more prominent than left HF, but RV dysfunction, when clinically manifest, is usually associated with LV dysfunction in an advanced stage of CCCD.^343 , 349^

The classification of HF of CD etiology, considering the presence or absence of functional and/or structural defects in general and LV systolic function especially, is useful when applied to CCCD, after mild modifications from the 2011 SBC guidelines, enabling the identification of different subgroups or evolutionary stages from the prognostic and therapeutic viewpoint.^1^The HF classification according to LVEF is shown in Table 5.1 . The classification into progressive stages is shown in Table 5.2 .

**Table 5.1:** – Chronic cardiomyopathy of Chagas disease: normal systolic function and heart failure classification according to left ventricular ejection fraction (LVEF).

CATEGORY	CRITERION
Normal global systolic ventricular function	• LVEF ≥ 55% - without segmental dysfunction - with segmental dysfunction
Heart failure with mildly reduced ejection fraction (HFmrEF)	• LVEF between 41% and 54%
Heart failure with reduced ejection fraction (HFrEF)	• LVEF ≤ 40%
Heart failure with improved ejection fraction (HFimpEF)	• Baseline LVEF ≤ 40%, a ≥ 10-point increase from baseline LVEF, and a second measurement of LVEF > 40%

**Table 5.2:** – Classification of chronic Chagas disease into progressive stages.

	Indeterminate form	Chronic cardiomyopathy of Chagas disease			
	Stage A	Stage B1	Stage B2	Stage C	Stage D
**Characteristics**	Asymptomatic; with no structural cardiac or digestive disease (ECG and radiological study); risk of developing CCCD (30%)	Structural cardiac disease; normal global systolic ventricular function; no symptom of HF	Structural cardiac disease; global systolic ventricular dysfunction; no symptom of HF	Structural cardiac disease; global systolic ventricular dysfunction; previous or current symptoms of HF	Structural cardiac disease; global systolic ventricular dysfunctionl; symptoms of HF at rest, refractory to optimized clinical treatment
**ECG**	Normal	Altered	Altered	Altered	Altered
**Segmental ventricular dysfunction**	Usually absent	May be present	May be present	May be present	May be present
**LVEF (ECHO – Simpson)**	≥ 55%	≥ 55%	< 55% (usually between 41% and 54%)	< 55% (usually ≤ 40%)	Usually ≤ 25%
**Functional class (NYHA)**	Not applicable	I	I	I, II, III or IV	IV
**Cardiomegaly (chest X-ray)**	Absent	Absent	May be present	Usually present	Present
Complex ventricular arrhythmia (24-h Holter)*	Usually absent	May be present	Usually present	Present	Present
**Myocardial fibrosis (late enhancement on CMRI)**	May be present	Usually present	Usually present	Present	Present

*couplets/runs of ventricular extrasystoles. CCCD: chronic cardiomyopathy of Chagas disease; ECG: electrocardiogram; LVEF: left ventricular ejection fraction; HF: heart failure; NYHA: New York Heart Association, CMRI: cardiac magnetic resonance imaging.

5.2.4.4. Systemic and Pulmonary Thromboembolic Syndrome

This syndrome is very common in CCCD, venous and arterial thromboembolic phenomena being the third cause of death.^352 , 358^From the clinical viewpoint, thromboembolic phenomena to the brain predominate, followed by embolism to other systemic organs and limbs, and lastly by pulmonary embolism diagnosed during life. Stroke can be the first devastating manifestation of the disease.

The high frequency of the thromboembolic syndrome in CCCD can be due to several factors that can predominate depending on the disease’s phase. Thus, the apical aneurysm can be an early alteration in CCCD, but, more commonly, thrombosis in systemic veins that can cause pulmonary embolism are complications of HF, when cardiac output and venous return are hindered. In the presence of HF, dilation of the chambers favors atrial and ventricular mural thrombosis, causing systemic and/or pulmonary embolism. Atrial fibrillation is more frequent in advanced cases of CCCD, increasing the risk for thromboembolic complications.

Chagas disease is a major cause of stroke in Latin America, accounting for up to 20% of this complication in endemic areas.^358 - 360^The incidence of stroke in patients with known CD ranges from 0.56 to 2.67 per 100 individuals-year.^361^Thus, CCCD should be regularly included in the differential diagnosis of stroke in Latin America.^362^Ventricular systolic dysfunction, enlarged left atrial volume, apical aneurysm, cavitary mural thrombosis, and arrhythmias, such as AF, seem to be important risk factors for stroke of CD etiology, characteristically of cardioembolic nature.^363^In 50-70% of the patients, stroke manifests as a partial anterior circulation syndrome, which includes two of the three signs: motor or sensory deficit involving face, arm and leg; homonymous hemianopsia; and upper cerebral dysfunction, expressed as aphasia or visuospatial deficit. Less often, patients will have lacunar or posterior circulation syndrome.

A risk score for stroke ( *IPEC-FIOCRUZ)* has been developed in a prospective observational study with 1043 patients.^364^As discussed in a specific chapter of this guideline on thromboembolic complications of CD, that score needs to be reviewed to contemplate updated scientific considerations.

## 6. Diagnosis of Cardiomyopathy of Chagas Disease

### 6.1. Methods to Determine the *T. cruzi* Infection

#### 6.1.1. Introduction

The diagnosis of an infectious disease should be based on clinical, epidemiological, and laboratory data, which should be considered to confirm or rule out the diagnosis.^365^

Some clinical data can be very suggestive of CCCD, such as RBBB on ECG.^366^However, there is neither a CCCD-specific electrocardiographic abnormality nor one occurring in all patients with the disease. Epidemiological data, particularly patient’s origin from known endemic areas, aid in making the diagnosis. In addition, the family history should be taken, because two thirds of the patients from endemic areas have family members infected, particularly mother or siblings, or even report sudden death in their families.^367^

Laboratory tests can detect the parasite or, more commonly, the anti- *T. cruzi* antibodies. In the chronic phase of CD, most patients have low parasitemia and parasites cannot be found on the blood smear. Thus, the CD etiology cannot be ruled out based on the absence of the parasite. On the contrary, almost all chronically infected individuals have anti- *T. cruzi* antibodies at varied levels. Therefore, a clinician willing to confirm or rule out the *T. cruzi* etiology in a patient with heart disease should initially request serological tests.

#### 6.1.2. Serological Tests Available and Which to Request

The serological tests available can be classified as conventional and non-conventional. Each laboratory uses different conventional tests, such as indirect immunofluorescence (IIF), indirect hemagglutination (IHA), enzyme-linked immunosorbent assay (ELISA), and, in recent years, non-conventional tests, such as chemiluminescent microparticle immunoassay (CMIA) and electrochemiluminescence immunoassay (ECLIA) in automated platform, in addition to rapid tests. Non-purified and purified products (recombinant, synthetic, and others) can be used as antigens in all those tests.^365^For the diagnosis of CD, the WHO recommends two tests of different principles, either conventional or non-conventional.^2 , 38 , 60 , 368^

#### 6.1.3. Interpretation of the Results

The combination of the results of the two tests enables the classification of the patient’s serum as positive (two reagent tests) or negative (two non-reagent tests), considering concordant results of the two tests performed.^369^Two positive tests indicate that the patient is seropositive, with detection of anti- *T. cruzi* antibodies by use of two different methodologies, meaning that the patient is infected with *T. cruzi* . When the result is non-reagent (two non-reagent tests of different principles), the serology is negative; in such cases, usually there is no epidemiology, and clinical manifestations, if present, can be explained by causes other than *T. cruzi* infection. In a third unusual possibility (< 5% of the cases), the results are non-concordant, with a reagent test and another non-reagent ( Figure 6.1 ).

**Figura 6.1:** Flowchart for the serological diagnosis of Chagas disease. R: reagent; NR: non-reagent; U: undetermined. Note: Test 1: IIF or IHA or ELISA or chemiluminescence or electrochemiluminescence; Test 2: test different from test 1; Test 3: test with a different principle from test 1 and test 2.

Finally, in another possibility, one of the test’s results is undetermined, that is, in the narrow range between negative and positive, the ‘grey’ region. An example is the passive transfer of antibodies from an infected mother to her child. The progressive decrease in the level of maternal antibodies in the non-infected offspring, around the third month of age, can correspond to that grey region, an undetermined result.^369^

In those rare situations of discordance, the clinician, after analyzing the epidemiological and clinical data, should assess if the patient underwent previous specific treatment and if there is any history of cutaneous leishmaniasis or other diseases, especially autoimmune ones. In such cases, a new blood sample should be collected. Usually, the discordant result becomes concordant in the new sample. If the result remains undetermined, the patient should be referred to a specialized service/laboratory, where other techniques will be performed for a conclusion to be reached. Exceptionally the referral laboratory will not reach a conclusion regarding the individual’s infectious status, and parasitological methods can be used. In such cases, a clinical assessment with ECG should be performed. However, even in the presence of a normal EGC, the patient with an inconclusive serology should be instructed not to donate blood.

#### 6.1.4. Special Situations

6.1.4.1. Inconclusive Serological Results

As already mentioned, inconclusive serological results are not usually found (< 5%), being often associated with the presence of other diseases, especially visceral or cutaneous leishmaniasis, systemic lupus erythematosus, and chronic liver diseases, usually with increased gamma globulin levels. These are the so-called cross reactions. Thus, other causes should be investigated, and the patient asked about any previous treatment with benznidazole. If affirmative, the individual’s antibody levels might have decreased as a consequence of the treatment, turning the serology result undetermined.

6.1.4.2. Test Result Not Corresponding to Clinical Expectation

As already mentioned, two serological tests of different principles should be requested, preferably including the titles obtained, indicating the levels of antibodies. Usually both results are either positive or negative. Rarely the results of the two tests are discordant as follows: one negative and the other positive, or one positive and the other undetermined. In these situations, a new blood sample should be collected, using the same techniques and, if possible, a third one [for example, if ELISA is reagent and IIF is non-reagent, IHA or chemiluminescence immunoassay (CLIA) or ELISA with other antigens should be requested]. Usually, this procedure enables a conclusive result.

Several interferences can lead to a false reagent result, not confirmed by two other negative tests. In other cases, one test can be non-reagent while the same serum is reagent by the other two tests. Clinical and epidemiological data usually lead to a diagnosis. In other circumstances, the clinical and epidemiological data may point to *T. cruzi* infection, while the serological tests are negative, requiring a new blood sample collection in a referral or specialized laboratory for new tests. According to such laboratories, when the results of three tests of different principles are negative, usually it is not *T. cruzi* infection. Thus, there are cases of RBBB due to other causes, and families with some members not infected by *T. cruzi* are often found, leading to the hypothesis of natural resistance to CD, already known in other infections, such as hanseniasis and tuberculosis. In addition, spontaneous cure, although rare, can occur. Exceptional cases of *T. cruzi* infection without the detection of serum antibodies have been reported.^370^In such cases and if suspected, parasitological tests should be performed to solve the doubt.

6.1.4.3. Parasitemia

Although most chronic patients have low parasite load in peripheral blood, approximately 20% of them can have high parasitemia detected by serial multiplication tests (blood culture, PCR). In cases of RCD due to immunosuppression (HIV and others), most patients have high parasitemia. It is worth noting that “reactivation” means that the individual, from the laboratory viewpoint, is in the acute phase, defined by the detection of parasites in the peripheral blood on direct examination, which is only observed in a short period of the early acute phase and during reactivation itself in the chronic phase. Of note, the laboratory definition of the acute phase consists in the presence of viable parasites in peripheral blood.

6.1.4.4. Negative Serology in Patients in the Chronic Phase

Although possible, it is exceptional and has been reported in Bolivia.^370^

6.1.4.5. Spontaneous Cure

Spontaneous cure of CD has been reported by Zeledón *et al* .^371^after collecting blood from infected patients in Central America years after their acute phase, which had been duly registered with positive direct parasitological tests when no specific treatment was available. The rarity of spontaneous cure has been confirmed by a later study with 110 individuals in the chronic phase of CD, followed up for over 10 years, reporting that none of them showed further levels lower than the initial ones.^372^Nevertheless, the occurrence of spontaneous cure has been shown to be < 1%. Usually, in the second sample collection, some antibodies are always detected, meaning that their levels do not become totally negative. If they do, other hypotheses should be assessed, of which the most likely is difference between tests of distinct origins.

6.1.4.6. Acute Phase Diagnosis

Exceptional in Brazil nowadays, it is practically limited to cases of oral transmission through the ingestion of food contaminated with infected triatomines or their feces, particularly in the Amazonian region (approximately 350 cases per year). Oral transmission currently represents the major cause of acute disease in several South American countries.^114^

In general, acute CD can be caused by triatomines (vector and oral transmission), contaminated blood transfusion or solid organ transplantation, vertical or congenital transmission, and laboratory accident. In a naturally or iatrogenically immunosuppressed individual, RCD is considered as acute phase. In such cases, the laboratory diagnosis is made through the direct search of the parasite by use of parasitological methods including PCR.^373^

6.1.4.7. Blood Services

The objective of blood services is to provide good-quality blood and, thus, they should use high-sensitivity tests, capable of detecting > 99% of infected samples. However, that reasoning does not apply to the CD diagnosis. As a result of the necessary care to obtain blood with no infectious agent, the specificity can be lower (98%), leading to blood exclusion, but not necessarily meaning that a particular donor is infected. Frequently, when donating blood, an individual can be notified that he/she might be infected. In such circumstance, two serological tests of different principles should be requested, as already mentioned. Although, in the case series of referral services, 70% to 80% of the donors excluded are effectively infected, a significant proportion (20% to 30%) of those individuals will not be confirmed to have CD, reinforcing the need for a new blood collection and two serological tests.^374^

6.1.4.8. Congenital Transmission

Vertical (maternal-fetal) transmission represents the major *T. cruzi* transmission pathway in vector-free regions and in several endemic areas.^59^The prevalence rate of that type of transmission in Brazil is 1.7%, one of the lowest indices as compared to those of other South American countries.^57 , 87^It is important to consider that babies with clinical signs suggestive of acute CD and born from women with chronic *T. cruzi* infection should undergo the diagnostic tests for infection as soon as possible. The early diagnosis of congenital CD is extremely important, because the trypanocidal treatment of infected babies in their first year of life leads to cure in 100% of the cases.

However, when the mother is infected with *T. cruzi* and no fetal infection occurs, the possibility of passing antibodies (IgG) from mother to fetus through the placenta during pregnancy should be considered. Thus, to detect congenital transmission, the parasitological diagnosis is recommended in the umbilical cord blood or in the newborn in the first 72 hours. Alternatively, in the absence of signs and symptoms of infection, the diagnosis can be made during the first months of life by use of direct parasitological methods (blood smear, microhematocrit, leukocyte cream, and PCR), with assessment of two or three samples to increase sensitivity.^2^Babies with negative initial parasitological tests should undergo serologic tests between 9 and 12 months of age, when maternal antibodies have disappeared. The persistence of unchanged anti- *T. cruzi* antibody titles in children from the age of 9 months on indicates congenital infection, while absence of those antibodies at that time rules out the possibility of infection in the child.^2^

6.1.4.9. Serology of Infected Individuals Treated with Chemotherapy Drugs

The follow-up of patients by use of laboratory tests after specific treatment of the infection is approached with more details in the chapter regarding chemotherapy for the disease in general. Here, for those looking for support to exclude or confirm the diagnosis, it is worth noting that it is a very sensitive and complex subject to be reduced to a few principles. According to J. R. Cançado, “it is obvious that, if an infected individual has antibodies and parasites, both need to disappear so that the individual can be considered cured (after chemotherapy)”. This precept applies to treated individuals in the acute phase (70% of cure) in a period of months, as well as to children receiving the trypanocidal treatment in the chronic but recent phase, confirming a negative serology (ELISA with recombinant antigens) in 58% to 62% of the cases after 3 to 4 years of follow-up.^375 , 376^In addition and to a smaller proportion (25%), patients treated in the late chronic phase showed a negative serology only decades after undergoing treatment. This relates to the duration of the parasite-patient coexistence (weeks, years, decades).^377 - 381^The analysis of the cure should be based on negative serological tests or even on the reduction (as long as significant) of antibody levels, preferably with diagnostic tests using non-purified antigens.

6.1.4.10. Rapid Serological Tests

Rapid diagnostic tests are typically easy to handle and do not require a reference laboratory for specialized diagnosis as compared to the classical serological techniques. Several types of rapid tests are available for the diagnosis of CD. Many can be performed on serum or peripheral blood and stored at room temperature for long periods of time. Their use is indicated in endemic areas, mainly in field research (serological and epidemiological inquiries), because they contribute to increase access to diagnosis. However, despite being used for that purpose, the rapid tests for CD are not usually recommended as an independent diagnostic method by the WHO, because of their low sensitivity.^382^

6.1.4.11. Parasitological Tests

Parasitological tests should be requested in special situations, not routinely. There are several types of parasitological tests for the chronic phase of CD, which, because of low parasitemia, are aimed at promoting the multiplication of the few parasites present by use of blood culture, xenodiagnosis, inoculation in experimental animals, or identification of nucleic acids (DNA or RNA) specific to the protozoan parasite, using the PCR technique.

The multiplication of the parasites can take several weeks; thus, the result can take time. It involves “in house” techniques, which require special conditions (reagents, insectary, *bioterium* ), as well as highly-qualified individuals. Usually, those techniques are performed only at specialized research centers. Blood culture and xenodiagnosis used in the chronic phase have low and variable sensitivities (approximately 20%); when repeated, the probability of detection can be increased, reaching up to 60% of sensitivity.^365 , 368 , 369 , 383^For some patients with very low parasitemia, even successive tests will yield persistently negative results.

In the PCR method, the identification of part of the parasite’s genetic material requires less time (hours), but special reagents and technical conditions are also needed. Because of its importance, the PCR technique is detailed below.

6.1.4.11.1. Indications for Parasitological Tests, particularly Polymerase Chain Reaction

One of the major indications for parasitological tests is the follow-up of patients treated with benznidazole or other chemotherapy drugs. Accurate diagnostic methods and reliable markers of the response to the parasiticide treatment are priorities in the research and resource development in general for application in CD.^384^PCR has been valued for patients’ assessment and monitoring when a positive PCR result for the parasite genetic material by the end of the trypanocidal treatment indicates therapeutic failure.^385^However, after treatment, a negative PCR result does not indicate cure of the infection. It is worth noting that the negative serological conversion in treated chronic patients who respond favorably to treatment may take years.^386^The PCR can indicate in advance therapeutic failure, evidencing resistance to the trypanocidal treatment and therapeutic ineffectiveness.

In addition, in cases of RCD, PCR is useful, enabling the early detection of reactivation. Monitoring RCD in immunosuppressed individuals is an area of increasing interest. The RCD in infected patients in the chronic phase who acquire HIV or during immunosuppressive therapies, after organ transplantation, autoimmune diseases or cancer, usually increases parasitemia, characterizing acute CD.^387 - 389^In cases of immunosuppression resulting from CTX, the exclusion of rejection and the detection of RCD can be done early by use of PCR performed in peripheral blood samples and endomyocardial biopsy.^390 - 392^

6.1.4.11.2. Interpretation of the Results of Parasitological Tests

By definition, parasitological tests can only be valued if positive, showing the presence of parasites or their amplified structures (PCR). A negative test, per se, has no value, because the result is only valid for the sample collected. Another sample collected on another day can be positive. Thus, a negative parasitological test does not mean that the individual is not infected by *T. cruzi* nor that the infection has been cured.

6.1.4.12. Polymerase Chain Reaction

Since the 1990s, PCR has been used as a supportive molecular method for the diagnosis of patients in the chronic phase of CD, because of its higher sensitivity as compared to that of parasite multiplication tests (blood culture and xenodiagnosis) and its high potential for use in trypanocidal chemotherapy monitoring.^393 - 396^

Several studies have shown positive PCR results in 40% to 70% of chronic patients previously diagnosed by use of conventional serology. This variability in positivity depends on several factors, such as parasite load, volume of the blood collected, blood sample for DNA isolation, DNA purification method, target region to be amplified, characteristics of the populations studied, as well as the high genetic diversity observed in the parasite DTU.^397 - 401^

Different combinations of molecular targets, sets of reaction initiators, extraction methods, and DNA amplification platforms have been used to assess the accuracy of the method in peripheral blood samples of patients with chronic CD. Usually, the diagnostic sensitivity is lower as compared to that of serological tests.^401^For those patients, molecular-based detection methods have a limited diagnostic value, because their sensitivity is significantly lower than that of serological tests.^400 , 402^

It is worth noting that a positive PCR result confirms the parasite’s presence in a certain sample; however, because of parasites’ scarcity and intermittent circulation, which characterize the chronic phase, a negative PCR result does not exclude infection.^398^Nevertheless, in case of positive samples, the PCR enables the characterization of the *T. cruzi* infectant DTU directly in the patient’s blood, not requiring the parasite’s previous isolation.

For selecting the molecular target to detect the *T. cruzi* genetic material, the following is recommended: the use of conserved DNA sequences (present in all genetic lineages of the parasite), which are exclusive of *T. cruzi* (specificity); and these sequences should be represented in multiple copies in the genome (higher sensitivity). The most often used targets in conventional PCR (qualitative) have been the DNA from the kinetoplasts or kDNA (mitochondrial genome) and the repeating units (satellite DNA) present in the nuclear genome.^402^

The real time or quantitative PCR (qPCR) can determine the parasite load by quantifying specific DNA sequences. For the quantification assays, satellite DNA sequences are preferably used, because of their smaller variability in the number of copies between the different genetic lineages of *T. cruzi* as compared to the kDNA minicircles.^403^

6.1.4.13. Operational Procedures for PCR Use

1. Blood collection: 10mL of peripheral blood (minimum of 5mL) are usually collected in tubes with EDTA (any other anticoagulant inhibits the enzyme of the reaction). The blood is immediately transferred to a tube containing the same volume (1:1) of a lysis buffer for sample preservation, the 6M guanidine hydrochloride - 0.2M EDTA solution (pH 8.0).

2. Sample processing: the blood in the guanidine solution is boiled in water bath (100ºC, 15 min) to promote a homogeneous distribution of the parasite’s target DNA sequences, enabling DNA extraction from a lower sample volume (300 µL). The boiled material remains at room temperature for 48 to 72 hours and can undergo DNA extraction. The remaining material is stored in the refrigerator or cold chamber, without freezing.

3. Two 300-µL copies undergo DNA extraction using commercial kits based on purification through silica minicolumns, according to the manufacturer’s recommendations.

4. The PCR protocols follow those standardized in-house by the laboratories, usually based on the international consensus description.^402^

5. For qualitative PCR, the test result is the visualization of the amplified product (of the kDNA or satellite DNA) from the agarose gel electrophoresis stained with fluorescent agents that intersperse with DNA.

6. For qPCR, the protocols also follow the international consensus^403^and require the inclusion, in each assay, of standard samples with preestablished concentrations of parasites (parasite equivalent per reaction), which serve as calibrating samples for the absolute *T. cruzi* quantification. The results generated in qPCR are visualized in real time as graphs issued by the equipment, without requiring running an electrophoresis.

7. The use of positive (DNA extracted from cultivation of *T. cruzi* cells) and negative (DNA extracted from blood known to be non-infected and a tube containing ultrapure water without DNA) controls is highly recommended.

8. In cases with negative PCR in qualitative assays, DNA extraction should be repeated in two other samples of blood in guanidine (300 µL) for the new PCR test directed to some human gene (β-globin, β-actin, etc). This is a decisive step to exclude false-negative results due to the presence of inhibitors in blood samples or the loss or poor quality of the DNA extracted.

9. Recently, a diagnosis kit for PCR produced by the FIOCRUZ (Bio-Manguinhos) has been made available and approved by the sanitary authorities, which will facilitate its use in the Public Health Central Laboratories (LACEN).

## 6.2. Diagnostic Methods for Structural and Functional Cardiac Abnormalities

Table 6.1 shows the additional tests used in the diagnosis of cardiomyopathy in individuals with suspected or confirmed CD. In addition, it shows the strength of each recommendation and its level of evidence. For some tests, in addition to their diagnostic extent, their prognostic connotation is added.

**Table 6.1:** – Additional methods for the diagnosis and prognosis of chronic cardiomyopathy of Chagas disease (CCCD)

Tests	Recommendation grade	Level of evidence
**12-lead Electrocardiogram**		
Initial diagnostic and prognostic assessment of every individual with positive serology for Chagas disease	Strong	B
Annual (ECG with specific changes*) or biannual (normal ECG or with unspecific changes†) repetition for assessment of progression and prognosis	Strong	C
Repetition at any time in the presence of changes in clinical findings	Strong	C
**Chest X-ray**		
Initial diagnostic and prognostic assessment of every individual with CCCD	Strong	B
Clinical evidence of pulmonary or systemic congestion	Strong	B
**Exercise or cardiopulmonary test**		
Initial diagnostic and prognostic assessment of every individual with CCCD	Conditional	C
Presence of symptoms, such as chest pain, palpitations, syncope, or presyncope related to physical exertion, or dubious, or of unknown origin	Strong	B
Periodical assessment of implantable devices for programming optimization and assessment of physical capacity	Conditional	C
Cardiopulmonary test for functional assessment, risk stratification, and support for cardiac transplantation indication in advanced HF	Strong	B
**24-hour Holter**		
Initial diagnostic and prognostic assessment of every individual with CCCD	Strong	B
Investigation of symptoms, such as palpitation, presyncope, and syncope	Strong	B
Follow-up of patients with complex ventricular arrhythmias and assessment of antiarrhythmic drug therapy	Conditional	C
Follow-up of patients with sinus node dysfunction or potentially risky AV/IV conduction disorders	Conditional	C
Follow-up of individuals with implantable cardiac devices (PM, ICD, CRT)	Strong	C
**Intracardiac electrophysiological study**		
Assessment of syncope (or unquestionable presyncope) and suspicion of brady- or tachyarrhythmia, when noninvasive tests are inconclusive	Strong	B
Differential diagnosis of wide QRS complex tachycardia and uncertain diagnosis	Conditional	C
**Conventional echocardiography**		
Initial diagnostic and prognostic assessment of every individual with CCCD	Strong	B
Suspicion of CCCD based on history, clinical examination, or electrocardiographic changes	Strong	B
When HF symptoms aggravate or in the presence of syncope, arrhythmic events, stroke, or peripheral thromboembolism	Strong	B
Periodical reassessment** regardless of the presence of global or regional systolic dysfunction on previous test	Conditional	C
Assessment of individuals with normal ECG and suggestive symptoms of CCCD	Conditional	C
**Nuclear medicine (radionuclide ventriculography)**		
Assessment of ventricular function, especially the right ventricle, in addition to echocardiography, as an alternative to magnetic resonance imaging	Conditional	C
Identification of regional contractility defects, when echocardiography is technically inappropriate, as an alternative to magnetic resonance imaging	Conditional	C
**Nuclear medicine (myocardial perfusion)**		
Initial assessment of individuals with CCCD and chest pain	Conditional	C
Additional assessment and detection of microvascular defects in individuals with chest pain and angiographically normal coronary arteries	Conditional	B
**Nuclear medicine (sympathetic innervation assessment)**		
Additional method for the assessment of complex ventricular arrhythmias	Conditional	C
**Cardiac magnetic resonance imaging**		
In the suspicion of concomitance of CCCD and coronary artery disease or other non-ischemic cardiomyopathy and to assess the etiology and extesion of myocardial fibrosis	Conditional	B
Morphological and functional, global and segmental assessment and search for thrombi, as an alternative to echocardiography with technical limitations	Conditional	B
When there is no initial clinical suspicion of Chagas disease (nonendemic area), CMRI can favor an extremely probable diagnosis of CCCD based on the late enhancement pattern and motivate the performance of serological tests to confirm the etiological diagnosis	Conditional	C
Discordance between symptoms and degree of ventricular dysfunction in the context of ambiguous indication of procedures, such as ICD implantation	Conditional	C
Planning of electrophysiological studies with possible RF ablation (to guide the local of ablation) or device implantation to define if resynchronization therapy should or not be added (extensive lateral and/or septal fibrosis is considered a relative contraindication because it increases the rate of nonresponders)	Conditional	C
**Computed tomography**		
Assessment of coronary anatomy in patients with CCCD and high probability of obstructive coronary artery disease	Conditional	B
Characterization of normal myocardium/fibrosis on late enhancement as an alternative to CMRI	Conditional	C
Assessment of biventricular systolic function in addition to echocardiography in patients with contraindication to CMRI	Conditional	C
**Cardiac catheterization**		
Assessment of coronary anatomy in patients with CCCD and high probability of obstructive coronary artery disease	Strong	B
Assessment of pulmonary vascular resistance in candidates for cardiac transplantation with noninvasive evidence of pulmonary hypertension	Conditional	B

*Mainly when multiple: complete right bundle branch block, especially associated with left anterior fascicular block (LAFB), frequent ventricular extrasystoles, pathological Q waves or electrical inactive areas, second-degree (Mobitz 2) or third-degree atrioventricular block (AVB), and atrial fibrillation; †Mainly when isolated: isolated LAFB, sinus bradycardia, first-degree AVB, low-voltage QRS complex on peripheral leads, and nonspecific abnormalities of the ST-T segment. HF: heart failure; AV: atrioventricular; IV: intraventricular; PM: pacemaker; ICD: implantable cardioverter-defibrillator; CRT: cardiac resynchronization therapy. **3 to 5 years for cases with preserved ejection fraction and no segmental contractility change on previous echocardiogram; 1 to 2 years for cases with global (even mild) or segmental left ventricular dysfunction. HF: heart failure CCCD: chronic cardiomyopathy of Chagas disease; ICD: implantable cardioverter-defibrillator; CMRI: cardiac magnetic resonance imaging; RF: radiofrequency.

### 6.2.1. Electrocardiogram in Chagas Disease

The ECG is the most important initial cardiovascular test to assess patients with CD, enabling the classification of its clinical forms.^330 , 404^Thus, well-defined electrocardiographic changes in the infected individual indicate the presence of cardiomyopathy.^334^The most frequent and defined alterations are atrioventricular conduction blocks, right bundle branch and left anterosuperior fascicle blocks, ventricular repolarization alterations, and ventricular ectopic beats. Almost all electrocardiographic abnormalities can be found in CD, in which cardiac electric activity formation and conduction alterations predominate.

Complete or incomplete RBBB is the most common conduction disorder in CD, found in 10% to 50% of infected patients, depending on the characteristics of the sample studied.^330 , 404 , 405^RBBB is frequently associated with LAFB, the most commonly found combination in CCCD. Left bundle branch block (LBBB) is rare and of worse prognosis.

Atrioventricular blocks are common, of varied degrees, and can be the first manifestation of the disease. Advanced AVBs result from extensive lesions of the atrioventricular node and His-Purkinje system, can progress with syncope and need for definitive PM implantation, predisposing to sudden death from asystole.

Sinus node dysfunction frequently expresses as bradycardia and can cause episodes of sinoatrial block and sinus arrest. Sinus node dysfunction accompanied by symptoms of reduced cerebral blood flow characterizes sinus node disease, which, in some patients, typically alternates bradycardia and tachycardia episodes.

Atrial fibrillation in CCCD is a late alteration, found in up to 5% of the ECG tracings.^330 , 332 , 404^Usually, AF is associated with a more pronounced and extensive myocardial damage, diffuse involvement of the conduction system, ventricular arrhythmias, and stroke.

Ventricular arrhythmias, such as polymorphic VE and ventricular tachycardia (VT), are predictors of syncope and sudden cardiac death due to VF. Pathological Q waves and loss of R wave progression from V1 to V3-V4 indicate inactive electrical areas and result from myocardial fibrosis. Diffuse conduction disorders and low QRS voltage are usually associated with marked ventricular dysfunction.^332^

Association of two or more abnormalities in the same electrocardiographic tracing is one characteristic of severe cardiopathy. Conduction disorders associated with ventricular arrhythmias are the most frequent. Coexistence of pathological Q waves indicates more significant impairment of ventricular function. Thus, the higher the number of electrocardiographic changes, the worse the prognosis.

Traditional epidemiological studies, assessing electrocardiographic changes in CD, have been conducted predominantly with individuals infected through classical vectorial transmission and included younger individuals.^334 , 404^With the current more comprehensive control of vectorial transmission and the *T. cruzi-* infected population aging, chronic diseases, such as hypertensive and ischemic heart diseases, can coexist with CCCD and the typical abnormalities of these conditions can overlap the typical ones of CD.^332^In addition, although there are typical abnormalities of CCCD, they are neither specific of that condition nor appear in all cases.

Regarding the relationship between electrocardiographic changes and prognosis, recent investigations by independent groups of researchers have highlighted the potential contribution of the analysis of those changes, using artificial intelligence and machine learning resources, to predict ventricular dysfunction and myocardial fibrosis, two fundamental prognostic factors in CD.^406 , 407^

When CD diagnosis is suspected or confirmed, ECG should be performed and repeated regularly to assess the appearance or progression of abnormalities. In individuals with normal ECG, new changes indicate progression to the cardiac form, requiring additional tests.^5 , 7^For patients with symptoms suggestive of cardiac arrhythmias, such as palpitations, syncope, and recovery from sudden death, an ECG at rest is mandatory before performing new tests, such as Holter, stress ECG, or intracardiac electrophysiological study (EPS).

## 6.2.2. Chest Radiography

Considering its large availability, chest X-ray is one of the most used tests to diagnose cardiovascular impairment, and mainly to assess pulmonary congestion. Even in symptomatic patients, an enlarged cardiac area with little congested pulmonary fields is often found. In addition, RV enlargement signs on posteroanterior and lateral projections are common and significant, and signs of right pleural effusion secondary to systemic congestion can be found. An increased CTI is an independent predictor of death in individuals with CCCD.^408^A recent study has shown that the presence of cardiomegaly based on the CTI can be properly identified by an increased LVDD, measured on ECHO.^337^

## 6.2.3. Echocardiography

Echocardiography is the most used imaging test for the initial assessment and follow-up of patients with CD.^339^The echocardiographic signs can vary from localized alterations in segmental contraction in the initial stages of cardiopathy to important dilation of cardiac chambers with biventricular dysfunction in more advanced stages. The presence and severity of the alterations on ECHO, in association with clinical data, are criteria used for the classification of CD in stages A to D, with an intrinsic prognostic value, as mentioned in another chapter of this guideline.

6.2.3.1. Left Ventricular Systolic Function

The DCM of CD is characterized by LV enlargement and segmental and/or diffuse hypokinesia, and LV systolic dysfunction is the most important predictor of death.^408^Because of the presence of geometrical and segmental alterations, M mode is not recommended for assessing LV dimensions and systolic function. Those should be analyzed preferably by using two-dimensional mode and estimation of volumes, with the biplanar method (Simpson). As in other cardiomyopathies, three-dimensional ECHO is superior to two-dimensional ECHO for assessing volumes and ejection fraction, mainly in suspected LV foreshortening in apical view or in the presence of segmental contraction abnormalities with distortion of the suspected geometry, such as in aneurysms frequently identified with the method.

Speckle tracking ECHO enables the early diagnosis of systolic dysfunction by assessing myocardial deformation in patients with CD. Several studies have assessed systolic deformation, in the longitudinal, radial, and circumferential axes, of patients with the IFCD or cardiopathy. The most consistent results have assessed GLS, as in other nonischemic cardiomyopathies. Even in patients in the earliest stages of cardiopathy, as those with preserved ejection fraction (stage B1) or those with the IFCD (stage A), regional alterations in myocardial deformation are observed. In patients with the IFCD, the regional alterations identified on STE occur mainly in the LV inferior and inferolateral segments.^313 , 409 , 410^The prognostic value of those early regional alterations in patients with the IFCD has not been defined. A recent study, including 144 patients with CD, but without evidence of cardiac impairment, has shown that the radial strain assessed on STE was a predictor of the development of cardiomyopathy.^411^In patients with reduced LVEF and CCCD or idiopathic DCM, reduced GLS was a predictor of combined outcomes regardless of the LVEF.^412^

6.2.3.2. Segmental Changes in Ventricular Contractility

Segmental changes can be present in 10% of patients in the CD’s early stage and in up to 50% in the presence of dilatation and systolic dysfunction. These regional wall motion abnormalities, when incipient, identify individuals at risk for progressing to global ventricular dysfunction and arrhythmias.^341 , 413^In patients with CCCD, an altered segmental motion score at rest (> 1) could identify those at higher risk for clinically relevant outcomes, such as global mortality, despite the initially preserved global ventricular function.^342^Segmental alterations are most frequently found in the inferior and inferolateral walls, in addition to the apical segments. The regional pattern of impairment, not related to the coronary territory, is characteristic of this cardiomyopathy.

Ventricular aneurysms vary in size and shape, from small finger-like (“glove finger”) to large apical (“saccular”) aneurysms, which can be hard to differentiate from those found in ischemic cardiopathy.^339^The mean prevalence of apical aneurysm in different echocardiographic series was 8.5% (ranging from 1.6% to 8.6%) in asymptomatic patients or those with mild cardiopathy and up to 55% (ranging from 47% to 64%) in patients with moderate to severe LV systolic dysfunction.^339^The aneurysms are not restricted to the apex or inferolateral wall, and can be found in the septum, anterolateral wall, and right ventricle.^340^Intraventricular thrombi can be associated with those aneurysms, being considered important risk factors for embolic events.

Although transthoracic ECHO at rest is fundamental in the CCCD assessment because it enables the identification of segmental alterations, mainly apical aneurysms, its execution can be technically challenging. The use of deep breath and non-conventional echocardiographic views, such as the intermediate between the apical 4- and 2-chamber views, with posterior angulation of the transducer, can be necessary, as well as the complementary use of ultrasound contrast imaging.

6.2.3.3. Left Ventricular Diastolic Function

The alteration in myocardial relaxation is the first to appear, and can be present even in patients with the IFCD. As the cardiomyopathy progresses, the diastolic dysfunction can worsen and show a typical restrictive pattern.^414 , 415^The diastolic function analysis can be challenging because of confounding factors, resulting from the occasional presence of AF and PM in the right chambers. The gradual increase in the E/e’ ratio occurs from the IFCD and a value greater than 15 is a predictor of worse outcome in patients with mild to moderate systolic dysfunction.^416^There is evidence that the E/e’ ratio correlates independently with serum levels of brain natriuretic peptide (BNP).^417^

Diastolic dysfunction contributes decisively to atrial remodeling, whose volume can be increased in any stage of the CCCD.^418^The left atrial volume correlates independently with mortality.^342 , 415 , 419^Left atrial function in CCCD is more impaired than in other etiologies, such as idiopathic DCM, probably because of the associated intrinsic atrial myopathic impairment.^420^When assessed by use of strain, the left atrial function also was an independent predictor of clinical events in patients with CD.^418^Similarly, left atrial dysfunction indices assessed on three-dimensional ECHO and by strain were independent predictors of recent-onset AF in those patients’ follow-up.^421^

6.2.3.4. Right Ventricular Assessment

Right ventricular assessment on conventional ECHO, using dedicated projections, allows the quantification of RV dimensions, volumes (three-dimensional ECHO), and contractile function, and should be performed in all patients with CCCD. Although frequently associated with LV dysfunction,^348^RV impairment more rarely can occur primarily and prematurely as compared to LV impairment.^345^The RV systolic dysfunction, assessed by use of conventional echocardiographic parameters, such as Tei index, was an independent predictor of poor prognosis in CCCD.^350^The RV systolic function analysis by use of STE, especially in the chamber’s free wall, showed satisfactory accuracy, correlating with other methods, such as CMRI.^347^In addition, three-dimensional ECHO is a promising tool to assess RV systolic function.

6.2.3.5. Stress Echocardiography

Pharmacological stress (and possibly exercise stress) ECHO can show the two-phase contractile reserve in those patients, who typically have no subepicardial coronary artery obstruction.^422^Although the pharmacological test usually uses dobutamine, which has an arrhythmogenic potential, the method proved safe in CCCD, and an altered segmental contraction index at rest is an independent predictor of arrhythmias during the test.^423^

## 6.2.4. Cardiac Magnetic Resonance Imaging

Although CMRI is not a test for the initial assessment of CD, the method has proven useful for CCCD diagnosis and risk stratification. Patients undergoing investigation of cardiomyopathy with no specific suspicion of CD and not residing in an endemic area frequently do not undergo serological tests for CD. In such cases, a typical global or regional systolic dysfunction pattern associated with a specific myocardial fibrosis pattern and location on CMRI can raise the suspicion and indicate the need for the specific serological test.

In addition, CMRI can estimate the prognosis. The amount of myocardial fibrosis strongly correlates with markers of disease severity, ventricular arrhythmias, severe cardiovascular events, and even death.^311 , 424^In addition, CMRI can be useful to detect early myocardial involvement in CD, mainly in the IFCD, when all other tests are usually normal.^310 , 311^

On CMRI, new noninvasive tools can identify myocardial inflammatory activity (edema and myocardial hyperemia) at an early stage before the development of irreversible lesions, such as necrosis and fibrosis, and aid risk stratification and even therapeutic decision-making.^310 , 425^

The CMRI can be useful to detect intracardiac thrombi in certain patients, especially those with limited echocardiographic images and no indication for invasive angiocardiography.^310 , 311 , 426 , 427^

Recent investigations have shown that CMRI has a good potential to assess the prognosis of patients with CCCD, independently of that provided by the RASSI score, allowing the re-stratification of those at low to intermediate risk of death.^428 , 429^The prognostic potential of CMRI in CCCD will much likely depend on confirmation by on-going studies and should corroborate the expansion of the already used risk stratification methods.^430^

The CMRI should include biventricular systolic function assessment through SSFP (steady-state free precession) techniques, T2-weighted images and/or T2 mapping for the assessment of myocardial edema, in addition to the mandatory use of late gadolinium enhancement to detect gross regional myocardial fibrosis. In addition, the pre- (native) and post-contrast myocardial T1 mapping technique should be included to calculate the myocardial extracellular volume, which is a measure of interstitial and diffuse fibrosis that can be present in this cardiomyopathy, even in myocardial regions with no evident late enhancement. The global pre- and post-contrast T1-weighted enhancement (rapid spin-echo acquisition, similarly to the Lake Louise criterion, originally for viral myocarditis) or the early gadolinium enhancement can be useful to detect hyperemia/inflammation. In addition, long inversion time late gadolinium enhancement (~ 600ms) should be used, specifically in suspected intracavitary thrombus, to increase the sensitivity of its detection.

To assess mitral or tricuspid regurgitation, usually present in advanced CCCD, cine magnetic resonance imaging (MRI) and phase contrast cine MRI (mapping sequence) are used.

It is worth noting the classical example of CCCD on CMRI, which involves the basal and medial inferolateral segments and the LV apex, with typical contractile changes on cine MRI and characteristic myocardial fibrosis pattern and distribution on late enhancement. A typical finger-like LV apical aneurysm can be clearly seen on cine and late enhancement MRI.

Recent scientific position statement on CD of the American Heart Association has recommended CMRI in certain patients with cardiopathy to assess the fibrosis extension and even serial CMRI for individuals with complex ventricular arrhythmias, especially NSVT.^7^

Another consensus document on imaging in CD of the European Association of Cardiovascular Imaging and the SBC Cardiovascular Imaging Department has recommended that CRMI should be indicated for certain patients with severe ventricular arrhythmias to quantify the extension of myocardial fibrosis and assess the risk of sudden death with potential impact on the indication for an implantable cardioverter-defibrillator (ICD). In addition, CMRI should be indicated for the assessment of LVEF when 2-D ECHO is considered unsatisfactory and contrast or three-dimensional ECHO is not available.

## 6.2.5. Nuclear Medicine

Nuclear medicine is a noninvasive imaging modality but requires the use of radiation. In CD, it can be used to assess biventricular function as an alternative to CMRI and to analyze myocardial perfusion when stenotic epicardial or microvascular coronary artery disease is suspected, in addition to assessing cardiac sympathetic innervation.^205^

6.2.5.1. Radionuclide Ventriculography

Nuclear medicine is an option for the analysis of the systolic function of both ventricles, especially in patients with contraindication to CMRI and in the rare cases in which ECHO cannot be technically performed. Radionuclide ventriculography could be considered the gold-standard method for measuring the ejection fraction of both ventricles because it allows integrated sampling of several cardiac cycles. Therefore, it minimizes the occasional variability that limits, in some circumstances, the reliability of the methods that analyze only a few cycles. In addition, radionuclide ventriculography is used to determine the diastolic and systolic volumes without resorting to geometrical assumptions, as well as to provide information on regional contractility and the presence of ventricular aneurysms, characteristic of CD.

Radionuclide ventriculography can be used, with limitations, to assess diastolic function, whose alteration can be one of the earliest manifestations of CD. In addition, RV dysfunction, which can also be an early sign of CCD, can be accurately assessed with nuclear medicine techniques, whose use in patients with CCCD, however, is still logistically limited.^205 , 431^

6.2.5.2. Myocardial Perfusion

The prevalence of obstructive coronary artery disease is not usually high among patients with CCCD, even in the presence of precordial pain. However, several researchers have independently reported coronary microcirculation dysfunction in those patients, and the presence of perfusion defects have prognostic value, because they can precede the development of myocardial contractile dysfunction.

The presence of scintigraphic changes in patients with CCCD can translate the inflammatory mechanism through which, at least partially, the cardiac muscle is destroyed in CCCD and replaced by fibrous tissue. Myocardial perfusion imaging by single-photon emission computed tomography (SPECT) is effective in detecting cardiac muscle perfusion disorders, even when lesions in epicardial coronary arteries are absent.^182^

With lower logistic availability, PET/CT is an alternative test to study inflammatory and perfusion alterations, as well as myocardial viability loss or preservation, in ventricular areas that show contractile deficit, being a suitable test for microcirculation study.^431^

6.2.5.3. Sympathetic Innervation Assessment

Depression of the myocardial sympathetic innervation occurs at the ventricular level early in CD and maybe at higher intensity than in other heart diseases. This can be associated with the loss of reflex autonomic control and even precede any other cardiac impairment. Nuclear medicine, by use of I-MIBG scintigraphy,^123^enables the detection of ventricular sympathetic innervation defects, especially in the inferior, posterolateral, and apical walls, long before any contractile defect in those segments appears.^183^These myocardial innervation alterations might be associated with a higher risk for SVT and worse prognosis.^205 , 208^

## 6.2.6. Computed Tomography of Coronary Arteries

Computed tomography of coronary arteries, similarly to invasive angiocardiography based on cardiac catheterization, uses ionizing radiation and iodine contrast medium, being primarily used for the noninvasive study of coronary anatomy in several clinical contexts.

In CD, the experience with this approach in clinical practice is limited and applies to patients with contraindication to other imaging methods, such as CMRI and myocardial scintigraphy, in whom the echocardiographic study shows technical limitations.^432^Computed tomography of coronary arteries in general is more indicated when the probability of obstructive subepicardial coronary artery disease is low but should be ruled out in patients with CCCD and atypical precordial pain.

Preliminary experience with the method has shown that Brazilian patients with CD have a reduced prevalence of obstructive subepicardial coronary artery disease, thus corroborating older evidence supported by invasive angiographic studies.^269^

## 6.2.7. Ambulatory Electrocardiography (Holter)

The pathogenesis of CCCD is complex and multifactorial, includes tissue damage by the parasite and exacerbated immune response, leading to inflammatory reaction, autonomic nervous system involvement, and microcirculation impairment. The result of these pathogenetic mechanisms is cell necrosis and their replacement by localized areas of myocardial fibrosis.

The zones of fibrosis have predilection for the conduction system (sinus node, atrioventricular node, and His-Purkinje system branches and fascicles),^433^being usually a primordial manifestation of cardiac involvement. The combination of areas of fibrosis, autonomic dysfunction, and conduction system impairment favors the occurrence of both bradyarrhythmias and tachyarrhythmias, sometimes before cardiac structural alterations are detected on imaging tests, such as ECHO. This early manifestation of arrhythmias in CCCD characterizes the disease as an arrhythmogenic cardiomyopathy,^434^whose first clinical manifestation can be sudden death.^353^In fact, sudden cardiac death is the major cause of death in CCCD, accounting for approximately 60% of the deaths.^352^

The detection of cardiac arrhythmias on Holter or during exercise testing is an essential part of the routine assessment of patients with CCCD, enabling the diagnosis of sinus node dysfunction, atrioventricular conduction disorders, supraventricular tachyarrhythmias and ectopic beats, ventricular ectopic beats, and NSVT or SVT.

A study assessing the occurrence of ventricular ectopic beats on Holter in patients with CCCD has shown that the apparently random behavior of that arrhythmia in 24-hour recordings disappears when longer periods, of 7 days, are analyzed, suggesting that longer Holter recordings would be more suitable in this context.^435^

The presence of NSVT on Holter is an independent predictor of all-cause mortality in patients with CCCD.^408 , 436^In the RASSI score, the identification of NSVT on Holter adds 3 points of a total of 18 or 20 possible points (women and men, respectively). In addition to risk stratification, Holter enables the assessment of symptoms, such as palpitations and syncope, frequent in those patients and usually resulting from the arrhythmias found.

Holter also enables the autonomic nervous system assessment by use of heart rate variability (HRV) analysis. Several studies have shown autonomic alterations in different stages and forms of CD.^437 , 438^Although parasympathetic dysfunction predominates, sympathetic involvement (lower intensity) is also present.^204^A retrospective study has reported preliminary evidence that such alterations, reflected in several HRV parameters, can signal the risk for sudden death.^439^The HRV assessed during short Holter recordings and with machine learning technique has shown the ability to predict echocardiographic alterations^440^and could be correlated with the RASSI score, the most endorsed prognosticator of mortality risk, in patients with cardiomyopathy with or without associated digestive involvement.^441^

## 6.2.8. Intracardiac Electrophysiological Study

In CCCD, there are reentry arrhythmogenic substrates related to areas of fibrosis, and the EPS enables the induction of SVT or even VF, which, in some contexts, gain a prognostic conotation.^442^In addition, the EPS allows the sinus node and atrioventricular conduction assessment, as well as defining accurately whether the dromotropic disorder is in the atrioventricular node or His bundle, or is infra-Hisian. The frequent paroxysmal occurrence of TAVB due to the His-Purkinje system impairment, consequent to its well-known conduction behavior in the “everything or nothing” form (either conducts or not, without intermediate stages of poor function), determines that, in certain cases, only the invasive investigation with EPS allows the accurate diagnosis and proper treatment.^442^

## 6.2.9. Exercise and Cardiopulmonary Tests

The conventional maximum exercise test and the cardiopulmonary assessment test can detect important alterations, such as ventricular arrhythmias induced by exercise and chronotropic incompetence.^405 , 443^However, the general clinical applicability of the exercise test has not been well established, although the cardiopulmonary test, with direct measurement of oxygen consumption (VO_2_max), can be considered gold-standard for assessing physical functioning and efficacy of rehabilitation programs.^444^

Exertion-induced ventricular arrhythmias are a marker of cardiovascular death risk in patients with CD.^443^Because these arrhythmias also occur in patients with no apparent cardiopathy, conventional maximum exercise test is clinically relevant for risk stratification in the population with CD, especially regarding work legal advice.

Few studies have assessed the efficacy of exercise test variables in predicting the survival of patients with CCCD. The VO_2_peak is an important criterion for CTX in patients with advanced forms of cardiopathy. However, its prognostic value should be better understood in the context of preventive strategies, risk stratification, and early diagnosis. In addition, cutoff points should be established to be specifically used in CCCD.

## 6.2.10. Cardiac Catheterization

As already mentioned, patients with CCCD frequently have atypical chest pain and electrocardiographic abnormalities, such as ST-segment changes and pathological Q waves, in addition to regional contractile and myocardial perfusion disorders that mimic coronary atherosclerotic disease. In most cases, assessment of epicardial coronary arteries shows no obstructive subepicardial atherosclerotic disease, those changes being attributed to coronary microvascular dysfunction.^336 , 445^

Recent studies have evidenced that the ventricular dysfunction associated with CD microvascular disease is more prominent than that observed in the microcirculatory disorder from other etiologies.^446^In addition, researchers have reported improvement in symptoms and myocardial perfusion when patients with CCCD were treated with platelet inhibitors and microvascular vasodilators, the first demonstration of benefit achieved in this context.^447^

Cardiac catheterization, thus, can be used when patients with intermediate or high probability of obstructive coronary artery disease have typical anginal pain and/or multiple risk factors for atherosclerotic disease or a large ischemic area evidenced on noninvasive tests. During the hemodynamic study, contrast left ventriculography, because of its high temporal and spatial resolution, can show small apical aneurysms and/or other segmental ventricular contractile changes, which might not be detected on other imaging methods.^336^In addition, cardiac catheterization can be performed in candidates to CTX due to advanced HF to assess pulmonary vascular resistance. Moreover, cardiac catheterization enables performing post-transplant endomyocardial biopsy when the differentiation between rejection and *T. cruzi-* infection reactivation is mandatory in some patients.

## 7. Risk Stratification and Prognosis

Chronic cardiomyopathy of Chagas disease can manifest in several forms, depending basically on the severity of the myocardial and electrical conduction system alterations, presence and type of arrhythmias, and existence of HF. Two systematic reviews with meta-analyses have been recently published. One of them has assessed the risk of progressing to chronic cardiomyopathy in individuals in the acute phase (global estimated occurrence of 4.6% [95% CI, 2.7%-7.9%] annually) or in those with the IFCD (annual estimate of 1.9% [95% CI, 1.3%-3.0%]).^297^The other systematic review with 52 studies including only patients with manifest cardiopathy has shown an annual mean rate of mortality of 7.9% [95% CI, 6.3%-10.1%], but with highly heterogeneous results, and individual rates ranging from 0.5% to 38.3%/year, depending on the baseline characteristics of each study population^448^( Figure 7.1 ).

**Figure 7.1:** – Annual progression rate of Chagas disease (diagnosed acute phase and chronic indeterminate form) to cardiomyopathy and from chronic cardiac form to death.

In recent decades, several risk factors for morbidity and mortality have been identified to quantify the severity of CCCD, assess its prognosis, and suggest more suitable therapeutic strategies. However, when considered in isolation, variables associated with worse prognosis usually have low positive predictive value, which limits their use. Thus, prognostic models built from several combinations of demographic, clinical, and laboratory parameters began to be investigated.

To be applied in clinical practice, the risk stratification model should be simple and use a non-excessive number of variables that are well defined, easily accessed, and have satisfactory discriminatory power (C statistics). Even more important, the model should be validated by researchers from other centers (geographical validation) and in posterior periods (temporal validation), and, if possible, be capable of predicting other outcomes different from that to which it was developed and in different scenarios (wide or expanded validation).^449^

It is worth emphasizing that prognostic models without external validation, even if properly developed, are of little use and have low evidence-based sustainability, being not recommended for daily practice use. Usually, the prognostic model performs better in the data set from which it originated than in the new data set in validation analyses.^449^

In 2006, Rassi Jr *et al* .^408^developed and validated a risk score to predict all-cause death in CCCD. In an original cohort involving 424 outpatients with a mean 7.9-year follow-up, total mortality was 31% (130/424), deaths due to cardiovascular causes represented 87% of the total (113/130), and sudden cardiac death represented 62% of the total (81/130). In the external validation cohort (153 patients), the total mortality rate was 23% (35/153) in a mean 7.7-year follow-up, and most deaths (57%) occurred suddenly.

Multivariate analysis identified six independent predictors of mortality, which were attributed points corresponding to their strength of association with the outcome in question (total mortality) based on the Cox model beta regression coefficient ( Figure 7.2A ). The individuals were classified into subgroups of low, intermediate, and high risk, based on the total sum of each patient’s points. Total mortality in 10 years and the survival curves of those three subgroups are shown in Figure 7.2B and 7.2C . The C statistics for the system of points was 0.84 in the development cohort and 0.81 in the validation cohort. Except for male sex for cardiovascular death and low QRS voltage for sudden death, which showed borderline statistical significance, the other variables were also strong predictors of risk for those two specific types of death.^408^

**Figure 7.2:** – RASSI score. (A) Risk markers and respective points; (B) Total mortality in 10 years in the low-, intermediate-, and high-risk subgroups; (C) Kaplan-Meier actuarial curves. pts: points, NSVT: Nonsustained ventricular tachycardia.

In addition to showing unequivocal discriminatory power, the RASSI score has the following advantages: uses only six easily-measurable or collectable variables, extracted from usually available tests (ECG, chest X-ray, two-dimensional ECHO, and 24-hour Holter), which are part of the initial mandatory investigation of patients with CCCD; assesses LV function subjectively, dismissing ejection fraction measurement by the Simpson method and valuing both global and segmental myocardial contractility changes, which have recently been confirmed as important independent predictors of risk for cardiovascular events by use of careful analysis of the BENEFIT study database;^342^enables replacing the CTI measurement on chest X-ray by LVDD measurement on ECHO, because a good correlation between CTI > 0.50 and LVDD > 60mm has been observed;^337^requires the use of neither formulas nor calculators because it is a feasible score of simple memorization; can predict the three major causes of death: total, cardiovascular, and sudden;^408^and was externally validated in four different cohorts, at different times, and by independent researchers.^408 , 424 , 450 , 451^Of note in two of those cohorts,^424 , 451^the outcome assessed was different from that of the original publication (total death), and, even so, the RASSI score showed highly reproducible results ( Table 7.1 ).

**Table 7.1:** – RASSI score: results of the original cohort (Hospital São Salvador, Goiânia) and external validation in four different cohorts.

Author	Study period	Study site	N of patients	Outcome	% Outcome (5 years)			% Outcome (10 years)			C Statistics
					low risk (0-6 pts)	intermediate risk (7-11 pts)	high risk (12-20 pts)	low risk (0-6 pts)	intermediate risk (7-11 pts)	high risk (12-20 pts)	
Rassi A Jr. et al. ^3^	1986-1991	Hospital São Salvador (Goiânia)	331*	Total death	2 (0-5)	18 (8-28)	63 (51-75)	10 (5-14)	44 (31-57)	84 (74-93)	0.84 (0.79-0.89)^†^
Rassi A Jr. et al. ^3^	1990-2001	Hospital Evandro Chagas (RJ)	153	Total death	0	15 (1-28)	53 (31-75)	9 (2-16)	37 (16-59)	85 (63-100)	0.81 (0.72-0.90)^†^
Rocha MOC & Ribeiro AL ^6^	1998-2006	Universidade Federal de Minas Gerais	158	Total death	3 (1-7)	10 (4-22)	67 (30-90)	NA	NA	NA	0.84 (0.72-0.96)
Benchimol Barbosa PR et al. ^7^	1995-2003	Hospital Universitário Pedro Ernesto (RJ)	100	Cardiac death or VT^§ //^	4 (1-11)^¶^	42 (18-83)^¶^	50 (6-100)^¶^	28 (18-43)	58 (29-100)	75 (15-100)	0.79 (0.70-0.88)^†^
				Cardiac death^#^	NA	NA	NA	NA	NA	NA	0.81 (0.69-0.93)^†^
Senra T et al. ^8^	2001-2011	Instituto do Coração - INCOR (SP)	130	Total death, cardiac transplantation, ICD appropriate therapy or resuscitated CA^//^	16	42	76	NA	NA	NA	NA
				Total death^#^	11	33	57.5	NA	NA	NA	NA

The numbers between parentheses correspond to the 95% confidence interval. * multivariate model applied to 331 patients from the original cohort of 424 patients (those with missing data were excluded);^†^relative to 10 years;^§^defined as 3 or more successive beats;^//^primary outcome;^¶^outcome by 50 months;^#^secondary outcome; ICD: implantable cardioverter-defibrillator; N: number; NA: not available; CA: cardiac arrest; VT: ventricular tachycardia.

The RASSI score strength, particularly regarding its accuracy for stratification in subgroups of risk, is supported by results of recent investigations in different contexts, showing, for example, a strong positive correlation of the risk levels with the following: cardiac dysautonomia grade;^441 , 452^myocardial fibrosis presence and extension on CMRI detected with late enhancement technique^424 , 428 , 453^or T1 mapping,^425^the latter assessing the interstitial component of myocardial fibrosis; and the induction of sustained ventricular tachyarrhythmias on EPS.^454^

In another study, assessing patients who had undergone cardiopulmonary exercise testing, the RASSI score addition to the anaerobic threshold increased the area under the ROC curve from 0.706 to 0.800, and all-cause death was the primary outcome on logistic regression analysis.^455^

When assessing the prevalence and prognostic value of ventricular dyssynchrony on ECHO and of the RASSI score in patients with CCCD, considering as outcome the combination of total death and hospitalization, only the RASSI score could predict the combined events on multivariate analysis (OR = 1.19; 95% CI, 1.02-1.40; p = 0.01).^456^

In patients with HF and LVEF< 45%, when assessing the prognostic value of the variables obtained on cardiopulmonary exercise testing and of other variables, only the increased VE/VCO_2_slope (HR = 2.80; 95% CI, 1.30-5.80; p = 0.001, cutoff point of 32.5) and the RASSI score (HR = 1.28; 95% CI, 1.10-1.48; p = 0.001) associated with higher mortality on multivariate analysis after a mean 32-month follow-up.^457^

A systematic review of 12 studies (1985 to 2006),^458^using multivariate analysis to better assess prognosis in CCCD and comprising approximately 4300 patients, has shown that the most consistent and relevant predictors of total mortality, sudden cardiac death, and cardiovascular death were New York Heart Association (NYHA) functional class III or IV, cardiomegaly on chest X-ray, LV systolic dysfunction assessed on ECHO or cineventriculography, in addition to NSVT on 24-hour Holter. Using these four variables in an integrated way, an algorithm can be elaborated to stratify the risk of death in patients with CD in a simplified and logic way by use of clinical parameters and complementary methods available in most cardiologic services in our country ( Figure 7.3 ).

**Figure 7.3:** – Risk stratification algorithm in Chagas disease. NSVT: nonsustained ventricular tachycardia ; LV: left ventricular. *can be replaced by LV diastolic diameter > 60 mm on echocardiogram; †global or segmental. Adapted from Rassi A Jr, Rassi A, Rassi SG. Predictors of mortality in chronic Chagas disease. Circulation. 2007;115:1101-8. ^458^

The presence of NYHA functional class III or IV, *per se* , identifies high-risk cases, because almost all these patients have systolic ventricular dysfunction on ECHO and NSVT on Holter. Of note, the combination of ventricular dysfunction with NSVT, independently of the functional class, identifies a group with a 15-fold higher risk as compared to patients without both abnormalities.^458^

Although the RASSI score has a well-established theoretical base as an independent predictor of fatal events and has been externally validated in multiple studies, it is infrequently used on the daily practice. One of the reasons for that might be the low availability in the Brazilian SUS, outside university-affiliated hospitals, of the simple diagnostic methods used for calculating the score, such as ECHO and 24-hour Holter. This guideline, by strongly recommending the application of the RASSI score as the principal method for risk stratification of all patients as soon as the diagnosis of cardiomyopathy is confirmed, as already established in other consensus of international societies,^459^expects to solve that deficiency.

In addition, despite projecting the risk of death in the long run and under highly heterogeneous prognostic conditions, the RASSI score usefulness to guide clinical management and subsequent therapy is yet to be determined. It is worth noting that the valuable information on risk provided by the score to patients and their physicians can guide follow-up and, possibly, treatment strategies. In this way, the Brazilian multicenter RCT CHAGASICS ( *CHronic use of Amiodarone aGAinSt Implantable Cardioverter-defibrillator* ) is in its conclusion phase. It compares amiodarone *versus* defibrillator for total mortality reduction as a primary prevention strategy and has the following inclusion criteria: presence of at least one NSVT episode on 24-hour Holter and a RASSI score ≥ 10 points.^460^

Furthermore, it is plausible to speculate that low-risk patients (10-year mortality of 10%, similarly to that of the low-risk group according to the Framingham score) can undergo annual clinical reviews, while intermediate- or high-risk patients should do it more often (every 3 or 6 months).

Other studies on the CCCD prognosis have focused on different risk markers, such as: LVEF reduction;^461 - 463^RV systolic dysfunction (Tei index);^350^LV diastolic dysfunction (E/e’ ratio);^416^left atrial volume increase;^415^changes in myocardial deformation indices;^412 , 464^parasympathetic and sympathetic dysfunction;^207 , 465^specific changes on ECG;^303^T-wave amplitude variability;^466^T-wave axis deviation;^467^QT interval dispersion;^463 , 468^changes on high-resolution ECG (spectral turbulence and filtered QRS);^436 , 451^HRV reduction;^451^prolonged QRS complex duration;^469^VO_2_peak reduction;^461^exercise time reduction;^470^increased serum levels of type B natriuretic peptides (BNP and NT-proBNP);^469 , 471^and others.^472^Such factors and variables, when analyzed by use of multivariate models or transformed into risk scores, associate with worse prognosis. In addition, they provide information on the mechanisms related to disease progression and the less explored aspects of their complex prognostication.

However, as the above mentioned studies are very heterogeneous, acknowledging their limitations is necessary.^473 , 474^The following limitations affect their applicability: use of non-standardized variables that are difficult to measure and to reproduce, usually extracted from complementary tests of restrict access or not available in common practice; inclusion of a reduced number of patients or outcomes; non-inclusion of all variables known to be associated with worse prognosis in most of those models (the four previously cited); and, particularly, absence of external validation.^473^

Thus, the scores proposed are not ready to be used in routine medical care, because almost all studies lack external and independent validation.^473 , 474^It seems there is a growing number of publications trying to develop new risk models rather than validating or improving the existing ones.^474^

A recent study, using data from the NIH SaMi-Trop cohort,^475^has developed a simplified score to be used in endemic regions without access to cardiological investigation other than ECG. The score included clinical and electrocardiographic data, in addition to NT-proBNP measurement, to predict the risk of death in 2 years of patients with CCCD.^469^Five independent predictors of death were identified and points were attributed as follows: age (10 points per decade); NYHA functional class greater than I (15 points); HR ≥80 beats/min (20 points); QRS duration ≥ 150ms (15 points); and abnormal age-adjusted NT-proBNP (55 points). Patients were then classified into three risk categories (low, < 50 points; intermediate, between 50 and 100 points; and high, > 100 points). External validation was performed applying the score to another independent population with CD. After a 2-year follow-up, in the development cohort, 110 patients died, and the global mortality rate was 3.5 deaths per 100 individuals-year. The mortality rates observed in the groups of low, intermediate, and high risk were 0%, 3.6%, and 32.7%, respectively, in the derivation cohort, and 3.2%, 8.7%, and 19.1%, respectively, in the validation cohort. The score discrimination was good in the development cohort (C statistics: 0.82) and the validation cohort (C statistics: 0.71).^469^The major limitations of the score are the use of NT-proBNP measurement, which is not usually available in PHC, and lack of independent and extensive external validation, as occurs with the RASSI score.

In two other studies, the mere identification^428^or quantification^424^of myocardial fibrosis with the late enhancement technique in CMRI, as a continuous variable (expressed in unitary value of additional gram) or as dichotomous variable (using the cutoff point of 12.3 g), proved to be an important risk predictor for severe cardiovascular events, such as total death, cardiovascular death, and occurrence of sustained ventricular tachyarrhythmias, independently of the ventricular function and RASSI score. It is worth noting that one of the studies^424^enabled the direct comparison between the prognostic value of the myocardial fibrosis amount and the RASSI score.

Using total mortality as the final outcome (considered secondary outcome), after a median 5.4-year follow-up, the power of association of the RASSI score was more significant than that of myocardial fibrosis. Expressed as categorical variables (low, intermediate, and high risk for the RASSI score and mass < 12.3 and ≥ 12.3g for myocardial fibrosis), only the RASSI score was associated with worse prognosis (HR: 1.24; 95% CI, 1.13-1.36; p < 0.001 *versus* HR: 1.33; 95% CI, 0.68-2.61; p = 0.406). Expressed as continuous variables (RASSI score in points and myocardial fibrosis in grams), both were risk predictors, but with higher relevance for the RASSI score (HR: 1.23; 95% CI, 1.12-1.35; p < 0.001 *versus* HR: 1.02; 95% CI, 1.00-1.04; p = 0.043), that is, for each additional point in the RASSI score, the risk of death increases by 23%, while for each 1 additional gram of myocardial fibrosis, that increase is of only 2%. The mass of fibrosis, as dichotomous variable, had a C statistic of 0.709 (95% CI, 0.618-0.793) to predict death from any cause, while, for the RASSI score, that value was not informed.^476^

Another relevant result of that study was the ability of the RASSI score and myocardial fibrosis to predict the combined outcome of death from any cause, CTX, resuscitated sudden death, appropriate shock or ICD antitachycardia therapy, a less important outcome from the risk hierarchical viewpoint, but considered the primary outcome in the study in question. Despite these expressive results with the RASSI score,^477^the authors concluded only that myocardial fibrosis could contribute to improve risk stratification and might guide the treatment of patients with CCCD.^424^

In the assessment of CCCD, CMRI is not one of the initial diagnostic tests and might not be included in the routine cardiological assessment because of its high cost, unavailability in several centers, and some relative or absolute contraindications to its performance (for example, claustrophobic patients, individuals with orthoses/prostheses, older PM models, or renal failure when gadolinium injection is required). Also, the method should still be tested to assess if it can improve the performance of the already existing risk stratification models by use of new statistical techniques, such as reclassification table, net reclassification index (NRI) and integrated discrimination index (IDI).^478^

According to the prevalence of the risk groups in the RASSI score, it is known that if it is applied to 1000 patients with cardiomyopathy, 610 will be classified as at low risk for total death, 190 as at intermediate risk, and 200 as at high risk. With death rates in 10 years of 10%, 44%, and 84%, respectively, for the three subgroups,^408^by the end of 10 years we would have 61 deaths in the low-risk group, 84 deaths in the intermediate-risk group, and 168 deaths in the high-risk group. Thus, of a total of 313 deaths in the 10-year follow-up, although most deaths (168 or 54%) occur in the high-risk group (which is desirable in terms of risk stratification), we would still have 145 deaths in the low- and intermediate-risk groups. For a new risk predictor to prove its clinical usefulness, once added to the RASSI score, it should ideally be able to correctly identify the low- and intermediate-risk patients who will die or, less likely, those at high risk who will survive. Myocardial fibrosis might be that marker, a hypothesis yet to be tested.

In addition, there is no report in the literature that a change in the RASSI score (particularly a reduction in the number of points) can assess and monitor the efficacy of a certain treatment and improve patients’ prognosis. However, because the score includes six variables, attributing 0 to 20 points to the set, and two variables are more likely to undergo changes (NYHA functional class III/IV and presence of NSVT on Holter), this is another interesting investigation to be considered.

Finally, it is worth emphasizing that patients aged > 70 years, or with artificial cardiac PM, or documented SVT or VF, by being *a priori* at high risk for death, as well as patients with ischemic, hypertensive or associated valvular disease, to prevent confusion with deaths not related to CCCD, were excluded from the calculation and standardization of the RASSI score.^408^

The prognostic value of the EPS in patients with CCCD is yet to be established. Regarding primary prevention of sudden death, data available suggest that the EPS has no prognostic usefulness in patients with isolated VE or NSVT, provided the LV systolic function is normal. In a study including 72 patients with preserved LV function (mean ejection fraction of 0.60) and 400 to 1200 VE/hour on Holter (35% with NSVT), the programmed ventricular stimulation did not induce SVT in any patient.^479^In the mean 36-month follow-up, only 1 of the 72 patients presented spontaneous SVT.

Another study^480^has assessed the prognostic value of SVT induction in response to programmed ventricular stimulation in 78 patients with NSVT on Holter (mean LVEF of 0.47 ± 0.18) and no clinical history of sustained arrhythmias. Monomorphic SVT was induced in 25 patients (32%), all of them treated with class III antiarrhythmic drugs, most of them with amiodarone and only one with sotalol. After mean 56-month follow-up, the probabilities of cardiac death and combined events (cardiac death, spontaneous SVT, or syncope recurrence) were 2.2 and 2.6 times greater (p < 0.05), respectively, in inducible patients as compared to non-inducible. In contrast, the induction of polymorphic VT or VF had no prognostic significance, being, probably, an unspecific ventricular response to the test.^480^

Regarding secondary prevention (patients with documented sustained ventricular arrhythmias or resuscitated sudden death), some authors have assessed the importance of EPS for risk stratification and antiarrhythmic therapy choice, but data available are limited.

The largest observational study^481^has included 115 patients with symptomatic VT (mean LVEF of 0.49 ± 0.14), of whom, 78 with spontaneous SVT and 37 with spontaneous NSVT and EPS-induced SVT. After impregnation with Vaughan-Williams class III antiarrhythmic drugs (sotalol or amiodarone), the patients were divided into three groups, based on their responses to a subsequent electrophysiological test. Patients in group 1 had no inducible SVT, those in group 2 had well-tolerated inducible SVT, and those in group 3 had hemodynamically unstable inducible SVT. After a mean 52-month follow-up, the total mortality rate was significantly higher in group 3 as compared to groups 1 and 2 (69% *versus* 26% and 22%, respectively).^481^

Based on those results, although the EPS can identify patients at higher risk for death or who do not respond well to treatment with antiarrhythmic drugs, its role in guiding other types of therapies, such as ICD implantation, remains undefined, resulting in little usefulness of the method for that purpose.

## 8. Therapeutic Management in the Indeterminate Form of Chagas Disease

The IFCD is a latent period that usually begins right after the end of the acute phase and can last indefinitely, for an individual’s entire life. This stage of CD has been recognized since the first studies by Carlos Chagas^229 , 304 , 482^and was classically confirmed in 1985,^301^when it was defined as the situation of an asymptomatic chronically *T. cruzi-* infected individual, with normal physical examination and no changes on chest X-ray, conventional ECG, and esophageal and colonic contrast radiography.

The classical definition of the IFCD does not include individuals with “unspecific” electrocardiographic changes, which do not define CCCD.^483 , 484^In this situation, the term “without apparent cardiopathy” should be used instead. Similarly, that classical denomination does not apply to patients without digestive symptoms, who had undergone neither esophageal nor colonic assessment by use of contrast imaging tests.

Chart 8.1 shows changes that usually define the presence of CCCD and those that alone are not sufficient to make that diagnosis, being considered “non-defining”. These “unspecific” changes should be cautiously interpreted, taking into consideration the underlying clinical context.^328 , 485^For example, low QRS complex voltage in the frontal plane, which indicates poor prognosis according to the RASSI score, can also be detected in individuals with emphysema or morbid obesity ( Chart 8.1 ).

**Chart 8.1:** – Electrocardiographic changes of Chagas disease

Defining of CCCD	Non-defining (unspecific)^*^
Sinus bradycardia ≤ 40 bpm	Sinus bradycardia > 40 bpm
Polymorphic ventricular extrasystole	Isolated ventricular extrasystole
Complete right bundle branch block	Incomplete right bundle branch block
Primary ventricular repolarization abnormalities	Secondary ventricular repolarization abnormalities
2^nd^and 3^rd^degree atrioventricular block	1^st^degree atrioventricular block
Complete left bundle branch block	1^st^degree left bundle branch block
Electrically inactive area	Mean QRS electrical axis deviation to the left
Sinus node dysfunction	Sinus arrhythmia (non-respiratory)
Nonsustained and sustained ventricular tachycardia	Sinus tachycardia
Atrial fibrillation	Left anterior fascicular block
Atrial flutter	Low-voltage QRS
Ventricular fibrillation	Wandering atrial pacemaker

*The ‘unspecific’ changes, when isolated, are usually considered insufficient to make the diagnosis of chronic cardiomyopathy of Chagas disease (CCCD). When combined, however, they make that diagnosis more likely. For example, ventricular extrasystole in association with low-voltage QRS and intraventricular conduction disorders (even without complete block) should suggest the diagnosis of CCCD. Individual situations of uncertainty in grey zones of ECG changes may exist and should be judiciously clarified by the physician. Adapted from Biolo et al. ^485^

In addition, it is worth noting that a normal ECG finding, despite being a defining criterion of IFCD, is not a reliable absolute indicator of absence of cardiac impairment. When deepening the investigation by using other diagnostic methods, such as ECHO,^312 , 313 , 486 - 489^exercise or cardiopulmonary test,^490 , 491^24-hour Holter,^413 , 492 - 496^noninvasive autonomic function tests,^236 , 405 , 438 , 497 - 499^cardiac scintigraphy,^183 , 345 , 500 , 501^hemodynamic and coronary angiographic studies,^502^CMRI,^311 , 453 , 503 , 504^and even endomyocardial biopsy,^505^a substantial number of patients with normal ECG shows abnormalities in some of those tests that are usually mild or of low intensity or of isolated frequency.

Most of those test abnormalities with little if any clinical repercussion can be occasionally found in healthy individuals not infected with *T. cruzi* .^506 - 508^Thus, although some studies have evidenced those changes in some individuals, the concept of IFCD remains valid,^299^being practical and widely applicable.^300^The proposal for modifying the criteria – including replacing chest X-ray with transthoracic ECHO at rest – did not move forward.^306^

However, although some studies have shown that those alterations do not impact progression to CCCD,^211 , 509^thus supporting the notion that they lack prognostic connotation, most studies’ results require better assessment in a longer follow-up. So far, there is no definitive proof that those changes do not act as potential triggers for future cardiovascular events.

Despite decades of research, the factors that lead to the development of CCCD in 30% of the individuals with the IFCD have not been totally clarified.^176^Several factors are involved in the risk for IFCD progression to CCCD, such as: age; male sex; geographical origin; parasite load intensity; *T. cruzi* strain and its discrete typing units (TcI–TcVI and Tc-bat); host’s genetic aspects; severity of the initial acute infection related to the transmission mode; exposure to reinfection with the parasite in sustained vectorial transmission areas; host’s nutritional status and presence of comorbidities; social context; quality of life of individuals with CD; and absence of trypanocidal treatment.^247 , 297 , 510 - 517^

Although knowledge on the natural history of CD is scarce, it is worth emphasizing the usually good prognosis of IFCD,^509 , 518 , 519^with mortality overlapping that of the non-infected general population, as long as the ECG findings are normal.^404^An individual with IFCD can remain for several decades in that condition,^299 , 300^and annual or even biannual serial ECG can detect progression to CCCD.^2^

Electrocardiographic changes can appear during follow-up in varying percentages, but with no direct relation to LVEF, which usually remains unaltered.^211^The annual rates of progression from IFCD to CCCD vary from 0.3% to 10.3% (mean of 1.9%).^297^In IFCD, the presence of changes on ECHO due to regional dyssynergies, even when the global systolic ventricular function is preserved, can imply a risk for clinical events, such as TAVB, stroke, ventricular tachyarrhythmias and/or HF, indicating worse prognosis as compared to that of individuals in the IFCD with normal ECHO findings.^341 , 418^

The good prognosis of patients with the IFCD has been reported in several longitudinal studies that concluded that individuals with the IFCD and controls not infected by *T. cruzi* in the same age group have similar mortality rates.^299 , 404 , 519^The annual incidence of sudden death among individuals with CD and normal ECG is low and similar to that of the population without CD.^520^Sudden death is a rare complication that occurs in the general population equally; thus, its cause should not straightaway be atributted to CD.

Regarding treatment with trypanocidal drugs in the chronic phase of CD, the IFCD is one of the major indications,^8 , 60^and young adults treated with those drugs less often progress to CCCD as compared to non-treated ones.^320 - 323 , 521^

The follow-up of individuals with the IFCD should be maintained at the PHC level, with annual or biannual electrocardiographic assessment, because some electrocardiographic changes have an evolutive character and define the CCCD.^522^A patient with normal ECG and segmental ventricular contractile abnormalities on ECHO should undergo the same investigative assessment given to a patient with an electrocardiographic abnormality that defines CCCD.

Patients with the IFCD can have comorbidities whose frequency increases as the population ages.^523^In patients with IFCD, the relation between aging and comorbidities seems not to depend on the presence of CD itself.^524^However, old patients with IFCD constitute a particularly vulnerable population group regarding the harmful effects of chronic degenerative diseases.^525^Among cardiovascular comorbidities, systemic arterial hypertension (SAH) predominates, followed by coronary artery disease.^526^The monitoring and treatment of these comorbidities, in addition to dyslipidemia and diabetes *mellitus* , should be individualized. Controlling these conditions is fundamental to the secondary prevention of CCCD.^527 , 528^

The general medical management of an individual chronically infected by *T. cruzi* (confirmed by at least two positive serological tests with different laboratory techniques) should be conservative aiming to characterize IFCD and establish the following recommendations: 1) In the absence of cardiovascular and digestive symptoms (particularly, dysphagia and constipation) with normal findings on physical examination and ECG (preferably with 30-sec recording acquired on a single lead), no additional test is necessary, not even chest, esophageal, and colonic radiography; 2) Directed anamnesis, physical examination, and ECG should be repeated annually or biannually; 3) Physical exercises should not be restricted, not even competitive ones; 4) No professional restriction applies, not even for the conduction of collective vehicles; and 5) Psychological support is essential, explaining the favorable prognostic notions, which guide the most conservative medical managements.

In patients with IFCD, performing ECG annually or biannually is strongly recommended, with level of evidence B. It is worth emphasizing that trypanocidal treatment with benznidazole should be offered to individuals with the IFCD up to the age of 50 years, as a strong recommendation, level of evidence B.

## 9. Etiological Treatment of Chagas Disease

### 9.1. Introduction

Ensuring effective, efficient, and safe access to etiological treatment (trypanocidal) for *T. cruzi* infection remains a critical challenge when analyzing the advances over the past 50 years.^2 , 3 , 38 , 44 , 56 , 58 , 112 , 113 , 529^The importance of that type of treatment for CD is unequivocal for individuals affected and for their families and communities. This is a central issue for the national health systems, and barriers should be overcome to ensure access to diagnosis and proper treatment to all patients.^2 , 5^This ethical dilemma requires a more proactive behavior of healthcare managers and professionals (particularly physicians, including cardiologists), social movements, and all stakeholders.

Chagas disease belongs in the large group of NTD, and critical flaws of science, market environment, and public health make its management even more challenging.^49 , 52 , 530^Advances in the field have been insufficient for a consistent public health response aimed at controlling the disease in the local health care network of several countries.^44 , 54 , 94 , 531 - 533^In many locoregional scenarios, diagnostic methods and therapeutic drugs are not available, and the local populations are not properly informed about them.

For the past five decades, there has been a huge limitation of etiological treatment options, with availability of only two drugs that proved effective, benznidazole (1971) and nifurtimox (1965).^1 , 2 , 5 , 8 , 44 , 54 , 377 , 534 - 536^There is strong evidence that both drugs are effective in reducing disease’s duration and clinical severity by enabling the elimination of parasites with early treatment,^1 , 2 , 8 , 44 , 46 , 60 , 377 , 379^with potential gains in quality of life through prevention of physical functioning limitations.^43 , 537^

Benznidazole is still the most effective trypanocidal drug, which has been systematically confirmed in clinical trials comparing it with new drugs. However, there are critical gaps for the development of new less toxic therapeutic options aimed at improving the safety profile and access to treatment. The conduction of new studies is strategic to assess the use of not only combined therapies, but also shorter therapeutic schedules, with fixed and smaller doses. In addition, better and more reliable clinical parameters and laboratory biomarkers to assess treatment efficacy should be sought.^536^However, studies available so far have not allowed the recommendation of therapeutic schedules different from those classically established. It is worth noting that, in the Brazilian SUS, benznidazole is the most available and used drug, despite its limited operationalization relative to the expected demand.^2 , 54 , 113^

Proper etiological treatment is known to be cost-effective^5 , 50 , 52 , 53^and has the potential benefit of reducing parasitemia, with a positive impact on the patient’s clinical evolution, such as halting progression to the cardiac form, reducing clinical complications in the two phases of disease, increasing life expectancy, and improving physical functioning and quality of life.^2 , 8 , 32 , 41 , 43 , 53 , 318 , 322 , 323 , 537 - 540^

One of the major challenges is the need to make etiological treatment available and implemented in local health systems^2 , 8 , 112 , 113 , 533^and to offer the drug continuously, which is still hindered by the limited number of its providers and its low demand in local health systems.^56^Thus, it is fundamental to avoid missed opportunities to establish the diagnosis and treatment. Considering that CD relates to poverty and social vulnerability, the comprehensive healthcare of individuals with CD will be able to reduce health inequities, particularly in endemic territories.^2 , 8 , 49 , 54 , 113^

This specific chapter on etiological treatment is based on the analysis of consensus, clinical protocols, and therapeutic guidelines, which have been written and recently updated in different contexts. They represent relevant strategies aimed at contributing to increase access to diagnosis and treatment to support clinical decisions.^2 , 41 , 49 , 56^The following referential documents were based on the methodological procedures of the GRADE system,^27^specifically adapted for these guidelines (see chapter related).

One of the regional clinical guidelines analyzed is the 2019 PAHO/WHO Guidelines for the Diagnosis and Treatment of Chagas Disease ( *Guía para el diagnosis y el tratamiento de la enfermedad de Chagas* ).^60^In addition, the 2011 I Latin-American Guideline for the Diagnosis and Treatment of Cardiopathy of Chagas Disease, coordinated by the SBC, was contemplated in the review.^1^

Considering that this is a national guideline and respecting the specificities of the SUS pacts and organization, the major reference for its elaboration was the Clinical Protocol and Therapeutic Guidelines (PCDT) in CD, conducted by the National Commission of Technology Incorporation in the SUS (CONITEC), Secretariat of Science, Technology, and Strategic Supplies of the Brazilian Ministry of Health.^8^

Additionally, the 2015 Second Brazilian Consensus on CD, an important landmark, coordinated by the Health Surveillance Secretariat of the Brazilian Ministry of Health in partnership with the Brazilian Society of Tropical Medicine,^2^was analyzed in the sequence of the 2005 Brazilian Consensus on CD.^3^

### 9.2. Drugs and Administration

Two antiparasite nitroheterocyclic compounds with efficacy established for the etiological treatment of CD are available: benznidazole, a nitroimidazole agent, and nifurtimox, a nitrofuran compound.^54 , 60 , 377 , 534 , 535^Some clinical studies have included other drugs without proven efficacy, such as alopurinol and azole antifungal drugs (by molecule repositioning),^8^which, however, are not included in the scope of this guideline.

Studies in the past 7 years have assessed the efficacy and safety of monotherapy or therapies combining benznidazole and other agents, such as posaconazole or fosravuconazole. Those studies were conducted in *T. cruzi* -infected individuals without evidence of damaged target organs, and their results were limited to only parasitological aspects with long-term qPCR assessment (12 months).^385 , 541 , 542^Despite the disappointing results with the new drugs, the comparative studies reinforced the relevant role of benznidazole in the CD treatment.

A recent review has identified 109 epidemiological studies published after 1997 on the etiological treatment of CD (31 observational and 78 interventional), including 23 116 individuals. The studies were highly heterogeneous regarding not only clinical management for etiological treatment, but also study design and conduction, which limits the evidence available.^543^

The ‘conditional’ grade of recommendation established by PAHO for benznidazole and nifurtimox use, mainly in patients with chronic CD, is justified by the limited certainty level of the body of evidence on the results of efficacy, originating from the scarcity of RCT in this area.^60^In great part, evidence on CD should be presented because it is grounded on *T. cruzi* infection treatment. Once proved the trypanocidal action, in the absence of experimental randomized studies with relevant clinical outcomes, evidence from less robust, observational and good quality studies should be considered.

Moreover, considering the principle of asymmetry, the magnitude of an occasional damage from treatment, if it occurs, is significantly smaller than the benefit associated, particularly with qualified follow-up. Thus, the etiological treatment for CD is justified in a considerable number of cases.

From the perspective of the managers, treatment with benznidazole, thus, can be adopted as a health policy in specific contexts, considering the balance between benefits, risks, and health priorities. Healthcare professionals might have different choices for decision-making, which should always be shared with the individuals affected by the disease. Finally, most individuals affected, when well informed, most likely would want to receive the intervention.

Benznidazole is the first option in the Brazilian context, because of not only the larger experience with its use, but also its profile of adverse events and availability, particularly the pediatric presentations.^2 , 8 , 54 , 113^The use of nifurtimox in Brazil is recommended when benznidazole is not tolerated, as in the occurrence of severe adverse events and in some other particular and specific circumstances.^2 , 8 , 46^

The etiological treatment with any of these drugs should not be routinely and indiscriminately instituted for women of childbearing potential who are not on an effective contraceptive method.^2 , 8 , 60^Similarly, their indication in cases with other severe conditions (liver and kidney failure) should be carefully and individually assessed, according to clinical severity.

Benznidazole is available as tablets of 100mg and 50mg, for adults, and of 12.5 mg and 50 mg, for children. Its absorption occurs in the gastrointestinal tract, while its excretion is predominantly renal, with a mean life of 12 hours.^1^In the Brazilian SUS network, only tablets of 100 mg and 12.5 mg are available.^54 , 544^In 2017, benznidazole was approved by the USA Food and Drug Administration (FDA) for *T. cruzi* infection treatment, which, however, did not ensure full access to the drug in that country.^545^

The Brazilian Ministry of Health acquires the 100mg tablets of benznidazole and delivers them to the State Health Secretariats in response to requirements in the Strategic Supply Information System. The distribution flow to the regional health agencies and/or municipalities is established by each secretariat, integrating actions of pharmaceutical care, epidemiological surveillance, and primary healthcare.^2 , 54^However, the distribution of 12.5mg tablets of benznidazole is centralized in the Ministry of Health, because of the limited registry of pediatric cases in Brazil.^2 , 54^

The process to define the proper dose of benznidazole that ensures efficacy and tolerability has been established through a trial-and-error approach.^546^For adults with chronic CD, benznidazole is administered orally at the dosage of 5mg/kg/day divided into two or three doses, for 60 days, with a recommended maximum dosage of 300mg/day. For individuals with acute CD, the dose can be of as much as 10mg/kg/day. For individuals weighing over 60 kg, the therapeutic schedule can be extended to achieve the ideal target dose, maintaining 300 mg as the daily limit to prevent adverse events.^2 , 8^The 300mg benznidazole regimen can be used for the number of days equivalent to the individual’s weight, limited to a total of 80 days even for individuals weighing over 80 kg.^1 , 2 , 8^This dosage, which seems better tolerated, has been originally proposed by Professor Anis Rassi ( *in memoriam* ) and adopted later in the second half of the research with approximately 1500 individuals enrolled in the BENEFIT study, published in 2015.^324^

For children, the benznidazole dosage can vary from 5 to 10mg/kg/day, divided into two daily doses for 60 days, with a maximum dosage of 300mg/day. If the daily dosage exceeds 300 mg, extension of the treatment duration is recommended so the total calculated dosage for 60 days can be reached.^1 , 2 , 8^The pediatric formulation of 12.5mg soluble tablets can be used, enabling the treatment of newborns and children up to 2 years of age.^2 , 54^The major advantage of 50-mg tablets (not available in Brazil) is the possibility of treating the rest of the pediatric population, including adolescents and young adults.^544^

More recently, the randomized clinical trials CHAGASAZOL,^385^STOP-CHAGAS^542^and E1224,^541^showing no long-term antiparasite effect of posaconazole or fosravuconazole alone, have reported evidence greater than 85% for early parasite clearance (negative PCR) after 2 to 4 weeks of treatment with benznidazole alone or in association with posaconazole or fosravuconazole, an effect sustained during the 12-month follow-up.^385 , 541 , 542^

Later, the BENDITA clinical trial, a phase 2, multicenter, randomized, double-blind, double-dummy clinical trial, conducted in Bolivia, including individuals aged from 18 years to 50 years with the IFCD, has been published.^531^That clinical trial has evidenced that benznidazole induced effective antiparasite response (ranging from 83% to 89%), regardless of treatment duration (2 or 4 weeks), daily dosage (150mg or 300mg), or combination with fosravuconazole, being well tolerated (3% of severe adverse events) by adults with chronic disease.^531^Although not “definitive”, the study suggests the use of benznidazole as standard treatment and emphasizes the need for new studies with shorter drug regimens or with reduced benznidazole dosages.^531^

These findings increase the evidence that new benznidazole regimens could expand access to etiological treatment and ensure greater tolerability.^536^However, stronger evidence for the adoption of short-course therapy lacks. In this context, other ongoing clinical trials are as follows: the BETTY trial – a double-blind, noninferiority, randomized, controlled trial of short-course benznidazole treatment to reduce *T. cruzi* parasite load in women of reproductive age;^547^the MULTIBENZ study – a phase II, randomized, noninferiority, double-blind, multicenter clinical trial assessing the efficacy and safety of different dosages of benznidazole for the treatment of adults with chronic CD;^548^and the TESEO study - an open-label, randomized, prospective, phase-2 clinical trial assessing the safety and efficacy of new therapeutic schedules with benznidazole and nifurtimox for adults with chronic CD, in addition to assessing biomarkers.^549^

Despite the benznidazole’s efficacy demonstrated in several studies, limitations regarding tolerability apply because of its relatively high toxicity, which can lead to the treatment interruption in approximately 10-25% of the cases.^1 , 2 , 5 , 8 , 58 , 60 , 324 , 536 , 550 , 551^The mean incidence of adverse events associated with benznidazole use is approximately 50%, and cutaneous manifestations, gastrointestinal symptoms, and nervous system disorders have been the most common causes of treatment interruption.^2 , 60 , 324^

Cutaneous adverse events are the most frequent, particularly urticarial dermatitis (45%) and rash (30%), and usually do not require treatment interruption because of their low intensity.^8^Dermatitis begins by the end of the first week of treatment, and shows good response to treatment with antihistamines or small doses of oral corticosteroids.^2 , 58^In addition, gastrointestinal intolerance (13%), with nauseas, vomiting and diarrhea, paresthesia (10%), and arthralgias (8%) can be found.^8^Based on Amazonian case series in areas of higher occurrence of CD, the frequency of adverse events due to benznidazole was 20.2% in children and adolescents with CD in the acute phase. In these reports, the cutaneous alterations ( *rash* , urticarial eruption or heterogeneous desquamative exanthema, and angioneurotic edema) were the most commonly found (72%), followed by alopecia (3%), gastrointestinal disorders (2%), and insomnia (2%).^279 , 291^

Peripheral polyneuropathy with paresthesia and pain in the lower limbs is most common in adults and usually begins by the end of the 60 day treatment, particularly after 50 days. It can have a significant impact on functioning and quality of life because it can last several months, even after treatment interruption, and does not respond well to treatment with anti-inflammatory drugs and polyvitamins. The occurrence of fever, adenomegaly, and oropharyngeal pain suggests early bone marrow depression and agranulocytosis, one of the most severe, although rare, effects of benznidazole. In such cases, significant leukopenia develops at the expense of segmented neutrophils (febrile neutropenia), indicating the need for immediate drug interruption and proscription. That is the reason why routine hemogram is indicated 3 weeks after beginning treatment.^1 , 2 , 8 , 58^

Briefly, despite all that, etiological treatment with benznidazole can be safely conducted in the context of PHC. A protocol by the Doctors Without Borders has shown consistent results, because up to 89.8% of the individuals treated could conclude the treatment, although 56.0% developed an adverse event.^58^The success achieved was associated with close monitoring of the cases, which strengthened surveillance, as well as counseling with qualified information and timely identification of adverse events with their management, leading to a lower dropout rate,^58^reinforcing the importance of longitudinal care.^44^

In the context of pharmaceutical care, the protocol of benznidazole dispensation at intervals of approximately 7 days is recommended, which can increase use safety by enabling a closer and qualified follow-up, with timely detection and registry of adverse events.^58^Considering the chronic nature of CD, the pharmacotherapeutic follow-up enables, in addition to etiological treatment, the recognition of events associated with other drugs used, improving adhesion to treatment and quality of life.^5 , 43 , 44 , 536 , 552^

In the case of intolerance to benznidazole, nifurtimox can be recommended. It is available as tablets of 120mg (adults) and 30mg (children).^1 , 2 , 8 , 534 , 535^In 2020, the FDA/USA approved its use for the treatment of CD in children under the age of 18 years,^534 , 535^extending access to treatment based on evidence available.^5^

Nifurtimox has gastrointestinal absorption, hepatic metabolization via cytochrome P450, and preferential renal elimination.^1 , 534 , 535^Nifurtimox is not available in the pharmaceutical market in Brazil, its provision being regulated by a standard protocol of the Health Surveillance Secretariat of the Ministry of Health via PAHO, according to specific demand, usually related to suspicion or confirmation of resistance or intolerance to benznidazole.^2^

In adults, nifurtimox is used at the dosage of 10mg/kg/day orally, in three daily doses for 60 days. In children, the recommended dosage is 15 mg/kg/day orally, also in three daily doses for 60 days.^2 , 534 , 535^The CHICO study, a clinical prospective, controlled trial to assess the efficacy and safety of a new pediatric formulation of nifurtimox for children with CD aged between 0 and 17 years after 1 year of treatment, has confirmed that the 60-day treatment regimen was more effective than the same dosage for 30 days.^553^

With nifurtimox, the mean frequency of adverse events is approximately 85%, the most frequent being gastrointestinal intolerance, such as anorexia and weight loss (60%), rheumatological events, such as arthralgias (35%), and cutaneous manifestations (15%).^8^In the USA, of 243 individuals being treated, 222 (91.4%) reported at least one adverse event (total of 1155 adverse events, median of 4 per patient). The categories of adverse events reported were gastrointestinal (68.7%), neurological (60.5%), and constitutional (46.5%), and the most reported were nausea (50.6%), anorexia (46.1%), weight loss (35.0%), headache (33.3%), and abdominal pain (23.1%). At least 90% of the patients of all age ranges studied (under 18 years, 18-50 years, and over 50 years) reported adverse events.^554^Of 1042 adverse events with available data regarding severity, 680 (65.3%) were mild, 254 (24.4%) were moderate, and 108 (10.4%) were severe. The most frequent severe adverse events were depression (22.6%), peripheral neuropathy (18.5%), paresthesia (17.9%), and dizziness/vertigo (17.2%). The proportion of individuals with at least one severe adverse event was higher among individuals over the age of 50 years (31.8%) as compared to those aged 18-50 years (18.1%).^554^

Based on a comparative analysis of adverse effects, the 2019 PAHO guidelines for the diagnosis and treatment of CD reported no substantial difference between benznidazole and nifurtimox considering the evidence analyzed and expertise of PAHO’s technical panel. However, specific profiles of predominant adverse events were recognized, nifurtimox being associated mainly with weight loss and psychiatric adverse effects, while benznidazole was associated with cutaneous and neurological reactions.^60^The adverse events and toxicity of nifurtimox stand out as reduced digestive tolerance, reflected by anorexia, nauseas, and vomiting, with weight loss and psychiatric disorders most frequent in adults.^1 , 2 , 8 , 534 , 536 , 554^

For both antiparasite drugs, ensuring clinical monitoring of their use is fundamental for the assessment and timely management of adverse events, with emphasis on tolerability.^1 , 2 , 8 , 58 , 535 , 536^ Chart 9.1 summarizes the major adverse effects of benznidazole and the proper management of each situation.

**Chart 9.1:** – Adverse effects of benznidazole and recommended management for each situation.

BENZNIDAZOLE						
Adverse effects	Appearance	Characteristics	Location	Intensity	Management	Additional measures
Dermatopathy due to hypersensitivity is the most frequent. It is NEITHER dose-dependent NOR related to *T. cruzi* infection. Recovery without sequelae	10th day of treatment, but can occur earlier or later	Non bullous erythema polymorphe, pruriginous, followed by desquamation. Onycholysis and angioneurotic edema rarely occur Rare reports of Stevens-Johnson syndrome	Focal (restricted to the tegument) or generalized	Mild (usually focal)	Continue treatment	Specific treatment for dehydration and cutaneous desquamation Hospitalization according to clinical findings
				Moderate (focal or generalized)	Continue treatment and associate corticosteroid (prednisone) at a low dose. Interrupt if worsening occurs	
				Severe, usually accompanied by fever and lymphadenomegaly or Stevens-Johnson syndrome	Interrupt treatment. Use corticosteroid	
Peripheral polyneuropathy. Dose-dependent, of slow regression (months)	End of treatment	Pain and paresthesia	Plantar (more frequent) and palmar regions	Mild to moderate	Interrupt treatment	General treatment for peripheral polyneuropathy
Ageusia (rare). Recovery without sequelae	End of treatment	Total or partial loss of taste	-	-	Interrupt treatment	-
Bone marrow depression and recovery without sequelae	Between the 20th and 30th days of treatment	Fever, adenomegaly, and sore throat can indicate leukopenia with neutropenia of varied degrees, which can reach agranulocytosis	-	-	Interrupt treatment	General treatment for bone marrow depression
Digestive intolerance (rare) is controlled by use of medication for gastritis and peptic ulcers. Severe hepatic impairment is rare. Renal impairment has not been observed.						

Adapted from the 2º Consenso Brasileiro em DC, 2015 (Dias, 2016) ^2^ and Protocolo Clínico e Diretrizes Terapêuticas em DC, 2018 (Brazil, 2018). ^8^

### 9.3. Etiological Treatment of Individuals with Chagas Disease

As already mentioned in another chapter of this guideline, considering the natural history of CD, most individuals with established infection remain asymptomatic throughout life. In the acute phase, 90% of the classical vectorial transmission cases remain asymptomatic or oligosymptomatic, and, of the 10% with evidence of clinical syndrome, less than half progress to more severe forms or death.^2 , 7 , 46 , 555^In oral transmission contexts (outbreaks and familial micro epidemics), in 75% to 100% of the cases, mild clinical syndrome occurs, as in children, or evident illness with prolonged febrile syndrome is observed.^279 , 556^

It is worth noting that the lesions derived from the *T. cruzi* infection in the acute phase depend exclusively on the presence of the parasite, while, in the chronic phase, these lesions are partially explained by the parasite persistence in the tissues and the immune response to the parasite.^1 , 5 , 38 , 46 , 323^

The chronic phase of CD includes the indeterminate form (asymptomatic) and the cardiac, digestive, and cardiodigestive forms.^2 , 46^In the chronic phase, approximately 60-70% of the cases remain asymptomatic, while 30-40% progress to the disease’s clinical forms usually after several years,^32 , 46 , 539^with some potentially severe complications, particularly the cardiovascular ones, associated with high morbidity and mortality.^1 , 2 , 7 , 46 , 297 , 448^Treatment, when indicated in the chronic phase, is aimed at reducing the parasitemia levels, preventing the appearance or progression of lesions in target organs, in addition to preventing transmission.^1 , 2 , 297 , 318 , 322^

The response to etiological treatment, confirmed in parasitological terms, varies and depends on the following factors: age at the time of diagnosis; disease’s phase and duration; complementary tests used to assess therapeutic efficacy; post-treatment follow-up duration; associated conditions; and susceptibility of the different *T. cruzi* lineages (TcI to TcVI) to antiparasite drugs.^38 , 248 , 379 , 538 , 550 , 557 , 558^These aspects reinforce the importance of following all cases up, independently of the place of treatment in the health care network.

Thus, the etiological treatment of an individual with CD should be conducted according to the individual’s characteristics and the disease’s clinical form, as shown in Chart 9.2 .^2 , 8^

**Chart 9.2:** – Recommendations for etiological treatment of Chagas disease according to disease phase or clinical form and age group.

Phase/form of CD	Age group	Etiological treatment
Acute or congenital	All age groups	1^st^line: benznidazole 2^nd^line: nifurtimox
Chronic indeterminate or digestive	Children (≤ 12 years) and adolescents (13-18 years)	1^st^line: benznidazole 2^nd^line: nifurtimox
	Adults < 50 years	1^st^line: benznidazole Do not use nifurtimox
	Adults ≥ 50 years	Shared decision-making: possibility of treatment, if there is no contraindication 1^st^line: benznidazole Do not use nifurtimox
Not advanced chronic cardiac (stage: B1*)	All age groups	Shared decision-making: possibility of treatment, if there is no contraindication 1st line: benznidazole Do not use nifurtimox
Chronic cardiac or digestive (advanced phase)	All age groups	Do not treat

*See Table 5.2 for the cardiopathy stages. Adapted from Protocolo Clínico e Diretrizes Terapêuticas em DC, 2018 (Brazil, 2018). ^8^

### 9.4. Acute Infection

In the acute phase of CD, the grade of recommendation of the etiological treatment for all cases (children, adolescents, and adults) is ‘strong’, even with level of evidence B of moderate quality regarding the benefit of the trypanocidal effect.^60^The treatment should be performed as early as possible after the infection is diagnosed, independently of the *T. cruzi* transmission mode, considering the potential benefits.^1 , 2 , 5 , 8 , 32 , 60 , 555 , 556 , 559 - 562^

In the acute phase, despite the moderate level of scientific evidence and limited certainty regarding clinical outcomes, the treatment has high efficacy, increases the likelihood of serological and/or parasite tests becoming negative, in addition to improving the potentially severe clinical syndrome of the acute phase, thus, preventing progression to the manifest chronic form by reducing damages to specific organs.^8 , 41 , 44 , 291 , 556 , 560 , 562 - 565^Considering the association of nontreated acute CD with mortality in up to 5% of diagnosed cases^559^and the potential progression to the chronic phase in all cases, potential benefits are much superior regarding adverse events, which are mostly mild.^60 , 555^

Thus, even in asymptomatic cases or when diagnostic confirmation is impossible, but suspicion persists (compatible clinical syndrome and epidemiological link, evidence of individuals living with infected individuals or exposed to triatomines, or suspicion of oral or congenital transmission), empirical treatment can be considered.^8^

Therefore, the intervention should be adopted by health managers as a health policy in most situations, given that most healthcare professionals agree with the recommendation of that treatment and that most individuals affected, when informed, want to undergo the intervention.

For pregnant women, at any gestational age, with severe acute clinical syndrome related to myocarditis or meningoencephalitis, antiparasite treatment should be indicated independently of the gestational age, because of high maternal morbidity and mortality.^8^In addition, even with level of evidence C, this indication is justified by the associated high risk (20-70%) of congenital transmission, with potential impact on the health of the neonates, and considering that the rare reports of etiological treatment during pregnancy would be associated with small evidence of malformations.^2 , 57 , 86 , 559^

However, pregnant women in the acute phase of CD without clinical severity should ideally wait for the second gestational trimester to undergo etiological treatment. Despite the potential benefit of reducing neonatal CD, the occasional occurrence of perinatal mortality or fetal malformation is uncertain. Thus, counseling about the risks and benefits of the etiological treatment should be performed, with shared decision, knowing that, in some cases, non-treatment is justifiable.^2 , 8^

### 9.5. Congenital Infection

Similarly to individuals with acute infection, those diagnosed with CD through congenital transmission should receive etiological treatment. In such cases, the grade of recommendation is ‘strong’, independently of the diagnosis being established by use of parasitological methods still in the first weeks, or conventional serological tests, 9 months after birth.^1 - 3 , 5 , 8 , 57 , 60 , 86 , 112 , 553 , 559 , 566^

This strong recommendation, despite the moderate quality of the evidence available (level B) favoring trypanocidal treatment, is based on both the predictable benefits in a potentially severe clinical situation and the higher likelihood of actual cure of the infection.^1 , 2 , 8 , 60^

The etiological treatment of an individual in the chronic phase of suspected congenital transmission should be performed considering the current age, the time of *T. cruzi* infection, and the disease’s clinical stage.^2^These aspects will be detailed in the following sections. It is worth noting that, given the current evidence about CD and the relevance of epidemiological surveillance of chronic cases in Brazil, expanding access to health care is strategic, as is the development of comprehensive healthcare beyond etiological treatment, always keeping in mind the possibility of mother-to-child transmission.

### 9.6. Children and Adolescents with Chronic Infection

For this population, the etiological treatment has grade of recommendation ‘strong’ and level of evidence B.^60^For this management, it is worth emphasizing the potential benefits in a more severe epidemiological context, in addition to the likelihood of influencing, with treatment, outcomes such as turning serology and parasitemia negative.^5 , 8 , 60 , 375 , 376 , 567 , 568^

Antiparasite treatment is indicated to all children (aged 12 years or less) and adolescents (aged 13 to 18 years) diagnosed with the IFCD, considering the higher likelihood of turning serology negative, which indicates proper response to therapy.^1 , 2 , 5 , 8 , 60 , 375 , 376 , 567^This decision is based on significant benefits regarding the reduction of damages to specific organs, without increasing the risk for adverse effects given the better tolerance to antiparasite drugs in those age groups.^60^

In addition, the longer life expectancy of that population justifies that the treatment might be more effective in children as compared to adults.^8^Cohorts with long-term follow-up using conventional serological methods, such as cure control with a mean follow-up of over 10 years for each case, and conducted in the Amazon context have shown the success of the etiological treatment in this population. In these cohorts, the treatment caused minimal complications with potential for chronicity, despite the persistence of reactive serology.^291^

However, evidence relative to prevention of the disease’s clinical manifestations with the use of benznidazole is limited by the short follow-up period of the studies, and that is even more limited for nifurtimox, which should remain as a therapeutic alternative.^2 , 8^In addition, the use of nifurtimox can be considered a valid alternative, particularly in cases involving children, adolescents, and young adults with recent infection in the presence of intolerance to benznidazole.^2 , 8 , 534^

### 9.7. Women of Reproductive Age with Chronic Infection

For women of reproductive age (15 to 49 years) with chronic *T. cruzi* infection, the grade of recommendation for the etiological treatment with benznidazole is ‘strong’, considering the additional strategic benefit of controlling congenital transmission of CD.^2 , 5 , 8 , 60 , 86 , 89 , 112 , 569 - 572^

Antiparasite treatment reduces significantly the likelihood of congenital transmission, and neither fetal nor neonatal adverse events have been observed.^8 , 60 , 86 , 89 , 559 , 569 , 570 , 572^Thus, even with level of evidence B, with moderate certainty regarding the risk/benefit analysis, the grade of recommendation for the treatment was established as ‘strong’.^60^In addition, the effective use of contraceptive methods systematically and correctly should be recommended to these women during the entire trypanocidal treatment period, and pregnancy should be ruled out before beginning treatment.^2 , 8 , 41 , 60 , 89 , 569 , 570 , 572^In endemic areas, these women should be systematically advised and assessed regarding the presence of triatomines, which should be eliminated from the households and their vicinities to prevent reinfection.

For pregnant women with chronic CD, treatment is not recommended given that the risk of congenital transmission is low, around 1.5% to 2% in Brazil.^2 , 46 , 57 , 86 , 559^However, pregnant women with acute and severe CD, expressed as myocarditis or meningoencephalitis, or even in the acute phase of non-severe disease diagnosed in the first trimester should be carefully assessed and the decision regarding etiological treatment should be shared, individualized, as previously discussed.^8^

In the Brazilian Amazonian region, where acute infections predominate, vertical transmission has been reported due to lack of knowledge about the pregnant status in contexts of outbreak or familial microepidemics, with some very good documentation on congenital infection even after beginning maternal treatment with benznidazole.^279 , 556^

Finally, it is worth noting the important international initiative, the ‘CUIDA Chagas’, to which the Brazilian Ministry of Health adhered, involving Bolivia, Colombia, and Paraguay, in addition to five Brazilian states (Bahia, Goiás, Minas Gerais, Pará, and Rio Grande do Sul). The project began in 2022 and includes diagnostic and therapeutic measures and models for the elimination of the vertical transmission of CD among women of reproductive age chronically infected with *T. cruzi* , to be assessed over four years. One of the relevant objectives of this international consortium is to assess, in a randomized controlled study, whether a shorter trypanocidal therapeutic regimen with benznidazole (two weeks) is at least as effective as the usual one (60 days) and whether it has fewer adverse effects.^573^

### 9.8. Adults with Chronic Infection

The potential benefit of etiological treatment for all adults with chronic CD is not supported by a strong recommendation with high level of evidence for that indication, nor generically for any clinical-epidemiological situation.^8 , 60^This recommendation has, thus, a conditional level depending on the case analyzed, given the limited evidence available for some populations.^60^However, in such cases, aspects relative to the principle of asymmetry (if the potential benefit exceeds the risk of adverse effects) should be considered. Thus, this decision should be shared between the physician, health team, individual affected and family, depending on the phase of infection, and patient’s age and clinical conditions.^1 , 2 , 5 , 8^

It is worth noting that, as reported for adolescents, for adults of any age with recently acquired infection, regardless of the transmission mode, the grade of recommendation for treatment is ‘strong’, with level of evidence B.^60^

Considering the stratification defined in the research plan of a significant number of consistent studies to assess etiological treatment, this document established the age cutoff point of 50 years, as in other national and international reference documents.^2 , 5 , 8^It is worth noting that the PAHO clinical practice guidelines published in 2018 did not adopt that age stratification, considering the questions: ‘ *Which is the safest and most effective therapy for adults with chronic T. cruzi infection with/without lesions in specific organs* ?’ For cases in the IFCD, etiological treatment was established as ‘conditional’ with level of evidence ‘weak’, while, for cases with lesions in organs, the treatment was not recommended, with moderate level of evidence. The methodological procedures of the PAHO guidelines were developed from systematic reviews and primary studies published up to August 2017 (PubMed, EMBASE, Cochrane) and manual research with analysis using the GRADE system.^27 , 60^

Since 2017, new studies have been published increasing the body of evidence and demarcating the establishment of the recommendations present in this document, expanding the opportunity of access to treatment of *T. cruzi* infection.

In adults up to 50 years of age with the IFCD, treatment is recommended, considering that its advantages seem to exceed the disadvantages, and that there is more evident benefit regarding cardiac disease prevention.^2 , 8 , 38 , 318 , 320 - 323 , 379 , 574^The recommendation is strong with level of evidence B, considering more recent studies recognizing that the etiological treatment can reduce the risk of long-term cardiac disease,^2 , 60 , 297 , 318 , 321 - 325 , 542^even without clear evidence about the impact on mortality.^8 , 60 , 575 - 577^The probability of negative parasitemia in the short run is higher, while non-reagent serology is observed only in the long run.^38 , 41 , 60 , 318 , 324 , 542 , 557 , 578 , 579^However, the treatment can be associated with considerable risk for adverse events, which are mostly mild and minimized by use of qualified monitoring,^58 , 60 , 318 , 323 , 536^but in some cases are sufficiently severe to cause therapy interruption.

In individuals aged 50 years and older in the chronic phase of CD, the benefit of etiological treatment in the IFCD has an even higher grade of uncertainty, leading to a conditional recommendation for etiological treatment with level of evidence C.^1 , 2 , 5 , 60 , 318 , 322 - 325 , 542 , 579^

As previously shown, the factor ‘age’ should be relativized in terms of etiological treatment and particularly considered for individuals with recent infection (for example, in epidemiological contexts of oral or blood transfusion transmission in which age is an independent factor of clinical evolution) or who were infected during adulthood with no comorbidities and in a clear process of demographic transition in the Brazilian society, with longer life expectancy.^2 , 8^Usually, these perspectives indicate the possibility of conditional recommendation for etiological treatment in this population.

For adults with determined chronic forms in initial non-advanced phases (cardiac and digestive), the indication for etiological treatment should be a shared decision, with information about potential benefits and risks, thus offering the possibility of treatment, being ‘treating with benznidazole’ or ‘not’ valid alternatives, if no contraindication applies. In such cases, the recommendation of etiological treatment is conditional, with level of evidence C.^1 , 2 , 5 , 8 , 60 , 318 , 322 , 323 , 542^The initial phases of the CCCD consist in only ECG changes (ventricular repolarization disorder, VE, RBBB, LAFB, first-degree AVB), with global systolic ventricular function preserved or slightly reduced (LVEF > 40%), and stages B1 and B2 of HF without severe arrhythmias.^1 , 2 , 8 , 318 , 322 , 323^

Etiological treatment can be considered independently of the diagnosis of isolated or associated chronic digestive form, that is, cardiodigestive disease,^8^because the treatment is aimed at preventing cardiac damage. For patients with digestive disorders installed and even in those without the digestive form, there is no evidence that the antiparasite treatment prevents or delays the appearance or progression of megaesophagus and megacolon.^8 , 559^Some patients with megaesophagus can have the efficacy of treatment with benznidazole hindered by interference with its ingestion or absorption.^2 , 8^Although the diagnosis of chronic digestive form represents no contraindication to etiological treatment, clinical rehabilitation, dilation or surgical correction of megaesophagus is recommended before initiating the etiological treatment to ensure drug transit and absorption in the digestive tract.^2 , 8 , 44^

When chronic cardiomyopathy is already installed, usually there is no evidence that the etiological treatment might significantly impact evolution to death or progression of cardiac disease, although it increases the probability of eliminating parasitemia, assessed by PCR.^60 , 323 - 325^Thus, antiparasite treatment should not be recommended for individuals with advanced organ lesions (stages C and D of cardiac forms) or very old.^1 , 2 , 5 , 8 , 32 , 60 , 318 , 322 , 324 , 540 , 579^In such cases, the etiological treatment does not change the disease’s natural history, can be associated with increased risk for severe adverse events, in addition to inducing direct and indirect costs for the individuals affected and their families, thus expanding their social vulnerability.

So, all efforts should be made for timely diagnosis and etiological treatment in cases of CD to prevent disease progression. It should be noted that the annual risk of mortality in CCCD is considerable (7.9%; 95% CI, 6.3-10.1%) and mainly attributed to cardiovascular disorders, especially in the presence of low LVEF and classified as stages C and C/D.^448^

Individuals with severe megaesophagus, which hinders the proper absorption of the trypanocidal agent, are in a special situation. In the absence of manifest cardiopathy or in its initial stage, in which the etiological treatment is aimed at preventing cardiovascular disease progression, etiological treatment can be indicated after surgery for megaesophagus.^2^In such situation, the indication for etiological treatment would have ‘conditional’ recommendation and level of evidence C.

Notably the careful analysis of the results from the BENEFIT trial, the most comprehensive RCT on trypanocidal therapy in patients with CCCD (mostly nonadvanced), enabled the identification of some relevant aspects. In fact, when considering the entire population, involving patients from five Latin American countries (Brazil, Argentina, Colombia, Bolivia, and El Salvador), the etiological treatment with benznidazole had no favorable impact on mortality and other severe outcomes from cardiomyopathy.^324^In addition, when compared to placebo, there was no benefit regarding regional ventricular dysfunction, an early alteration often detected in those individuals and with a poor prognosis.^342^

However, the global analysis of that study’s results has been criticized, which might have prevented the duly appreciation of some methodological flaws with relevant implications in the applicability of the study’s results.^325^For example, as compared to the group treated with placebo, the group treated with benznidazole showed a statistically significant reduction in the rate of hospitalization due to cardiovascular causes, which, although highly valued in several studies involving patients with HF, was not even discussed in the primary analysis of the BENEFIT trial.^324^

There are other aspects that deserve critical appreciation. The initial analysis of the BENEFIT trial subgroups was arbitrary, non-prespecified, and did not follow defensible criteria, being, therefore, biased.^324 , 325^In contrast, *post-hoc* analysis of that study’s results evidenced that the etiological treatment effect on Brazilians patients (40% of that study’s sample) might have been positive, particularly when comparing the results in the Brazilian subgroup with those observed in the other four countries participating in BENEFIT.^325^

In the meantime, this possibility should be considered the generator of a hypothesis to be tested in a subsequent study specifically designed to prove or refute it. The corollary hypothesis of the interpretation that the etiological treatment is more effective when applied to Brazilians already with CCCD is biologically plausible and can be supported by the predominance of TcII parasite genotype observed in Brazil, which can be more sensitive to benznidazole as compared to other *T. cruzi* strains and nifurtimox. There are scientific reasons why the treatment of patients based on trypanocidal drugs (including those in the validation phase) should consider both the parasite genetic diversity^248^and the complex interaction of several parasite lineages with the human host, resulting in several clinical expression forms.^250^

Based on these considerations, the ‘conditional’ grade of recommendation for offering etiological treatment to individuals already with non-advanced CCCD in Brazil can be considered with emphasis on the higher potential benefit than that in other countries. Finally, it should be pointed out the significant severity of CD and the need for timely diagnosis and comprehensive care to individuals with cardiopathy, based on qualified clinical management.^1 , 2 , 44 , 60 , 324^

In addition, considering current evidence about etiological treatment as well as relevance of epidemiological surveillance, mandatory reporting of cases of chronic CD should be implemented, enabling the extension of access to diagnosis and treatment to more individuals affected.^8 , 44 , 56 , 91 , 94 , 113^

### 9.9. Reactivation of Chagas Disease

The RCD consists in the aggravation of *T. cruzi* chronic infection, characterized by an increase in parasitemia (similar to that of the disease’s acute phase) and immune system’s inability to control the infection, usually associated with drug-induced immunosuppression – transplantations, immunosuppressive treatments – or coinfection with HIV.^1 , 2 , 8 , 83 , 84 , 580^

The RCD is associated with high morbidity and mortality because of the central nervous system infection and myocarditis and has a critical impact on quality of life.^2 , 8 , 83 , 84^The prevalence of RCD based on parasitemia in immunosuppressed individuals with CD, without trypanocidal prophylaxis, was approximately 28%, and distributed as follows: 1.8% in liver transplanted individuals; 23.3% in bone marrow transplanted; 27.3% in kidney transplanted; 30.9% in heart transplanted; and 39.6% in individuals with HIV infection/AIDS.^60^

If RCD occurs, the etiological treatment indicated for the acute phase of CD should be initiated.^2 , 8 , 83 , 84^Despite the moderate level of evidence (B), the recommendation is classified as strong, because the antiparasite drugs can have benefits in preventing the consequences of reactivation, as well as in its control and even recurrence.^5 , 60 , 84 , 580 - 584^

In HIV infection, in the presence of chronic CD without reactivation and no previous etiological treatment, patients should preferably be treated with benznidazole and have their immune *status* assessed because of the increased risk for immune reconstitution inflammatory syndrome.^8 , 83 , 84^

For transplanted patients with RCD, the same treatment schedule used for non-transplanted ones is indicated, benznidazole being the preferred alternative due to its better profile regarding adverse events and the largest experience with its use in the country.^2 , 8^There is no consistent evidence to recommend secondary prophylaxis for transplanted individuals, but that can be indicated in selected cases, particularly the most immunosuppressed ones.^8 , 60^

Usually, the etiological treatment can contribute to prevent clinical complications, such as cardiopathy, and should be considered with the same recommendations and levels of evidence used in other situations related to non- immunosuppressed individuals with chronic CD.^8 , 84^

For both HIV-infected and transplanted individuals, qPCR can aid in clinical monitoring; however, its routine recommendation is yet to be defined.^8 , 60 , 580^It is worth noting that the RCD episodes can occur repeatedly and should be treated when documented, justifying regular parasitological monitoring while the immunosuppression condition is maintained.^2 , 83 , 84^

### 9.10. Accidental Infection

In laboratory accidents with biological material contaminated with *T. cruzi* and at high risk for disease transmission, such as sharp instruments, contact with non-intact mucosa or skin, or manipulation of biological material with live parasites ( *T. cruzi* culture samples, biological samples of cases with high parasitemia, and infected material from autopsy, vectors, and experimental animals), primary prophylaxis is indicated, beginning with benznidazole at the dosage of 7 to 10 mg/kg immediately after the accident and for 10 days.^1 , 2 , 558 , 585^The grade of recommendation for this management is ‘strong’, despite the limited level of evidence (C), but considering the principle of asymmetry.^2 , 5 , 8 , 60 , 83^

Serological tests should be performed before initiating treatment and on the 20th, 40th, and 60th days after treatment for monitoring an occasional seroconversion.^2^If the serological tests are reagent, the conventional antiparasite treatment should be performed as previously described for the acute phase. In situations of minimum risk, such as the mere superficial contact with blood of individuals in the chronic phase of CD, drug prophylaxis is not indicated and serological tests are recommended immediately after and on the 20^th^, 40^th^, and 60th days after the accident.^2^If seroconversion occurs, the conventional treatment for the acute phase of CD should be initiated with post-therapeutic monitoring as recommended for the acute phase. If the serology remains positive after treatment, a possible therapeutic failure should be documented for a new treatment with benznidazole or nifurtimox.^1 , 2 , 83 , 534^

### 9.11. Assessment of Chagas Disease Cure After Etiological Treatment

In a disease with only two therapeutic options with consistent indications for use, there is no evidence available about complementary methods to assess, in the routine context of health services, the etiological treatment effect on parasite elimination, particularly in the chronic phase.^1 , 8 , 41 , 60^Ensuring access to treatment is fundamental, which has a clear social function given the negligence regarding individuals affected by the disease. Usually, arguments associated with adverse events and not being able to document cure are used to justify non-treatment in the SUS. As a chronic condition, CD requires comprehensive and longitudinal care to all individuals affected.

There is no complementary method to confirm advancement for cure (which would be considered gold-standard), making serological and molecular tests, even with all technical limitations, potentially available and useful methods to assess response to antiparasite treatment in the chronic phase.^8 , 38 , 60 , 379^

Thus, there is no evidence regarding the need for follow-up with serological control after a complete treatment or retreatment course.^8 , 60^The quality of the evidence supporting the use of negative serology to replace clinically relevant outcomes is ‘low’ or ‘very low’, actually representing an indirect outcome.^8 , 60^

In addition, in adults, it may take more than two decades for a serological test to become negative after treatment,^38 , 379 , 557^which usually occurs in only 1/3 of the cases, depending on different factors, such as age at the time of treatment, time elapsed between treatment and follow-up, and the area in which the infection occurred.^586^For children and adolescents, a serological test can become negative within 5 years in 3/4 of the cases.^376 , 553 , 556 , 560 - 567^Analyses of children and adolescents with acute CD in the Amazon region indicate persistent reactive serological tests in almost 55% of the cases, in a mean follow-up of approximately 11 years after treatment, and 17% of the cases showed sustained negative serological responses.^291^

Although some studies have suggested the use of PCR to monitor and control therapeutic response, the sensitivity of PCR is variable^587^and it is not available as a validated and agreed technique in the SUS, restricting its applicability to research activities.^8 , 46^However, a positive PCR in the first 24 months after treatment indicates the possibility of therapeutic failure.^562 , 587^

The percentages of cure reported by several studies after antiparasite treatment of CD differ, but the importance of the etiological treatment is acknowledged in both the acute phase and some clinical forms of chronic disease.^2 , 8 , 557^

In addition, even with all the already mentioned limitations of the current antiparasite therapy, elimination of parasitemia can be achieved in some scenarios,^1 , 2 , 8 , 46 , 320 , 324 , 540 , 574 , 579^making the usefulness of etiological treatment unquestionable in a considerable part of the clinical situations, independently of the demonstration of cure, except for acute CD. Thus, for the chronic phase of CD, defining the criterion for cure has no practical use, contributing greatly as a strong barrier to access.

#### 9.11.1. Where to Treat an Individual with Chagas Disease

In addition to the technical-scientific leadership in CD management, Brazil has a great differential as compared to most CD endemic countries: the SUS, the Brazilian public health system that provides comprehensive and democratic health care, a right ensured to Brazilian citizens by the 1988 Federal Constitution. Thus, the SUS provides access to diagnosis and treatment for CD in the country.^2 , 49 , 113^

However, despite the favorable context and referential available from ordinances, guidelines, consensus, and PCDT itself,^1 - 3 , 8 , 60^with clinical benefits in the short and long run, diagnosis, treatment, and surveillance of chronic CD could not be consistently implemented in the Brazilian territory.^113^Questions such as centralization of actions, surveillance and control of CD have contributed to this situation. Thus, a global unified overview of the current development stage of initiatives to control CD in Brazil, despite the achievements over almost 120 years, indicates the need for implementation and integration of the measures in the PCDT with sustained surveillance of CD and adhesion to national and international guidelines.^113^

Particularities of the SUS healthcare network should be considered, recognizing, however, that etiological treatment of the *T. cruzi* infection is feasible, safe, and operationally practicable in the PHC.^2 , 8 , 44 , 58 , 94 , 113 , 529 , 533 , 536^The PHC can assume the management of individuals with non-severe acute CD, the IFCD, or even the chronic forms (cardiac, digestive, or cardiodigestive) of stable non-severe disease, as well as of pregnant women with chronic CD without comorbidities.^8 , 44^There is evidence that family doctors and their teams, knowing the particularities of the medications and disease, can clinically manage those cases.^2 , 44 , 58 , 533^

Depending on the severity of the clinical conditions, mainly in the acute phase or RCD, as well as decompensated chronic forms, support might be needed for the plan of care or referral to more specialized healthcare units, or even hospitalization in sporadic conditions.^2 , 8 , 44 , 94^

Table 9.1 summarizes the recommendations for etiological treatment of *T. cruzi* infection in different contexts of CD, according to the strength of recommendation and level of evidence, based on the GRADE system.

**Table 9.1:** – Recommendation for etiological treatment in different contexts of Chagas disease, according to the grade of recommendation and level of evidence (adapted from the GRADE system)

ETIOLOGICAL TREATMENT OF CHAGAS DISEASE *Trypanosoma cruzi* infection	Recommendation grade	Level of evidence
Children with acute infection	Strong	B
Children with congenital infection	Strong	B
Adolescents and adults in general with acute or recently acquired infection	Strong	B
Children and adolescents with chronic infection	Strong	B
Women of reproductive age with chronic infection	Strong	B
Individuals in general with reactivation of chronic infection (HIV/AIDS or other immunosuppressive conditions, including transplantation)	Strong	B
Individuals in general with accidental infection with biological material in laboratory or healthcare settings	Strong	C
Pregnant women with severe acute clinical syndrome - myocarditis or meningoencephalitis	Strong	C
Adults aged < 50 years with chronic infection (chronic indeterminate form)	Strong	B
Adults aged ≥ 50 years with chronic infection (chronic indeterminate form)	Conditional	C
Adults in general with chronic infection (initial phases of chronic determined forms – non-advanced cardiac and digestive)	Conditional	C
Individuals in general with chronic infection and advanced organ injury in the digestive form (not associated with advanced cardiac disease), after surgical repair	Conditional	C
Individuals in general with chronic infection and advanced organ injury in the chronic cardiac or digestive form (associated with advanced cardiac disease) should not be treated	Strong	C

When this chapter was being concluded, the update of an old systematic review and respective meta-analysis was published regarding studies on the etiological treatment with benznidazole for individuals with *T. cruzi* infection.^588^The essential conclusions of that publication are in accordance with the recommendations presented in this guideline. Finally, in addition to the search for more solid scientific evidence, all efforts should be made to ensure access to diagnosis and etiological treatment of CD in the national health systems.

## 10. Therapeutic Management of Ventricular Dysfunction and Heart Failure

### 10.1. Pharmacological Resources

#### 10.1.1. Heart Failure Classification

Our recommendations prioritize patients with reduced LVEF, because most pharmacological therapies have been validated in that scenario. The difference between the scientific study inclusion criteria and the clinical indication should be understood. Studies usually select patients with lower LVEF (< 35% or < 40%) to optimize the incidence of the outcome of interest, increasing the statistical power. Because the magnitude of the absolute effect (NNT) is more relevant in high-risk patients (that is, we should treat few patients to obtain a benefit) and no plausible reason is identified for the occurrence of qualitative interaction (disappearance of effect) when a certain LVEF cutoff point is exceeded, we chose to generalize our recommendations for the use of the essential drugs to treat HF in patients with LVEF < 55%, preventing excessive categorization.

However, it is worth considering the existence of a *continuum* of inverse relation between LVEF and therapeutic benefit, that is, the lower the LVEF value, the higher the absolute benefit of the therapy proposed. To simplify, strong recommendations for LVEF ≤ 40% will be ‘conditional’ for LVEF between 41% and 54%. In addition, we consider that in patients with segmental contractility abnormalites, but without global ventricular dysfunction, who are classified as stage B of HF, the body of evidence is still insufficient to promote any recommendation.

#### 10.1.2. Maximum Dosage of Medications

This guideline does not support the obstinacy in reaching the maximum dosage of medications to the detriment of polypharmacy, preferring to emphasize the individualization of each drug best dosage for each patient. The *rationale* for that is based on some justifications. The dosage proposed or even reached for patients in clinical trials is part of a scientific strategy, aimed to generate contrast between groups and test conceptual hypotheses. Once the concept has been demonstrated, it should be applied in an individualized way, weighting benefits and risks. Thus, choosing a drug dosage relates more to clinical reasoning than to evidence. There are no convincing scientific data about the magnitude of the incremental benefit related to maximum dosage ( *versus* weighted dosage) and whether it overcomes unintentional consequences. In addition, tolerability and adverse effects are underestimated in RCTs of efficacy, in which ideal candidates for the treatment in question are usually selected and care is better controlled. Thus, we do not transform efficacy into effectiveness by standardizing the maximum. The increase in effectiveness results from careful individualization.

#### 10.1.3. The Contemporary Patient

As the life of patients with CCCD and HF is prolonged, they tend to suffer from other diseases that appear with aging.

Recently, a RCT of patients with CCCD from a single center (FIOCRUZ) has reported mean age of 65 years, mean body mass index of 27.4 kg/m^2^, and SAH in 1/3 of the patients,^589^differing, thus, from studies showing younger individuals frequently without comorbidities. Also, there is a possibility of a different clinical course of HF from CD as compared to HF from ischemic and idiopathic dilated etiologies.^590^That might be due to the more severe autonomic dysfunction, higher density of ventricular arrhythmia and intracardiac blocks, greater load of myocardial fibrosis, most frequent RV impairment, and higher degree of cardiac sphericity/remodeling and myocardial inflammation – factors that could interfere with response to standard pharmacological treatment.^591 , 592^

The worst clinical course, merely from the statistical viewpoint, suggests greater absolute benefit from treatment with level B of evidence when compared to the target populations of the studies, and should not implicate in the violation of the principle of indirect evidence, that is, extrapolation.

The previously cited study from FIOCRUZ^589^is part of a comprehensive initiative of translational research aimed at experimentally and clinically exploring hypotheses about the potential benefit of supplementation with nutrients, such as selenium, and antagonism to inflammatory factors to change the CCCD course.^593^The primordial value of these incipient investigations might reside in their strongly pathophysiology-based hypothesis on the inflammatory nature of CCCD. Only guided research will be able to provide the responses to those interventions.

#### 10.1.4. Literature Review

For each drug or class of drug used in the treatment of HF, a systematic review of the literature up to August 22, 2021, has been conducted, aiming to answer the following PICO question of EBM: “Are these drugs effective to relieve symptoms and/or reduce mortality of symptomatic patients with systolic HF secondary to CCCD, with a safety profile similar to that for HF of other etiologies?”. The following *Medical Subject Headings* (MESH) were used: “ *beta-blockers, spironolactone, sacubitril-valsartan, ivabradine, sodium-glucose transporter 2 inhibitors* ”, “ *heart failure* ” or “ *Chagas disease* ”, with limit of the type of publication (“ *clinical trial* ”). The MedLine/PubMed, Lilacs, Web of Science and EMBASE databases were used as search sources.

#### 10.1.5. Pharmacological Therapy

10.1.5.1. Diuretics

The diuretic therapy in HF is misunderstood in its magnitude of effect. The lack of RCTs comparing diuretic *versus* placebo can cause the mistaken impression that, differently from beta-blockers or ACEIs, loop diuretics do not reduce mortality. This lack of studies could be due to the absence of *equipoise* for the type of patient whose prognostic benefit was validated for other therapies. That is, in HF, the administration of a diuretic is a therapy of extreme plausibility, corresponding to the parachute paradigm,^31^justifying, in this guideline, level of evidence C regarding pharmacological indication. Thus, we strongly recommend diuretic therapy for HF with moderate to severe ejection fraction reduction and for cases with mild ejection fraction reduction, in the presence of pulmonary or systemic congestion.

10.1.5.2. Renin-Angiotensin-Aldosterone System Inhibitors

Several clinical trials of quality have shown that, in patients with HF and reduced LVEF, several ACEI reduce relevant outcomes of morbidity and mortality.^594 - 596^In addition, these drugs can be replaced by angiotensin II receptor blockers (ARBs) in case of poor tolerability.^597^However, in HF of CCCD, there is no direct evidence of benefit from RCT performed specifically in that population. Thus, the evidence regarding the use of ACEI in CCCD is indirect, originated from studies of excellent quality that tested the efficacy of that treatment in the most common types of cardiomyopathy (ischemic and idiopathic dilated) (level B). Considering that ejection fraction is a prognostic *continuum* , rather than a binary, dichotomic variable, the higher the degree of ventricular dysfunction, the higher the absolute benefit. Thus, the recommendation is strong for patients with HF and LVEF ≤ 40%, while ‘conditional’ for patients with HF with mildly reduced ejection fraction (HFmrEF).

Studies with a very reduced number of patients, assessing captopril and enalapril in HF of CCCD, have evidenced a reduction in the sympathetic neuro-humoral activation and in serum angiotensin levels, in addition to improvement of diastolic dysfunction and ventricular remodeling.^598 - 600^These patients frequently have reduced systolic blood pressure, and can become more hypotensive and symptomatic with the introduction of ACEI or ARB, which should be gradually up-titrated, aiming to reduce the dosages of diuretics when the patient no longer has edema.

In recent decades, international guidelines have emphasized the search for the therapeutic target dosage of ACEI or ARB for patients with HF with reduced ejection fraction (HFrEF), which can be misleading and a limitation in clinical practice, considering that patients with CCCD are prone to symptomatic arterial hypotension. Thus, we should search for the best tolerated dosage and proceed to slow titration in that group of patients subject to dosage limitations.^601^

10.1.5.3. Beta-blockers

The first experiences using beta-blockers for the treatment of patients with HF date back to the 1970s, when some researchers assessed the effect of the drug on seven patients with cardiomyopathy, advanced HF, and tachycardia.^602^On that occasion, one patient received alprenolol, 50 mg twice a day, and the others, practolol at dosages ranging from 50 mg to 400 mg, twice a day. The authors reported clinical improvement, cardiomegaly reduction, and ventricular function improvement assessed on phonocardiogram, ECHO, apexcardiogram, and carotid pulse curve. Despite the promising results reported by the Swedish group, the use of beta-blockers in HF was only properly assessed in the 1990s.

The seminal study suggesting benefit from beta-blockers in HFrEF was the *U.S. Carvedilol Heart Failure Study* ,^603^which randomized 1094 patients for carvedilol or placebo and showed a reduction in mortality.

In the past 25 years of clinical investigation, beta-blockers were consolidated for the treatment of HF. In a meta-analysis^604^involving 10 clinical trials and 18 254 patients with HFrEF, beta-blockers reduced global mortality by 27%.

Importantly, patients with HFrEF can get worse in the initial phase of the beta-blocker treatment.^21^Thus, surveillance is mandatory regarding worsening, appearance of bradycardia, cardiac block, and hypotension, especially in the first weeks of treatment adjustment.

In the context of HF, that is especially important, because patients with CCCD are more susceptible to the occurrence of those adverse manifestations when on beta-blockers. Although CCCD has not been included in large multicenter studies investigating beta-blockers and mortality, in addition to the peculiarities of that syndrome, known to be associated with dysregulation of the autonomic nervous system, as reviewed in another chapter of this guideline, there is no biological plausibility in questioning the benefit of beta-adrenergic block in the treatment of HFrEF from CCCD.

Analysis of a small group of patients (n = 68) with HF due to CD from the REMADHE trial^605^has compared those on beta-blockers with those not using those drugs. Despite the limitation of the small sample size for direct comparison, the authors have suggested beneficial effects of beta-blockers related to increased survival (non-adjusted p = 0.05, that is, borderline). It is worth noting that, in that study, the use of beta-blocker was not randomized, with a high risk of confounding bias due to indication.

We consider that the evidence regarding the use of beta-blockers for patients with HFrEF due to CD is indirect, originating from studies of excellent quality that tested the efficacy of that treatment in the most common types of cardiomyopathy (level B). Considering that ejection fraction is a prognostic *continuum* , rather than a dichotomic variable, more severe systolic dysfunction tends to be associated with a higher absolute benefit. Thus, the recommendation is strong for patients with HF and LVEF ≤ 40% and conditional for patients with HFmrEF.

Severe ventricular arrhythmia requiring amiodarone is a special case. The association of a beta-blocker with amiodarone can be inappropriate because of bradycardia and/or QT interval prolongation. We consider that, in such context, there is no proof that a beta-blocker should be the priority. This is a case to base decision-making on clinical judgement, and the physician should decide which drug to prescribe initially based on the arrhythmia severity (favors amiodarone) *versus* the HF severity (favors a beta-blocker). This is a rare situation in which this guideline recognizes the limitation of static recommendations, being open to the dynamism of medical thinking grounded in rationality and evidence (not to be mistaken with concepts of efficacy allegedly based, in a naïve and inconsequential way, only on the infamous “clinical eye”).

10.1.5.4. Spironolactone

Spironolactone is the preferable antagonist of mineralocorticoid receptor, the major binding site of aldosterone and responsible for its physiological actions, with direct involvement in the pathophysiology of HF.

In general, spironolactone is indicated for all patients with symptomatic HF and LVEF ≤ 35%, regardless of the concomitant use of ACEI, ARB, or beta-blockers, except for patients with serum creatinine > 2.5mg/dL or creatinine clearance < 30mL/min/1.73m^2^, or serum potassium level > 5.0mEq/L.

The RALES study, a randomized, double-blind, placebo-controlled study supporting that indication, was published in 1999. That study assessed if the use of spironolactone, at dosages ranging from 25 mg to 50 mg, would be better than placebo for HFrEF (≤ 35%) and functional class III-IV, with the concomitant use of ACEI and furosemide.^606^The study was early terminated after 24 months, with a satisfactory number of outcomes to indicate precision and interim analysis showing 35% reduction in the risk of death.

Patients with creatinine > 2.5 mg/dL were excluded, and the incidence of hyperkalemia was minimum in both groups. This should be highlighted because the Canadian study of epidemiological surveillance reported that the spironolactone prescription rate increased substantially after the publication of the RALES study, followed by an increase in the morbidity and mortality rates associated with hyperkalemia.^607^Thus, observation of the contraindication criteria for spironolactone use and judicious surveillance are essential in the clinical management of patients on that drug.

Despite the minimum representation of CCD in the RALES study (and this was not specified in its baseline table), there is no biological plausibility to question the potential benefit of aldosterone block regarding HFrEF progression in CCD. Thus, we consider it a good application of level of evidence B (indirect, good quality). Considering indications and contraindications, the recommendation should be strong for patients with symptomatic CCCD, LVEF ≤ 40%, creatinine ≤ 2.5mg/dL, and serum potassium ≤ 5.0mEq/dL, while conditional for patients with HFmrEF.

10.1.5.5. Ivabradine

Ivabradine is a selective funny channels current blocker and, thus, an inhibitor of the PM activity in the sinus node, resulting in selective reduction of HR without changing hemodynamic parameters, such as blood pressure or myocardial contractility, and without interfering in intracardiac electrical conduction.

The SHIFT trial, a randomized, double-blind, placebo-controlled clinical study, published in 2010, supports the use of ivabradine for HF.^608^In that study, ivabradine was tested at the maximum dosage of 7.5 mg, twice a day, in patients with HF (LVEF ≤ 35%), sinus rhythm, and HR > 70bpm, regardless of the use of beta-blockers when tolerated. The study reported a 26% relative reduction in the risk of hospitalization, in addition to mortality from HF of 26%.

A substudy of the SHIFT trial,^215^by use of *post-hoc* analysis, has assessed 38 patients with HF due to CD, assigned to two groups as follows: ivabradine group, 20 patients; placebo group, 18 patients. Although patients with CCCD usually have a worse prognosis, with higher prevalence of RBBB, lower blood pressure levels, higher rate of use of diuretics, spironolactone, digoxin, and lower rate of use of ACEI/ARB and beta-blockers, when compared to the general population of the SHIFT study, ivabradine did not associate with higher prevalence of severe bradycardia, AVB, hypotension, or syncope. In addition, ivabradine was effective in reducing the HR of those patients and improving the HF functional class.

A therapeutic recommendation should not be based on exploratory study. In the SHIFT trial, the CCCD was not well represented. However, generalization does not depend only on representativeness, and we recognize no probable mechanism of interaction that raises the suspicion that the CD etiology changes the effect of therapy with ivabradine to the point of losing efficacy as shown in the general set of patients included in the SHIFT trial. Thus, we defined the level of evidence B, which represents indirect evidence of good quality for the use of ivabradine in patients with CCCD and HF. The strength of the recommendation should be ‘conditional’ because it depends on the perception that HR is high when the beta-blocker dosage cannot be increased. Given this specificity, we chose not to extend that indication to patients with LVEF > 40%.

10.1.5.6. Digoxin

When reviewing the literature, we identified no study assessing the safety and efficacy of digoxin in CD. Thus, we will use indirect scientific evidence that digoxin improves symptoms and reduces hospitalizations.^609 , 610^In clinical practice, the drug can be indicated for patients in NYHA functional class III and IV, despite optimized treatment with other drugs, especially in the presence of AF with high ventricular response.

Regarding digitalis, the therapeutic dosage is very close to the toxic one, which increases the potential for adverse effects, because of the conduction system involvement, causing bradyarrhythmias, AVB, and other general clinical manifestations.

10.1.5.7. Sacubitril-valsartan

Sacubitril-valsartan is a drug combination of a neprilysin inhibitor (increases the availability of atrial natriuretic peptides), sacubitril, and a traditional angiotensin II receptor blocker, valsartan. The PARADIGM-HF trial was the major study for scientific validation of this drug combination^611^as compared to enalapril. Although it can be considered a precise study with a low risk of bias, showing a 20% relative risk reduction with that drug association in the primary combined outcome of hospitalization from HF and cardiovascular death, there was margin for scientific questioning of its conceptual design.^612^With a heterodox comparator, the study was not able to differentiate whether the benefit found was due to the innovative molecule in the treatment (sacubitril) or whether it was due to the inadequate difference regarding the dosages of the traditional angiotensin system inhibitors (valsartan, at the maximum daily dosage of 320 mg *versus* enalapril at the submaximal, maybe insufficient, daily dosage of 20 mg). Another limitation was the presence of a *run-in* phase in a phase III study, which overestimates the applicability of the treatment, because it selected in advance the patients who tolerate a more intense vasodilating therapy.

From the PARADIGM-HF trial publication, many cardiologists began to perceive the sacubitril-valsartan combination as more effective than the traditional vasodilation with ACEI, which has influenced recommendations of guidelines on HF. In Brazil, the sacubitril-valsartan use was approved in May 2017 by the Brazilian Health Surveillance Agency (ANVISA), and, in August 2019, it was incorporated into the SUS.^613^

In addition, unlike the homogeneous benefit verified with several angiotensin-II system inhibitors studied, the sacubitril-valsartan combination did not prove superiority in other contexts. Two examples of that are the PARAGON-HF study,^614^of patients with HF and LVEF ≥ 45%, and the PARADISE-MI study, of HF complicating acute myocardial infarction.^615^In both scenarios, the results could not reject the null hypothesis in their primary analyses. It is worth noting that the PARADISE-MI study was the only to compare sacubitril-valsartan with the proper dosage of ACEI (10 mg/day of ramipril).

Thus, we consider inappropriate an indication based on the expectation that this drug combination is superior to the traditional therapy. On the other hand, there is no evidence that this therapy is harmful, making it a valid therapeutic alternative, if the doctor wants to modify a standard treatment due to clinical or logistic reason. It is worth noting that the report supporting the incorporation of sacubitril-valsartan into SUS has estimated an incremental cost-effectiveness ratio of R$22.769 per year of life gained with quality.^613^

Regarding the indication of that combination for patients with HF caused by CCCD, in addition to the literature review technique already mentioned, Google Scholar was used to search the gray literature for any reference that could clarify this issue, and annals of medical congresses were assessed for this information.

Thus, a case series conducted with patients with CCCD treated with sacubitril-valsartan in a reference hospital for that disease in Brazil has reported, after 6 months, symptomatic improvement of the individuals.^616^

In a prospective observational study of 136 consecutive patients with HF in a single university-affiliated hospital center, including the HF etiologies of ICM, CCCD, and idiopathic cardiomyopathy,^617^the authors reported that up to 44% of the patients had the major criteria of exclusion of the PARADIGM-HF trial. In addition, they reported that the lower blood pressure levels, common in CCCD, could lead to underuse of some drugs in this context.

Another study, assessing the proportion of patients with CCCD randomized to two recent clinical trials (PARADIGM-HF and ATMOSPHERE), has found that only 7.6% of the randomized patients in Latin America had that etiology.^592^

A *post-hoc* analysis of a subgroup of the PARADIGM-HF trial has suggested that sacubitril-valsartan, as compared to enalapril, could lead to a similar or even higher (37%) reduction in death and hospitalization of patients with CCCD, as compared to those with HF of other etiologies, despite lack of statistical significance and imprecision of estimates of effect.^618^The ongoing PARACHUTE trial (ClinicalTrials.gov Identifier: NCT04023227) is an exclusive study of patients with HF from CCCD. Similarly to the PARADIGM trial,^611^the comparators are not the orthodox ones and the effect of sacubitril associated with the maximized dosage of valsartan will be compared to that of enalapril at a non-maximum dosage, of 20 mg daily.

In summary, patients with CCCD have not been well represented in the sacubitril-valsartan studies. Thus, although we presented some evidence on the use of that therapy for patients with HF caused by CCCD, it is not enough to support a recommendation, due to its exploratory character. However, the evidence serves to exemplify the principle of indirect evidence: generalization does not depend only on representativeness, and we do not recognize any probable mechanism of interaction that makes us suspect that the etiology of the heart disease modifies the effect of this therapy. Therefore, we defined that the sacubitril-valsartan treatment for patients with HFrEF and CCCD is an alternative with level of evidence B, although not a superior innovation as compared to the traditional treatment. Regarding recommendation, this should not be the preferred treatment, but considered an alternative when clinical judgement suggests the need for therapeutic change (conditional recommendation). This indication is not to be extended to patients with LVEF > 40%.

10.1.5.8. Sodium-Glucose Cotransporter-2 Inhibitors

In recent years, this class of drugs brought enthusiasm to the scientific community because of the demonstration of incremental benefit to the traditional treatment, in terms of prognostic improvement of HF and renal dysfunction. In this chapter, we revise whether the level of evidence is proportional to the enthusiasm and translate it into decision-making in the context of HF from CCCD.

Sodium-glucose cotransporter-2 (SGLT2) inhibitors are drugs originally tested for the treatment of hyperglycemia in patients with type 2 diabetes *mellitus* . The SGLT2 acts in the proximal convoluted tubule and responds for 90% of the reabsorption of the filtered glucose in the glomerulus. The SGLT2 inhibitors promote renal excretion of glucose, which is the mechanism of its glycemia reduction effect.^619^There were two initial observations regarding the intermediate effects: first, the efficacy of those inhibitors as glycemia reductors in individuals with diabetes is modest, with mean reductions in glycated hemoglobin ranging from 0.4% to 1.1%, as compared to placebo;^619 - 621^second, they promote consistent body weight reduction as compared to other antidiabetic drugs.^622 , 623^

The initial strategy of the manufacturers of this class of drugs was to assess its safety for individuals with diabetes, focusing on macrovascular outcomes (cardiovascular death, myocardial infarction, and stroke) and using counterintuitive approach of non-inferiority testing compared to placebo. Although counterintuitive, the deviation of the null hypothesis to a value different from zero is a suitable method to test safety, because a tolerance interval of adverse effect can be justified based on a demonstrated benefit.

Non-inferiority compared to placebo (safety) has been confirmed in several studies of that class of drugs,^624 - 626^which is scientifically valid. However, the clinical question persisted: in the absence of proven incremental benefit, would the demonstration of safety be a justification to recommend the addition of that treatment to patients with diabetes?

Then an apparent reduction in the HF-related outcomes in the groups treated was perceived. These were the secondary outcomes of studies, except for the DECLARE–TIMI 58 clinical trial,^624^which comprised a primary efficacy outcome if the non-inferiority hypothesis for severe event was demonstrated. SGLT2 inhibitors promote natriuresis, osmotic diuresis (due to glucosuria), and weight loss, mechanisms that *a priori* increase the probability of the benefit demonstrated. Based on those results, the hypothesis that the SGLT2 inhibitors improved the prognosis of patients with HFrEF was tested.

Thus, clinical trials with SGLT2 inhibitors began to be conducted in patients with symptomatic HF, regardless of the presence of type 2 diabetes *mellitus* . The DAPA-HF^627^and EMPEROR-Reduced^628^studies have assessed the effect of dapagliflozin and empagliflozin, respectively, on the incidence of the combined outcome of cardiovascular death and hospitalization from HF, as compared to placebo, in patients with HFrEF. The first to be published, the DAPA-HF study, included 4744 patients with HF and LVEF ≤ 40%, in NYHA functional class II to IV, already using optimized pharmacological therapy, and elevation of NT-proBNP levels. Diabetes *mellitus* was present in 42% of the sample and 99% of the individuals were in functional class II or III at the time of randomization. The HF etiology was nonischemic in 44% of the cases, with no mention to CD, although it was a multicontinental clinical trial, with approximately 17% of the participants recruited in Latin-American centers.^627^

The patients were randomized for the use of dapagliflozin, 10 mg/day, or placebo, at a 1:1 ratio. After a median 18-month follow-up, dapagliflozin was associated with a reduction in the risk for the primary outcome, which included cardiovascular death and hospitalization from HF (386 *versus* 502 events, respectively; HR 0.74; 95% CI, 0.65-0.85). The benefit was observed in both components of the primary outcome and proved to be consistent in the pre-specified analyses in different subgroups, such as the presence or absence of type 2 diabetes *mellitus* .^627^In addition, there was a reduction in the risk of all-cause death in the group treated with dapagliflozin *versus* the placebo group (276 vs 329; HR, 0.83; 95% CI, 0.71-0.97).

A substudy of the DAPA-HF study has more carefully assessed the potential influence of the HF etiology, classified as ischemic and nonischemic (hypertensive, idiopathic, “other”, and unknown cause), regarding the benefit of dapagliflozin on the primary outcome and found no change of effect.^629^

The safety profile of dapagliflozin was satisfactory, with low incidence of severe adverse events. Importantly, when assessing the eligibility criteria of the DAPA-HF study, systolic blood pressure < 95 mm Hg and glomerular filtration rate < 30mL/min/1.73m^2^were exclusion criteria.

The EMPEROR-Reduced study, published in 2020,^628^investigated the effect of empagliflozin, as compared to placebo, in patients with HFrEF (≤ 40%) on optimized medical therapy and defined inclusion criteria and primary outcome similar to those of the DAPA-HF study. However, the 3730 participants of the study (50% with type 2 diabetes *mellitus* ) showed higher mean levels of atrial natriuretic peptides and lower mean LVEF values as compared to those of the dapagliflozin study sample. Once more, CD was not represented as an etiology of HF, although 34% of the study participants had been recruited in Latin-American countries. After a median 16-month follow-up, empagliflozin reduced by 25% the combined risk of hospitalization from HF and cardiovascular death as compared to placebo (19.4% *versus* 24.7%; HR 0.75; 95% CI, 0.65-0.86). However, differently from the DAPA-HF study, that benefit seemed to be basically due to reduction in hospitalizations from HF. In the pre-specified subgroup analyses, the effect of empagliflozin on the primary outcome remained consistent.^628^

Similarly to the DAPA-HF study, in the EMPEROR-Reduced study, patients on SGLT2 inhibitor showed lower values of systolic blood pressure, body weight, and NT-proBNP after a 1-year follow-up as compared to baseline values.

More recently, the EMPEROR-Preserved trial^630^has extended the investigation of empagliflozin to patients with HFmrEF (> 40%). The relative reduction in the risk of events was similar to that observed in patients with LVEF ≤ 40%, which is expected, because an arbitrary limit of ejection fraction does not define two different diseases. Of note patients with HFmrEF have better prognosis, which reduces the absolute magnitude of the benefit: NNT of 19 in the first two studies with LVEF < 40% and NNT of 30 in the EMPEROR-Preserved trial.

Those studies have satisfactory statistical precision and low risk of bias. Thus, it seems adequate to state that there is effect of benefit, whose magnitude, represented by a 25% relative risk reduction, is at the level (marginal) of most therapies approved for HF. Therefore, from the pragmatic viewpoint, these drugs are safe and moderately beneficial.

Regarding cost-effectiveness, the recent incorporation of dapagliflozin into SUS was based on a report from the CONITEC presenting an economic model with incremental cost-effectiveness ratio of R$9296 per year of life saved with quality, being within an acceptable definition of efficiency.

However, there is one conceptual question: how much of the benefit of those drugs derives from diuretic therapy improvement *versus* how much of that is due specifically to the molecule innovation? There are reports of favorable effects of those drugs on intermediate outcomes, both metabolic and neuro-humoral, such as an increase in the circulating levels of vasodilators and a reduction in the levels of vasoconstrictors. However, clinical trials have not focused on the pertinent proof of concept that these are the effects that mediate the clinical benefit. None of them has generated a counterfactual (second control group) based on the diuretic therapy to answer the question: if a patient not receiving the innovative drug had the same level of diuresis improvement, would his outcome be different? This question could also be explored by use of mediation analysis (causal inference), with data from the clinical trials and a post-randomization mediator variable that represented the effect on diuresis. We have not detected this type of approach in the literature.

Finally, how to translate our interpretation of the evidence into a recommendation of therapy with gliflozins in individuals with HF of CD? Once more, that subpopulation has not been represented in clinical trials. In accordance with data presented for other contexts, generalization does not depend only on representativeness, and we do not know any probable mechanism of interaction that raises the suspicion that the etiology of CCCD modifies the effect of that therapy to the point of loss of efficacy. Therefore, we defined that there is level of evidence B for HF from CCCD, that is, indirect and of good quality. Regarding the strength of recommendation, in the absence of the counterfactual that there is benefit beyond the diuretic effect, we chose a conditional recommendation for patients with HFrEF, and prescription should be based on clinical findings suggesting the need for therapeutic increment.

The recommendations for the pharmacological treatment of HF in CCCD are shown in Table 10.1 and Figures 10.1 and 10.2 .

**Table 10.1:** – Recommendations for the pharmacological management of heart failure in CCCD

Heart failure with reduced ejection fraction (HFrEF): ≤ 40%	Recommendation grade	Level of evidence
Loop diuretics to control systemic or pulmonary congestion	Strong	C
Thiazide diuretic associated with loop diuretic to control persistent systemic or pulmonary congestion	Strong	C
ACEI to reduce morbidity and mortality	Strong	B
ARB for patients intolerant to ACEI (cough/angioedema) to reduce morbidity and mortality	Strong	B
Sacubitril-valsartan to replace ACEI/ARB for patients already on optimized triple therapy, who remain symptomatic (NYHA II or III), to reduce morbidity and mortality	Conditional	B
Carvedilol, metoprolol succinate, or bisoprolol to reduce morbidity and mortality in hemodynamically stable patients	Strong	B
Spironolactone associated with standard treatment with ACEI (or ARB) and BB to reduce morbidity and mortality (symptomatic CCCD, creatinine ≤ 2.5mg/dL, and serum potassium ≤ 5.0 mEq/dL)	Strong	B
Association of hydralazine and nitrate in patients with contraindication to ACEI/ARB (renal failure and/or hyperkalemia) to reduce morbidity and mortality	Conditional	B
Ivabradine for patients with optimized therapy, sinus rhythm, and HR > 70 bpm to reduce morbidity and mortality	Conditional	B
SGLT2 inhibitors (dapagliflozin or empagliflozin) for patients with or without diabetes, on optimized triple therapy, to reduce cardiovascular outcomes and progression to renal dysfunction	Conditional	B
Digoxin for symptomatic patients with AF and increased ventricular response, despite the use of BB, to reduce symptoms and hospitalizations	Conditional	B
Digoxin for symptomatic patients in sinus rhythm, despite optimized triple therapy, to reduce symptoms and hospitalizations	Conditional	B
**Heart failure with mildly reduced ejection fraction (HFmrEF): 41-54%**	**Recommendation grade**	**Level of evidence**
Loop diuretics to control systemic or pulmonary congestion	Strong	C
Thiazide diuretic associated with loop diuretic to control persistent systemic or pulmonary congestion	Strong	C
ACEI to reduce morbidity and mortality	Conditional	B
ARB for patients intolerant to ACEI (cough/angioedema) to reduce morbidity and mortality	Conditional	B
Carvedilol, metoprolol succinate, or bisoprolol to reduce morbidity and mortality	Conditional	B
Spironolactone associated with standard treatment with ACEI (or ARB) and BB to reduce morbidity and mortality	Conditional	B
Digoxin for symptomatic patients with AF and increased ventricular response, despite the use of BB, to reduce symptoms and hospitalizations	Conditional	B

CCCD: chronic cardiomyopathy of Chagas disease; BB: beta-blocker; ARB: angiotensin II receptor blocker; AF: atrial fibrillation; HR: heart rate; ACEI: angiotensin-converting-enzyme inhibitor; NYHA: New York Heart Association; SGLT2: sodium-glucose cotransporter-2.

**Figure 10.1:** – Algorithm for the pharmacological treatment of patients with heart failure and reduced ejection fraction. EF: ejection fraction; ACEI: angiotensin-converting-enzyme inhibitor; ARB: angiotensin II receptor blocker; SGLT2: sodium-glucose cotransporter-2; HR: heart rate; NSVT: nonsustained ventricular tachycardia; NYHA: New York Heart Association; AF: atrial fibrillation; H-N: hydralazine-nitrate.

**Figure 10.2:** – Algorithm for the pharmacological treatment of patients with heart failure and mildly reduced ejection fraction. EF: ejection fraction; ACEI: angiotensin-converting-enzyme inhibitor; ARB: angiotensin II receptor blocker; NSVT: nonsustained ventricular tachycardia; AF: atrial fibrillation.

## 10.2. Non-Pharmacological Resources

### 10.2.1. Cardiac transplantation

Despite all the advance observed in drug treatment, in intensive care, and surgical strategies, including the use of implantable cardiac devices, for the treatment of HF, this clinical syndrome persists with high morbidity and mortality and considerable economic impact on the health system, mainly in its more advanced phases.^631^

The CTX is still considered the best treatment for refractory HF, with evident influence on the patient survival increase and quality of life improvement, especially in CCCD, whose prognosis is poor as compared to HF of other etiologies.^632 - 634^Thus, CTX in advanced CCCD is considered a strong recommendation, with level of evidence B, similarly to that occurring in other cardiac diseases with classical indications, as long as there is no contraindication to the procedure and some peculiarities are considered, such as, unfavorable socioeconomic conditions and presence of megacolon and/or megaesophagus, which can increase the risks of postoperative complications and jeopardize the CTX result.^20^

10.2.1.1. Immunosuppression Strategies

The immunosuppressive regimens for CTX can be classified as of induction and of maintenance and they do not depend on the etiology of the HF leading to CTX indication. Induction regimens cause intense postoperative early immunosuppression, while maintenance regimens are used throughout life to prevent rejection.^635 , 636^

10.2.1.2. Induction Therapy

The induction therapy for transplanted patients consists in intense immunosuppressive treatment, during transplant or in the immediate postoperative period, used in those at high risk for rejection to reduce this risk or delay the use of higher doses of calcineurin inhibitors, minimizing renal damage, particularly in patients with impaired renal function.^635^Patients at high risk for fatal rejection, who could benefit from induction therapy are those with high titers of anti-HLA antibodies in the immune panel (PRA = panel reactive antibody > 10%), who are considered more vulnerable: young women with a previous pregnancy, patients with previous multiple transfusions, and users of mechanical circulatory support. The major inductors are polyclonal antithymocyte immunoglobulin (polyclonal antibody - thymoglobulin) and IL-2 receptor inhibitors, which have low immunogenicity, such as daclizumab and basiliximab.^635^

Although those agents can reduce the risk of early rejection and/or minimize renal damage, they are associated with an increased risk of infection, and, thus, can reactivate *T. cruzi* infection. Induction therapy is still controversial, and despite being used in 50% of the cardiac recipients, so far large RCTs have not been conducted to demonstrate the benefit of induction therapy *versus* no induction therapy.^636 , 637^There are no data available on its effects on a recipient with CCCD.

10.2.1.3. Maintenance Therapy

The basic maintenance immunosuppressive therapy in heart transplanted patients usually includes a calcineurin inhibitor, such as cyclosporine A or tacrolimus. These agents should be associated with mycophenolate mofetil (MMF) or mycophenolate sodium or azathioprine or rapamycin or everolimus. Prednisone is also added to this standard regimen, and, in most patients, it can and should be suspended approximately 6 months after transplantation if no rejection occurs.^635^

In the context of CCCD, the induction and/or maintenance immunosuppressive therapy can reactivate *T. cruzi* infection.^2 , 20^There is no study comparing immunosuppressive regimens in patients with CCCD; however, a higher number of reactivations has been diagnosed with the use of MMF *versus* azathioprine.^638^Thus, strategies to change the immunosuppressive regimen, such as replacement of MMF with azathioprine or MMF dose reduction, have been proposed, but these strategies have not been tested in RCTs.

The early reduction in immunosuppressive agents, mainly corticosteroids, is recommended to prevent RCD, but this can facilitate rejection episodes. So, patients with CCCD should receive the least intense immunosuppressive therapy possible, provided that there is no rejection.^2 , 20^

Table 10.2 summarizes the immunosuppression strategies after CTX in the context of CD.

**Table 10.2:** – Recommendations related to immunosuppressive therapy in CTX recipients with CCCD

Summary of recommendations	Recommendation grade	Level of evidence
Induction therapy should only be used in patients at high risk for acute rejection or renal function worsening: PRA > 10%; reduced renal function; previous pregnancy; previous multiple transfusions; users of MCS	Strong	C
Use immunosuppressive agents at the lowest possible dose	Strong	B
Prefer azathioprine or mycophenolate at a reduced dose in association with a calcineurin inhibitor (CsA or Tacrolimus)	Conditional	B
In the absence of rejection, suspend corticosteroids gradually 6 months after the CTX	Conditional	C
In the presence of rejection, readjust the regimen and dose of immunosuppressive agents	Strong	B

CCCD: chronic cardiomyopathy of Chagas disease; CsA: cyclosporin A; PRA: panel reactive antibody; MCS: mechanical circulatory support; CTX: cardiac transplantation.

### 10.2.2. Diagnosis and Treatment of Rejection

The incidence of rejection requiring treatment has progressively reduced over the years, currently affecting only 12.6% of the recipients in the first year after CTX.^636^Rejection is classified into hyperacute, mediated by antibodies, and acute cellular, which represents the most prevalent form of rejection. Histologically, rejection is defined by an inflammatory infiltrate, in which lymphocytes typically predominate, and myocyte damage. The International Society for Heart & Lung Transplantation (ISHLT) revised the categories of acute cellular rejection (R) as follows: 0R (no rejection), 1R (mild), 2R (moderate), or 3R (severe).^639^

Hyperacute rejection is an uncommon event, mediated by antibodies previously formed in the recipients, and manifests as severe graft failure within minutes or a few hours after the CTX procedure.^639^The frequency of hyperacute rejection after CTX due to CD has not been reported.

Acute cellular rejection occurs in 10% to 14% of the recipients with CCCD and there is no difference in the incidence of acute cellular rejection episodes (2R or 3R) between CTX recipients with or without CD.^636 , 639 - 643^

Endomyocardial biopsy remains the standard method for the diagnosis of rejection, and the frequency of the biopsies varies according to the transplant center protocol. Secondary myocarditis to reactivation of the *T. cruzi* infection can occur in the transplanted heart, making the differential diagnosis between rejection and RCD a great challenge.^390 , 635 , 643 , 644^

The definition for one of those two conditions is difficult if parasites are not found in the biopsy fragments. Depending on the routine histopathological staining techniques, if parasites are not seen, the histopathological inflammatory characteristics found in rejection (2R or 3R) and in RCD are verry similar. Thus, the mononuclear inflammatory infiltrate detection on the endomyocardial biopsy slides is not sufficient to rule out the diagnosis of RCD and represents a dilemma, because the aggressive immunosuppressive treatment to abort rejection can facilitate and intensify RCD.^390 , 644^The presence of nests of *T. cruzi* amastigotes with mononuclear inflammatory infiltrate in endomyocardial biopsy fragments does not exclude concomitant graft rejection, because the two conditions can occur simultaneously.^390 , 644^

Therapy for rejection in transplanted patients with or without CD is similar. Usually, mild rejection (1R), in the absence of clinical or hemodynamic impairment, requires no additional intervention. However, more severe rejection (≥ 2R) requires aggressive supplementary immunosuppressive therapy.^635 , 636^Rejection is a risk factor for RCD, and more than 85% of the patients have at least one episode of rejection before RCD occurs.^645^

### 10.2.3. Diagnosis and Treatment of T. cruzi-Infection Reactivation

10.2.3.1. Clinical Presentation

Immunosuppressive therapy increases the risk of RCD, whose incidence after CTX ranges from 19.6% to 90%.^2 , 638 , 642 , 643 , 646 - 649^Considering its potential morbidity and mortality, the diagnosis and proper management of RCD in the context of organ transplant is extremely important.

The CTX should be performed following a clinical and laboratory structured protocol to monitor the infection reactivation and its subsequent treatment.^2 , 20 , 648 - 651^The diagnosis of RCD is based on clinical signs and symptoms and on the presence of parasites in the blood, cerebrospinal and other fluids, bone marrow, or tissues.^2^

Monitoring is aimed at identifying the firsts signs of reactivation and timely establishing anti- *T. cruzi* treatment. The clinical reactivation has cardiac and extracardiac manifestations that include: myocarditis; ventricular dysfunction; arrhythmias; new atrioventricular/intraventricular blocks on ECG; skin lesions (subcutaneous nodules, panniculitis); fever; bone marrow impairment; and neurological manifestations, such as meningoencephalitis, chagomas, cerebral abscess, and stroke.^2 , 640 , 642 , 643 , 651 - 653^The myocarditis of reactivation can be mistakenly diagnosed as graft rejection and treated with intensification of the immunosuppressive treatment, which will worsen reactivation.^644^The differential diagnosis between myocarditis of rejection and of reactivation is still a great challenge.^390 , 644 , 654^In the presence of inflammatory infiltrate, nests of amastigotes and/or positive PCR for *T. cruzi* in the myocardium, we can claim that reactivation is present, but associated graft rejection cannot be safely excluded. Despite this complexity, the survival rate of recipients with CCCD undergoing CTX does not differ from those of cardiomyopathies of other etiologies.^2 , 636 , 640 , 643^

10.2.3.2. Parasitological Diagnosis of Reactivation

The objective of laboratory monitoring is to identify any subclinical sign of RCD before cardiac and extracardiac symptoms appear, as well as graft dysfunction.^2 , 649 - 651 , 655^Serological tests are useful only for potential donors, diagnosis of CCCD in potential recipients, and seronegative recipients who receive organs from seropositive donors.^2^They play no role in the diagnosis of RCD.

Traditionally, laboratory monitoring uses parasitological methods (direct search for *T. cruzi* and blood cultures) and serial histological testing of endomyocardial biopsy, in the search for *T. cruzi* amastigotes, but those are low-sensitivity tests.^2^In recent years, several studies have evidenced the value of PCR in peripheral blood and myocardium to detect early RCD before the appearance of symptoms and/or graft dysfunction.^388 , 390 , 391 , 401 , 403 , 656 - 658^

Regarding the frequency of clinical visits and laboratory monitoring, there is no consensus in the literature. Table 10.3 suggests a protocol for clinical, laboratory, and histological monitoring of patients with CCCD undergoing CTX, in addition to a suggestion of etiological treatment, based on the major guidelines available.^2 , 20 , 647 , 650 , 651^

**Table 10.3:** – Clinical, histological, and laboratory monitoring of *T. cruzi* infection reactivation after CTX in Chagas disease and etiological treatment

Procedure	Recommendation grade	Level of evidence
**Before transplantation**		
Serological tests for Chagas disease in the donor	Strong	C
Serological tests for Chagas disease in the potential recipient with possible CCCD	Strong	C
**After transplantation**		
Periodical clinical consultations with attention to signs/symptoms of reactivation, including ECG and echocardiogram	Strong	C
Routine *T. cruzi* search in the blood (smear, blood culture) to diagnose infection reactivation	Strong	C
Routine *T. cruzi* search in the blood by use of PCR	Strong	C
Periodical routine endomyocardial biopsies with *T. cruzi* search (histology and immunohistochemistry)	Strong	C
Periodical routine endomyocardial biopsies with *T. cruzi* search by use of PCR, if available	Strong	C
*T. cruzi* search in tissues (skin, bone marrow) in the presence of findings compatible with *T. cruzi* infection reactivation	Strong	C
Frequency of post-transplantation procedures:	Conditional	C
• 1^st^month: weekly • 2^nd^month: every two weeks • 3^rd^to 6^th^month: monthly • 7^th^to 12^th^month: every 3 months • After 12 months: every 6 months		
**Etiological treatment of reactivation**		
Benznidazole 5mg/kg/day for 60 days	Strong	C

CCCD: chronic cardiomyopathy of Chagas disease; CTX: cardiac transplantation; PCR: polymerase chain reaction.

10.2.3.3. Etiological Treatment of Reactivation

In the presence of signs/symptoms and/or identification of the parasite in blood, cerebrospinal fluid, or tissue specimens, etiological treatment should be initiated immediately.^2 , 20^Benznidazole is recommended as first-line treatment.^2 , 563^Its tablets contain 100mg of active substance. Its absorption is through the gastrointestinal tract, its excretion is predominantly renal, and its half-life is 12 hours. Its recommended dosage is 5mg/kg/day, for 60 days, the daily dose being divided into two or three doses.^2 , 563^Its most important side effect is urticarial dermatitis, which occurs in approximately 30% to 60% of the patients as early as by the end of the first week of treatment, but with good therapeutic response to antihistamines or small doses of corticosteroids. A few individuals can have fever and adenomegaly, whose presence determines medication suspension. Other adverse effects include polyneuropathy (later), with pain and/or paresthesia in lower limbs, and anorexia. Significant leukopenia and agranulocytosis are rare but, when present, determine treatment interruption.^2 , 563^Nifurtimox is not routinely available in Brazil. These trypanocidal medications are contraindicated to pregnant women and patients with important renal or hepatic failure.^2^There is no sufficient evidence to support the prophylactic anti- *T. cruzi* treatment for RCD. It is worth considering that those drugs have important side effects, not every recipient has RCD, and one patient can have more than one episode of RCD after treatment. Monitoring of reactivation should be maintained even after anti- *T. cruzi* treatment^2 , 20 , 650 , 651^( Table 10.3 ).

10.2.3.4. Post-Heart Transplant Complications and Survival

The clinical outcomes, morbidity and mortality, in CTX recipients with or without CD are similar.^636 , 640 - 642^In both categories of patients, the major complications reported after CTX are almost the same: graft dysfunction (20%); rejection 2R or 3R (10%-14%); postoperative bleeding (10%); infection not related to *T. cruzi* (20%-30%); and acute renal failure (up to 70%). However, in recipients with CCDC, graft coronary artery disease appears to be less frequent, while the incidence of neoplasms seems to be higher, although none of these reported differences has been confirmed in all case series.^584 , 640 - 642 , 659^

Despite all the complexity of CD in the context of CTX, the results are good. In Brazil, the survival rates of patients with CCCD undergoing CTX in 6 months, 5 years, and 10 years are 76%, 71%, and 46%, respectively, superior to those of the cohort of patients undergoing CTX due to other etiologies.^641 , 642^One reason for that better performance would derive from the baseline characteristics, since patients with CCCD are usually younger, have fewer comorbidities, and less often have undergone previous cardiac surgery.^584 , 640 - 642^However, a recent study has reported the evolution of 376 transplanted patients between 1997 and 2019 in a single center in Northeastern Brazil, comparing the following etiologies of HF: CCCD, CMI, and nonischemic cardiomyopathy. A mean 5-year follow-up evidenced stability in survival for individuals with CCCD, while that parameter improved subsequently in the other two groups.^660^

### 10.2.4. Mechanical Circulatory Support

Mechanical circulatory support devices (MCSDs) are used to restore tissue perfusion in patients with advanced HF or cardiogenic shock refractory to optimized clinical therapy, including the use of inotropic drugs. They can provide support to the left or right ventricle, or both.^661^

Mechanical circulatory support can be indicated as bridge to CTX or to recovery, in the perspective of ventricular function improvement after acute injury, or even as a bridge to decision-making in critical patients, when improvement of clinical findings is uncertain. In specific situations, mainly in the presence of contraindication to CTX, MCSDs can be used as permanent therapy.^21^

Evidence on the use of MCSDs for patients with CD is limited to a few reports or case series, mainly as bridge to CTX.^662 - 666^The indication and contraindication criteria for the use of MCSDs in patients with CD can be the same applied to other etiologies.^661^Although RV systolic dysfunction is relatively common in patients with CCCD, especially in those who also have LV systolic dysfunction, there is no consensus regarding the choice of the most appropriate type of device in this condition.^667^

The first report of the successful use of MCSDs as bridge to CTX in a patient with CCCD occurred in 1994.^662^After that, in a noncontrolled phase I clinical trial, including six patients with advanced biventricular HF, Moreira *et al* . reported the successful use of left MCSD as bridge to CTX in only two cases.^663^

In developed countries, MCSDs have been implanted in immigrants with HF due to CD. Kransdorf *et al* . have reported the use of biventricular MCSD in two patients with CD from a cohort of patients undergoing CTX in the USA.^664^Ruzza *et al* . have reported a successful case of total artificial heart as bridge to CTX in a patient with CCCD.^665^In the Netherlands, a MCSD has been implanted as bridge to CTX in a patient with refractory HF due to CD.^666^More recently, Atik *et al* . have reported another successful case of axial flow left MCSD used in a patient with CD and biventricular systolic dysfunction.^667^

Mechanical cardiac support is usually considered to have a high potential for success as bridge to CTX, recovery, decision-making, or permanent therapy in patients with CCCD. However, currently, the major limitations to its applicability are high cost, RV dysfunction, and need for a specialized team for device implantation and management. The SBC guideline on mechanical circulatory support recommends careful assessment of the RV function as mandatory before implantation, and, in the presence of moderate to severe dysfunction, one should be prepared for biventricular support implantation.^661^

The major indices to assess RV dimensions and function are: RV longitudinal and radial contractility semiquantitative assessment; fractional area change; tricuspid annular plane systolic excursion (TAPSE) on M mode; lateral tricuspid annulus peak systolic velocity estimated by tissue Doppler (s’); and RV myocardial performance index. Left ventricular MCSD implantation should be considered with restrictions to patients with CCCD and significant RV dilation, moderate to severe tricuspid insufficiency, tricuspid annulus > 45 mm, and central venous pressure (CVP) > 15mmHg.^661^The hemodynamic parameters considered optimal regarding RV function and that would reduce the risk of RV dysfunction after implantation are: central venous pressure ≤ 8mmHg, pulmonary capillary pressure (PCP) ≤ 18mmHg, CVP/PCP ≤ 0.66, pulmonary vascular resistance < 2 UW, and RV work index ≥ 400mL/m^2^.^661^

## 11. Therapeutic Management of Cardiac Arrhythmias

### 11.1. Pharmacological Resources

#### 11.1.1. Introduction

Medical literature related to arrhythmia treatment and sudden death prevention in CCCD is scarce and insufficient for the formulation of strong recommendations supported by evidence directly obtained from randomized studies and that indisputably proves therapeutic efficacy (level of evidence A).^1 , 668^However, CCCD shares similarities with several extensively studied cardiopathies, particularly those with myocardial fibrosis and systolic dysfunction (global or segmental), such as ICM and DCM,^669^allowing the therapeutic rationale to follow similar pathophysiological bases. Thus, the treatment and prevention of ventricular and supraventricular arrhythmias in CCCD tend to follow directions similar to those of the other cardiopathies.

However, some specific characteristics that can influence antiarrhythmic treatment are usually more striking in CCCD. Sinus node dysfunction, atrioventricular and intraventricular conduction disorders, and ventricular arrhythmias are frequently found in both asymptomatic and more advanced disease forms.^669 - 672^Despite the classical direct relationship between the degree of ventricular dysfunction and the higher frequency of ventricular arrhythmia, the prevalence of ventricular arrhythmias in CCCD is higher as compared to that in other cardiopathies.^672 , 673^

In addition, RV impairment,^343^presence of intracardiac thrombi,^674^and cardiac dysautonomia due to parasympathetic neuronal lesion^222^are more frequent in CCCD. All those factors could explain the shorter survival of patients with CCCD as compared to that of patients with cardiomyopathies of other etiologies and similar myocardial damage.^675^

Sinus node dysfunction and atrioventricular and intraventricular conduction disorders require more caution with the use of beta-blockers, digitalis, and amiodarone, because of the risk of excessive bradycardias and appearance or worsening of preexisting blockades.

In addition, ventricular tachyarrhythmias demand treatment with drugs frequently associated with severe side effects. Right ventricular impairment, present in 42% of the patients with LV dysfunction,^343^tends to cause more systemic congestion, requiring higher doses of diuretics that can induce severe hypopotassemia, increasing the risk for global, sudden, and cardiovascular death.^676^In this context, in addition to the routine use of aldosterone inhibitors (spironolactone/eplerenone),^606 , 677^oral supplementation with potassium, aimed to maintain its serum levels between 4.0 and 5.0mEq/L, might be necessary.

#### 11.1.2. Sudden Death Prevention with Non-Antiarrhythmic Drugs

Sudden death, sometimes unexpected and affecting individuals with good functional capacity during exercise training, clearly predominates in outpatient subpopulations with CCCD.^352 , 408^The RASSI score, developed in that subgroup of CCCD, stratifies the risk of total mortality, which includes the predominant occurrence of sudden death.^408^

However, many of those patients often have varied ventricular dysfunction degrees and clinically manifest HF. The optimized HF treatment in patients with CCCD is likely to result in a potential auxiliary benefit to prevent malignant ventricular arrhythmia and its most feared consequence, sudden death. This has not been specifically proven, and only a few studies on HF have included small samples of patients with HF due to CD.

Thus, the use of some pharmacological treatments for CCCD complicated with HF, aimed at reducing sudden death, has been extrapolated from results obtained in patients with HF of other etiologies, assuming the existence of clinical and pathophysiological similarities between them.

Optimized dosages of ACEI or ARB, as well as of beta-blockers (carvedilol, bisoprolol, and metoprolol succinate) and spironolactone, should be aimed at for HF due to CD with that auxiliary antiarrhythmic perspective.^678^For example, the MERIT-HF trial, comparing metoprolol succinate *versus* placebo for patients with HF and ejection fraction ≤ 40%, was early discontinued (after a mean 12-month follow-up) due to a 40%-60% reduction in overall mortality, in mortality from HF worsening, and in sudden death.^679^Similar results have been observed with carvedilol and bisoprolol in patients with HFrEF.^680 , 681^

It is worth noting that in the REMADHE observational study, beta-blockers were less often used for patients with CCCD than for those with other etiologies.^605^In a small, but CCCD-specific study, carvedilol has been well tolerated and associated with a trend to increase LVEF.^682^More recently, in the PARADIGM-HF trial, in patients with HFrEF, sacubitril-valsartan significantly reduced the incidence of sudden death as compared to enalapril at a non-optimal dose in the group receiving ICD (51% reduction) and in that not undergoing ICD implantation (19% reduction).^683^Thus, other recommendations from international guidelines might be applied to reduce total and sudden death in CCCD with HF.^684^

#### 11.1.3. Ventricular Arrhythmias in Cardiopathies of Other Etiologies

Ventricular arrhythmias can occur in any cardiopathy and manifest as: monomorphic or polymorphic, isolated, bigeminy, trigeminy, and paired VE; NSVT or SVT, which can also be monomorphic or polymorphic. Ventricular arrhythmias can be asymptomatic and, in their most severe forms (SVT and VF), cause syncope, low output, and sudden death.

The prevalence of NSVT episodes on 24-hour Holter has ranged from 21% to 25% in the SCD-HeFT study, which assessed mortality of patients with HF of ischemic and nonischemic etiologies.^685^The EMIAT study, assessing patients after myocardial infarction with LVEF < 40%, has reported prevalence of ventricular arrhythmia (defined as 10 or more VE/hour or NSVT on Holter) in 39% to 41% of the individuals.^686^The GESICA study, in patients with severe HF of several etiologies, has reported high occurrence of VE > 10/hour (71%), paired VE (56%), and NSVT (33%) on Holter.^687^

#### 11.1.4. Amiodarone for Patients with Cardiopathies of Other Etiologies: Primary Prevention

Amiodarone has the four antiarrhythmic effects of the Vaughan-Williams classification: sodium channel block (class I); noncompetitive alpha- and beta-adrenergic inhibition (class II); interference with potassium channels, leading to prolongation of the action potential, repolarization, and refractoriness (class III); and calcium channel block (class IV).

The use of amiodarone *versus* placebo, other antiarrhythmic drugs, or control group for the primary prevention of total mortality and sudden death has been assessed in several meta-analyses. In 1997, the ATMA study,^688^using individual data of patients from eight RCTs after acute myocardial infarction (EMIAT, CAMIAT, GEMICA, PAT, SSSD, BASIS, Hockings *et al* ., and CAMIAT-P) and five studies including patients with congestive HF (CHF-STAT, GESICA, EPAMSA, Nicklas *et al* ., and Hamer *et al* .), showed a reduction of 13% in the risk of total death (p = 0.03) and of 29% in the risk of arrhythmogenic sudden death (p = 0.0003) with amiodarone. There was no excess of nonarrhythmic deaths with amiodarone, and both groups of patients (after acute myocardial infarction and congestive HF) benefited from the antiarrhythmic treatment.

In that same year, another meta-analysis,^689^using Bayesian hierarchical modeling and data from the same 13 studies included in the ATMA trial in addition to two other studies (CASCADE and ASSG) involving survivors of cardiac arrest or SVT, concluded that amiodarone reduces all-cause mortality by approximately 19% (p < 0.01) and determines slightly higher reductions in cardiac mortality (23%, p < 0.001) and sudden death (30%, p < 0.001). There was a trend towards a higher reduction in the risk of death in the studies requiring evidence of frequent or complex ventricular ectopia as an inclusion criterion (25%) as compared to other studies (10%).

With the encouraging amiodarone results and the ICD emergence, the next most likely step would be comparison focused on random assignment of patients treated with amiodarone or ICD or placebo in primary prevention of death from any cause. This was the major objective of the SCD-HeFT study, published in 2005, including 2521 patients with ejection fraction ≤ 35%, NYHA functional class II or III, HF of ischemic origin in 52% of the patients and nonischemic in the remaining.^685^After a median 45.5-month follow-up, total mortality was 29% in the placebo group, 28% in the amiodarone group, and 22% in the ICD group, that is, while amiodarone had no effect on total mortality as compared to placebo, the ICD therapy caused a 23% relative risk reduction (p = 0.007).

Based on pre-specified subgroup analysis, the results did not vary according to HF etiology, but varied according to NYHA functional class. Thus, in class III patients, an increase in mortality was observed with amiodarone (as compared to placebo), while no difference was observed between the ICD and placebo treatments. Despite the extremely significant (p < 0.001) interaction between ICD and functional class, the authors ignored these results and concluded that, in both classes (II and III), therapy with single-chamber ventricular ICD could reduce total mortality.

According to that same paradigm, all guidelines began to recommend ICD, prophylactically, in patients with ejection fraction ≤ 35% and NYHA functional class II and III. Despite the indisputable results of the SCD-HeFT study, two limitations apply. First, the inclusion criterion was ventricular dysfunction and not the recording of complex and frequent ventricular arrhythmia on Holter. Second, of all subgroup analyses, the most important one, from our viewpoint, comparing amiodarone to placebo in patients with recorded NSVT (22% of the study population), for unknown reasons, was not contemplated.

In the CHF-STAT study,^690^randomizing 674 patients with HF (ejection fraction ≤ 40%) of ischemic and nonischemic etiology and at least 10 VE/hour on 24-hour Holter to receive amiodarone or placebo, after a median 45-month follow-up, amiodarone significantly reduced the ventricular arrhythmia frequency and improved ventricular function but could not increase survival.

In pre-specified subgroup analysis and based on randomization stratified according to HF etiology, there was a trend towards lower mortality with amiodarone in nonischemic patients (p = 0.07). At the time the SCD-HeFT study was published, the results of two small RCTs, EPAMSA (127 patients)^691^and AMIOVIRT (103 patients)^692^were known. The first compared amiodarone with a control group in patients with ischemic and nonischemic heart disease, ejection fraction < 35%, and Lown’s grade 2 or 4 ventricular arrhythmia on Holter. The second compared amiodarone with ICD for exclusively nonischemic HF, ejection fraction ≤ 35%, and NSVT on Holter. In the Argentine pilot study EPAMSA, which included 24 patients with CCCD, after a 1-year follow-up, the reductions in total death and sudden death with amiodarone were 71% (p = 0.02) and 71% (p = 0.04), respectively.^691^

In the AMIOVIRT study, early terminated because of the futility criterion, survivals after 1 year (90% *versus* 96%) and 3 years (88% *versus* 87%) did not statistically differ between the amiodarone and the ICD groups (p = 0.8), respectively.^692^

Positive results with amiodarone have also been observed in another Argentine study (GESICA),^687^which included 516 patients with severe HF of ischemic and nonischemic etiology (48 patients with CCCD), mainly NYHA functional class III or IV, presenting at least two of the following three criteria: ventricular systolic dysfunction indices: CTI > 0.55; ejection fraction ≤ 35%; and LVDD ≥ 32 cm/m^2^. Patients were randomized to an amiodarone or a control group. After a mean 13-month follow-up, total mortality was 41.4% in the control group and 33.5% in the amiodarone group, a relative risk reduction of 28% (p = 0.024). Patients were randomized according to the presence of NSVT on Holter on admission, which was observed in 33.5% of the entire population studied. The reduction in the risk of death with amiodarone occurred regardless of the presence of ventricular arrhythmia but was numerically higher in patients with recorded NSVT (34% *versus* 24.5%).

More recently, the long-term results of the SCD-HeFT study have been published.^693^After a 11-year follow-up, the benefit of ICD as compared to placebo remained statistically significant, but attenuation of effect was observed, with relative reduction in the risk of death decreasing from 23% (after 45.5 months) to 13% (after 11 years, p = 0.028). In addition, there was a significant interaction between follow-up duration (less or more than 6 years) and benefit from ICD (p < 0.0015) and the subgroup analysis according to HF etiology showed heterogeneous long-term results. While the beneficial effect of ICD persisted in patients with ischemic HF (RRR 19%, p = 0.009), in those with nonischemic HF, a reduction in mortality with the use of ICD was no longer observed (RRR 3%, p = 0.802). Considering that, after the publication of the original trial, more than half of the patients assigned to the placebo or amiodarone group received an ICD or resynchronization therapy and that the recommended statistical analysis was “intention to treat”, this crossover might have interfered with the results. However, these results did not change when the “as treated” analysis was used, comparing the groups according to the treatment received and not according to the initial assignment.

After the SCD-HeFT study publication, more meta-analyses were performed. The first meta-analysis^694^identified 15 studies (only 1 of secondary prevention, OPTIC), with a total of 8522 patients randomized to amiodarone or placebo/control. Amiodarone reduced the risk of sudden death by 29% (p < 0.001) and of cardiovascular death by 18% (p = 0.004). The reduction in the risk of all-cause mortality (13%) did not reach statistical significance (p = 0.093). Pre-specified subgroup analysis showed a 19% reduction in the risk of total death (95% CI, 2%-32%) with amiodarone doses > 200mg/day. However, doses ≤ 200mg/day were not effective (1% reduction; 95% CI, -31% to 25%). In addition, the use of amiodarone was associated with a two- and five-fold increase in the risk of pulmonary and thyroid toxicity, respectively. The authors concluded that amiodarone is a feasible alternative to prevent sudden cardiac death in ineligible patients or in those with no access to ICD therapy.^694^

Another systematic review with meta-analysis, following the Cochrane systematic review guidelines, was published in 2015^695^and included 24 RCTs with a total of 9997 patients. The objective was to compare amiodarone *versus* placebo/control or other antiarrhythmic drugs in primary (high-risk patients for sudden death) and secondary (survivors of cardiac arrest or patients with syncopal SVT) preventions.

In the studies of primary prevention (total of 18), amiodarone significantly reduced sudden, cardiovascular, and all-cause mortality, but the quality of the evidence was considered low (compared to placebo) or moderate (compared to other antiarrhythmic drugs). In the studies of secondary prevention (total of 6), no reduction in sudden and all-cause mortality was observed, and the quality of the evidence was considered low or very low.^695^

Based on those results: 1) it is reasonable to conclude that, as compared to placebo or control group or other antiarrhythmic drug, regarding primary prevention, amiodarone modestly reduces all-cause death, having a more expressive reducing effect on sudden death in patients with HF of both ischemic and mainly nonischemic etiology; 2) it is plausible to speculate that the beneficial effect of amiodarone is higher when NSVT and high ventricular arrhythmia density can be recorded on Holter, which seems to be highly relevant in the presence of ventricular dysfunction. Corroborating this assumption, a meta-analysis^696^including 11 studies with patients with HF (ischemic and nonischemic) or nonischemic DCM associated with LV dysfunction has shown that the presence of NSVT on Holter is an independent predictor of sudden cardiac death (OR 3.03; 95% CI, 2.44-3.77); 3) the only RCT that compared directly amiodarone to ICD in primary prevention (AMIOVIRT)^692^has shown no superiority of ICD; however, that study was limited by its small sample size.

The SCD-HeFT study,^685 , 693^although not comparing amiodarone to ICD, but each of them to placebo instead, did not require the presence of ventricular arrhythmia as an inclusion criterion, did not perform subgroup analysis based on the presence of NSVT, and showed ICD benefit only for patients in NYHA functional class II (but not class III), a benefit that was maintained in the long run only for those with ischemic HF. All these aspects should be considered when attempting to extrapolate the SCD-HeFT study results to CCCD.

#### 11.1.5. Amiodarone for Patients with Cardiopathies of Other Etiologies: Secondary Prevention

Secondary prevention of sudden death relates to patients who recovered from cardiac arrest due to VF or pulseless VT, or who already had at least one documented SVT episode. The other example of this group are patients with syncope of cardiac etiology, whose EPS showed induction of VF or hemodynamically unstable (or even stable according to some authors) SVT.

Sustained ventricular tachyarrhythmias have been typically grouped into a single category and collectively named “life-threatening” or “malignant”. Although VF predictably precipitates cardiac arrest, unless if its duration is short and reverts spontaneously (a very rare and poorly documented event), SVT has a wide range of hemodynamic and clinical manifestations.

Thus, indiscriminate grouping of these arrhythmic entities should be avoided because they differ regarding their prognoses and treatments. The ventricular dysfunction degree (expressed by LVEF) and the symptoms associated with the arrhythmia and the type of structural cardiopathy should be considered during the patients’ assessment.

The dichotomizing cutoff point for LVEF has usually been 35% or 40%, and a symptom grading has proposed four classes: I - no symptom or only palpitations; II - lipothymia, chest pain or dyspnea; III - syncope, altered mental state or other evidence of important hemodynamic impairment (signs and symptoms of low output, acute pulmonary edema); and IV - cardiac arrest (neither pulse nor breathing).^697^

It is very likely that the prognosis and treatment of a patient with ischemic heart disease, who recovered from cardiac arrest due to VF and with LVEF of 30%, differ from those of a patient with DCM, such as CCCD, hemodynamically stable SVT, relatively preserved LVEF, and palpitations.

Three RCTs (AVID, CIDS, and CASH)^698 - 700^have compared amiodarone (or other antiarrhythmic drug) with ICD for secondary prevention of all cause death.

The AVID study,^698^the first and largest of them, has assessed 1016 patients (81% with ischemic heart disease) who had been resuscitated from cardiac arrest due to VF (45% of the patients) or who had syncopal SVT (21%) or SVT, with LVEF ≤ 40% and symptoms suggestive of severe hemodynamic impairment (34%). The patients were randomized for therapy with ICD or antiarrhythmic drugs (amiodarone in 96% of the cases), had a mean age of 65 years, mean LVEF of 32% in the ICD group and 31% in the antiarrhythmic group, and 79% were of the male sex. After a mean 18.2-month follow-up, the study was early terminated because of the ICD superiority in reducing total death (15.8% *versus* 24%), with relative risk reductions of 39%, 27%, and 31%, after 1, 2, and 3 years of follow-up, respectively (p < 0.02, adjusted to multiple analyses).

The Canadian Implantable Defibrillator Study (CIDS)^699^has randomized 659 patients (83% with ischemic heart disease) for ICD or amiodarone, 48% of whom had survived cardiac arrest due to VF, 13% had syncopal SVT, 25% had SVT (HR ≥ 150 bpm), LVEF ≤ 35%, causing presyncope or angina, and 14% had syncope and SVT induced by programmed electrical stimulation. Their mean age was 64 years, mean LVEF was 34% in the ICD group and 33% in the amiodarone group, and 85% were of the male sex. After a mean 3-year follow-up, there was a nonsignificant reduction in the annual risk of total death (10.2% *versus* 8.3%, p = 0.142) and of arrhythmic death (4.5% *versus* 3.0%, p = 0.094) with ICD.

The smallest of the three studies, the CASH,^700^was performed in the city of Hamburg, Germany, and compared ICD to different antiarrhythmic drugs (amiodarone, metoprolol, and propafenone). Differently from the previous trials, it included only patients resuscitated from cardiac arrest (due to VF, 84%, and due to VT, 16%). The propafenone arm (58 patients) was discontinued after a mean 11.3-month follow-up because of a mortality rate 61% higher than that observed in the ICD group. The other patients, total of 288 equally assigned to the ICD, amiodarone, and metoprolol groups, remained in the study. The mean age was 58 years, mean LVEF of 46%, 80% were of the male sex, and 73% had ischemic heart disease. In a mean 57-month follow-up, all-cause mortality was lower in the ICD arm as compared to the amiodarone/metoprolol arm (36.4% *versus* 44.4%), although the difference did not reach statistical significance (1-sided p = 0.08).

The early termination of the AVID study, which could have overestimated the benefit of ICD, in addition to the smaller number of patients enrolled in the CIDS and CASH studies, which could have reduced the power of the statistical tests to detect a real benefit of the ICD treatment, have motivated the conduction of a meta-analysis comparing ICD exclusively with amiodarone.^701^Individual patient data from the three studies were merged into a master database according to a pre-specified protocol and the results were published in 2000. The meta-analysis included 1866 patients (ICD = 934; amiodarone = 932) with mean age of 63.5 years, mean LVEF of 33.5%, most of them of the male sex (81.5%) and diagnosed with ischemic heart disease (82%). The estimates of ICD benefit from the three studies were consistent with each other (p heterogeneity = 0.306). There was a 28% relative risk reduction in death from any cause (HR = 0.72; 95% CI, 0.60-0.87; p = 0.0006) and a 50% reduction in arrhythmic death (HR = 0.50; 95% CI, 0.37-0.67; p < 0.0001) with the ICD, reflecting a mean survival gain of 4.4 months after a mean 6-year follow-up.

However, in the subgroup analysis, the benefit related to survival increase with ICD was observed only in patients with LVEF ≤ 35% (HR = 0.66) and not in those with LVEF > 35% (HR = 1.2; p, interaction = 0.01). Although the use of beta-blockers was higher in the ICD group (42% *versus* 19%) and the benefit of ICD therapy also higher in those on beta-blockers (HR = 0.58 *versus* HR = 0.88), this difference was not statistically significant (p, interaction = 0.095).^701^

Based on the results from those RCTs,^698 - 700^their meta-analysis,^701^and some observational studies,^702^the major international guidelines^684 , 703^began to strongly recommend therapy with ICD to all patients resuscitated from cardiac arrest due to VF or VT, as well as to all patients with structural cardiopathy and SVT, regardless of the LVEF and the presence and type of arrhythmia-related symptoms, except, obviously, for the cases of arrhythmias due to probably secondary or reversible causes, such as electrolytic disorders, myocardial ischemia, and proarrhythmia, or individuals with life expectancy shorter than 1 year.

It is worth noting that none of those three RCTs included patients with hemodynamically stable or well-tolerated SVT and the analysis of the subgroups of the previously mentioned meta-analysis showed no benefit of ICD as compared to amiodarone in patients with LVEF > 35%. In addition, according to these guidelines (potentially biased when generalizing, despite the evidence of problems with the analyses of the results), only when ICD therapy is unavailable or contraindicated or refused by the patient, amiodarone could be used in an attempt to reduce sudden death (class IIb).^684 , 703^

#### 11.1.6. Ventricular Arrhythmias in Patients with Chronic Cardiomyopathy of Chagas Disease: Characteristics and Treatment

As already mentioned, although more commonly found in more advanced phases of CCCD, ventricular arrhythmias can occur at the early disease stages and even in the absence of significant global systolic ventricular function impairment.^672^

11.1.6.1. Ventricular Extrasystoles

Ventricular extrasystoles are present in 86% to 88% of the patients with CCCD without HF (functional classes I and II) and in almost all patients with HF (functional classes III and IV) on 24-hour Holter.^672 , 704^The density of VE is also elevated, so that 45% and 89% of the patients without and with HF, respectively, have more than 1000 VE/h on Holter.^672^

When VEs occur in asymptomatic patients with preserved ventricular function, they require no treatment. However, in asymptomatic patients with high arrhythmia density (> 16-20% of VE on 24-hour Holter), tachycardiomyopathy may develop,^705^that is, ventricular systolic dysfunction caused or worsened by the arrhythmia, which can be attenuated or reversed with suppression of the VEs, and, thus, survival may be increased.^706^

When the VEs are very symptomatic, even in the absence of ventricular dysfunction or late enhancement (fibrosis) on CMRI, the use of antiarrhythmic drugs is mandatory, but should be individualized, and amiodarone should be avoided at first, because of its adverse effects. In this situation, the use of a beta-blocker (e.g., nadolol or sotalol) or propafenone is suggested. However, in the presence of ventricular dysfunction or arrhythmogenic substrate of fibrosis on CMRI, class I antiarrhythmic drugs should not be used, because of their proarrhythmic effects and occasional negative inotropic effect, which could increase mortality, as described for other cardiopathies.^707^Thus, amiodarone can be indicated, at the dosage of 200 to 600 mg/day, because of its high efficacy in significantly reducing the VE density.^708^

11.1.6.2. Nonsustained Ventricular Tachycardia

Nonsustained ventricular tachycardia affects 42% of the patients with CCCD without HF and 89% of those with HF,^672^a prevalence much higher than that in other cardiopathies. It can be observed even in patients with normal ventricular function and its detection on Holter or during exercise testing is an independent marker of poor prognosis.^408 , 436 , 458^When associated with LV dysfunction (global or segmental), a relatively common finding in CCCD, it increases the risk of death by 15 times as compared to that of patients without NSVT and with normal ventricular function.^458^In the absence of data to assess relevant outcomes, such as mortality and hospitalization, a meta-analysis of old studies with amiodarone in CCCD has shown an important reduction in the ventricular arrhythmia density on serial Holter records (93% of isolated VEs, 79% of paired ones, and 100% of the VT episodes).^708^

As already mentioned, the Argentine RCTs GESICA^687^and EPAMSA,^691^including patients with CCCD, have shown a reduction in mortality with amiodarone. However, the small number of individuals with CCCD in those two studies (only 72) hinders a definitive conclusion. Thus, how to manage patients with NSVT? In the absence of ventricular dysfunction, the pharmacological treatment should follow, in general, the same recommendation for the treatment of VEs. When ventricular dysfunction is present, three options are available: beta-blocker, amiodarone, and ICD, which is discussed ahead.

The treatment should be aimed at relieving symptoms (when present), improving ventricular function, and preventing sudden death. Because there are no convincing data derived from RCT to support any of those three options, the recommendations for the treatment of those patients are based on extrapolation of the results from the studies performed in other heart diseases and on CCCD-related observational data, which are limited.

After a thorough analysis of the literature, we chose to preferably indicate a selective beta-blocker (metoprolol succinate, carvedilol, or bisoprolol), either associated or not with amiodarone, a decision that should be tailored to the patients’ needs and shared with them.

11.1.6.3. Sustained Ventricular Tachycardia and Ventricular Fibrillation

Although the prevalence of sustained ventricular arrhythmias is not widely known, patients with CCCD, regardless of the ventricular function, can have monomorphic SVT, polymorphic SVT ( *torsades de pointes* ), and VF, which should be timely reversed in the emergency room. Amiodarone is the best drug option in cases of stable SVT and refractory or recurrent VF.

In the presence of hemodynamic instability, immediate electrical cardioversion is recommended, according to the protocol of Advanced Cardiovascular Life Support (ACLS). In case of immediate relapse (“electrical storm”), administration of antiarrhythmic drugs (preferably amiodarone) should be considered, with proper oxygen support, cardiac monitoring, and correction of possible electrolyte disorders.

According to the ACLS protocol, two drugs are indicated for the treatment of sustained ventricular arrhythmias in the emergency room, amiodarone and procainamide, both by the intravenous route. The dosages recommended are shown in Chart 11.1 .

**Chart 11.1:** – Treatment of sustained ventricular arrhythmias in the emergency room.

**AMIODARONE** (1 ampoule = 150mg)	**Stable SVT:** 1 ampoule IV for 10 minutes, can be repeated in case of no reversion. Intravenous administration of 1mg/min for 6 hours followed by 0.5mg/min IV in the subsequent 18 hours can be performed to stabilize ventricular arrhythmia. **Cardiac arrest due to refractory VF/pulseless VT:** 2 ampoules (300mg) IV in bolus after the third shock and 1 more ampoule (150mg) IV in case of no success after the fifth shock.
**PROCAINAMIDE** (1 ampoule = 500mg)	**Stable SVT:** Attack dose: 10-17mg/kg (20-50mg/min) IV Maintenance dose: 1-4mg/min IV

It is worth noting that the intravenous administration of amiodarone can cause phlebitis and arterial hypotension, reduce sinus rate, increase the duration of the QRS complex and of the QT interval, increase atrioventricular node refractoriness, reduce the HR (slow down) of the SVT, and improve the ICD defibrillation threshold.

Procainamide can increase the PR interval, the QRS complex duration, and the ICD defibrillation threshold. In addition, it can cause arterial hypotension and LVEF reduction, diarrhea and nausea, and trigger symptoms and signs of the lupus erythematosus syndrome.

Other antiarrhythmic drugs of intravenous administration, such as lidocaine, verapamil, beta-blockers (metoprolol, esmolol, or propranolol), and sotalol have low efficacy in reversing sustained ventricular tachyarrhythmias and should be avoided in CCCD or used only as secondary options in a few contexts.

Once sustained ventricular tachyarrhythmia is controlled or the cardiac arrest is reversed, the subsequent treatment is aimed at preventing relapse and, mainly, sudden death. Despite the high prevalence of CCCD in Latin America and the high fatality rate from those arrhythmias, there is no properly controlled RCT with proper sampling to finally clarify which is the best management to be adopted in each case. Regarding such management, some of the major options are the use of amiodarone, ICD implantation, catheter ablation, or an association of these therapies. The choice for the best option is based either on the results of observational studies or reports on CCCD records, which are very heterogeneous and conflicting, or still on data extrapolated from RCTs or guidelines on other cardiopathies, which have some inconsistencies and might not be applied to CCCD because of its peculiarities.

Thus, the strategy *“One-size fits all: what’s good for the gander is good for the goose”* , that is, ICD for all patients as the ideal therapy to prevent sudden death, seems not to be the most appropriate in CCCD. Obviously, when we talk about sudden death prevention, we are actually referring to all-cause death, because sometimes one cannot distinguish the exact mechanism of death (adjudication error might exist), and there is no use in a treatment that prevents sudden death without reducing total mortality, because it would be only modifying the mode of death.

It is worth noting that, as compared to ischemic and nonischemic heart diseases of other etiologies, patients with CCCD treated with ICD for secondary prevention tend to have higher LVEF, higher spontaneous ventricular arrhythmia density and complexity, higher number of both appropriate (shock and antitachycardia therapy) and inappropriate ICD therapies, shorter time for the first appropriate shock after implantation, most frequent electrical storms, and shorter survival free from ICD therapy, factors that can influence the choice of treatment. Regardless of the type of heart disease, LVEF is the major factor to determine the prognosis and selection of the most suitable therapy.

Because of its high antiarrhythmic efficacy, low incidence of proarrhythmia and of intolerable side effects, mainly when used at lower dosages, in addition to its good safety profile, even when administered to patients with ventricular dysfunction, amiodarone (introduced in Brazil more than four decades ago) is considered the first choice for the treatment of patients with CCCD and high-risk ventricular arrhythmias.

Rassi Jr *et al* .^352^were the first to study the impact of antiarrhythmic drug treatment on the evolution of patients with CCCD. They have analyzed the survival curve of 34 patients with monomorphic SVT empirically treated with amiodarone, isolated or in association with other antiarrhythmic drugs, and compared it with the curve of another cohort of 42 patients not treated or treated with procainamide or quinidine, the only drugs available then. Those authors have reported a significantly higher survival in the group treated with amiodarone. After 1, 4, and 8 years of follow-up, survivals were 87%, 65%, and 59%, respectively, for the group treated with amiodarone, and 57%, 22%, and 7%, respectively, for the group not treated or treated with those class I antiarrhythmics (p < 0.01).

Scanavacca *et al* .^709^have also reported long-term results with the empirical use of amiodarone in Brazil in a cohort of 35 patients with CCCD and sustained ventricular tachyarrhythmia (resuscitated from cardiac arrest = 8.5%; SVT with syncope or presyncope = 77%; SVT with well-tolerated tachycardic palpitations = 14.5%). The mean age was 50 years, mean LVEF was 41%, 68.5% of the patients were of the male sex, and 86% were in functional class I/II. After 27 months, the probabilities of SVT recurrence were 38%, 44%, and 56% in the 1-year, 2-year, and 3-year follow-up, respectively. The cardiac mortality rates were 4%, 11%, and 18%, and the sudden death rates were 0%, 4%, and 11% in the 1-year, 2-year, and 3-year follow-up, respectively. It is worth noting that all patients in functional class III or IV and with LVEF < 30% had SVT recurrence, and the cardiac mortality in that group was 80%. However, only 30% of the patients in NYHA functional class I/II and with LVEF > 30% had recurrence of SVT (p < 0.05) and none died. The mean dose of amiodarone, by the end of the study, was 356 mg/day and 15 (43%) patients reported side effects.

Leite *et al* .^481^have assessed the role of programmed ventricular stimulation to predict the long-term efficacy of class III antiarrhythmics. Those authors have studied 115 patients with CCCD and sustained ventricular tachyarrhythmia (recurrent SVT with syncope or presyncope = 54%; NSVT with syncope or presyncope and induction of SVT on EPS = 32%; and hemodynamically stable SVT with tolerable symptoms=14%). Mean age was 52 years, mean LVEF was 49%, 60% of the patients were of the male sex, and 83% were in functional class I/II. Based on the EPS results, after loading with amiodarone (78 patients) or sotalol (37 patients), the patients were divided into three groups: group 1 – no SVT induction (20%); group 2 – hemodynamically stable SVT induction (39%); and group 3 – hemodynamically unstable SVT induction (41%).

After a mean 52-month follow-up, total mortality was 39.1% (9%/year), significantly higher in group 3 than in groups 2 and 1 (69%, 22.2%, and 26%, respectively, p < 0.0001). The SVT recurrence was significantly smaller in group 1 than in groups 2 and 3 (39.1%, 62.2%, and 74.5%, respectively, p = 0.005). Thus, in patients with SVT and relatively preserved LVEF treated with class III antiarrhythmics, EPS apparently identifies those at lower risk of death who could remain on drug treatment. However, for those with worse prognosis, ICD could be an a more appropriate option based on those observations.^481^

Sarabanda *et al* .,^710^studying the predictors of mortality in 56 patients with CCCD and VT (SVT in 28 and NSVT in 28), have identified only LVEF as an independent marker of poor prognosis: LVEF < 40% increases the risk of death by 12 times (p = 0.0001). The most accurate cutoff points for sudden death and total death were LVEF of 40% and 38%, respectively. In the 28 patients with SVT, all empirically treated with amiodarone (when ICD implantation was not available), mean age was 54 years, mean LVEF was 42%, 64% were of the male sex, 100% were in functional class I/II, 43% had history of syncope, and the survival rates after 1 and 3 years of follow-up were 85% and 67%, respectively.

#### 11.1.7. Care During the Use of Amiodarone

Adverse effects with amiodarone include corneal microdeposits, sinus bradycardia, AVB, QT interval prolongation, dermatologic effects (photosensitivity and blue skin discoloration), thyroid dysfunction (hypothyroidism, most frequent, or hyperthyroidism), pulmonary toxicity, and, less commonly, hepatotoxicity. Late neurological side effects, such as tremors, paresthesia, and ataxia can occur.^711^

Pulmonary toxicity is the most severe and potentially fatal complication of amiodarone. Pulmonary impairment secondary to amiodarone manifests as interstitial pneumonia (most frequent) or eosinophilic pneumonia, organized pneumonitis, acute respiratory failure, or diffuse alveolar hemorrhage. The initial symptoms are dyspnea and dry cough, with or without fever. The chest X-ray shows diffuse or localized, reticular or consolidated opacities. Chest tomography shows interstitial impairment and bilateral diffuse opacities.

The first reports on pulmonary toxicity indicated a prevalence ranging from 5% to 15% when maintenance doses ≥ 400mg/day were regularly administered. Currently, with the reduction of the dose to 200mg/day, the incidence ranges from 1% to 5%. The most important risk factors for pulmonary toxicity are, in addition to the high daily doses of amiodarone (≥ 400mg), higher cumulative doses (long periods of administration), previous pulmonary disease, thoracic surgery, and pulmonary angiography.

In a meta-analysis with four studies involving a total of 1465 patients, there was no significant difference in the occurrence of pulmonary toxicity in patients receiving amiodarone at a low dose (defined as 150-330 mg/day) as compared to placebo.^712^

In another meta-analysis with 43 studies and 11 395 patients, the relative risk for adverse pulmonary events secondary to the use of amiodarone was 1.77, at doses ≥ 300 mg/day and clinical follow-up > 12 months. Doses lower than 300 mg/day were not associated with the increased incidence of pulmonary complications as compared to placebo.^713^

In another study, however, even doses lower than 200 mg/day were associated with an increase in pulmonary alterations. Thus, patients on amiodarone should receive the lowest effective dose and undergo clinical and laboratory monitoring periodically and systematically.^714^

The initial oral doses of amiodarone for individuals on outpatient care should range from 400 to 600mg/day, until the cumulative attack dose of 6-10 grams is achieved. For in-hospital patients, the attack doses can range from 400 to 1200 mg/day. Maintenance should be individualized, and the lowest effective dose determined.

Table 11.1 summarizes the recommendations for clinical and laboratory monitoring of patients on amiodarone.

**Table 11.1:** – Recommendations for clinical and laboratory monitoring of patients on amiodarone.

System/Organ	Test/examination	Follow-up	Adverse effects	Recommendation
Cardiovascular	ECG	Biannual	AVB, TdP	Reduce or stop
Dermatological	Ectoscopy	When necessary	Photosensitivity	Avoid sun exposure
Endocrine	Free T4 / TSH	Biannual	Hypo/Hyperthyroidism	Endocrine treatment
Liver	AST / ALT	Biannual	Elevation > 3x	Reduce or stop
Neurological	Physical examination	When necessary	Tremors and ataxia	Reduce or stop
Ophthalmic	Ophthalmic examination	When necessary	Corneal micro deposits Optical neuropathy	Ophthalmic guidance
Pulmonary	CXR / CT / CMDCT	In the presence of cough or dyspnea	Pulmonary toxicity	Suspend amiodarone Use corticosteroid

AVB: atrioventricular block; CMDCT: carbon monoxide diffusion capacity test; CXR: chest X-ray; CT: computed tomography of the lungs; TdP: torsades de pointes with increased QT interval.

#### 11.1.8. Prevention of Electrical Shock Recurrence in Patients Treated with Implantable Cardioverter-Defibrillator

In individuals with ICD, multiple shocks, either appropriate or not, and electrical storms are common in CCCD and affect the patient’s prognosis and quality of life. An observational study has reported the evolution of 89 patients with CCCD and ICD, most of whom due to secondary prevention, for a mean 12-month period.^715^In that short follow-up, 42% of the patients received appropriate shocks and 15.7% experienced electrical storms, numbers much higher than those of individuals with ICD and other heart diseases (appropriate shocks in 8.4% and 12.1% of the cases of primary and secondary prevention, respectively). The mean LVEF of patients with CCCD treated with an ICD was 40 ± 11%, indicating that a significant part of that subpopulation did not have severe ventricular systolic dysfunction.^715^

The association of amiodarone with beta-blockers (preferably propranolol, nadolol or atenolol, in case of satisfactory ventricular function, and metoprolol or carvedilol, in case of poor ventricular function) is considered to have the highest potential to reduce arrhythmic death and the number of both appropriate and inappropriate electrical shocks delivered by the ICD.^716^The OPTIC study, which has not included patients with CCCD, has shown the superiority of the association of amiodarone and beta-blockers to prevent shocks as compared to sotalol or any other beta-blocker used in isolation.^716^Empirically, that pharmacological association can be indicated to prevent the recurrence of shocks in patients with ICD and CCCD.

The recommendations for the pharmacological management of cardiac arrhythmias and sudden death prevention in CCCD are shown in Table 11.2 and Figure 11.1 .

**Table 11.2:** – Recommendations for pharmacological management of cardiac arrhythmias and prevention of sudden death in CCCD

Summary of recommendations	Recommendation grade	Level of evidence
Optimized treatment of HF according to the recommendations of this guideline for prevention of arrhythmias and sudden death in CCCD	Strong	B
Appropriate doses of ACEI, beta-blockers, and mineralocorticoid receptor antagonists for patients with HF and EF ≤ 40% to reduce total and sudden death	Strong	B
Association of beta-blockers, mineralocorticoid receptor antagonists, and sacubitril-valsartan in patients with HF and EF ≤ 40% to reduce total and sudden death	Conditional	B
Correction of hypopotassemia during treatment of ventricular arrhythmias	Strong	C
Beta-blockers (metoprolol succinate, carvedilol, or bisoprolol) either associated or not with amiodarone to treat patients with symptomatic NSVT and EF ≤ 40%	Strong	B
Beta-blockers (metoprolol succinate, carvedilol, or bisoprolol) either associated or not with amiodarone to treat patients with asymptomatic NSVT and EF ≤ 40%	Conditional	C
Beta-blockers, sotalol, propafenone, and amiodarone (refractory cases) in the treatment of very frequent asymptomatic VEs (> 16-20% of beats on 24-h Holter), the first 3 in the absence of AV conduction disorders, ventricular dysfunction, segmental changes, or myocardial fibrosis detected on CMRI	Conditional	C
Beta-blockers, sotalol, propafenone, and amiodarone (refractory cases) in the treatment of symptomatic VEs, the first 3 in the absence of AV conduction disorders, ventricular dysfunction, segmental changes, or myocardial fibrosis detected on CMRI	Strong	C
Amiodarone in the treatment of hemodynamically stable SVT with EF > 40%	Conditional	B
Amiodarone in the treatment of SVT with syncope and EF > 40%	Conditional	B
Amiodarone in the treatment of patients with syncope, monomorphic SVT induced on EPS, and EF > 40%	Conditional	B
Amiodarone in the treatment of spontaneous SVT or symptomatic NSVT with SVT induction on EPS and posterior non-induction of SVT after impregnation with antiarrhythmic drugs (amiodarone or sotalol)	Conditional	B
Amiodarone for patients with strong recommendation for ICD, but with limited life expectancy or no access to the device	Conditional	C
When used empirically, amiodarone should be administered at the lowest effective dose, and the patients should undergo periodical clinical and laboratory control for assessment of adverse effects	Strong	B
Patients with asymptomatic VEs and preserved left ventricular systolic function require no antiarrhythmic treatment	Strong	C

CCCD: chronic cardiomyopathy of Chagas disease; ACEI: angiotensin-converting-enzyme inhibitor; EF: ejection fraction; HF: heart failure; NSVT: nonsustained ventricular tachycardia; CMRI: cardiac magnetic resonance imaging; AV: atrioventricular; VE: ventricular extrasystole; SVT: sustained ventricular tachycardia; ICD: implantable cardioverter-defibrillator.

**Figure 11.1:** – Algorithm for the management of patients with CCCD and NSVT. CCCD: chronic cardiomyopathy of Chagas disease; NSVT: nonsustained ventricular tachycardia; LV: left ventricular; VE: ventricular extrasystole; BB: beta-blocker.

#### 11.1.9. Pharmacological Treatment of Atrial Fibrillation in Chronic Cardiomyopathy of Chagas Disease

Atrial fibrillation and HF frequently coexist. According to the Framingham Heart Study, approximately 40% of the patients with AF develop HF and vice-versa.^717^In HFrEF, the prevalence of AF increases with the functional class (NYHA) aggravation, ranging from 4.2% in class I to 49.8% in class IV.^718^The appearance of AF associates with increased all-cause mortality in patients with HF of any etiology, including CCCD.

The prevalence of AF in CCCD is increased as compared to that in the general population. A meta-analysis of 49 studies, including 34 023 patients, has revealed that the prevalence of AF in CCCD was twice that in the general population.^719^However, the prevalence of AF in CCCD does not seem to be higher than that in other structural cardiomyopathies.^720^

The pharmacological treatment of AF in CCCD is hindered by systolic biventricular dysfunction, as well as by electrical automatism and dromotropism disturbances. Thus, optimization of the HF therapy is mandatory, and the use of ACEI or ARB in HFrEF can reduce the incidence of AF.^721^

#### 11.1.10. Treatment in the Emergency Room

The initial management of patients admitted to the emergency room with AF of high ventricular response is to control HR and anticoagulation with proper medications. Then, indication for arrhythmia reversion should be assessed.

The ventricular rate of AF in patients with CCCD is often low, but, if hemodynamic instability with tachycardia occurs, the most appropriate management can be immediate anticoagulation, followed by electrical cardioversion. Symptomatic, but stable, patients with AF for less than 48 hours, without mural thrombi detectable on transesophageal ECHO, can undergo cardioversion with propafenone or amiodarone. Patients with AF duration ≥ 48 hours or unknown, or refractory AF history, should initially undergo anticoagulation and be medicated for HR control. Asymptomatic patients and/or with low HR and those with intense atrial dilatation usually should be only anticoagulated.^722^

#### 11.1.11. Outpatient Treatment

11.1.11.1. Reversion to Sinus Rhythm

The strategy of AF reversion is usually more appropriate when AF is of recent onset, occurs in younger patients, is very symptomatic, with little atrial dilatation and high ventricular response. When HF develops or worsens, rhythm reversion with amiodarone or even catheter ablation might be necessary.^723^Amiodarone can be especially indicated when, in addition to AF, patients with CCCD have ventricular arrhythmias, which is usually observed.

11.1.11.2. Heart Rate Control

The chronotropic control strategy, without reversion to sinus rhythm, is usually more indicated in the presence of long-duration AF or large dilatation of the chambers and in very old patients with multiple comorbidities and recurrence of arrhythmia. When beta-blockers are not sufficient to control ventricular response, the addition of digoxin can be considered. It is worth noting that calcium channel blockers are contraindicated in patients with HFrEF. Amiodarone can be occasionally used for chronotropic control when beta-blockers and calcium channel blockers are contraindicated, and catheter ablation is not possible.^722^

The recommendations for the pharmacological treatment of AF in CCCD are shown in Table 11.3 .

**Table 11.3:** – Pharmacological treatment of atrial fibrillation in CCCD

Summary of recommendations	Antiarrhythmic drugs	Recommendation grade	Level of evidence
Reversion of AF of recent onset in patients without evidence of structural or functional cardiac abnormalities	Propafenone (or other group I drugs)	Strong	B
Reversion of AF of recent onset but with structural and/or functional cardiac abnormalities	Amiodarone	Strong	B
Maintenaince of sinus rhythm after reversion of persistent AF or in paroxysmal AF in the absence of evidence of cardiac abnormalities	Propafenone (or other group I drugs)	Conditional	B
Maintenaince of sinus rhythm after reversion of persistent AF or in paroxysmal AF with structural and/or functional cardiac abnormalities	Amiodarone	Conditional	B
	Do not use propafenone (or other group I drugs)	Strong	B
Control of HR in AF with or without HF as long as there is no contraindication	Beta-blockers	Strong	B
Control of HR in symptomatic patients with high HR, who cannot undergo catheter ablation and to whom beta-blockers and calcium-channel blockers are contraindicated	Amiodarone	Conditional	B
	Digoxin	Strong	B

CCCD: chronic cardiomyopathy of Chagas disease; AF: atrial fibrillation, HR: heart rate; HF: heart failure.

## 11.2. Pacemaker, Cardioverter-Defibrillator, and Cardiac Resynchronization Therapy

### 11.2.1. Artificial Cardiac Pacemaker

The inflammatory processes, necrosis and fibrotic reaction, that follow the severe disorganization of myocardial architecture and structure in CCCD affect not only the contractile fibers, but also the autonomic nervous system and the tissue that generates and conducts the electrical impulse in the heart.^724 , 725^

Sinus node dysfunction occurs early in CCCD. The sinus node replacement with fibrotic reaction determines different expressions of the sinus node disease, its most frequent manifestation being sinus bradycardia. In addition, CCCD causes intraventricular blocks, of which isolated or LAFB-associated RBBB predominates.^724 , 726^All degrees of AVB are commonly found and can be asymptomatic or cause lipothymia, syncope, and even HF or sudden death.^727^According to the Brazilian Pacemaker Registry, CCCD is the first cause of AVB in Latin America, accounting for approximately 25% of PM indications.^728^

The prevalence of the PM use in patients with CCCD has been reported in a few cohorts, with rates ranging from 3.5% to 14.1%.^330^Sinus node disease and TAVB are the bradyarrhythmias most commonly treated with PM implantation in patients with CCCD.^729 , 730^The indication of PM implantation for patients with TAVB due to CD, since its beginning in the 1970s, can be considered as obeying the extreme plausibility principle. In fact, the evidence of the clear benefit of PM implantation consists solely in the observational study historically comparing the survival curves of 147 patients followed up before the PM appearance (survival of only 70%, 37%, and 6% after 1, 5, and 10 years of follow-up, respectively) with those of 74 patients followed up after the PM appearance (significantly higher survival of 86%, 57%, and 44% after 1, 5, and 10 years of follow-up, respectively, p < 0.05).^352^

Few studies have reported on the anthropometric and epidemiological characteristics or mortality predictors of patients with CCDC and a PM. A prospective cohort study published in 2018 included 396 patients with a PM followed up for, at least, 24 months. Their mean age was 62.5 ± 12.0 years, and most were of the female sex (64%). Approximately 95% of the patients were in NYHA functional class I or II. Approximately 75% had advanced AVB as the indication for PM implantation, and RV stimulation occurred in 82.2% of the cases. The annual mortality rate was 8.4%.^731^

It is worth noting the potential protective role of avoiding unnecessary ventricular stimulation and of considering the indication for direct stimulation of the conduction system, which is a more physiological modality, but that has not been properly tested in CCCD.^732 - 736^

In general, the indications for PM implantation in CCCD do not differ from the classical ones applied to heart diseases of other etiologies.^13 , 737^ Tables 11.4 , 11.5 , and 11.6 show the criteria used for PM implantation.

**Table 11.4:** – Indications for pacemaker implantation in CCCD: sinus node dysfunction

Summary of recommendations	Recommendation grade	Level of evidence
Spontaneous and irreversible SND with syncope, presyncope, dizziness, or HF clearly related to sinus bradycardia (< 40 bpm) or sinus pauses > 3.0s during vigil	Strong	C
SND induced by essential drugs with syncope, presyncope, dizziness, or HF clearly related to bradycardia (< 40 bpm) or sinus pauses > 3.0s during vigil	Strong	C
SND with symptoms of LCF clearly related to chronotropic incompetence	Strong	C
Bradycardia-tachycardia syndrome with no indication for catheter ablation or patient’s refusal	Strong	C
Sinus pause > 6.0s in a patient with LCF symptoms	Strong	C
Spontaneous and irreversible SND in patients with syncope, presyncope, or dizziness probably related to bradycardia, but whose association has not been clearly documented	Conditional	C
SND induced by essential drugs in patients with syncope, presyncope, or dizziness probably related to bradycardia, whose association has not been clearly documented	Conditional	C
Sinus bradyarrhythmia that triggers or aggravates HF, angina pectoris, or tachyarrhythmias	Conditional	C
Bradycardia (HR < 40 bpm) during vigil with mild symptoms, not definitely associated with bradyarrhythmia	Conditional	C
Patient with asymptomatic pause > 6.0s	Conditional	C
Asymptomatic SND or SND with symptoms proven not related to bradycardia – PM not indicated	Strong	C
Sinus bradycardia or sinus pauses due to the use of nonessential or replaceable drugs – PM not indicated	Strong	C
Sinus pauses or sinus bradyarrhythmia exclusively during sleep – PM not indicated	Strong	C

CCCD: chronic cardiomyopathy of Chagas disease; SND: sinus node dysfunction; LCF: low cerebral flow; HR: heart rate; HF: heart failure; PM: pacemaker.

**Table 11.5:** – Indications for pacemaker implantation in CCCD: atrioventricular blocks

Summary of recommendations	Recommendation grade	Level of evidence
TAVB, advanced AVB, 2^nd^degree AVB Mobitz II, of irreversible, permanent or intermittent cause, regardless of symptoms and QRS duration	Strong	C
2^nd^degree AVB Mobitz I, of irreversible, permanent or intermittent cause, with defined LCF symptoms consequent to bradycardia	Strong	C
Irreversible AF or atrial flutter with HR < 40 bpm, defined LCF symptoms consequent to bradycardia	Strong	C
TAVB or intra- or infra-his advanced AVB, induced by atrial stimulation or pharmacological test	Strong	C
AF or atrial flutter with mean HR < 40 bpm, during vigil, irreversible or consequent to essential drugs, in asymptomatic patients	Conditional	C
Irreversible 2^nd^degree AVB Mobitz I (up to conduction periods of 2:1) with indication of antiarrhythmics or beta-blocker, in asymptomatic patients	Conditional	C
TAVB, advanced AVB, intermittent and reversible or consequent to nonessential drugs – PM not indicated	Strong	C
1^st^degree AVB, 2nd degree AVB Mobitz I, and AVB 2:1, asymptomatic and supposedly AV nodal – PM not indicated	Strong	C

CCCD: chronic cardiomyopathy of Chagas disease; TAVB: total atrioventricular block; AVB: atrioventricular block; LCF: low cerebral flow; AF: atrial fibrillation; HR: heart rate; PM: pacemaker.

**Table 11.6:** – Indications for pacemaker implantation in CCCD: intraventricular blocks

Summary of recommendations	Recommendation grade	Level of evidence
Documented alternating bundle branch block, regardless of the presence of symptoms	Strong	C
IVB with HV interval > 70ms spontaneously on EPS in patients with syncope, presyncope, or dizziness of unknown etiology	Strong	C
IVB with HV interval > 100ms spontaneously on EPS in asymptomatic patients	Strong	C
Bifascicular blocks without documentation of intermittent TAVB in patients with syncope, presyncope, or repetitive dizziness of unknown etiology	Conditional	C
Bundle branch or bifascicular block with or without 1st degree AVB in asymptomatic patients – PM not indicated	Strong	C

CCCD: chronic cardiomyopathy of Chagas disease; IVB: intraventricular block; EPS: electrophysiological study; TAVB: total atrioventricular block; AVB: atrioventricular block; PM: pacemaker

### 11.2.2. Implantable Cardioverter-Defibrillator in CCCD

11.2.2.1. Primary Prevention of Sudden Cardiac Death

The success of primary prevention of sudden cardiac death relates to the identification of individuals at high risk for the event. The risk stratification of total mortality, which is mainly sudden in patients with CCCD, relies on a tool of simple and rapid use, the RASSI score,^408^already discussed in another chapter of this guideline.

Recently, relevant additional evidence of the role of myocardial fibrosis in the identification of high-risk individuals with CCCD has been presented. The quantification of myocardial fibrosis > 12.3g has been reported as an independent risk factor for the combined outcome of all-cause mortality, CTX, antitachycardia stimulation or appropriate ICD shock, and aborted sudden cardiac death.^424^The impact of this new factor is detailed in that same chapter of this guideline, in the general context of risk stratification and its relationship with the RASSI score.

The study of the correlation between the CCCD stages and the mortality causes has revealed that sudden cardiac death usually affects patients from the stage B on, being onward more relevant in stage C and a little less in stage D, in which refractory HF is the cause of most deaths. In general terms, the major mechanism of sudden death in CCCD is arrhythmogenic, and SVT (subsequent VF) accounts for most lethal events.^352^The structural abnormalities of CCCD, characterized by inflammation, cellular death, and reactive or reparative fibrosis, are the anatomic substrate more likely to trigger sudden cardiac death, because they create slow conduction areas and unidirectional blocks prone to the occurrence of electrical reentry. The VEs, frequent in CCCD, act as triggers of those circuits, resulting in VT/VF.^738^

The scientific evidence regarding primary prevention of sudden cardiac death in CCCD by using antiarrhythmic drugs (basically amiodarone) is scarce and has already been discussed. Regarding ICD, there is only the report of the findings of a series of 13 patients, not allowing conclusions on therapeutic efficacy.^739^

Although the role of programmed ventricular stimulation in risk stratification of patients with CCCD has not been well established, Silva *et al* .^480^have conducted a study with 78 patients with NSVT and syncope or presyncope (mean age of 46 years, mean LVEF of 47%, 58% of the male sex, and 85% in functional class I/II) with a mean follow-up of 56 months. Those authors have shown that the induction of monomorphic SVT in 25 patients (32%), all subsequently treated with amiodarone, was a predictor of spontaneous VT and of cardiac and total mortality.

In addition, as already reported in this guideline, Leite *et al* .^481^have shown that, in patients with NSVT and induced SVT (n = 37) or with spontaneous SVT (n = 78), the EPS could predict the long-term efficacy of class III antiarrhythmic drugs (mainly amiodarone). Immediately after oral loading with antiarrhythmic drugs, the induction of hemodynamically unstable SVT was related to higher total, cardiac, and sudden mortality when compared to patients in whom arrhythmia could not be induced or the arrhythmia induced was well-tolerated SVT.

These two studies, although observational, suggest that the EPS could identify patients with ventricular tachyarrhythmias, who, once treated with antiarrhythmic drugs, would evolve to worse prognosis and higher risk of death, and, in such cases, ICD could be a feasible alternative.

Briefly, so far, there is no scientific evidence supporting the use of ICD, with strong recommendation, for primary prevention of sudden cardiac death in CCCD. The ongoing CHAGASICS trial should soon provide relevant information.^460^It is a multicenter, open RCT designed to compare the effects of ICD with amiodarone for primary prevention of mortality in CCCD, in patients with NSVT on 24-hour Holter and RASSI score ≥ 10 points. The indications for ICD implantation in primary prevention of sudden cardiac death are shown in Table 11.7 .

**Table 11.7:** – Indications for ICD implantation in CCCD: primary prevention of sudden cardiac death

Summary of recommendations	Recommendation grade	Level of evidence
NSVT with syncope or presyncope of probable cardiac etiology and hemodynamically unstable SVT induction on EPS	Conditional	B
NSVT with SVT induction on EPS, followed by impregnation with amiodarone and EPS repetition, with hemodynamically unstable SVT induction on EPS	Conditional	**B**

CCCD: chronic cardiomyopathy of Chagas disease; ICD: implantable cardioverter-defibrillator; NSVT: nonsustained ventricular tachycardia; SVT: sustained ventricular tachycardia; EPS: electrophysiological study.

11.2.2.2. Secondary Prevention of Sudden Cardiac Death

The ICD is usually considered a resource to be used in some contexts of secondary prevention of sudden cardiac death for patients with CCCD. Its efficacy consists in the interruption of the life-threatening arrhythmic event by use of electric shock or rapid ventricular stimulation (antitachycardia), preventing the occurrence of cardiac arrest and subsequent death, although some arrhythmias aborted by the ICD could revert spontaneously, not necessarily culminating in death. The choice of this therapeutic option involves the thorough analysis of five essential factors: 1. duly documentation of the arrhythmic event causing the cardiac arrest (SVT or VF) and its correlation with irreversibility of the cause; 2. conviction that less invasive clinical therapy and/or procedures, of similar efficacy, have failed; 3. certainty that the underlying heart disease is being fully treated; 4. appreciation of the underlying cardiomyopathy risk stratification; and 5. patient’s clinical condition, expressed mainly by the ventricular dysfunction (LVEF) grade and the arrhythmia-related symptom type.

These factors have received little attention in studies of secondary prevention of sudden cardiac death in CCCD. There is no RCT in that population and the scientific evidence is limited to data from records from manufacturers of implantable devices,^740 , 741^single-center observational clinical studies assessing small population samples,^355 , 742 - 750^and meta-analyses of those studies.^751 , 752^

The largest cohort of patients with CCCD treated with ICD implantation for secondary prevention in a single center has enrolled 116 consecutive patients, 62% of the male sex, and mean age of 54 years. The mean LVEF was 42%, 83% of the patients were in NYHA functional class I/II, and the reason for ICD implantation was resuscitation from cardiac arrest in 18% and symptomatic SVT in 82% of the cases. In a mean 45-month follow-up, the following was reported: annual total mortality rate of 7.1%; appropriate therapies in 50% and inappropriate in 11% of the population. The independent factors of worse prognosis were NYHA functional class III and low LVEF. Patients with RV stimulation rate over 40% also had shorter survival.^746^

On the other hand, in a retrospective cohort of 90 consecutive patients with CCCD (68% of the male sex, mean age of 59 years, and mean LVEF of 47%) treated with ICD implantation, 30% of whom had preserved cardiac function, in a mean 756-day follow-up, the annual mortality rate was high (16.1%). Of the patients who died, 88% were in functional class I at the time of ICD implantation. Although 65% of the patients experienced appropriate shock and antitachycardia therapy, the monthly rate of shocks was the only independent predictor of mortality.^743^

The mortality rate of another retrospective cohort of 76 patients with CCCD and ICD has been compared to that of a historical series of 28 patients with SVT treated only with amiodarone.^747^A 72% reduction in total mortality and a 95% reduction in sudden cardiac death were reported in the cohort treated with ICD. However, when subgroup analysis was performed, there was a significant interaction between LVEF and benefit from ICD. While patients with reduced LVEF (< 40%) had a significant and expressive benefit with ICD, those with relatively preserved LVEF (≥ 40%) had little or no benefit.^747^

These data are consistent with the results of a meta-analysis of RCTs of secondary prevention in other heart diseases (AVID, CIDS, and CASH), which showed a reduction in total and sudden mortality with ICD (as compared to amiodarone) only in patients with LVEF < 35%.^701^

It is worth emphasizing that a meta-analysis including that study and other five observational studies in CCCD has shown no difference in total mortality between the use of amiodarone (9.6%/year) and ICD (9.7%/year).^751^

Recently, a systematic review and meta-analysis has been published including 13 observational studies on CCCD to reassess the global efficacy of ICD to prevent total and sudden death. It included 1041 patients, 92% of them in secondary prevention and only 8% in primary prevention, with mean age of 57 years, 64% of the male sex, mean LVEF of 38%, 79% in functional class I/II, 79% on amiodarone, and 44% on beta-blockers. In a 2.8-year follow-up, total mortality rate was 9.0% per year, and, in a 2.6-year follow-up, sudden cardiac death rate was 2.0% per year. Appropriate ICD therapies (shocks or antitachycardia interventions) occurred in 24.8% of the patients, annually. High rates of inappropriate shocks (4.7%/year) and arrhythmic storms (9.1%/year) have also been observed.^752^

Regarding the prognosis of the arrhythmia types that usually indicate the need for ICD implantation, Lima *et al* .^753^have compared the clinical evolution of two groups of patients: group 1, with 318 patients, 36% of whom had CCCD, whose cause of ICD implantation was symptomatic SVT (syncope and/or hemodynamic instability) or SVT induction on EPS in patients with recurrent syncope of unknown etiology; and group 2, with 97 patients, 15% of whom had CCCD, whose cause of ICD implantation was resuscitation from cardiac arrest due to VF or pulseless SVT. While sex (male: 75% *versus* 73%) and NYHA functional class I/II (77% *versus* 76%) did not differ between groups 1 and 2, the mean age was higher (57 *versus* 51 years, p = 0.0004) and the mean LVEF was lower (38% *versus* 43%, p = 002) in group 1. After a mean follow-up of 24 months for group 1 and of 26 months for group 2, mortality was higher in group 2 (24.7% *versus* 13.5%, p < 0.005), and both groups experienced appropriate ICD shocks similarly (31% of group 1 patients *versus* 26% of group 2 patients, p = 0.09), which might indicate higher arrhythmia severity in the subgroup of patients resuscitated from cardiac arrest.

In another study, Leite *et al* .^754^have assessed the impact of syncope on total and cardiac mortality of 78 patients with monomorphic SVT (mean age of 53 years, mean LVEF of 50%, 58% were male patients, 88% were in functional class I/II). Syncope during SVT was observed in 45 patients (58%), but not in 33 patients (42%). After a mean 49-month follow-up, neither total (33% *versus* 39%) and cardiac mortality (27% *versus* 30%), nor nonfatal SVT recurrence (58% *versus* 54%) differed between patients with and without syncope, respectively. However, the presence of syncope during recurrences was significantly higher among patients who had that symptom initially (65% *versus* 18%, p < 0.01). Thus, in CCCD, syncope during monomorphic SVT seems not to be associated with an increase in total and cardiac mortality.

Given the set of results summarized in the previous paragraphs, one can conclude that more scientifically based evidence is still necessary to support the use of ICD for secondary prevention of sudden cardiac death in patients with CCCD. This should ideally be solved with the conduction of an RCT. However, several researchers claim to have ethical concerns about adopting that scientific path, and currently there is no perspective on that.

Others have claimed that there is large positive experience built up over the years with the use of protocols supported by international and national guidelines for patients with ICM or DCM treated with ICD implantation. This created a scenario favorable to the extrapolation of those rules to clinical practice, so that patients with CCCD would be more liberally treated with ICD. However, secondary prevention with ICD implantation in CCCD should be always supported by a thoughtful individualized decision-making with risk/benefit analysis.

That general principle derives from two essential notions. The first is that even in scenarios supported by international guidelines for patients with other cardiopathies, the benefit of ICD is relatively restricted to the existence of severe ventricular systolic dysfunction, being less significant in the absence of that factor. The other one, already mentioned, is that the complex and peculiar pathophysiology of CCCD implies that therapeutic principles only partially validated for other cardiopathies can hardly be properly extrapolated to CCCD. Therefore, both LVEF, considering the ideal cutoff point of 40%, and the type of arrhythmia and symptom associated have been used to better support the indications of ICD for secondary prevention of sudden cardiac death.

Of note, by the time this guideline was being finished, a recent publication of the European Society of Cardiology^755^for the treatment of ventricular arrhythmias explicitly mentioned CCCD and restricted the indications for ICD in that context, very similarly to our recommendations. Table 11.8 and Figure 11.2 show the recommendations of this guideline for ICD implantation in CCCD.

**Table 11.8:** – Indications for ICD implantation in CCCD: secondary prevention of sudden cardiac death

Summary of recommendations	Recommendation grade	Level of evidence
Resuscitated cardiac arrest due to VF or documented pulseless SVT (excluding the reversible causes, and life expectancy < 1 year), regardless of EF	Strong	B
Hemodynamically unstable SVT (low output), regardless of EF	Strong	B
SVT with syncope and EF ≤ 40%	Strong	B
Syncope with monomorphic SVT induced on EPS and EF ≤ 40%	Strong	B
Hemodynamically stable SVT and EF ≤ 40%	Strong	B
SVT with syncope and EF > 40%	Conditional	B
Syncope with monomorphic SVT induced on EPS and EF > 40%	Conditional	B
Hemodynamically stable SVT and EF > 40%	Conditional	B

CCCD: chronic cardiomyopathy of Chagas disease; ICD: implantable cardioverter-defibrillator; VF: ventricular fibrillation; SVT: sustained ventricular tachycardia; EF: ejection fraction; EPS: electrophysiological study.

**Figure 11.2:** – Algorithm for the management of patients with CCCD and sustained ventricular tachyarrhythmias. CCCD: chronic cardiomyopathy of Chagas disease; ICD: implantable cardioverter-defibrillator; CA: cardiac arrest; VF: ventricular fibrillation; VT: ventricular tachycardia; SVT: sustained ventricular tachycardia; EPS: electrophysiological study; TTE: transthoracic echocardiography; LVEF: left ventricular ejection fraction; AA: antiarrhythmic.

### 11.2.3. Cardiac Resynchronization Therapy

There are no solid data from RCTs to support the use of cardiac resynchronization therapy (CRT) in CCCD. The CRT has been recommended to patients with DCM and ICM, presenting with advanced HF, severe systolic dysfunction, and ventricular dyssynchrony, mainly represented by a widened QRS complex. The CRT has been described as acting positively on LV remodeling, promoting a significant reduction in the functional class of HF, and improving quality of life, based on several other functional parameters.^756 - 760^

Some studies have evidenced a benefit of CRT regarding HF mortality reduction,^756 , 761 , 762^especially in the presence of LBBB, LVEF ≤ 35%, QRS duration ≥ 130ms, and mitral regurgitation.^763 , 764^However, in CCCD the prevalence of LBBB is low, which limits the formal indication of CRT in that scenario. The presence and extension of myocardial fibrosis, which is associated with a worse prognosis regardless of LVEF,^424 , 428 , 765^as well as frequent ventricular arrhythmias, tricuspid regurgitation, and RV dysfunction are examples of other factors that do not favor CRT in CCCD and that can increase the patients’ risk of nonresponse.

Importantly, the implantation of the conventional (single-chamber) PM, often used in CCCD, causes LV dyssynchrony (“induced LBBB”) mainly when the lead is placed in the RV apical region. This is associated with hemodynamic impairment and worsens the prognosis of patient with HF treated with PM.^766 , 767^

So far, only five single-center, observational studies^768 - 772^have assessed the clinical course of patients with CCCD undergoing CRT, and three of them^770 - 772^have compared the effect of that therapy in CCCD with that in other cardiopathies ( Table 11.9 ). A statistically significant reduction in LVDD and a significant improvement in NYHA functional class and LVEF were observed with CRT in two studies including only patients with CCCD.^768 , 769^Annual all-cause mortality in those two studies ranged from 9.0% to 9.2%. In other three studies,^770 - 772^the survival of patients with CCCD was significantly lower and the percentage of nonresponders to CRT was significantly higher as compared to that of patients with other cardiomyopathies. In patients undergoing CRT, the presence of CCCD increased the risk of death by approximately two to four times.^770 , 772^

**Table 11.9:** – Observational studies of cardiac resynchronization therapy in CCCD.

CHARACTERISTICS	Araujo et al. 2014^**55**^	Menezes et al. 2018^**56**^	Martinelli et al. 2018^**57**^			Scorzini et al. 2018^**58**^			Passos et al. 2019^**59**^	
Population	CCCD	CCCD	CCCD	ICM	DCM	CCCD	ICM	Others	CCCD	Non- CCCD
N of patients	72	50	115	134	177	42	13	43	13	41
Male sex (%)	NA	56	65	83	51	59.5	92	56	31	66
Mean age (years)	NA	63	57	68	60	60	66	58	65	62
Types of block:										
• Induced LBBB (%)	15	30	74	31	17	21	0	5	NA	NA
• Spontaneous LBBB (%)	47	30	11	63	78.5	39	92	87	NA	NA
• Not LBBB (%)	38	40	15	7	4.5	39	8	8	NA	NA
CRT-ICD (%)	NA	74	23.5	33	26	31	31	26	0	0
Atrial fibrillation or flutter (%)	0	16	25	16	15	14	15	14	0	0
Functional class III/IV (%)										
• Pre-implantation	100	82	82	82	88	87.5	67	80	77	63
• Post-implantation	13	18	43.5	26	26	50	33	24	NA	NA
Mean LVEF (%)										
• Pre-implantation	27	29	26	26	24	26	27	24	27	26
• Post-implantation	44	39	27	28	29	26	34	30	NA	NA
Mean QRS length (ms)										
• Pre-implantation	148	150	163	164	162.5	161	154	160	NA	NA
• Post-implantation	NA	116	NA	NA	NA	139	134	135	NA	NA
LVEDD (mm)										
• Pre-implantation	66	NA	66	69	74	68	68	73	NA	NA
• Post-implantation	65	NA	68	68	71	65	65	69	NA	NA
Mean follow-up (months)	47	61	29	29	29	27	42	35	15	15
Nonresponders (%)	33	34	43.5	26	26	47	33	35	NA	NA
Annual mortality (%)	9.0	9.2	25.4	11.3	10.4	25.6	4.8	13.9	18.4	3.2

CCCD: chronic cardiomyopathy of Chagas disease; ICM: ischemic cardiomyopathy; DCM: dilated cardiomyopathy; LBBB: complete left bundle branch block; CRT-ICD: cardiac resynchronization therapy associated with implantable cardioverter-defibrillator; LVEF: left ventricular ejection fraction; LVEDD: left ventricular end-diastolic diameter; NA: not available.

Although plausible, the use of CRT as a PM upgrade remains controversial. While the COMBAT study, whose sample included 51.6% of patients with CCCD, has reported a significant improvement in the quality of life and an increase in LVEF of patients undergoing CRT as compared to simple RV stimulation,^773^the RAFT study, which included no patient with CCCD, has shown no benefit regarding mortality when 54 patients received CRT as compared to other 81 patients treated only with RV stimulation.^762^

A small RCT including 50 patients has reported a benefit in terms of quality of life when CRT was added to isolated RV simulation in patients mandatorily treated with PM implantation.^774^

Briefly, there is no specific and consistent scientific evidence to support the indication for CRT in CCCD, which will only be possible with the conduction of an RCT comparing CRT associated with optimized clinical treatment *versus* a control group with only optimized drug treatment. Thus, currently CRT should have its application derived from extrapolation from studies performed in patients with ICM and DCM with careful selection and individualization based on risk/benefit analysis for the patient with CCCD ( Table 11.10 ).

**Table 11.10:** – Indication for CRT in CCCD

Summary of recommendations	Recommendation grade	Level of evidence
Symptomatic HF, functional class II and III, with LVEF ≤ 35%, sinus rhythm, LBBB morphology, and QRS duration ≥ 130ms, despite optimized therapy to reduce morbidity and mortality	Conditional	B

CCCD: chronic cardiomyopathy of Chagas disease; CRT: cardiac resynchronization therapy; HF: heart failure; LVEF: left ventricular ejection fraction; LBBB: left bundle branch block.

## 11.3. Ablation Methods

### 11.3.1. Sustained Ventricular Tachycardia: Clinical Presentation, Electrophysiological Mechanisms, and Sites of Origin

The clinical manifestations of ventricular arrhythmias in CCCD are heterogeneous, ranging from asymptomatic or slightly symptomatic to disabling tachycardia with syncope, poorly tolerated ICD shocks, electrical storm, and even sudden death.^46 , 354 , 442 , 747 , 752 , 775^As already mentioned in other chapters of this guideline, although the coexistence of arrhythmias with HF findings is the most common manifestation, the occurrence of severe arrhythmias as the initial or predominant manifestation without HF is also a hallmark of CCCD.^353^

Several pathogenic mechanisms (such as myocardial injury directly caused by the parasite or immune mediated, autonomic denervation, and microcirculation disorders) cause myocardial damage and varied disorders at all levels of cardiac electrical impulse generation and conduction.^176 , 270 , 326^

The fundamental electrophysiological mechanism of SVT in CCCD is usually the electrical stimulus reentry in the ventricular scar, constituted by extensive interstitial fibrosis intermingled with viable myocardial fibers. This is more frequent in the LV inferolateral region (70% of the patients), but can also occur in the LV apical region or in the right ventricle.^776 - 779^These areas of fibrosis (scars) can be located in the LV subendocardial, intramyocardial, or subepicardial regions.^424 , 428 , 778 - 781^In addition, an isthmus of viable myocardium between the mitral annulus and a scar in the LV inferolateral region can form a macroreentrant circuit of SVT.^782^Finally, a macroreentrant circuit involving the right and left branches (bundle branch reentry) can be the least common cause of SVT.^783^

In general, the different reentrant mechanisms of SVT have been thoroughly investigated by use of invasive EPS, in which the programmed ventricular stimulation can reproduce that arrhythmia in more than 80% of the patients with clinical history of SVT or syncope and CCCD. In addition, endocardial and/or epicardial mapping has shown abnormal diastolic, presystolic, and mid-diastolic electrograms, predominating in the regions of LV akinesia and dyskinesia.^481 , 776 , 778 , 780 , 781^

During EPS using ventricular stimulation techniques (concealed entrainment), the critical isthmus of the reentry circuit can be differentiated from the other regions not involved in the VT mechanism, which can be confirmed by the interruption of VT during radiofrequency ablation.^481 , 776 , 778 , 780 , 781^In addition to fibrosis in circumscribed ventricular wall regions, the intracardiac autonomic nervous system injury, characterized by ganglionic neuronal depletion and cardiac dysautonomia, and the chronic myocardial inflammation are pathophysiological changes that can contribute to myocardial electrical instability and genesis of ventricular tachyarrhythmias.^193 , 208 , 222 , 784 - 788^

### 11.3.2. Clinical and Laboratory Pre-Ablation Assessment

Patients with CCCD and SVT usually have advanced heart disease^430^and HF (which should have its specific treatment optimized), requiring, in order to program ablation, the assessment of kidney function, the presence of infection, and the need for vasoactive drugs in cases of electrical storm. Usually, the presence of comorbidities should not contraindicate ablation, mainly in cases of electrical storm and recurrent shocks, because, without the intervention, mortality is very high.^743^

The *PAINESD* (chronic obstructive **P** ulmonary disease, **A** ge > 60 years, **I** schemic cardiomyopathy, **N** YHA III or IV, **E** jection fraction < 25%, VT **S** torm and **D** iabetes mellitus) risk score has been developed to identify patients that might have hemodynamic decompensation during VT ablation^789^and higher post-procedure early mortality. Because the original cohort included patients with ischemic and nonischemic cardiopathies, except CCCD, it cannot be used to predict 30-day mortality after VT ablation.^790 , 791^

In addition, patients with CCCD can have megaesophagus and/or megacolon.^792 , 793^The approach for ablation should be preferably epicardial;^794^however, in the presence of megacolon, the access to the pericardial space must be obtained through a surgical window or laparoscopy-guided puncture.^795^

Late gadolinium enhancement on CMRI is useful to identify areas of myocardial fibrosis^796^and to assess whether the target substrate is located on the epicardial or endocardial surface.^797^Coronary computed tomography angiography can identify the areas of thinning^798^and hypoperfusion, which are associated with the arrhythmia substrate. Both CMRI and coronary computed tomography angiography assess the local epicardial fat thickness and location of the coronary arteries, allowing integration with the electroanatomic mapping systems.^799^

Recently, some software for processing 3D images from CMRI have been developed, allowing the definition of potential circuits of arrhythmia.^800^These images enable integration with electroanatomic mapping systems and contribute to a successful ablation,^801^which becomes faster and more effective, thus eliminating the need for electroanatomic mapping reconstruction.^802^

Another important point in the ablation planning is the 12-lead ECG assessment during clinical VT, which, whenever possible, should be recorded. This allows comparison with procedure-induced VTs, being important in the search for elimination of clinical VT, given that patients with CCCD usually have multiple morphologies of VT.^781^This is a valid concept although ECG has limitations to define epicardial VT.^803^

Frequently, patients with CCCD have recurrences after VT ablation, requiring multiple procedures to be performed. Information on the previous procedures is fundamental to plan the new ablation. It is worth noting the following: the maps of previous procedures should be assessed and compared to the current one; the presence of an endo- or epicardial scarring area not approached in the previous procedure should be assessed; and information on bleeding during the previous epicardial access should be obtained, because, if affirmative, epicardial adherence can occur.

### 11.3.3. Mapping Techniques for Ventricular Tachycardias

The recurrence rates of VT episodes in individuals with CCCD are high, even after optimized drug therapy. A recent meta-analysis has reported rates of appropriate therapies and electrical storm of 9% and 25% per year, respectively, in patients with ICD for secondary prophylaxis.^752^Thus, radiofrequency ablation is indicated in many cases refractory to clinical treatment.^794^

A myocardial scar that propitiates reentry and SVT is usually located in the basal portions of the inferior and lateral LV walls, and mid-myocardial and epicardial impairment is frequent. Thus, the initial results of VT ablation with endocardial approach are frustrating with success rates around 17%.^777 , 804^

Epicardial access by use of percutaneous subxiphoid puncture with fluoroscope-guided Tuohy needle was described in 1996^804^and has contributed to optimize the results of VT ablations in patients with CCCD. In an RCT, the combined endocardial/epicardial approach, as compared to the exclusively endocardial one, showed a lower rate of recurrence, 40% and 80%, respectively, in a 2-year follow-up.^781^

The most feared complication related to percutaneous epicardial access is bleeding, which can occur in approximately 10% of the cases. Most bleedings are small and related to accidental RV puncture. Massive bleeding requiring surgical approach occurs in 2% of the cases. Hepatic and intestinal lesions can occur during epicardial puncture in the presence of significant hepatomegaly and megacolon. In these cases, pericardial access through either surgery or videolaparoscopy-guided subxiphoid puncture can be chosen.^795^

In recent years, the following variations of the original epicardial puncture technique have appeared: micropuncture;^805^insufflation of carbon dioxide (CO_2_) in the right atrial appendage^806^or coronary sinus;^807^needle embedded with a real-time pressure monitor;^808^computed tomography;^809^CMRI;^810^and puncture guided by electroanatomic mapping.^811^Of these, it is worth noting that, in a multicenter observational study, micropuncture has shown lower rates of massive pericardial effusion and of need for surgical correction of bleeding, as compared to the puncture technique with a high caliber needle.^805^

Some situations can limit the efficacy of ablation on the epicardial surface, such as an ablation target region located under the epicardial fat or close to the trajectory of the phrenic nerve or coronary arteries.^812^

Because of disease severity and procedural complexity, perioperative care is important to reduce the risk of complications. The search for previous intracavitary thrombi is mandatory, while invasive blood pressure monitoring, infusion of vasoactive drugs prior to anesthesia induction, and mechanical circulatory support in selected cases are useful to perioperative hemodynamic optimization.

Catheter ablation can be performed with the patient in VT or sinus rhythm. Each strategy has advantages and disadvantages and there is no study comparing the results in a population with CCCD. Although the procedure performed in a patient in VT favors the identification of tachycardia isthmuses more accurately, most induced VTs are poorly hemodynamically tolerated and warrant immediate electrical cardioversion.

However, even in hemodynamically stable VTs, the mapping time should be maximally shortened due to the risk of low cardiac output post intervention. Catheter ablation in a patient in sinus rhythm is aimed at modifying the substrate, which consists in the identification and elimination of possible tachycardia isthmuses. These areas are related to myocardial scarring, which is identified as a low-voltage region in the electroanatomic mapping system and represented by late, fragmented, and low-amplitude potentials. Although less specific, this technique has the advantage of maintaining the patient hemodynamically stable for a longer period during the procedure, when there is severe ventricular dysfunction.^813 , 814^

Of note the technological evolution of the electroanatomic mapping system, mainly the high-definition mapping catheters, significantly increased the accuracy of the anatomical definition of scar regions, in addition to its functional correlation with electrical propagation. However, studies related to VT ablation in patients with CCCD are scarce and practically do not contemplate the currently available technology.

### 11.3.4. Outcomes and Complications During Ventricular Tachycardia Ablation

Historically, programmed ventricular stimulation has been used as the major tool to assess the immediate effectivity of the VT ablation procedure.^815^However, varied definitions of non-inducibility (heterogeneous stimulation protocols and relevance of rapid or “nonclinical” VT induction) associated with the spontaneous daily variation in the results of programmed ventricular stimulation represent relevant limitations and deficiencies in the accuracy of this tool to predict the short- and long-term success of ablation.^816^

Despite those limitations, programmed ventricular stimulation at the end of the procedure remains the major tool to assess the immediate ablation success.^815^Patients who remain with slow VT (cycle > 300ms) induced at the end of the procedure more often have recurrence than those without induced VT.^817^Other strategies to assess the procedure’s result during ablation include verifying the elimination of excitability,^818^late potentials,^819 , 820^local abnormal ventricular activities (LAVA),^821^and scar channels,^822^in addition to checking substrate homogenization,^823^central isolation of the scar,^824 , 825^and lesion by use of imaging guidance.^826 , 827^

The acute complications include the vascular ones, pericardial effusion, cardiac tamponade, electromechanical dissociation, TAVB, phrenic nerve paralysis, stroke, and death.^781 , 828^

### 11.3.5. Ablation Results and Patients’ Follow-up

Recently, a prospective RCT on VT ablation in a small group of patients with CCCD has reported the superiority of the systematic epicardial/endocardial approach over the exclusively endocardial one, with 40% VT recurrence in a mean 19-month follow-up in the first group.^781^This recurrence rate was similar to that of VT ablation in patients with nonischemic cardiopathies in general.^828^

Post-ablation VT recurrence depends on several factors, the most common being related to the use of antiarrhythmic drugs, programming of implantable cardiac devices, and cardiomyopathy severity.^815 , 829^All available means for the detection of SVT episodes should be used, including an ICD monitoring zone, capable of detecting slow SVT induced during ablation. In addition to recurrence of any SVT, the follow-up should record the density of arrhythmias, occurrence of electrical storm, hospitalizations, and cardiac and noncardiac death.

Tables 11.11 and 11.12 show the recommendations and levels of evidence for the indication of SVT catheter ablation, as well as the methods used during the procedure in patients with CCCD.

**Table 11.11:** – Indications for catheter ablation of SVT in CCCD

Summary of recommendations	Recommendation grade	Level of evidence
Recurrent or incessant monomorphic SVT, refractory to treatment with antiarrhythmic drugs	Strong	B
Patient with ICD and recurrent monomorphic SVT triggering multiple shocks, despite treatment with antiarrhythmic drugs and ICD reprogramming	Strong	B
Patient with ICD and recurrent monomorphic SVT triggering multiple shocks, when treatment with antiarrhythmic drugs is contraindicated or poorly tolerated, despite ICD reprogramming	Strong	C
Monomorphic SVT induced on EPS to elucidate syncope	Conditional	C
Patient with ICD and first episode of spontaneous monomorphic SVT documented on ECG or interrupted by ICD	Conditional	C
Recurrent monomorphic SVT, without previous use of amiodarone	Conditional	C
Poorly tolerated monomorphic SVT induced on EPS to elucidate syncope	Conditional	C
Combined endocardial/epicardial ablation strategy is preferable to isolated endocardial estrategy to prevent VT recurrence	Strong	B
Ablation is contraindicated in cases of polymorphic SVT or VF secondary to severe metabolic disorders or to proarrhythmic drugs	Strong	C

CCCD: chronic cardiomyopathy of Chagas disease; SVT: sustained ventricular tachycardia; ICD: implantable cardioverter-defibrillator; EPS: electrophysiological study; VT: ventricular tachycardia; VF: ventricular fibrillation.

**Table 11.12:** – Methods used for catheter ablation of SVT in CCCD

Summary of recommendations	Recommendation grade	Level of evidence
SVT recording on 12-lead ECG before ablation, whenever the hemodynamic conditions allow	Strong	C
Clinical and laboratory assessment and by use of ECG, chest X-ray, and transthoracic ECHO (transesophageal in patients with AF)	Strong	C
Late gadolinium enhancement on CMRI before ablation	Conditional	C
CT coronary angiography, and chest and upper abdomen CT (identification of megacolon)	Conditional	C
Reserve operation room, blood bank, and intensive care unit recovery when considering epicardial approach	Strong	B
Coronary angiography when CT coronary angiography cannot be perfomed	Conditional	C
Invasive hemodynamic monitoring during SVT ablation	Strong	C
ICD reprogramming before and at the end of the procedure	Strong	B
General anesthesia	Conditional	C
Systemic anticoagulation after access to the left chambers, mantaining ACT > 350s during LV endocardial ablation	Strong	B
Electrophysiological assessment with programmed ventricular stimulation before and after ablation	Conditional	B
Epicardial access and mapping if endocardial ablation fails	Strong	B
Epicardial access and mapping after recurrence of endocardial ablation	Conditional	B
Mapping and endocardial and epicardial ablation in the first procedure	Conditional	B
Exclusive epicardial mapping and ablation in the first procedure or after unsuccessful endocardial ablation	Conditional	C
Limit the procedure to a maximum of 6 hours, except if the patient persists in incessant VT	Conditional	B
Clinical and laboratory assessment and by use of ECG and ECHO 24 hours after the procedure	Strong	B

CCCD: chronic cardiomyopathy of Chagas disease; SVT: sustained ventricular tachycardia; ECG: electrocardiogram; ECHO: echocardiogram; AF: atrial fibrillation; CMRI: cardiac magnetic resonance imaging; CT: computed tomography; ICD: implantable cardioverter-defibrillator; ACT: activated clotting time; LV: left ventricular; VT: ventricular tachycardia.

## 12. Managements to Prevent and Treat Thromboembolic Complications

### 12.1. Introduction

Thromboembolic complications represent a heterogeneous group of clinical manifestations associated with CCCD, corresponding to one of its three essential mechanisms of death alongside HF and sudden death.^1^In most cases, mortality due to thromboembolic phenomena is related to cerebral and pulmonary embolisms. Considering that neurologic events are usually the most expressive clinical manifestations, the cerebral thromboembolic complications are most frequently detected in medical practice.^830^

Cardioembolic stroke can be the first clinical manifestation of CCCD, occur even in early stages of the disease, affect individuals of several age groups, and new episodes can frequently occur when secondary prophylaxis is not established. The clinical manifestations are usually due to embolism of intracardiac thrombi that, because of their dimensions, have a high potential of obstructing the proximal circulation in the central nervous system, being usually associated with severe and disabling neurological sequelae if not leading directly to death.^1^

### 12.2. Epidemiology of Thromboembolic Events

Postmortem studies have shown a variable frequency of cardiac thrombosis in CD, with prevalence ranging from 27% to 79% and a slight predominance of impairment of the right chambers over the left ones (22% to 54% versus 21% to 46%, respectively).^831 - 834^In those studies, thromboembolic phenomena were more common in the systemic circulation and caused relatively more deaths due to pulmonary embolism.^831^The incidence of cardiac thrombi was higher in the HF clinical syndrome (36%) than in cases of sudden death (15%), with relationship to neither age nor sex.

Endocardial inflammatory lesions and intracavitary blood stasis are considered important factors in the pathogenesis of parietal thrombosis in the heart, related to the occurrence of multiple thromboembolic phenomena and high risk of death due to embolism.^831^In addition, apical aneurysm is a relevant factor, being present in 53.2% of 148 autopsies, of which 36.8% would be complicated by localized thrombosis, while only 11.1% of the hearts without apical aneurysm had intracavitary thrombi.^835^Another study, involving 1153 autopsies, identified the presence of apical aneurysm in 52% of the cases,^836^predominating in the male sex.

In a prospective observational study of 55 patients with CCCD and apical aneurysm assessed by use of cine ventriculography, Albanesi Filho *et al* .^837^have reported the presence of intraventricular thrombi in only 14.5% of the cases. The low frequency of thrombi described in that study could be attributed to the lower sensitivity of the assessment method as compared to postmortem studies, which, in addition, were probably performed in more advanced phases of the disease.

### 12.3. Risk Factors and Mortality

The presence of severe myocardial dysfunction, LV apical lesion, intracavitary thrombi, previous thromboembolic phenomena, dilatation of the cardiac chambers, and HF has been associated with a higher risk of thromboembolic accidents in anatomopathological and clinical studies.^838^Regional ventricular dyskinesias, mainly apical, are a major characteristic of CCCD, in which their prevalence is higher as compared to that in other etiologies, thus predisposing to the formation of mural thrombi and embolic events, especially systemic ones.^830^Similarly to other cardiopathies, cardiac dilatation and HF are well-known risk factors for the occurrence of thromboembolic events. Atrial fibrillation, a relatively late manifestation and usually associated with ventricular dysfunction, is an additional thrombogenic factor in that cardiopathy.^830^

The mortality associated with thromboembolic events in CCCD is usually related to cerebral and pulmonary embolisms, with more than one arterial territory commonly affected.^839^Regarding pulmonary embolism, most events originate in the right cardiac cavities, differently from other cardiopathies, in which the thrombi commonly originate from the lower limbs.^839^Pulmonary embolic phenomena are clinically underestimated in CCCD, considering their high prevalence in postmortem examinations,^830^the same occurring with noncerebral systemic embolisms. Pulmonary thromboembolism can affect up to 37% of patients with HF, but it is rarely reported in patients without HF. In 85% of the cases, it associates with mural thrombosis of the right cardiac chambers.^363^

Systemic thromboembolism affects mainly the brain and can be the initial clinical manifestation of CD, associating with the presence of mural thrombi and LV apical aneurysm. Because of its higher clinical expression, stroke has been in the center of several investigations. Embolic stroke in CCCD was first reported by Nussenzveig *et al* . in 1953.^840^In 1955, Rocha & Andrade described systemic thromboembolic phenomena in patients with CCCD.^841^

The presence of CCCD is considered an independent risk factor for the occurrence of ischemic stroke. Case-control studies have shown that HF, arrhythmias on ECG, female sex, and LV apical aneurysm are independent risk factors for cerebral thromboembolism in patients with CD.^842 , 843^

In a study using transthoracic and transesophageal ECHO and assessing 75 patients, LV mural thrombi were found in 23% of the cases, in a clear association with the history of stroke. Apical aneurysm was identified in 47% of the patients and significantly related to mural thrombosis and stroke. Thrombosis of the left atrial appendage was identified in 4 patients, while thrombosis of the right atrial appendage, in 1 patient. There were 13 deaths in a 24-month follow-up, 7 of which were sudden, 5 due to HF progression, and 1 due to stroke. Differently from other cardiopathies, in CCCD, stroke was more frequent in patients with mild LV systolic dysfunction and NYHA class I.^363^

Two hospital case series have reported a low annual incidence (1% to 2%) of thromboembolic phenomena in patients with CCCD and mild to moderate ventricular dysfunction.^363 , 358^However, that incidence was significantly higher (60% per year) in patients with manifest HF, in whom LV apical aneurysm and LV mural thrombosis were observed in 23% and 37% of the cases, respectively. Overall, lumping all the case series, the prevalence of thrombosis of the right chambers (53%) exceeded that of the left chambers (43%).^831 - 834^

The COVID-19 pandemic, the disease caused by SARS-CoV-2, has evidenced higher predisposition of infected patients to arterial and venous thrombotic complications, because of inflammatory changes, those of the endothelial microcirculation and blood stasis.^844^Considering that CCCD also propitiates a proinflammatory and prothrombotic state, the association of those two diseases could act synergistically to potentialize the appearance of thromboembolic events.

However, so far, there has been no clear evidence of more relevant clinical events in the association of both diseases. Nevertheless, interventional settings have reported that acute coronary syndromes in patients with COVID-19 tend to present later after symptom onset and with higher clinical severity.^845^Therefore, ongoing studies are testing more aggressive antithrombotic therapies in those settings. Patient management when both infections coexist is addressed in a specific topic of this guideline.

### 12.4. Risk Assessment of Stroke

As already discussed, patients with more advanced forms of CCCD are at higher risk for developing thromboembolic episodes because they are more susceptible to thrombus formation, due to the presence of venous stasis and low blood flow, dilatation of cardiac chambers, LV systolic dysfunction, and inflammatory vascular phenomena. Other factors, such as segmental hypocontractility and arrhythmias, especially AF, contribute to increase the risk for thromboembolism.^832^Even patients with CCCD without global ventricular dysfunction can have a significant increase in the risk markers of thrombosis, suggesting a prothrombotic state in the earliest stages of disease.^846^

A systematic review of eight observational studies, involving 4158 patients, has evidenced the clear association between CCCD and the risk for stroke. That study has shown that, in chronically *T. cruzi* -infected patients, as compared to noninfected ones, there was an excessive risk for stroke of approximately 70% (RR = 1.70; 95% CI, 1.06-2.71).^361^

A prospective cohort of 1043 patients with CD (with and without cardiopathy), in a mean 5.5-year follow-up, has reported a 3% incidence of cardioembolic stroke, that is, 0.56% per year.^364^The authors have proposed a risk score ( *IPEC-FIOCRUZ)* for the stroke occurrence based on points and the indication for prophylaxis of embolic events, considering the presence of LV systolic dysfunction (any grade and location - 2 points), apical aneurysms (1 point), primary alterations of ventricular repolarization on ECG (1 point), and age > 48 years (1 point). Patients with 4-5 points were considered at high risk for cardioembolic stroke. That analysis excluded the classical risk factors associated with cardioembolic complications in other cardiopathies, for which the indication of prophylaxis is already ensured, such as AF, intracavitary thrombi, and previous cardioembolic events. However, the frequency of events was higher than that in other cardiopathies in paired analyses for the same grade of systolic dysfunction, showing that CCCD is in fact a more thrombogenic entity.^364^

Recent studies have suggested that atrial flutter and AF might be more frequent in CCCD than initially reported, and their prevalence increases in more advanced disease stages, accompanying the ventricular dysfunction worsening and being an additional thrombogenic factor.^847^

However, although cardioembolic complications are very frequent in CCCD and should always be assessed as a potential causal factor for ischemic stroke, other mechanisms, such as atherothrombotic or lacunar strokes, and, more rarely, several etiologies of vasculitis and coagulopathies^848^can be implicated in the ischemic stroke genesis of patients with CD. The recent increase in life expectancy of that population and changes in lifestyle, with contribution of the classical cardiovascular risk factors for atherosclerosis (SAH, dyslipidemia, and diabetes *mellitus* ), make ischemic stroke one of the major causes of death in historical cohorts of patients with CD,^849^although not always the cardioembolic mechanism is implicated.

Occasionally, even the cardioembolic risk condition might not mean a manifestation of CCCD but be associated with the progression of cardiopathy in the elderly, also responsible for the increased incidence of AF. The precise causal nexus of stroke in the elderly and patients with multiple clinical comorbidities might not always be established. However, comprehensive care to patients should be considered the most relevant theme, and appropriate treatment and/or prophylaxis should be established in each situation.

### 12.5. Clinical Findings and Diagnostic Investigation of Ischemic Stroke in Chagas Disease

Stroke is defined as a usually focal neurological deficit, of sudden onset, lasting at least for 24 hours, of presumably vascular cause, occasionally followed by death. The presence of focal neurological signs and symptoms that disappear in less than 24 hours characterizes the transient ischemic attack (TIA). The diagnosis of stroke is based on clinical manifestations, with the presence of at least one of the following neurological changes: motor or sensory deficit, aphasia or dysphasia, hemianopsia, conjugate eye deviation, or sudden onset of apraxia, ataxia, or sensory perceptual deficit.^850^

In patients with CD, because of the predominance of cardioembolic stroke, symptoms of cortical manifestation are frequently observed and the anterior circulation syndromes, related to impairment of the territory of the middle and anterior cerebral arteries, are the most common.^843^They are characterized by signs of superior cortical dysfunction (alterations of speech, visuospatial functioning, or consciousness level), homonymous hemianopsia (visual deficit affecting both eyes equally), and motor deficit and/or sensory alteration of at least two body areas (face, upper limbs, and lower limbs). Extensive cortical lesions usually cause all those neurological disorders, thus characterizing the total anterior circulation syndrome. Less extensive cortical lesions can lead to partial anterior circulation syndrome, with two of those three sets of neurological manifestations.

The posterior circulation syndromes, related to impairment of the posterior cerebral artery territory, such as the cerebellum and brain stem, are less frequent. These syndromes manifest with at least one of the following alterations: paralysis of the cranial nerves associated with contralateral sensory/motor deficit; bilateral sensory/motor deficit; conjugate eye deviation; cerebellar dysfunction without ipsilateral long-tract deficit; isolated hemianopsia or cortical blindness.^851^

The signs and symptoms of ischemic stroke secondary to atherosclerosis of the great vessels can be similar, and, thus, indistinguishable from those present in cardioembolic events regarding sensory/motor deficit, however without alteration of the cortical functions, such as speech or cognitive functions.

Some patients can experience lacunar syndromes, usually related to the presence of other cardiovascular risk factors concomitant with CD, such as SAH and diabetes *mellitus.* Lacunar strokes are characterized by the presence of sensory/motor deficits, which can occur in isolation or in combination, or by ataxic hemiparesis.

Silent ischemic stroke can occur in a significant proportion of patients with CCCD, having been reported in 18% of the individuals with CD included in a case-control study.^852^

Brain computed tomography or MRI is recommended to confirm the diagnosis of structural lesions due to vascular events, to classify the type of event, and to exclude differential diagnoses.^853^When assessing acute stroke, computed tomography is the most cost-effective strategy, because it is a fast method largely available in most emergency services. The MRI is particularly useful to assess posterior circulation lesions, small cortical infarctions, lacunar infarctions, and mainly to analyze unusual images when there is doubt about the diagnosis of stroke.^854^

In patients with CD and diagnosis of stroke, the imaging tests show a predominance of cerebral lesions in the territory of the middle cerebral arteries, which is usually the most affected area in individuals with ischemic stroke. A large proportion of patients with CD and ischemic stroke of undetermined etiology have structural impairment of the territory of the cerebral artery lower branches, which can be associated with embolism from the heart, possibly due to anatomic and hemodynamic factors.^855^

The neurological signs and symptoms of stroke can be the first clinical manifestations of patients with CD.^856^Thus, investigation of *T. cruzi* infection by use of serological tests should be considered in cases of ischemic stroke in patients from CD endemic areas or children of mothers at the same endemic risk, mainly in cases of cerebrovascular events secondary to thromboembolism or of undetermined etiology.^857^

Most patients with CD who develop stroke are known to have cardiomyopathy signs.^361^Cardiac arrhythmias, mainly atrial flutter and AF, LV systolic dysfunction, left atrial dilatation, apical aneurysm, and intracavitary thrombosis are associated with stroke in patients with CCCD.^358 , 360 , 364 , 849^Thus, ECG and transthoracic ECHO at rest are recommended to investigate those risk factors. For patients with poor echocardiographic window for proper assessment of the LV apex, microbubble contrast ECHO and CMRI can be useful to identify aneurysms and mural thrombi in the region.^858^

In cases of embolic ischemic stroke whose thrombogenic source remains undetermined after initial assessment, additional diagnostic investigation can be made with 24-hour Holter monitoring and transesophageal ECHO. If the patient has a PM or ICD, the device’s event recording can be assessed aiming to identify arrhythmias with the potential to produce embolization.

The concomitant prevalence of other risk factors for cardiovascular diseases, such as SAH, diabetes *mellitus* , dyslipidemia, and smoking, in patients with CD can be high, mainly in those with ischemic stroke.^856^In such cases, noninvasive investigation of atheromatous disease of the carotid and vertebral arteries by use of Doppler ultrasound, computed tomography angiography, or magnetic resonance angiography is recommended, mainly for patients with cerebral infarction related to the anterior cerebral circulation. In addition, transcranial Doppler can be useful in such cases.

Differential diagnosis with other rare clinical conditions, such as vasculitis and thrombophilias, in cases of clinical suspicion or when the diagnosis remains undetermined should be investigated by use of blood coagulation tests, with assessment of prothrombin time (international normalized ratio - INR) and platelet count or specific search for other rare etiologies.

Table 12.1 summarizes the recommendations for investigation of ischemic stroke in CD.

**Table 12.1:** – Investigation of ischemic stroke in CCCD

Indication	Test	Recommendation grade	Level of evidence
Diagnostic confirmation of acute neurological event, classification of the type of event, and assessment of differential diagnoses or complications	Brain CT	Strong	B
	Brain MRI (particularly to assess small infarctions or posterior circulation impairment)	Conditional	B
Investigation of T. cruzi infection in individuals with ischemic stroke and epidemiological antecedents	Serology for Chagas disease (two different serological techniques)	Strong	C
Investigation of cardiac impairment and source of embolism associated with CCCD	ECG and transthoracic ECHO	Strong	B
	Cardiac MRI or microbubble contrast ECHO (if inappropriate window for LV apical assessment)	Conditional	B
	24-h Holter (undetermined source of embolism after initial assessment)	Strong	B
	TEE (undetermined source of embolism after initial assessment)	Conditional	C
Differential diagnosis with atherothrombotic etiology in patients with cardiovascular risk factors	Carotid and vertebral artery doppler	Strong	B
	Brain CT angiography or MRI/angiography	Conditional	B
	Transcranial doppler	Conditional	C
Differential diagnosis with other etiologies	Blood coagulation tests with prothrombin time (INR) and platelet count	Strong	B

CCCD: chronic cardiomyopathy of Chagas disease; CT: computed tomography; MRI: magnetic resonance imaging; ECG: electrocardiogram; ECHO: echocardiogram; LV: left ventricular; TEE: transesophageal echocardiogram; INR: international normalized ratio.

### 12.6. Treatment of Ischemic Stroke in Chagas Disease

The therapeutic management of ischemic stroke in CD depends on the time from symptom onset, comorbidities, and severity and extension of the ischemic area. The cerebral ischemia can present as TIA, silent cerebral infarctions, or ischemic stroke with mild or severe motor sequelae and hemorrhagic transformation, causing death, chronic cognition impairment or drastic physical limitation.^859 - 861^

The initial therapeutic approach to stroke in CCCD is similar to that in other etiologies, aiming to stabilize and reduce damages, and prevent complications through admission to the specific intensive care unit for patients with stroke, where the following general measures should be observed:^862^1) Control of vital functions and temperature; 2) Management of SAH, avoiding hypotension and consequent cerebral ischemia worsening; 3) Control of hyper- or hypoglycemia; 4) Careful hydration and serum sodium level control; 5) Protection of the airways and swallowing, preventing infection from bronchoaspiration; 6) Early identification of hypoventilation, preventing CO_2_retention and hypoxemia by use of oxygen supplementation; 7) Prevention of deep venous thrombosis by use of heparin or its oral substitutes or mechanical methods of pneumatic compression when indicated; and 8) Determination of the cerebral damage extension by use of brain computed tomography or MRI to treat cerebral edema and identify the risk for or presence of hemorrhagic transformation, assessing suggestive symptoms, such as intense and persistent headache, sleepiness, decreased level of consciousness, in addition to worsening of the motor/sensory deficits.

For acute and more severe cases presenting in the therapeutic window (time from symptom onset <4.5 hours) and with no contraindication, thrombolysis should be instituted, usually with intravenous rt-PA. If initial brain computed tomography suggests early hypodensity equal to or greater than one third of the middle cerebral artery territory, thrombolysis is contraindicated because of the high risk for hemorrhagic transformation. In specific cases, endovascular thrombectomy enables treatment with a larger therapeutic window, but still shorter than 24 hours.^862^

After the acute phase of the ischemic event, oral anticoagulation with warfarin is the treatment established for the secondary prophylaxis of thromboembolic complications originated from the heart, in the presence of either arrhythmias or intracavitary thrombosis.^1 , 2 , 722 , 862^Frequent anticoagulation adjustments are necessary to maintain the target therapeutic range (INR between 2 and 3) and treatment should continue throughout life.

As a simpler option, for not requiring recurrent medical visits to adjust anticoagulation, the new oral anticoagulants of direct or indirect action (rivaroxaban, edoxaban and apixabana) can be empirically used for patients with chronic atrial arrhythmias, such as atrial flutter or AF, with potentially beneficial results, even superior to warfarin.^722^More recently, meta-analyses comparing the new anticoagulants to warfarin in individuals with LV thrombosis associated with ICM or DCM have suggested that those drugs would have efficacy similar to that of vitamin K antagonists regarding frequency of thrombus resolution, and prevention of stroke or other thromboembolic events and hemorrhagic complications.^863^This plausibility might apply to patients with CCCD, but remains to be demonstrated, and the cost of long-term treatment might limit its use in populations of known vulnerability and social marginalization.

The right time to begin chronic oral anticoagulation after ischemic stroke is controversial and has not been studied systematically. For patients with TIA, it is reasonable to begin anticoagulants 24 hours after symptom onset; for patients with mild deficits, after 3 days; for patients with moderate deficits, after 6 to 8 days; and for patients with severe deficits, after 12 to 14 days, as long as, in all these situations, hemorrhagic transformation is ruled out after neuroimaging assessment.^862^

Risk scores, such as CHADS2 and CHA2DS2-VASc, are used to guide the primary and secondary prophylaxis of cardioembolic stroke in the presence of AF and other cardiovascular risk factors,^2 , 722 , 862^regardless of the cardiopathy etiology. At first, all patients with 2 or more points (maybe also men with at least 1 point) benefit from prophylaxis with anticoagulants; however, the risk for hemorrhagic complications should be always assessed during the chronic use of those drugs.

The HAS-BLED score (based on uncontrolled hypertension; abnormal renal/hepatic function; previous stroke; history of or predisposition to bleeding, such as anemia; labile INR; age > 65 years; concomitant use of drugs/alcohol) has been validated in different cohorts (but not in individuals with CD) to define the risk for hemorrhagic complications, with high risk of bleeding identified by a score ≥ 3.^722 , 862^In that context, the risk *versus* benefit assessment should be defined individually and shared with the patients and their families.

### 12.7. Prevention of Cardioembolic Events in Chagas Disease

Prevention of cardioembolic events in patients with CCCD is extremely important because of the high potentially negative impact of those complications on morbidity, mortality, and quality of life.^2^During clinical follow-up, it is necessary to periodically screen the potential risks for cardioembolic events, such as: systolic ventricular dysfunction and HF, and presence of ventricular aneurysms, mural thrombi, and arrhythmias (especially AF).^1 , 364^

All patients with CCCM should undergo ECG and ECHO periodically during outpatient follow-up and at the clinical assessment of an acute or previous cardioembolic event.^1 , 2^A long ECG tracing of at least 30s should be ideally performed to enable the identification of atrial arrhythmias.^1^The ECHO allows the visualization of the cardiac cavities, identifying different grades of LV systolic dysfunction, regional areas of dyskinesia, aneurysms (mainly apical), spontaneous contrast, and mural thrombi, characterizing them as mobile or sessile, with high potential for embolism, or organized.^858^

Arrhythmias with high potential for embolism, such as atrial flutter or AF, should be actively sought during the clinical follow-up of patients with CD by use of annual ECG, a strategy to identify disease progression from the indeterminate chronic form to cardiopathy.^2^

In addition, the clinical history and physical examination are essential for the assessment of symptoms, such as palpitation, tachycardia, chest pain, dizziness, lipothymia, malaise, weakness, dyspnea, and functional class worsening, which lead to the clinical suspicion of arrhythmia. Regarding physical examination, the most important is the detection on auscultation of irregular pulse or cardiac rhythm. Because arrhythmic events can occur in paroxysms, arrhythmia might not be identified at the time of clinical assessment. If the clinical suspicion persists, continuous electrocardiographic monitoring by use of 24-hour Holter is required.^1 , 2 , 722^

In patients with implanted devices, such as PM, ICD or cardiac resynchronization, the irregular rhythm might not be detected on physical examination, and an ECG assessment might be necessary or, even more appropriately, to resort to the event recording of those devices.^2 , 722^These intracardiac devices should be systematically assessed on each visit, aiming at identifying the recording of silent episodes of AF. A proper interface between the clinical follow-up teams (clinical cardiology and arrhythmology) is necessary for the recommendation of appropriate interventions, such as the beginning of anticoagulation for primary prevention.

According to recent guidelines on HF and arrhythmology, considering heart diseases of several etiologies, the identification of mural thrombosis, previous thromboembolic phenomena, and AF with CHA2DS2-VASc ≥ 2 would already indicate anticoagulation for prophylaxis against cardioembolic events.^722 , 863^It is plausible to admit that the same recommendations would empirically apply, by extrapolation, to patients with CCCD.

As already mentioned, recognizing the CCCD higher potential to produce embolism, a risk score specific for cardioembolic stroke was developed for this etiology, widening the recommendations classically established for other cardiopathies.^364^By use of risk-benefit analysis, the researchers proposing that *IPEC-FIOCRUZ* score have also suggested that, for individuals with maximum score (4-5 points), the 4.4% annual incidence of stroke would exceed the estimated 2.0% annual rate of severe bleeding associated with the use of warfarin.^364^

Although previous guidelines have confirmed the use of that score,^1 , 2^its review is currently mandatory for its specific use in patients with CCCD (that is, not including the IFCD subgroup, which is practically not at risk for stroke), with occasional correction of the points attributed to the variables (for example, 2 points were attributed to the independent variable systolic dysfunction, with a 2.6 beta coefficient of regression, while the correct would be rounding it up to 3 points), more adequate definition of age groups, and mainly external validation.

The external validation of risk scores is especially relevant for their recommendation in clinical practice, in the context of CCCD, considering the current concepts.^473 , 864 , 865^With the implementation of the methodological principles, the score can be invigorated and, in consistency with its undeniable and historical scientific role, recover its coverage and applicability.^474 , 866^In addition, the empirical therapeutic managements suggested by the time it was formulated^364^should be ideally supported by randomized studies of efficacy.^474 , 866 , 867^

Considering the clear trend towards longer survival of patients with CD recently observed with a consequent increase in the frequency of cardiovascular risk factors in that population, there is an increase in the prevalence of AF (not necessarily related to CCCD, but occasionally associated with the cardiopathy of the elderly or other clinical comorbidities), resulting in additional risk for cardioembolic stroke. Thus, recommendations for lifestyle changes with control of SAH, diabetes *mellitus* , and dyslipidemia, as well as smoking cessation, weight loss, and regular physical activity^722^are also important to reduce cardioembolic events in that population. Table 12.2 summarizes the recommendations for treatment and prevention of cardioembolic stroke in CCCD.

**Table 12.2:** – Treatment and prevention of cardioembolic events in CCCD

Indication	Management	Recommendation grade	Level of evidence
Treatment (∆t ≤ 4.5h), excluded the contraindications and potential risk of hemorrhagic transformation	IV thrombolysis (rt-PA)	Strong	B
Oral anticoagulation (primary or secondary prevention)	AF with CHADS-VASc ≥ 2 (women) and ≥ 1 (men)	Strong	B
	Mural thrombus	Strong	C
	Previous ischemic stroke	Strong	C
Investigation of cardiac impairment and source of embolism associated with CCCD	Transthoracic ECHO	Strong	C
	24-h Holter	Strong	C
	Interrogate Holter for events (patients with PM/ICD)	Strong	C
Reduction of the risk for AF (regardless of intrinsic predisposition to CCCD)	Lifestyle change (control SAH, DM, dyslipidemia, weight loss, physical activity, smoking cessation)	Strong	C

CCCD: chronic cardiomyopathy of Chagas disease; ∆t: time from symptom onset; IV: intravenous; rt-PA: recombinant tissue plasminogen activator; AF: atrial fibrillation; ECHO: echocardiogram; PM: pacemaker; ICD: implantable cardioverter-defibrillator; SAH: systemic arterial hypertension; DM: diabetes mellitus.

## 13. Management in Special Subgroups and Handling of Issues Related to *T. cruzi* -HIV Coinfection, Pregnancy, Physical Activity, Surgical Risk, General Anesthesia, and COVID-19

### 13.1. *T. cruzi* -HIV Coinfection

With estimates of 37 million individuals living with HIV/AIDS worldwide, the risk of *T. cruzi-* HIV coinfection^868^is a reality in endemic and nonendemic areas with immigrants infected with the parasite.^869 , 870^

The *T. cruzi-* HIV coinfection was initially reported in 1990^871^as RCD, and cited in 1988 in Brazil with the identification of the parasite in the cerebrospinal fluid of a patient with AIDS.^83^Described mainly in Brazil and Argentina, but also in other countries (Bolivia, Chile, Spain, USA, Colombia, Venezuela, Jamaica, Germany, and Switzerland), the RCD is characterized by high morbidity and mortality and maternal-fetal transmission,^83 , 84 , 580 , 872 , 873^interfering in the evolution of both CD and HIV infection. The *T. cruzi-* HIV coinfection usually affects HIV-infected patients with severe immunodeficiency (CD4^+^cells < 200/mm^3^) and detectable viral load due to failure to respond to effective antiretroviral therapy. In active HIV infection, the marked reduction in CD4^+^cells expresses the deficiency in TH1 response,^874^responsible for the activation of CD4^+^and macrophages capable of secreting IFN-γ and destroying the parasites, thus increasing parasitemia and tissue parasitism.^875^

The RCD presents as meningoencephalitis in approximately 2/3 of the cases, followed by myocarditis, meningoencephalitis plus myocarditis, pericarditis, duodenitis, gastritis, erythema nodosum, and colpitis.^83 , 580 , 872 , 873^In the congenital form of the *T. cruzi-* HIV coinfection,^872 , 873^abortions, low birth weight, sepsis, and meningoencephalitis occur. More rarely, oligosymptomatic forms manifest as fever, erythema nodosum, myelitis, and as an asymptomatic postpartum woman with a stillborn baby due to congenital CD.^872^

The meningoencephalitis caused by *T. cruzi* should be differentiated from toxoplasmosis, tumors, and infectious and degenerative diseases. Acute myocarditis in RCD should be differentiated from decompensated CCCD. Levels of CD4+ ≤ 200/mm^3^are observed in approximately 2/3 of the cases and are even lower in RCD than in patients without RCD. A study has reported a 52.5% mortality in RCD (63 patients out of 120).^83^Retrospective studies have reported RCD in 10%-15% of the coinfection cases, while prospective studies have reported RCD in 10% of patients in previous follow-up.^83 , 580 , 873^

The prevalence of coinfection has been estimated at 1.5%-5.0% in Brazil^83 , 580^and 4.2% in Argentina,^876^being higher among illicit drug users.^877^Approximately 4570-15 360 cases of coinfection have been estimated based on the number of patients infected with *T. cruzi* and HIV in Brazil and Argentina, suggesting a usually underestimated number in the literature.

Of the mortality causes in coinfection,^878^AIDS was the underlying cause of death in 2/3 of the cases and CD in 17.5%. The IFCD predominated in approximately half of the coinfection cases, the cardiac form occurred in 37%, followed by the digestive and cardiodigestive forms in 5% and 6%, respectively.^873^Reduced levels of CD4^+^(in the diagnosis of coinfection) have been associated with the prognosis of RCD and mortality from RCD. In addition, the presence of parasitemia has been associated with the TH2 response, suggesting imbalance favoring the parasite.

Thus, cases of HIV infection or CD should be actively investigated from the clinical and epidemiological viewpoints with indication for serological screening, aimed at the early diagnosis and control of both infections.

Coinfection is diagnosed by two positive serological tests for both infections and/or parasitological tests for the diagnosis of CD.^580 , 873^In CD, in case of discordant tests (ELISA, IIF or IHA), a confirmation test (immunoblotting/immunochromatography) or immunoenzymatic test with recombinant antigen or immunofluorescence is indicated. For the HIV infection, a positive ELISA or CLIA for the HIV1 and HIV2 antigens should be confirmed by use of immunoblotting/immunochromatography for the HIV1 and HIV2 antigens.^580 , 873^Indirect parasitological tests and PCR for *T. cruzi* are specific but have low sensitivity for the diagnosis (approximately 50% in the chronic form), although higher in the coinfection.^879 - 881^

The diagnosis of RCD should be made by use of the gold-standard methods of direct microscopic detection of the parasite in blood and biological fluids (cerebrospinal and pericardial fluids) and/or in stained tissues.^83 , 580^Concentration techniques (leukocyte cream, microhematocrit, Strout) are more sensitive than the parasite search in simple peripheral blood smear or fresh peripheral blood. Biopsy can be indicated when other noninvasive methods fail.^83 , 580^Patients with RCD can have negative CD serological tests,^876^but the investigation should continue by use of direct microscopic methods. Qualitative PCR and indirect parasitological tests with enrichment, such as blood culture and xenodiagnosis, have low positive predictive value for the diagnosis of RCD, because they can be positive in chronic patients without reactivation.^879^However, semiquantitative tests, such as nymph count on the xenodiagnosis^580^and qPCR, are usually useful for RCD monitoring.^880 , 881^

The antiparasite treatment with benznidazole is mandatory for patients with RCD,^83 , 580 , 873^at the dose of 5 mg/kg/day for 60 days. Nifurtimox is indicated as second choice when benznidazole is not available or in the presence of an adverse event contraindicating its use.^83 , 580^In the first weeks after treatment, the direct search for the parasite in the leukocyte cream helps monitor therapeutic failure in positive cases; negative results do not indicate therapeutic success in the short run. Cure control should be followed up with qualitative PCR or indirect parasitological tests (blood culture) within 3, 6, 9, 12, and 24 months of therapy onset and with serological tests within 6, 12, and 24 months of therapy onset.

Coinfected patients without RCD have shown better antiparasite response in the presence of higher parasitemia levels or higher levels initially.^580^

Follow-up is recommended at reference healthcare services to control both the viral load, with effective antiretroviral therapy control to restore the TH1 response, and CD, with parasitemia monitoring to avoid RCD or to enable its early diagnosis and treatment ( Table 13.1 ).

**Table 13.1:** – Recommendations for diagnosis and treatment of *T. cruzi* /HIV coinfection

Summary of recommendations	Recommendation grade	Level of evidence
Active search for the coinfection diagnosis, based on clinical-epidemiological suspicion, by use of serological tests	Strong	B
Monitoring of coinfected patients by means of parasitemia and use of molecular and parasitological methods to prevent reactivation, preferably every 3 months	Strong	B
Use of effective antiretroviral therapy to maintain proper immune response	Strong	A
Diagnosis of reactivation by use of concentration methods and direct microscopy in blood and biological fluids or biopsy	Strong	B
Use of quantitative PCR to diagnose reactivation after establishing the boundaries between reactivation and non-reactivation in the endemic region, based on differences between the number of copies in reactivation and in non-reactivation in a large number of patients, by using known initiators and prevalent molecular lineages	Conditional	C
Use neither indirect parasitological methods nor positive qualitative methods for the diagnosis of reactivation in the chronic phase of Chagas disease	Conditional	B
Treat reactivation with benznidazole immediately after the diagnosis, with hospitalization in severe cases with encephalic, myocardial and/or bone marrow impairment	Strong	C
Preemptive treatment of coinfectied patients with high parasitemia on xenodiagnosis (>20% of nymphs +) or standardized quantitative PCR with higher values than the median ones in the region	Conditional	B
Maintain effective antiretroviral therapy to ensure proper immune response, CD4+ levels >200 cells/mm^3^, and undetectable viral load to prevent reactivation	Conditional	B
Secondary prophylaxis 2x/week with benznidazole for patients with CD4^+^<200 cells/mm^3^	Conditional	C

The indication for secondary prophylaxis with benznidazole (5mg/kg/day 3x/week) in patients with CD4^+^< 200cells/mm^3^, similarly to the prevention of other opportunistic infections, is controversial, and there are neither reliable prospective studies nor retrospective series on CD.

### 13.2. Seropositivity in Potential Donors at Blood Banks

In Brazil, serological screening for CD has been mandatory for all blood donors since 1969.^882^After the appearance of AIDS in the 1980s, several measures and legislations have been developed and adopted to increase the control of blood banks and donors, particularly with the creation of blood centers and the centralization of the control and surveillance activities under the responsibility of the State Health Secretariats.^883^

The Ministry of Health Ordinance nº 158 from 2016 established as unfit for blood donation individuals with history of household contact with triatomines in endemic areas in addition to those with a clinical or laboratory diagnosis of CD.^884^The serological screening for CD comprises automated serological tests of high sensitivity and specificity to detect anti- *T. cruzi* IgG antibodies, of which the most often used are ELISA and, more recently, CLIA.^885 , 886^For the serological screening at blood banks, only one serological test is necessary and it can be repeated if the result is positive.^887^In such case, the donated blood cannot be used and the donor should be contacted and referred to a CD reference health center for diagnosis elucidation.

With the control of vectorial and blood transfusion transmission, the mean prevalence of CD among blood donors has been decreasing rapidly. More recent projections have estimated a CD prevalence of 0.18% of the potential blood donors in Brazil.^2^However, these rates can vary according to the areas where the donations occur and the age of donors, being usually higher in historically endemic regions and at the older groups.^888^

Recent studies conducted with blood donors in the Northeast region have reported prevalence of 0.17% to 0.57% in the state of Ceará and of 0.18% to 2.4% in the state of Piauí.^889 , 890^A study performed in the city of Uberaba, state of Minas Gerais, with a large number of donors for 15 years has shown a 0.03% annual drop in the prevalence rate, and, in the last year studied, only 0.08% of the donors were ineligible due to seropositivity.^891^

With the drop in prevalence among younger donors, an increase in inconclusive or undetermined cases has been observed, most of which resulting from false-positive tests.^885^All positive cases should be referred to CD reference health centers to undergo new tests to confirm or rule out the diagnosis of CD.

### 13.3. Physical Activity

The practice of physical activity is an important intervention strategy to prevent and treat several chronic diseases, mainly those related to the cardiovascular system.^892^Recently, the WHO has published recommendations on the practice of physical activity by healthy individuals and those with specific health conditions and diseases. Usually, 150 minutes of moderate physical activity and/or 75 minutes of vigorous physical activity per week are recommended for cardiovascular health benefits.

In addition, moderate strengthening exercises for the major muscle groups should be performed at least twice a week and assessed by use of the perceived exertion scale. Flexibility and balance exercises should also be practiced, mainly by the elderly, to maintain amplitude of movement and autonomy for daily life activities.

Health benefits can be obtained even at lower physical activity levels, and previous inactive individuals should begin training gradually.^893^Small amounts of physical activity can be more beneficial to health as compared to being inactive, and, due to the dose-response relationship, higher amounts can be even more beneficial.^893 , 894^

However, the benefits of physical activity to physical and mental health of individuals with CD have not been completely explored. Some studies have shown promising results regarding improvement of physical functioning and quality of life.^895 - 897^Such studies, however, have included only patients with the cardiac form of CD, and so far there has been no proper assessment of the influence of that strategy on the IFCD.

Thus, the recommendation of physical exercises for individuals with the IFCD should be identical to those of the general population, aiming to control comorbidities, as well as to improve physical fitness and quality of life. Lifestyle interventions that gradually increase physical activity levels should be encouraged, considering each individual’s physical capacity and functioning. Some studies have shown that the practice of physical exercises is associated with improvement in bowel transit, but its effects on individuals with the digestive form of CD have not been investigated.^898^

The effects of physical activity on CCCD have been recently assessed, mainly by use of cardiovascular rehabilitation programs.^895 - 897^In a pioneer study on the topic, a RCT has assessed the effects of a cardiovascular rehabilitation program on patients with CCCD followed up for 3 months, and the physical training promoted improvement of physical fitness and quality of life.^895^

Later, an intervention study has reported that a cardiovascular rehabilitation program for patients with HF due to CD was associated with improvement of the cardiac function assessed by use of LVEF, of respiratory muscle strength, and of quality of life after 8 months of follow-up.^896 , 899^

More recently, the PEACH study, a RCT, has reported improvement of physical functioning and microcirculation after 6 months of a cardiovascular rehabilitation program for patients with CCCD with and without HF.^897 , 900^Thus, physical exercise has been proven to be an effective intervention strategy to improve several clinical and quality of life parameters in CCCD ( Table 13.2 ).

**Table 13.2:** – Recommendations of physical activity practice for individuals with Chagas disease

Summary of recommendations	Recommendation Grade	Level Of Evidence
Benefits of physical activity for individuals with the indeterminate form of Chagas disease	Strong	C
Benefits of physical activity for individuals with the cardiac form of Chagas disease	Conditional	B
Benefits of physical activity for individuals with the digestive form of Chagas disease	Conditional	C

### 13.4. Pregnant Women

The prevalence of *T. cruzi* infection among pregnant women ranges from < 1% to 70.5% depending on the country, geographical area, and location (rural or urban), while the vertical transmission rate in endemic countries ranges from 0% to 18.2%.^38 , 39^The *T. cruzi* vertical transmission rate differs between regions, varying from around 1.0% in Brazil to 4%-12% in other Southern Cone countries, and seems to depend on parasite- and host-related factors.^40^

The congenital transmission of CD can occur at any phase of the maternal disease; however, the highest transmission rate occurs among pregnant women in the acute phase of disease, approximately 30%, while the overall rate is 4.7%.^88 , 901^In Brazil, the congenital transmission rate of CD ranges from 0% to 5.2%; however, there is great heterogeneity depending on the geographical region assessed. The highest regional congenital transmission rate was observed in the Southern-Southeastern region (2.1%), followed by the Northeastern (1.6%) and West-Central (0.9%) regions.^87^

The evidence of increased risk for abortion or prematurity among seropositive pregnant women is inconclusive. However, studies have suggested that the maternal chronic infection influences neither the clinical course of pregnancy nor the newborn health, as long as there is no vertical transmission. However, the fetal infection increases the likelihood of premature delivery, low birth weight, and natimortality.^902^

The congenital transmission of *T. cruzi* is a complex process, resulting from the interaction of multiple factors related to the parasite, placenta, and fetal and maternal immune response.^903^The parasite load of women infected during pregnancy is fundamental to congenital transmission.^904^Parasitemia can reappear with the RCD usually associated with the transient physiological immunosuppression of pregnancy.^905^In addition, the role of maternal age and number of gestations in increasing the risk for transmission awaits further investigation. However, there is evidence that the innate immune response activation, by use of pro- and anti-inflammatory mediators, in pregnant women can contribute to reduce the occurrence and severity of the congenital infection.^906^

The impact of CD on the course of pregnancy is controversial. Some studies have pointed to the benignity of the association, while others have reported a high incidence of pregnancy complications and perinatal mortality, as well as of neonatal hypotrophy, considering *T. cruzi* -infected pregnant women a group at high obstetrical risk.^907^

The prognosis of pregnant women with CCCD is closely related to the severity of ventricular dysfunction and functional class at the beginning of pregnancy. Patients in functional class I/II at the beginning of pregnancy usually reach delivery uneventfully; however, those in functional class III/IV have a 25% to 50% probability of death.^908^Heart disease, as long as assisted and not severe, does not contraindicate pregnancy. Patients with HF and/or severe arrhythmias should be oriented not to get pregnant, but if they do, they require special follow-up and care.

The etiological treatment should not be administered to pregnant women nor women at reproductive age not using contraception. Importantly, there is evidence that etiological treatment reduces the risk for congenital transmission in a subsequent pregnancy.^569 - 571^

In addition, in the exclusive case of acute CD, the etiological treatment can be instituted for pregnant women, considering maternal morbidity and mortality, higher risk of congenital transmission, and impact on the newborn health. Pregnant women with severe acute CD (myocarditis or meningoencephalitis) should be treated regardless of the gestational age because of the high maternal morbidity and mortality, in addition to the high risk of congenital transmission of CD (22% to 71%), and the potential impact on the newborn health. Pregnant women with non-severe acute CD should be ideally treated after the second trimester of pregnancy, because of the potential risk of congenital malformation related to benznidazole.^8^

The use of cardiovascular drugs by pregnant women with CD should follow selective and individualized medical indication, because of the potential risk of side effects on the fetus. Infected mothers should be treated after the delivery and lactation period to prevent interrupting breastfeeding because of possible adverse reactions. CD should be investigated systematically in the relatives and other children born from infected mothers (serological diagnosis), and the positive cases should be assessed clinically and treated according to the already discussed principles.^86^

### 13.5. Newborns

Currently, in vector-free areas inside and outside Latin America, *T. cruzi* infection is mainly disseminated through congenital or perinatal vertical transmission, which exceeds blood transfusion and organ transplantation. Despite underreporting and underestimation worldwide, over two million women at reproductive age are infected with *T. cruzi* and 1%-10% of the children from infected mothers are born with CD. Based on recent demonstrations that congenital transmission can be avoided, the OMS changed its objective in 2018, from control to elimination of congenital CD.^86 , 569 , 571^

The severity of congenital CD varies widely, from asymptomatic cases to fatal infection, which is related to the parasitemia level at birth.^904^Studies conducted in Brazil, Argentina, Chile, and Paraguay have shown that 60% to 90% of the newborns with congenital infection are asymptomatic. Among the symptomatic ones, the most frequent clinical manifestations were prematurity, low birth weight, fever, and hepatosplenomegaly.^86^

The congenital infection should be investigated in all children born from seropositive mothers, not only in the first month of life, but also at 6 and 12 months of age. Follow-up for 1 year is essential, because a significant proportion of cases is initially negative, the disease being only detected at a later stage.^86^

The most recommended diagnostic methods in the first month after birth are the direct parasitological tests, by using methods of centrifuge concentration, such as microhematocrit.^908^When positive, those tests provide the undeniable and definitive diagnosis of the infection; however, when the parasite load is low, mainly when transmission occurs in the last trimester of pregnancy or during delivery, the tests can generate false negative results. Thus, more sensitive and automated tests are necessary to the early detection of congenital infection. The positive result determines immediate etiological treatment.^86^Congenital CD is considered acute, thus requiring mandatory reporting.

In the case of a negative parasitological test, the diagnostic investigation should include serological tests (with two different techniques), after the 7th month of life. A serological study before the 6th month is not useful, because of the passive transfer of maternal antibodies to the baby. After the 10^th^month, such antibodies disappear and the diagnosis of congenital CD is more precise; however, delay in the diagnosis reduces the efficacy of the treatment and increases the risk of losing to follow-up.^86^A negative serology after that period allows excluding the diagnosis of *T. cruzi* infection.

The molecular methods represent a promising alternative and have been widely used for the early detection of congenital infections, mainly in Europe. However, they are expensive, and require considerable technical training and careful standardization, which hinder their implementation in laboratory routine. Thus, the molecular methods require wider clinical validations before being considered gold-standard for the diagnosis of congenital infections.^86^

The treatment of the *T. cruzi-* infected newborn is highly effective and can be performed with benznidazole (first option in Brazil) or nifurtimox, for 30 to 60 days, with fewer adverse events as compared to that of adults, and the rate of cure exceeds 90%. The doses recommended for children are 10mg/kg/day of benznidazole in 3 or 2 doses, and 15mg/kg/day of nifurtimox in 3 doses. Benznidazole is available as tablets of 12.5mg, which can be diluted into water and is provided by the State Health Secretariats, while nifurtimox should be requested to the PAHO, through the CD technical group of the Health Surveillance Secretariat of the Brazilian Ministry of Health.

A recently published clinical trial using nifurtimox to treat children (0 to 17 years of age) in Argentina, Colombia, and Bolivia has compared treatments for 30 days *versus* 60 days. By the end of a 12-month follow-up, both regimens showed significant seroconversion or a reduction in the serum levels as compared to historical controls, and the 60 day regimen was superior to the 30 day regimen in the age group of 2-17 years. Nifurtimox was well tolerated, with mainly mild or moderate adverse effects without sequelae, and only 4% of those events determined interruption of treatment.^909^

The etiological treatment should be followed up by use of parasitological and/or molecular tests in the weeks following treatment onset for neonates with parasitemia. After the end of treatment, the patients should be followed up with quantitative serological tests every 6 months. The patient is considered cured when the serology turns negative in two consecutive tests.^86^The time necessary for a test to become negative depends on the patient’s age and treatment onset. The serology of children diagnosed in the first months of life will become negative between the 2^nd^and 12^th^month after treatment onset. The healthcare systems should assess and implement strategies to facilitate the earliest possible diagnosis of the congenital infection, considering the frequent poor adhesion of mothers to follow-up visits in healthcare centers.^86^

Considering the risk of transmission to the newborn through the contact with maternal secretions, breastfeeding should be temporarily suspended only for mothers with RCD or in the acute phase, and more emphatically for those with nipple fissures or bleedings. The individualized assessment of each case is important, considering the great benefit of breastfeeding in the first months of life. In conclusion, mothers on antiparasite treatment for at least 30 days, even in the cases mentioned, can breastfeed freely.^2^

### 13.6. Surgical and Anesthetic Risk

Patients with CCCD have increased surgical and anesthetic risks due to several reasons, which should be considered in the pre-, intra-, and postoperative periods.

The most important preoperative care is HF control with drug optimization and the correction of occasional electrolyte disorders.

Ventricular function should be assessed by ECHO whenever possible. All candidates to surgery should undergo ECG, and, for those with arrhythmias or compatible symptoms, ambulatory ECG monitoring (Holter) may be necessary. Antiarrhythmic drugs should not be suspended, but oral anticoagulants should be interrupted. The new oral anticoagulants, direct thrombin inhibitors, or direct factor Xa inhibitors, such as rivaroxaban, apixaban, and edoxaban, can simply be suspended 24 to 48 hours prior to surgery. Warfarin, however, should be ideally suspended 5 days prior to surgery, which can be performed when the INR is lower than 1.5. During warfarin suspension, the patients at high risk for thromboembolic events should undergo anticoagulation with heparins, such as subcutaneous full-dose enoxaparin. Finally, preoperative assessment of patients with CD should consider the occasional presence of megaesophagus, which increases the risk of bronchoaspiration in the intra- and postoperative periods.^910^

During surgery, patients with CCCD require individualized anesthetic management. The anesthesiologist should consider hemodynamic aspects, such as myocardial dysfunction, sometimes biventricular, which limit the volume infusion and increase the risk for cardiac arrhythmias.^910^Continuous electrocardiographic monitoring is essential to control malignant ventricular arrhythmias and bradyarrhythmias. Invasive arterial and central venous hemodynamic monitoring is useful and should be implemented in more severe cases and major surgeries. Implantation of a temporary transvenous cardiac PM should be considered in patients with advanced AVB, mainly when associated with intraventricular conduction disorders. Intraoperative transesophageal ECHO provides valuable information on the inotropic response to anesthetic medication and the patient’s blood volume status, being useful in selected cases.

Anesthetic care is very important. In patients with ventricular dysfunction, the induction of anesthesia can result in rapid hemodynamic deterioration, which occurs mainly by peripheral vasoconstriction and negative inotropic action induced by anesthetic agents.^910^Intraoperative volume infusion should be judicious. In patients on HF treatment with vasodilators and diuretics, blood pressure levels are commonly low and the time needed for the onset of venous anesthetic action is longer because of the slower blood circulation.^911^In addition, hepatic failure, consequent to right HF, and renal failure alter the pharmacokinetics of most medications.

The autonomic dysfunction of patients with CCCD reduces the contractile reserve and can attenuate the action of exogenous catecholamines, requiring higher doses than the usual ones for hemodynamic stabilization.^422^The best anesthetic regimen should cause the lowest possible grade of myocardial depression and vasodilation. All inhalation anesthetics and most venous anesthetics are myocardial depressant drugs,^912^requiring titration and judicious monitoring by the anesthesiologist. Whenever possible, according to the surgery type, regional anesthesia techniques should be used isolated or associated with general anesthesia due to the lower risk of hemodynamic instability.^910^

Patients with CCCD frequently have implantable electronic devices for the treatment of arrhythmias and/or HF. If a thermal cautery is used during surgery, specific attention is needed. The production of electrical noise by the thermal cautery might lead the device to misinterpret cardiac electrical events, resulting in inhibition of necessary electrical stimuli or triggering inappropriate electrical shock. Patients with a PM should have their devices placed in the DOO or VOO mode. ICD patients should have their devices turned off during surgery or a magnet should be placed on the device to prevent occasional inappropriate shocks. Central venous access should be carefully obtained in those patients because the guidewire can generate noise by contacting the shocking electrode, leading to inappropriate electrical discharges.^913^Regardless of the device type, the thermal cautery should be placed in the bipolar mode at the lowest effective power and be used intermittently with the neutral plate placed as far as possible from the generating unit.

In the postoperative period, patients with CCCD and ventricular dysfunction or cardiac arrhythmias, as well as those undergoing major surgeries, should be at an intensive care unit. The oral anticoagulant used prior to surgery that was temporarily suspended perioperatively, as well as the other medications for HF and arrhythmias, should be reintroduced as soon as possible.

### 13.7. Chagas Disease and Coronavirus Infection

The worldwide spread of COVID-19 made the WHO declare it a pandemic in March 2020. Following the same global epidemiological profile, studies have shown an interrelationship between potential of severity and comorbidities with emphasis on cardiovascular disease and higher fatality rates in patients with those diseases as compared to the general population.^914^

Of the patients with COVID-19, more than 80% have mild symptoms, such as fever, sore throat, and cough,^915^but mortality rates can vary from 2.3% to 27%^916 , 917^in vulnerable populations, such as the elderly and patients with comorbidities,^918 , 919^due to severe complications, including pneumonia, thromboembolism, sepsis, renal failure, and HF.^920 , 921^The SARS-CoV-2 infection can affect the cardiovascular system in several ways, such as inflammatory myocardial injury (myocarditis), intravascular thrombosis, and takotsubo syndrome, causing HF, arrhythmias, and circulatory shock.^922^Patients with HF have higher mortality from COVID-19 than those without HF,^923^and that rate can reach 40%.^924^Thus, previous HF is an undeniable risk factor for mortality from COVID-19.^923^

The consequences of the COVID-19 pandemic on the health of patients with CD have not been totally understood.^71^Because many of those patients have heart disease, they are vulnerable to severe infections and can have severe complications from COVID-19,^925^such as higher mortality in the presence of HF.^923^In addition, there is high prevalence of comorbidities in the population with CD, which is aging due to CD transmission control and global improvement of the health system.^926^Thus, it is possible, and even likely, that the COVID-19-related morbidity/mortality is higher in patients with CD. However, a recent comprehensive registry in Brazil has shown that the in-hospital mortality from COVID-19 was similar between patients with and without CD, paired for sex, age, hypertension, and diabetes *mellitus* , even with HF and AF being more prevalent in the group with chronic *T. cruz* i infection.^104^

The COVID-19 prevention for patients at any stage of CD follows the same recommendations for the general population presented in the Brazilian Ministry of Health guidelines, although with emphasized recommendations and special attention to vaccine indications according to the age groups, for prophylaxis of pneumococcal infections, influenza virus, and COVID-19.^927^Patients with CD have priority indication for anti-COVID-19 vaccination and are important risk groups in vaccination strategies, not only for COVID-19 but also for other immune preventable diseases with risk for the development of severe pneumonias and/or cardiac impairment.

For individuals with CD who get infected with SARS-CoV-2, medical care should be instituted since the PHC level, with emphasis on the risks associated with myocarditis and thromboembolic intravascular phenomena ( Figure 13.1 ).

**Figura 13.1:** – Flowchart recommended for the medical care of patients with Chagas disease in two situations: COVID-19 prevention and coinfection. CD: Chagas disease; ACD: acute Chagas disease; CCCD: chronic cardiomyopathy of Chagas disease; SaO2: O2 saturation; ICU: intensive care unit

In moderate or severe COVID-19 cases, corticotherapy can be used, almost always from the 6th day of disease on and for a short period of time. There is no contraindication to the use of corticosteroids for patients with CD coinfected with SARS-CoV-2 because their immunosuppressant effect, which could reactivate *T. cruzi* infection, is not reached at the recommended doses and for short periods of time. However, the management and indications should be shared by an infectiologist and a cardiologist.^928^

For patients with CCCD and mild COVID-19, the previously estabilished cardiovascular medication and anticoagulation should be maintained, because there is no indication that they are harmful. For moderate or severe cases, oral anticoagulation should be replaced by low-molecular-weight heparin and the cardiovascular medication reassessed according to the patient’s hemodynamic state.

### 13.8. Noncardiac Transplantation and Immunosuppressive Therapy

The transmission of CD through organ transplantation was described for the first time in Brazil in 1981 after renal transplantation.^929^After that, CD transmission after kidney, liver, heart, or bone marrow donation has been reported worldwide, and the transmission rate has varied according to the transplanted organ: 13%^930^to 16%^931^for kidney, 20%^930^to 22%^932^for liver, and 75% for heart.^930^In addition, RCD has been detected in patients with chronic CD recipients of solid organs.

The largest experience is in renal transplantation, in which RCD occurs mainly in the first year and varies among centers from 8% to 22%.^933 , 934^Regarding liver transplantation, the experience is limited, and the incidence of RCD varies according to the center similarly to that in renal transplantation.^934^For bone marrow transplantation in patients with asymptomatic chronic CD, the RCD has ranged from 17% to 40%.^583^

In Brazil, the Ordinance n^o^2600 from 2009 determines testing for CD: (1) in all donations, with the same algorithms used for blood donor screening; (2) for registration of potential organ recipients in the Unified Technical Register; and (3) in all dead donors of organs, tissues, cells, or body parts prior to graft allocation. That ordinance establishes that the heart from a donor with CD should not be used for transplantation, while the kidneys, pancreas, liver, and lungs of donors with CD can be transplanted as long as authorized by the recipient and transplant team, despite the risk of transmission and implicating in need for post-procedure monitoring.

#### 13.8.1. Donor with Chagas Disease and Recipient without Chagas Disease

In this situation, there is risk for CD transmission. Living donors with CD should be ideally treated with benznidazole for 60 days before the procedure. If there is no time for treatment completion, the transplantation can occur after 14 days of treatment,^2 , 651 , 934^based on the parasitemia drop in that phase of treatment.^587^

For untreated donors, monitoring the occurrence of CD transmission is recommended, as well as treatment of the diagnosed cases, when good results are observed with a high rate of cure.^2 , 583 , 931 , 935 , 936^However, the prophylactic use of benznidazole, which would be routinely applied, is controversial, considering its toxicity and the low transmission rate.

Monitoring is performed with the direct search for *T. cruzi* in peripheral blood weekly for up to 60 days, in addition to indirect parasitological and serological tests at 30 and 60 days after transplantation. Then, clinical, serological, and parasitological (direct/indirect/PCR) tests should be performed every 2 months up to 1 year of follow-up; after that, every 6 months, while immunosuppression, whose duration depends on the transplant modality and type, persists. In addition to the usual monitoring efforts, any suspicious clinical sign of acute CD should be investigated by use of parasitological tests.

PCR can be used in the place of indirect parasitological tests.^2^At any time, if acute infection is detected, conventional antiparasite treatment should be instituted.^2^In addition, it is worth noting that the serological tests might not turn positive because of the patients’ immunosuppression. Monitoring should be more frequent right after transplantation, because, in most cases, transmission and acute infection occur between the 3rd and 29th weeks (mean of 8 weeks).^930^

A study comparing 13 patients not correctly monitored with 19 who underwent weekly monitoring has shown that 5 patients in the first group were diagnosed with symptomatic CD, 4 of whom died, while, in the other group, 4 transmissions were confirmed, the patients received antiparasite treatment and did not develop symptomatic disease.^930^The parasitological tests should be performed weekly during treatment or up to two consecutive negative tests are obtained.^934^

#### 13.8.2. Recipient with Chagas Disease

The prevalence of CD among candidates for solid organ transplantation is higher in the heart group because of the CD specific characteristic of progressing to refractory HF in many cases.

Although the RCD can occur during the immunosuppression period following any solid organ transplantation, severe forms of that complication, such as meningoencephalitis^937^and intracerebral tumor-like lesions (“ *chagomas* ”),^938^are not common. In renal transplantation recipients, RCD occurs mainly in the first year after the procedure or when the immunosuppression intensifies after rejection episodes. The RCD can be totally asymptomatic but, when clinical manifestations appear, they usually consist of the subcutaneous lesions (erythema nodosum-like, panniculitis) in the limbs. If the treatment is not initiated, the lesions can progress to painful ulcers. Myocarditis and encephalitis are also described but less frequently. The response to treatment is good, with suitable long-term survival of the patient and graft.^933^

There are two managements for transplant recipients already diagnosed with CD: treat before transplantation the already infected recipient or remain vigilant to diagnose and treat an eventual RCD. The routine treatment of asymptomatic recipients with CD before undergoing transplantation could theoretically reduce the chance of an RCD after immunosuppression; there is no conclusive evidence, however, favoring that statement and the correspondent prophylactic management. On the contrary, failure of that management has been reported.^939^

Thus, the preferred management is the routine monitoring of parasitemia and other RCD evidence, so that early specific treatment can be initiated, increasing the treatment success with a smaller number of severe and fatal cases.^2 , 934^In addition, the result of the treatment of RCD is usually favorable, with high rates of cure and low mortality.

All *T. cruzi-* infected recipients should be followed up for the investigation of RCD once a week in the first 2 months, every 2 weeks from the third to the sixth month, and, after that, monthly up to 1 year. After intensification of immunosuppression, the follow-up should be weekly for 2 months, or at any time if acute CD is clinically suspected.^934 , 940^

Direct parasitological tests are the preferred laboratory tests. The qPCR is the one to be used, because the qualitative one can be positive in asymptomatic patients. The qPCR is more sensitive and turns positive earlier than the direct parasitological methods.^401^Nests of *T. cruzi* amastigotes should be searched in all biopsies. The RCD is diagnosed by the identification of parasites in the peripheral blood by use of direct methods or qPCR, as already described, or *T. cruzi* identification in biopsies. The RCD should be considered in patients with unexplained fever, dermopathy, myocarditis, or encephalitis.

All infected recipients should be investigated once a year for the cardiac and digestive forms of CD. All individuals with RCD should be treated for 60 days with benznidazole (5mg/kg/day), nifurtimox (8mg/kg/day) being the second choice. During treatment, parasitological tests should be performed weekly until two negative tests are obtained.^934^

It is worth emphasizing that serological tests are not useful for the diagnosis of RCD and that negative seroconversion has been reported in patients with chronic CD after being transplanted because of immunosuppression.^931^

It is still uncertain whether the use of specific protocols of chemotherapy drugs can influence the RCD. Thus, avoiding the use of antithymocyte globulin and minimizing the use of mycophenolate seem recommendable. Some studies have suggested that mechanistic target of rapamycin (mTOR) inhibitors could favor the control of *T.* cruzi replication,^941^thus being a more appropriate regimen for patients at risk for CD. However, an optimal regimen has not been established.^655^

#### 13.8.3. Autoimmune Diseases

The experience with CD associated with autoimmune diseases is scarce and limited mainly to case reports, most of them related to systemic lupus erythematosus.^942^Thus, surveillance for RCD and proper treatment are recommended. In addition, there is no evidence favoring the prophylactic use of benznidazole prior to corticosteroid at immunosuppressive dose. Monitoring possible RCD is the best management.

## 13.9. Chagas Disease and Aging

The combination of successful public policies to control CD transmission, the increase in life expectancy of Brazilians, and the improvement in housing conditions in endemic regions has changed the patients’ profile, propitiating an increase in the mean age of *T. cruzi* chronically infected individuals.^926 , 943^However, *T. cruzi* infection remains an independent predictor of all-cause mortality and stroke in the elderly.^70 , 849^These are new challenges for the care of patients with CD, when the degenerative diseases of the elderly, SAH, diabetes *mellitus* , dyslipidemia, and coronary artery disease, compound the injury to the heart caused by CD, thus influencing the prognosis and quality of life of that population.

At the same time, information on how CD presents in the elderly is scarce, because most longitudinal studies have been performed a long time ago in predominantly young populations.^404^Cross-sectional studies conducted in the states of Ceará,^944^São Paulo (city of Campinas),^523^and Rio de Janeiro^926^in elderly individuals with CD on outpatient care have reported SAH as the most frequent comorbidity.

In addition to SAH, other comorbidities have been reported, such as dyslipidemia, osteoporosis, osteoarthritis, diabetes *mellitus* , HF, coronary artery disease, hypothyroidism, dyspepsia, depression, stroke, and renal failure. Thus, those patients require special attention. Moreover, chronic comorbidities can result in frequent visits to the medical office and risk for drug interactions, adverse effects, and the daily use of five or more medications difficult to be correctly managed by the elderly.^945^

In the cross-sectional studies mentioned, cardiomyopathy was the predominant clinical form of CD; however, information on the prognostic value of the changes in the elderly is still scarce.^946 , 947^In a study conducted in Bambuí, a cohort of elderly with and without CD, electrocardiographic changes were clearly more frequent in patients with CD.^303^The ECG abnormalities significantly associated with CD were sinus bradycardia, frequent ventricular or supraventricular extrasystoles, AF, RBBB, LAFB, 1^st^-degree AVB, and prolonged QT interval.

The RBBB, especially when associated with LAFB, was strongly associated with the presence of CD, being observed in 40% of the population with CD and in only 8% of the elderly without CD. The ECG variables independently associated with increased risk of death in patients with CD were frequent ventricular or supraventricular extrasystoles, AF, RBBB, inactive electrical zone, primary alterations of ventricular repolarization, and LV hypertrophy. Those with normal ECG findings or mild changes did not have increased risk of death as compared to the noninfected population.^303^

Many elderlies do not have either an initial clinical assessment to classify CD or appropriate follow-up care and treatment. This can be seen in the cross-sectional study conducted in the endemic area of São João do Piauí, in the Brazilian semiarid region. That study has evidenced high prevalence of CD in the elderly, which reached up to 34% in the age group of 61-75 years and 39% in the age group over 75 years. In that region, despite the disease transmission control, the diagnosis and treatment were suspended, and many elderlies never had an initial clinical assessment. That region, as well as others with socioenvironmental characteristics similar to those of the Brazilian semiarid region, continues to suffer with the scarcity of PHC groups trained to diagnose and treat the population.^948^

Classical studies in endemic areas have shown that the IFCD is the most prevalent form of CD and that 30% to 40% of the individuals can indefinitely persist in that clinical form.^300^In contrast, a study has reported that only 13% of the elderlies have normal ECG findings, suggesting that the severity of CD in the elderly can be similar to that in young adults.^303 , 947^This information requires further investigation for substantiation and confirmation.

## 14. Recommendations for Implantation of Structured Health Services for the Follow-Up of Individuals with Chronic Cardiomyopathy of Chagas Disease

Considering the impact of social, economic, and cultural factors on the genesis and evolution of CCCD, its clinical management in healthcare services requires the formation of a care network in a model that transcends biomedical dimensions. Thus, it should ensure access to comprehensive, hierarchical, and decentralized care, encompassing the social determination process that permeates this neglected disease, cause and consequence of structural poverty.

As already described, individuals with CCCD have high morbidity and mortality as compared to those with other cardiomyopathies. Most of them belong to underprivileged social classes and are highly vulnerable, which hinder their access to diagnosis and treatment.^949^Regarding healthcare provision, the conditions of individuals with CCCD are usually critical, which include long treatment with a low rate of success, and late diagnosis, usually in advanced stages of disease.

In addition, patients with CCCD face bias and stigma in different social contexts, which compounds even more their not only physical, but psychological and social suffering. Chagas disease is among the most neglected diseases worldwide, especially in Latin America, according to the WHO. It is a challenging chronic condition for any public health system because the affected individuals can demand health actions from low/medium technological complexity, in approximately 70% to 80% of the cases (mainly in PHC), up to access to tertiary and quaternary care, increasing public health-related costs. It is worth emphasizing the critical negative impact on the quality of life of affected individuals, their families, and communities.^950 - 952^

In Brazil, from 2000 to 2010, the CCCD burden corresponded to a total of 7 402 559 potential years of life impaired, 9% of which due to years of life lost and 91% due to years lived with disability.^953^

The SUS, in its hierarchical and decentralized conception, has been designed to reach comprehensiveness as a referential, particularly for PHC territories, with support from the matrix, such as referral services for more complex cases. However, it requires investments in addition to qualified and engaged public management that enables the formation of a care network grounded in lines of care strongly integrated with health surveillance actions.

There are some factors that can explain the persistence of the sanitary gap experienced by individuals with CD, even 113 years after CD discovery. As an example of the cycle of negligence, CD affects a silent and silenced population that faces persistent science, market, and public health challenges.

Basic questions remain unanswered in the endemic context: Who are these individuals? Where are they? How are they?^951 , 954^

In more complex clinical management contexts, when recommending the formation of structured services for the follow-up of individuals with CCCD, some aspects require attention, such as an appropriate outpatient space affiliated to a tertiary or quaternary hospital with cardiological assistance that can provide additional intermediary to high complexity tests for proper cardiac staging.

In addition, regarding access to better structured services, it is worth noting the need for follow-up of individuals living in challenging areas, such as the Amazonian region, rural areas, and urban peripheries. In such cases, the use of differentiated technological media, such as teleconsultation and remote ECG and chest X-ray reporting, might be necessary.

Structured health services in CCCD might become a regional and state reference for cases requiring more complex clinical management, aimed at diagnosis clarification and staging of organ impairment. In addition, those services might support state and municipal permanent education programs for PHC professionals (considering the entire health team), which include communitarian health and endemic combat agents, for the clinical management of CD, which, although endemic, is still underdiagnosed.

For a structured health service to be fully functional, it requires an interdisciplinary multiprofessional team, known as the best way to provide longitudinal and comprehensive care to chronic diseases. In addition to timely diagnosis and treatment, those diseases require rehabilitation and quaternary prevention.

When creating a health service dedicated to the management of individuals with CCCD, it is important to contemplate their peculiarities, understand their biopsychosocial context, and practice patient-centered, instead of disease- or organ-centered, medicine.

In that work model, the team should recognize the common elements that demand strong interaction between the professionals, as well as the work process specificities delimited by their acting possibilities and responsibilities. In addition, the team should have knowledge of CCCD, as well as its management, so that all can communicate well. This is meant to avoid distorted or untrue information.^951 , 955 , 956^

A structured service for management of CCCD cases should ideally count on the following professionals: physicians (cardiology, internal medicine, infectious disease, gastroenterology), nurses, psychologists, nutritionists, pharmacists, physical therapists, physical educators, and social workers. The team can be enlarged with the adoption of new people responsible for other interventions, and its dimension should be adjusted to the local reality, the possibilities of each health service, and, most of all, the demands from affected individuals.^951 , 955^

### 14.1. Assignments of Structured Health Services for the Follow-up of Individuals with Chronic Cardiomyopathy of Chagas Disease

1. Receive all cases proceeding from PHC units, secondary health care units [specialized emergency care units (UPAE)], cardiological and non-cardiological emergencies, maternity hospitals, public or private blood centers, transplant services, as well as HIV/AIDS specialized services, for CD diagnosis and staging;
2. The CD diagnosis confirmation requires qualified anamnesis, directed at the clinical epidemiological context, with serological confirmation, preferably by the LACEN;
3. According to the Ministry of Health Ordinance nº 1.061, from May 18, 2020, diagnosed chronic cases of CD must be reported (compulsory notification) to improve the INSS network organization for the chronic CD prevalence in Brazil (Brazilian Ministry of Health);^957^
4. Stage cardiac impairment, by use of additional tests, maintaining communication with the Basic Healthcare Units (UBS) and UPAE, in a decentralized way, to accomplish referral and counter-referral flow. Individuals with the IFCD or nonsignificant cardiac impairment can be followed up in the UBS close to their dwellings, thus reducing the demand for treatment in other centers;
5. Individuals with CD and indication for etiological treatment should be managed according to the recommendations in this guideline’s specific chapter and followed up in a UBS as long as the health team is trained in the clinical management of these cases;^957^
6. Women at reproductive age should be educated about the possibility of congenital transmission of CD and contraceptive methods. If they want to get or are already pregnant, they should be followed up by the PHC team in association with a referral obstetrics service and be treated according to the current guidelines;^957^
7. Patients with CD and HF, complex arrhythmias, need for PM or ICD implantation or CTX should be followed up at a higher complexity service. In some cases, the use of MCSD might be necessary as a bridge to CTX or an alternative to CTX if with good results;^8^
8. Identify associated digestive organic and functional impairment, and, when present, treat or refer the patient to a CD specialized service;^957^
9. Treat comorbidities or assess the need to refer patients for medical assessment at specialized services;^951^
10. Cardiac rehabilitation should be integrated into structured health services of care to individuals with CCCD because of the proven clinical benefit of supervised physical exercise for their health and quality of life;^897^
11. Individuals with difficulty to understand the prescriptions of the health team should be helped by a pharmacist from the multiprofessional team to clarify dosage, interval between doses, adverse events, drug interactions, and strategies;^552^
12. Provide permanent education (presential or virtual) to affected individuals, their families, and caregivers on the disease and selfcare to the timely identification of cardiac decompensation signs and symptoms, making a communication channel (for example, *DISC Chagas* , *DISC IC* ) and social media information available. With the dissemination of cell phones and the internet, remote care has proven to be greatly important in the management of more severe patients who cannot wait for a consultation or for small adjustments, which has been confirmed during the COVID-19 pandemic;
13. Educate on intracardiac devices, their functions, and need for PM or ICD implantation, as well as CTX, aiming to eliminate myths and beliefs that can negatively impact on the quality of life and adhesion to the treatments proposed, as well as on the possibility to donate blood, organs, and tissues;^951^
14. Value the knowledge of affected individuals about their own disease, inviting them to participate in educational meetings, enabling the exchange of experiences, potentiating autonomy and empowerment, and encouraging changes from a passive to an active behavior regarding their therapeutic process and demands;^958^
15. Promote group meetings with specific themes, such as nutritional aspects, physical activity, depression, rights of individuals with chronic diseases, medico-legal aspects, transportation aid, social security, sexuality, pregnancy, breastfeeding, myths and truth related to CD;^951^
16. Provide psychological support to reduce stigma, self-prejudice, taboos, and inappropriate beliefs regarding CD. Provide information on the prevention of aggravating factors, such as alcohol, smoking, illicit and licit drugs;^951 , 958^
17. Develop continuing education programs for health professionals, with specific focus on the CCCD characteristics, stimulating multiprofessional learning and research;^951 , 958^
18. Identify, through active search and doctor-patient relationship deepening, other family members in the same context of risk for *T. cruzi* exposure (including the possibility of congenital transmission) and incorporate the confirmed cases to the health service to determine the treatment to be adopted;^951^
19. Encourage and support the creation of new associations of individuals with CD aimed at their better integration, establishing an active and purposeful communication channel with society, particularly the scientific, political, and health community, regarding their right to health claims. This would represent a strong channel in the search for active citizenship to transform their pain and suffering into a political act;
20. Always support the fight against prejudice, such as the necessary exclusion of the adjective ‘Chagasic’, which belittles the individuals affected by the disease. In clinical practice, it means to replace the term ‘Chagasic’ by ‘individual affected with CD’;^951 , 954 , 959^
21. Publicize the existence of the FINDECHAGAS federation, created in 2010, as well as of the ‘14th of April’ as the CD World Day, recognized by the WHO in 2019;^954^
22. Create telemedicine services for medical consultations and reporting of additional tests, such as ECG and chest X-ray. Based on this remote assessment, refer selected cases for management at structured health services.

### 14.2. Expected Benefits of Structured Health Services for the Follow-up of Individuals with Chronic Cardiomyopathy of Chagas Disease

Structured referral services for the follow-up of individuals with CCCD will be able to confirm what has been described for other chronic diseases.^955^Once structured, the service is expected to be able to provide:

Strengthening of the relationship between health professionals and individuals with CD;
Development of active listening of individuals with CD and counseling about CD;
Updated knowledge on the disease for healthcare professionals and affected individuals;
Means to favor adhesion to pharmacological and nonpharmacological treatment;
Means to lower morbidity and mortality, thus reducing emergency visits and rehospitalizations;
Positive impact on quality of life; several recent studies have emphasized this relevant concept supported by coherent data;^43 , 515 , 960^
Stigma and prejudice reduction;
Empowerment, autonomy, and motivation of affected individuals to develop selfcare and search their rights (health, education);
Reduction in public health costs.

Although the implantation of a structured service requires financial as well as technical and operational investments, its structuration in a healthcare network is believed to favorably impact medium- and long-term cost and effectiveness.

Briefly, the major mission of structured services is to promote care that favors the clinical, psychological, and social stability of all individuals with CD.

## 15. Definition of Severe Cardiopathy and Medico-Legal Assessment

### 15.1. Introduction

Chronic cardiomyopathy of Chagas disease, still prevalent in Brazil, can progress to HF, ventricular arrhythmias, electrical conduction disorders, stroke, and other thromboembolic, pulmonary, and systemic complications, which represent severe situations,^7^sometimes with social and work implications.

The term “severe cardiopathy”, created by a multidisciplinary team, appeared for the first time in the Brazilian legislation in 1952 in the statute of the union civil servants, as the law 1711 (item 11, article 178). Severe cardiopathy was defined as “a disease that leads to a temporary or permanent reduction in the heart’s functional capacity, which can be life-threatening or prevent servants from doing their work activities”.^961^According to that document, the medico-legal expert had to rely on subjective data to conclude their diagnostic investigation. However, with the advances of medico-legal medicine, based on better knowledge of the clinical course and prognosis of patients with CCCD, in addition to advances related to complementary methods to diagnose cardiovascular dysfunction, the characterization of cardiopathy as a morbid entity has evolved, requiring diagnosis supported by strict clinical assessment and laboratory confirmation, according to the SBC II Brazilian Guideline on Severe Cardiopathy, published in 2006.^962^

### 15.2. Concept and Scope

The term “severe cardiopathy” can be found in several legal processes, according to the Federal Law nº 7713/1988, article 6th, item XIV.^961^Severe cardiopathy comprises a large group of illnesses and clinical conditions of cardiac origin, characterized by a significant reduction in the survival perspective or significant limitation in physical capacity or both. The typification of severe cardiopathy is mainly aimed at complying with work issues (such as disability retirement, changes in responsibility, and workplace adaptation) or providing financial benefits (release of FGTS and PIS/PASEP) and tax benefits (exemption from income tax as described in the normative ordinance Nº 1174/MD of the Defense Ministry Manual, of September 6, 2006, chapter III), or income increasing (25% increase in the retirement value due to conditions requiring a caregiver).

In addition, it is important to clarify that the severe cardiopathy *status* is only defined after no satisfactory response to the appropriate clinical or surgical treatment, when recommended, or when there is no satisfactory therapy, or, even if there is, it is not sufficient to change the individual’s clinical condition and prognosis.

Occasional changes in additional tests do not automatically imply diagnosis of severe cardiopathy. The verification of functional limitations and prognostic assessment result from a comprehensive investigation and contextualization of the clinical scenario of a patient with cardiopathy. Among the major inclusion criteria in the roll of severe cardiopathies, complete clinical assessment should be ensured, to provide information on the patient’s physical capacity and, in parallel, information on the estimated survival rate for the condition in question should be obtained.

Complete clinical assessment is obtained through medical consultation with detailed anamnesis and physical examination, complemented with tests, such as ECG, chest X-ray, Doppler ECHO, 24-hour Holter, exercise testing, or cardiopulmonary exercise testing. In specific situations, more sophisticated or invasive tests might be necessary, such as myocardial scintigraphy, CMRI, coronary CT angiography, or coronary angiography.^963^The information on survival derives from the level of evidence of the risk of death, which can be obtained, in the specific case of CCCD, by using scores validated and published in specialized journals.^408 , 474 , 964^

### 15.3. Score to Predict the Risk of Death in Patients with Chronic Cardiomyopathy of Chagas Disease

The CCCD course is variable and unpredictable, and one of its presentations is death, which can either be sudden, due to HF progression or result from thromboembolic phenomena. Therefore, estimating the risk of death of patients with CCCD is a clinical challenge and has been facilitated with the introduction of a score developed with that purpose.

That is the score created by Rassi Jr. *et al* ., published in 2006, when following up a cohort of 424 patients with CCCD.^408^During the study period, approximately 8 years, 130 patients died. Those authors identified six variables associated with death: NYHA functional class III or IV = 5 points; evidence of cardiomegaly on chest X-ray = 5 points; global or segmental LV dysfunction on ECHO = 3 points; NSVT on 24-hour Holter = 3 points; low-voltage QRS on ECG in all frontal leads = 2 points; and male sex = 2 points. Based on this score, those authors defined three risk categories: low risk (0-6 points); intermediate risk (7-11 points); and high risk (12-20 points). The 10-year mortality in the three groups was 10%, 44%, and 84%, respectively.^408^

By using the RASSI score, the work of the medical expert can be easily parameterized, translating into numbers the patient’s clinical reality. Thus, a RASSI score ≥ 12 points certainly indicates severe cardiopathy. However, it is worth noting that the SBC II Brazilian Guideline on Severe Cardiopathy, published in 2006,^962^which currently supports the medico-legal expert diagnosis of severe cardiopathy, is based rather on the patient’s physical capacity/quality of life after usual therapeutic resources have failed, than on the risk prediction science. Despite the importance of the clinical findings and functional class, the search for new prognostic tools to refine clinical data is fundamental to subsidize better medico-legal investigations and their conclusions.

The need for urgent review of that guideline is noticeable so that the usefulness of those scientific advances on the release of medico-legal investigative reports about patients with CD can be fully debated.

### 15.4. Clinical Aspects

The most characteristic clinical aspects of CCCD are congestive HF, complex ventricular arrhythmias requiring ICD implantation, thromboembolic phenomena, and severe impairment of liver and kidney functions secondary to the underlying heart disease. It is worth noting that it is crucial to assess the physical functioning of those patients regarding their life expectancy reduction despite the optimized therapeutic arsenal to classify them as having severe cardiopathy caused by CD.

### 15.5. Medico-Legal Expert Assessment

The medico-legal expert is a professional trained to assess and provide (or not) the *status* of severe cardiopathy to individuals who seek the social security to receive benefits because of their typification. To become an efficient medico-legal expert, in addition to an academic education in the health area, the professional must take training and specialization courses. There are several manuals for that correct practice. In addition, it involves embracing the law. Severe cardiopathy caused by CD is usually within the severe cardiopathy spectrum and supported by three laws, which refer to the following legal regimens: unified legal regimen (law nº 8.112/90); social security regimen (law nº 8.213/91); and taxation regimen (law nº 11.052/04).

From the didactical viewpoint, severe cardiopathy can be classified as follows: 1) acute cardiopathies, of rapid progression, that can gradually turn into chronic cardiopathies, characterized by the loss of patient’s physical ability and of heart functional capacity; 2) chronic cardiopathies, characterized by progressive limitation of the physical aptitude and heart functional capacity, exceeding the limits of efficiency of the cardiac compensation mechanisms, regardless of the appropriate clinical and/or surgical treatment adopted; 3) chronic or acute cardiopathies requiring permanent inotropic pharmacological (dopamine, dobutamine) or mechanical (intra-aortic balloon, biopump) support; and 4) terminal cardiopathy, when life expectancy is extremely reduced, nonresponsive to any type of therapy.

Differently from the medical board, the medico-legal expert acting results from the routine work of one single expert, designated to assess whether the severe cardiopathy *status* applies to a certain individual. This function requires emotional balance (not to be influenced by aspects other than the specific criteria) and discernment (to extract the elements to support typification of the clinical findings amidst a large number of documents). The expert, based on the medical report and additional tests, reassesses the individual to validate or not the severe cardiopathy condition litigated. A LVEF lower than 40% on optimized medication, is usually one of the major functional parameters adopted. Usually, a more detailed assessment is necessary to investigate all aspects of the clinical findings and of the additional tests because borderline situations occur, with discordance between clinical findings and diagnostic methods, divergent test results, or need for aditional equally relevant data for a proper decision.

In cases of discordance or divergence in the criteria selected for classification, and having the expert denied the presence of the disease, the judicial pathway is the natural choice in the presence of sufficient documentation.

In addition to a RASSI score ≥ 12 points, other important information to indicate the possible diagnosis of severe cardiopathy in patients with CCCD is as follows: isolated NYHA functional class III or IV; repeated episodes of syncope with no possibility of definitive control; presence of VT, mainly if symptomatic or requiring emergency care; marked cardiomegaly; and presence of cardiac thrombus or previous thromboembolic episodes.^965^

It is worth noting that the presence of symptomatic sinus node dysfunction or advanced AVB (Mobitz II, 3:1, 4:1, etc, and TAVB) not necessarily implies permanent functional limitation, because PM implantation might reverse the clinical findings and significantly improve the prognosis, particularly when those alterations occur in isolation. However, in CCCD, mainly in advanced stages, bradyarrhythmias and advanced blocks associated with myocardial dysfunction or complex ventricular arrhythmias are common, indicating more severe impairment. In such cases, a comprehensive cardiac assessment, as previously suggested, allows the medico-legal expert to identify the patient’s actual situation in terms of definitive limitation, regarding both function and prognosis.

Similarly, the mere presence of positive serology for CD or its association with an electrocardiographic change, such as RBBB, is not sufficient to characterize severe cardiopathy. Although some of those individuals are known to progress to disabling forms, most can remain for decades in that stage, without symptoms, or even complete their life without clinical worsening.

For the medico-legal investigation to be fully evaluated, the assistant physician should issue detailed reports describing precisely and clearly the patient’s clinical findings and add the tests that confirm the diagnosis.

### 15.6. Conclusion

The definition of severe cardiopathy is currently facilitated by advances of the knowledge on parameterized clinical evolution, clinical therapy, and additional tests, most of them with scientific support in terms of prognosis. This information, qualified and organized as scores developed in Brazilians, is highly valuable to support the medico-legal expert in their assessment. However, the physician’s clinical rationale should be fully used to aggregate characteristic signs and symptoms, as well as data from the additional tests performed.
